# DNA methylation changes following narrative exposure therapy in a randomized controlled trial with female former child soldiers

**DOI:** 10.1038/s41598-021-98067-9

**Published:** 2021-09-16

**Authors:** Samuel Carleial, Daniel Nätt, Eva Unternährer, Thomas Elbert, Katy Robjant, Sarah Wilker, Vanja Vukojevic, Iris-Tatjana Kolassa, Anja C. Zeller, Anke Koebach

**Affiliations:** 1 Department of Psychology, Centre for Psychiatry, University of Konstanz, Feuerstein-Strasse. 55, Haus 22, 78479 Konstanz, Germany; 2 Division of Neurobiology, Department of Biomedical and Clinical Sciences, University of Linköping, Building 463, Room 12.023, Linköping, Sweden; 3 Child- and Adolescent Research Department, Psychiatric University Hospitals Basel (UPK), University of Basel, Basel, Switzerland; 4 Vivo International E.V., Postbox 5108, 78430 Konstanz, Germany; 5 Department of Psychology and Sports Science, University of Bielefeld, 33501 Bielefeld, Germany; 6 Psychiatric University Clinics, Transfaculty Research Platform, University of Basel, Wilhelm Klein-Strasse 27, CH-4012 Basel, Switzerland; 7 Department of Clinical and Biological Psychology, Institute of Psychology & Education, University of Ulm, Ulm University, Ulm, Germany

**Keywords:** Human behaviour, DNA methylation

## Abstract

The aftermath of traumatization lives on in the neural and epigenetic traces creating a momentum of affliction in the psychological and social realm. Can psychotherapy reorganise these memories through changes in DNA methylation signatures? Using a randomised controlled parallel group design, we examined methylome-wide changes in saliva samples of 84 female former child soldiers from Eastern DR Congo before and six months after Narrative Exposure Therapy. Treatment predicted differentially methylated positions (DMPs) related to *ALCAM*, *RIPOR2*, *AFAP1* and *MOCOS*. In addition, treatment associations overlapped at gene level with baseline clinical and social outcomes. Treatment related DMPs are involved in memory formation—the key agent in trauma focused treatments—and enriched for molecular pathways commonly affected by trauma related disorders. Results were partially replicated in an independent sample of 53 female former child soldiers from Northern Uganda. Our results suggest a molecular impact of psychological treatment in women with war-related childhood trauma.

**Trial registration**: Addressing Heightened Levels of Aggression in Traumatized Offenders With Psychotherapeutic Means (ClinicalTrials.gov Identifier: NCT02992561, 14/12/2016).

## Introduction

Former child soldiers, particularly female combatants, are at high risk of experiencing multiple, severe and traumatic stressors including violence and repeated rape. These extreme and intense stressors produce lasting changes in mind and body leading to a range of mental disorders. These include posttraumatic stress disorder (PTSD), depression, dissociative symptoms and high levels of aggression against oneself and others^1,2^. Frequent exposure to severe stressors is decisively detrimental. Such exposure reorganises the functioning of the brain and mind in a lasting, self-perpetuating manner such that simple cues, sometimes arising from imaginative processes alone, may activate a physical response as part of the defence cascade^3^. Most importantly, the hypothalamic–pituitary–adrenal (HPA) axis may be tilted^4–6^, resulting in anything from a long-lasting immune problem^7–9^ via deficient metabolic systems^10–12^ to the broad trauma related psychopathology maintained by the intrinsic dynamics of traumatic memories.

These processes are realised through epigenetic modifications in neuronal and peripheral tissues, and in particular have been associated with DNA methylation (hereafter DNAm)^13,14^. For example, PTSD and depression have been associated with DNAm changes in blood and buccal cells at genes involved in glucocorticoid functioning, such as the FKBP prolyl isomerase 5 (*FKBP5*) and the nuclear receptor subfamily 3 group C member 1 (*NR3C1*)^15–19^. Emerging evidence suggests that these associations could even be transferable across generations^20–22^, although studies in mammals are still scarce^23^. Aggression has also been associated with multiple differentially methylated regions (DMRs) in at least 30 gene promoters^24^. Likewise, antisocial personality disorder has been associated with DNAm changes in the monoamine oxidase A (*MAOA*) and B (*MAOB*)^25^, two genes relevant in the dopaminergic and serotonergic pathways.

It is yet to be seen whether these epigenetic marks can be modified through treatment such that the clinical symptoms may recede. A few studies with small sample sizes that investigated only a limited number of target genes nonetheless provide early evidence that this may be possible^26–28^. However, so far, there have been no randomised controlled trials using an epigenome-wide association approach to investigate the impact of successful trauma treatment in any sample, let alone in children of war. Given that childhood experiences are important for both aetiology and epigenetic predisposition of mental disorders^29–31^, investigating therapeutic impacts on the epigenome in patients with childhood and war-related trauma may support further development of treatment for mental disorders and contribute to our understanding of therapeutic agents.

We examined a subsample of 84 highly traumatized female former child soldiers who participated in a randomised controlled trial^32^ using an epigenome-wide approach (EWAS) that investigated associations between differentially methylated positions (DMPs), trauma related mental disorders and social consequences as well as Narrative Exposure Therapy (NET)^33^ in comparison to a less effective treatment [step 1]. NET is one of the empirically supported psychotherapies for trauma-related disorders^34^, alongside CPT (Cognitive Processing Therapy), EMDR (Eye Movement Desensitization and Reprocessing) and PE (Prolonged Exposure Therapy). It particularly targets survivors of multiple traumatic stressors. Recent adaptations of NET have successfully treated trauma related suffering, focusing on PTSD, depression and appetitive aggression, and mitigated social problems such as current violent behaviour and perceived social acknowledgment as victim or survivor^32,35,36^. Then, we identified overlap in associated DMPs [step 2] and further investigated outcome and treatment associations using gene-ontology and gene-network enrichments to determine potential functions [step 3]. Finally, we used an independent cohort of 53 female former child soldiers from Northern Uganda who also had been successfully treated using NET to replicate our findings [step 4].

## Results

**Figure 1:**
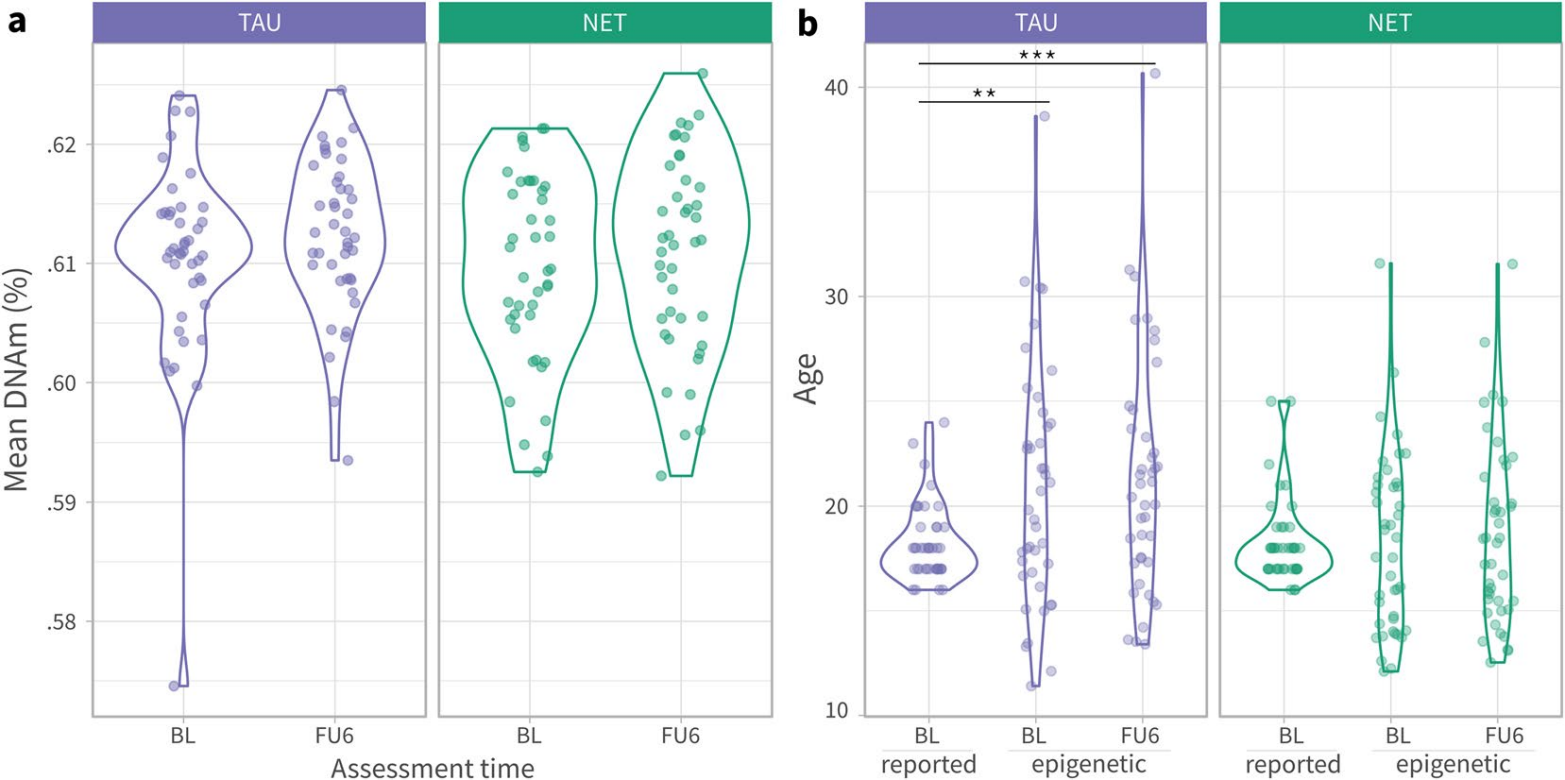
Violin plots showing individual observations of discovery sample. (**a**) Mean DNAm observed over all estimates (average Beta value) of probes used in EWAS. (**b**) Mean reported age and mean epigenetic ages. Points represent study participants. Colors represent treatment condition (TAU vs. NET). Assessment time refers to baseline (BL) and 6 month follow-up (FU6). Asterisks mark significant t-test comparisons (***p < 0.001; **p < 0.01).

At baseline, mean Beta per probe ranged from 0.030 to 0.968 with an overall mean value of 0.61 (0.609:0.611; 95% CI). Most probes (N = 305,868) had a methylation status of more than 0.5 (69.7%). Mean DNAm per participant (average Beta over all CpGs) was 0.61 (SD = 0.008). It did not significantly change over time (likelihood-ratio test; LRT = 0.78, p = 0.378) nor did it differ between treatment groups (LRT = 0.14, p = 0.078; Fig. 1A). Although participants in TAU and NET had a similar age of 18 (SD = 1.8) (t = 0.06, df = 81, p = 0.955), epigenetic age differed among groups. In TAU, epigenetic age was larger than reported age at baseline (t = 3.29, df = 41, p = 0.002) and follow-up (t = 3.83, df = 41, p < 0.001), whereas no statistical differences were found in NET (Fig. 1B). For summary statistics see Table 1.

**Table 1:** Summary statistics of the discovery and replication samples.

	Discovery sample						Replication sample				
	TAU			NET			NET				
N =	n = 42			n = 42			n = 53				
Reported age	18 (1.8)			18 (2)			34 (9.1)				
Trauma perpetrated	14 (4.9)			15 (4.7)			16 (5.2)				
Trauma experienced	15 (1.7)			15 (1.9)			34 (5.7)				

	BL	FU6	Cohen's *d*	BL	FU6	Cohen's *d*	BL	FU4	Cohen's *d*	FU10	Cohen's *d*
Epigenetic age	21 (5.8)	21 (5.7)	0.15 [− 0.21,0.46]	18 (4.2)	19 (4.3)	0.19 [− 0.12,0.5]	43 (7.2)	44 (7.9)	0.19 [− 0.12,0.48]	44 (7.7)	0.26 [− 0.04,0.54]
DNAm (Beta)	0.61 (0.008)	0.612 (0.006)	0.34 [0.04,0.63]	0.61 (0.008)	0.611 (0.008)	0.24 [− 0.09,0.55]	0.56 (0.01)	0.56 (0.01)	0.01 [− 0.28,0.28]	0.56 (0.01)	0.04 [− 0.24,0.31]
PSS-I sumscore*	38 (9.4)	22 (14)	− 1.03 [− 1.51,− 0.43]	39 (8.4)	17 (14)	− 1.51 [− 1.92,− 1.01]	16 (4.1)	7.1 (5.5)	− 1.48 [− 1.95,− 0.9]	5.9 (5.2)	− 1.56 [− 2,− 0.99]
PHQ-9 sumscore†	13 (5.3)	7 (3.6)	− 1.16 [− 1.48,− 0.79]	12 (5)	6.8 (3.8)	− 1.09 [− 1.33,− 0.78]	13 (9.7)	7.9 (8.7)	− 0.44 [− 0.75,− 0.08]	6.6 (7.6)	− 0.53 [− 0.82,− 0.19]
AAS sumscore	22 (11)	10 (12)	− 0.78 [− 1.23,− 0.24]	26 (11)	7.6 (7.8)	− 1.5 [− 1.82,− 1.13]	31 (17)	n.a	n.a	n.a	n.a
CVB sumscore	19 (7.1)	12 (9.1)	− 0.69 [− 1.04,− 0.27]	20 (5.8)	7.9 (6.3)	− 1.76 [− 2.16,− 1.25]	n.a	n.a	n.a	n.a	n.a
AAGS sumscore‡	− 0.85 (3.3)	0.28 (3.5)	0.38 [0.04,0.69]	0.03 (3.4)	0.94 (3.9)	0.2 [− 0.12,0.52]	n.a	n.a	n.a	n.a	n.a
SAQ sumscore	13 (8.8)	23 (10)	0.87 [0.51,1.2]	13 (8.9)	29 (10)	1.53 [1.03,1.93]	n.a	n.a	n.a	n.a	n.a

Sample size, reported age, nr. of traumatic events (perpetrated and experienced), epigenetic age, DNAm (Beta), and sumscores for PTSD (PSS-I), depression (PHQ-9), appetitive aggression (AAS), current violent behaviour (CVB), feelings of guilt (AAGS) and social acknowledgement (SAQ) are shown. Except for sample size, all values are reported as means with standard deviations in parentheses. Cohen’s *d* effect sizes are reported with a 95% bootstrap confidence interval in square brackets.

BL: baseline; FU4,6,10: follow-ups after 4, 6 and 10 months from baseline, respectively; *PDS Scale was used for replication sample; † HSCL Scale was used for replication sample; ‡sumscores of AAGS are scaled; n.a.: not available.

### Step 1: CpGs and genes associated with outcomes and treatment

Linear models (EWAS) showed a varying number of CpGs that were significantly associated (FDR = 0.05) with clinical and social outcomes at baseline. We found significant DNAm associations with PSS-I (DMP at cg02192673; *NPFFR2*), CVB (51 DMPs), AAGS (DMP at cg20866785; *ARHGAP10*) and SAQ (85 DMPs), but we did not find significant associations with PHQ-9 or AAS. For details on all estimates see supplement 2.

**Figure 2:**
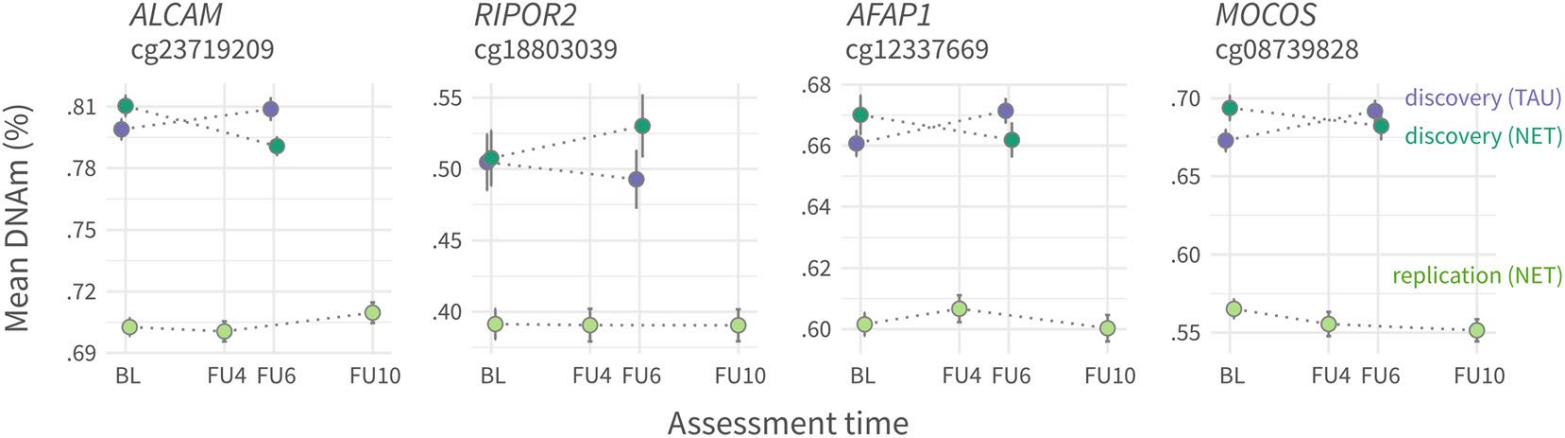
Significant associations between treatment condition and DNAm change. Mean DNAm (%) as a function of assessment time at discovery and replication sample (colour coded) for the four CpGs that significantly had an association between treatment effect and DNAm. Dots represent group means and bars show standard errors. Note that samples had different follow-up assessment times (FU4,6,10; numbers represent months) after baseline (BL). Replication sample does not support findings statistically in discovery sample.

Patients who received NET showed a significant DNAm reduction at cg23719209 (*ALCAM*), cg12337669 (*AFAP1*) and cg08739828 (*MOCOS*; promoter-linked), and DNAm increase at cg18803039 (*RIPOR2*) (Fig. 2). Linear model outputs are shown in supplement S2.

### Step 2: Intersections of associated CpGs and genes

**Figure 3:**
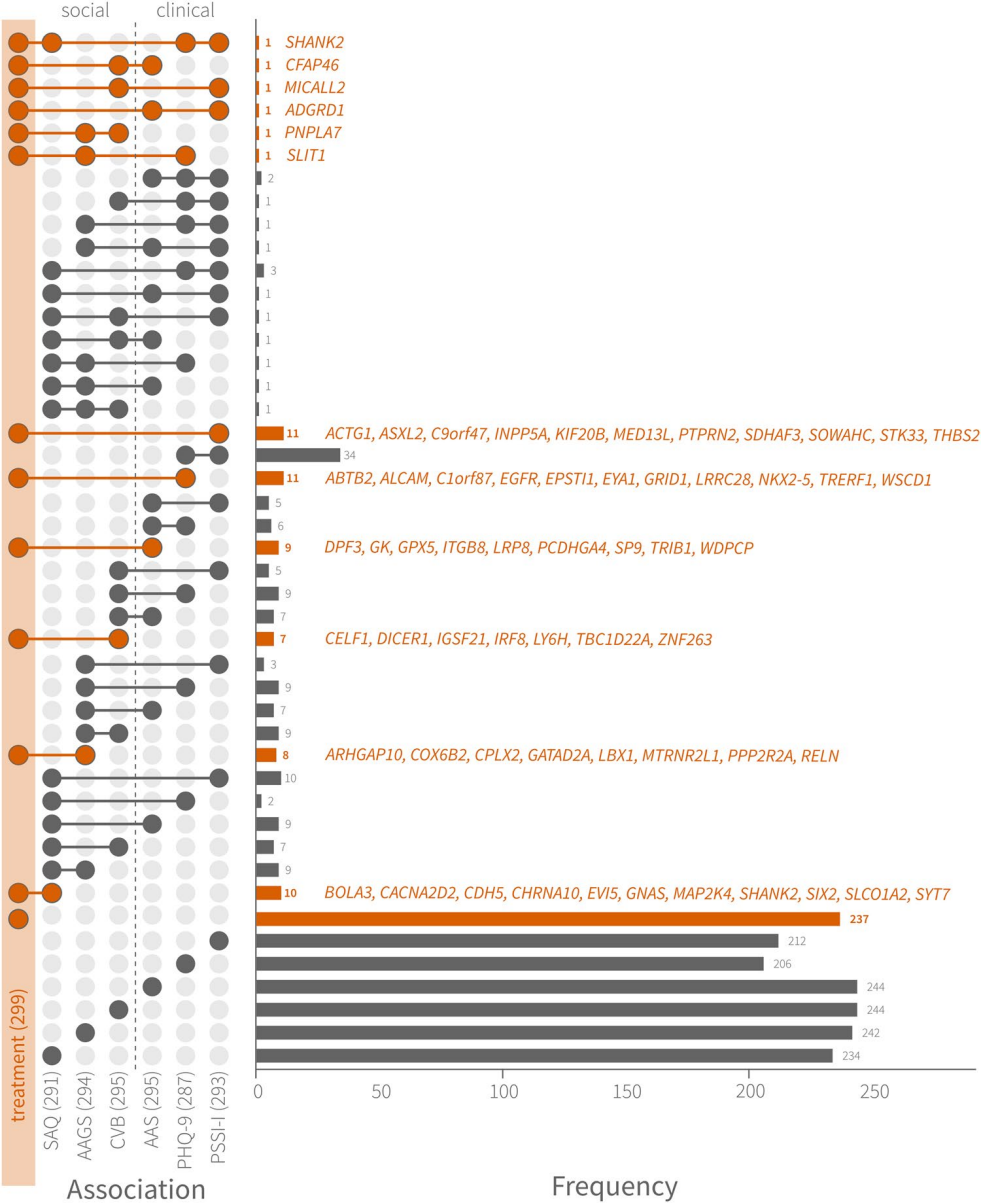
Intersection plot of genes associated with outcomes and treatment. On the left, intersections are shown as two or more circles connected by lines, whereas single associations are shown as isolated circles at the bottom. On the right, horizontal bars show the total number of intersections and single associations. Intersections with treatment are additionally specified with gene symbols. Treatment is marked in orange. Association sample sizes derive from each of the top 0.1% DMPs identified after EWAS (n = 305 per association).

Significantly associated CpGs and their respective genes were specific to each tested outcome. Only when considering the top 0.1% CpGs ranked by significance (305 probes per association), CpG sites were found to be associated with more than one outcome, with the highest intersection between PSS-I and PHQ-9 (31 CpGs). Six other CpGs were associated both with treatment and any of the mental health measures (for details, see supplement S3). Regarding gene intersections, we found again the highest overlap between PSS-I and PHQ-9 (42 genes). Various other intersections were found among associations. Importantly, a total of 62 genes were associated with treatment and one or more outcome (Fig. 3) (supplement S3).

### Step 3: Gene function and network enrichments

Genes associated with treatment and clinical/social outcomes (from top 0.1% CpGs after EWAS) were significantly enriched for multiple networks and functional pathways, after statistical overrepresentation tests in STRING (for details, see supplement S4). We did not find significant enrichment for genes associated with PSS-I. For treatment-related CpGs (PPI enrichment < 0.001), we found a gene-ontology enrichment linked to cortisol and aldosterone synthesis and secretion (strength = 0.88 and 0.77; FDR = 0.015), extracellular matrix (ECM) receptor interaction (strength = 0.77, FDR = 0.016), bile secretion (strength = 0.76, FDR = 0.027), glutamatergic synapse (strength = 0.74, FDR = 0.015), gonadotropin releasing hormone (GnRH) signalling pathway (strength = 0.73, FDR = 0.017). Further enrichment pointed to general body functions, such as nervous system and morphogenesis. Of the seven significantly enriched biological processes, three were associated with embryonic development. When considering the 62 genes intersecting between treatment and outcomes, we further found enrichment for cardiomyopathy (strength = 1.16, FDR = 0.025), oxytocin signalling (strength = 0.93, FDR = 0.043), epidermal growth factors (strength = 0.88, FDR = 0.035) and several biological processes and cellular components (supplement S4).

### Step 4: Replication

For *ALCAM* and *AFAP1* we found respectively 4 and 8 CpGs significantly associated with treatment, but these did include cg23719209 or cg12337669 which we found associated in the discovery sample; cg18803039 (*RIPOR2*) and cg08739828 (*MOCOS*) were also not successfully replicated. Regarding the treatment *vs.* outcome association intersections (131 CpGs related to 62 genes), replication was successful for several loci. Eleven out of 64 (17%) CpGs that were associated with treatment in the discovery sample were successfully replicated, namely cg06125671 (*ABTB2*) and cg22356726 (*KIF20B*) with the strongest replications (> 1.5% percentage change), followed by cg12774902 (*CHRNA10*), cg03259703 (*IGSF21*), cg16662768 (*IRF8*), cg09102332 (*ITGB8*), cg15509069 (*SDHAF3*), cg04070142 (*SLCO1A2*), cg06665305 (*SOWAHC*), cg20401896 (*WDPCP*), cg26212904 (*WSCD1*) (> 0.5% percentage change). For details, see supplement S5.

## Discussion

The present study investigated patterns of DNAm associated with trauma related mental problems and psychotherapeutic treatment in female former child soldiers from Eastern DR Congo. DNAm of different CpGs were associated with treatment but were not replicated in an independent sample. Associated genes found in discovery sample play a critical role in memory formation and fear learning/extinction and, in addition, have been associated with processes of the immune system. Moreover, 62 genes were related to both clinical/social outcomes and treatment; replication was successful in 17% of the tested CpGs. Enrichment analyses corroborated that NET might alter gene regulatory processes involved in memory formation (ECM interaction, focal adhesion, glutaminergic signalling) along with alterations of the glucocorticoid and aldosterone system amongst others. Interestingly, genes involved in GnRH signalling were associated with both social acknowledgement and treatment.

### Effect of NET treatment on DNAm

Using a longitudinal parallel group design to draw causal effects of NET *vs.* TAU on DNAm change, we found that treatment significantly predicted DNAm change from baseline to six month follow up in four CpGs (*ALCAM*, *AFAP1*, *MOCOS* and *RIPOR2).* Activated leukocyte cell adhesion molecule (*ALCAM*) and actin filament associated protein 1 (*AFAP1*) were replicated, despite the limited power of the replication sample. Products of both genes have previously been associated with the formation of memory. In NET, restructuring of the memory and particularly the establishment of hypersalient/traumatic memories in ‘time and space’ of the autobiographic memory has been postulated as cardinal agent of change for the treatment of PTSD and appetitive aggression^33,36,37^. Substantial evidence corroborates the involvement of actin filaments in neural plasticity, specifically the generation, stabilisation and consolidation of long-term memory^38^. Actin dynamics interact with several of the identified enriched gene networks, namely glutaminergic signalling, focal adhesion, ECM-receptor interaction (see review by Rudy^39^) as well as the RAP1 signalling pathway^40^. *ALCAM* is a member of the immunoglobulin superfamily and important for the growth of midbrain dopamine neurons through trans-heterophilic interactions^41,42^. Dopamine neurons in the midbrain are crucial for reinforcement learning (see review by Daw et al.^43^). Actin polymerization and midbrain dopamine neurons have both been found to modulate fear extinction in animal studies^44–47^. *ALCAM* was furthermore linked to maintaining the integrity of the blood–brain-barrier and the proliferation of T-cells^48,49^. In line with this, NET has previously been found to normalise regulatory T cells^50^, which have been reported to be reduced in individuals with PTSD^51^.

Further, among the top 0.1% significant CpGs (n = 305), loci on 62 genes were associated both with the effect of treatment and clinical/social outcomes in the discovery sample and 11 (17%) were replicated in the independent sample (percentage change of 0.5% on the same CpG in the same direction). Percentage change in the replication sample was strongest in *ABTB2* and *KIF20B.* The former encodes the ankyrin repeat and BTB/POZ domain-containing protein 2, which inhibits the aggregation of alpha-synuclein and exerts a protective effect on dopamine neurons usually killed in Parkinson disease^52^. The second encodes a kinesin-like protein relevant to cytokinesis during cell division^53,54^. Moreover, *CHRNA10* encodes neuronal nicotinic acetylcholine receptor (nAChR) complex α9 and α10, and its inhibition has recently been suggested to prevent neuropathic pain^55^. Deletion of nAChR α9 also resulted in altered bone structure in mice^56^*.* Other genes have been related to the immune system (*IGSF21, IRF8, SOWAHC*), bioenergetics (*SDHAF3*) and integrins (*ITGB8*).

Finally, epigenetic age is expected to be related to disease and mental health^57^. Increasing evidence suggests that biological, social and environmental factors induce an accelerated epigenetic aging^58^. Unfortunately, we did not find conclusive results regarding the effect of treatment on epigenetic age, since our groups had differing baseline values and there were no differences in epigenetic age between timepoints. More research is also needed in that direction.

### Gene ontology analysis

Gene ontology analysis provided further insights into biological mechanisms underlying the changes triggered by NET. Seven out of 63 genes involved in *cortisol synthesis and secretion* have been found associated with treatment in our sample, indicating changes in HPA-regulation, i.e., alterations in the elite force of defence^5^. Additionally, multiple genes associated with treatment are involved in *aldosterone syntheses and secretion*. Aldosterone is a mineralocorticoid steroid hormone that is involved in the renin–angiotensin–aldosterone-system (RAAS), crucial for the homeostatic regulation of body fluids and electrolytes. It is also implicated in cardiovascular and metabolic diseases, by mediating immune cell activation^59^. Elevated aldosterone levels were previously found in patients with depression and PTSD^60–62^. *Glutaminergic signalling* is another pathway related to treatment in our sample. Most cortical synapses in the human brain are glutamatergic (80–90%). Alteration of glutaminergic signalling is essential in the development of trauma related psychiatric disorders, which motivated the investigation into a variety of therapeutics, but with inconsistent outputs^63^. Further pathways that currently emerged in association with the treatment have traditionally been related to social behaviour. For example, *oxytocin signalling* has been described to mediate complex social behaviours in animal and human studies^64^. Moreover, *GnRH* was related with both social acknowledgement and treatment. GnRH is synthesised and released by GnRH neurons in the hypothalamus, especially during puberty and adolescence.

### DNAm markers associated with clinical and social outcomes

Our analysis revealed a mixture of overlapping epigenetic signatures associated with clinical and social outcomes (Fig. 3). This result may be explained by the fact that psychological outcomes share commonalities and are intrinsically correlated with each other, although addressing distinct mental constructs. Significant associations were found for perceived social acknowledgement as victim or survivor, current violent behaviour, PTSD severity and guilt, but not for depression or AAS. Identified genes are involved in HPA axis activation and pain modulation (*NPFFR2*)^65,66^, neural functioning (*SLC2A4* and *KCNG2*)^67,68^ immune system (*TRIM59*; ref: UniProtKB Q8IWR1), chromatic remodelling (*UBL4A*)^69^, insulin metabolism (*SLC2A4*)^70^, and transcriptional fine tuning in the early post-natal period (*DNMT3A*)^71,72^, amongst others. The multiple DMPs (i.e., on promoters) associated with social acknowledgement indicate a potential sensitivity of DNAm for processes that originate in the social environment.

### Is DNA methylation associated with mental disorders reversible?

If we assume that complex traits, such as trauma related disorders are in part influenced by epigenetic markers, one might expect that treating mental disorders reverses epigenetic changes in specific loci responsible for the development of symptoms. Our data, however, does not support this hypothesis. It is noteworthy that almost none of the CpGs associated with trauma related disorders were also associated with treatment, despite various intersections at the gene level. This indicates that treatment does not promote a simple reversal of epigenetic alterations on specific CpG sites that correlate with trauma related disorder symptoms. Instead, an adaptation of regulatory routes that are pathological or render survivors socially dysfunctional might contribute to psychological and physiological changes in response to treatment. Additionally, it is still unclear whether and to which degree methylation changes could effectively produce meaningful biological impacts in the context of psychotherapy. Research investigating biomarkers for trauma related disorders are still incipient, compared to substance use^73^, smoking^74^ or cancer^75^. Considering that our study detected small levels of DNAm change after treatment application, our findings should be interpreted with caution. Further studies should also test the hypothesis that therapy treats phenotypic conditions through alternative epigenetic routes rather than by reversing specific methylated positions on the genome.

### Limitations

Although our study has several strengths, the results need to be interpreted in light of the following limitations. Firstly, we cannot be sure that the associations found in our study directly reflect gene expression and protein levels in participants, although a growing body of research suggests that DNAm is a reliable epigenetic marker for gene regulation^76^. Secondly, the lack of significant CpG sites associated with PTSD and appetitive aggression might be due to the low variance in these particular phenotypes, since participants were preselected based on presence of a PTSD diagnosis and apparent aggressive behaviour. Thirdly, we should acknowledge that individual measures of DNAm might have been affected by aspects outside of the control of our design, such as menstrual cycle, contact with chemical substances or diet. However, systematic bias should be reduced by the randomized study design. Also, although the estimation and processing of methylation data in our study was done meticulously, we did not confirm it using a validation technique such as pyrosequencing. Further, we did not correct for multiple testing at the level of psychological outcomes, which could reduce the number of significant associations. Also, our replication method supported the association of some DMPs, but the magnitude of change was relatively small, which may mean there was not an effective change in methylation status after 6–9 months of treatment application. As indicated above, methylation change might qualitatively distinguish treatment groups, but the degree of change has still an uncertain meaning. Lastly, we cannot rule out tissue-specificity and genetic population stratification in our study. Genetic restriction should not be mistakenly understood as genetic bias however. Since we applied a paired sample design, baseline versus follow-up, individuals were their own genetic controls.

In conclusion, this study is the first to suggest that evidence based trauma therapy—specifically Narrative Exposure Therapy (NET)—might affect DNAm markers in multiple genes associated with psychiatric conditions such as PTSD, but also with trauma related social problems such as current violent behaviour and social acknowledgement. We found evidence that NET might effectively alter DNAm of loci close to genes of general and specific biological processes and pathways cardinal to trauma related problems and somatic complaints as well as social behaviour and reproduction.

## Methods

### Study procedure and participants

We applied a prospective randomised parallel group design in a sample of 88 female former child soldiers in the Eastern DR Congo. All participants fulfilled the DSM-5 diagnostic criteria for PTSD. Inclusion criteria were female sex, minimum age of 16 years and armed group involvement. Recruited through a local non-profit organisation, patients were invited to meet with psychological interviewers, where the study was outlined in detail and written informed consent was obtained. Simple randomisation was applied to eligible women for allocation to Narrative Exposure Therapy (NET) or treatment-as-usual (TAU; see Robjant et al.^32^) condition via SPSS by a person who was not involved in interviews or treatment. Four women did not offer saliva samples and were thus excluded from this analysis. The final sample included 84 women (n_net_ and n_tau_ = 42) with saliva samples collected at baseline (BL) and 6-month follow-ups (FU6). Participants had a mean age of 18 (SD = 1.9) years at baseline. All women had a history of abduction and numerous lifetime traumatic events^77^. It is very uncommon to smoke tobacco within this micro-culture, and other drugs were not available or affordable for these women, therefore this information was not gathered during interviews. All methods were performed in accordance with the relevant guidelines and regulations.

### Assessment

Structured psychodiagnostic interviews were conducted by trained local interviewers blind to treatment condition. Baseline assessment included (1) sociodemographic questions, (2) a 44-items checklist for traumatic events and perpetrated acts of violence, and (3) clinical and social outcomes. Clinical outcomes were PTSD (PSS-I; DSM-5 Posttraumatic Stress Symptom Scale Interview^78^), depression (PHQ-9; Patient Health Questionnaire^79^) and appetitive aggression (AAS; Appetitive Aggression Scale^80^). Social outcomes were current violent behaviour (CVB; checklist of Current Violent Behaviour against children, intimate partner, and others^81^), feelings of guilt (AAGS, Attitudes About Guilt Survey^82^) and perceived recognition of the trauma and support in the women’s social environment (SAQ; Social Acknowledgement Scale^83^). A more detailed description of the sample, study design, questionnaire measures and treatment is provided in Robjant et al.^32^.

**Figure 4:**
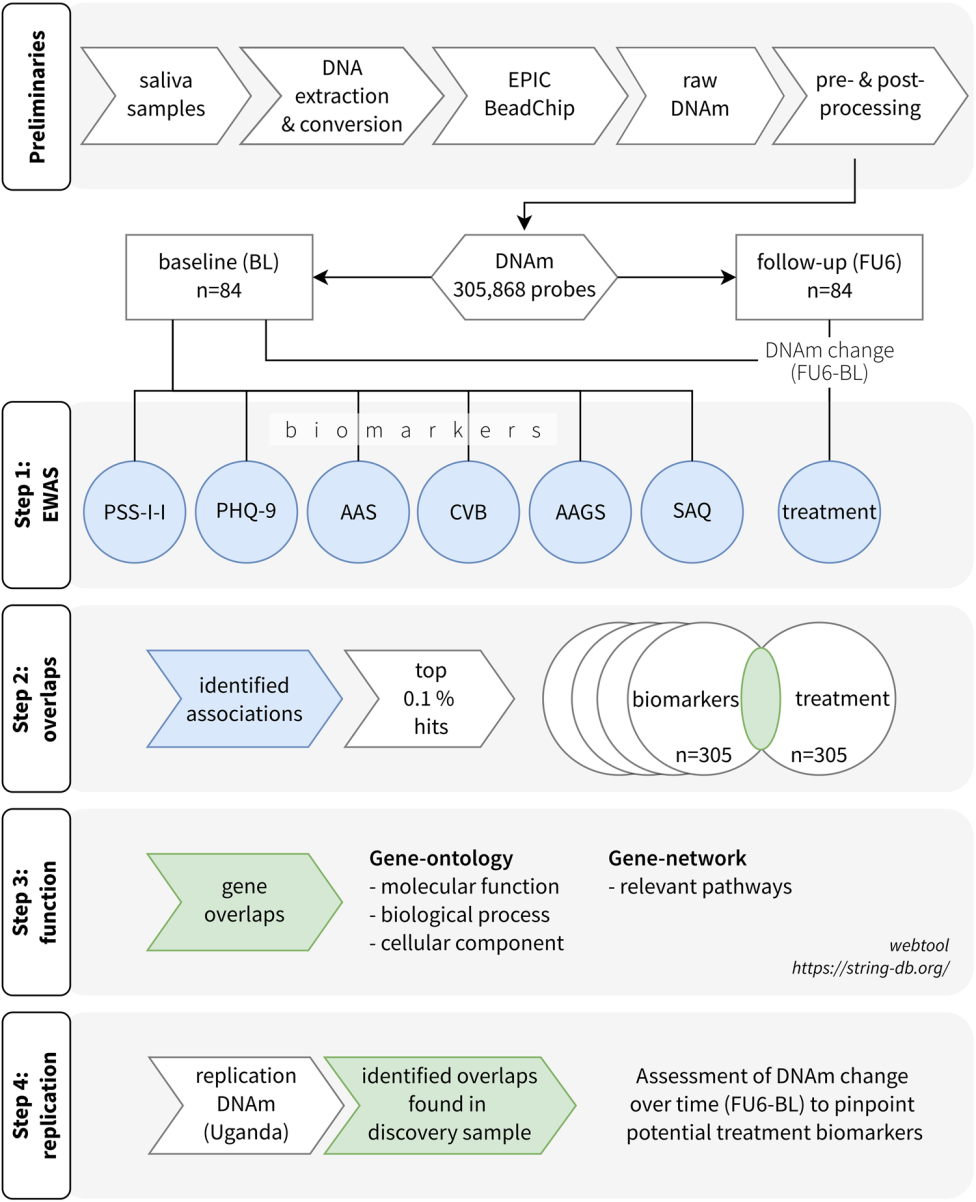
Scheme of study methodology. From top to bottom, diagram shows the four main steps of data analysis, after preliminary data collection and processing. Step 1: epigenome-wide association analysis to identify biomarkers for clinical and social outcomes considering baseline data only. Treatment association considered DNAm change accounting for baseline and follow-up data. Step 2: extraction of CpGs and genes from identified associations and test for intersections. Step 3: gene-ontology and gene-network analysis was conducted for those associations where treatment and other outcomes had overlapping results to infer relevant body functions. Step 4: replication of results in an independent sample using linear models and observing directionality and intensity of methylation change.

Saliva samples were collected at baseline and 6-month follow-up using Oragene DNA OG-500 kits (DNA Genotek, Ontario, Canada), which efficiently preserve DNA material at room temperature and above even over long periods of time^84^. Samples were stored in dry carton boxes in Goma, DR Congo, before DNA extraction and DNAm profiling (GenomeScan, Leiden, Netherlands). DNA was purified using QIAsymphony DSP DNA Midi Kit (Qiagen, Hilden, Germany) and concentrations were determined using Invitrogen Quant-iT™ PicoGreen™ dsDNA Assay Kit (Thermo Fisher Scientific, USA). Bisulfite conversion using 500 ng of genomic DNA was performed using EZ DNA Methylation Gold Kit (Zymo Research, USA), and methylation profiling was conducted using the Infinium MethylationEPIC BeadChip array (Illumina, California, USA). There were no deviations from the Illumina protocol and all experiments were performed in compliance with GenomeScan Standard Operating Procedures (SOPs). Quality control was carried out using Illumina Technical Controls Plots and using the MethylAid R package^85^. Less than 16 months elapsed between saliva collection and biochemical analysis. Raw methylation data containing 865,859 probes was processed to remove probes of bad quality (n = 12,933), non CpG or SNPs probes (n = 29,873), probes at male sex chromosomes (n = 58) and cross-reactive probes (n = 124,489). The remaining 698,540 probes were filtered to select differentially methylated positions (DMPs). In total, 305,868 CpGs were selected for statistical analyses. During processing of methylation data, we accounted for batch effects and generated estimates of epigenetic age^86^, sex (all participants were confirmed as female), and cell counts^87^ (see supplement S1) (Fig. 4).

### Data analysis

To investigate general differences in overall DNAm between treatment groups, we conducted a generalized linear mixed model using the formula *DNAm* ~ *treatment* + *time* + *treatment* × *time* + *reported age* + *nr. traumatic events* + *cell counts*, where response was mean Beta across all CpGs. Control variables were set to account for individual differences, whereas participant IDs were set as random effects. Epigenetic age was compared among groups using t-tests.

To investigate the effect of treatment on DNAm, we performed an analysis in four steps: [1] **EWAS:** an epigenome wide association study (EWAS) approach to find DNAm associations with outcomes and treatment by fitting robust generalized linear models using the formula *Y* ~ *X* + *reported age* + *nr. of lifetime traumatic events* + *cell counts*. We chose a robust model to account for potential violation of statistical assumptions. For outcomes, *Y* was the M-value (transformation of Beta values to normalize data) at baseline for each probe, and *X* was one of the outcomes. We fitted these model structures for each outcome separately to avoid multicollinearity. For treatment, *Y* was the Beta value difference between FU6 and BL, *X* was treatment condition, and *cell counts* were the mean cell count values between BL and FU6. Reported age, number of lifetime traumatic events (perpetrated and experienced) and cell counts were treated as covariates to control for individual differences and past experiences. *P*-values were adjusted for multiple testing after Benjamini-Hochberg^88^ and used to rank probes by significance (cut-off: FDR = 0.05) per outcome. QQ and MD plots were used to evaluate model fits. Statistical models were implemented in lme4^89^ and limma^90^, using R 3.5.1^91^ in RStudio^92^. [2] **Probe annotation, CpG/gene intersections:** first, metadata for probes were gathered using minfi^93^ and complemented with the updated Illumina EPIC array annotation release 03.13.2017^94^. CpGs that did not correspond to a specific gene were manually investigated using the UCSC genome browser (GRCh37/hg19 assembly), and the closest protein-coding gene was selected. All gene symbols were double-checked against the most recent HUGO (Human Genome Organization) nomenclature. Second, to find biomarkers in our sample that were both associated with clinical/social outcomes and with treatment, we intersected CpGs and genes associated with those variables. For this, we selected the top 0.1% CpGs (305 probes) ordered by adjusted *p*-value for each variable. Contrary to a conservative FDR cut-off, this allowed us to inspect a more comparable number of sets per association. [3] **Gene network analysis:** to investigate gene function and important biological pathways, we conducted a gene-network and gene-ontology analysis using STRING^95^. [4] **Replication:** statistical analyses were replicated in a subsample of female former child soldiers abducted by the Lord’s Resistance Army in Northern Uganda of whom all received NET (N = 53). These participants had a mean age of 34 (SD = 9.1) years and a history of trauma and perpetration comparable to the discovery sample. Phenotype and saliva samples were collected at baseline, 4-month (FU4) and 10-month (FU10) follow-up interviews. Phenotypic data and DNAm processing are described elsewhere^96,97^. Baseline biomarker correlates were investigated for clinical outcomes only: PTSD (PDS subscale employed as a structured interview^98^), depression (HSCL^99^) and appetitive aggression (AAS). To also control for trauma load, we used a trauma checklist specific for the Ugandan context, where information about violence exposure and perpetration was present^100^. Linear models were calculated for those CpG sites found in associations within the discovery sample by using the formula and procedure mentioned above. Replication was considered successful for the effect of treatment when the direction of DNAm change was identical with a minimum percentage change of 0.5% in both discovery and replication samples. Average percentage change in CpG methylation across all CpG sites for the entire discovery sample was 0.26% (SD = 0.89).

### Statement of ethics

All participants have given written informed consent. Concerning the discovery sample, the study protocol was approved by the Ethical Commission of the University of Konstanz and the Social Funds of the DR Congo. Concerning the replication sample, the study protocol was approved by the Ethical Commission of the University of Konstanz, the Gulu University Research and Ethics Committee (Uganda), the Lacor Hospital Institutional Research Ethics Committee (Uganda), and the Ugandan National Council of Science and Technology.

## Supplementary Information

Supplementary Information 1.

Supplementary Information 2.

Supplementary Information 3.

Supplementary Information 4.

Supplementary Information 5.

## Data Availability

The datasets generated during and/or analysed during the current study are available from the corresponding author on reasonable request.
