# Supplementary material for: DNA methylation changes following narrative exposure therapy in a randomized controlled trial with female former child soldiers
Source: Sci Rep. 2021 Sep 16;11:18493. doi: 10.1038/s41598-021-98067-9 (PMC8445994; doi:10.1038/s41598-021-98067-9)
Supplement: Supplementary file 1 — Supplementary Information 1. [file 41598_2021_98067_MOESM1_ESM.pdf]

DNA methylation changes following Narrative Exposure Therapy in a randomized controlled trial with female former child soldiers

Samuel Carleial, Daniel Nätt, Eva Unternährer, Thomas Elbert, Katy Robjant, Sarah Wilker, Vanja Vukojevic, Iris-Tatjana Kolassa, Anja C. Zeller, and Anke Koebach

---

Supplement S1

Quality control and data processing – from saliva samples to DNA methylation.

ServiceXS

Project Report

Illumina Methylation

Contact Information Customer:

Fachbereich Psychologie
Universität Konstanz
Anke Köbach
Fach 905,
78457 Konstanz
Germany

Project Information:

Reference:	AXS805209-2/ 103408-001
Project Manager:	Floor Pepers
Researcher:	Fahim Behrouz
Type of Service:	Illumina Methylation
Type of array:	Human Mehtylation 850k BeadChip

Genome scan

TESTING RvA L 518

illumina CSPro CERTIFIED SERVICE PROVIDER

This project report contains all information regarding submitted DNA samples, experimental procedures and the resulting data that was generated after completion of the experiments. If you have any additional questions regarding this report, please do not hesitate to contact your GenomeScan Project Manager.

Material:

GenomeScan received in total 192 saliva samples in 12 ml tubes on 2018-06-29. Prior to assessing the quantity of samples, genomic DNA was purified using the QIAsymphony instrument and QIAsymphony DSP DNA Midi Kit from Qiagen. Purification was succeeded for all samples, except for samples 103408-001-184, 185, 186, 187, 188, 192, which will be repeated together with samples of the next batch. After purification, concentration of DNA was determined using the PicoGreen assay from Invitrogen. Gel-electrophoresis was performed to assess the quality of the DNA samples. All samples passed our Quality control. The concentrations measured by PicoGreen can be found in appendix 1 attached to this report.

Bisulfite Conversion and QC:

Bisulfite conversion using 500 ng genomic DNA input was performed on samples 103408-001-001/002-176) using the EZ DNA Methylation Gold Kit (Zymo Research). A bisulfite QC on the samples (see **Conversion QC IC2 columns** in appendix I) was performed consisting of a qPCR reaction and melting curve analysis. Except for 4 samples, all samples passed our Quality Control.

Because of the shelf-life of bisulfite converted DNA samples, we continued with all samples directly after the bisulfite QC.

Experimental setup:

The converted samples (6 µl of each sample) were hybridized on the Illumina HumanMethylation850 BeadChip. For each locus, two types of beads are present on the array, one specific for the methylated nucleotide, and one specific for the unmethylated nucleotide. The hybridization schedule can be found in Table 1 in the appendix attached to this report.
The GenomeScan protocol that describes the method for sample preparation, hybridization and washing of the BeadChip arrays as well as the scanning procedure, was adapted from the Illumina protocol:
"Infinium II Methylation Assay Manual Protocol". The samples were scanned using the Illumina iScan array scanner. There were no deviations from the Illumina protocol and all experiments were performed in compliance with GenomeScan Standard Operating Procedures (SOPs).

The experiments were performed at the following sites: (1) GenomeScan B.V., Plesmanlaan 1d, 2333 BZ, Leiden and (2) Leids Universitair Medisch Centrum (LUMC), Albinusdreef 2 2333 ZA Leiden. The LC480 for q-PCR and purification of DNA was done at site (2). The rest of the experiments were performed at site (1).

Materials and Methods:

Please use the text below as a basis for publication purposes.
Genomic DNA was bisulfite-converted using the EZ DNA Methylation Gold Kit (Zymo Research) and used for microarray-based DNA methylation analysis, performed at GenomeScan (GenomeScan B.V., Leiden, The Netherlands) on the HumanMethylation850 BeadChip (Illumina, Inc., San Diego, CA, U.S.A). This array interrogates over 850,000 CpG sites representing about 99% of the RefSeq genes. The bisulfite-converted DNA was processed and hybridized to the HumanMethylation850 BeadChip (Illumina, Inc.), according to the manufacturer's instructions. The BeadChip images were

scanned on the iScan system and the data quality was assessed using the R script MethylAid (M. van Iterson, 2018) using default analysis settings.

Data analysis and results:
Except for one sample (103408-001-030), all samples showed a detected CpG (p0.01) above 95%, >807,500 detected CpG (p0.01). The sample failed because of a technical error in the array which was confirmed by parameters and plots showing the Illumina system controls.

A print of the Quality Control (QC) report with all the Illumina system controls present on the bead chips is included in the report. The Illumina system controls for the Infinium Methylation assay are according to expectation.

In conclusion, all QC parameters are within specifications and therefore this project was performed successfully.

Limitations:
The data was prepared for research purposes only. In case the data or part of the data is to be used in the context of medical treatment of individual patients in this study, we strongly recommend consulting a genetic counselor. In some cases, the counselor may order a confirmatory test using an independent sample. Consulting a genetic counselor is also recommended if study participants request to obtain a copy of the methylation data.

ServiceXS

Quality Control Report

Illumina Infinium HD Methylation

MethylAid Tool for Data and Sample Quality Control

**Project number:**	**103408**
**Commisioned by:**	**Universitat Konstanz**
**BeadChip type:**	**Methylation850k(Epic)**

Introduction

Quality control (QC) of data is an important step when performing any microarray study. After all data has been collected, a combination of the metrics and techniques for ascertaining data quality can help to assess the overall data quality and to identify possible outlier samples. The potential cause of outlier data determines whether a sample needs to be repeated or removed from the dataset.

GenomeScan uses the R script **MethylAid** [M. van Iterson, Aug 2014] to assess the data quality (**data QC**) using the Illumina Technical Controls Plots. The plots are not included in this report. The controls can be viewed by opening the included interactive visualization tool.

The five Filter Plots can be used to define possible sample outliers (**sample QC**), these are represented in the next pages of this report.

Together with this report, an R and a shiny-methyl environment are delivered to you enabling assessing the data QC and sample QC plots interactively.

Data QC

The categories for the Illumina Controls are divided into 'sample-independent' and 'sample-dependent' controls. Below table describes the system controls used for this project and if the QC has passed or failed.

**Control metric**	**Type of control**	**Result**
Staining	Sample-independent	QC Passed
Hybridization	Sample-independent	QC Passed
Target Removal	Sample-independent	QC Passed
Extension	Sample-independent	QC Passed
Bisulfite Conversion I	Sample-dependent	QC Passed
Bisulfite Conversion II	Sample-dependent	QC Passed
Specificity I	Sample-dependent	QC Passed
Specificity II	Sample-dependent	QC Passed
Negative	Sample-dependent	not included
Non-polymorphic	Sample-dependent	QC Passed

Sample QC
Besides the Illumina system controls, five filter control plots are created to determine bad-quality samples. The sample QC analysis helps to identify samples for which the data characteristics are significantly different than the majority.

To make optimal use of the plotting area and filtering out bad quality samples easier by using just one threshold all plots except the one that is based on the detection p-value are 45 degrees rotated like the traditional microarray MA-plots. The default thresholds of this tool are validated by ServiceXS data.

There are five different filter plots:

MU plot based on the median Methylated and Unmethylated log2 intensity
Overall sample-dependent control plot based on non-polymorphic sample-dependent quality control probes
Bisulfite conversion control plot based on Type I bisufite conversion quality control probes
Overall sample-dependent control plot based on sample-dependent hybridization quality control probes
Detection p-value plot representing per sample the fraction

Please notice that the background data is obtained from the HumanMethylation 450 Bead Chip. The threshold in the filter plots are not based on the Methylation Epic data.

Rotated M vs U plot

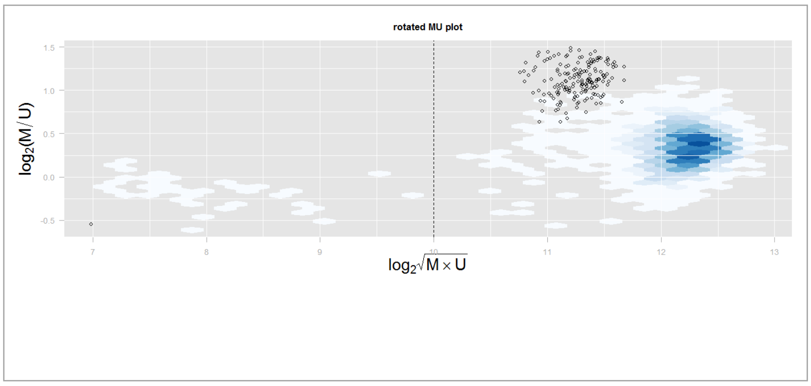

Threshold: the median Methylated and Unmethylated log2 intensity > 10

Sample-dependent overall quality plot

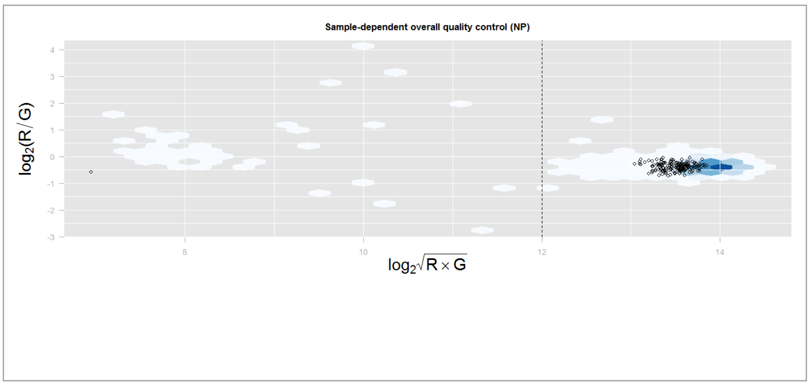

Threshold: an average log2 intensity of the expected signals in green and red channel of non-polymorphic controls > 12

Bisulfite conversion I plot

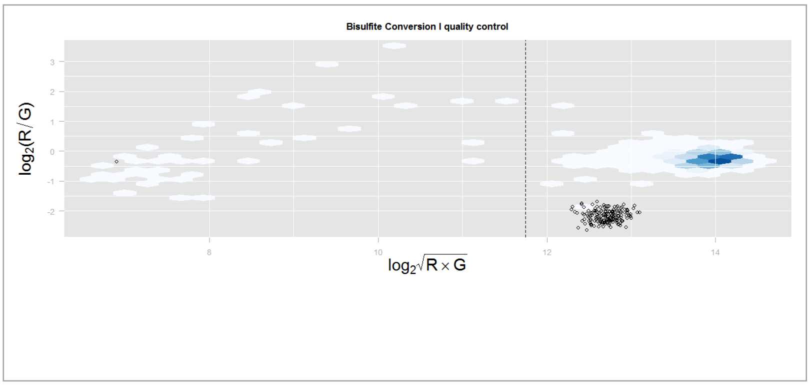

Threshold: an average log2 intensity of converted Bisulfite Type I controls in green and red channel > 11.75
For samples that are processed with the TrueMethyl Kit, the log2 intensity is generally slightly lower.

Sample-independent overall quality plot

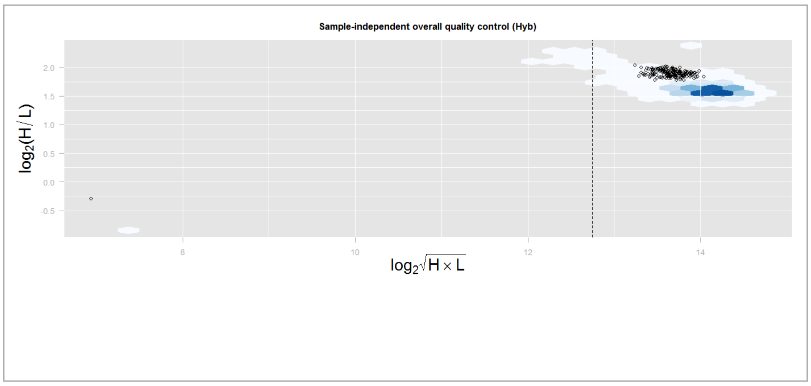

Threshold: an average log2 intensity of High and Low hybridization controls (green channel) > 12.75

Detection p-value plot

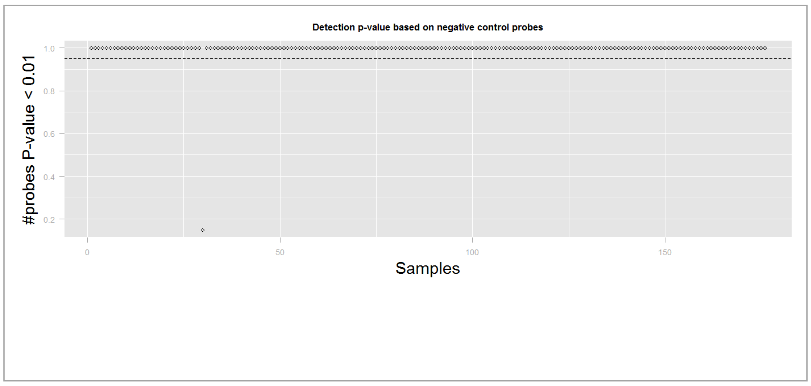

Threshold: > 95% of detected CpG's (p=0.01) above the background signal

DNA methylation data pre-processing

Data pre-processing was done in R 3.5.1 (R Core Team, 2018) using the minfi R package (Aryee et al., 2014) within the computing cluster servers of the University of Konstanz (Baden-Württemberg State, Germany). First, we removed two samples that did not pass quality control for bisulphite conversion according to the genome centre. These samples belonged to the same control treated patient (baseline and follow up). To control for sample and probe quality, we filtered observations that had at least 90% of acceptable detection p-values (cut-off: 1%). In this step, all samples passed quality control, but 12,933 probes were removed. To normalize unwanted variation in Beta values, we regressed out the variability explained by control probes present on the array using the *preprocessFunnorm* function. Then, we filtered out non-CpG methylations and SNPs (29,873) annotated in the IlluminaHumanMethylation-EPICanno.ilm10b4.hg19 package (Hansen, 2017) with the functions *dropMethylationLoci* and *dropLociWithSnps*, respectively. We removed cross-reactive probes (124,489) after Pidsley et al. (2016), and probes at the male chromosome (58). At this stage, we had an array data of 170 samples each with 698,540 probes.

Quality control and biological variation assessment

To inspect the quality of the methylation data, we used standard visualization plots implemented in minfi across the pre-processing stage. To verify that samples matched to the same individuals between timepoints, we used a simple genotyping algorithm to assess the Beta value quartiles over the SNP probes. We categorized Beta values into a lower (<0.25) and upper extreme (>.75) or intermediary. This allowed us to infer a mendelian heterozygous pattern, which if nonmatching would suggest sample mix-up. We found four samples from the control treatment that did not correspond with their counterparts (two baseline/follow up pairs). By swapping the follow up samples of these mix-ups, we found a complete match. To avoid sample size loss, we carried out the further analyses including these two individuals (four samples) with their samples adequately matched. We found another patient sample that did not match between baseline and follow up and she was excluded from analysis. Further, we confirmed that all participants were women by using the *getSex*. In addition, we estimated the epigenetic age (Horvath, 2013) of participants using the *agep* function in the 10atermelon R package (R Pidsley, Wong, Volta, Lunnon, & Mill, 2013). Epigenetic and reported ages were positively correlated for baseline (26.9%; $t_{[df = 83]} = 2.54$, p-value = .013) and follow up (28.7%; $t_{[df = 83]} = 2.73$, p-value = .008). To account for cell type heterogeneity in our samples extracted from saliva, we adapted a formula from Eipel et al. (2016) that allowed us to estimate epithelial cell percentages. Cell percentage estimations were calculated as a single numerical value for each individual sample. Additionally, we estimated cell counts (B, Buccal, CD4T, CD8T, Gran, Mono, NK cells) with the function *meffil.estimate.cell. counts.from.betas* in the meffil package (Suderman, Hemani, & Min, 2020).

DNA methylation data post-processing

Pre-processed array data (168 samples each with 698,540 probes) was then handled to adjust for batch-effects using plate identities (Illumina sentrix ID). For this, we used the *ComBat* function in the sva R package (Leek et al., 2019), which adjusts for batch-effects using an empirical Bayesian framework (adapted from Johnson, Li, & Rabinovic, 2007). For this, we fed the function with normally distributed M-values obtained from transformation of the beta DNAm values. In those cases where Beta to M-value transformation were inappropriate (Beta = 0 or 1 was found 100 times in our sample), we truncated the data to the lower/upper extremes

of M-values to avoid probe loss. After batch-effect correction, we back-transformed the new M-values into Betas. Batch-effect correction was controlled by comparing the Principal Components (PCA) of Beta values before and after running the *ComBat* function. To further reduce the size of our data, we finally selected only the differentially methylated CpG probes showing at least 5% DNAm variation (after Edgar, Jones, Robinson, & Kobor, 2017; Lemire et al., 2015). This procedure allows to ease statistical analysis, by removing bias from spurious and outlier effects. The final dataset contained 168 samples with 305,868 probes each for 84 participants evenly distributed in the control and treatment groups.

Mean DNA methylation in sample subsets

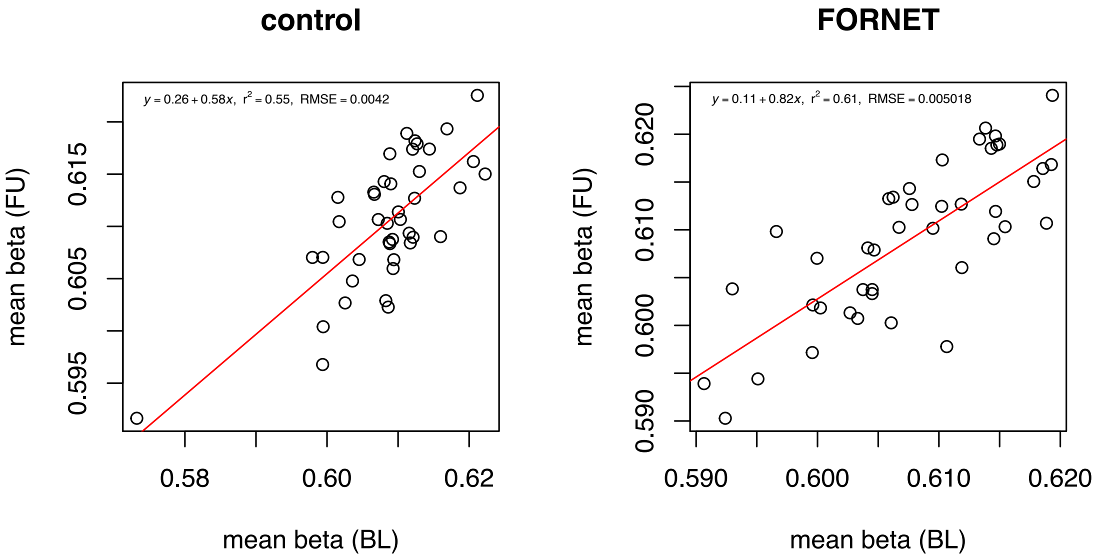

Figure S1.1. Scatterplots further showing that for both control (left) and FORNET (right) there is a positive correlation between beta values measured ad baseline and follow-up. Note that methylation values indicate and overall hypermethylation in the epigenome of study participants.

Reported age vs. Epigenetic age

Figures S1.2-3 show the relationship between reported age (assessed at baseline) and the (predicted) epigenetic ages estimated from collected DNAm samples at baseline and follow-up in the control and FORNET treatment. Note that TAU group (at follow-up) there was one unpaired extra sample.

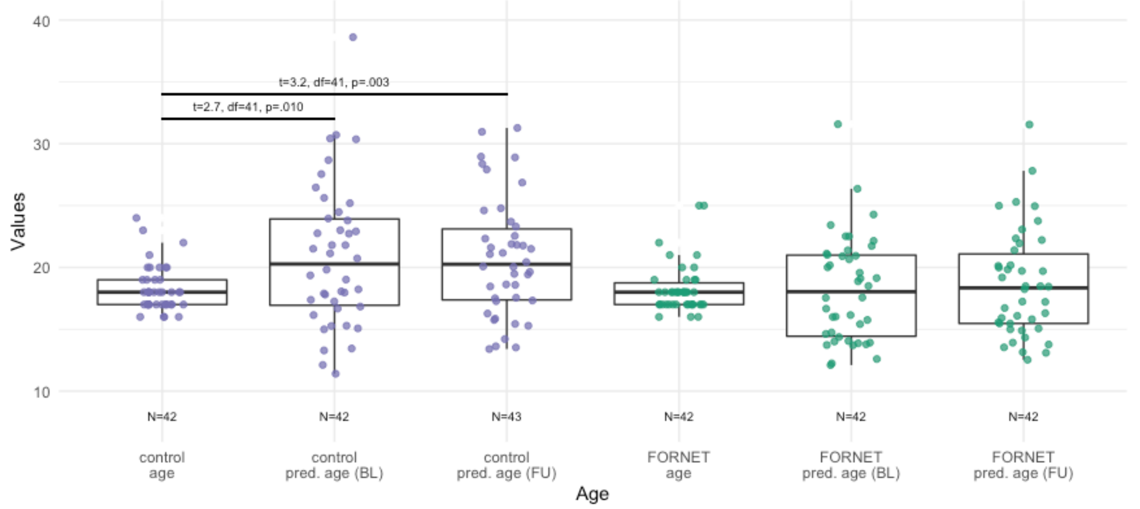

Figure S1.2. Comparison of reported vs. predicted epigenetic age in control (TAU; purple) and treatment (FORNET; green) groups. Note: t-tests show that participants had larger "epigenetic" age compared to reported age at the control, but not at the treatment group. Total sample size analyzed in the EWAS was N=84, but for control at follow-up we also have an additional unpaired sample.

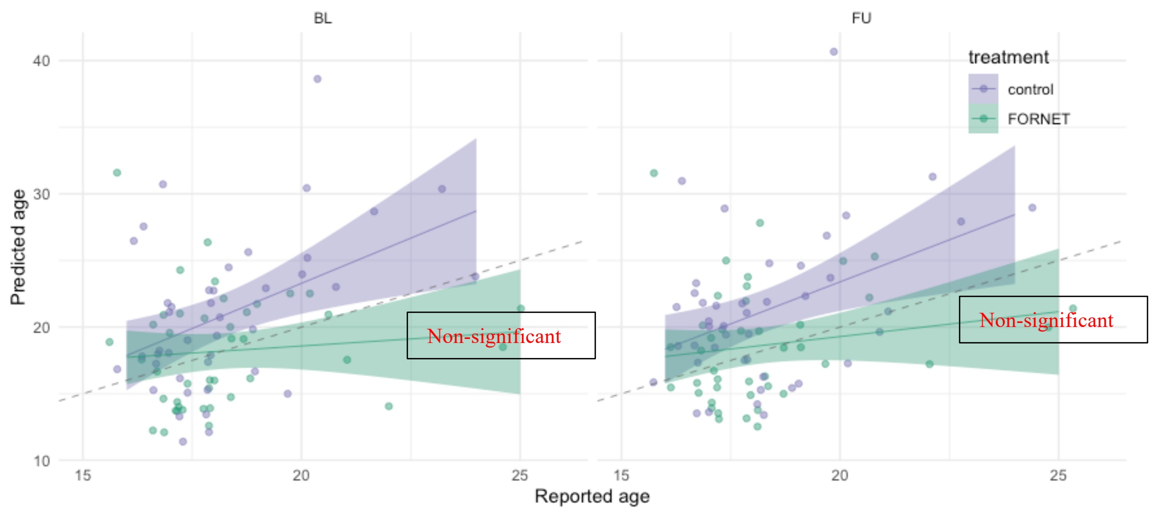

Figure S1.3. Scatterplot of reported vs. predicted epigenetic age for baseline (BL) and follow-up (FU) for control (TAU; purple) and treatment group (FORNET; green). Diagonal dotted lines in grey represent a perfect positive correlation. Note that the predicted epigenetic age is larger than reported age for the control (purple), whereas for the FORNET (green) it is the opposite. Fitted interval refer to separate linear models; for FORNET (green), we did not find significant effects.

Correlations (shown above)

Regarding the regression lines shown above in A1.4, correlation tests are as follows:
Reported vs. Predicted age (treatment control at baseline): 0.43 (t=3.0, df=40, p=.004)
Reported vs. Predicted age (treatment control at follow-up): 0.44 (t=3.1, df=40, p=.004)
Reported vs. Predicted age (treatment FORNET at baseline): 0.10 (t=0.6, df=40, p=.500)
Reported vs. Predicted age (treatment FORNET at follow-up): 0.17 (t=1.1, df=40, p=.300)

QQ-plots from p-value in linear models

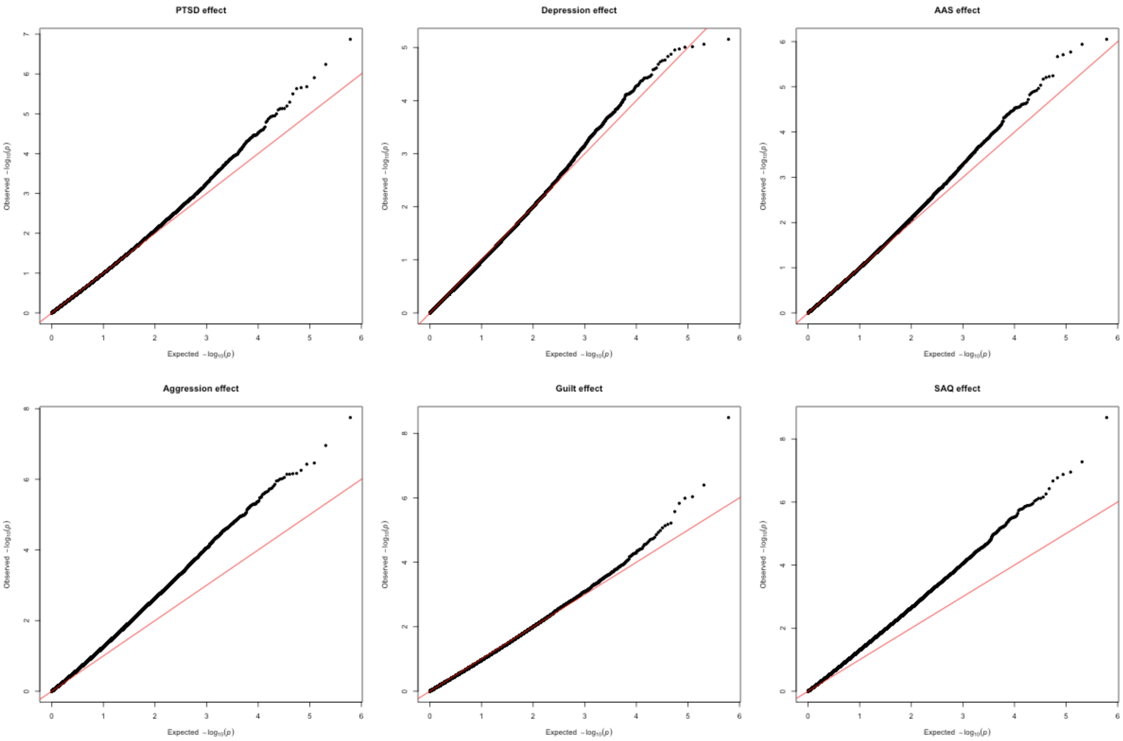

Figure S1.4. Quantile-quantile plots from p-values in the EWAS study testing the effect of the six clinical and social traits on DNAm at baseline of Kibumba women in DRC.

CpG positions of biomarkers found in study

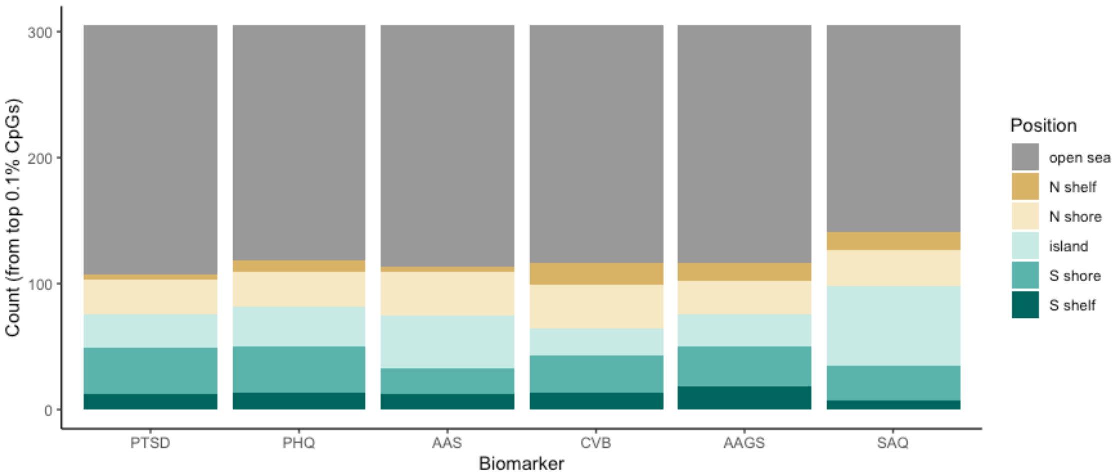

Figure S1.5. Barplot showing the position where associated CpGs are present in the genome. Positions of CpGs were taken from the Illumina annotation file designed for the Illumina EPIC 850k technique. Number of CpGs refer to the top 0.1% (n=305) cut-off used in the analysis.

Cell count estimations

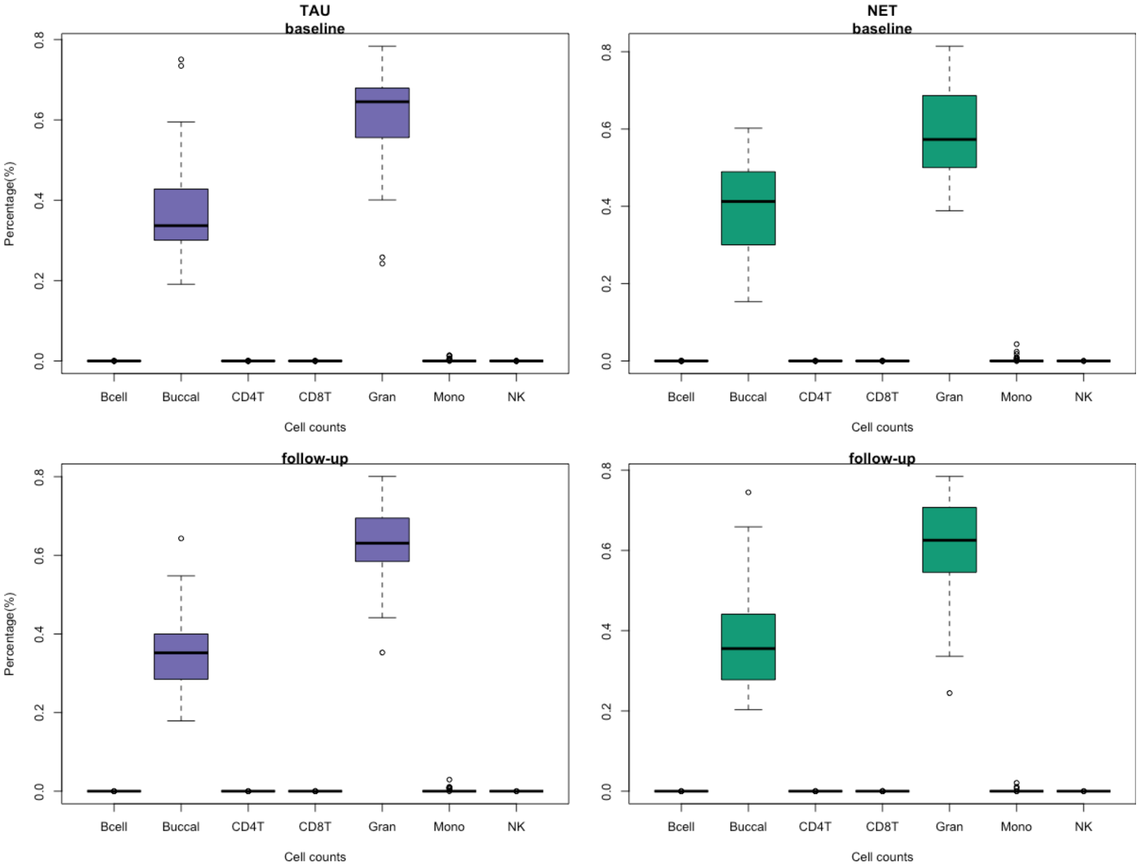

Figure S1.6. Boxplots showing the estimated cell counts per sample subset (calculated with the R package meffil). Treatment groups are labeled with colors.

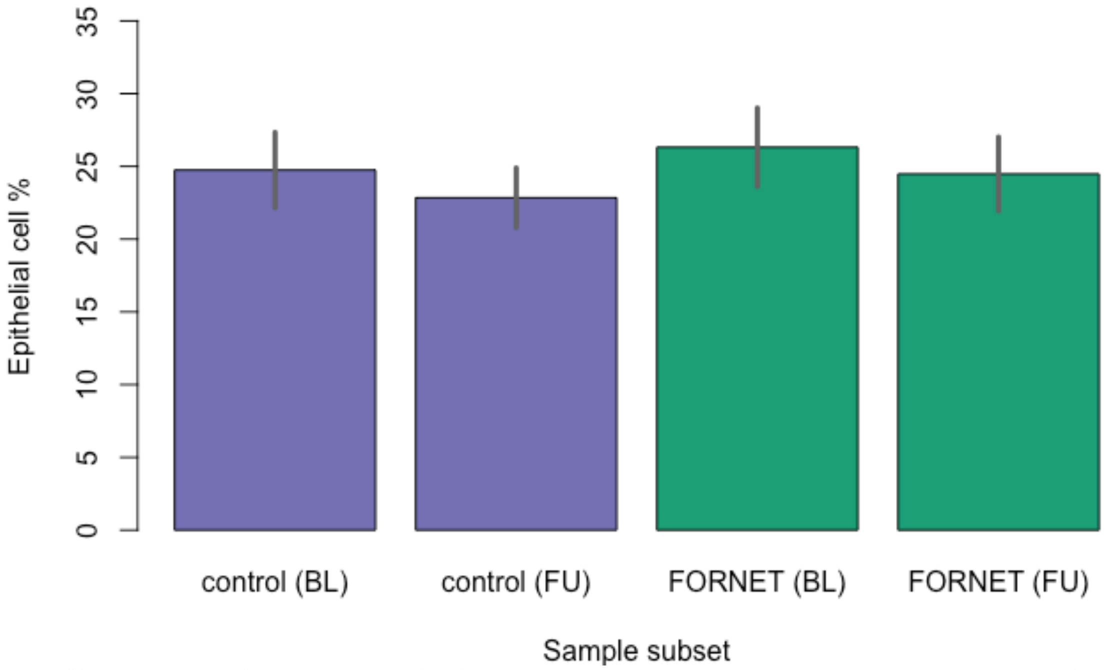

Figure S1.7. Bar plots showing the estimated epithelial cell percentages per sample subset. Treatment groups are labeled with colors. Bars represent approximate confidence intervals based on standard errors.

References

Aryee, M. J., Jaffe, A. E., Corrada-Bravo, H., Ladd-Acosta, C., Feinberg, A. P., Hansen, K. D., & Irizarry, R. A. (2014). minfi: a flexible and comprehensive Bioconductor package for the analysis of Infinium DNA methylation microarrays. *Bioinformatics, 30*, 1363 - 1369. doi:https://doi.org/10.1093/bioinformatics/btu049

Edgar, R. D., Jones, M. J., Robinson, W. P., & Kobor, M. S. (2017). An empirically driven data reduction method on the human 450K methylation array to remove tissue specific non-variable CpGs. *Clinical epigenetics, 9*(1), 11.

Eipel, M., Mayer, F., Arent, T., Ferreira, M. R., Birkhofer, C., Gerstenmaier, U., . . . Wagner, W. (2016). Epigenetic age predictions based on buccal swabs are more precise in combination with cell type-specific DNA methylation signatures. *Aging (Albany NY), 8*(5), 1034-1044. doi:10.18632/aging.100972

Hansen, K. D. (2017). IlluminaHumanMethylationEPICanno.ilm10b4.hg19: Annotation for Illumina's EPIC methylation arrays. R package version 0.6.0. https://bitbucket.com/kasperdanielhansen/Illumina_EPIC.

Horvath, S. (2013). DNA methylation age of human tissues and cell types. *Genome biology, 14*(10), 3156.

Johnson, W. E., Li, C., & Rabinovic, A. (2007). Adjusting batch effects in microarray expression data using empirical Bayes methods. *Biostatistics, 8*(1), 118-127.

Leek, J. T., Johnson, W. E., Parker, H. S., Fertig, E. J., Jaffe, A. E., Storey, J. D., . . . Torres, L. C. (2019). sva: surrogate variable analysis. R package version 3.30.1.

Lemire, M., Zaidi, S. H., Ban, M., Ge, B., Aïssi, D., Germain, M., . . . Gagnon, F. (2015). Long-range epigenetic regulation is conferred by genetic variation located at thousands of independent loci. *Nature communications, 6*, 6326.

Pidsley, R., Wong, C., Volta, M., Lunnon, K., & Mill, J. (2013). A data-driven approach to preprocessing Illumina 450K methylation array data. *BMC Genomics, 14*(1), 293.

Pidsley, R., Zotenko, E., Peters, T. J., Lawrence, M. G., Risbridger, G. P., Molloy, P., . . . Clark, S. J. (2016). Critical evaluation of the Illumina MethylationEPIC BeadChip microarray for whole-genome DNA methylation profiling. *Genome biology, 17*(1), 208.

R Core Team. (2018). R: a language and environment for statistical computing. Vienna, Austria: R Foundation for Statistical Computing. Retrieved from https://www.R-project.org

Suderman, M., Hemani, G., & Min, J. (2020). meffil: efficient algorithms for DNA methylation. R package version 1.1.1. https://github.com/perishky/meffil.
