# Supplementary material for: DNA methylation changes following narrative exposure therapy in a randomized controlled trial with female former child soldiers
Source: Sci Rep. 2021 Sep 16;11:18493. doi: 10.1038/s41598-021-98067-9 (PMC8445994; doi:10.1038/s41598-021-98067-9)
Supplement: Supplementary file 2 — Supplementary Information 2. [file 41598_2021_98067_MOESM2_ESM.pdf]

DNA methylation changes following Narrative Exposure Therapy in a randomized controlled trial with female former child soldiers

Samuel Carleial, Daniel Nätt, Eva Unternährer, Thomas Elbert, Katy Robjant, Sarah Wilker, Vanja Vukojevic, Iris-Tatjana Kolassa, Anja C. Zeller, and Anke Koebach

---

Supplement S2

Multiple linear model results (EWAS). Output tables showing the top 0.1% CpG probes (n = 305) and their respective estimates and annotation found to be significantly associated with DNAm through the effects of PSS-I, PHQ-9, AAS, CVB, AAGS, SAQ and treatment.

TREATMENT

rank	cpg	cpg.position	region	annotation	gene	gene.group	gene.feature	AveExpr	logFC	CI.L	CI.R	t	P.Value	adj.P.Val	fdr	sign
1	cg23719209	chr3:105333123	open sea		ALCAM			-0.005	0.031	0.020	0.041	5.632	2.50E-07	0.044207218	**	+
2	cg18803039	chr6:25119579	open sea	CMAHP	RIPOR2	Body		0.005	-0.035	-0.048	-0.023	-5.597	2.89E-07	0.044207218	**	-
3	cg12337669	chr4:7881857	open sea	AFAP1	AFAP1	5'UTR		0.001	0.025	0.016	0.034	5.440	5.51E-07	0.04884945 1	**	+
4	cg08739828	chr18:33768144	island	MOCOS	MOCOS	Body	promoter-linked	0.004	0.035	0.022	0.048	5.405	6.39E-07	0.04884945 1	**	+
5	cg10034690	chr17:79469842	S shelf		ACTG1			0.006	0.029	0.018	0.041	5.076	2.41E-06	0.126146394	ns	+
6	cg26066917	chr11:117072446	open sea	TAGLN	TAGLN	Body		0.005	0.023	0.014	0.032	5.070	2.47E-06	0.126146394	ns	+
7	cg19146902	chr11:47516584	open sea	CELF1	CELF1	TSS1500		0.005	-0.043	-0.060	-0.025	-4.909	4.69E-06	0.19646779	ns	-
8	cg24376689	chr6:32016336	open sea	TNXB	TNXB	Body		-0.003	-0.032	-0.045	-0.019	-4.885	5.14E-06	0.19646779	ns	-
9	cg19528502	chrX:100663176	island	HNRNPH2	HNRNPH2	TSS200	promoter-linked	-0.005	0.027	0.016	0.038	4.776	7.85E-06	0.245678939	ns	+
10	cg16202803	chr2:240014758	open sea	HDAC4	HDAC4	Body		0.002	-0.018	-0.026	-0.011	-4.770	8.03E-06	0.245678939	ns	-
11	cg20105042	chr16:71470299	open sea		TLE7		unknown cellspecific	0.008	0.028	0.016	0.040	4.634	1.35E-05	0.376366153	ns	+
12	cg06899168	chrX:122583724	open sea	GRIA3	GRIA3	Body		-0.006	-0.038	-0.055	-0.021	-4.559	1.80E-05	0.459981917	ns	-
13	cg21361971	chrX:114546624	island		LUZP4			0.001	-0.013	-0.019	-0.008	-4.527	2.03E-05	0.477460834	ns	-
14	cg03139519	chr11:46388733	S shore	DGKZ	DGKZ	Body		0.003	-0.020	-0.029	-0.011	-4.485	2.38E-05	0.500917256	ns	-
15	cg06052122	chr9:123546098	open sea	FBXW2	FBXW2	Body		-0.014	-0.048	-0.069	-0.027	-4.477	2.46E-05	0.500917256	ns	-
16	cg03295070	chr18:67966894	open sea	SOCS6	SOCS6	5'UTR		0.007	0.019	0.011	0.027	4.452	2.69E-05	0.503320554	ns	+
17	cg08219991	chr14:100054932	open sea	CCDC85C	CCDC85C	Body	unknown cellspecific	-0.004	-0.024	-0.034	-0.013	-4.442	2.80E-05	0.503320554	ns	-
18	cg11366422	chr12:2950388	open sea	ITFG2	ITFG2	Body		-0.001	-0.022	-0.032	-0.012	-4.409	3.17E-05	0.537920033	ns	-
19	cg23342785	chr12:12200307	open sea		ETV6			0.006	0.024	0.013	0.034	4.392	3.36E-05	0.541629347	ns	+
20	cg03188000	chr12:68634913	open sea		IL22			0.021	0.050	0.027	0.072	4.367	3.70E-05	0.564499059	ns	+
21	cg09102332	chr7:20498337	open sea		ITGB8			-0.008	-0.042	-0.062	-0.023	-4.338	4.12E-05	0.564499059	ns	-
22	cg10683527	chr4:178230606	N shore	NEIL3	NEIL3	TSS1500	promoter-linked	0.005	-0.023	-0.034	-0.013	-4.329	4.25E-05	0.564499059	ns	-
23	cg19895297	chr3:160938722	N shore	NMD3	NMD3	TSS1500	promoter-linked cellspecific	0.000	-0.017	-0.025	-0.009	-4.316	4.46E-05	0.564499059	ns	-
24	cg27230044	chr7:94285993	island	SGCE	SGCE	TSS1500	promoter-linked	-0.006	0.038	0.020	0.055	4.309	4.59E-05	0.564499059	ns	+
25	cg08123045	chr1:156467188	N shelf	MEF2D	MEF2D	5'UTR	promoter-linked cellspecific	-0.004	-0.039	-0.056	-0.021	-4.306	4.63E-05	0.564499059	ns	-
26	cg07518851	chr10:135038446	island	KNDC1	KNDC1	3'UTR		0.009	0.022	0.012	0.032	4.296	4.80E-05	0.564499059	ns	+
27	cg00254793	chr9:112541442	N shore	PALM2	PALM2	TSS1500		0.002	0.030	0.016	0.043	4.270	5.29E-05	0.582845642	ns	+
28	cg21993406	chr5:68484783	N shore	CENPH	CENPH	TSS1500	promoter-linked	-0.005	0.027	0.014	0.040	4.267	5.35E-05	0.582845642	ns	+
29	cg16662768	chr16:86135313	open sea		IRF8			-0.010	-0.031	-0.046	-0.017	-4.258	5.53E-05	0.582845642	ns	-
30	cg03926951	chr20:35065797	S shore	DLGAP4	DLGAP4	Body		-0.007	0.022	0.012	0.032	4.241	5.87E-05	0.585339938	ns	+
31	cg20216752	chr17:6913521	N shelf	ALOX12	ALOX12	Body		0.000	-0.023	-0.034	-0.012	-4.221	6.31E-05	0.585339938	ns	-
32	cg10044179	chr21:15352983	S shore	ANKRD20A11P	LIPI	TSS1500		-0.006	0.030	0.016	0.044	4.212	6.52E-05	0.585339938	ns	+
33	cg13933773	chrX:103217102	N shore	TMSB15B	TMSB15B	Body	unknown cellspecific	0.004	0.034	0.018	0.050	4.207	6.64E-05	0.585339938	ns	+
34	cg21537297	chr8:144298583	open sea	GPIHBP1	GPIHBP1	3'UTR	unknown cellspecific	-0.001	-0.029	-0.042	-0.015	-4.202	6.76E-05	0.585339938	ns	-
35	cg23996800	chr11:76507176	N shelf	TSKU	TSKU	Body	unknown cellspecific	-0.001	0.024	0.013	0.035	4.191	7.05E-05	0.585339938	ns	+
36	cg16575427	chr17:2603795	S shore	CLUH	CLUH	Body		0.005	0.026	0.014	0.038	4.187	7.13E-05	0.585339938	ns	+
37	cg03409880	chr2:241227962	open sea	SNORD3P1	GPC1			-0.006	-0.019	-0.029	-0.010	-4.186	7.17E-05	0.585339938	ns	-
38	cg22351666	chr1:1365730	S shelf	LINC01770	TMEM88B	Body		0.002	0.024	0.012	0.035	4.182	7.27E-05	0.585339938	ns	+
39	cg16939412	chr1:93131829	open sea	EVI5	EVI5	Body		-0.003	-0.032	-0.048	-0.017	-4.167	7.70E-05	0.588582139	ns	-
40	cg04417860	chr6:28505377	open sea		GPX5			0.003	0.033	0.017	0.049	4.167	7.70E-05	0.588582139	ns	+
41	cg15079605	chr14:73704078	island	PAPLN	PAPLN	TSS200	unknown cellspecific	0.006	0.020	0.010	0.029	4.156	7.99E-05	0.596236842	ns	+
42	cg17311726	chr5:537694	S shore		SLC9A3			0.002	0.024	0.013	0.036	4.147	8.24E-05	0.60020632	ns	+
43	cg24719112	chr4:28790914	open sea		MIR4275			0.005	-0.054	-0.081	-0.028	-4.127	8.88E-05	0.616099513	ns	-
44	cg03259703	chr1:18432992	N shore	IGSF21	IGSF21	TSS1500		-0.003	0.016	0.008	0.024	4.120	9.09E-05	0.616099513	ns	+
45	cg19047660	chr17:46619923	N shore	HOXB2	HOXB2			-0.007	-0.021	-0.031	-0.011	-4.119	9.14E-05	0.616099513	ns	-
46	cg02261696	chr4:186933456	open sea		SORBS2			-0.003	-0.017	-0.026	-0.009	-4.110	9.41E-05	0.616099513	ns	-
47	cg00199091	chr6:42228954	S shore	TRERF1	TRERF1	Body	gene-linked	0.008	0.041	0.021	0.060	4.109	9.47E-05	0.616099513	ns	+
48	cg15354892	chr7:72935613	island	BAZ1B	BAZ1B	Body		0.004	0.026	0.013	0.039	4.090	0.000101103	0.627595565	ns	+
49	cg18446895	chr1:44398886	N shore	ARTN	ARTN	TSS200	unknown cellspecific	-0.003	0.024	0.012	0.036	4.083	0.000103599	0.627595565	ns	+
50	cg05339414	chr12:125423730	open sea		DHX37		promoter-linked	-0.008	-0.029	-0.043	-0.015	-4.061	0.000112152	0.627595565	ns	-
51	cg12449295	chr20:43387352	open sea	RIMS4	RIMS4	Body		-0.009	-0.022	-0.032	-0.011	-4.059	0.000112934	0.627595565	ns	-
52	cg10996267	chr11:73965049	open sea	PPME1	PPME1	3'UTR		0.008	0.017	0.009	0.026	4.059	0.000113212	0.627595565	ns	+
53	cg12087836	chr10:54644098	open sea		MBL2			0.006	0.028	0.014	0.041	4.054	0.000114884	0.627595565	ns	+
54	cg07223751	chr12:128603402	open sea	LINC02368	TMEM132C	Body		0.004	0.037	0.019	0.055	4.053	0.000115558	0.627595565	ns	+
55	cg12637676	chr8:11587161	open sea	GATA4	GATA4	Body		-0.005	-0.022	-0.033	-0.011	-4.048	0.000117377	0.627595565	ns	-
56	cg01149400	chr12:56665134	open sea	RP11-977G19.14	CS		gene-linked cellspecific	-0.001	-0.025	-0.037	-0.013	-4.048	0.000117444	0.627595565	ns	-
57	cg17130486	chr12:52437360	open sea	NR4A1	NR4A1			0.006	0.023	0.012	0.034	4.046	0.000118394	0.627595565	ns	+
58	cg04127455	chr1:156572731	open sea	GPATCH4	GPATCH4	TSS1500		0.006	0.027	0.014	0.040	4.044	0.000119007	0.627595565	ns	+
59	cg16967191	chr5:175281742	open sea	CPLX2	CPLX2	5'UTR		-0.008	0.042	0.021	0.062	4.035	0.000123064	0.637987885	ns	+
60	cg01001792	chr17:28707461	S shore	CPD	CPD	TSS200		0.017	0.028	0.014	0.042	4.019	0.000130254	0.647981949	ns	+
61	cg12082522	chr15:90380255	open sea	AP3S2	AP3S2	Body		-0.006	-0.021	-0.032	-0.011	-4.011	0.000134057	0.647981949	ns	-
62	cg14794991	chr10:87971936	open sea	GRID1	GRID1	Body		-0.003	0.026	0.013	0.039	4.008	0.000135355	0.647981949	ns	+
63	cg17291136	chr8:26087851	open sea	PPP2R2A	PPP2R2A			-0.001	-0.030	-0.045	-0.015	-4.002	0.000138155	0.647981949	ns	-
64	cg15779716	chr3:45188208	S shore	CDCP1	CDCP1	TSS1500		-0.004	-0.019	-0.028	-0.010	-4.002	0.000138426	0.647981949	ns	-
65	cg12147998	chrX:92928934	S shore	FAM133A	FAM133A	TSS200	unknown cellspecific	-0.008	0.022	0.011	0.032	4.001	0.000138652	0.647981949	ns	+
66	cg14426672	chr6:32093854	N shelf	ATF6B	ATF6B	Body		0.001	-0.023	-0.034	-0.012	-3.999	0.000139821	0.647981949	ns	-
67	cg03454225	chr6:168225472	N shore	AFDN	AFDN	Body		0.002	0.019	0.010	0.029	3.987	0.000145908	0.651249682	ns	+
68	cg19797049	chr7:102985504	S shore	DNAJC2	DNAJC2	TSS200	promoter-linked	0.007	0.044	0.022	0.065	3.979	0.000150272	0.651249682	ns	+
69	cg17431779	chr12:17368683	open sea		RPL7P40			-0.016	-0.034	-0.052	-0.017	-3.972	0.000153693	0.651249682	ns	-
70	cg27546241	chr19:7701753	island	STXBP2	STXBP2	TSS1500	promoter-linked	-0.002	-0.019	-0.028	-0.009	-3.970	0.00015479	0.651249682	ns	-
71	cg08008065	chr1:62673941	open sea	L1TD1	L1TD1	Body		-0.001	-0.034	-0.050	-0.017	-3.970	0.000155176	0.651249682	ns	-
72	cg10699312	chr3:10203470	N shelf		IRAK2			-0.014	-0.033	-0.049	-0.016	-3.968	0.000156249	0.651249682	ns	-
73	cg27434326	chr2:45395252	N shore	AC009236.2	SIX2			0.000	0.020	0.010	0.031	3.963	0.00015848	0.651249682	ns	+
74	cg23898189	chr14:80682691	open sea	DIO2	DIO2			-0.001	0.017	0.008	0.025	3.961	0.00015993	0.651249682	ns	+
75	cg25214158	chr10:101289600	N shore	LINC01475	NKX2-3			-0.002	0.026	0.013	0.039	3.960	0.000160441	0.651249682	ns	+
76	cg17717357	chr1:68699954	S shelf	WLS	WLS			0.003	-0.040	-0.060	-0.020	-3.958	0.000161818	0.651249682	ns	-
77	cg11705746	chr11:2398061	N shore	CD81	CD81	TSS1500		-0.004	0.019	0.009	0.028	3.946	0.000168575	0.657116697	ns	+
78	cg25955818	chr16:71916669	N shore	ZNF821	ZNF821	5'UTR	promoter-linked	-0.005	-0.027	-0.041	-0.014	-3.940	0.000171662	0.657116697	ns	-

79	cg01256539	chr5:119802022	S shore	PRR16	PRR16	5'UTR		0.001	0.017	0.009	0.026	3.938	0.000172809	0.657116697	ns	+
80	cg16547480	chr19:23445883	open sea	IPO5P1	ZNF724	Body		0.008	0.030	0.015	0.045	3.932	0.000177019	0.657116697	ns	+
81	cg18289156	chr2:84743445	N shore	DNAH6	DNAH6	TSS200		0.001	-0.011	-0.016	-0.005	-3.931	0.000177537	0.657116697	ns	-
82	cg02156776	chr6:71013139	open sea	COL9A1	COL9A1	TSS1500		-0.001	-0.025	-0.037	-0.012	-3.930	0.000178153	0.657116697	ns	-
83	cg25584862	chr11:69706901	island		FGF3			-0.004	-0.021	-0.031	-0.010	-3.927	0.000180093	0.657116697	ns	-
84	cg02793774	chr7:130737598	open sea	LINC-PINT	MKLN1	Body		0.002	-0.031	-0.047	-0.015	-3.926	0.000180463	0.657116697	ns	-
85	cg11489846	chr19:30250286	open sea		C19orf12			-0.001	0.016	0.008	0.025	3.921	0.000183658	0.657765392	ns	+
86	cg08713851	chr9:140392837	N shelf	PNPLA7	PNPLA7	Body		0.004	0.018	0.009	0.027	3.919	0.000184942	0.657765392	ns	+
87	cg08045042	chr11:1046454	open sea		MUC6			0.001	0.036	0.018	0.054	3.907	0.000192921	0.668605298	ns	+
88	cg11723782	chr3:159726973	open sea	IL12A	IL12A	Body		0.009	-0.024	-0.036	-0.012	-3.902	0.000195922	0.668605298	ns	-
89	cg23784400	chr19:19576195	open sea	GATAD2A	GATAD2A	1stExon		0.000	0.017	0.008	0.026	3.901	0.000196728	0.668605298	ns	+
90	cg24107728	chr1:53760337	open sea	LRP8	LRP8	Body		0.010	0.028	0.014	0.043	3.901	0.000197161	0.668605298	ns	+
91	cg10258962	chr7:23526811	N shelf		RPS2P32			-0.002	-0.025	-0.037	-0.012	-3.896	0.000200679	0.668605298	ns	-
92	cg09430505	chr7:22404511	open sea		RAPGEF5			-0.004	-0.017	-0.026	-0.008	-3.892	0.00020315	0.668605298	ns	-
93	cg15898408	chr1:161720174	S shore	DUSP12	DUSP12	Body	promoter-linked	0.000	0.019	0.009	0.028	3.889	0.000205428	0.668605298	ns	+
94	cg01857595	chr14:38016188	open sea	MIPOL1	MIPOL1	3'UTR		-0.003	0.033	0.016	0.051	3.886	0.000207422	0.668605298	ns	+
95	cg07313064	chr7:55072617	island		EGFR			-0.001	-0.019	-0.029	-0.009	-3.885	0.000208083	0.668605298	ns	-
96	cg06405860	chr1:60539458	island	C1orf87	C1orf87	TSS200		-0.001	0.019	0.009	0.029	3.880	0.000211589	0.668605298	ns	+
97	cg15509069	chr7:97080009	open sea		SDHAF3			0.000	-0.014	-0.022	-0.007	-3.880	0.000212035	0.668605298	ns	-
98	cg03945604	chr19:40724609	S shore	TTC9B	TTC9B	TSS1500		0.000	-0.027	-0.041	-0.013	-3.875	0.000215545	0.672738935	ns	-
99	cg10242825	chr1:146697520	open sea	FMO5	FMO5	TSS1500		0.000	0.020	0.010	0.030	3.872	0.000217926	0.673298008	ns	+
100	cg07545195	chr6:34081581	open sea	GRM4	GRM4	Body		0.003	0.022	0.011	0.033	3.865	0.000222945	0.679829044	ns	+
101	cg19205240	chr7:103126837	open sea	RELN	RELN	Body		0.002	0.018	0.009	0.028	3.863	0.000224485	0.679829044	ns	+
102	cg18818644	chr6:147924672	open sea	SAMD5	SAMD5			0.014	0.022	0.011	0.033	3.859	0.000227973	0.680893137	ns	+
103	cg07094075	chr17:1182236	open sea	TRARG1	TRARG1	TSS1500		0.000	0.018	0.009	0.027	3.857	0.000229336	0.680893137	ns	+
104	cg03014236	chr16:25030752	open sea	LOC100421171	ARHGAP17			0.006	-0.031	-0.047	-0.015	-3.853	0.000232586	0.680893137	ns	-
105	cg11389565	chr17:12121413	open sea		MAP2K4			0.001	0.014	0.007	0.021	3.850	0.00023476	0.680893137	ns	+
106	cg10086221	chrX:77126440	open sea	MAGT1	MAGT1	ExonBnd		-0.001	-0.021	-0.032	-0.010	-3.849	0.000235967	0.680893137	ns	-
107	cg17091818	chr1:245242515	open sea	EFCAB2	EFCAB2	Body		-0.005	-0.038	-0.057	-0.018	-3.846	0.00023866	0.682228553	ns	-
108	cg14015300	chr19:55864308	island	COX6B2	COX6B2	3'UTR		0.005	0.016	0.008	0.024	3.839	0.000244129	0.685010573	ns	+
109	cg20516364	chr11:873680	open sea	CHID1	CHID1	Body		0.014	-0.031	-0.047	-0.015	-3.836	0.000247087	0.685010573	ns	-
110	cg00470144	chr3:107723657	open sea		CD47			-0.012	-0.028	-0.042	-0.013	-3.832	0.000250224	0.685010573	ns	-
111	cg00576773	chr15:89909904	N shore	MIR9-3HG	POLG	TSS1500		0.001	-0.025	-0.038	-0.012	-3.832	0.000250298	0.685010573	ns	-
112	cg15590863	chr7:102449340	open sea	FAM185A	FAM185A	Body		0.004	-0.019	-0.029	-0.009	-3.829	0.000252216	0.685010573	ns	-
113	cg21374546	chr10:17555104	open sea		ST8SIA6			-0.006	-0.020	-0.030	-0.009	-3.828	0.000253071	0.685010573	ns	-
114	cg18108844	chr1:2716235	N shore	TTC34	TTC34			0.002	0.017	0.008	0.026	3.826	0.000255455	0.68539961	ns	+
115	cg10923455	chr8:127804315	open sea	RP11-351C8.1	LRATD2			0.005	-0.021	-0.032	-0.010	-3.819	0.000260986	0.689273758	ns	-
116	cg01168822	chr6:33334703	open sea	LYPLA2P1	KIFC1	TSS1500		0.007	0.031	0.015	0.047	3.819	0.000261406	0.689273758	ns	+
117	cg09066963	chr1:33070069	open sea	ZBTB8A	ZBTB8A	3'UTR		-0.013	-0.037	-0.057	-0.018	-3.815	0.000265346	0.693683242	ns	-
118	cg16362480	chr16:30077084	island	ALDOA	ALDOA	5'UTR	promoter-linked	-0.002	0.031	0.015	0.048	3.807	0.000272294	0.705813427	ns	+
119	cg24446178	chr12:100750702	open sea	SLC17A8	SLC17A8	TSS200		0.009	0.037	0.018	0.057	3.796	0.000282931	0.719899793	ns	+
120	cg07482800	chr19:14661123	open sea	TECR	TECR	Body		0.007	0.015	0.007	0.024	3.793	0.000285381	0.719899793	ns	+
121	cg12557562	chrX:106196726	open sea	MORC4	MORC4	Body		0.007	0.021	0.010	0.032	3.792	0.000286287	0.719899793	ns	+
122	cg26480862	chr22:28839667	open sea	TTC28	TTC28	Body	unknown cell specific	-0.004	0.026	0.012	0.039	3.790	0.00028861	0.719899793	ns	+
123	cg19839614	chrX:50212149	N shore	DGKK	DGKK	Body		-0.001	0.021	0.010	0.032	3.787	0.00029155	0.719899793	ns	+
124	cg06562969	chr13:43567153	S shore	EPSTI1	EPSTI1	TSS1500	promoter-linked	0.004	0.030	0.014	0.046	3.782	0.000296629	0.719899793	ns	+
125	cg23025942	chr1:154325339	open sea		ATP8B2		unknown cell specific	-0.003	0.034	0.016	0.052	3.776	0.000302655	0.719899793	ns	+
126	cg13304093	chr3:62360058	S shore	FEZF2	FEZF2	TSS1500		0.003	0.021	0.010	0.033	3.775	0.000303955	0.719899793	ns	+
127	cg06254264	chr22:39189559	N shore	DNAL4	DNAL4	5'UTR		0.006	-0.020	-0.030	-0.009	-3.774	0.0003052	0.719899793	ns	-
128	cg09392245	chr16:3331498	N shore	ZNF263	ZNF263			-0.002	-0.024	-0.036	-0.011	-3.773	0.000306034	0.719899793	ns	-
129	cg02636808	chr10:134535351	N shore	INPP5A	INPP5A	Body		0.009	0.038	0.018	0.057	3.770	0.000309508	0.719899793	ns	+
130	cg20019489	chr20:57414351	N shore	GNAS	GNAS	Body	unknown cell specific	0.008	0.031	0.014	0.047	3.769	0.000310263	0.719899793	ns	+
131	cg17739400	chr7:24855509	open sea	OSBPL3	OSBPL3	Body		0.002	-0.016	-0.024	-0.007	-3.768	0.000310835	0.719899793	ns	-
132	cg25246113	chr19:33523251	open sea	RHPN2	RHPN2	Body		0.000	0.018	0.009	0.028	3.766	0.00031292	0.719899793	ns	+
133	cg20052923	chr1:203028185	open sea	PPFIA4	PPFIA4	Body		-0.002	0.019	0.009	0.029	3.764	0.000315874	0.719899793	ns	+
134	cg25472462	chr7:2516194	open sea	GRIFIN	GRIFIN	TSS200	unknown cell specific	0.004	0.021	0.010	0.031	3.761	0.000318234	0.719899793	ns	+
135	cg23155089	chr6:44698962	S shelf		SUPT3H			-0.001	-0.016	-0.025	-0.008	-3.760	0.000319169	0.719899793	ns	-
136	cg18452703	chr10:102949930	open sea	LINC01514	LBX1			0.000	-0.027	-0.041	-0.012	-3.753	0.000327469	0.719899793	ns	-
137	cg21552319	chr6:24356996	N shore	DCDC2	DCDC2	Body		0.006	-0.019	-0.030	-0.009	-3.753	0.000327624	0.719899793	ns	-
138	cg17969437	chr3:49755497	N shore	AMIGO3	AMIGO3	1stExon		0.004	0.015	0.007	0.023	3.752	0.000328392	0.719899793	ns	+
139	cg25645198	chrX:30675444	S shelf	GK	GK	Body		0.004	-0.023	-0.035	-0.011	-3.752	0.00032861	0.719899793	ns	-
140	cg05588508	chr3:120007636	S shelf		LRRC58			-0.006	-0.029	-0.044	-0.014	-3.750	0.000330411	0.719899793	ns	-
141	cg22232830	chr4:128541611	N shelf		INTU			0.005	-0.015	-0.022	-0.007	-3.748	0.000333261	0.719899793	ns	-
142	cg04233211	chrX:35937394	open sea	CFAP47	CFAP47	TSS1500		0.002	-0.016	-0.024	-0.007	-3.745	0.000336249	0.719899793	ns	-
143	cg03584693	chr3:101280436	N shore	TRMT10C	TRMT10C	TSS1500	promoter-linked	-0.020	0.045	0.021	0.069	3.744	0.000337559	0.719899793	ns	+
144	cg23808031	chr15:74659841	S shore	CYP11A1	CYP11A1	TSS1500		0.018	0.031	0.014	0.047	3.743	0.000338923	0.719899793	ns	+
145	cg26784032	chr1:215697002	open sea		KCTD3			0.001	-0.040	-0.061	-0.019	-3.735	0.00034885	0.731363675	ns	-
146	cg08651540	chr10:111171828	open sea		XPNPEP1			0.013	0.027	0.012	0.041	3.732	0.000351634	0.731363675	ns	+
147	cg27375378	chr17:77806402	island		CBX4		unknown cell specific	0.002	-0.018	-0.028	-0.008	-3.729	0.000354622	0.731363675	ns	-
148	cg16549396	chr13:68765417	open sea		ELL2P3			-0.003	-0.027	-0.041	-0.012	-3.729	0.00035564	0.731363675	ns	-
149	cg19791321	chr8:126625800	open sea		TRIB1			0.006	0.024	0.011	0.037	3.726	0.000358974	0.731363675	ns	+
150	cg14899504	chr14:71164558	open sea	LINC01269	TTC9	TSS1500		0.003	0.013	0.006	0.020	3.724	0.00036161	0.731363675	ns	+
151	cg00056497	chr10:134785521	open sea	LINC01168	CFAP46			0.007	0.027	0.013	0.042	3.723	0.000362513	0.731363675	ns	+
152	cg07544459	chr12:131864810	open sea		ADGRD1			0.010	0.024	0.011	0.037	3.721	0.000364584	0.731363675	ns	+
153	cg02466544	chr2:74641453	N shore	C2orf81	C2orf81	3'UTR		0.007	0.025	0.012	0.038	3.720	0.00036584	0.731363675	ns	+
154	cg06578561	chr8:139508625	N shore	FAM135B	FAM135B	5'UTR		0.009	-0.044	-0.067	-0.020	-3.717	0.00037045	0.73319549	ns	-
155	cg13786031	chr9:91424986	open sea		C9orf47			-0.005	-0.027	-0.042	-0.013	-3.713	0.000375427	0.73319549	ns	-
156	cg17008375	chr7:101754831	open sea	CUX1	CUX1	Body		0.000	-0.028	-0.042	-0.013	-3.712	0.000375988	0.73319549	ns	-
157	cg08467089	chr1:46915530	S shore	LINC01398	FAAH	TSS200		0.003	0.018	0.008	0.027	3.710	0.00037851	0.73319549	ns	+
158	cg07786370	chr1:22182005	open sea	HSPG2	HSPG2	ExonBnd		0.006	0.015	0.007	0.024	3.710	0.000378741	0.73319549	ns	+
159	cg06349981	chr2:10443745	island	HPCAL1	HPCAL1	TSS200	promoter-linked	-0.003	0.016	0.008	0.025	3.700	0.000391509	0.752562365	ns	+

160	cg13801500	chr11:35039550	open sea	PDHX	PDHX			0.002	-0.019	-0.029	-0.009	-3.698	0.000393666	0.752562365	ns	-
161	cg26307359	chr17:27332646	island	SEZ6	SEZ6	Body		-0.005	0.018	0.008	0.027	3.689	0.000406771	0.769937414	ns	+
162	cg09987128	chr9:84602238	open sea	SPATA31D1	SPATA31D1	TSS1500		-0.003	-0.029	-0.044	-0.013	-3.688	0.000408214	0.769937414	ns	-
163	cg23094614	chr2:36795418	open sea	FEZ2	FEZ2	Body		0.006	-0.028	-0.044	-0.013	-3.682	0.000416319	0.769937414	ns	-
164	cg03486475	chr5:140796240	N shore	PCDHGA4	PCDHGA4	Body		0.014	0.028	0.013	0.043	3.679	0.000419914	0.769937414	ns	+
165	cg05662676	chr6:37170837	open sea		TMEM217		unknown cell specific	0.011	0.036	0.017	0.056	3.678	0.000422553	0.769937414	ns	+
166	cg26212904	chr17:5608761	open sea		WSCD1			-0.005	0.030	0.014	0.046	3.677	0.000422943	0.769937414	ns	+
167	cg18335505	chr2:131514588	S shore	AMER3	AMER3	5'UTR		0.002	-0.016	-0.024	-0.007	-3.674	0.000426725	0.769937414	ns	-
168	cg14422899	chr14:95538385	open sea		DICER1			0.004	-0.021	-0.033	-0.010	-3.671	0.000431404	0.769937414	ns	-
169	cg06418169	chrX:153055275	N shelf	IDH3G	IDH3G	ExonBnd		0.003	0.013	0.006	0.021	3.671	0.000432183	0.769937414	ns	+
170	cg11003725	chr14:105349607	N shore	CEP170B	CEP170B	Body	unknown cell specific	0.005	-0.017	-0.026	-0.008	-3.670	0.000433651	0.769937414	ns	-
171	cg02633371	chr17:35298618	island	LHX1	LHX1	Body		-0.002	0.017	0.008	0.027	3.669	0.000433983	0.769937414	ns	+
172	cg16525762	chr5:134294026	open sea	PCBD2	PCBD2	Body		0.015	0.027	0.012	0.041	3.669	0.000434058	0.769937414	ns	+
173	cg09580244	chr16:188732	island	NPRL3	NPRL3	TSS200	promoter-linked	-0.005	0.021	0.010	0.032	3.666	0.000439401	0.769937414	ns	+
174	cg15547852	chr6:11606739	open sea	RP11-679B17.2	TMEM170B			0.007	0.026	0.012	0.040	3.665	0.000440148	0.769937414	ns	+
175	cg22139852	chr1:183605108	island	RGL1	RGL1	TSS200	promoter-linked	0.007	0.018	0.008	0.028	3.661	0.000446768	0.769937414	ns	+
176	cg27080361	chr5:149447589	open sea	CSF1R	CSF1R	Body		0.006	0.019	0.009	0.030	3.661	0.000446919	0.769937414	ns	+
177	cg09435890	chr16:90144788	S shore	PRDM7	PRDM7		promoter-linked	-0.006	-0.023	-0.036	-0.010	-3.660	0.000448024	0.769937414	ns	-
178	cg06572849	chr11:45354316	open sea		SYT13			-0.003	-0.016	-0.025	-0.007	-3.660	0.000448065	0.769937414	ns	-
179	cg18200075	chr1:161706293	open sea		FCRLB			0.008	0.021	0.010	0.033	3.651	0.000461059	0.787838613	ns	+
180	cg10285992	chr1:157369614	open sea		FCRL5			0.000	0.025	0.011	0.038	3.647	0.000468358	0.789306319	ns	+
181	cg05708452	chr8:86377153	S shore	CA2	CA2	TSS1500		0.000	-0.037	-0.058	-0.017	-3.645	0.000470743	0.789306319	ns	-
182	cg16872437	chr17:4711346	S shore	PLD2	PLD2	Body		0.003	0.015	0.007	0.024	3.644	0.000472188	0.789306319	ns	+
183	cg05081908	chr4:1020943	N shore	RP11-460I19.2	FGFRL1			0.001	-0.016	-0.025	-0.007	-3.634	0.000489248	0.789306319	ns	-
184	cg00039452	chr8:16972354	open sea	MICU3	MICU3	Body		-0.002	-0.027	-0.041	-0.012	-3.633	0.000490062	0.789306319	ns	-
185	cg05542299	chr6:168895488	open sea	SMOC2	SMOC2	Body		0.002	0.016	0.007	0.025	3.633	0.000490159	0.789306319	ns	+
186	cg16807457	chrX:133371689	island	CCDC160	CCDC160	5'UTR		-0.003	0.050	0.023	0.078	3.631	0.000494504	0.789306319	ns	+
187	cg27066201	chr5:140528841	N shelf	PCDHB6	PCDHB6	TSS1500		0.000	0.025	0.011	0.039	3.630	0.000495763	0.789306319	ns	+
188	cg13843366	chr12:2429573	open sea	CACNA1C	CACNA1C	Body		0.010	0.023	0.011	0.036	3.628	0.000497751	0.789306319	ns	+
189	cg09894758	chr19:32743570	open sea		ZNF507			0.002	0.020	0.009	0.031	3.628	0.000498676	0.789306319	ns	+
190	cg07771859	chr19:9534005	open sea	ZNF266	ZNF266	5'UTR		0.007	-0.020	-0.030	-0.009	-3.626	0.000501591	0.789306319	ns	-
191	cg22308949	chr2:206628553	open sea	NRP2	NRP2	Body		-0.008	0.026	0.012	0.040	3.626	0.000502349	0.789306319	ns	+
192	cg20731529	chr2:200296767	open sea	SATB2	SATB2	Body		-0.001	-0.017	-0.026	-0.008	-3.624	0.000504667	0.789306319	ns	-
193	cg12774902	chr11:3687530	N shore	CHRNA10	CHRNA10	Body		-0.002	-0.023	-0.036	-0.010	-3.624	0.000505537	0.789306319	ns	-
194	cg03490719	chr11:61328702	open sea	SYT7	SYT7	Body		0.004	0.015	0.007	0.024	3.622	0.000507792	0.789306319	ns	+
195	cg26036443	chr19:48216402	N shore	EHD2	EHD2	TSS200		0.001	0.015	0.007	0.023	3.621	0.000509508	0.789306319	ns	+
196	cg11450744	chr17:30347981	open sea	LRRC37B	LRRC37B	TSS200		0.000	-0.020	-0.031	-0.009	-3.621	0.000510245	0.789306319	ns	-
197	cg24318558	chrX:106959829	island	TSC22D3	TSC22D3	Body	promoter-linked	0.002	-0.018	-0.028	-0.008	-3.620	0.000511852	0.789306319	ns	-
198	cg09815769	chr1:119539738	N shelf		TBX15			-0.005	-0.034	-0.053	-0.015	-3.620	0.000512026	0.789306319	ns	-
199	cg11697653	chr10:122740406	S shore	MIR5694	WDR11	Body		0.005	0.020	0.009	0.031	3.619	0.000513529	0.789306319	ns	+
200	cg04589118	chr4:2848840	S shelf	ADD1	ADD1	5'UTR		0.000	-0.016	-0.024	-0.007	-3.613	0.000523923	0.798161373	ns	-
201	cg01738095	chr2:26101831	S shore	ASXL2	ASXL2	TSS1500	promoter-linked	-0.007	0.031	0.014	0.048	3.612	0.000525281	0.798161373	ns	+
202	cg03529987	chr4:102518728	open sea	BANK1	BANK1			0.001	0.020	0.009	0.031	3.609	0.000531081	0.798161373	ns	+
203	cg18450270	chr4:8308672	S shore	HTRA3	HTRA3	3'UTR		0.004	0.013	0.006	0.020	3.609	0.00053154	0.798161373	ns	+
204	cg02142926	chr6:134994674	open sea		FAM8A6P			0.001	0.037	0.016	0.057	3.608	0.000532337	0.798161373	ns	+
205	cg09476154	chr1:43472373	N shore		SLC2A1			0.003	0.023	0.010	0.035	3.607	0.000535238	0.798595892	ns	+
206	cg16735303	chr12:64942835	open sea		TBK1			0.000	0.015	0.007	0.024	3.603	0.000541471	0.803974023	ns	+
207	cg05684381	chr11:70677281	open sea	SHANK2	SHANK2	Body		0.001	-0.030	-0.047	-0.013	-3.601	0.000545184	0.804761122	ns	-
208	cg04463549	chrX:70828492	open sea	GCNA	GCNA	Body		-0.002	-0.020	-0.031	-0.009	-3.599	0.000548764	0.804761122	ns	-
209	cg07590058	chr14:67805064	open sea	ATP6V1D	ATP6V1D	3'UTR		0.001	-0.025	-0.039	-0.011	-3.597	0.000552833	0.804761122	ns	-
210	cg26838617	chr7:22221702	open sea	RAPGEF5	RAPGEF5	Body		0.012	0.037	0.017	0.057	3.595	0.000556151	0.804761122	ns	+
211	cg04508467	chr19:50931622	island	SPIB	SPIB	3'UTR		0.001	0.032	0.014	0.050	3.593	0.000561161	0.804761122	ns	+
212	cg15161171	chr2:38634397	open sea	AC016995.3	ATL2			0.004	0.019	0.008	0.029	3.592	0.0005622	0.804761122	ns	+
213	cg23617039	chr16:333737	S shore	PDIA2	PDIA2	Body	unknown cell specific	-0.007	-0.023	-0.036	-0.010	-3.588	0.00056895	0.804761122	ns	-
214	cg15360665	chr16:3006598	open sea		FLYWCH1			0.005	0.028	0.012	0.043	3.588	0.000569216	0.804761122	ns	+
215	cg09314675	chr15:99947826	open sea		LRRC28			0.008	0.022	0.010	0.034	3.588	0.000569252	0.804761122	ns	+
216	cg06665305	chr2:110372933	island	SOWAHC	SOWAHC	1stExon		0.002	0.013	0.006	0.020	3.587	0.000570573	0.804761122	ns	+
217	cg18246640	chr10:112136000	open sea		SMNDC1			0.000	-0.021	-0.033	-0.009	-3.587	0.000570943	0.804761122	ns	-
218	cg21535156	chr7:1499152	S shore	MICALL2	MICALL2	TSS200	promoter-linked	-0.003	0.023	0.010	0.035	3.581	0.00058296	0.809303141	ns	+
219	cg19729930	chr2:74357872	island	RP11-287D1.4	BOLA3			0.007	0.023	0.010	0.035	3.573	0.000597851	0.809303141	ns	+
220	cg01844176	chr12:27176208	S shore	MED21	MED21	Body	promoter-linked	-0.007	-0.043	-0.067	-0.019	-3.571	0.000602387	0.809303141	ns	-
221	cg22607472	chr14:73086455	open sea	DPF3	DPF3	Body		0.000	-0.020	-0.032	-0.009	-3.569	0.00060518	0.809303141	ns	-
222	cg16850704	chr5:129579574	open sea		CHSY3			0.000	0.020	0.009	0.031	3.567	0.00060942	0.809303141	ns	+
223	cg03618256	chr2:19562750	island		OSR1			-0.006	-0.028	-0.044	-0.012	-3.566	0.00061257	0.809303141	ns	-
224	cg07974485	chr11:1025843	S shore	MUC6	MUC6	Body		0.007	0.021	0.009	0.033	3.565	0.000613629	0.809303141	ns	+
225	cg21579539	chr18:43751229	N shelf		C18orf25			0.004	-0.040	-0.062	-0.017	-3.564	0.000616011	0.809303141	ns	-
226	cg10875499	chr7:1428900	open sea		MICALL2			0.011	-0.027	-0.042	-0.012	-3.564	0.000616211	0.809303141	ns	-
227	cg06660395	chr4:1396306	island		NKX1-1		unknown cell specific	-0.004	-0.018	-0.029	-0.008	-3.564	0.000616602	0.809303141	ns	-
228	cg05097579	chr3:136750029	N shore		IL20RB			0.016	-0.028	-0.043	-0.012	-3.559	0.000625852	0.809303141	ns	-
229	cg23982638	chr1:101897715	open sea		S1PR1		unknown cell specific	0.008	0.019	0.008	0.030	3.559	0.000626417	0.809303141	ns	+
230	cg23619970	chr4:148686219	open sea	ARHGAP10	ARHGAP10	Body		0.008	-0.060	-0.093	-0.026	-3.559	0.00062763	0.809303141	ns	-
231	cg26750133	chr1:47644648	open sea	LINC00853	PDZK1IP1	TSS1500	promoter-linked	0.001	0.025	0.011	0.039	3.558	0.000628794	0.809303141	ns	+
232	cg06125671	chr11:34385288	open sea		ABTB2			0.006	-0.045	-0.069	-0.020	-3.558	0.000629683	0.809303141	ns	-
233	cg03583734	chr19:46390327	S shore	IRF2BP1	IRF2BP1	TSS1500	promoter-linked	0.000	0.029	0.013	0.046	3.555	0.000634476	0.809303141	ns	+
234	cg07694417	chr14:91730592	N shore		CCDC88C		unknown cell specific	0.003	0.045	0.020	0.070	3.554	0.000637656	0.809303141	ns	+
235	cg04070142	chr12:21566749	open sea	SLCO1A2	SLCO1A2			0.002	0.017	0.007	0.026	3.553	0.00063823	0.809303141	ns	+
236	cg08957544	chr9:128191269	open sea		MAPKAP1			0.002	0.018	0.008	0.028	3.552	0.000639966	0.809303141	ns	+
237	cg12228863	chr13:95069222	open sea		GPC6			0.010	0.029	0.013	0.045	3.552	0.000640657	0.809303141	ns	+
238	cg13358038	chr12:1023702	open sea	RAD52	RAD52	ExonBnd		-0.004	-0.025	-0.039	-0.011	-3.551	0.000642386	0.809303141	ns	-
239	cg00901987	chrX:128892191	open sea	XPNPEP2	XPNPEP2	Body	unknown cell specific	0.002	-0.023	-0.036	-0.010	-3.551	0.000642412	0.809303141	ns	-
240	cg16905966	chr9:80548129	open sea	GNAQ	GNAQ	Body		0.010	-0.022	-0.034	-0.010	-3.550	0.000645028	0.809303141	ns	-

241	cg11107560	chr2:96907142	open sea	STARD7	STARD7	Body		0.006	-0.027	-0.042	-0.012	-3.548	0.000648907	0.809303141	ns	-
242	cg18390345	chr10:98939912	open sea	SLIT1	SLIT1	Body		0.007	0.027	0.012	0.043	3.548	0.000650386	0.809303141	ns	+
243	cg08026930	chr19:56459137	open sea	NLRP8	NLRP8	TSS200		-0.003	0.021	0.009	0.033	3.546	0.000652437	0.809303141	ns	+
244	cg11664139	chr2:175204249	N shore		SP9		unknown cellspecific	-0.013	-0.041	-0.063	-0.018	-3.546	0.000653643	0.809303141	ns	-
245	cg21279806	chr6:169654719	open sea	THBS2	THBS2	TSS1500		0.001	-0.016	-0.026	-0.007	-3.544	0.00065812	0.809303141	ns	-
246	cg06343109	chr5:496081	S shore	SLC9A3	SLC9A3	Body		0.000	-0.014	-0.021	-0.006	-3.544	0.000658367	0.809303141	ns	-
247	cg23749317	chr13:85953968	open sea	LINC00351	SLITRK6	Body		-0.001	-0.017	-0.026	-0.007	-3.544	0.000658372	0.809303141	ns	-
248	cg22356726	chr10:91597320	island	LINC00865	KIF20B		unknown cellspecific	-0.006	-0.016	-0.025	-0.007	-3.543	0.000660557	0.809303141	ns	-
249	cg26382412	chr7:24803104	open sea		GSDME			0.001	-0.024	-0.037	-0.010	-3.542	0.000663069	0.809303141	ns	-
250	cg22348290	chr8:72459499	N shore	EYA1	EYA1			0.000	0.034	0.015	0.053	3.541	0.000664069	0.809303141	ns	+
251	cg10380652	chr1:65612850	N shore	AK4	AK4	TSS1500		-0.001	0.022	0.009	0.034	3.541	0.000664127	0.809303141	ns	+
252	cg08828694	chr1:33636091	open sea	TRIM62	TRIM62	Body		0.014	0.025	0.011	0.040	3.535	0.000676881	0.819558435	ns	+
253	cg14667769	chr10:134769136	open sea	LINC01166	CFAP46			0.002	0.018	0.008	0.028	3.535	0.000677901	0.819558435	ns	+
254	cg09438837	chr15:74537916	island	CCDC33	CCDC33	Body		-0.004	0.018	0.008	0.029	3.533	0.000681797	0.821023439	ns	+
255	cg14018454	chr9:16310537	open sea		C9orf92			0.002	0.016	0.007	0.026	3.528	0.000693492	0.82971122	ns	+
256	cg09470850	chr21:33467447	open sea	LINC00159	HUNK			0.000	-0.015	-0.024	-0.007	-3.524	0.000702386	0.82971122	ns	-
257	cg07841848	chr5:172676916	S shelf		NKX2-5			0.004	0.020	0.009	0.031	3.522	0.000705787	0.82971122	ns	+
258	cg25510201	chr5:175980531	open sea	CDHR2	CDHR2	5'UTR		0.011	0.024	0.010	0.037	3.522	0.000706636	0.82971122	ns	+
259	cg13916931	chr7:44162801	N shore	POLD2	POLD2	5'UTR	promoter-linked	0.001	0.022	0.010	0.035	3.522	0.000707106	0.82971122	ns	+
260	cg12255710	chr1:90900170	open sea		BARHL2			-0.010	-0.036	-0.056	-0.015	-3.521	0.000709634	0.82971122	ns	-
261	cg06300961	chr11:1459507	open sea	BRSK2	BRSK2	Body		0.004	0.015	0.006	0.023	3.520	0.000710474	0.82971122	ns	+
262	cg20971488	chr1:228786295	S shore	RHOU	RHOU	Body		-0.007	-0.016	-0.025	-0.007	-3.519	0.000712837	0.82971122	ns	-
263	cg19776942	chr1:32714164	island	FAM167B	FAM167B	Body		0.000	-0.016	-0.024	-0.007	-3.519	0.000713426	0.82971122	ns	-
264	cg06024411	chr2:112895681	N shore	FBLN7	FBLN7	TSS1500	unknown cellspecific	-0.002	0.020	0.009	0.032	3.515	0.000722183	0.83347741	ns	+
265	cg02964602	chr2:203130213	N shore	NOP58	NOP58	TSS1500	promoter-linked	-0.002	-0.029	-0.045	-0.012	-3.516	0.000722233	0.83347741	ns	-
266	cg13617521	chrX:153996788	open sea	DKC1	DKC1	Body		0.003	-0.024	-0.038	-0.010	-3.514	0.000724839	0.83347741	ns	-
267	cg26276120	chr12:6977747	S shore	TPI1	TPI1	Body	promoter-linked	0.006	-0.020	-0.032	-0.009	-3.512	0.000730395	0.834267942	ns	-
268	cg15194638	chrX:102510578	open sea	TCEAL8	TCEAL8	TSS1500		0.019	0.049	0.021	0.076	3.511	0.000733716	0.834267942	ns	+
269	cg08984281	chr1:89546987	open sea		GBP1			0.000	0.016	0.007	0.025	3.510	0.000735007	0.834267942	ns	+
270	cg13033387	chr16:65943439	open sea		CDH5			0.000	-0.015	-0.024	-0.007	-3.509	0.000736436	0.834267942	ns	-
271	cg13291298	chr15:69690671	open sea	PAQR5	PAQR5	Body		-0.006	-0.019	-0.030	-0.008	-3.508	0.00073957	0.834726403	ns	-
272	cg03599590	chr3:112282031	S shore	SLC35A5	SLC35A5	5'UTR		0.006	-0.031	-0.048	-0.013	-3.507	0.000743682	0.835796403	ns	-
273	cg08516846	chr14:106450381	open sea		IGHV1-2			0.006	0.011	0.005	0.018	3.505	0.000745983	0.835796403	ns	+
274	cg03447137	chr8:61194801	S shore	CA8	CA8	TSS1500		0.001	-0.011	-0.017	-0.005	-3.504	0.000749573	0.835997059	ns	-
275	cg09451427	chr3:50488230	open sea	CACNA2D2	CACNA2D2	Body	unknown cellspecific	0.000	-0.040	-0.062	-0.017	-3.501	0.000758166	0.835997059	ns	-
276	cg12449058	chr14:61125920	S shore	SIX1	SIX1			0.001	0.034	0.015	0.054	3.501	0.000758913	0.835997059	ns	+
277	cg02663909	chr5:2180505	S shore		IRX4			0.001	-0.013	-0.021	-0.006	-3.498	0.00076383	0.835997059	ns	-
278	cg08082389	chr14:60561405	S shelf	PCNX4	PCNX4	5'UTR		-0.001	-0.024	-0.037	-0.010	-3.498	0.000765151	0.835997059	ns	-
279	cg19925780	chr1:101509557	open sea	LOC102606465	DPH5			-0.004	0.032	0.014	0.050	3.498	0.000765311	0.835997059	ns	+
280	cg14153452	chr22:38862568	N shore	KDELR3	KDELR3	TSS1500		0.002	-0.019	-0.030	-0.008	-3.496	0.000768225	0.835997059	ns	-
281	cg11382589	chr17:22052191	island		MTRNR2L1			0.000	-0.014	-0.023	-0.006	-3.495	0.000772497	0.835997059	ns	-
282	cg20401896	chr2:63855118	open sea	WDPCP	WDPCP			-0.007	-0.024	-0.037	-0.010	-3.493	0.000775873	0.835997059	ns	-
283	cg14211350	chr7:157670094	island	PTPRN2	PTPRN2	Body		0.003	-0.024	-0.038	-0.010	-3.490	0.000785487	0.835997059	ns	-
284	cg15388974	chr14:30291201	open sea	PRKD1	PRKD1	Body		0.000	-0.013	-0.021	-0.006	-3.489	0.000786653	0.835997059	ns	-
285	cg25545391	chr4:114936716	open sea		ARSJ			0.002	-0.025	-0.040	-0.011	-3.486	0.00079422	0.835997059	ns	-
286	cg11828983	chr14:104734272	open sea	LINC02691	KIF26A			-0.002	-0.026	-0.041	-0.011	-3.486	0.000794347	0.835997059	ns	-
287	cg20398969	chr22:48109889	open sea	LOC284930	TBC1D22A	Body		0.004	0.014	0.006	0.022	3.485	0.000797153	0.835997059	ns	+
288	cg20776758	chr12:115980536	open sea		MED13L			0.006	0.030	0.013	0.047	3.484	0.000799851	0.835997059	ns	+
289	cg15056189	chr12:49176428	island	ADCY6	ADCY6	1stExon		0.001	0.020	0.008	0.031	3.483	0.000803695	0.835997059	ns	+
290	cg16932050	chr7:97953404	open sea	BAIAP2L1	BAIAP2L1	Body		-0.004	-0.012	-0.019	-0.005	-3.482	0.000803938	0.835997059	ns	-
291	cg23313924	chr14:22950829	open sea	TRA	TRA			-0.010	-0.025	-0.039	-0.011	-3.482	0.000805517	0.835997059	ns	-
292	cg16285941	chr8:144212930	open sea		LY6H			0.008	0.017	0.007	0.027	3.481	0.000807445	0.835997059	ns	+
293	cg27103348	chr21:46321529	island	ITGB2	ITGB2	Body		0.007	0.019	0.008	0.031	3.481	0.000807974	0.835997059	ns	+
294	cg27483478	chr5:175969167	N shore	CDHR2	CDHR2	TSS1500		-0.003	-0.013	-0.020	-0.005	-3.480	0.000809145	0.835997059	ns	-
295	cg18984151	chr3:47555476	S shore	ELP6	ELP6	TSS1500	promoter-linked	0.004	-0.023	-0.037	-0.010	-3.480	0.000810236	0.835997059	ns	-
296	cg18086386	chr5:162516650	open sea	RP11-541P9.3	CCNG1			0.009	-0.019	-0.030	-0.008	-3.478	0.000815591	0.835997059	ns	-
297	cg17700835	chr6:27390890	open sea		ZNF184			0.005	-0.023	-0.036	-0.010	-3.476	0.000819939	0.835997059	ns	-
298	cg13820942	chr1:109642776	island	SCARNA2	TMEM167B	TSS200	promoter-linked	0.008	0.023	0.010	0.036	3.475	0.000824145	0.835997059	ns	+
299	cg27431761	chr11:8361327	open sea		STK33			0.008	-0.037	-0.058	-0.016	-3.474	0.000827345	0.835997059	ns	-
300	cg27398117	chr5:56612736	open sea		GPBP1			-0.002	0.020	0.009	0.032	3.473	0.000830164	0.835997059	ns	+
301	cg02896796	chr15:52797352	open sea	MYO5A	MYO5A	Body		0.007	0.029	0.012	0.045	3.471	0.000835439	0.835997059	ns	+
302	cg26105725	chr6:27511112	open sea		TRS-AGA1-1			-0.001	-0.021	-0.033	-0.009	-3.471	0.00083554	0.835997059	ns	-
303	cg02930951	chr20:48806066	N shore	CEBPB	CEBPB	TSS1500		-0.003	0.017	0.007	0.027	3.469	0.000840091	0.835997059	ns	+
304	cg19801825	chr6:97945804	open sea	LOC101927314	MMS22L	Body	unknown cellspecific	0.001	-0.019	-0.030	-0.008	-3.468	0.000841671	0.835997059	ns	-
305	cg05861228	chr11:33522693	open sea	KIAA1549L	KIAA1549L			0.004	0.037	0.016	0.058	3.466	0.000848111	0.835997059	ns	+

PSSI-I: PTSD

rank	cpg	cpg.position	region	annotation	gene	gene.group	gene.feature	AveExpr	logFC	CI.L	CI.R	t	P.Value	adj.P.Val	fdr	sign
1	cg02192673	chr4:72897565	island	NPFFR2	NPFFR2	1stExon		0.393	-0.018	-0.024	-0.012	-5.770	1.35E-07	0.041278909	**	-
2	cg14185541	chr13:95366203	S shore	SOX21	SOX21			-1.308	0.022	0.014	0.030	5.423	5.76E-07	0.08806017	*	+
3	cg14470223	chr15:69431645	open sea	MIR548H4	GLCE	Body		1.650	0.011	0.007	0.016	5.234	1.24E-06	0.119202754	ns	+
4	cg02255004	chr4:80748660	open sea	PCAT4	ANTXR2	Body		0.948	0.011	0.007	0.015	5.105	2.09E-06	0.119202754	ns	+
5	cg08564625	chr8:134263111	open sea	NDRG1	NDRG1	Body		-1.411	0.012	0.007	0.017	5.092	2.21E-06	0.119202754	ns	+
6	cg24912565	chr1:155189890	open sea	GBAP1	MTX1	Body		0.329	0.015	0.009	0.020	5.078	2.34E-06	0.119202754	ns	+
7	cg04350355	chr22:36962768	S shore	CACNG2	CACNG2	Body	unknown cellspecific	2.954	-0.017	-0.024	-0.010	-5.002	3.17E-06	0.138332706	ns	-
8	cg06565605	chr22:49485048	N shelf		TAFA5			1.676	0.013	0.008	0.019	4.881	5.10E-06	0.187174574	ns	+
9	cg21046355	chr19:52772717	island	ZNF766	ZNF766	TSS200	non-gene-linked	-3.274	0.018	0.011	0.025	4.823	6.40E-06	0.187174574	ns	+
10	cg06170374	chr11:37533403	open sea		IFTAP			2.061	-0.022	-0.031	-0.013	-4.787	7.38E-06	0.187174574	ns	-
11	cg02712878	chr3:138327370	N shore	FAIM	FAIM	TSS200	promoter-linked	-1.742	-0.028	-0.040	-0.017	-4.786	7.44E-06	0.187174574	ns	-
12	cg01835527	chr5:148214451	open sea		ADRB2			2.733	0.015	0.009	0.021	4.784	7.46E-06	0.187174574	ns	+
13	cg01461733	chr16:3018302	island	PAQR4	PAQR4	TSS1500		1.491	0.017	0.010	0.025	4.767	7.96E-06	0.187174574	ns	+
14	cg00305797	chr4:186989236	open sea	TLR3	TLR3	TSS1500	promoter-linked cellspecific	0.251	0.019	0.011	0.027	4.708	1.00E-05	0.202161384	ns	+
15	cg13896387	chr11:122357689	open sea		UBASH3B			1.799	0.015	0.009	0.022	4.679	1.12E-05	0.202161384	ns	+
16	cg05417618	chr1:145630725	open sea	RNF115	RNF115	Body		-1.417	0.021	0.012	0.029	4.674	1.14E-05	0.202161384	ns	+
17	cg16341836	chr10:90641389	S shore	STAMBPL1	STAMBPL1	5'UTR		-0.098	0.018	0.011	0.026	4.671	1.16E-05	0.202161384	ns	+
18	cg24229052	chr14:95862525	open sea		SYNE3			-1.997	0.014	0.008	0.020	4.663	1.19E-05	0.202161384	ns	+
19	cg15717212	chr19:43942802	open sea		TEX101			-1.821	0.021	0.012	0.030	4.645	1.28E-05	0.205529093	ns	+
20	cg09186101	chr17:80197062	S shore	SLC16A3	SLC16A3	3'UTR	promoter-linked cellspecific	0.717	0.011	0.006	0.016	4.629	1.36E-05	0.207360933	ns	+
21	cg24657846	chr8:17628113	open sea	MTUS1	MTUS1	5'UTR		0.402	0.011	0.006	0.016	4.601	1.51E-05	0.219796501	ns	+
22	cg18412223	chrX:133810956	open sea	PLAC1	PLAC1			1.310	0.015	0.008	0.021	4.581	1.63E-05	0.226468683	ns	+
23	cg24276541	chr11:59806469	open sea	OOSP2	OOSP2	TSS1500		-1.243	0.012	0.007	0.017	4.509	2.14E-05	0.27659198	ns	+
24	cg08984179	chr10:119117730	open sea	PDZD8	PDZD8	Body		0.637	0.017	0.010	0.025	4.489	2.31E-05	0.27659198	ns	+
25	cg05457417	chr10:93518662	open sea		TNKS2		unknown cellspecific	-0.370	-0.019	-0.028	-0.011	-4.466	2.51E-05	0.27659198	ns	-
26	cg20822969	chr17:48290763	open sea	LOC101927230	COL1A1	TSS1500		0.669	0.016	0.009	0.024	4.463	2.54E-05	0.27659198	ns	+
27	cg12691572	chr10:114574959	open sea	VTI1A	VTI1A	Body		-1.556	0.031	0.017	0.045	4.456	2.64E-05	0.27659198	ns	+
28	cg08729810	chr21:34396944	island	OLIG2	OLIG2	TSS1500	unknown cellspecific	-2.631	-0.016	-0.023	-0.009	-4.451	2.66E-05	0.27659198	ns	-
29	cg18438770	chr4:153130797	open sea	RP11-18H21.3	FBXW7			0.957	0.019	0.011	0.028	4.441	2.76E-05	0.27659198	ns	+
30	cg13154294	chr6:52182896	open sea		MCM3		unknown cellspecific	-2.714	0.015	0.008	0.022	4.423	2.95E-05	0.27659198	ns	+
31	cg25279318	chr15:81488917	open sea	IL16	IL16	TSS1500		2.625	0.014	0.008	0.020	4.423	2.96E-05	0.27659198	ns	+
32	cg27568504	chr10:133058901	S shore	TCERG1L	TCERG1L	Body		2.979	-0.016	-0.023	-0.009	-4.401	3.21E-05	0.27659198	ns	-
33	cg02759751	chr7:1491889	island	MICALL2	MICALL2	Body	unknown cellspecific	3.586	0.014	0.008	0.021	4.391	3.33E-05	0.27659198	ns	+
34	cg25057234	chr1:214821134	open sea	CENPF	CENPF	Body		1.518	0.011	0.006	0.015	4.382	3.45E-05	0.27659198	ns	+
35	cg17790685	chrX:17708220	open sea	NHS	NHS	Body		0.514	0.015	0.008	0.022	4.382	3.45E-05	0.27659198	ns	+
36	cg17351862	chr17:1172501	N shore	BHLHA9	BHLHA9	TSS1500	unknown cellspecific	1.858	0.012	0.006	0.017	4.381	3.46E-05	0.27659198	ns	+
37	cg18101474	chrX:134478119	N shore	ZNF449	ZNF449	TSS1500	promoter-linked	-0.438	-0.017	-0.025	-0.009	-4.381	3.46E-05	0.27659198	ns	-
38	cg04207666	chr17:10648071	open sea	TMEM220	TMEM220	Body	unknown cellspecific	1.310	0.018	0.010	0.026	4.379	3.48E-05	0.27659198	ns	+
39	cg16608857	chr7:127580948	open sea	SND1	SND1	Body		1.052	0.014	0.008	0.021	4.376	3.53E-05	0.27659198	ns	+
40	cg20421623	chr1:98895512	open sea		SNX7			3.006	0.024	0.013	0.035	4.352	3.85E-05	0.2877638	ns	+
41	cg24843535	chr1:146525992	S shelf	NBPF13P	NBPF12			1.976	0.008	0.004	0.012	4.350	3.87E-05	0.2877638	ns	+
42	cg00098216	chr20:46056627	open sea		ZMYND8			0.544	0.013	0.007	0.019	4.344	3.97E-05	0.2877638	ns	+
43	cg00849282	chr11:74405496	open sea		CHRDL2		unknown cellspecific	0.761	0.008	0.004	0.012	4.339	4.05E-05	0.2877638	ns	+
44	cg20661182	chr4:140101180	S shore		ELF2			0.993	0.017	0.009	0.024	4.330	4.17E-05	0.289021323	ns	+
45	cg24794697	chr3:18278987	open sea	LOC339862	SATB1	Body		2.279	-0.012	-0.018	-0.007	-4.325	4.25E-05	0.289021323	ns	-
46	cg23758158	chr10:43602387	S shore	RET	RET	Body		2.592	0.013	0.007	0.018	4.308	4.52E-05	0.291373058	ns	+
47	cg24626660	chr12:32551988	N shore	FGD4	FGD4		unknown cellspecific	-3.685	0.021	0.011	0.031	4.302	4.64E-05	0.291373058	ns	+
48	cg17267260	chr5:12322	open sea		PLEKHG4B			1.043	-0.018	-0.026	-0.010	-4.297	4.72E-05	0.291373058	ns	-
49	cg07390210	chr18:74961196	N shore	GALR1	GALR1	TSS1500		-0.854	-0.013	-0.020	-0.007	-4.295	4.76E-05	0.291373058	ns	-
50	cg14176626	chr9:99617320	S shore	ZNF782	ZNF782	TSS1500		-2.150	0.021	0.011	0.030	4.290	4.84E-05	0.291373058	ns	+
51	cg24642789	chrX:112756151	open sea	LOC101928437	AMOT	Body		-0.018	-0.009	-0.014	-0.005	-4.286	4.91E-05	0.291373058	ns	-
52	cg02688694	chr16:75339528	open sea	CFDP1	CFDP1	Body		-2.661	-0.016	-0.024	-0.009	-4.284	4.95E-05	0.291373058	ns	-
53	cg07268431	chr3:47436106	open sea	PTPN23	PTPN23	Body	promoter-linked cellspecific	2.518	-0.017	-0.024	-0.009	-4.265	5.31E-05	0.306584546	ns	-
54	cg19041857	chr6:27730383	open sea	LOC100131289	H3C10			1.888	-0.057	-0.084	-0.031	-4.268	5.47E-05	0.309668814	ns	-
55	cg00633768	chr12:131496287	open sea	ADGRD1	ADGRD1	Body		2.160	0.017	0.009	0.025	4.251	5.57E-05	0.309944884	ns	+
56	cg13174688	chr18:8801435	open sea	MTCL1	MTCL1	Body		2.469	-0.018	-0.027	-0.010	-4.238	5.86E-05	0.317277615	ns	-
57	cg05773155	chr1:201911097	open sea	LMOD1	LMOD1	Body		2.951	-0.014	-0.020	-0.007	-4.232	5.99E-05	0.317277615	ns	-
58	cg14429204	chr1:16553327	island	ANO7L1	CPLANE2			-2.535	0.016	0.009	0.024	4.231	6.02E-05	0.317277615	ns	+
59	cg24881334	chr17:69409104	open sea		SOX9			1.852	-0.012	-0.017	-0.006	-4.209	6.50E-05	0.334208055	ns	-
60	cg09428856	chr22:17307104	open sea	HSFY1P1	XKR3	TSS1500		1.184	0.014	0.008	0.021	4.207	6.56E-05	0.334208055	ns	+
61	cg04458920	chr2:54483619	S shore	TSPYL6	TSPYL6	TSS1500		2.131	0.009	0.005	0.013	4.199	6.75E-05	0.338428096	ns	+
62	cg25763292	chr10:115442231	S shelf	CASP7	CASP7	5'UTR		1.191	0.011	0.006	0.016	4.189	7.01E-05	0.341401406	ns	+
63	cg25838959	chr3:56528527	open sea		ERC2		unknown cellspecific	2.446	0.029	0.015	0.043	4.191	7.03E-05	0.341401406	ns	+
64	cg11002763	chr11:78387503	S shore	TENM4	TENM4	Body		1.140	0.013	0.007	0.019	4.164	7.66E-05	0.352872644	ns	+
65	cg05322352	chr19:11749535	N shore	ZNF833P	ZNF627			2.558	0.012	0.006	0.017	4.162	7.70E-05	0.352872644	ns	+
66	cg18813028	chr22:39130228	open sea	GTPBP1	GTPBP1		gene-linked	1.923	0.011	0.006	0.017	4.158	7.84E-05	0.352872644	ns	+
67	cg10472919	chr5:135266805	S shore	FBXL21P	LECT2	5'UTR		-1.547	0.019	0.010	0.028	4.158	7.84E-05	0.352872644	ns	+
68	cg04004158	chr16:1152474	island		C1QTNF8			1.891	-0.019	-0.029	-0.010	-4.157	7.84E-05	0.352872644	ns	-
69	cg27301893	chr9:91584970	open sea		C9orf47			0.831	-0.009	-0.013	-0.004	-4.150	8.07E-05	0.357610088	ns	-
70	cg10799055	chr6:27725188	open sea		H2BC13		unknown cellspecific	-3.428	0.019	0.010	0.029	4.139	8.40E-05	0.366977896	ns	+
71	cg11732055	chr10:91482145	open sea	KIF20B	KIF20B	Body		2.151	0.014	0.007	0.021	4.128	8.73E-05	0.371116917	ns	+
72	cg24485995	chr6:135375451	N shore	HBS1L	HBS1L	Body		-3.140	-0.020	-0.029	-0.010	-4.127	8.75E-05	0.371116917	ns	-
73	cg23336678	chr1:100495946	open sea	SLC35A3	SLC35A3			1.661	0.010	0.005	0.014	4.124	8.86E-05	0.371116917	ns	+
74	cg11454008	chr19:39486939	open sea	FBXO27	FBXO27			0.402	0.010	0.005	0.015	4.109	9.35E-05	0.386591289	ns	+
75	cg07408779	chr11:65315205	island	LTBP3	LTBP3	Body	unknown cellspecific	0.095	0.010	0.005	0.014	4.101	9.61E-05	0.387445536	ns	+

76	cg11305179	chr4:190580204	open sea	LINC01262	FRG1	TSS1500	non-gene-linked	-0.974	-0.013	-0.019	-0.007	-4.089	0.0001003	0.387445536	ns	-
77	cg06720578	chr15:71641850	open sea	THSD4	THSD4	Body		2.513	0.013	0.007	0.020	4.086	0.000101512	0.387445536	ns	+
78	cg04874103	chr6:33872383	open sea	RP3-468B3.2	MLN			-3.329	0.024	0.012	0.036	4.078	0.000104689	0.387445536	ns	+
79	cg16345311	chr16:26505221	open sea		HS3ST4			2.273	0.015	0.008	0.022	4.076	0.000105124	0.387445536	ns	+
80	cg00866476	chr2:95740682	N shore		MRPS5		unknown cell specific	-0.711	0.012	0.006	0.017	4.075	0.000105563	0.387445536	ns	+
81	cg08438525	chr7:96816507	open sea		SDHAF3			0.067	0.027	0.014	0.040	4.075	0.000106063	0.387445536	ns	+
82	cg05061754	chr7:4254313	open sea	SDK1	SDK1	Body		2.823	-0.013	-0.019	-0.007	-4.073	0.000106315	0.387445536	ns	-
83	cg02489219	chr2:197676272	open sea	C2orf66	C2orf66	TSS1500		2.395	-0.009	-0.013	-0.004	-4.070	0.000107332	0.387445536	ns	-
84	cg18414238	chr5:45578708	open sea	HCN1	HCN1	Body		2.561	-0.011	-0.017	-0.006	-4.066	0.000108981	0.387445536	ns	-
85	cg03413202	chr10:61317733	open sea		SLC16A9			-0.867	0.021	0.011	0.031	4.065	0.000109153	0.387445536	ns	+
86	cg15377409	chr6:27730551	open sea	LOC100131289	H2BC13	Body		2.033	-0.048	-0.071	-0.024	-4.071	0.000110233	0.387445536	ns	-
87	cg02562678	chr2:25966986	open sea	ASXL2	ASXL2	Body		1.135	0.013	0.007	0.020	4.060	0.000111371	0.387445536	ns	+
88	cg16648509	chr4:86936044	open sea	MAPK10	MAPK10			0.948	0.015	0.008	0.023	4.057	0.000112561	0.387445536	ns	+
89	cg13021507	chr5:134736026	S shore	MACROH2A1	MACROH2A1	TSS1500		0.425	0.020	0.010	0.029	4.053	0.000113991	0.387445536	ns	+
90	cg13843325	chr2:233002660	open sea	DIS3L2	DIS3L2	Body		-1.519	0.013	0.007	0.019	4.052	0.000114539	0.387445536	ns	+
91	cg13501538	chr6:1616430	S shore		FOXC1			1.523	0.013	0.006	0.019	4.049	0.000115606	0.387445536	ns	+
92	cg21058963	chr6:137543824	S shelf		IFNGR1			-3.597	0.015	0.007	0.022	4.047	0.000116576	0.387445536	ns	+
93	cg02276825	chr2:130184989	open sea		RAB6C			3.081	0.017	0.008	0.025	4.044	0.000117804	0.387445536	ns	+
94	cg27203412	chr11:61244868	open sea	RP11-286N22.8	PPP1R32			0.584	-0.013	-0.019	-0.007	-4.038	0.000120287	0.390324072	ns	-
95	cg12074638	chr6:110282771	open sea		GPR6			-0.334	0.016	0.008	0.024	4.031	0.000123259	0.390324072	ns	+
96	cg07626366	chr7:40939069	open sea		SUGCT			2.122	-0.013	-0.020	-0.007	-4.030	0.00012387	0.390324072	ns	-
97	cg24716530	chr11:65321342	island	LTBP3	LTBP3	Body		0.361	0.015	0.008	0.023	4.028	0.000124811	0.390324072	ns	+
98	cg11213471	chr16:4409546	open sea	CORO7	CORO7	Body	gene-linked	1.799	0.008	0.004	0.011	4.026	0.000125576	0.390324072	ns	+
99	cg02901871	chr13:107519802	open sea		FAM155A			-2.796	0.018	0.009	0.027	4.023	0.00012716	0.390324072	ns	+
100	cg21846666	chr6:70332953	open sea		LMBRD1			-1.133	-0.020	-0.030	-0.010	-4.020	0.000128518	0.390324072	ns	-
101	cg02060919	chr4:32932085	open sea		PCDH7			2.188	-0.012	-0.018	-0.006	-4.018	0.000129427	0.390324072	ns	-
102	cg05505961	chr17:66375071	open sea	ARSG	ARSG	Body		1.647	-0.013	-0.020	-0.007	-4.014	0.000130993	0.390324072	ns	-
103	cg27541317	chr1:149871625	island	BOLA1	BOLA1	Body	promoter-linked	-1.432	0.016	0.008	0.025	4.012	0.000131989	0.390324072	ns	+
104	cg05104740	chr8:99846870	open sea	STK3	STK3	Body		2.876	-0.018	-0.026	-0.009	-4.009	0.000133191	0.390324072	ns	-
105	cg12218467	chr2:11144629	open sea		KCNF1			1.719	0.009	0.005	0.014	4.006	0.000134745	0.390324072	ns	+
106	cg06665305	chr2:110372933	island	SOWAHC	SOWAHC	1stExon		-1.801	0.016	0.008	0.024	4.003	0.000136149	0.390324072	ns	+
107	cg14132895	chr11:95433641	open sea	RP11-644L4.1	FAM76B			-0.984	0.011	0.005	0.016	4.002	0.000136545	0.390324072	ns	+
108	cg15461017	chr12:34519837	open sea		ALG10		unknown cell specific	1.438	0.012	0.006	0.018	3.998	0.000138705	0.392827653	ns	+
109	cg18872783	chr19:4594471	open sea		SEMA6B			-1.754	0.010	0.005	0.016	3.993	0.000141382	0.393835489	ns	+
110	cg21791252	chr6:33864756	open sea	LINC01016	MLN		unknown cell specific	0.643	0.014	0.007	0.020	3.992	0.000141901	0.393835489	ns	+
111	cg14016200	chr19:10399157	N shore	ICAM5	ICAM5	TSS1500		-1.376	0.012	0.006	0.018	3.989	0.000143035	0.393835489	ns	+
112	cg25896883	chr2:190272670	open sea		WDR75			1.073	0.015	0.007	0.022	3.987	0.000144211	0.393835489	ns	+
113	cg02866033	chr13:49528555	open sea		FNDC3A			2.927	-0.007	-0.011	-0.004	-3.982	0.000146847	0.397485327	ns	-
114	cg04640197	chr7:102718772	S shelf	ARMC10	ARMC10	Body		0.991	0.016	0.008	0.025	3.975	0.000150615	0.401874143	ns	+
115	cg21650737	chr8:674525	open sea	ERICH1	ERICH1	Body		0.305	-0.035	-0.052	-0.017	-3.979	0.000151096	0.401874143	ns	-
116	cg12586933	chr2:190744230	open sea	C2orf88	C2orf88			0.940	-0.016	-0.024	-0.008	-3.966	0.000155133	0.402629029	ns	-
117	cg25256099	chr17:79484960	N shore	ACTG1	ACTG1			0.844	0.016	0.008	0.024	3.964	0.000156094	0.402629029	ns	+
118	cg07313836	chr9:114898273	open sea	SUSD1	SUSD1	Body		2.370	-0.012	-0.019	-0.006	-3.963	0.000157127	0.402629029	ns	-
119	cg00690968	chr6:41437676	N shore		FOXP4		unknown cell specific	-2.241	0.022	0.011	0.033	3.960	0.000158761	0.402629029	ns	+
120	cg09565670	chr19:39368408	open sea	RINL	RINL	5'UTR		-1.101	-0.017	-0.026	-0.008	-3.959	0.000159369	0.402629029	ns	-
121	cg24312283	chr9:79628488	N shore		FOXB2			-2.271	-0.021	-0.031	-0.010	-3.950	0.000163995	0.402629029	ns	-
122	cg25573635	chr4:153859560	S shore	FHDC1	FHDC1			-0.661	0.014	0.007	0.020	3.948	0.000165425	0.402629029	ns	+
123	cg13330005	chr7:516966	island		PDGFA			1.575	-0.011	-0.017	-0.006	-3.946	0.000166299	0.402629029	ns	-
124	cg09307985	chr19:36643070	island	COX7A1	COX7A1	Body	unknown cell specific	2.616	-0.011	-0.017	-0.006	-3.945	0.00016718	0.402629029	ns	-
125	cg22540575	chr15:69353143	open sea	NOX5	NOX5	Body	unknown cell specific	2.599	0.011	0.005	0.017	3.944	0.000167546	0.402629029	ns	+
126	cg21829923	chr2:171573891	island	SP5	SP5	Body		-2.251	0.013	0.006	0.019	3.943	0.000168055	0.402629029	ns	+
127	cg17758878	chr1:231229208	open sea		FAM89A			0.028	0.045	0.022	0.068	3.950	0.000168895	0.402629029	ns	+
128	cg21442825	chr11:43968792	S shelf	RP11-613D13.4	C11orf96			-1.578	0.008	0.004	0.012	3.942	0.000168929	0.402629029	ns	+
129	cg27576692	chr22:23802502	island	AP000344.4	IGLL1			2.647	-0.031	-0.047	-0.015	-3.944	0.000169809	0.402629029	ns	-
130	cg13423262	chr6:35108939	island	TCP11	TCP11	5'UTR	unknown cell specific	-3.929	0.057	0.028	0.085	3.946	0.00017201	0.404710143	ns	+
131	cg01379173	chr18:67513211	open sea	DOK6	DOK6	3'UTR		2.976	-0.010	-0.016	-0.005	-3.924	0.000180174	0.420684002	ns	-
132	cg02308280	chr7:73985757	open sea	GTF2IRD1	GTF2IRD1	Body		1.551	0.016	0.008	0.024	3.917	0.000184311	0.424064189	ns	+
133	cg03039335	chr6:26350587	open sea		BTN3A2			2.336	0.014	0.007	0.021	3.916	0.00018467	0.424064189	ns	+
134	cg27335100	chr7:94536810	N shore	PPP1R9A	PPP1R9A	TSS200		-2.062	0.011	0.005	0.016	3.914	0.000186392	0.424064189	ns	+
135	cg01443467	chr2:232333030	S shelf	NCL	NCL			0.175	0.009	0.004	0.014	3.912	0.000187498	0.424064189	ns	+
136	cg02963206	chr19:44457959	S shelf	ZNF221	ZNF221	5'UTR		3.065	0.017	0.008	0.025	3.907	0.000190799	0.424064189	ns	+
137	cg09238401	chr16:86286477	open sea	LINC01081	FOXF1	Body	unknown cell specific	-3.340	-0.028	-0.042	-0.014	-3.908	0.000191594	0.424064189	ns	-
138	cg02129005	chr2:86412960	open sea	IMMT	IMMT	Body	promoter-linked cell specific	-0.993	0.011	0.005	0.016	3.904	0.000192832	0.424064189	ns	+
139	cg17632590	chr1:245812859	open sea	KIF26B	KIF26B	Body		-0.719	0.010	0.005	0.015	3.904	0.000192867	0.424064189	ns	+
140	cg20140735	chr1:1494726	N shelf	SSU72	SSU72	Body		1.770	-0.012	-0.018	-0.006	-3.900	0.000195773	0.424064189	ns	-
141	cg08331138	chr17:5001281	S shore		ZFP3			-2.467	0.017	0.008	0.025	3.900	0.000195866	0.424064189	ns	+
142	cg00961418	chr11:8616172	S shore	STK33	STK33	TSS1500		-2.516	0.019	0.009	0.029	3.895	0.000198755	0.424064189	ns	+
143	cg00819871	chr1:48702914	open sea	SLC5A9	SLC5A9	Body		0.992	-0.009	-0.014	-0.005	-3.895	0.000198869	0.424064189	ns	-
144	cg14125353	chr10:31892769	S shore	RP11-472N13.2	ZEB1			-1.497	0.017	0.008	0.026	3.893	0.000200248	0.424064189	ns	+
145	cg01906237	chr10:71417741	open sea		FAM241B			3.069	0.018	0.009	0.028	3.892	0.000201032	0.424064189	ns	+
146	cg05443973	chr7:33149694	S shore	RP9	RP9	TSS1500		-0.213	0.016	0.008	0.024	3.889	0.000203402	0.426123804	ns	+
147	cg24735358	chr14:59773700	open sea	DAAM1	DAAM1	Body		1.036	-0.008	-0.013	-0.004	-3.886	0.000205425	0.427435619	ns	-
148	cg10181192	chr1:56840643	open sea		PLPP3		unknown cell specific	2.688	0.009	0.004	0.014	3.873	0.000215051	0.444440617	ns	+
149	cg17087907	chr6:167104677	open sea	RPS6KA2	RPS6KA2	Body		1.642	-0.011	-0.017	-0.005	-3.870	0.000216785	0.445016795	ns	-
150	cg24386456	chr13:27536371	open sea		USP12			1.356	-0.008	-0.012	-0.004	-3.868	0.000218723	0.446002852	ns	-
151	cg22542208	chr4:6460496	open sea	PPP2R2C	PPP2R2C	Body		1.251	-0.015	-0.022	-0.007	-3.864	0.00022193	0.447117142	ns	-
152	cg02768671	chr6:169470822	open sea		THBS2			1.640	-0.011	-0.017	-0.006	-3.861	0.000223763	0.447117142	ns	-

153	cg12739724	chr17:7485930	N shore	MPDU1	MPDU1	TSS1500		-0.552	0.025	0.012	0.037	3.860	0.000226054	0.447117142	ns	+
154	cg24241410	chr7:157780706	open sea	PTPRN2	PTPRN2	Body		2.373	-0.012	-0.018	-0.006	-3.858	0.000226204	0.447117142	ns	-
155	cg07902030	chr6:20402276	island	E2F3	E2F3	5'UTR		-2.902	0.018	0.009	0.028	3.858	0.000226579	0.447117142	ns	+
156	cg27429745	chr4:99831386	open sea	EIF4E	EIF4E	Body		2.773	-0.009	-0.014	-0.004	-3.852	0.000230848	0.448222019	ns	-
157	cg03507593	chr12:124939480	N shore	NCOR2	NCOR2	Body		2.692	0.014	0.007	0.021	3.850	0.000232348	0.448222019	ns	+
158	cg13009860	chr13:113768927	S shelf	F7	F7	Body		2.461	0.011	0.005	0.016	3.850	0.000232396	0.448222019	ns	+
159	cg27485334	chr17:4336413	open sea	SPNS3	SPNS3	TSS1500		1.589	0.011	0.005	0.017	3.844	0.000237136	0.448222019	ns	+
160	cg08869869	chr1:221079754	open sea		HLX			1.819	-0.014	-0.021	-0.007	-3.844	0.000237895	0.448222019	ns	-
161	cg18926249	chr4:1635946	open sea	FAM53A	FAM53A			0.998	0.011	0.005	0.017	3.843	0.000238202	0.448222019	ns	+
162	cg07851597	chr8:28360385	open sea	FZD3	FZD3	5'UTR		-0.070	-0.015	-0.022	-0.007	-3.843	0.000238561	0.448222019	ns	-
163	cg22939000	chr2:71673632	open sea		DYSF			2.186	0.010	0.005	0.016	3.841	0.000240099	0.448222019	ns	+
164	cg17109552	chr14:81790174	open sea	STON2	STON2	Body		2.428	-0.011	-0.017	-0.005	-3.838	0.00024283	0.448222019	ns	-
165	cg11298133	chr9:112323165	open sea		PTPN3			1.940	-0.010	-0.015	-0.005	-3.833	0.000246862	0.448222019	ns	-
166	cg06319204	chr8:68069778	open sea	CSPP1	CSPP1	Body		1.900	0.009	0.004	0.014	3.832	0.000247691	0.448222019	ns	+
167	cg16082705	chr2:198175980	S shore	ANKRD44	ANKRD44	TSS1500		-2.780	-0.012	-0.018	-0.006	-3.830	0.000249207	0.448222019	ns	-
168	cg02497783	chr10:105607848	open sea	SH3PXD2A	SH3PXD2A	Body		1.929	0.013	0.006	0.020	3.829	0.000250301	0.448222019	ns	+
169	cg17951713	chr8:130698161	open sea		GSDMC			-2.904	0.013	0.006	0.020	3.827	0.000252152	0.448222019	ns	+
170	cg00732775	chr8:22546694	N shore	EGR3	EGR3	3'UTR		2.283	0.009	0.005	0.014	3.824	0.000254648	0.448222019	ns	+
171	cg09856474	chr22:22590391	open sea	IGL	IGL			-1.954	0.019	0.009	0.029	3.821	0.000256742	0.448222019	ns	+
172	cg18642369	chr13:99651231	open sea	DOCK9	DOCK9	Body	unknown cellspecific	0.581	0.012	0.006	0.018	3.821	0.000257011	0.448222019	ns	+
173	cg24913794	chr2:126716943	open sea		GYPC			-0.771	0.020	0.010	0.031	3.819	0.000259014	0.448222019	ns	+
174	cg04711118	chr17:29279190	open sea	ADAP2	ADAP2	Body		2.513	-0.010	-0.015	-0.005	-3.819	0.000259045	0.448222019	ns	-
175	cg07433054	chr14:25275498	open sea		STXBP6			-2.933	-0.036	-0.055	-0.017	-3.823	0.000260297	0.448222019	ns	-
176	cg03895221	chr1:31717101	open sea		NKAIN1			1.533	-0.009	-0.014	-0.004	-3.814	0.000263142	0.448222019	ns	-
177	cg15555985	chr3:73533854	open sea	PDZRN3	PDZRN3	Body		1.181	0.013	0.006	0.020	3.814	0.000263778	0.448222019	ns	+
178	cg13719314	chr3:45839377	S shore	SLC6A20	SLC6A20	TSS1500	unknown cellspecific	1.144	0.012	0.006	0.018	3.813	0.000264344	0.448222019	ns	+
179	cg16660547	chr10:114574152	open sea	VTI1A	VTI1A	Body		-0.011	0.027	0.013	0.041	3.814	0.000264591	0.448222019	ns	+
180	cg10229660	chr10:95581037	open sea		LGI1			0.792	0.014	0.007	0.021	3.812	0.000265586	0.448222019	ns	+
181	cg03549146	chr16:69966902	open sea	WWP2	WWP2	TSS200		-0.839	0.019	0.009	0.029	3.811	0.00026653	0.448222019	ns	+
182	cg26192769	chr15:77286576	open sea	PSTPIP1	PSTPIP1	TSS1500		-4.047	-0.033	-0.050	-0.016	-3.815	0.000266705	0.448222019	ns	-
183	cg20114103	chr6:2397671	open sea	GMDS-DT	GMDS		unknown cellspecific	2.503	-0.010	-0.016	-0.005	-3.806	0.000270537	0.452178393	ns	-
184	cg10661091	chr17:1464048	N shore	PITPNA	PITPNA	Body		1.804	-0.010	-0.015	-0.005	-3.794	0.000282469	0.468700693	ns	-
185	cg20933317	chrX:10464360	open sea	MID1	MID1	Body		0.888	0.014	0.007	0.021	3.793	0.000283487	0.468700693	ns	+
186	cg11129779	chr17:55064945	open sea	SCPEP1	SCPEP1	Body		1.303	0.025	0.012	0.038	3.789	0.000287944	0.468922248	ns	+
187	cg15627933	chr7:50871576	open sea		GRB10			1.138	0.007	0.003	0.011	3.786	0.000289873	0.468922248	ns	+
188	cg07390411	chr3:167091871	open sea	ZBBX	ZBBX	5'UTR		1.100	0.011	0.005	0.017	3.785	0.000290485	0.468922248	ns	+
189	cg20747538	chr3:137838021	S shelf		DZIP1L			-1.024	0.009	0.004	0.014	3.785	0.000291318	0.468922248	ns	+
190	cg03807298	chr5:174159039	island		MSX2			-3.200	0.021	0.010	0.032	3.782	0.000293788	0.468922248	ns	+
191	cg02434836	chr4:568205	N shore	TMEM271	TMEM271			-1.937	-0.010	-0.016	-0.005	-3.781	0.000295012	0.468922248	ns	-
192	cg19079337	chr19:41903617	S shore	EXOSC5	EXOSC5	TSS1500	promoter-linked	-2.928	-0.031	-0.047	-0.014	-3.783	0.000295997	0.468922248	ns	-
193	cg17979628	chr1:212606106	island	NENF	NENF	TSS200	promoter-linked	-2.929	0.017	0.008	0.026	3.778	0.000297414	0.468922248	ns	+
194	cg11410920	chr20:43997025	open sea	SYS1	SYS1	3'UTR	unknown cellspecific	-0.098	0.010	0.005	0.016	3.778	0.000297419	0.468922248	ns	+
195	cg22854836	chr7:100844414	N shore	MOGAT3	MOGAT3	TSS200		-2.137	-0.014	-0.021	-0.007	-3.777	0.000299167	0.469259014	ns	-
196	cg03040821	chr1:212966209	S shore	TATDN3	TATDN3	Body		1.220	0.009	0.004	0.014	3.774	0.000302226	0.469779934	ns	+
197	cg17840774	chr1:236109168	open sea		NID1			1.405	0.013	0.006	0.020	3.773	0.000303242	0.469779934	ns	+
198	cg19533760	chr15:56299636	open sea	CNOT6LP1	NEDD4			-0.781	-0.038	-0.058	-0.018	-3.778	0.000304106	0.469779934	ns	-
199	cg12949461	chr6:33872364	open sea	RP3-468B3.2	MLN			-3.918	0.018	0.009	0.028	3.769	0.000307679	0.470919006	ns	+
200	cg06809344	chr15:26962955	open sea	GABRB3	GABRB3	TSS1500		0.926	-0.016	-0.024	-0.007	-3.768	0.000308699	0.470919006	ns	-
201	cg24817873	chr18:55471563	S shore	ATP8B1	ATP8B1	TSS1500		3.143	0.019	0.009	0.030	3.767	0.000309463	0.470919006	ns	+
202	cg01171067	chrX:152077467	open sea		ZNF185			2.729	0.018	0.008	0.027	3.763	0.000313825	0.473369836	ns	+
203	cg22740144	chr17:56021390	open sea	CUEDC1	CUEDC1	5'UTR		2.764	0.009	0.004	0.015	3.762	0.000314229	0.473369836	ns	+
204	cg25584814	chr19:345306	island	MIER2	MIER2	TSS1500	promoter-linked	3.508	0.021	0.010	0.032	3.761	0.000315716	0.473369836	ns	+
205	cg11585500	chr5:34657986	S shore	RAI14	RAI14	5'UTR	unknown cellspecific	2.116	0.012	0.005	0.018	3.754	0.000322968	0.481881167	ns	+
206	cg25621286	chr5:195609	S shore	LRRC14B	LRRC14B		unknown cellspecific	2.572	0.010	0.005	0.015	3.748	0.000330173	0.486520193	ns	+
207	cg22612033	chr10:75568895	N shelf	NDST2	NDST2	5'UTR		2.082	0.010	0.005	0.016	3.747	0.000331005	0.486520193	ns	+
208	cg25923606	chr3:184099881	S shore	CHRD	CHRD	5'UTR		-0.673	-0.006	-0.010	-0.003	-3.747	0.000331203	0.486520193	ns	-
209	cg00523861	chrX:137827166	open sea	FGF13	FGF13	Body		2.150	0.008	0.004	0.013	3.746	0.000332574	0.486520193	ns	+
210	cg01334432	chr1:209604583	open sea	MIR205HG	CAMK1G	Body		2.348	-0.010	-0.015	-0.005	-3.744	0.000334705	0.486520193	ns	-
211	cg04585717	chr15:79350759	open sea	RASGRF1	RASGRF1	Body		2.070	0.012	0.006	0.019	3.743	0.000335644	0.486520193	ns	+
212	cg01546378	chr2:38892901	open sea	GALM	GALM	TSS200	promoter-linked cellspecific	-2.683	0.020	0.009	0.030	3.741	0.000338188	0.486520193	ns	+
213	cg17201805	chr16:4711507	open sea	MGRN1	MGRN1	Body		1.587	0.012	0.006	0.018	3.740	0.000338802	0.486520193	ns	+
214	cg00158530	chr16:69966973	open sea	WWP2	WWP2	TSS200		-0.199	0.019	0.009	0.029	3.738	0.000341768	0.487502965	ns	+
215	cg16796107	chrX:71363217	open sea	NHSL2	NHSL2	Body		0.782	0.009	0.004	0.014	3.737	0.000342674	0.487502965	ns	+
216	cg01073976	chrX:73406663	open sea	FTX	ZCCHC13	Body		2.570	0.015	0.007	0.024	3.734	0.000345581	0.489361215	ns	+
217	cg05328667	chr15:28015357	island	OCA2	OCA2	Body		2.692	-0.020	-0.030	-0.009	-3.731	0.000350052	0.49340848	ns	-
218	cg24073777	chr1:10832698	open sea	CASZ1	CASZ1	5'UTR		1.657	0.013	0.006	0.020	3.729	0.000352414	0.494459866	ns	+
219	cg14883291	chr11:86383761	S shore	ME3	ME3	TSS1500		-1.555	0.018	0.008	0.028	3.725	0.000357164	0.496849466	ns	+
220	cg06597503	chr4:19311002	open sea	RP11-3J1.1	SLIT2			2.088	0.009	0.004	0.014	3.724	0.000357635	0.496849466	ns	+
221	cg17865872	chr12:32551910	N shore	FGD4	FGD4			-0.042	0.019	0.009	0.029	3.722	0.000359896	0.496849466	ns	+
222	cg14582523	chr6:169952299	open sea	WDR27	WDR27	Body		2.534	-0.013	-0.020	-0.006	-3.720	0.000362316	0.496849466	ns	-
223	cg20622612	chr20:51463896	open sea		TSHZ2			-2.628	-0.024	-0.037	-0.011	-3.718	0.000366847	0.496849466	ns	-
224	cg23713156	chr17:80606235	island	WDR45B	WDR45B	5'UTR	promoter-linked	-0.752	0.007	0.003	0.011	3.716	0.000367818	0.496849466	ns	+
225	cg20587196	chr4:920607	open sea	GAK	GAK	Body	gene-linked	2.228	-0.011	-0.016	-0.005	-3.715	0.000369223	0.496849466	ns	-
226	cg10987788	chr11:34535377	open sea	ELF5	ELF5	TSS200		2.710	0.011	0.005	0.017	3.714	0.000369858	0.496849466	ns	+
227	cg08551870	chr22:30968831	open sea	GAL3ST1	GAL3ST1			0.645	-0.007	-0.011	-0.003	-3.714	0.000370301	0.496849466	ns	-
228	cg13518672	chr6:34787640	open sea	UHRF1BP1	UHRF1BP1	Body		-0.714	0.011	0.005	0.017	3.714	0.000370361	0.496849466	ns	+
229	cg11952493	chr10:28570835	open sea	MPP7	MPP7	5'UTR		0.726	0.012	0.006	0.019	3.711	0.000374674	0.499134525	ns	+

230	cg21896142	chrX:80457315	open sea	HMGN5	HMGN5	1stExon	promoter-linked	-0.804	0.015	0.007	0.022	3.710	0.000375328	0.499134525	ns	+
231	cg25447202	chr4:3409885	open sea	RGS12	RGS12	Body		1.939	-0.013	-0.019	-0.006	-3.704	0.00038311	0.50545106	ns	-
232	cg00031362	chr7:30384280	open sea	ZNRF2	ZNRF2	Body		0.114	0.012	0.006	0.019	3.702	0.000385152	0.50545106	ns	+
233	cg26809868	chr16:2886233	open sea	ZG16B	ZG16B			-3.584	0.020	0.009	0.031	3.702	0.000386436	0.50545106	ns	+
234	cg25661792	chr3:194014530	island	LINC00887	CPN2			1.450	0.014	0.006	0.021	3.701	0.000386688	0.50545106	ns	+
235	cg18659248	chr10:3679062	open sea	LOC105376360	KLF6			2.568	-0.015	-0.022	-0.007	-3.697	0.000392741	0.50571406	ns	-
236	cg09974253	chr18:8800513	open sea	MTCL1	MTCL1	Body		2.295	-0.013	-0.020	-0.006	-3.695	0.000394787	0.50571406	ns	-
237	cg19881557	chr14:20967426	open sea		RNASE10			-0.381	0.013	0.006	0.021	3.694	0.000395559	0.50571406	ns	+
238	cg02475236	chr21:42217001	N shore	DSCAM	DSCAM	Body		2.495	-0.010	-0.015	-0.004	-3.694	0.000395821	0.50571406	ns	-
239	cg21646955	chr6:35108921	island	TCP11	TCP11	Body	unknown cellspecific	-3.249	0.046	0.021	0.070	3.701	0.000396434	0.50571406	ns	+
240	cg21043573	chr1:52135013	open sea	OSBPL9	OSBPL9	Body		0.597	0.014	0.006	0.021	3.693	0.000397797	0.50571406	ns	+
241	cg04883131	chrX:9734059	island	GPR143	GPR143	TSS200		-0.207	0.018	0.008	0.028	3.691	0.000400826	0.50571406	ns	+
242	cg22375663	chr12:69725435	open sea	RP11-1143G9.5	LYZ			-2.850	0.016	0.007	0.025	3.689	0.000402302	0.50571406	ns	+
243	cg13411999	chr1:32166930	N shelf	COL16A1	COL16A1	Body		-0.519	-0.012	-0.019	-0.006	-3.688	0.000404348	0.50571406	ns	-
244	cg10372399	chr1:247522735	open sea		ZNF496			2.471	0.009	0.004	0.014	3.686	0.000406671	0.50571406	ns	+
245	cg26110907	chr19:54175137	open sea	MIR1323	DPRX	TSS200		-1.366	0.009	0.004	0.014	3.686	0.000407595	0.50571406	ns	+
246	cg14572689	chr10:95276766	open sea	CEP55	CEP55	Body		2.548	0.013	0.006	0.020	3.685	0.000408124	0.50571406	ns	+
247	cg20408337	chr22:43167595	S shore		ARFGAP3			1.954	0.009	0.004	0.014	3.683	0.000411679	0.50571406	ns	+
248	cg10072237	chr6:33872861	open sea	RP3-468B3.2	MLN			0.081	0.033	0.015	0.051	3.687	0.000411751	0.50571406	ns	+
249	cg02571944	chr2:37416022	open sea	SULT6B1	SULT6B1	TSS1500		2.224	0.008	0.004	0.013	3.682	0.000413136	0.50571406	ns	+
250	cg06621027	chr2:80179759	open sea	CTNNA2	CTNNA2	Body		0.263	0.021	0.010	0.032	3.681	0.000414892	0.50571406	ns	+
251	cg19366877	chr1:204010639	open sea	LINC00303	SOX13	TSS1500		1.059	-0.011	-0.017	-0.005	-3.680	0.000414997	0.50571406	ns	-
252	cg19246089	chr18:47901096	N shore	SKA1	SKA1	TSS1500	unknown cellspecific	-0.854	0.016	0.008	0.025	3.679	0.000416754	0.505840495	ns	+
253	cg18761976	chr15:91807630	open sea	SV2B	SV2B	Body		0.406	0.015	0.007	0.023	3.678	0.000418669	0.506155734	ns	+
254	cg24512794	chr2:203640233	S shore	ICA1L	ICA1L	3'UTR		2.171	0.009	0.004	0.013	3.674	0.000423303	0.508803451	ns	+
255	cg06282247	chr2:151281841	open sea		RND3			-1.010	-0.009	-0.014	-0.004	-3.674	0.000424186	0.508803451	ns	-
256	cg26175789	chr2:75821892	open sea		EVA1A			2.243	0.018	0.008	0.028	3.668	0.000432207	0.516399395	ns	+
257	cg24795933	chr15:96632333	open sea	LOC105369212	NR2F2			1.939	0.014	0.006	0.022	3.665	0.00043658	0.519594523	ns	+
258	cg02082273	chr11:10473038	S shore	AMPD3	AMPD3	Body	promoter-linked cellspecific	-0.143	-0.009	-0.014	-0.004	-3.664	0.000438923	0.520358757	ns	-
259	cg26933021	chr1:12618574	open sea		DHRS3			1.699	0.010	0.004	0.015	3.661	0.000443189	0.523299175	ns	+
260	cg22774691	chr1:100515842	open sea	MFSD14A	MFSD14A	Body		-2.327	-0.011	-0.018	-0.005	-3.659	0.000446249	0.523299175	ns	-
261	cg14376987	chr10:91553565	open sea		KIF20B		unknown cellspecific	-2.028	-0.008	-0.013	-0.004	-3.658	0.000446536	0.523299175	ns	-
262	cg07905442	chr8:37748443	open sea	RAB11FIP1	RAB11FIP1	Body		0.290	0.008	0.003	0.012	3.655	0.000451577	0.524196464	ns	+
263	cg11363022	chr17:47963758	open sea	RP11-304F15.4	TAC4		unknown cellspecific	2.452	0.012	0.005	0.018	3.655	0.000452351	0.524196464	ns	+
264	cg08800242	chr12:116192997	open sea	RP11-110L15.1	MED13L			3.004	0.012	0.005	0.018	3.655	0.000452443	0.524196464	ns	+
265	cg05287206	chr19:17377977	open sea	BABAM1	BABAM1	TSS1500	promoter-linked	-2.373	-0.015	-0.023	-0.007	-3.653	0.000454599	0.524706341	ns	-
266	cg00680277	chr2:3465103	S shelf	TRAPPC12	TRAPPC12	Body		2.263	-0.009	-0.014	-0.004	-3.650	0.000459406	0.528261201	ns	-
267	cg22997856	chr9:14199114	open sea	NFIB	NFIB	Body		2.392	0.013	0.006	0.020	3.644	0.000469308	0.535661706	ns	+
268	cg11049325	chr8:77492619	open sea	RP11-115I9.1	ZFHX4			1.741	0.011	0.005	0.017	3.644	0.000469344	0.535661706	ns	+
269	cg00627447	chr16:83233690	open sea	CDH13	CDH13	5'UTR		1.007	-0.014	-0.022	-0.006	-3.640	0.000474754	0.539565605	ns	-
270	cg11611536	chr17:74907612	open sea	MGAT5B	MGAT5B	Body		0.666	0.009	0.004	0.015	3.639	0.000476293	0.539565605	ns	+
271	cg00349061	chr1:26371865	N shore	SLC30A2	SLC30A2	Body	unknown cellspecific	0.931	0.019	0.009	0.029	3.637	0.000478943	0.540565692	ns	+
272	cg17330097	chr1:206316676	open sea	CTSE	CTSE	TSS1500		0.845	0.010	0.005	0.016	3.635	0.00048321	0.541616689	ns	+
273	cg01194279	chr12:114073142	open sea		LHX5			1.425	0.013	0.006	0.020	3.634	0.000484958	0.541616689	ns	+
274	cg12087004	chr3:195906436	open sea		ZDHHC19		promoter-linked	-4.239	-0.022	-0.033	-0.010	-3.633	0.0004862	0.541616689	ns	-
275	cg18394845	chr5:172731192	open sea		STC2			2.407	0.014	0.006	0.021	3.630	0.000491639	0.541616689	ns	+
276	cg21240528	chr17:3695070	open sea	ITGAE	ITGAE	Body		-1.008	-0.014	-0.022	-0.006	-3.629	0.000492057	0.541616689	ns	-
277	cg03651951	chr1:182041216	open sea		ZNF648			0.350	0.015	0.007	0.024	3.629	0.000492642	0.541616689	ns	+
278	cg24390713	chr3:152456507	open sea		P2RY1			-0.051	-0.015	-0.023	-0.007	-3.628	0.000493953	0.541616689	ns	-
279	cg26839028	chr6:88457622	open sea		AKIRIN2			-1.537	0.008	0.004	0.012	3.628	0.00049404	0.541616689	ns	+
280	cg21836919	chr1:207351463	open sea	C4BPAP1	C4BPA			-1.133	0.016	0.007	0.025	3.627	0.000495885	0.541697633	ns	+
281	cg13172130	chr3:14241168	open sea	LSM3	LSM3			2.367	-0.011	-0.017	-0.005	-3.626	0.000497764	0.541814711	ns	-
282	cg08358392	chr6:130339410	N shore	L3MBTL3	L3MBTL3	TSS1500		0.968	0.011	0.005	0.018	3.621	0.00050557	0.543195057	ns	+
283	cg12342334	chr10:99531038	island	SFRP5	SFRP5	Body	unknown cellspecific	-2.993	0.022	0.010	0.034	3.622	0.000505651	0.543195057	ns	+
284	cg22178367	chr15:81156076	open sea	CEMIP	CEMIP	5'UTR		0.460	0.009	0.004	0.014	3.621	0.000505972	0.543195057	ns	+
285	cg05338320	chr1:1510984	S shore	SSU72	SSU72	TSS1500		-3.780	-0.019	-0.030	-0.009	-3.621	0.000506653	0.543195057	ns	-
286	cg26070865	chr5:959141	island		TRIP13			4.037	-0.018	-0.029	-0.008	-3.617	0.000513548	0.543195057	ns	-
287	cg22290782	chr17:48608794	open sea	MYCBPAP	MYCBPAP	3'UTR		1.919	0.011	0.005	0.017	3.615	0.000515902	0.543195057	ns	+
288	cg23103009	chr10:134564232	S shore	INPP5A	INPP5A	Body		2.661	-0.011	-0.017	-0.005	-3.615	0.00051593	0.543195057	ns	-
289	cg18880088	chr6:88654493	open sea		SPACA1			0.877	-0.010	-0.015	-0.004	-3.612	0.000520553	0.543195057	ns	-
290	cg24752487	chr2:38324188	open sea	CYP1B1	CYP1B1			1.825	0.032	0.014	0.050	3.616	0.000521347	0.543195057	ns	+
291	cg04961265	chr3:37284183	N shore	GOLGA4	GOLGA4	TSS1500	promoter-linked	-1.337	0.013	0.006	0.019	3.611	0.000522275	0.543195057	ns	+
292	cg04567952	chr2:200775554	N shore	C2orf69	C2orf69	TSS1500	promoter-linked	-3.076	0.045	0.020	0.070	3.617	0.000523223	0.543195057	ns	+
293	cg11438560	chr5:148521623	S shore	ABLIM3	ABLIM3	5'UTR		-1.493	-0.013	-0.021	-0.006	-3.611	0.000523331	0.543195057	ns	-
294	cg18087143	chr7:38209440	open sea		STARD3NL			2.094	0.013	0.006	0.020	3.611	0.000523633	0.543195057	ns	+
295	cg02740572	chr6:53214303	S shore	ELOVL5	ELOVL5	TSS1500		-3.585	-0.018	-0.027	-0.008	-3.611	0.000523894	0.543195057	ns	-
296	cg04262428	chr11:70673256	S shore	SHANK2	SHANK2	Body		-2.693	-0.031	-0.048	-0.014	-3.613	0.000526819	0.543701435	ns	-
297	cg01823699	chr9:28236136	open sea	LINGO2	LINGO2	5'UTR		1.514	0.009	0.004	0.014	3.608	0.000528929	0.543701435	ns	+
298	cg17199113	chr17:32486621	S shelf	ASIC2	ASIC2			1.252	-0.008	-0.013	-0.004	-3.607	0.000529716	0.543701435	ns	-
299	cg19556758	chr9:37504792	open sea		POLR1E			3.295	0.017	0.008	0.027	3.600	0.000542552	0.549712296	ns	+
300	cg25349900	chr10:102290114	S shore	NDUFB8	NDUFB8	TSS1500		1.319	0.010	0.004	0.015	3.599	0.000545176	0.549712296	ns	+
301	cg07341300	chr17:73259496	S shore	MRPS7	MRPS7	Body		2.382	0.009	0.004	0.014	3.597	0.00054823	0.549712296	ns	+
302	cg22974467	chr11:77168514	open sea	PAK1	PAK1	5'UTR		0.994	0.016	0.007	0.025	3.597	0.000548432	0.549712296	ns	+
303	cg12500690	chr4:140221944	N shore	NDUFC1	NDUFC1	5'UTR		-2.664	-0.012	-0.018	-0.005	-3.597	0.000548682	0.549712296	ns	-
304	cg25271063	chr1:159771375	open sea	FCRL6	FCRL6	TSS1500		2.075	0.012	0.005	0.018	3.597	0.000548862	0.549712296	ns	+
305	cg27331989	chr17:79216949	S shelf		NDUFAF8			1.643	-0.009	-0.014	-0.004	-3.596	0.000549816	0.549712296	ns	-

PHQ-9: DEPRESSION

rank	cpg	cpg.position	region	annotation	gene	gene.group	gene.feature	AveExpr	logFC	CI.L	CI.R	t	P.Value	adj.P.Val	fdr	sign
1	cg05360958	chr12:15038440	open sea	MGP	MGP	Body		-0.835	-0.033	-0.047	-0.019	-4.800	7.02E-06	0.477025935	ns	-
2	cg17351862	chr17:1172501	N shore	BHLHA9	BHLHA9	TSS1500	unknown cellspecific	1.858	0.022	0.013	0.031	4.744	8.71E-06	0.477025935	ns	+
3	cg02479022	chr8:22250005	open sea	SLC39A14	SLC39A14	5'UTR		2.612	-0.017	-0.025	-0.010	-4.717	9.67E-06	0.477025935	ns	-
4	cg12018751	chr8:93112853	N shore	RUNX1T1	RUNX1T1	TSS1500		0.094	0.023	0.013	0.032	4.711	9.90E-06	0.477025935	ns	+
5	cg19776282	chr8:683148	S shore	ERICH1	ERICH1		unknown cellspecific	2.017	0.027	0.016	0.039	4.691	1.07E-05	0.477025935	ns	+
6	cg18101474	chrX:134478119	N shore	ZNF449	ZNF449	TSS1500	promoter-linked	-0.438	-0.027	-0.038	-0.016	-4.680	1.12E-05	0.477025935	ns	-
7	cg08984179	chr10:119117730	open sea	PDZD8	PDZD8	Body		0.637	0.028	0.016	0.040	4.631	1.34E-05	0.477025935	ns	+
8	cg01815259	chr2:56317578	open sea	CCDC85A	CCDC85A			-2.491	-0.027	-0.039	-0.016	-4.606	1.48E-05	0.477025935	ns	-
9	cg00885957	chr13:20160888	open sea	LINC00350	TPTE2	TSS1500		1.249	0.018	0.010	0.026	4.565	1.73E-05	0.477025935	ns	+
10	cg10680490	chr17:707018	N shore	NXN	NXN	Body		3.321	-0.031	-0.045	-0.018	-4.560	1.76E-05	0.477025935	ns	-
11	cg24276541	chr11:59806469	open sea	OOSP2	OOSP2	TSS1500		-1.243	0.020	0.011	0.029	4.545	1.87E-05	0.477025935	ns	+
12	cg26348226	chr1:21617442	S shore	ECE1	ECE1	TSS1500		-2.816	-0.026	-0.038	-0.015	-4.517	2.07E-05	0.477025935	ns	-
13	cg24277817	chr19:13135662	island	NFIX	NFIX	Body		-1.152	-0.036	-0.052	-0.020	-4.475	2.43E-05	0.477025935	ns	-
14	cg24626660	chr12:32551988	N shore	FGD4	FGD4		unknown cellspecific	-3.685	0.033	0.018	0.048	4.462	2.55E-05	0.477025935	ns	+
15	cg18087143	chr7:38209440	open sea		STARD3NL			2.094	0.021	0.012	0.031	4.454	2.63E-05	0.477025935	ns	+
16	cg18458017	chr10:60421397	open sea	BICC1	BICC1	Body		2.784	0.025	0.014	0.037	4.396	3.27E-05	0.477025935	ns	+
17	cg06654369	chr7:158793845	S shelf		DYNC2I1			1.374	-0.027	-0.040	-0.015	-4.377	3.50E-05	0.477025935	ns	-
18	cg02157117	chr18:28286221	open sea		DSC3			2.187	-0.019	-0.028	-0.010	-4.370	3.60E-05	0.477025935	ns	-
19	cg03794433	chr15:90548061	S shelf	ZNF710	ZNF710	5'UTR	unknown cellspecific	-2.785	-0.022	-0.032	-0.012	-4.361	3.72E-05	0.477025935	ns	-
20	cg14788049	chr11:64018549	island	PLCB3	PLCB3	TSS1500	promoter-linked	-2.116	-0.021	-0.030	-0.011	-4.360	3.74E-05	0.477025935	ns	-
21	cg02255004	chr4:80748660	open sea	PCAT4	ANTXR2	Body		0.948	0.018	0.010	0.026	4.360	3.74E-05	0.477025935	ns	+
22	cg08413370	chr3:8973066	open sea	RAD18	RAD18	Body		1.011	0.029	0.016	0.043	4.357	3.77E-05	0.477025935	ns	+
23	cg12387154	chr6:32119639	S shore	PRRT1	PRRT1	1stExon		-2.615	-0.026	-0.038	-0.014	-4.340	4.02E-05	0.477025935	ns	-
24	cg01938245	chr16:23071151	open sea		USP31			0.930	0.021	0.012	0.031	4.327	4.22E-05	0.477025935	ns	+
25	cg10923466	chr2:74619442	S shore	DCTN1	DCTN1	Body	promoter-linked	-3.073	-0.027	-0.039	-0.014	-4.323	4.28E-05	0.477025935	ns	-
26	cg01461733	chr16:3018302	island	PAQR4	PAQR4	TSS1500		1.491	0.027	0.015	0.040	4.318	4.36E-05	0.477025935	ns	+
27	cg09428856	chr22:17307104	open sea	HSFY1P1	XKR3	TSS1500		1.184	0.029	0.015	0.042	4.300	4.66E-05	0.477025935	ns	+
28	cg06062998	chr5:154164389	open sea	LARP1	LARP1	Body	promoter-linked cellspecific	1.082	0.022	0.012	0.032	4.282	4.97E-05	0.477025935	ns	+
29	cg24642789	chrX:112756151	open sea	LOC101928437	AMOT	Body		-0.018	-0.015	-0.022	-0.008	-4.270	5.21E-05	0.477025935	ns	-
30	cg04874103	chr6:33872383	open sea	RP3-468B3.2	MLN			-3.329	0.037	0.020	0.054	4.270	5.22E-05	0.477025935	ns	+
31	cg07434260	chr19:10698036	island	AP1M2	AP1M2	TSS200		-2.101	-0.025	-0.037	-0.013	-4.265	5.30E-05	0.477025935	ns	-
32	cg09253736	chr8:124184214	open sea		TBC1D31			1.801	0.022	0.012	0.032	4.258	5.44E-05	0.477025935	ns	+
33	cg03240727	chr9:37589495	N shelf	TOMM5	TOMM5	3'UTR		1.159	0.023	0.012	0.035	4.244	5.72E-05	0.477025935	ns	+
34	cg04711118	chr17:29279190	open sea	ADAP2	ADAP2	Body		2.513	-0.018	-0.026	-0.009	-4.226	6.11E-05	0.477025935	ns	-
35	cg14350358	chr17:48913317	open sea	WFIKKN2	WFIKKN2	1stExon		1.602	0.020	0.010	0.029	4.212	6.43E-05	0.477025935	ns	+
36	cg08209934	chr16:69966975	open sea	WWP2	WWP2	TSS200		0.316	0.031	0.016	0.045	4.202	6.67E-05	0.477025935	ns	+
37	cg16175713	chr6:46617172	N shelf	CYP39A1	CYP39A1	Body		-3.072	-0.031	-0.045	-0.016	-4.200	6.73E-05	0.477025935	ns	-
38	cg14851284	chr11:70713732	open sea	SHANK2	SHANK2	Body		1.730	0.018	0.009	0.027	4.196	6.83E-05	0.477025935	ns	+
39	cg11978884	chr5:135415819	N shore		TGFBI		unknown cellspecific	-1.243	0.034	0.018	0.051	4.187	7.06E-05	0.477025935	ns	+
40	cg02192673	chr4:72897565	island	NPFFR2	NPFFR2	1stExon		0.393	-0.023	-0.034	-0.012	-4.186	7.07E-05	0.477025935	ns	-
41	cg07293736	chr12:132670858	N shelf		GALNT9		unknown cellspecific	-1.118	-0.027	-0.039	-0.014	-4.184	7.13E-05	0.477025935	ns	-
42	cg13456086	chr8:110704757	S shore	SYBU	SYBU	TSS1500		1.493	-0.024	-0.035	-0.012	-4.171	7.47E-05	0.477025935	ns	-
43	cg06634140	chr14:95956325	open sea		SYNE3			-0.697	0.025	0.013	0.038	4.170	7.50E-05	0.477025935	ns	+
44	cg02386604	chr8:110656245	N shore	SYBU	SYBU	Body		0.137	-0.016	-0.024	-0.009	-4.169	7.51E-05	0.477025935	ns	-
45	cg14545159	chr17:41479473	S shore		ARL4D			2.932	0.022	0.011	0.032	4.167	7.57E-05	0.477025935	ns	+
46	cg08346575	chr19:45779837	open sea	MARK4	MARK4	Body		-0.060	-0.018	-0.027	-0.010	-4.167	7.59E-05	0.477025935	ns	-
47	cg16598016	chr4:176708523	open sea	GPM6A	GPM6A	1stExon		2.024	-0.021	-0.031	-0.011	-4.162	7.71E-05	0.477025935	ns	-
48	cg05901357	chr2:27529325	island	TRIM54	TRIM54	Body	unknown cellspecific	-1.228	-0.027	-0.040	-0.014	-4.161	7.74E-05	0.477025935	ns	-
49	cg21699506	chr4:80786707	open sea	PCAT4	ANTXR2			0.915	-0.017	-0.025	-0.009	-4.160	7.76E-05	0.477025935	ns	-
50	cg02493602	chr11:86383696	S shore	ME3	ME3	TSS1500		-1.761	0.049	0.026	0.073	4.162	7.80E-05	0.477025935	ns	+
51	cg04515200	chr5:135415762	N shore		TGFBI		unknown cellspecific	-1.099	0.042	0.022	0.062	4.140	8.36E-05	0.501412817	ns	+
52	cg18061711	chr5:71683955	open sea		PTCD2			1.897	0.021	0.011	0.031	4.112	9.24E-05	0.535405748	ns	+
53	cg00158530	chr16:69966973	open sea	WWP2	WWP2	TSS200		-0.199	0.036	0.018	0.053	4.111	9.28E-05	0.535405748	ns	+
54	cg12714719	chr6:43135100	N shelf		SFR			1.346	0.019	0.010	0.028	4.098	9.73E-05	0.539959927	ns	+
55	cg00337945	chr7:26132233	open sea		NFE2L3			3.318	0.016	0.008	0.023	4.096	9.78E-05	0.539959927	ns	+
56	cg24561270	chr5:170026067	open sea	KCNIP1	KCNIP1	5'UTR		-2.550	-0.022	-0.033	-0.011	-4.093	9.89E-05	0.539959927	ns	-
57	cg07306263	chr21:19165163	open sea	C21orf91	C21orf91	3'UTR		1.020	0.023	0.012	0.035	4.070	0.000107473	0.561013987	ns	+
58	cg27136869	chr6:56534923	open sea	DST	DST	Body		0.378	0.021	0.011	0.031	4.069	0.0001079	0.561013987	ns	+
59	cg11835197	chr1:36038514	N shore	TFAP2E	TFAP2E	TSS1500	unknown cellspecific	1.065	-0.015	-0.023	-0.008	-4.068	0.000108216	0.561013987	ns	-
60	cg01952537	chr14:105051261	N shelf	C14orf180	C14orf180	5'UTR	unknown cellspecific	0.963	0.016	0.008	0.024	4.061	0.0001109	0.565347408	ns	+
61	cg25431946	chr4:40313893	open sea	LOC101060498	CHRNA9	Body		-3.017	-0.046	-0.069	-0.024	-4.052	0.000115168	0.569894256	ns	-
62	cg03880977	chr3:122609490	open sea	LOC100129550	SLC49A4	Body	unknown cellspecific	-3.126	0.027	0.014	0.040	4.037	0.000120657	0.569894256	ns	+
63	cg10700647	chr1:28832638	open sea	RCC1	RCC1	Body	promoter-linked	-2.777	-0.029	-0.043	-0.015	-4.031	0.000123248	0.569894256	ns	-
64	cg04585717	chr15:79350759	open sea	RASGRF1	RASGRF1	Body		2.070	0.022	0.011	0.033	4.030	0.000123723	0.569894256	ns	+
65	cg08662052	chr19:44764603	S shore	ZNF233	ZNF233	5'UTR		-0.708	-0.032	-0.048	-0.016	-4.024	0.00012649	0.569894256	ns	-
66	cg06739303	chr10:42863444	S shore	LOC441666	CCNYL2	Body		-1.259	0.050	0.025	0.075	4.027	0.000126762	0.569894256	ns	+
67	cg18855674	chr8:72469553	island	RP11-1102P16.1	EYA1			-3.528	-0.023	-0.034	-0.011	-4.012	0.000132003	0.569894256	ns	-
68	cg15607708	chr19:54041308	island	ZNF331	ZNF331	TSS200	promoter-linked	-1.037	0.020	0.010	0.030	4.009	0.00013358	0.569894256	ns	+
69	cg14505078	chrX:52840083	open sea	XAGE5	XAGE5	TSS1500		0.883	0.020	0.010	0.029	4.007	0.000134502	0.569894256	ns	+
70	cg00736299	chr16:4730465	island	MGRN1	MGRN1	Body		-1.533	-0.033	-0.049	-0.017	-4.004	0.000135755	0.569894256	ns	-
71	cg24118715	chr10:70232072	S shore	DNA2	DNA2	TSS200		-1.391	-0.018	-0.027	-0.009	-3.992	0.000141709	0.569894256	ns	-
72	cg01989611	chr22:31221853	S shelf	OSBP2	OSBP2	Body		3.068	-0.023	-0.034	-0.012	-3.990	0.000142552	0.569894256	ns	-
73	cg06998981	chr2:212602163	open sea	ERBB4	ERBB4	Body		0.020	0.024	0.012	0.036	3.990	0.000142608	0.569894256	ns	+
74	cg19397294	chr4:166826744	open sea	TLL1	TLL1	Body		1.285	-0.017	-0.026	-0.009	-3.990	0.000142844	0.569894256	ns	-
75	cg19787251	chr13:98227620	open sea		RAP2A			2.714	0.016	0.008	0.024	3.985	0.000145115	0.569894256	ns	+

76	cg01339752	chr11:73433318	open sea	RAB6A	RAB6A	Body		1.327	0.024	0.012	0.036	3.982	0.000146571	0.569894256	ns	+
77	cg25609434	chr3:194078242	open sea	LRRC15	LRRC15	3'UTR		2.352	-0.021	-0.031	-0.010	-3.981	0.00014735	0.569894256	ns	-
78	cg23996381	chr17:70030378	open sea	LINC01152	SOX9	Body		2.578	-0.022	-0.033	-0.011	-3.980	0.000147907	0.569894256	ns	-
79	cg00937982	chr21:38080274	island	SIM2	SIM2	Body		-1.624	0.018	0.009	0.027	3.963	0.000157021	0.569894256	ns	+
80	cg08094924	chr14:89970089	open sea	FOXN3	FOXN3	5'UTR		2.092	-0.021	-0.032	-0.011	-3.954	0.00016212	0.569894256	ns	-
81	cg13642872	chr15:78527113	open sea	ACSBG1	ACSBG1	TSS1500		2.106	0.020	0.010	0.030	3.953	0.00016272	0.569894256	ns	+
82	cg06157313	chr10:98788828	open sea	SLIT1	SLIT1	Body		2.832	0.021	0.010	0.031	3.952	0.000163308	0.569894256	ns	+
83	cg04084052	chr1:165668349	S shore	ALDH9A1	ALDH9A1	TSS1500		1.310	0.021	0.010	0.031	3.949	0.000164606	0.569894256	ns	+
84	cg17330388	chr8:58171766	N shore		BPNT2			2.556	0.021	0.011	0.032	3.948	0.000165363	0.569894256	ns	+
85	cg19079337	chr19:41903617	S shore	EXOSC5	EXOSC5	TSS1500	promoter-linked	-2.928	-0.051	-0.077	-0.025	-3.948	0.000167031	0.569894256	ns	-
86	cg10446869	chr1:155147431	island	TRIM46	TRIM46	Body		-2.632	-0.023	-0.035	-0.012	-3.945	0.000167221	0.569894256	ns	-
87	cg15742273	chr19:45974051	S shore	FOSB	FOSB	Body		2.151	-0.013	-0.019	-0.006	-3.942	0.000168981	0.569894256	ns	-
88	cg25974881	chr16:89423869	open sea	ANKRD11	ANKRD11	Body		-2.236	-0.019	-0.029	-0.009	-3.932	0.000175108	0.569894256	ns	-
89	cg07329451	chr2:45494671	open sea	LINC01121	SRBD1			1.691	-0.014	-0.020	-0.007	-3.931	0.000175713	0.569894256	ns	-
90	cg14322570	chr9:135769402	open sea	TSC1	TSC1	3'UTR		2.465	0.018	0.009	0.027	3.927	0.000178056	0.569894256	ns	+
91	cg08965763	chr6:125667609	open sea		HDDC2			-0.261	0.014	0.007	0.021	3.926	0.000178638	0.569894256	ns	+
92	cg11433098	chr15:99987894	open sea		LRRC28			-0.188	-0.025	-0.037	-0.012	-3.923	0.000180316	0.569894256	ns	-
93	cg01747665	chr16:1843424	S shore	IGFALS	IGFALS	Body		1.980	0.018	0.009	0.028	3.922	0.000180885	0.569894256	ns	+
94	cg24740868	chr1:60539362	island	C1orf87	C1orf87	1stExon		-2.022	-0.021	-0.032	-0.010	-3.917	0.000184124	0.569894256	ns	-
95	cg19083626	chr7:55322087	N shore	EGFR	EGFR			2.437	0.034	0.017	0.052	3.913	0.000186624	0.569894256	ns	+
96	cg03604763	chr1:85527490	open sea	DNAI3	DNAI3	TSS1500		1.118	-0.026	-0.040	-0.013	-3.912	0.000187547	0.569894256	ns	-
97	cg07617283	chr7:127913660	S shore		LEP			2.340	-0.032	-0.049	-0.016	-3.912	0.000187716	0.569894256	ns	-
98	cg22740144	chr17:56021390	open sea	CUEDC1	CUEDC1	5'UTR		2.764	0.018	0.009	0.028	3.910	0.000188888	0.569894256	ns	+
99	cg08581297	chr2:198215400	open sea		ANKRD44			1.681	0.014	0.007	0.022	3.905	0.000192397	0.569894256	ns	+
100	cg02586268	chr1:173883567	open sea	SERPINC1	SERPINC1	Body		1.856	-0.020	-0.030	-0.010	-3.902	0.000194015	0.569894256	ns	-
101	cg03346523	chr11:32951061	open sea	QSER1	QSER1	Body		2.258	0.020	0.010	0.031	3.897	0.000197344	0.569894256	ns	+
102	cg04071118	chr20:37555933	S shore	FAM83D	FAM83D	Body	promoter-linked	-2.178	-0.021	-0.032	-0.010	-3.897	0.000197676	0.569894256	ns	-
103	cg05773155	chr1:201911097	open sea	LMOD1	LMOD1	Body		2.951	-0.020	-0.030	-0.010	-3.895	0.000198813	0.569894256	ns	-
104	cg23258304	chr3:140456169	open sea		TRIM42		unknown cellspecific	-1.217	0.027	0.013	0.040	3.894	0.000199786	0.569894256	ns	+
105	cg07391577	chr2:154211042	open sea	AC079150.3	RPRM			0.198	-0.029	-0.044	-0.014	-3.893	0.000200427	0.569894256	ns	-
106	cg08036554	chr10:27080026	open sea	ABI1	ABI1	Body		1.071	0.024	0.012	0.037	3.891	0.000201502	0.569894256	ns	+
107	cg21309351	chr6:138540608	open sea	ARFGEF3	ARFGEF3	Body		2.724	0.014	0.007	0.022	3.891	0.000201635	0.569894256	ns	+
108	cg26022684	chr1:16904881	open sea	NBPF1	NBPF1	Body	unknown cellspecific	1.414	-0.061	-0.092	-0.030	-3.896	0.000202303	0.569894256	ns	-
109	cg18488946	chr15:25230295	open sea	PWAR5	SNRPN	Body		1.710	-0.030	-0.046	-0.015	-3.886	0.000205121	0.569894256	ns	-
110	cg08083923	chr7:107658005	open sea		LAMB4			1.182	-0.015	-0.022	-0.007	-3.881	0.000209112	0.569894256	ns	-
111	cg18986273	chr5:137673771	island	FAM53C	FAM53C	5'UTR	promoter-linked	-2.987	-0.019	-0.029	-0.009	-3.880	0.000209337	0.569894256	ns	-
112	cg25927520	chr7:82137880	open sea		CACNA2D1			0.263	-0.028	-0.043	-0.014	-3.880	0.00020984	0.569894256	ns	-
113	cg25806563	chr18:35282100	open sea		CELF4			-3.979	-0.034	-0.052	-0.017	-3.879	0.000210542	0.569894256	ns	-
114	cg26173959	chr9:124575779	open sea		TTLL11			2.927	0.018	0.009	0.027	3.871	0.000216125	0.569954566	ns	+
115	cg04121938	chr1:201509415	S shore		CSRP1			-2.176	0.028	0.013	0.042	3.871	0.000216551	0.569954566	ns	+
116	cg00244747	chr12:58016626	S shore	SLC26A10	SLC26A10	Body		2.621	0.018	0.009	0.028	3.869	0.000217512	0.569954566	ns	+
117	cg18494043	chr13:67568518	open sea	PCDH9	PCDH9	Body	unknown cellspecific	-1.614	-0.017	-0.026	-0.008	-3.869	0.000218018	0.569954566	ns	-
118	cg26896946	chr5:135416405	island	VTRNA2-1	TGFBI	TSS200	unknown cellspecific	0.179	0.025	0.012	0.037	3.859	0.000225298	0.57955844	ns	+
119	cg18176842	chr2:14776096	S shore	LRATD1	LRATD1	3'UTR		-2.000	-0.025	-0.038	-0.012	-3.858	0.000226195	0.57955844	ns	-
120	cg01673485	chr6:42391208	open sea	TRERF1	TRERF1	5'UTR		1.754	0.015	0.007	0.022	3.857	0.000227376	0.57955844	ns	+
121	cg26744079	chr10:77167091	island	ZNF503	ZNF503	Body		-0.638	0.035	0.017	0.053	3.848	0.000234186	0.588408469	ns	+
122	cg03253150	chr9:140587892	N shore	EHMT1	EHMT1	Body		-2.424	0.026	0.012	0.039	3.847	0.000234695	0.588408469	ns	+
123	cg13699963	chr17:71548861	open sea	SDK2	SDK2	Body		1.282	-0.018	-0.027	-0.009	-3.839	0.000241209	0.593256339	ns	-
124	cg20594802	chr3:189406736	open sea	TP63	TP63	Body		1.043	0.023	0.011	0.035	3.837	0.000243025	0.593256339	ns	+
125	cg10623728	chr9:98879927	open sea	LOC158434	ERCC6L2	TSS1500		2.270	0.018	0.009	0.028	3.836	0.000244361	0.593256339	ns	+
126	cg24881334	chr17:69409104	open sea		SOX9			1.852	-0.018	-0.028	-0.009	-3.834	0.00024572	0.593256339	ns	-
127	cg22195209	chr6:108372344	open sea	OSTM1	OSTM1	Body		0.645	0.023	0.011	0.035	3.833	0.000246327	0.593256339	ns	+
128	cg12949461	chr6:33872364	open sea	RP3-468B3.2	MLN			-3.918	0.031	0.015	0.048	3.829	0.00024989	0.593942875	ns	+
129	cg26276367	chr11:66515246	S shelf	C11orf80	C11orf80	5'UTR		2.739	0.019	0.009	0.028	3.827	0.000252069	0.593942875	ns	+
130	cg00124993	chr5:135416412	island	MIR886	TGFBI	TSS200	unknown cellspecific	-0.297	0.029	0.014	0.044	3.825	0.000253417	0.593942875	ns	+
131	cg14308466	chr9:136781308	open sea	VAV2	VAV2	Body		1.426	0.039	0.019	0.059	3.825	0.000254379	0.593942875	ns	+
132	cg02527139	chr2:208937549	open sea		PLEKHM3			-0.657	-0.021	-0.032	-0.010	-3.820	0.000257653	0.597028009	ns	-
133	cg05332565	chr6:131642853	open sea		AKAP7			1.254	0.028	0.014	0.043	3.817	0.000260917	0.598647065	ns	+
134	cg07873248	chrX:117896197	open sea	IL13RA1	IL13RA1	Body		-1.809	-0.014	-0.022	-0.007	-3.804	0.000272781	0.598647065	ns	-
135	cg12501010	chr3:138176115	open sea	ESYT3	ESYT3	Body		0.589	-0.011	-0.017	-0.005	-3.801	0.000275148	0.598647065	ns	-
136	cg15764052	chr5:115962584	open sea		SEMA6A			1.755	-0.027	-0.041	-0.013	-3.801	0.000275366	0.598647065	ns	-
137	cg07933757	chrX:150066987	island	CD99L2	CD99L2	Body		-1.085	-0.013	-0.020	-0.006	-3.799	0.000277379	0.598647065	ns	-
138	cg11476866	chr17:41920415	open sea		CD300LG			1.621	0.033	0.016	0.051	3.799	0.000277383	0.598647065	ns	+
139	cg26699292	chr16:78079801	island	CLEC3A	CLEC3A			-2.870	0.052	0.025	0.079	3.798	0.000281648	0.598647065	ns	+
140	cg24827726	chr3:14581937	open sea	GRIP2	GRIP2	Body		0.226	0.029	0.014	0.045	3.792	0.000284193	0.598647065	ns	+
141	cg22914344	chr10:91187065	open sea		SLC16A12			1.504	-0.020	-0.030	-0.009	-3.788	0.000287619	0.598647065	ns	-
142	cg23283423	chr17:821546	open sea	NXN	NXN	Body		3.190	0.036	0.017	0.056	3.781	0.000295588	0.598647065	ns	+
143	cg16391801	chr3:135794793	open sea	PPP2R3A	PPP2R3A	Body		0.099	0.029	0.014	0.045	3.778	0.000297576	0.598647065	ns	+
144	cg21091128	chr14:104583799	island	MIR203A	ASPG	Body		-1.259	0.018	0.009	0.028	3.776	0.000300384	0.598647065	ns	+
145	cg20352728	chr22:25758737	S shelf	LRP5L	LRP5L	TSS1500	gene-linked	0.892	0.013	0.006	0.020	3.774	0.000301748	0.598647065	ns	+
146	cg19538873	chr18:13794129	open sea		RNMT			1.520	0.026	0.012	0.040	3.773	0.000302802	0.598647065	ns	+
147	cg23750531	chr16:230196	N shore	HBQ1	HBQ1	TSS200		0.471	0.034	0.016	0.051	3.773	0.00030314	0.598647065	ns	+
148	cg07851597	chr8:28360385	open sea	FZD3	FZD3	5'UTR		-0.070	-0.024	-0.037	-0.011	-3.771	0.000304794	0.598647065	ns	-
149	cg19904905	chr19:49469667	S shore	FTL	FTL	Body	promoter-linked	2.189	0.021	0.010	0.032	3.766	0.000309955	0.598647065	ns	+
150	cg01429764	chr3:117796270	open sea	RP11-384F7.1	IGSF11			2.316	-0.018	-0.028	-0.009	-3.766	0.000310453	0.598647065	ns	-
151	cg02493846	chr1:151809534	N shore	LOC100132111	C2CD4D	TSS1500		-2.208	0.029	0.014	0.044	3.765	0.000311802	0.598647065	ns	+
152	cg11516377	chr17:1933153	island	DPH1	DPH1	TSS1500	promoter-linked	-2.231	-0.020	-0.031	-0.009	-3.764	0.00031259	0.598647065	ns	-

153	cg10666559	chr8:95052953	open sea		CDH17			3.309	-0.019	-0.029	-0.009	-3.763	0.000313765	0.598647065	ns	-
154	cg15747933	chr2:152146478	S shore	NMI	NMI	TSS200	promoter-linked cellspecific	-3.666	-0.028	-0.042	-0.013	-3.758	0.000318842	0.598647065	ns	-
155	cg01818063	chr12:110258216	open sea	TRPV4	TRPV4	5'UTR		2.871	-0.028	-0.044	-0.013	-3.757	0.000319733	0.598647065	ns	-
156	cg10725855	chr5:140589613	N shore	PCDHB12	PCDHB12	1stExon		1.355	0.023	0.011	0.034	3.757	0.000320358	0.598647065	ns	+
157	cg23870575	chr18:70197719	open sea		CBLN2			2.499	0.017	0.008	0.026	3.756	0.000321145	0.598647065	ns	+
158	cg26265281	chr6:75583742	open sea		COL12A1			2.183	0.022	0.010	0.033	3.753	0.000324635	0.598647065	ns	+
159	cg10650127	chr11:2170870	open sea	IGF2	IGF2	TSS200		1.425	-0.019	-0.030	-0.009	-3.753	0.000324968	0.598647065	ns	-
160	cg20784841	chr1:196866322	open sea	CFHR4	CFHR4	Body		0.118	-0.026	-0.039	-0.012	-3.752	0.000325215	0.598647065	ns	-
161	cg08604223	chr5:75903700	open sea	IQGAP2	IQGAP2	Body		2.658	0.019	0.009	0.030	3.752	0.000325635	0.598647065	ns	+
162	cg10885779	chr11:34331908	open sea	ABTB2	ABTB2	Body	unknown cellspecific	1.513	-0.022	-0.034	-0.010	-3.752	0.000325689	0.598647065	ns	-
163	cg16346555	chr2:69374392	open sea	ANTXR1	ANTXR1	Body		2.398	0.016	0.008	0.025	3.749	0.000329425	0.598647065	ns	+
164	cg20366522	chr11:33193745	open sea	CSTF3	CSTF3	Body		2.035	0.024	0.011	0.037	3.746	0.000332604	0.598647065	ns	+
165	cg09252911	chr17:4945668	open sea	SLC52A1	SLC52A1		unknown cellspecific	-0.102	0.013	0.006	0.020	3.744	0.000334416	0.598647065	ns	+
166	cg08284097	chr12:32178932	open sea		RESF1		unknown cellspecific	2.118	0.016	0.008	0.025	3.742	0.000336602	0.598647065	ns	+
167	cg21117808	chr1:9887936	S shelf	AL357140.1	CLSTN1			2.270	-0.022	-0.033	-0.010	-3.742	0.00033729	0.598647065	ns	-
168	cg04408266	chr1:212457538	N shore	PPP2R5A	PPP2R5A	TSS1500	unknown cellspecific	0.835	0.022	0.010	0.034	3.740	0.000339235	0.598647065	ns	+
169	cg14086396	chr5:140479263	N shelf	PCDHB3	PCDHB3	TSS1500		2.157	-0.027	-0.041	-0.013	-3.737	0.000342038	0.598647065	ns	-
170	cg00818326	chr17:7855243	open sea		CNTROB			2.284	0.016	0.007	0.025	3.736	0.000343561	0.598647065	ns	+
171	cg15977798	chr19:30862847	N shelf	ZNF536	ZNF536	TSS1500		1.425	0.017	0.008	0.026	3.735	0.000344945	0.598647065	ns	+
172	cg03895221	chr1:31717101	open sea		NKAIN1			1.533	-0.015	-0.024	-0.007	-3.734	0.000345503	0.598647065	ns	-
173	cg06962011	chr2:177156471	open sea	MTX2	MTX2	Body		2.179	0.020	0.009	0.030	3.734	0.000345636	0.598647065	ns	+
174	cg26587870	chr6:27730563	open sea	LOC100131289	H2BC13			0.797	-0.084	-0.129	-0.040	-3.742	0.000345726	0.598647065	ns	-
175	cg02168270	chr8:50110978	open sea	RP11-10H3.1	PPDPFL			-0.749	0.069	0.032	0.105	3.738	0.000348879	0.598647065	ns	+
176	cg08730256	chr4:170022252	open sea	SH3RF1	SH3RF1	Body		3.146	0.017	0.008	0.027	3.731	0.000349485	0.598647065	ns	+
177	cg01823699	chr9:28236136	open sea	LINGO2	LINGO2	5'UTR		1.514	0.016	0.008	0.025	3.731	0.000349907	0.598647065	ns	+
178	cg25057234	chr1:214821134	open sea	CENPF	CENPF	Body		1.518	0.020	0.009	0.031	3.727	0.000353864	0.598647065	ns	+
179	cg11644987	chr3:122336141	open sea	PARP15	PARP15	Body		-0.542	0.026	0.012	0.040	3.726	0.000355398	0.598647065	ns	+
180	cg14525270	chr1:43766647	N shelf	TIE1	TIE1	TSS200		-1.341	-0.026	-0.041	-0.012	-3.725	0.000356409	0.598647065	ns	-
181	cg04246132	chr19:2943570	N shore	ZNF77	ZNF77	Body		-2.615	0.014	0.007	0.022	3.725	0.000356678	0.598647065	ns	+
182	cg07774964	chr15:65133105	N shore	PLEKHO2	PLEKHO2	TSS1500		0.281	-0.028	-0.044	-0.013	-3.725	0.000356919	0.598647065	ns	-
183	cg04721828	chr11:48285200	open sea	OR4X1	OR4X1	TSS1500		0.660	-0.048	-0.073	-0.022	-3.727	0.000358407	0.598647065	ns	-
184	cg18820942	chr11:70066469	open sea		FADD			-3.386	-0.022	-0.034	-0.010	-3.722	0.000360126	0.598647065	ns	-
185	cg05497504	chr16:71775540	open sea	AP1G1	AP1G1	Body		1.411	0.025	0.012	0.038	3.716	0.000367272	0.602113167	ns	+
186	cg16181613	chrX:77359860	island	PGK1	PGK1	1stExon	promoter-linked	-1.703	-0.017	-0.026	-0.008	-3.716	0.000367617	0.602113167	ns	-
187	cg19754833	chrX:110039080	island	CHRDL1	CHRDL1	5'UTR		-1.991	-0.043	-0.065	-0.020	-3.716	0.000369789	0.602113167	ns	-
188	cg00098216	chr20:46056627	open sea		ZMYND8			0.544	0.020	0.009	0.031	3.713	0.000371937	0.602113167	ns	+
189	cg17659363	chr1:83783719	open sea		TTLL7			0.956	-0.018	-0.027	-0.008	-3.713	0.000372054	0.602113167	ns	-
190	cg27342129	chr16:49857310	open sea	ZNF423	ZNF423	TSS1500		-1.283	-0.021	-0.032	-0.010	-3.707	0.00037884	0.609868184	ns	-
191	cg00792008	chr12:122189621	open sea	TMEM120B	TMEM120B	Body		1.446	0.046	0.021	0.070	3.701	0.000390265	0.622500322	ns	+
192	cg13793157	chr19:51415450	N shore	KLK4	KLK4	TSS1500		-0.131	0.025	0.012	0.039	3.696	0.00039311	0.622500322	ns	+
193	cg06065769	chr2:99065000	S shelf	INPP4A	INPP4A	5'UTR	unknown cellspecific	0.800	0.021	0.010	0.032	3.696	0.000393698	0.622500322	ns	+
194	cg12304663	chr19:55361685	open sea	KIR3DL2	KIR3DL2	TSS1500		1.968	-0.029	-0.045	-0.014	-3.694	0.000395678	0.622500322	ns	-
195	cg13428516	chr19:49224033	island	MAMSTR	MAMSTR	TSS1500	promoter-linked	-1.501	-0.037	-0.057	-0.017	-3.694	0.000397398	0.622500322	ns	-
196	cg21533994	chr12:129294939	open sea	SLC15A4	SLC15A4	Body		3.007	0.059	0.027	0.090	3.695	0.000400984	0.622500322	ns	+
197	cg27452939	chr2:156698957	open sea		NR4A2			1.860	-0.026	-0.039	-0.012	-3.686	0.000406734	0.622500322	ns	-
198	cg26946277	chr17:57394467	open sea		YPEL2		unknown cellspecific	-0.899	0.015	0.007	0.023	3.686	0.000406852	0.622500322	ns	+
199	cg08498208	chr6:108445534	open sea	OSTM1	OSTM1			-0.658	0.021	0.010	0.033	3.684	0.000409868	0.622500322	ns	+
200	cg11410920	chr20:43997025	open sea	SYS1	SYS1	3'UTR	unknown cellspecific	-0.098	0.016	0.007	0.025	3.684	0.000410098	0.622500322	ns	+
201	cg18787447	chr5:85830499	open sea		COX7C			2.168	0.013	0.006	0.020	3.681	0.000414088	0.622500322	ns	+
202	cg27397893	chr2:120282216	S shore	SCTR	SCTR	TSS200		0.548	-0.018	-0.027	-0.008	-3.678	0.000418021	0.622500322	ns	-
203	cg00607755	chr2:42274082	open sea	PKDCC	PKDCC	TSS1500		-0.844	-0.028	-0.043	-0.013	-3.678	0.000418208	0.622500322	ns	-
204	cg24532437	chr20:60593461	open sea	TAF4	TAF4	Body		2.177	0.019	0.009	0.029	3.678	0.000418254	0.622500322	ns	+
205	cg10655471	chr19:41025339	island	SPTBN4	SPTBN4	Body		-0.864	0.018	0.008	0.028	3.678	0.000418293	0.622500322	ns	+
206	cg21965980	chr19:35614992	N shore	FXYD3	FXYD3	3'UTR		1.950	0.015	0.007	0.023	3.676	0.000420945	0.622500322	ns	+
207	cg06790168	chr10:6488016	open sea	PRKCQ	PRKCQ	Body		-2.449	-0.022	-0.035	-0.010	-3.674	0.000424429	0.622500322	ns	-
208	cg25166062	chrX:48958423	island	WDR45	WDR45	TSS1500	promoter-linked	-0.418	-0.024	-0.038	-0.011	-3.673	0.000424971	0.622500322	ns	-
209	cg10001949	chrX:134186517	S shore	RTL8A	RTL8A	TSS1500	promoter-linked	0.561	-0.022	-0.034	-0.010	-3.672	0.000427232	0.622500322	ns	-
210	cg04981300	chr7:66129857	open sea	RABGEF1	RABGEF1	Body		2.629	0.020	0.009	0.030	3.669	0.000430323	0.622500322	ns	+
211	cg17104562	chr13:47217600	open sea	LRCH1	LRCH1	Body		-0.193	0.028	0.013	0.043	3.669	0.000431014	0.622500322	ns	+
212	cg13039082	chr11:77123331	S shore	PAK1	PAK1	5'UTR	promoter-linked	-1.627	-0.014	-0.021	-0.006	-3.665	0.000437387	0.622500322	ns	-
213	cg02571944	chr2:37416022	open sea	SULT6B1	SULT6B1	TSS1500		2.224	0.015	0.007	0.023	3.663	0.000439658	0.622500322	ns	+
214	cg13644160	chr10:125671712	open sea	CPXM2	CPXM2			-0.274	-0.057	-0.088	-0.026	-3.666	0.000442886	0.622500322	ns	-
215	cg00119597	chr20:31235563	open sea	C20orf203	C20orf203	3'UTR		-1.175	0.016	0.008	0.025	3.661	0.000443042	0.622500322	ns	+
216	cg00122679	chr10:125673028	open sea	CPXM2	CPXM2			1.355	-0.045	-0.070	-0.021	-3.662	0.000445074	0.622500322	ns	-
217	cg15717183	chr3:181438122	S shore	SOX2	SOX2	Body		-2.507	-0.035	-0.054	-0.016	-3.659	0.000446403	0.622500322	ns	-
218	cg14463292	chr3:192289252	open sea	FGF12	FGF12	Body		-0.624	-0.042	-0.066	-0.019	-3.659	0.00044882	0.622500322	ns	-
219	cg03953193	chr4:77247610	open sea	CCDC158	CCDC158	Body		1.115	0.026	0.012	0.040	3.657	0.000449352	0.622500322	ns	+
220	cg19719042	chr10:65480632	open sea	RP11-170M17.1	REEP3			-0.256	0.036	0.017	0.056	3.656	0.000450991	0.622500322	ns	+
221	cg24487656	chr1:11986728	S shore	KIAA2013	KIAA2013	TSS1500	promoter-linked cellspecific	-2.936	-0.026	-0.040	-0.012	-3.651	0.000457424	0.622500322	ns	-
222	cg26197136	chr6:154801049	open sea	CNKSR3	CNKSR3	Body		1.332	0.026	0.012	0.040	3.649	0.000460995	0.622500322	ns	+
223	cg18527574	chr2:222434303	N shore	EPHA4	EPHA4	Body		0.839	-0.027	-0.041	-0.012	-3.649	0.000461507	0.622500322	ns	-
224	cg20695175	chr11:134556096	open sea		B3GAT1			1.737	-0.026	-0.040	-0.012	-3.649	0.000461615	0.622500322	ns	-
225	cg12206846	chr1:19292493	open sea		IFFO2		unknown cellspecific	1.692	-0.018	-0.029	-0.008	-3.648	0.000462571	0.622500322	ns	-
226	cg19981409	chr11:89225042	S shore	NOX4	NOX4	TSS1500		0.021	0.025	0.011	0.038	3.647	0.000464049	0.622500322	ns	+
227	cg17179881	chr22:43507665	S shore	BIK	BIK	5'UTR	promoter-linked	-0.791	0.020	0.009	0.031	3.647	0.000464202	0.622500322	ns	+
228	cg26714230	chr13:43566642	S shore	EPSTI1	EPSTI1	TSS1500	promoter-linked	-2.970	0.027	0.012	0.042	3.646	0.000464787	0.622500322	ns	+
229	cg19770749	chr11:98886917	open sea		CNTN5		unknown cellspecific	-2.695	0.021	0.009	0.032	3.644	0.000468199	0.622500322	ns	+

230	cg01735503	chr16:69966815	open sea	WWP2	WWP2	TSS200		1.657	0.019	0.009	0.030	3.641	0.000472694	0.622500322	ns	+
231	cg21960512	chr6:138002351	open sea		OLIG3			-1.249	0.029	0.013	0.044	3.639	0.000475844	0.622500322	ns	+
232	cg20979986	chr5:79477625	open sea	SERINC5	SERINC5	Body	unknown cell specific	-3.021	0.021	0.009	0.032	3.639	0.000476054	0.622500322	ns	+
233	cg26326021	chr5:116365800	open sea	RP11-492L8.2	SEMA6A			0.965	-0.014	-0.022	-0.007	-3.638	0.000478008	0.622500322	ns	-
234	cg12522342	chr17:64831573	island	CACNG5	CACNG5			-1.005	-0.011	-0.016	-0.005	-3.638	0.000478568	0.622500322	ns	-
235	cg13158379	chr4:40211657	open sea	RHOH	RHOH	5'UTR		-0.149	-0.017	-0.027	-0.008	-3.638	0.000478684	0.622500322	ns	-
236	cg15649817	chr8:69636828	open sea	C8orf34	C8orf34	Body		1.764	0.015	0.007	0.023	3.637	0.000480305	0.622500322	ns	+
237	cg10441244	chr17:81051797	S shore	METRNL	METRNL	Body		1.323	0.017	0.008	0.026	3.632	0.000487835	0.624233412	ns	+
238	cg15010006	chr6:31937973	N shore	STK19	STK19	TSS1500		2.585	-0.017	-0.026	-0.008	-3.632	0.000488596	0.624233412	ns	-
239	cg17172683	chr2:133343618	S shore	GPR39	GPR39	Body		1.750	0.025	0.011	0.038	3.630	0.000490782	0.624233412	ns	+
240	cg03836146	chr5:131891674	N shore	RAD50	RAD50	TSS1500		1.922	0.016	0.007	0.024	3.630	0.000491679	0.624233412	ns	+
241	cg09601904	chrX:135402568	open sea	ADGRG4	ADGRG4	Body		0.968	-0.021	-0.033	-0.010	-3.630	0.000491847	0.624233412	ns	-
242	cg19908374	chr2:96829849	open sea		DUSP2			-0.586	0.014	0.006	0.022	3.626	0.000498027	0.626511056	ns	+
243	cg14436939	chr10:49614303	open sea	MAPK8	MAPK8	Body		0.199	0.048	0.022	0.074	3.627	0.00050146	0.626511056	ns	+
244	cg12578063	chr4:74588907	open sea		CXCL8			-1.554	0.032	0.014	0.049	3.623	0.000501858	0.626511056	ns	+
245	cg17737314	chr1:44114355	N shore	KDM4A	KDM4A	TSS1500		-2.781	-0.022	-0.034	-0.010	-3.623	0.000503426	0.626511056	ns	-
246	cg06153538	chr2:176867886	S shore	LNPK	LNPK	TSS1500		2.063	0.018	0.008	0.028	3.621	0.000505408	0.626511056	ns	+
247	cg02348430	chr10:60567288	open sea	BICC1	BICC1	Body		3.210	0.018	0.008	0.028	3.621	0.000505931	0.626511056	ns	+
248	cg17611045	chr3:192289245	open sea	FGF12	FGF12	Body		-0.203	-0.043	-0.067	-0.019	-3.620	0.000510863	0.630005084	ns	-
249	cg26816421	chr22:32022314	N shelf	PISD	PISD	5'UTR	unknown cell specific	1.031	-0.010	-0.015	-0.004	-3.617	0.000512872	0.630005084	ns	-
250	cg21115307	chr16:74943526	open sea	WDR59	WDR59	Body		2.172	0.024	0.011	0.037	3.614	0.00051738	0.633000197	ns	+
251	cg03291996	chr3:178524908	open sea	KCNMB2	KCNMB2	5'UTR		1.312	0.022	0.010	0.034	3.611	0.00052332	0.635028314	ns	+
252	cg06588743	chr11:70139195	open sea	PPFIA1	PPFIA1	Body		2.485	0.021	0.009	0.033	3.611	0.000523966	0.635028314	ns	+
253	cg23719209	chr3:105333123	open sea		ALCAM			2.060	-0.017	-0.026	-0.008	-3.610	0.000525266	0.635028314	ns	-
254	cg13110781	chr19:39108836	N shore	MAP4K1	MAP4K1	TSS200	promoter-linked	-2.530	-0.025	-0.040	-0.011	-3.609	0.000527361	0.635050142	ns	-
255	cg01658523	chr10:103348867	S shore	POLL	POLL	TSS1500	promoter-linked	1.270	-0.018	-0.028	-0.008	-3.606	0.000532235	0.638406035	ns	-
256	cg16852323	chr17:6348225	S shore	PIMREG	PIMREG	5'UTR		-3.223	-0.022	-0.034	-0.010	-3.602	0.000538753	0.640785869	ns	-
257	cg16987982	chr5:58082727	open sea	RAB3C	RAB3C	Body		1.359	-0.030	-0.047	-0.013	-3.602	0.000539132	0.640785869	ns	-
258	cg13097800	chr14:47104140	open sea		MDGA2			0.662	-0.014	-0.021	-0.006	-3.600	0.000543064	0.640785869	ns	-
259	cg24900244	chr12:122692290	S shelf	DIABLO	DIABLO	Body	gene-linked cell specific	2.906	0.016	0.007	0.024	3.599	0.000544635	0.640785869	ns	+
260	cg16400350	chr21:46414569	S shelf	LINC00163	LINC00163			1.666	0.022	0.010	0.034	3.598	0.000546404	0.640785869	ns	+
261	cg01577475	chr2:114033581	island	PAX8	FAM207A	Body		-3.817	0.030	0.014	0.047	3.598	0.000546789	0.640785869	ns	+
262	cg23009254	chr19:42055750	open sea	CEACAM21	CEACAM21	TSS200		-3.665	0.021	0.009	0.033	3.591	0.000558382	0.649835058	ns	+
263	cg04250136	chr1:23515551	open sea		HTR1D			2.610	-0.021	-0.032	-0.009	-3.590	0.000560241	0.649835058	ns	-
264	cg24283027	chr11:67984320	S shelf		KMT5B			3.311	0.022	0.010	0.034	3.589	0.00056306	0.649835058	ns	+
265	cg15640047	chr6:10417516	island	TFAP2A	TFAP2A	Body	unknown cell specific	-3.039	-0.038	-0.059	-0.017	-3.589	0.000564241	0.649835058	ns	-
266	cg14803733	chr20:31100703	open sea	NOL4L	NOL4L	Body		1.409	0.021	0.009	0.032	3.588	0.000565133	0.649835058	ns	+
267	cg04525002	chr17:6066300	open sea		WSCD1			2.746	0.019	0.009	0.030	3.583	0.000574934	0.658055602	ns	+
268	cg06925836	chrX:48542214	open sea	WAS	WAS	5'UTR	promoter-linked cell specific	-1.543	-0.019	-0.029	-0.008	-3.582	0.000576585	0.658055602	ns	-
269	cg17586516	chrX:70502859	N shore	NONO	NONO	TSS200	promoter-linked	1.054	-0.021	-0.032	-0.009	-3.580	0.000580631	0.660210243	ns	-
270	cg13029423	chr16:86549276	island		FOXF1			-2.035	-0.019	-0.029	-0.008	-3.576	0.000587425	0.663005956	ns	-
271	cg05234035	chr5:172673203	S shore		NKX2-5			-1.823	0.025	0.011	0.039	3.576	0.000587425	0.663005956	ns	+
272	cg06580879	chr17:4443121	S shelf	SPNS2	SPNS2	3'UTR	unknown cell specific	3.139	0.019	0.009	0.030	3.574	0.000591429	0.663142472	ns	+
273	cg15015204	chr17:52976225	N shore	TOM1L1	TOM1L1			1.816	-0.038	-0.059	-0.017	-3.575	0.000591882	0.663142472	ns	-
274	cg15461542	chr1:20712767	open sea	LINC01141	VWA5B1	Body		1.257	-0.019	-0.029	-0.008	-3.571	0.000597711	0.665618713	ns	-
275	cg18676958	chr4:126672262	open sea	RP11-399F2.2	VWA5B1			2.208	-0.024	-0.037	-0.010	-3.570	0.000599484	0.665618713	ns	-
276	cg09437423	chr3:108541505	open sea	TRAT1	FAT4	TSS200	unknown cell specific	0.097	0.030	0.013	0.046	3.569	0.000600621	0.665618713	ns	+
277	cg06274263	chr12:10335159	open sea	TMEM52B	TMEM52B	ExonBnd		-2.939	-0.027	-0.042	-0.012	-3.566	0.000607989	0.671351177	ns	-
278	cg09563657	chr6:116382880	open sea	FRK	FRK	TSS1500		1.616	0.015	0.006	0.023	3.563	0.000612635	0.673226087	ns	+
279	cg25424310	chr6:90220046	open sea	ANKRD6	ANKRD6	5'UTR		-2.268	-0.023	-0.036	-0.010	-3.563	0.000614193	0.673226087	ns	-
280	cg25261703	chr6:155510046	open sea	TIAM2	TIAM2	Body		-1.125	-0.033	-0.051	-0.015	-3.562	0.00061629	0.673226087	ns	-
281	cg15866393	chr12:132900480	island	GALNT9	GALNT9	Body	unknown cell specific	1.356	-0.019	-0.029	-0.008	-3.559	0.000621447	0.673267469	ns	-
282	cg06557644	chr7:30510463	open sea	NOD1	NOD1	5'UTR		-0.269	0.016	0.007	0.024	3.558	0.000622888	0.673267469	ns	+
283	cg24645513	chr4:80748683	open sea	PCAT4	ANTXR2	Body		-0.113	0.011	0.005	0.017	3.558	0.000624218	0.673267469	ns	+
284	cg07969668	chr8:142841913	island		MROH5			2.690	-0.023	-0.036	-0.010	-3.556	0.000626668	0.673267469	ns	-
285	cg19184818	chr17:73235564	open sea	GGA3	GGA3	Body	promoter-linked	2.236	-0.015	-0.024	-0.007	-3.556	0.00062819	0.673267469	ns	-
286	cg16375547	chr10:87484170	open sea	GRID1	GRID1	Body		1.342	-0.015	-0.024	-0.007	-3.555	0.000629535	0.673267469	ns	-
287	cg05942199	chr13:49661095	open sea	FNDC3A	FNDC3A	Body		0.954	-0.020	-0.031	-0.009	-3.552	0.000635495	0.677222577	ns	-
288	cg10987788	chr11:34535377	open sea	ELF5	ELF5	TSS200		2.710	0.020	0.009	0.031	3.551	0.000638516	0.677222577	ns	+
289	cg08265622	chrX:17567760	open sea	NHS	NHS	Body		1.527	-0.016	-0.026	-0.007	-3.550	0.00064011	0.677222577	ns	-
290	cg25352714	chr15:64391206	S shelf	SNX1	SNX1	Body		2.003	0.012	0.005	0.019	3.549	0.00064229	0.677222577	ns	+
291	cg13180165	chr5:125867455	open sea		ALDH7A1			2.817	-0.016	-0.025	-0.007	-3.548	0.000644303	0.677222577	ns	-
292	cg04458920	chr2:54483619	S shore	TSPYL6	TSPYL6	TSS1500		2.131	0.014	0.006	0.021	3.546	0.000648549	0.678891033	ns	+
293	cg02164605	chrX:13016240	open sea		TMSB4X			0.013	0.022	0.010	0.035	3.545	0.00065033	0.678891033	ns	+
294	cg05650511	chr1:112024620	open sea		TMIGD3			0.208	-0.023	-0.036	-0.010	-3.541	0.000659372	0.685989217	ns	-
295	cg11641916	chr8:1923815	S shore	KBTBD11	KBTBD11	Body		2.444	0.011	0.005	0.018	3.536	0.000669119	0.692795939	ns	+
296	cg06282247	chr2:151281841	open sea		RND3			-1.010	-0.017	-0.027	-0.008	-3.536	0.000670445	0.692795939	ns	-
297	cg25152193	chr1:197874469	S shelf	C1orf53	C1orf53	Body		-1.019	-0.028	-0.043	-0.012	-3.534	0.000674689	0.693355509	ns	-
298	cg26975265	chr18:8801547	open sea	MTCL1	MTCL1	Body		3.160	-0.026	-0.040	-0.011	-3.532	0.000678423	0.693355509	ns	-
299	cg02002333	chr1:49146361	open sea	AGBL4	AGBL4	Body		2.693	-0.020	-0.031	-0.009	-3.532	0.000680016	0.693355509	ns	-
300	cg00849282	chr11:74405496	open sea		CHRDL2		unknown cell specific	0.761	0.013	0.006	0.020	3.532	0.000680054	0.693355509	ns	+
301	cg17270101	chr1:57286560	open sea	FYB2	FYB2	TSS1500		0.084	-0.020	-0.031	-0.009	-3.529	0.000686246	0.697344154	ns	-
302	cg06994882	chr3:15482242	open sea	EAF1	EAF1	3'UTR		2.559	-0.020	-0.031	-0.009	-3.527	0.000691143	0.699240459	ns	-
303	cg08491314	chrX:124336782	island		TEX13C			-2.298	-0.032	-0.050	-0.014	-3.526	0.000692753	0.699240459	ns	-
304	cg24499994	chr15:65866127	open sea	HACD3	HACD3	Body		1.459	0.024	0.011	0.038	3.524	0.000696929	0.699240459	ns	+
305	cg15848553	chr10:126390998	open sea	FAM53B	FAM53B	Body		-2.132	0.012	0.005	0.019	3.523	0.000700531	0.699240459	ns	+

AAS: APPETITIVE AGGRESSION

rank	cpg	cpg.position	region	annotation	gene	gene.group	gene.feature	AveExpr	logFC	CI.L	CI.R	t	P.Value	adj.P.Val	fdr	sign
1	cg09170207	chr15:71071082	open sea	RP11-138H8.3	UACA			2.140	-0.009	-0.012	-0.005	-5.317	8.89E-07	0.131447024	ns	-
2	cg15863118	chr4:190480671	open sea		FRG1			2.259	0.015	0.010	0.021	5.254	1.15E-06	0.131447024	ns	+
3	cg26514430	chr5:140249080	island	PCDHA6	PCDHA6	Body		-0.541	-0.014	-0.019	-0.008	-5.157	1.70E-06	0.131447024	ns	-
4	cg05697527	chr15:48889252	open sea	FBN1	FBN1	Body		-0.303	-0.058	-0.080	-0.035	-5.140	1.95E-06	0.131447024	ns	-
5	cg27491702	chr10:124311123	open sea		DMBT1		unknown cell specific	-0.567	-0.015	-0.021	-0.009	-5.099	2.15E-06	0.131447024	ns	-
6	cg26875805	chr1:166890429	island	ILDR2	ILDR2	Body		-4.222	-0.027	-0.039	-0.016	-4.855	5.70E-06	0.230017585	ns	-
7	cg14221852	chr3:197457184	open sea	RUBCN	RUBCN	Body	unknown cell specific	1.571	0.014	0.008	0.020	4.843	5.92E-06	0.230017585	ns	+
8	cg14285795	chr17:71179222	open sea	RP11-143K11.5	COG1			2.181	0.014	0.008	0.019	4.831	6.21E-06	0.230017585	ns	+
9	cg19192159	chr5:140743798	N shore	PCDHGA5	PCDHGA5	TSS200		-1.813	-0.025	-0.035	-0.015	-4.810	6.77E-06	0.230017585	ns	-
10	cg06661574	chr14:67994779	open sea	TMEM229B	TMEM229B			2.787	0.010	0.006	0.015	4.730	9.20E-06	0.280298696	ns	+
11	cg07195851	chr5:3517522	open sea	LINC01019	IRX1			1.199	-0.013	-0.019	-0.008	-4.688	1.08E-05	0.280298696	ns	-
12	cg00922157	chr8:1117323	S shelf	DLGAP2	DLGAP2			2.579	-0.016	-0.023	-0.009	-4.659	1.21E-05	0.280298696	ns	-
13	cg01204927	chr6:28505394	open sea	TRI-TAT3-1	GPX5			-1.108	-0.012	-0.017	-0.007	-4.649	1.25E-05	0.280298696	ns	-
14	cg17794320	chr15:90190822	N shore	KIF7	KIF7	Body		2.359	-0.016	-0.023	-0.009	-4.640	1.30E-05	0.280298696	ns	-
15	cg00808170	chr5:140807787	island	PCDHGA4	PCDHGA4	Body		0.000	-0.008	-0.012	-0.005	-4.625	1.37E-05	0.280298696	ns	-
16	cg23646888	chr3:112929672	N shore	BOC	BOC			0.022	-0.007	-0.010	-0.004	-4.604	1.49E-05	0.285381991	ns	-
17	cg01837367	chr3:194989568	N shore	XXYLT1	XXYLT1	Body		2.775	-0.009	-0.013	-0.005	-4.539	1.91E-05	0.291614952	ns	-
18	cg25619978	chr5:135558602	open sea	TRPC7	TRPC7	Body		1.795	0.010	0.006	0.015	4.501	2.20E-05	0.291614952	ns	+
19	cg04634417	chr5:140220803	N shore	PCDHA6	PCDHA6	Body		2.478	-0.015	-0.022	-0.009	-4.484	2.35E-05	0.291614952	ns	-
20	cg20211004	chrX:96276763	open sea	DIAPH2	DIAPH2	Body		0.736	0.011	0.006	0.016	4.483	2.36E-05	0.291614952	ns	+
21	cg02506275	chr12:130650643	S shore		FZD10			1.676	0.016	0.009	0.023	4.477	2.42E-05	0.291614952	ns	+
22	cg18731546	chr10:3023726	open sea		PFKP			1.804	0.009	0.005	0.013	4.476	2.42E-05	0.291614952	ns	+
23	cg23348353	chr4:183795310	open sea		DCTD			2.706	0.013	0.007	0.019	4.470	2.48E-05	0.291614952	ns	+
24	cg25413843	chr2:71126691	N shore	VAX2	VAX2	TSS1500		-2.520	-0.009	-0.013	-0.005	-4.446	2.71E-05	0.291614952	ns	-
25	cg20802033	chrX:107277111	open sea		VSIG1			1.899	-0.014	-0.021	-0.008	-4.441	2.77E-05	0.291614952	ns	-
26	cg10078507	chr17:55632268	open sea	MSI2	MSI2	Body		2.826	0.011	0.006	0.017	4.436	2.81E-05	0.291614952	ns	+
27	cg09008712	chr14:90956536	open sea	RP11-1078H9.6	TTC7B			3.123	0.012	0.006	0.017	4.430	2.88E-05	0.291614952	ns	+
28	cg25710235	chr13:21276842	N shore	IL17D	IL17D	TSS1500		-2.989	-0.012	-0.018	-0.007	-4.429	2.89E-05	0.291614952	ns	-
29	cg21376883	chr1:236850232	island	ACTN2	ACTN2	Body	unknown cell specific	-2.450	-0.017	-0.024	-0.009	-4.428	2.90E-05	0.291614952	ns	-
30	cg03216580	chr10:3818357	open sea	KLF6	KLF6	Body		0.152	-0.006	-0.009	-0.003	-4.426	2.93E-05	0.291614952	ns	-
31	cg00962635	chrX:99986352	N shore	SYTL4	SYTL4	5'UTR		-1.456	-0.015	-0.022	-0.008	-4.423	2.96E-05	0.291614952	ns	-
32	cg21179056	chr20:56444780	open sea		PMEPA1			1.722	-0.012	-0.017	-0.006	-4.398	3.25E-05	0.292351567	ns	-
33	cg08399336	chr4:129488643	open sea		JADE1			2.391	-0.013	-0.019	-0.007	-4.393	3.31E-05	0.292351567	ns	-
34	cg14358088	chr6:2063957	open sea	GMDS	GMDS	Body		0.659	0.013	0.007	0.018	4.386	3.40E-05	0.292351567	ns	+
35	cg17092985	chr8:20119213	open sea	LZTS1	LZTS1			1.678	-0.014	-0.020	-0.008	-4.381	3.46E-05	0.292351567	ns	-
36	cg10682219	chr10:63660856	N shelf	ARID5B	ARID5B	TSS200		-4.080	-0.030	-0.044	-0.016	-4.385	3.46E-05	0.292351567	ns	-
37	cg08701429	chr17:15394553	open sea	CDRT4	CDRT4			1.227	0.019	0.010	0.028	4.370	3.60E-05	0.292351567	ns	+
38	cg14911856	chr1:47999856	S shore		FOXD2			2.510	-0.013	-0.018	-0.007	-4.365	3.67E-05	0.292351567	ns	-
39	cg07380881	chr2:233707561	open sea	GIGYF2	GIGYF2	Body		-0.219	0.020	0.011	0.030	4.351	3.86E-05	0.292351567	ns	+
40	cg02873954	chr21:45161415	open sea	PDXK	PDXK	Body		0.635	0.047	0.026	0.069	4.352	4.01E-05	0.292351567	ns	+
41	cg25982505	chr11:59318777	open sea	TRR-TCT3-2	OSBP		promoter-linked	-2.261	-0.010	-0.014	-0.005	-4.341	4.01E-05	0.292351567	ns	-
42	cg17951445	chr4:30842020	open sea	PCDH7	PCDH7	Body		0.269	0.015	0.008	0.022	4.336	4.09E-05	0.292351567	ns	+
43	cg09738410	chr11:2442304	island	TRPM5	TRPM5	Body		-0.749	0.010	0.005	0.014	4.334	4.11E-05	0.292351567	ns	+
44	cg11445191	chr5:140227765	N shore	PCDHA6	PCDHA6	Body		-0.205	-0.022	-0.032	-0.012	-4.325	4.26E-05	0.295802443	ns	-
45	cg16134323	chr5:140562034	N shore	PCDHB16	PCDHB16	5'UTR		-3.033	-0.013	-0.018	-0.007	-4.312	4.47E-05	0.29616722	ns	-
46	cg11659341	chr15:53150412	open sea		ONECUT1			0.883	-0.011	-0.017	-0.006	-4.308	4.53E-05	0.29616722	ns	-
47	cg09242378	chrX:11783772	open sea	MSL3	MSL3	Body		3.322	0.022	0.012	0.033	4.306	4.58E-05	0.29616722	ns	+
48	cg05830480	chr1:147123523	open sea	ACP6	ACP6	Body		2.427	-0.023	-0.034	-0.013	-4.294	4.79E-05	0.29616722	ns	-
49	cg12738529	chr14:56039435	open sea	KTN1	KTN1			2.773	0.012	0.006	0.017	4.290	4.83E-05	0.29616722	ns	+
50	cg12304113	chr8:139926188	open sea	COL22A1	COL22A1	5'UTR		-2.990	-0.012	-0.017	-0.006	-4.290	4.84E-05	0.29616722	ns	-
51	cg19183885	chr5:172207414	open sea	Y_RNA	DUSP1			1.522	-0.010	-0.015	-0.005	-4.244	5.72E-05	0.33856836	ns	-
52	cg19475030	chr3:75273912	open sea		HNRNPA3P6			2.570	-0.009	-0.013	-0.005	-4.243	5.76E-05	0.33856836	ns	-
53	cg11996983	chr16:58269857	open sea	CCDC113	CCDC113			-0.303	-0.007	-0.011	-0.004	-4.230	6.04E-05	0.34848415	ns	-
54	cg12684668	chr5:150403466	S shelf	GPX3	GPX3	Body		-2.363	0.014	0.007	0.020	4.209	6.52E-05	0.369253352	ns	+
55	cg21483311	chr7:1793123	open sea		ELFN1			-1.553	0.014	0.007	0.020	4.198	6.77E-05	0.376719383	ns	+
56	cg11728928	chr17:34415818	open sea	CCL3	CCL3	3'UTR		-1.188	-0.015	-0.022	-0.008	-4.193	6.90E-05	0.376719383	ns	-
57	cg10994379	chr5:35939420	open sea	CAPSL	CAPSL	TSS1500		1.555	-0.009	-0.013	-0.005	-4.183	7.14E-05	0.380468978	ns	-
58	cg15963673	chr14:21623717	open sea	OR5AU1	OR5AU1	1stExon		2.667	0.013	0.007	0.018	4.181	7.21E-05	0.380468978	ns	+
59	cg05119290	chr17:4439394	island	SPNS2	SPNS2	Body	unknown cell specific	2.625	-0.021	-0.031	-0.011	-4.172	7.45E-05	0.384010436	ns	-
60	cg01683883	chr16:66613053	island	CMTM2	CMTM2	TSS1500	unknown cell specific	-3.047	-0.018	-0.027	-0.010	-4.169	7.53E-05	0.384010436	ns	-
61	cg11226105	chr1:29102519	S shore		YTHDF2			1.430	-0.015	-0.022	-0.008	-4.152	7.99E-05	0.39147688	ns	-
62	cg12792526	chr21:43098958	open sea	LINC00111	RIPK4	TSS1500		-0.100	-0.009	-0.013	-0.004	-4.150	8.05E-05	0.39147688	ns	-
63	cg09684998	chr3:111823744	open sea	C3orf52	C3orf52	Body		0.637	0.012	0.006	0.018	4.145	8.20E-05	0.39147688	ns	+
64	cg10195647	chr11:6585145	open sea	DNHD1	DNHD1	Body		3.083	-0.018	-0.026	-0.009	-4.145	8.22E-05	0.39147688	ns	-
65	cg26340700	chr1:231663886	N shore	TSNAX-DISC1	TSNAX	TSS1500	promoter-linked	-1.098	-0.011	-0.016	-0.005	-4.138	8.40E-05	0.39147688	ns	-
66	cg18041719	chr7:84671560	open sea	SEMA3D	SEMA3D	Body		1.748	0.009	0.005	0.014	4.133	8.58E-05	0.39147688	ns	+
67	cg08048709	chr18:9173689	open sea	ANKRD12	ANKRD12	5'UTR		1.843	0.018	0.009	0.027	4.122	8.90E-05	0.39147688	ns	+
68	cg12135344	chr1:6188120	island	CHD5	CHD5	Body	unknown cell specific	1.061	-0.021	-0.031	-0.011	-4.120	8.98E-05	0.39147688	ns	-
69	cg26976437	chr8:143781644	island	LY6K	LY6K	1stExon		0.486	-0.012	-0.018	-0.006	-4.117	9.08E-05	0.39147688	ns	-
70	cg05569877	chr16:22203612	S shore		SDR42E2		promoter-linked	-2.643	-0.035	-0.052	-0.018	-4.120	9.19E-05	0.39147688	ns	-
71	cg15752954	chr7:35548006	open sea	AC007652.1	HERPUD2			2.886	-0.009	-0.014	-0.005	-4.113	9.22E-05	0.39147688	ns	-
72	cg11288769	chr15:37188101	open sea	MEIS2	MEIS2	Body		2.082	-0.010	-0.015	-0.005	-4.103	9.53E-05	0.39147688	ns	-
73	cg09757109	chr11:111848638	S shore	DIXDC1	DIXDC1	Body	promoter-linked	-3.212	-0.015	-0.022	-0.008	-4.103	9.55E-05	0.39147688	ns	-
74	cg06258593	chr16:48199609	open sea	RP11-3M1.1	ABCC11			-2.704	-0.013	-0.020	-0.007	-4.101	9.60E-05	0.39147688	ns	-
75	cg17669572	chr1:156130625	open sea	SEMA4A	SEMA4A	Body	gene-linked cell specific	-2.113	-0.010	-0.015	-0.005	-4.094	9.86E-05	0.39147688	ns	-

76	cg23639072	chr10:17045334	open sea	CUBN	CUBN	Body		-0.387	0.016	0.008	0.024	4.093	9.89E-05	0.39147688	ns	+
77	cg10062919	chr17:38503802	S shore	RARA	RARA	Body	unknown cell specific	-0.396	-0.006	-0.009	-0.003	-4.093	9.90E-05	0.39147688	ns	-
78	cg13066289	chr2:134135267	open sea	NCKAP5	NCKAP5	Body		-0.754	0.013	0.007	0.020	4.085	0.000101679	0.39147688	ns	+
79	cg10822116	chr9:35733370	S shore	CREB3	CREB3	Body	promoter-linked	-2.847	0.012	0.006	0.018	4.085	0.000101746	0.39147688	ns	+
80	cg16425161	chr15:47624584	open sea	SEMA6D	SEMA6D			1.489	-0.011	-0.016	-0.006	-4.083	0.000102391	0.39147688	ns	-
81	cg13223677	chr6:10534773	open sea	GCNT2	GCNT2	Body	unknown cell specific	3.188	0.012	0.006	0.018	4.075	0.000105651	0.394483335	ns	+
82	cg11926937	chr17:39509300	open sea		KRT33A			2.290	-0.011	-0.016	-0.005	-4.068	0.000108019	0.394483335	ns	-
83	cg24650267	chr10:110226691	S shore		SORCS1			2.007	-0.012	-0.018	-0.006	-4.068	0.000108085	0.394483335	ns	-
84	cg00643323	chrX:31154609	open sea	DMD	DMD	Body		0.878	0.014	0.007	0.021	4.065	0.000109438	0.394483335	ns	+
85	cg19067854	chr1:173272600	open sea	LOC100506023	TNFSF4	Body		0.406	0.012	0.006	0.018	4.064	0.000109626	0.394483335	ns	+
86	cg25075860	chr7:31553568	open sea	ITPRID1	ITPRID1	TSS200		1.337	-0.012	-0.018	-0.006	-4.059	0.000111743	0.397424035	ns	-
87	cg22629554	chr19:49063736	S shore	SULT2B1	SULT2B1	Body		1.581	0.008	0.004	0.012	4.053	0.000113934	0.399430573	ns	+
88	cg01698058	chrX:81485693	open sea		SH3BGRL			0.427	0.014	0.007	0.021	4.051	0.000114918	0.399430573	ns	+
89	cg19495013	chr12:52214119	N shore	FIGNL2	FIGNL2	1stExon		-1.930	0.015	0.007	0.022	4.045	0.000117207	0.400822479	ns	+
90	cg13718185	chr8:22877275	open sea	RHOBTB2	RHOBTB2	3'UTR	promoter-linked	-1.345	0.012	0.006	0.018	4.044	0.00011794	0.400822479	ns	+
91	cg07395826	chr2:63377498	open sea	WDPCP	WDPCP	Body		-1.529	0.020	0.010	0.030	4.036	0.000121633	0.408832372	ns	+
92	cg04769341	chrX:102585202	open sea	TCEAL7	TCEAL7	1stExon		0.375	-0.007	-0.011	-0.004	-4.027	0.000125084	0.414941258	ns	-
93	cg06099218	chrX:39473248	island		MIR3937		unknown cell specific	-3.034	-0.019	-0.029	-0.010	-4.025	0.000126164	0.414941258	ns	-
94	cg00702547	chr16:34214362	open sea		CCNYL3			-1.100	-0.010	-0.015	-0.005	-4.015	0.00013069	0.415071701	ns	-
95	cg08948575	chr6:129128924	open sea		LAMA2			-2.165	0.009	0.004	0.013	4.012	0.000131801	0.415071701	ns	+
96	cg01165776	chr2:119602546	N shore	EN1	EN1	Body		-2.950	-0.010	-0.015	-0.005	-4.010	0.000132917	0.415071701	ns	-
97	cg15138986	chr10:131935156	island	GLRX3	GLRX3	Body	promoter-linked cell specific	-2.387	-0.020	-0.030	-0.010	-4.008	0.00013412	0.415071701	ns	-
98	cg25278153	chr18:1805667	open sea		METTL4			-0.662	0.013	0.007	0.019	4.006	0.000135033	0.415071701	ns	+
99	cg04865056	chr12:132896405	island	GALNT9	GALNT9	Body		2.314	0.014	0.007	0.021	4.003	0.000136037	0.415071701	ns	+
100	cg15635761	chr13:45411234	open sea		NUFIP1			2.022	0.012	0.006	0.018	3.998	0.000138846	0.415071701	ns	+
101	cg03234839	chr21:46408037	island		FAM207A			1.517	-0.019	-0.029	-0.010	-3.993	0.00014111	0.415071701	ns	-
102	cg03954344	chr11:32417924	open sea	WT1	WT1	Body		3.305	-0.027	-0.041	-0.014	-3.996	0.000141286	0.415071701	ns	-
103	cg02961533	chrX:138287257	island	FGF13	FGF13	TSS200		-1.712	-0.021	-0.032	-0.011	-3.993	0.000141678	0.415071701	ns	-
104	cg16819392	chr10:23326418	open sea	ARMC3	ARMC3	3'UTR		2.548	-0.010	-0.015	-0.005	-3.991	0.000142096	0.415071701	ns	-
105	cg09063936	chr2:74408977	S shelf		MOB1A		unknown cell specific	-2.024	-0.008	-0.013	-0.004	-3.990	0.000142488	0.415071701	ns	-
106	cg18675097	chr6:28227127	island	NKAPL	NKAPL	5'UTR	promoter-linked cell specific	-1.726	-0.023	-0.034	-0.011	-3.986	0.000145562	0.415498423	ns	-
107	cg13900873	chr14:65696017	open sea		MAX			0.531	0.007	0.003	0.010	3.979	0.000148081	0.415498423	ns	+
108	cg02402091	chr10:114135092	open sea	ACSL5	ACSL5	TSS1500	promoter-linked	0.870	0.010	0.005	0.014	3.977	0.000149222	0.415498423	ns	+
109	cg11234688	chr2:1609660	open sea	AC144450.1	PXDN			0.726	-0.021	-0.031	-0.010	-3.971	0.000152857	0.415498423	ns	-
110	cg02775256	chrX:64708686	open sea	ZC3H12B	ZC3H12B	TSS200		2.606	-0.011	-0.017	-0.006	-3.970	0.000152944	0.415498423	ns	-
111	cg20798375	chr4:23947535	open sea	PPARGC1A	PPARGC1A			-1.155	0.020	0.010	0.030	3.969	0.000154076	0.415498423	ns	+
112	cg07204658	chr1:160139687	open sea	ATP1A4	ATP1A4	Body	unknown cell specific	-1.243	0.015	0.007	0.022	3.967	0.000154584	0.415498423	ns	+
113	cg06345027	chr10:74695298	open sea	PLA2G12B	PLA2G12B	3'UTR		2.273	-0.011	-0.017	-0.006	-3.962	0.000157264	0.415498423	ns	-
114	cg00935967	chr1:3822762	N shore	LINC01134	C1orf174	Body		2.038	0.016	0.008	0.024	3.961	0.000158063	0.415498423	ns	+
115	cg02897385	chr5:142528481	open sea	ARHGAP26	ARHGAP26	Body		1.084	0.012	0.006	0.017	3.958	0.000159501	0.415498423	ns	+
116	cg13904497	chr13:105789144	open sea		DAOA			3.059	-0.007	-0.011	-0.004	-3.957	0.000160102	0.415498423	ns	-
117	cg01030624	chr6:151814669	N shore	CCDC170	CCDC170	TSS1500		1.297	0.015	0.007	0.023	3.957	0.000160462	0.415498423	ns	+
118	cg08016417	chr4:153588259	open sea	TMEM154	TMEM154	Body		0.513	0.015	0.007	0.022	3.956	0.000160997	0.415498423	ns	+
119	cg06981242	chrX:138287529	island	FGF13	FGF13	TSS1500		-0.395	-0.014	-0.021	-0.007	-3.954	0.000161833	0.415498423	ns	-
120	cg13851904	chr1:47899230	island	FOXD2	FOXD2	Body		-2.279	-0.013	-0.019	-0.006	-3.952	0.000163011	0.415498423	ns	-
121	cg05435490	chr11:72313195	open sea	PDE2A	PDE2A	Body		1.964	0.010	0.005	0.015	3.942	0.000168767	0.423246073	ns	+
122	cg22968887	chr2:222506138	open sea	AC068489.1	EPHA4			0.014	0.010	0.005	0.015	3.936	0.000172652	0.423246073	ns	+
123	cg19049194	chr2:175193754	island	LINC01305	SP9			-1.509	-0.009	-0.013	-0.004	-3.932	0.000174772	0.423246073	ns	-
124	cg00945580	chr11:131416222	open sea	NTM	NTM	Body		1.878	0.012	0.006	0.018	3.932	0.000175081	0.423246073	ns	+
125	cg16713962	chr16:49217909	open sea		CBLN1			0.574	0.007	0.004	0.011	3.930	0.000176316	0.423246073	ns	+
126	cg07620843	chr1:34864643	open sea		C1orf94			-1.229	0.013	0.006	0.020	3.929	0.000176847	0.423246073	ns	+
127	cg21757281	chr4:183795822	open sea		DCTD			-0.318	0.021	0.011	0.032	3.928	0.000177972	0.423246073	ns	+
128	cg23746638	chr6:40991828	N shelf		UNC5CL			1.789	0.010	0.005	0.016	3.927	0.000178129	0.423246073	ns	+
129	cg05715544	chr17:3783348	open sea	CAMKK1	CAMKK1	Body		2.397	-0.007	-0.010	-0.003	-3.923	0.000180197	0.423246073	ns	-
130	cg16354207	chr2:238874908	N shore	UBE2F	UBE2F	TSS1500		-2.538	-0.015	-0.022	-0.007	-3.923	0.00018066	0.423246073	ns	-
131	cg19031085	chr5:135228288	open sea	IL9	IL9	Body		-0.030	0.015	0.007	0.023	3.915	0.000185345	0.423246073	ns	+
132	cg19920602	chr2:101397446	open sea		NPAS2			-0.878	0.012	0.006	0.018	3.914	0.000186296	0.423246073	ns	+
133	cg07033722	chr1:40539032	open sea	PPT1	PPT1	3'UTR	gene-linked cell specific	-1.333	0.014	0.007	0.021	3.912	0.000187485	0.423246073	ns	+
134	cg05986687	chrX:46936365	N shore	RGN	RGN	TSS1500		1.405	0.010	0.005	0.015	3.907	0.000190874	0.423246073	ns	+
135	cg24014578	chr8:29352838	open sea		DUSP4			1.085	0.009	0.005	0.014	3.906	0.000191684	0.423246073	ns	+
136	cg04974775	chr5:114516854	S shore	TRIM36	TRIM36	TSS1500		2.502	-0.007	-0.011	-0.004	-3.905	0.000191999	0.423246073	ns	-
137	cg12053762	chr12:107167543	N shore	RIC8B	RIC8B	TSS1500	promoter-linked	-1.316	0.011	0.005	0.016	3.905	0.000192509	0.423246073	ns	+
138	cg02600505	chr5:169026626	open sea	SPDL1	SPDL1	Body		3.235	0.011	0.005	0.016	3.898	0.000197139	0.423246073	ns	+
139	cg18477771	chr11:120195466	N shore	TLCD5	TLCD5	TSS1500		2.015	-0.011	-0.016	-0.005	-3.891	0.000201983	0.423246073	ns	-
140	cg00695416	chr21:37442476	island	CBR1	CBR1	1stExon		-3.767	0.030	0.015	0.045	3.895	0.000202336	0.423246073	ns	+
141	cg11533763	chr10:88227406	open sea	WAPL	WAPL	Body		1.934	-0.007	-0.011	-0.004	-3.890	0.000202532	0.423246073	ns	-
142	cg18977891	chr8:134571813	open sea	ST3GAL1	ST3GAL1	5'UTR	unknown cell specific	-2.864	-0.015	-0.023	-0.007	-3.890	0.000202792	0.423246073	ns	-
143	cg04691233	chr11:133989435	open sea	JAM3	JAM3	Body		2.755	-0.011	-0.016	-0.005	-3.888	0.000204009	0.423246073	ns	-
144	cg13834443	chr15:65221205	open sea	ANKDD1A	ANKDD1A	Body		0.430	-0.013	-0.020	-0.006	-3.888	0.000204186	0.423246073	ns	-
145	cg24107728	chr1:53760337	open sea	LRP8	LRP8	Body		2.075	0.014	0.007	0.021	3.887	0.00020493	0.423246073	ns	+
146	cg13193497	chr6:159344810	open sea		C6orf99			0.769	-0.005	-0.008	-0.003	-3.886	0.000205373	0.423246073	ns	-
147	cg18562935	chr20:56726000	island	C20orf85	C20orf85	5'UTR		-2.043	-0.007	-0.011	-0.004	-3.885	0.00020627	0.423246073	ns	-
148	cg21115391	chr11:93143810	open sea	DEUP1	DEUP1	Body		1.392	-0.011	-0.017	-0.005	-3.884	0.000206603	0.423246073	ns	-
149	cg26712714	chr14:101146370	open sea	RP11-566J3.2	DLK1			0.684	0.010	0.005	0.015	3.883	0.000207472	0.423246073	ns	+
150	cg08698523	chr17:33900679	open sea	SNORD7	PEX12	Body		2.040	0.011	0.005	0.016	3.881	0.000209009	0.423246073	ns	+
151	cg15748835	chr16:88654508	open sea	ZC3H18	ZC3H18	Body		3.114	-0.009	-0.014	-0.004	-3.881	0.000209064	0.423246073	ns	-
152	cg20454518	chr12:133135463	island	FBRSL1	FBRSL1	Body	unknown cell specific	-0.042	0.010	0.005	0.016	3.877	0.000212076	0.423246073	ns	+

153	cg15006828	chr20:45081360	open sea	ZNF663P	ELMO2			1.553	0.009	0.005	0.014	3.876	0.000212237	0.423246073	ns	+
154	cg07230786	chr12:113335032	open sea	RPH3A	RPH3A	3'UTR		3.118	0.007	0.003	0.011	3.875	0.000213512	0.423246073	ns	+
155	cg22901652	chr21:42741952	open sea	MX2	MX2	5'UTR		-2.571	-0.021	-0.032	-0.010	-3.874	0.000214482	0.423246073	ns	-
156	cg12306833	chr3:50227389	N shelf		SEMA3F			1.784	-0.009	-0.013	-0.004	-3.868	0.000218531	0.428015206	ns	-
157	cg18764544	chr1:54803684	open sea	SSBP3	SSBP3	Body	unknown cell specific	0.661	-0.014	-0.021	-0.007	-3.867	0.000219697	0.428015206	ns	-
158	cg25485092	chr8:24241360	open sea	ADAMDEC1	ADAMDEC1	TSS1500		2.009	-0.011	-0.017	-0.005	-3.862	0.000223271	0.429175874	ns	-
159	cg15013124	chr11:112223861	open sea	LINC02762	PLET1	Body		2.191	-0.008	-0.012	-0.004	-3.859	0.000225115	0.429175874	ns	-
160	cg01409204	chrX:119738143	open sea	MCTS1	MCTS1	TSS1500		-1.281	-0.007	-0.011	-0.003	-3.858	0.000226253	0.429175874	ns	-
161	cg17258741	chr17:27400787	open sea	MYO18A	MYO18A	3'UTR		1.571	0.011	0.005	0.016	3.857	0.000226814	0.429175874	ns	+
162	cg27521476	chr10:102896376	island	TLX1	TLX1	Body		-1.693	-0.011	-0.017	-0.005	-3.857	0.000227309	0.429175874	ns	-
163	cg18839750	chr5:140723331	N shore	PCDHGA2	PCDHGA2	Body	non-gene-linked	-2.751	-0.015	-0.023	-0.007	-3.853	0.000230139	0.431852834	ns	-
164	cg23832596	chr18:29629031	open sea	RNF125	RNF125	Body		1.090	0.013	0.006	0.020	3.843	0.000237966	0.438657697	ns	+
165	cg20054248	chr12:56414508	open sea	IKZF4	IKZF4	TSS200		-2.468	-0.011	-0.017	-0.005	-3.843	0.000238226	0.438657697	ns	-
166	cg16901566	chr2:1379691	open sea	TPO	TPO			1.257	-0.010	-0.015	-0.005	-3.840	0.000240643	0.438657697	ns	-
167	cg08494641	chr13:26649719	open sea		SHISA2			0.452	0.010	0.005	0.015	3.839	0.000241426	0.438657697	ns	+
168	cg12816088	chr14:73376412	open sea		DPF3			1.780	0.008	0.004	0.012	3.837	0.000243355	0.438657697	ns	+
169	cg13952899	chr1:226739292	S shore	STUM	STUM	Body		2.166	0.010	0.005	0.015	3.837	0.00024347	0.438657697	ns	+
170	cg14392985	chr9:129345450	open sea		LMX1B			1.839	-0.008	-0.013	-0.004	-3.836	0.000243804	0.438657697	ns	-
171	cg16193928	chr1:218329980	open sea		RRP15			-1.902	-0.011	-0.017	-0.006	-3.831	0.000248125	0.443821725	ns	-
172	cg13085507	chr10:75935875	N shore	ADK	ADK	Body	promoter-linked	-0.233	-0.019	-0.028	-0.009	-3.828	0.000251225	0.444680662	ns	-
173	cg02135916	chr14:57363870	open sea	OTX2	OTX2	Body		-1.858	0.015	0.007	0.023	3.827	0.000251513	0.444680662	ns	+
174	cg17177006	chr3:5232410	S shelf	EDEM1	EDEM1	Body	promoter-linked	-1.600	0.009	0.005	0.014	3.825	0.000253227	0.445138534	ns	+
175	cg17138880	chr13:27706117	open sea	USP12	USP12	Body		1.130	-0.011	-0.016	-0.005	-3.821	0.000256944	0.449090714	ns	-
176	cg11685162	chr2:242844876	island	LINC01237	RTP5			2.697	0.019	0.009	0.029	3.816	0.000261887	0.455129251	ns	+
177	cg16367606	chr16:71893832	N shore	ATXN1L	ATXN1L	Body		1.369	0.006	0.003	0.010	3.809	0.000268102	0.459223848	ns	+
178	cg06800558	chr11:29455031	open sea	RP11-460B17.3	KCNA4			2.418	0.007	0.003	0.010	3.808	0.000268877	0.459223848	ns	+
179	cg16806953	chrX:70272769	N shore		SNX12			0.931	0.008	0.004	0.012	3.806	0.00027053	0.459223848	ns	+
180	cg01330060	chr19:47960809	island	SLC8A2	SLC8A2	Body	unknown cell specific	-3.297	-0.011	-0.016	-0.005	-3.804	0.000272535	0.459223848	ns	-
181	cg19211568	chrX:115511218	open sea		SLC6A14			1.920	0.014	0.007	0.021	3.800	0.000276196	0.459223848	ns	+
182	cg24485032	chr7:6456507	open sea	DAGLB	DAGLB	Body		2.008	0.009	0.004	0.014	3.799	0.000276821	0.459223848	ns	+
183	cg15754399	chr4:170928131	open sea	MFAP3L	MFAP3L	5'UTR		0.260	0.007	0.003	0.011	3.798	0.000278167	0.459223848	ns	+
184	cg07539543	chr2:240430981	open sea		HDAC4-AS1			0.486	0.006	0.003	0.010	3.797	0.000278737	0.459223848	ns	+
185	cg12598937	chr10:10987228	open sea	CELF2	CELF2	Body		3.048	0.011	0.005	0.017	3.794	0.000281889	0.459223848	ns	+
186	cg11803375	chr1:55909462	open sea		USP24			2.647	0.017	0.008	0.026	3.793	0.000282804	0.459223848	ns	+
187	cg23416540	chr15:45571777	open sea	LOC101928414	SLC28A2	TSS1500	promoter-linked	-4.070	-0.038	-0.058	-0.018	-3.799	0.000283742	0.459223848	ns	-
188	cg07043360	chr8:126924388	open sea		TRIB1			-1.680	0.011	0.005	0.016	3.792	0.000284255	0.459223848	ns	+
189	cg02224988	chr2:32947733	open sea	TTC27	TTC27	Body		2.946	0.016	0.007	0.024	3.791	0.000284519	0.459223848	ns	+
190	cg07453773	chr11:8779319	open sea	DENND2B	DENND2B	5'UTR		2.931	-0.008	-0.012	-0.004	-3.791	0.000285262	0.459223848	ns	-
191	cg01040553	chr14:101554357	open sea		MEG9			1.100	0.008	0.004	0.012	3.787	0.000288635	0.461043056	ns	+
192	cg23533513	chr11:76849324	island	MYO7A	MYO7A	Body		-0.259	-0.019	-0.029	-0.009	-3.785	0.000290737	0.461043056	ns	-
193	cg09890286	chr17:77088710	open sea	RBFOX3	RBFOX3	Body		2.740	0.013	0.006	0.019	3.784	0.000291548	0.461043056	ns	+
194	cg13786328	chr10:62414644	open sea	ANK3	ANK3	Body		0.354	-0.012	-0.019	-0.006	-3.783	0.000292421	0.461043056	ns	-
195	cg00248938	chr22:32455196	open sea	SLC5A1	SLC5A1	1stExon		1.985	0.013	0.006	0.020	3.779	0.000296601	0.461893827	ns	+
196	cg11968948	chr11:59590619	open sea		CBLIF		unknown cell specific	-3.178	-0.011	-0.017	-0.005	-3.776	0.000300264	0.461893827	ns	-
197	cg02674728	chr22:19758575	S shelf	TBX1	TBX1	Body		2.109	-0.008	-0.012	-0.004	-3.775	0.000301388	0.461893827	ns	-
198	cg17583307	chr7:72765764	open sea	FKBP6	FKBP6	3'UTR		3.404	0.015	0.007	0.023	3.771	0.000304908	0.461893827	ns	+
199	cg21930118	chr5:140705845	open sea	AC005618.6	TAF7		promoter-linked	-3.430	-0.016	-0.024	-0.007	-3.771	0.00030501	0.461893827	ns	-
200	cg00111778	chr2:3833127	N shore	DCDC2C	DCDC2C			1.774	-0.017	-0.025	-0.008	-3.769	0.00030705	0.461893827	ns	-
201	cg18397975	chr5:33997484	open sea	AMACR	AMACR	Body		-0.207	0.011	0.005	0.017	3.769	0.000307153	0.461893827	ns	+
202	cg06160539	chr1:244573354	open sea	ADSS2	ADSS2	Body		1.236	0.011	0.005	0.017	3.768	0.000308446	0.461893827	ns	+
203	cg12116288	chr17:70215735	open sea	SOX9	SOX9			-2.969	-0.011	-0.017	-0.005	-3.767	0.000309376	0.461893827	ns	-
204	cg01140782	chrX:152864413	island	CCNQ	CCNQ	Body	promoter-linked	-0.197	-0.014	-0.021	-0.007	-3.766	0.000310396	0.461893827	ns	-
205	cg03929754	chr5:134481881	open sea	C5orf66	PITX1	5'UTR		2.473	-0.011	-0.017	-0.005	-3.766	0.000310467	0.461893827	ns	-
206	cg07738456	chr1:64309862	open sea	ROR1	ROR1	Body		0.955	0.009	0.004	0.013	3.765	0.000311143	0.461893827	ns	+
207	cg12894711	chr7:43821949	open sea	BLVRA	BLVRA	Body		-2.206	-0.009	-0.014	-0.004	-3.764	0.000312592	0.461893827	ns	-
208	cg18882670	chrX:118357084	island	RP4-555N2.4	PGRMC1			2.872	-0.013	-0.021	-0.006	-3.758	0.000318571	0.468465211	ns	-
209	cg13745832	chr17:15405942	open sea	TVP23C	TVP23C	3'UTR		1.588	0.018	0.009	0.028	3.756	0.0003219	0.470359741	ns	+
210	cg00847114	chr1:6255203	N shore	FAM160A2	FAM160A2	5'UTR		-1.195	-0.007	-0.011	-0.003	-3.754	0.000322935	0.470359741	ns	-
211	cg23958524	chr3:9289787	open sea	SRGAP3	SRGAP3	Body		1.033	0.009	0.004	0.013	3.749	0.000328637	0.474680831	ns	+
212	cg00481227	chr20:36322069	N shore	CTNNBL1	CTNNBL1	TSS1500	promoter-linked	-1.133	-0.012	-0.019	-0.006	-3.748	0.000330022	0.474680831	ns	-
213	cg14529070	chr10:79408312	open sea		KCNMA1			0.325	0.006	0.003	0.010	3.747	0.000331418	0.474680831	ns	+
214	cg12325314	chr6:20204519	open sea	MBOAT1	MBOAT1	Body	unknown cell specific	-0.168	0.008	0.004	0.012	3.743	0.000335477	0.474680831	ns	+
215	cg09106404	chr19:46572305	open sea	IGFL4	IGFL4	Body		1.104	-0.009	-0.013	-0.004	-3.743	0.000335622	0.474680831	ns	-
216	cg17215278	chr1:117114263	S shore	CD58	CD58	TSS1500		-0.599	0.014	0.007	0.022	3.742	0.000336646	0.474680831	ns	+
217	cg21191574	chr18:8859393	open sea		MTCL1			2.013	-0.010	-0.015	-0.005	-3.740	0.000338915	0.474680831	ns	-
218	cg07997529	chr6:142682670	open sea	ADGRG6	ADGRG6	Body		-1.007	-0.010	-0.015	-0.005	-3.739	0.000340146	0.474680831	ns	-
219	cg15524363	chr6:13874679	S shore		RNF182			2.305	-0.010	-0.015	-0.005	-3.738	0.000341124	0.474680831	ns	-
220	cg18839637	chr5:140723684	island	PCDHGA2	PCDHGA2	Body	non-gene-linked	-0.894	-0.011	-0.016	-0.005	-3.738	0.000341421	0.474680831	ns	-
221	cg02654379	chr18:615926	open sea	CLUL1	CLUL1	TSS1500		1.716	-0.009	-0.014	-0.004	-3.734	0.00034636	0.479368836	ns	-
222	cg19281347	chr2:83883578	open sea	RNU6-1312P	SUCLG1			-0.558	-0.018	-0.027	-0.008	-3.733	0.000347969	0.479426181	ns	-
223	cg07058694	chr12:111909145	open sea	ATXN2	ATXN2	Body		0.867	0.011	0.005	0.016	3.731	0.000350063	0.480147751	ns	+
224	cg17292491	chr2:146672431	open sea	AC062031.1	ZEB2			2.113	0.008	0.004	0.012	3.728	0.000352661	0.480864268	ns	+
225	cg24196622	chr2:14756087	open sea	AC011897.2	LRATD1			2.977	0.010	0.005	0.016	3.726	0.000355036	0.480864268	ns	+
226	cg05430886	chr16:68479922	N shore	SMPD3	SMPD3	5'UTR		2.943	-0.012	-0.018	-0.005	-3.725	0.000356795	0.480864268	ns	-
227	cg01464748	chr10:45922877	island	ALOX5	ALOX5	Body		3.272	-0.011	-0.017	-0.005	-3.725	0.000356874	0.480864268	ns	-
228	cg19432802	chr11:113156155	open sea	RP11-839D17.3	NCAM1			2.860	-0.011	-0.017	-0.005	-3.724	0.000358548	0.481001441	ns	-
229	cg08538549	chr3:118905497	open sea	UPK1B	UPK1B	5'UTR		3.106	0.009	0.004	0.014	3.722	0.000360644	0.481700944	ns	+

230	cg26391674	chr20:3148934	island	LZTS3	LZTS3	1stExon	unknown cell specific	-3.498	-0.024	-0.037	-0.011	-3.718	0.000368786	0.484276223	ns	-
231	cg20045970	chr12:110319019	S shore	GLTP	GLTP	TSS1500	promoter-linked	0.100	0.006	0.003	0.009	3.715	0.000368953	0.484276223	ns	+
232	cg19728520	chr13:67094267	open sea	PCDH9	PCDH9	Body		2.460	-0.011	-0.017	-0.005	-3.712	0.000372455	0.484276223	ns	-
233	cg27264384	chr10:134671625	N shore	CFAP46	CFAP46			2.636	-0.008	-0.013	-0.004	-3.712	0.000372947	0.484276223	ns	-
234	cg23490077	chr6:112610645	open sea	LAMA4	LAMA4			2.397	-0.009	-0.013	-0.004	-3.711	0.000374232	0.484276223	ns	-
235	cg23875045	chrX:48595527	island	AF196970.3	GLOD5		promoter-linked	0.560	0.012	0.005	0.018	3.710	0.000374749	0.484276223	ns	+
236	cg09659163	chr12:57584929	open sea	LRP1	LRP1	Body		3.066	-0.011	-0.017	-0.005	-3.709	0.000376034	0.484276223	ns	-
237	cg14511652	chr9:133783802	S shelf	FIBCD1	FIBCD1	Body		-0.258	-0.022	-0.034	-0.010	-3.711	0.000376129	0.484276223	ns	-
238	cg16349662	chr3:186314716	open sea	DNAJB11	DNAJB11			1.966	-0.014	-0.021	-0.006	-3.708	0.000378401	0.484276223	ns	-
239	cg03868882	chr19:12098180	N shore	CTD-2006C1.2	ZNF763			-2.031	-0.012	-0.019	-0.006	-3.708	0.000378405	0.484276223	ns	-
240	cg11620066	chr19:35615579	island	LGI4	LGI4	3'UTR		-3.668	-0.018	-0.028	-0.009	-3.702	0.000385552	0.490006191	ns	-
241	cg05719570	chr3:70904439	open sea		FOXP1			2.335	-0.010	-0.016	-0.005	-3.700	0.000388314	0.490006191	ns	-
242	cg19107511	chr12:52476217	S shelf	SMIM41	SMIM41			0.714	0.010	0.005	0.016	3.698	0.000390572	0.490006191	ns	+
243	cg09894276	chr6:169977394	island	WDR27	WDR27	Body		3.101	-0.028	-0.044	-0.013	-3.702	0.000390854	0.490006191	ns	-
244	cg14248883	chr13:44978677	open sea	TUSC8	SERP2	Body		2.861	0.020	0.009	0.030	3.699	0.000390893	0.490006191	ns	+
245	cg07511671	chr21:36259694	S shore	RUNX1	RUNX1	1stExon		-3.846	0.021	0.010	0.032	3.695	0.000396046	0.493498538	ns	+
246	cg00186699	chr10:21466288	S shelf		NEBL-AS1			2.335	-0.011	-0.017	-0.005	-3.693	0.000397562	0.493498538	ns	-
247	cg09962086	chr2:55260717	open sea	RTN4	RTN4	Body	promoter-linked cell specific	-0.752	0.014	0.006	0.021	3.692	0.000399519	0.493498538	ns	+
248	cg20208483	chr6:11655195	open sea		ADTRP			3.177	0.008	0.004	0.012	3.691	0.000400132	0.493498538	ns	+
249	cg12267069	chr1:156407809	S shore		C1orf61		unknown cell specific	-2.968	-0.012	-0.019	-0.006	-3.688	0.000404664	0.493742969	ns	-
250	cg09102332	chr7:20498337	open sea		ITGB8			0.310	-0.028	-0.044	-0.013	-3.692	0.000404848	0.493742969	ns	-
251	cg21964649	chr12:131572783	island	ADGRD1	ADGRD1	Body		1.812	0.010	0.004	0.015	3.684	0.000409581	0.493742969	ns	+
252	cg12777690	chr14:102171876	N shore	RP11-1029J19.5	PPP2R5C			0.237	0.006	0.003	0.009	3.684	0.000410035	0.493742969	ns	+
253	cg08470157	chrX:30671302	island	GK	GK	TSS200	promoter-linked	-0.968	-0.015	-0.024	-0.007	-3.684	0.000410382	0.493742969	ns	-
254	cg03846767	chr1:3607292	island	TP73	TP73	1stExon		1.245	0.008	0.003	0.012	3.683	0.000410633	0.493742969	ns	+
255	cg08496203	chr2:101044404	open sea		CHST10		unknown cell specific	1.347	0.013	0.006	0.020	3.683	0.00041163	0.493742969	ns	+
256	cg03680291	chr16:47052148	S shelf	RP11-169E6.4	DNAJA2			0.313	0.016	0.007	0.025	3.677	0.000419723	0.501484306	ns	+
257	cg11500952	chr9:135362985	S shore	CFAP77	CFAP77	Body	unknown cell specific	-1.096	0.013	0.006	0.020	3.670	0.000428931	0.501977916	ns	+
258	cg21674799	chrX:39910206	open sea	BCOR	BCOR			2.668	0.011	0.005	0.017	3.669	0.00043028	0.501977916	ns	+
259	cg11242773	chr8:42698994	S shore	THAP1	THAP1	TSS1500	promoter-linked	1.871	0.011	0.005	0.016	3.669	0.000430948	0.501977916	ns	+
260	cg09205359	chr13:32059853	open sea		B3GLCT			1.859	0.018	0.008	0.027	3.669	0.000431	0.501977916	ns	+
261	cg07061119	chr8:26712278	open sea	ADRA1A	ADRA1A	Body		2.741	0.008	0.004	0.013	3.669	0.000431138	0.501977916	ns	+
262	cg07820189	chr1:183149515	open sea		LAMC2			-0.518	0.017	0.008	0.027	3.669	0.000431405	0.501977916	ns	+
263	cg22516975	chr17:1617465	N shore	MIR22HG	WDR81	Body		-2.715	-0.012	-0.018	-0.005	-3.667	0.000433275	0.501977916	ns	-
264	cg07555125	chr5:140175691	island	PCDHA1	PCDHA1	Body		3.007	-0.014	-0.021	-0.006	-3.667	0.00043368	0.501977916	ns	-
265	cg21247178	chr15:48709883	open sea	FBN1	FBN1	Body		3.090	0.013	0.006	0.020	3.665	0.000436282	0.501977916	ns	+
266	cg00780036	chr8:52034593	open sea		PXDNL			2.857	-0.009	-0.014	-0.004	-3.665	0.000436548	0.501977916	ns	-
267	cg02506527	chrX:39868153	island	AC092198.1	BCOR		unknown cell specific	-3.696	-0.018	-0.028	-0.008	-3.664	0.000438351	0.502163408	ns	-
268	cg00613198	chr14:62010028	open sea	PRKCH	PRKCH	Body		1.395	-0.008	-0.012	-0.004	-3.659	0.000445178	0.504666949	ns	-
269	cg03817803	chr21:48058479	S shelf	PRMT2	PRMT2	Body		-0.983	0.007	0.003	0.011	3.657	0.000449048	0.504666949	ns	+
270	cg02122937	chr10:352668	open sea	DIP2C	DIP2C	Body		2.125	0.008	0.004	0.012	3.656	0.000449985	0.504666949	ns	+
271	cg23977670	chr11:2016848	N shore	H19	MRPL23	Body		2.379	-0.009	-0.013	-0.004	-3.655	0.000451126	0.504666949	ns	-
272	cg11849327	chr10:76682695	open sea	KAT6B	KAT6B	Body		1.727	0.009	0.004	0.013	3.654	0.000452975	0.504666949	ns	+
273	cg01499619	chr5:179205250	open sea	MAML1	MAML1			2.340	0.010	0.004	0.015	3.653	0.000455448	0.504666949	ns	+
274	cg14583312	chr21:45161273	open sea	PDXK	PDXK	Body		-2.445	0.039	0.018	0.060	3.659	0.000455701	0.504666949	ns	+
275	cg01386790	chr6:130838699	open sea		TMEM200A		unknown cell specific	2.005	-0.008	-0.012	-0.003	-3.652	0.000456255	0.504666949	ns	-
276	cg02014108	chr4:121387950	open sea		PRDM5			0.788	0.021	0.010	0.033	3.653	0.000456834	0.504666949	ns	+
277	cg07665352	chr10:5135453	open sea	AKR1C3	AKR1C3	TSS1500		2.377	0.008	0.004	0.013	3.651	0.000457036	0.504666949	ns	+
278	cg23351271	chr8:145644123	S shelf		SLC39A4			2.023	-0.007	-0.011	-0.003	-3.650	0.000459923	0.506027345	ns	-
279	cg23087300	chr19:36348415	S shore	KIRREL2	KIRREL2	Body		-0.961	-0.011	-0.017	-0.005	-3.647	0.000464694	0.507748568	ns	-
280	cg13269476	chr20:40619057	open sea	RP5-1121H13.4	PTPRT			-0.285	0.009	0.004	0.013	3.645	0.0004665	0.507748568	ns	+
281	cg22587855	chr1:149215474	open sea	RNVU1-23	FAM72C			-2.794	-0.021	-0.032	-0.009	-3.646	0.000466996	0.507748568	ns	-
282	cg18406472	chrX:101967643	island	GPRASP2	GPRASP2	5'UTR		-0.470	-0.011	-0.017	-0.005	-3.643	0.000469647	0.507748568	ns	-
283	cg13962730	chrX:79492581	open sea	CHMP1B2P	TENT5D	Body		0.148	-0.007	-0.011	-0.003	-3.643	0.000470491	0.507748568	ns	-
284	cg21580428	chr5:140792814	N shore	PCDHGA4	PCDHGA4	Body		-3.017	-0.012	-0.019	-0.006	-3.642	0.000472547	0.507748568	ns	-
285	cg00366917	chr18:74845154	island	MBP	MBP	TSS1500	promoter-linked	-3.364	0.020	0.009	0.030	3.642	0.000473318	0.507748568	ns	+
286	cg06695691	chr4:124154293	open sea	SPATA5	SPATA5	Body		0.653	-0.011	-0.018	-0.005	-3.639	0.000477142	0.507748568	ns	-
287	cg04184094	chr2:219187299	N shore	PNKD	PNKD	TSS1500	promoter-linked	-1.331	-0.009	-0.014	-0.004	-3.638	0.000478527	0.507748568	ns	-
288	cg13873558	chr15:70073380	open sea		PCAT29			-1.150	0.010	0.004	0.015	3.638	0.00047857	0.507748568	ns	+
289	cg05694921	chr1:29587117	island	PTPRU	PTPRU	Body	unknown cell specific	-2.944	-0.018	-0.028	-0.008	-3.636	0.000481064	0.507748568	ns	-
290	cg18266371	chr12:132628355	island	DDX51	DDX51	Body		-1.861	0.013	0.006	0.020	3.634	0.000484534	0.507748568	ns	+
291	cg02661437	chr10:15210818	S shore	NMT2	NMT2	TSS200	promoter-linked	-2.442	-0.011	-0.016	-0.005	-3.634	0.000485143	0.507748568	ns	-
292	cg02939120	chr17:14255156	open sea		HS3ST3B1			1.427	0.027	0.012	0.041	3.637	0.000485292	0.507748568	ns	+
293	cg11380570	chr6:28271503	open sea		PGBD1		unknown cell specific	1.557	0.021	0.009	0.032	3.634	0.000486387	0.507748568	ns	+
294	cg18113094	chr7:91187346	open sea	RP11-142A5.1	MTERF			1.708	-0.010	-0.015	-0.004	-3.631	0.000488992	0.507852199	ns	-
295	cg26636279	chr3:112677997	open sea	CD200R1	CD200R1	Body		-0.762	0.014	0.006	0.021	3.631	0.000489807	0.507852199	ns	+
296	cg13630901	chr11:15224445	open sea	INSC	INSC	Body		2.046	-0.007	-0.011	-0.003	-3.626	0.000497523	0.512747104	ns	-
297	cg08937075	chr2:46541625	open sea	EPAS1	EPAS1	Body		1.930	0.010	0.005	0.015	3.626	0.000498255	0.512747104	ns	+
298	cg05059891	chr1:31289407	open sea	RP5-1125N11.1	SDC3			-0.942	-0.006	-0.010	-0.003	-3.624	0.000500574	0.512747104	ns	-
299	cg24629279	chrX:53344821	open sea	IQSEC2	IQSEC2	Body		2.551	0.010	0.004	0.015	3.622	0.000503499	0.512747104	ns	+
300	cg14353114	chr19:1690915	open sea		TCF3			-3.548	0.022	0.010	0.035	3.624	0.000503945	0.512747104	ns	+
301	cg09076216	chr8:144367072	S shore		ZNF696			3.080	-0.014	-0.022	-0.006	-3.622	0.000504587	0.512747104	ns	-
302	cg22599122	chr1:34329757	open sea	HMGB4	HMGB4	5'UTR		2.257	0.007	0.003	0.011	3.619	0.000508987	0.51422565	ns	+
303	cg26577326	chr18:49063937	open sea	LINC01630	MEX3C	Body		-0.930	-0.014	-0.022	-0.006	-3.619	0.000509522	0.51422565	ns	-
304	cg03889540	chr17:80337659	S shelf		UTS2R		unknown cell specific	0.025	0.012	0.006	0.019	3.618	0.000511085	0.51422565	ns	+
305	cg06924233	chr14:97260744	N shelf		VRK1			2.528	-0.007	-0.011	-0.003	-3.617	0.000512956	0.514415508	ns	-

CVB: CURRENT VIOLENT BEHAVIOUR

rank	cpg	cpg.position	region	annotation	gene	gene.group	gene.feature	AveExpr	logFC	CI.L	CI.R	t	P.Value	adj.P.Val	fdr	sign
1	cg10204397	chr18:77587326	S shore		KCNG2			2.605	0.028	0.019	0.037	6.243	1.78E-08	0.005449811	***	+
2	cg07773368	chr16:70425197	open sea	ST3GAL2	ST3GAL2	Body		1.760	0.020	0.013	0.027	5.819	1.10E-07	0.016836703	**	+
3	cg27191499	chr1:176536552	open sea	PAPPA2	PAPPA2	Body		-3.050	0.047	0.030	0.064	5.548	3.45E-07	0.024408843	**	+
4	cg22522939	chr8:144210636	open sea		LY6H			3.134	0.032	0.021	0.044	5.527	3.74E-07	0.024408843	**	+
5	cg03331514	chr7:1577016	S shore	MAFK	MAFK	5'UTR		2.079	0.021	0.013	0.029	5.431	5.55E-07	0.024408843	**	+
6	cg18921776	chr15:96647029	open sea	LOC105369212	NR2F2			2.752	0.019	0.012	0.026	5.383	6.79E-07	0.024408843	**	+
7	cg25315503	chr21:45247038	S shore		AATBC			1.759	0.015	0.009	0.020	5.377	6.94E-07	0.024408843	**	+
8	cg01201215	chr5:151137430	N shore	ATOX1	ATOX1	Body		1.362	0.021	0.013	0.028	5.369	7.18E-07	0.024408843	**	+
9	cg27493997	chr1:156675392	island	CRABP2	CRABP2	TSS200		-2.779	0.018	0.011	0.025	5.368	7.20E-07	0.024408843	**	+
10	cg18885645	chr7:74057723	open sea		GTF2I			-0.702	0.033	0.020	0.045	5.318	8.86E-07	0.024408843	**	+
11	cg16976865	chr1:24128432	S shore	GALE	GALE	TSS1500		1.034	0.015	0.009	0.020	5.298	9.60E-07	0.024408843	**	+
12	cg19471343	chr3:183631171	open sea		ABCC5			1.948	0.020	0.012	0.027	5.292	9.84E-07	0.024408843	**	+
13	cg01097995	chr17:64959947	N shore	CACNG4	CACNG4	TSS1500		1.303	-0.027	-0.038	-0.017	-5.270	1.08E-06	0.024408843	**	-
14	cg06537005	chr16:758544	S shore	LA16c-380A1.2	FBXL16			1.708	0.022	0.014	0.030	5.261	1.12E-06	0.024408843	**	+
15	cg21441211	chr17:71313862	open sea		CDC42EP4			3.171	0.015	0.010	0.021	5.206	1.39E-06	0.028394789	**	+
16	cg02957147	chr13:106550111	open sea	SNORA25	DAOA			2.215	-0.026	-0.036	-0.016	-5.177	1.57E-06	0.030043632	**	-
17	cg24624586	chr7:139180385	open sea		KLRG2			2.073	0.043	0.027	0.060	5.149	1.77E-06	0.030321317	**	+
18	cg04929006	chr22:50686848	N shelf	HDAC10	HDAC10	Body		1.363	0.013	0.008	0.018	5.136	1.85E-06	0.030321317	**	+
19	cg02431562	chr2:96809970	N shore	DUSP2	DUSP2	Body		2.603	0.020	0.013	0.028	5.132	1.88E-06	0.030321317	**	+
20	cg24986336	chr19:36431977	S shore	LRFN3	LRFN3	Body		2.470	0.020	0.012	0.028	5.100	2.14E-06	0.031490521	**	+
21	cg10009116	chr2:10132241	open sea	GRHL1	GRHL1	Body		2.761	0.023	0.014	0.032	5.089	2.23E-06	0.031490521	**	+
22	cg20200811	chr7:23723014	S shelf	FAM221A	FAM221A	Body		2.562	0.017	0.010	0.023	5.079	2.33E-06	0.031490521	**	+
23	cg27419751	chr20:58778545	open sea	MIR646HG	CDH26	Body		1.069	0.011	0.007	0.015	5.075	2.37E-06	0.031490521	**	+
24	cg06521359	chr1:203767572	S shelf	ZC3H11A	ZC3H11A	5'UTR		1.180	0.015	0.009	0.021	5.055	2.56E-06	0.031980582	**	+
25	cg23658320	chr10:11737222	open sea		ECHDC3			2.605	0.034	0.020	0.047	5.050	2.61E-06	0.031980582	**	+
26	cg09631259	chr1:206229625	open sea	AVPR1B	AVPR1B	Body		1.633	0.018	0.011	0.024	5.036	2.77E-06	0.032545007	**	+
27	cg05129265	chr8:48295993	open sea	SPIDR	SPIDR	Body		1.580	0.021	0.013	0.029	4.999	3.20E-06	0.035124641	**	+
28	cg22341848	chr1:24106359	S shore	PITHD1	PITHD1	Body		1.772	0.018	0.011	0.025	4.993	3.27E-06	0.035124641	**	+
29	cg07553761	chr3:160167977	island	TRIM59	TRIM59	TSS1500	promoter-linked	-1.411	-0.025	-0.036	-0.015	-4.989	3.33E-06	0.035124641	**	-
30	cg18893120	chr8:125741209	island	MTSS1	MTSS1	TSS1500		2.564	0.020	0.012	0.028	4.943	4.00E-06	0.039414977	**	+
31	cg07886365	chr14:21505090	open sea	NDRG2	NDRG2	Body		0.394	0.014	0.008	0.019	4.923	4.33E-06	0.039414977	**	+
32	cg09533699	chr22:22781676	open sea	IGLV5-37	IGLV5-37			2.423	0.020	0.012	0.029	4.922	4.35E-06	0.039414977	**	+
33	cg26902279	chr5:134736598	S shore	H2AFY	H2AFY	TSS1500	unknown cellspecific	2.130	0.025	0.015	0.035	4.920	4.38E-06	0.039414977	**	+
34	cg18245230	chr7:157204586	island	DNAJB6	DNAJB6	Body	unknown cellspecific	1.807	0.015	0.009	0.021	4.892	4.89E-06	0.039414977	**	+
35	cg01371477	chr1:150207822	island	ANP32E	ANP32E	TSS1500		-2.635	0.047	0.028	0.066	4.888	5.01E-06	0.039414977	**	+
36	cg06016850	chr2:173099122	N shore		DLX2-DT		unknown cellspecific	0.693	-0.049	-0.069	-0.029	-4.890	5.02E-06	0.039414977	**	-
37	cg07061387	chr3:112645078	open sea	CD200R1	CD200R1	Body		-1.037	0.046	0.027	0.065	4.887	5.05E-06	0.039414977	**	+
38	cg14390012	chr4:145816012	open sea		HSPD1P5			1.556	0.039	0.023	0.054	4.884	5.05E-06	0.039414977	**	+
39	cg22911917	chr21:46198625	open sea	UBE2G2	UBE2G2	Body		1.238	0.024	0.014	0.034	4.880	5.12E-06	0.039414977	**	+
40	cg19177777	chr19:40175038	open sea	LGALS17A	LGALS14	Body		2.592	0.020	0.012	0.028	4.878	5.15E-06	0.039414977	**	+
41	cg00332269	chr11:46302469	S shelf	CREB3L1	CREB3L1	Body		2.059	0.020	0.012	0.028	4.864	5.46E-06	0.040119619	**	+
42	cg09420399	chr11:69973439	open sea	ANO1	ANO1	Body	unknown cellspecific	1.410	0.016	0.009	0.023	4.861	5.51E-06	0.040119619	**	+
43	cg25692732	chrX:153715426	S shore	UBL4A	UBL4A	TSS1500	promoter-linked	0.605	0.015	0.009	0.021	4.855	5.64E-06	0.040119619	**	+
44	cg20606037	chr4:129709502	open sea		JADE1			2.445	0.018	0.010	0.025	4.848	5.81E-06	0.040288317	**	+
45	cg16520712	chr8:1616450	N shore	DLGAP2	DLGAP2	Body		1.136	0.023	0.014	0.033	4.843	5.93E-06	0.040288317	**	+
46	cg23996325	chr22:38038349	S shelf	SH3BP1	SH3BP1	Body		2.075	0.022	0.013	0.031	4.822	6.44E-06	0.041795577	**	+
47	cg00647178	chr11:72396048	open sea	ARAP1	ARAP1		gene-linked	0.449	0.010	0.006	0.014	4.817	6.54E-06	0.041795577	**	+
48	cg15418980	chr19:50365759	island	PNKP	PNKP	Body	gene-linked	1.954	0.015	0.009	0.022	4.817	6.56E-06	0.041795577	**	+
49	cg20360704	chr4:113739170	open sea	ANK2	ANK2	TSS200	unknown cellspecific	-2.775	0.021	0.012	0.029	4.808	6.79E-06	0.04237936	**	+
50	cg06067169	chr16:89118861	island	CTD-2555A7.2	ACSF3		unknown cellspecific	2.258	0.034	0.020	0.048	4.793	7.19E-06	0.044010729	**	+
51	cg11239358	chr5:171607961	open sea	STK10	STK10	Body		2.365	0.024	0.014	0.033	4.784	7.45E-06	0.044676878	**	+
52	cg27287703	chr16:27468375	open sea		GTF3C1			-1.269	0.029	0.017	0.041	4.744	8.71E-06	0.050226188	*	+
53	cg22664800	chr2:110362333	open sea	SEPTIN10	SEPTIN10	Body		1.101	0.018	0.010	0.025	4.736	8.99E-06	0.050226188	*	+
54	cg23868752	chr2:30548843	open sea		LBH			2.397	0.016	0.009	0.022	4.733	9.08E-06	0.050226188	*	+
55	cg08869376	chr19:10226304	island	EIF3G	EIF3G	Body	gene-linked cellspecific	2.597	0.018	0.010	0.025	4.721	9.51E-06	0.050226188	*	+
56	cg24337786	chr19:39108672	N shore	MAP4K1	MAP4K1	TSS200	promoter-linked	-3.573	0.033	0.019	0.048	4.709	9.96E-06	0.050226188	*	+
57	cg04483267	chr16:70613641	open sea	IL34	IL34	TSS200		1.817	0.016	0.009	0.022	4.704	1.01E-05	0.050226188	*	+
58	cg15420763	chrX:73669880	open sea	SLC16A2	SLC16A2	Body		1.872	0.020	0.012	0.029	4.700	1.03E-05	0.050226188	*	+
59	cg26106178	chr17:38359665	open sea		RAPGEFL1			-0.363	0.034	0.020	0.048	4.699	1.04E-05	0.050226188	*	+
60	cg21624228	chr11:65372226	N shelf	MAP3K11	MAP3K11	Body		1.820	0.013	0.008	0.019	4.699	1.04E-05	0.050226188	*	+
61	cg07888289	chr20:3718325	open sea	HSPA12B	HSPA12B	5'UTR		1.994	0.014	0.008	0.020	4.698	1.04E-05	0.050226188	*	+
62	cg23815031	chr2:128407315	N shore	GPR17	GPR17	5'UTR		0.498	0.012	0.007	0.016	4.695	1.05E-05	0.050226188	*	+
63	cg02283735	chr1:44011587	open sea	PTPRF	PTPRF	Body		0.375	-0.035	-0.049	-0.020	-4.690	1.07E-05	0.050226188	*	-
64	cg13206010	chr8:131130913	open sea	ASAP1	ASAP1	ExonBnd		1.758	-0.021	-0.029	-0.012	-4.682	1.11E-05	0.050226188	*	-
65	cg02199799	chr1:246120866	open sea	SMYD3	SMYD3	Body		3.210	0.022	0.013	0.031	4.682	1.11E-05	0.050226188	*	+
66	cg22186219	chr1:15357571	open sea	KAZN	KAZN	Body		2.315	0.021	0.012	0.029	4.682	1.11E-05	0.050226188	*	+
67	cg03999444	chr7:2963703	open sea	CARD11	CARD11	Body	unknown cellspecific	2.623	0.018	0.011	0.026	4.681	1.11E-05	0.050226188	*	+
68	cg03916756	chr17:79032487	open sea	BAIAP2	BAIAP2	Body	unknown cellspecific	1.930	0.017	0.010	0.024	4.677	1.13E-05	0.050226188	*	+
69	cg17429802	chr15:40647680	N shelf	PHGR1	PHGR1	Body		3.099	0.024	0.014	0.035	4.676	1.13E-05	0.050226188	*	+
70	cg01614426	chr9:137114085	open sea		WDR5			2.024	0.016	0.009	0.022	4.671	1.15E-05	0.050428053	*	+
71	cg02376426	chr10:11825506	open sea		ECHDC3			2.410	0.022	0.013	0.032	4.661	1.20E-05	0.050852383	*	+
72	cg03468041	chr14:75688953	open sea		TMED10			0.533	0.024	0.013	0.034	4.657	1.22E-05	0.050852383	*	+
73	cg02318232	chr5:88599237	open sea	MEF2C	MEF2C			-1.660	-0.025	-0.036	-0.014	-4.656	1.22E-05	0.050852383	*	-
74	cg24741238	chrX:48935215	N shelf	WDR45	WDR45	Body		0.916	0.015	0.009	0.022	4.654	1.23E-05	0.050852383	*	+
75	cg02109988	chr1:234792752	open sea	LOC101927787	IRF2BP2	Body		1.006	0.022	0.013	0.032	4.641	1.30E-05	0.052350405	*	+

76	cg17819085	chr1:235122803	open sea		TOMM20			1.850	0.016	0.009	0.023	4.634	1.33E-05	0.052350405	*	+
77	cg21390852	chr10:126301842	N shore	LHPP	LHPP	Body		2.334	0.015	0.008	0.021	4.634	1.33E-05	0.052350405	*	+
78	cg18153437	chr15:93152738	open sea	RP11-386M24.9	FAM174B			1.653	0.015	0.009	0.022	4.633	1.33E-05	0.052350405	*	+
79	cg02201051	chr8:144252833	open sea		LY6H			2.349	0.018	0.010	0.026	4.627	1.37E-05	0.052742829	*	+
80	cg00406489	chr8:47109229	open sea		ASNSP1			2.413	0.026	0.015	0.037	4.623	1.39E-05	0.052742829	*	+
81	cg06249275	chr11:61691034	open sea		RAB3IL1			2.808	0.017	0.010	0.024	4.621	1.40E-05	0.052742829	*	+
82	cg00469205	chr16:4990919	S shelf	PPL	PPL			-0.893	0.020	0.011	0.029	4.617	1.42E-05	0.052909425	*	+
83	cg01345055	chr4:781041	S shore	CPLX1	CPLX1	Body		0.919	-0.031	-0.045	-0.018	-4.609	1.46E-05	0.053903002	*	-
84	cg23252378	chr11:17620974	open sea	OTOG	OTOG	Body		1.527	0.015	0.008	0.021	4.603	1.50E-05	0.054077163	*	+
85	cg21237806	chr1:144941403	open sea	PDE4DIP	PDE4DIP	Body		2.497	0.021	0.012	0.029	4.602	1.50E-05	0.054077163	*	+
86	cg15120380	chr2:171312686	open sea	MYO3B	MYO3B	Body		2.309	0.014	0.008	0.021	4.599	1.52E-05	0.054077163	*	+
87	cg07657463	chr4:40753247	S shore	NSUN7	NSUN7	Body		2.907	0.017	0.010	0.024	4.595	1.54E-05	0.054140825	*	+
88	cg21800537	chr4:12556866	open sea		ECM1P2			1.828	0.018	0.010	0.026	4.593	1.56E-05	0.054140825	*	+
89	cg06711298	chr2:97530602	island	SEMA4C	SEMA4C	Body		2.834	0.027	0.015	0.038	4.588	1.59E-05	0.054378826	*	+
90	cg27280787	chr2:98349537	N shore	ZAP70	ZAP70	Body	unknown cell specific	2.509	0.017	0.009	0.024	4.584	1.61E-05	0.054378826	*	+
91	cg18772409	chr12:102799874	open sea	IGF1	IGF1	Body		-0.691	0.016	0.009	0.024	4.583	1.62E-05	0.054378826	*	+
92	cg03021892	chrX:48329278	open sea	SLC38A5	SLC38A5	TSS1500		2.322	0.023	0.013	0.033	4.576	1.66E-05	0.055172679	*	+
93	cg10591636	chr3:52559151	S shore	NT5DC2	NT5DC2	Body		2.094	0.020	0.012	0.029	4.566	1.73E-05	0.056184259	*	+
94	cg13228313	chr17:41726469	S shelf	MEOX1	MEOX1	Body		3.050	0.019	0.011	0.027	4.565	1.73E-05	0.056184259	*	+
95	cg01389275	chrX:100611842	open sea	BTK	BTK	Body		1.688	0.021	0.012	0.030	4.560	1.76E-05	0.056184259	*	+
96	cg22666013	chr2:197792289	S shore	PGAP1	PGAP1	TSS1500		-2.624	0.029	0.016	0.042	4.558	1.78E-05	0.056184259	*	+
97	cg20597143	chr20:62327117	N shore	TNFRSF6B	TNFRSF6B	5'UTR		0.829	0.012	0.007	0.018	4.553	1.81E-05	0.056184259	*	+
98	cg10964639	chr16:24043296	open sea	PRKCB	PRKCB	Body		2.853	0.017	0.010	0.024	4.551	1.82E-05	0.056184259	*	+
99	cg05613192	chr2:223912416	open sea		KCNE4			0.680	0.023	0.013	0.033	4.550	1.83E-05	0.056184259	*	+
100	cg15641209	chrX:149011289	open sea	MAGEA8	MAGEA8	ExonBnd		2.682	0.023	0.013	0.033	4.548	1.85E-05	0.056184259	*	+
101	cg07262842	chr1:18391262	open sea	LOC101927876	IGSF21	TSS1500		0.147	0.027	0.015	0.039	4.542	1.89E-05	0.056184259	*	+
102	cg16405454	chr11:113258223	N shore	ANKK1	ANKK1	TSS1500		1.969	0.017	0.009	0.024	4.542	1.89E-05	0.056184259	*	+
103	cg09369954	chr1:32663763	N shelf	TXLNA	TXLNA	3'UTR		2.833	0.017	0.009	0.024	4.539	1.91E-05	0.056184259	*	+
104	cg21062155	chr3:52566695	N shore	NT5DC2	NT5DC2	Body		-2.359	0.021	0.012	0.030	4.539	1.91E-05	0.056184259	*	+
105	cg25927520	chr7:82137880	open sea		CACNA2D1			0.263	-0.026	-0.037	-0.014	-4.535	1.94E-05	0.056567042	*	-
106	cg09411495	chr16:2142770	S shore	PKD1	PKD1	Body		2.082	0.013	0.008	0.019	4.525	2.01E-05	0.058033452	*	+
107	cg12813621	chr14:100806964	open sea	WARS1	WARS1	Body		2.575	0.022	0.012	0.032	4.519	2.06E-05	0.05810346	*	+
108	cg21699381	chr10:105438046	open sea	SH3PXD2A	SH3PXD2A	Body	promoter-linked	-2.381	0.039	0.022	0.057	4.518	2.07E-05	0.05810346	*	+
109	cg22635096	chr21:46550644	N shelf	ADARB1	ADARB1	5'UTR		-2.354	0.038	0.021	0.054	4.517	2.08E-05	0.05810346	*	+
110	cg05725703	chr3:46906118	open sea	MYL3	MYL3	TSS1500		0.999	0.012	0.007	0.017	4.513	2.11E-05	0.05810346	*	+
111	cg00793719	chr14:95477946	open sea		DICER1			0.051	-0.024	-0.034	-0.013	-4.508	2.14E-05	0.05810346	*	-
112	cg13354121	chr18:74178097	open sea	ZNF516	ZNF516	5'UTR		2.890	0.032	0.018	0.046	4.508	2.15E-05	0.05810346	*	+
113	cg02784528	chr7:64342247	open sea	ZNF273	ZNF273			2.348	0.017	0.010	0.025	4.508	2.15E-05	0.05810346	*	+
114	cg10551427	chr18:919568	open sea		ADCYAP1			-1.676	-0.024	-0.035	-0.013	-4.503	2.19E-05	0.058660459	*	-
115	cg05242348	chr12:122237123	N shore	LINC01089	SETD1B	Body		0.512	0.022	0.012	0.031	4.496	2.25E-05	0.058707279	*	+
116	cg10383568	chr11:6589761	N shelf	DNHD1	DNHD1	Body		0.685	0.015	0.009	0.022	4.489	2.30E-05	0.058707279	*	+
117	cg00278227	chr17:46714701	S shore	RP11-357H14.17	HOXB9			1.636	0.015	0.009	0.022	4.489	2.31E-05	0.058707279	*	+
118	cg18560638	chr13:100008200	open sea	UBAC2	UBAC2	Body		1.177	0.015	0.008	0.021	4.488	2.31E-05	0.058707279	*	+
119	cg13943460	chr6:163841335	open sea	QKI	QKI	Body		0.411	0.023	0.013	0.033	4.484	2.35E-05	0.058707279	*	+
120	cg14934702	chr1:150954707	open sea	ANXA9	ANXA9	1stExon		0.988	0.013	0.007	0.019	4.481	2.38E-05	0.058707279	*	+
121	cg09499965	chr7:151220664	S shelf		RHEB			2.727	0.016	0.009	0.023	4.481	2.38E-05	0.058707279	*	+
122	cg24422029	chr10:18549378	open sea	CACNB2	CACNB2	Body		3.355	0.024	0.013	0.035	4.479	2.40E-05	0.058707279	*	+
123	cg14529102	chr17:19568270	open sea	ALDH3A2	ALDH3A2	Body		2.484	0.014	0.008	0.021	4.479	2.40E-05	0.058707279	*	+
124	cg01287788	chr2:86362935	open sea	PTCD3	PTCD3	TSS200		2.378	0.014	0.008	0.021	4.472	2.46E-05	0.058707279	*	+
125	cg08401219	chr7:1468958	open sea	MICALL2	MICALL2		unknown cell specific	2.783	0.018	0.010	0.026	4.469	2.49E-05	0.058707279	*	+
126	cg20184330	chr14:74228393	S shore	MIDEAS	MIDEAS	TSS1500	promoter-linked cell specific	-0.065	0.024	0.013	0.034	4.469	2.49E-05	0.058707279	*	+
127	cg11631070	chr8:143405122	N shelf	TSNARE1	TSNARE1	Body		2.317	0.018	0.010	0.026	4.468	2.49E-05	0.058707279	*	+
128	cg13305922	chr2:165478502	S shore	GRB14	GRB14	TSS200		-1.426	0.018	0.010	0.026	4.464	2.53E-05	0.058707279	*	+
129	cg07048504	chr1:55829278	open sea		GOT2P1			2.141	0.028	0.015	0.040	4.463	2.54E-05	0.058707279	*	+
130	cg26743024	chr17:64960282	N shore	CACNG4	CACNG4	TSS1500		-0.017	-0.028	-0.040	-0.015	-4.463	2.54E-05	0.058707279	*	-
131	cg09861240	chr22:38038956	S shelf	SH3BP1	SH3BP1	Body	promoter-linked	2.328	0.017	0.009	0.024	4.463	2.55E-05	0.058707279	*	+
132	cg19735250	chr17:43318610	island	FMNL1	FMNL1	Body		2.905	0.019	0.011	0.028	4.462	2.55E-05	0.058707279	*	+
133	cg12632264	chr10:97889379	N shore	ZNF518A	ZNF518A	TSS200	non-gene-linked	2.413	0.016	0.009	0.023	4.462	2.55E-05	0.058707279	*	+
134	cg07334644	chr1:14009082	open sea		PRDM2			2.082	0.017	0.009	0.024	4.460	2.58E-05	0.058854083	*	+
135	cg22658332	chr18:36612583	open sea	RNU6-706P	CELF4			2.087	0.015	0.008	0.021	4.451	2.66E-05	0.059832679	*	+
136	cg19302854	chr1:167789397	island	ADCY10	ADCY10	Body		-4.145	0.037	0.020	0.053	4.451	2.67E-05	0.059832679	*	+
137	cg14645027	chr12:52642706	N shore	KRT7	KRT7	3'UTR		1.982	0.017	0.009	0.024	4.449	2.68E-05	0.059832679	*	+
138	cg14913216	chr8:145033104	open sea	PLEC	PLEC	Body	unknown cell specific	2.514	0.022	0.012	0.032	4.447	2.70E-05	0.059910142	*	+
139	cg16756539	chr11:8230951	open sea		LMO1			2.036	-0.019	-0.028	-0.011	-4.441	2.77E-05	0.059936571	*	-
140	cg13636952	chr7:142447956	open sea	TRBC2	TRBC2			-1.853	-0.077	-0.112	-0.043	-4.451	2.78E-05	0.059936571	*	-
141	cg15305471	chr4:6152248	open sea	JAKMIP1	JAKMIP1	5'UTR		2.337	0.017	0.009	0.024	4.439	2.79E-05	0.059936571	*	+
142	cg00219210	chr17:12893648	open sea	ARHGAP44	ARHGAP44	3'UTR		2.765	0.019	0.010	0.027	4.437	2.80E-05	0.059936571	*	+
143	cg01510933	chr14:102088158	open sea		DIO3			2.461	0.021	0.012	0.030	4.436	2.81E-05	0.059936571	*	+
144	cg23337648	chr11:47546192	open sea	CELF1	CELF1	5'UTR	promoter-linked	0.552	0.011	0.006	0.016	4.435	2.82E-05	0.059936571	*	+
145	cg01715799	chr1:109371213	open sea	AKNAD1	AKNAD1	Body		0.716	0.019	0.010	0.027	4.431	2.87E-05	0.060051246	*	+
146	cg11221132	chr22:42540342	S shore	CYP2D7	CYP2D7	Body		2.009	0.012	0.006	0.017	4.431	2.87E-05	0.060051246	*	+
147	cg17023341	chr16:57837309	S shore	KIFC3	KIFC3	TSS1500		1.784	0.014	0.007	0.020	4.429	2.89E-05	0.060051246	*	+
148	cg23463608	chr19:2607757	island	GNG7	GNG7	5'UTR		1.603	0.026	0.015	0.038	4.428	2.91E-05	0.060051246	*	+
149	cg08108828	chr6:42907574	open sea	CNPY3	CNPY3			2.713	0.018	0.010	0.026	4.424	2.94E-05	0.060437226	*	+
150	cg08780735	chr3:66101729	open sea		SLC25A26			1.814	0.016	0.009	0.023	4.421	2.97E-05	0.060646715	*	+
151	cg27507473	chr16:86002958	N shelf		IRF8			-0.546	0.031	0.017	0.045	4.416	3.04E-05	0.061154981	*	+
152	cg07196408	chr20:62373115	N shore	SLC2A4RG	SLC2A4RG	Body	promoter-linked	2.255	0.018	0.010	0.026	4.416	3.04E-05	0.061154981	*	+

153	cg20927864	chr9:101782237	open sea	COL15A1	COL15A1	Body		1.904	0.018	0.010	0.026	4.412	3.08E-05	0.061211478	*	+
154	cg08631141	chr2:169439095	open sea	CERS6	CERS6	TSS1500		2.631	0.035	0.019	0.050	4.412	3.08E-05	0.061211478	*	+
155	cg15093040	chr2:207125019	open sea	GPR1-AS	ZDBF2	Body		0.556	0.017	0.010	0.025	4.398	3.24E-05	0.063195698	*	+
156	cg22471128	chr8:50821904	N shore	SNTG1	SNTG1	TSS1500		-1.641	-0.028	-0.041	-0.015	-4.398	3.25E-05	0.063195698	*	-
157	cg14490718	chr18:46389176	S shelf	CTIF	CTIF	3'UTR		3.011	0.022	0.012	0.032	4.397	3.26E-05	0.063195698	*	+
158	cg09624551	chr3:72128386	open sea	LINC00877	PROK2			-0.015	0.021	0.012	0.031	4.396	3.27E-05	0.063195698	*	+
159	cg14493267	chr9:136451116	island	FAM163B	FAM163B			-3.146	0.020	0.011	0.029	4.395	3.29E-05	0.063195698	*	+
160	cg24739596	chr1:243967381	open sea	AKT3	AKT3	Body		2.865	0.019	0.010	0.027	4.390	3.34E-05	0.063617216	*	+
161	cg06366611	chr22:19547305	open sea		CLDN5			2.477	0.018	0.010	0.026	4.390	3.35E-05	0.063617216	*	+
162	cg07357279	chr17:43318735	island	FMNL1	FMNL1	Body		2.334	0.015	0.008	0.022	4.384	3.42E-05	0.064623527	*	+
163	cg13837763	chrX:49115056	open sea	FOXP3	FOXP3	5'UTR		0.628	0.014	0.008	0.021	4.378	3.50E-05	0.064669468	*	+
164	cg21066876	chr4:95972466	open sea	BMPR1B	BMPR1B	TSS1500		2.596	0.021	0.011	0.030	4.376	3.52E-05	0.064669468	*	+
165	cg13486813	chrX:53106053	open sea	GPR173	GPR173	Body		2.793	0.027	0.015	0.039	4.375	3.54E-05	0.064669468	*	+
166	cg13799097	chr14:105888206	open sea	MTA1	MTA1	Body	unknown cell specific	-0.400	0.013	0.007	0.019	4.374	3.55E-05	0.064669468	*	+
167	cg10895844	chr13:111766437	open sea	ARHGEF7	ARHGEF7	TSS1500		-0.803	0.018	0.010	0.026	4.374	3.55E-05	0.064669468	*	+
168	cg23058239	chr12:54868395	open sea	GTSF1	GTSF1	TSS1500		0.663	0.018	0.010	0.026	4.374	3.55E-05	0.064669468	*	+
169	cg22319611	chr22:19840851	N shore	GNB1L	GNB1L	5'UTR		1.157	0.013	0.007	0.019	4.368	3.62E-05	0.065569245	*	+
170	cg01903503	chr17:40269447	island	KAT2A	KAT2A	Body	gene-linked	1.801	0.012	0.006	0.017	4.364	3.69E-05	0.06630243	*	+
171	cg24319836	chr19:10213465	N shelf	ANGPTL6	ANGPTL6	TSS200	promoter-linked cell specific	0.989	0.017	0.009	0.025	4.362	3.71E-05	0.066328129	*	+
172	cg19726666	chr1:18391290	open sea	LOC101927876	IGSF21	TSS1500		-0.248	0.025	0.013	0.036	4.354	3.83E-05	0.067960104	*	+
173	cg09365002	chr6:33288329	N shore	DAXX	DAXX	Body		2.245	0.030	0.016	0.044	4.349	3.90E-05	0.067960104	*	+
174	cg00925620	chr17:7330143	open sea	SPEM2	SPEM2	Body		2.637	0.022	0.012	0.032	4.349	3.90E-05	0.067960104	*	+
175	cg26238414	chr11:47374558	N shelf	MYBPC3	MYBPC3	TSS1500		1.893	0.011	0.006	0.017	4.346	3.94E-05	0.067960104	*	+
176	cg26662308	chr16:13623179	open sea	U91319.1	SHISA9			1.954	0.017	0.009	0.025	4.345	3.94E-05	0.067960104	*	+
177	cg16913737	chr12:109823985	open sea	MYO1H	MYO1H			1.048	0.026	0.014	0.038	4.340	4.02E-05	0.067960104	*	+
178	cg08001340	chr18:45560436	open sea	ZBTB7C	ZBTB7C	Body		2.473	0.016	0.009	0.024	4.340	4.03E-05	0.067960104	*	+
179	cg18502698	chr15:79936611	open sea		MINAR1			-1.530	0.015	0.008	0.022	4.339	4.04E-05	0.067960104	*	+
180	cg12277306	chrX:10319174	open sea	RP6-1O2.1	MID1			1.192	0.023	0.012	0.033	4.337	4.07E-05	0.067960104	*	+
181	cg24822529	chr5:134582880	open sea	C5orf66	MACROH2A1			2.029	0.018	0.010	0.027	4.337	4.08E-05	0.067960104	*	+
182	cg18697351	chr18:21693242	open sea	TTC39C	TTC39C	TSS200		0.081	0.011	0.006	0.016	4.335	4.09E-05	0.067960104	*	+
183	cg24396496	chr11:1942915	open sea	TNNT3	TNNT3	5'UTR	unknown cell specific	1.541	0.015	0.008	0.023	4.335	4.10E-05	0.067960104	*	+
184	cg15912148	chr17:39976558	open sea	FKBP10	FKBP10	Body		2.540	0.015	0.008	0.022	4.333	4.13E-05	0.067960104	*	+
185	cg16248287	chr10:21804965	N shore	SKIDA1	SKIDA1	Body	unknown cell specific	-1.208	0.010	0.006	0.015	4.332	4.14E-05	0.067960104	*	+
186	cg05365216	chr11:95861033	open sea	MAML2	MAML2	Body		-0.988	-0.022	-0.032	-0.012	-4.331	4.16E-05	0.067960104	*	-
187	cg12646006	chr13:113103842	N shelf		SPACA7			-0.137	0.010	0.005	0.015	4.331	4.16E-05	0.067960104	*	+
188	cg07069828	chr8:145565138	S shelf		SCRT1			2.774	-0.020	-0.030	-0.011	-4.329	4.20E-05	0.067960104	*	-
189	cg13054419	chr13:31438929	open sea		MEDAG			2.448	0.019	0.010	0.027	4.327	4.23E-05	0.067960104	*	+
190	cg19121684	chr12:120652844	S shore	PXN	PXN	Body		2.577	0.018	0.009	0.026	4.325	4.25E-05	0.067960104	*	+
191	cg00925516	chr6:39234947	open sea		KCNK17			2.800	0.023	0.013	0.034	4.325	4.26E-05	0.067960104	*	+
192	cg06189670	chr12:131300783	N shelf	STX2	STX2	Body		3.158	0.027	0.014	0.039	4.324	4.27E-05	0.067960104	*	+
193	cg09641390	chr14:91711520	open sea	GPR68	GPR68	TSS1500		1.112	0.015	0.008	0.022	4.321	4.32E-05	0.068310789	*	+
194	cg27235877	chrX:41524702	open sea	CASK	CASK	ExonBnd		1.201	0.018	0.010	0.027	4.316	4.39E-05	0.068310789	*	+
195	cg24804060	chr17:48425014	S shore	XYLT2	XYLT2	Body	promoter-linked cell specific	1.937	0.016	0.009	0.024	4.315	4.41E-05	0.068310789	*	+
196	cg02864756	chr11:69295162	open sea	AP000439.3	CCND1			2.136	0.014	0.007	0.020	4.312	4.47E-05	0.068310789	*	+
197	cg25925210	chr2:219576383	S shore	TTLL4	TTLL4	5'UTR	promoter-linked	-2.188	0.032	0.017	0.047	4.309	4.52E-05	0.068310789	*	+
198	cg03468072	chr12:39539422	island		KIF21A			-2.315	0.025	0.013	0.036	4.308	4.53E-05	0.068310789	*	+
199	cg17082549	chr3:128338853	S shore	RPN1	RPN1	3'UTR		1.944	0.026	0.014	0.039	4.307	4.54E-05	0.068310789	*	+
200	cg05330708	chr16:1077597	island		SSTR5-AS1		unknown cell specific	2.139	0.014	0.007	0.020	4.306	4.56E-05	0.068310789	*	+
201	cg11097968	chr14:103012188	S shore	LINC02323	ANKRD9			0.201	0.015	0.008	0.021	4.305	4.58E-05	0.068310789	*	+
202	cg24251448	chr8:141577100	open sea	ago.02	ago.02	Body		3.137	0.025	0.014	0.037	4.304	4.60E-05	0.068310789	*	+
203	cg02097498	chr16:10965851	open sea	RP11-876N24.2	CIITA			0.848	0.016	0.009	0.024	4.302	4.63E-05	0.068310789	*	+
204	cg13232468	chr3:42586459	open sea		SEC22C		unknown cell specific	1.108	0.014	0.007	0.020	4.302	4.64E-05	0.068310789	*	+
205	cg07341780	chr3:184059879	S shelf	FAM131A	FAM131A	Body		1.869	0.013	0.007	0.019	4.301	4.64E-05	0.068310789	*	+
206	cg00081714	chr5:116306180	open sea		SEMA6A			2.604	0.015	0.008	0.022	4.300	4.66E-05	0.068310789	*	+
207	cg20404850	chr9:99374660	open sea	CDC14B	CDC14B	Body		0.164	0.041	0.022	0.061	4.303	4.67E-05	0.068310789	*	+
208	cg14505439	chr9:134605210	open sea	RAPGEF1	RAPGEF1	Body	promoter-linked	1.461	0.012	0.006	0.018	4.300	4.67E-05	0.068310789	*	+
209	cg01309870	chr6:28584035	island	ZBED9	ZBED9			-1.239	-0.018	-0.027	-0.010	-4.299	4.68E-05	0.068310789	*	-
210	cg23183988	chrX:19908877	S shelf		SH3KBP1			0.883	0.016	0.009	0.023	4.299	4.69E-05	0.068310789	*	+
211	cg15846506	chr10:110612108	open sea		MAPKAPK5P1			2.678	0.051	0.027	0.074	4.299	4.77E-05	0.06911979	*	+
212	cg04320316	chr17:41712200	open sea		MEOX1			1.651	0.012	0.007	0.018	4.290	4.85E-05	0.06994195	*	+
213	cg19370684	chr1:1982688	island	PRKCZ	PRKCZ	Body		-3.158	0.027	0.015	0.040	4.284	4.95E-05	0.071012865	*	+
214	cg24686918	chr1:221613658	open sea		DUSP10			1.961	0.020	0.011	0.029	4.282	4.98E-05	0.071012865	*	+
215	cg22083633	chr5:4143214	open sea	CTD-2008N3.1	IRX1			2.078	-0.019	-0.028	-0.010	-4.282	4.99E-05	0.071012865	*	-
216	cg25069361	chr5:137779871	open sea	REEP2	REEP2	Body		2.343	0.014	0.008	0.021	4.278	5.06E-05	0.07136397	*	+
217	cg16551240	chr10:134690534	N shelf	CFAP46	CFAP46			3.032	0.019	0.010	0.029	4.278	5.07E-05	0.07136397	*	+
218	cg12354480	chr15:99572785	open sea	LUNAR1	PGPEP1L	Body		3.516	0.018	0.010	0.027	4.276	5.09E-05	0.07136397	*	+
219	cg17217296	chr1:159046937	open sea	AIM2	AIM2	TSS1500	promoter-linked	2.595	0.052	0.028	0.077	4.277	5.16E-05	0.07136397	*	+
220	cg13273219	chr2:177628677	open sea	AC092162.1	MTX2			2.068	0.016	0.008	0.023	4.270	5.21E-05	0.07136397	*	+
221	cg19990527	chr13:40450014	open sea		AZU1P1			0.890	0.019	0.010	0.027	4.269	5.23E-05	0.07136397	*	+
222	cg17361881	chr2:100623422	open sea	AFF3	AFF3	Body		2.875	0.015	0.008	0.022	4.269	5.23E-05	0.07136397	*	+
223	cg07941108	chr14:90741814	open sea	NRDE2	NRDE2			0.875	0.020	0.010	0.029	4.268	5.24E-05	0.07136397	*	+
224	cg25043021	chr11:763133	open sea	TALDO1	TALDO1	Body		0.618	0.012	0.006	0.017	4.267	5.26E-05	0.07136397	*	+
225	cg10140974	chr22:47643698	open sea		TBC1D22A			2.132	0.016	0.009	0.024	4.267	5.26E-05	0.07136397	*	+
226	cg05874348	chr17:48348086	N shelf	TMEM92	TMEM92	TSS1500		1.696	0.011	0.006	0.016	4.267	5.27E-05	0.07136397	*	+
227	cg08448284	chr3:130652221	open sea	ATP2C1	ATP2C1	Body		3.435	0.020	0.011	0.029	4.260	5.40E-05	0.072772688	*	+
228	cg24871821	chr1:6398680	open sea	ACOT7	ACOT7	Body		2.762	0.016	0.009	0.024	4.258	5.44E-05	0.073003609	*	+
229	cg23204757	chr21:18376383	open sea		CXADR			-0.981	0.072	0.038	0.105	4.261	5.59E-05	0.074683876	*	+

230	cg19311918	chr9:140306073	open sea	EXD3	EXD3	Body	unknown cell specific	-0.829	0.032	0.017	0.047	4.246	5.68E-05	0.075067709	*	+
231	cg09687417	chr11:63765561	N shore	OTUB1	OTUB1	Body	gene-linked	1.919	0.014	0.008	0.021	4.246	5.69E-05	0.075067709	*	+
232	cg22471647	chrX:25012039	open sea	POLA1	POLA1	Body		1.793	0.018	0.010	0.027	4.245	5.71E-05	0.075067709	*	+
233	cg06962642	chr14:35675373	open sea	PRORP	PRORP	Body		2.394	0.014	0.008	0.021	4.245	5.72E-05	0.075067709	*	+
234	cg12173521	chr13:113164993	open sea	TUBGCP3	TUBGCP3	Body		2.362	0.015	0.008	0.023	4.239	5.84E-05	0.076172606	*	+
235	cg15644970	chr1:3322363	S shore	PRDM16	PRDM16	Body	unknown cell specific	1.701	0.016	0.008	0.023	4.238	5.86E-05	0.076172606	*	+
236	cg05981016	chr15:55653321	open sea	CCPG1	CCPG1	Body		-0.155	0.027	0.014	0.040	4.237	5.88E-05	0.076172606	*	+
237	cg01857718	chr2:121304074	open sea		GLI2			2.572	0.026	0.014	0.038	4.234	5.95E-05	0.07680057	*	+
238	cg16110788	chr7:22602133	open sea	AC002480.3	STEAP1B			2.130	0.015	0.008	0.022	4.231	6.00E-05	0.07697566	*	+
239	cg19379721	chr19:19737143	N shore	LPAR2	LPAR2	Body	unknown cell specific	0.814	0.012	0.006	0.017	4.230	6.02E-05	0.07697566	*	+
240	cg23875350	chr8:144153190	N shore		LY6L			2.387	0.032	0.017	0.047	4.230	6.04E-05	0.07697566	*	+
241	cg01523763	chrX:129068084	S shore	RP4-537K23.4	UTP14A			1.420	0.016	0.008	0.023	4.228	6.07E-05	0.077048013	*	+
242	cg07591863	chr14:105235522	N shelf	RP11-982M15.2	AKT1		gene-linked	0.678	0.012	0.006	0.018	4.225	6.14E-05	0.077449592	*	+
243	cg25471520	chr2:166813804	S shelf	AC010127.3	TTC21B			3.040	0.018	0.010	0.027	4.224	6.16E-05	0.077449592	*	+
244	cg25587223	chr17:7905928	island	GUCY2D	GUCY2D	TSS200	unknown cell specific	-4.194	0.028	0.015	0.040	4.223	6.18E-05	0.077449592	*	+
245	cg14897803	chr15:98855084	open sea		FAM169B			-1.162	0.023	0.012	0.033	4.220	6.26E-05	0.078210787	*	+
246	cg21663100	chrX:70475282	S shore	ZMYM3	ZMYM3	TSS1500	promoter-linked	-1.108	0.017	0.009	0.026	4.217	6.33E-05	0.078705506	*	+
247	cg16966261	chr15:47874599	open sea	SEMA6D	SEMA6D			2.536	0.016	0.009	0.024	4.213	6.41E-05	0.079157404	*	+
248	cg20821276	chr17:65243119	S shore	HELZ	HELZ			0.240	0.010	0.005	0.014	4.213	6.42E-05	0.079157404	*	+
249	cg10425426	chrX:133733109	open sea	PLAC1	PLAC1	5'UTR		1.737	0.025	0.013	0.037	4.210	6.48E-05	0.079317929	*	+
250	cg19774218	chr20:44598991	N shore	ZNF335	ZNF335	Body	promoter-linked	2.176	0.015	0.008	0.022	4.210	6.49E-05	0.079317929	*	+
251	cg17160660	chr8:128746896	N shore	MYC	MYC	TSS1500		-2.669	0.034	0.018	0.051	4.209	6.51E-05	0.079317929	*	+
252	cg06943635	chr14:73695341	open sea		PSEN1			1.249	0.014	0.008	0.021	4.206	6.57E-05	0.079639531	*	+
253	cg01548742	chr16:3311857	N shelf		ZNF263			2.544	0.023	0.012	0.034	4.206	6.59E-05	0.079639531	*	+
254	cg02264935	chr16:66551909	open sea	TK2	TK2	Body		2.888	0.015	0.008	0.022	4.203	6.65E-05	0.079850551	*	+
255	cg05072719	chr20:61299592	open sea	SLCO4A1	SLCO4A1	Body	gene-linked	1.535	0.012	0.006	0.018	4.201	6.69E-05	0.079850551	*	+
256	cg05509871	chr8:28479326	N shore	EXTL3	EXTL3			0.505	0.019	0.010	0.028	4.200	6.72E-05	0.079850551	*	+
257	cg05902191	chr7:77084871	open sea		GSAP			2.551	0.021	0.011	0.030	4.200	6.74E-05	0.079850551	*	+
258	cg03126987	chr2:8800129	open sea		ID2-AS1			1.846	0.013	0.007	0.019	4.199	6.74E-05	0.079850551	*	+
259	cg04212679	chr6:82586524	open sea	LINC02542	TENT5A			2.644	0.015	0.008	0.022	4.198	6.78E-05	0.079850551	*	+
260	cg24764255	chr14:105939232	N shore	CRIP2	CRIP2	TSS200		2.578	0.016	0.008	0.023	4.196	6.82E-05	0.079850551	*	+
261	cg27143695	chr17:81015507	N shore		B3GNTL1			2.422	0.019	0.010	0.028	4.195	6.84E-05	0.079850551	*	+
262	cg21859527	chr21:30518297	open sea	MAP3K7CL	MAP3K7CL	5'UTR		1.502	0.021	0.011	0.031	4.194	6.87E-05	0.079850551	*	+
263	cg03553488	chr19:11480469	open sea	CTD-2342J14.6	PLPPR2			2.486	0.016	0.009	0.024	4.194	6.88E-05	0.079850551	*	+
264	cg25579477	chr7:151496334	open sea	PRKAG2	PRKAG2	5'UTR		1.205	0.013	0.007	0.020	4.192	6.92E-05	0.079850551	*	+
265	cg10746244	chr22:45044961	open sea		ANP32BP2			1.947	0.017	0.009	0.024	4.192	6.92E-05	0.079850551	*	+
266	cg02414922	chr9:139249212	open sea	GPSM1	GPSM1	TSS200		1.620	0.010	0.005	0.015	4.190	6.97E-05	0.079895643	*	+
267	cg16048837	chr13:29840956	open sea	MTUS2	MTUS2	Body		2.071	0.027	0.014	0.040	4.190	6.97E-05	0.079895643	*	+
268	cg07298985	chr8:22133076	island	PIWIL2	PIWIL2	5'UTR		2.516	-0.034	-0.050	-0.018	-4.178	7.31E-05	0.083420138	*	-
269	cg21515402	chr15:89991455	open sea		RHCG			1.980	0.020	0.011	0.030	4.172	7.44E-05	0.084548884	*	+
270	cg02402539	chr19:15399648	open sea	BRD4	BRD4			-0.635	0.015	0.008	0.021	4.168	7.56E-05	0.08544027	*	+
271	cg08810454	chr10:126392745	open sea	FAM53B	FAM53B	Body	gene-linked cell specific	2.454	0.014	0.007	0.021	4.167	7.57E-05	0.08544027	*	+
272	cg20072359	chr17:77147272	open sea	RBFOX3	RBFOX3	5'UTR		2.244	0.021	0.011	0.030	4.164	7.65E-05	0.086019263	*	+
273	cg01649837	chr19:16830643	open sea	NWD1	NWD1	TSS200		0.455	0.020	0.011	0.030	4.161	7.74E-05	0.086473406	*	+
274	cg07005351	chr10:80783093	open sea	ZMIZ1	ZMIZ1	Body		1.702	0.017	0.009	0.024	4.161	7.75E-05	0.086473406	*	+
275	cg06757038	chr15:40759027	open sea	BAHD1	BAHD1	3'UTR	unknown cell specific	2.556	0.016	0.008	0.024	4.157	7.86E-05	0.086738047	*	+
276	cg25414605	chr11:67182672	N shore	CARNS1	CARNS1	TSS1500	unknown cell specific	-0.750	0.016	0.008	0.023	4.157	7.87E-05	0.086738047	*	+
277	cg01505705	chr11:86384846	S shore	ME3	ME3	TSS1500		3.786	0.026	0.014	0.038	4.157	7.87E-05	0.086738047	*	+
278	cg22939000	chr2:71673632	open sea		DYSF			2.186	0.017	0.009	0.026	4.156	7.90E-05	0.086738047	*	+
279	cg22596973	chr11:72902395	open sea		P2RY2			1.819	0.017	0.009	0.025	4.154	7.93E-05	0.086738047	*	+
280	cg00853151	chr6:114150853	open sea	RP1-249H1.2	MARCKS			0.201	0.056	0.029	0.083	4.161	7.94E-05	0.086738047	*	+
281	cg19328228	chr19:40146583	open sea	LGALS16	LGALS16	5'UTR		0.742	0.022	0.011	0.032	4.149	8.08E-05	0.087030996	*	+
282	cg00103774	chr8:142673615	open sea		MROH5			1.931	0.014	0.007	0.021	4.147	8.14E-05	0.087030996	*	+
283	cg12349472	chr15:34650784	open sea	LPCAT4	LPCAT4			1.538	0.011	0.006	0.017	4.147	8.16E-05	0.087030996	*	+
284	cg12996637	chr1:44070613	N shore	PTPRF	PTPRF	Body		1.164	0.010	0.005	0.015	4.146	8.17E-05	0.087030996	*	+
285	cg24733260	chr7:46692046	open sea		HMGN1P19			1.963	0.013	0.007	0.020	4.145	8.21E-05	0.087030996	*	+
286	cg16466201	chr9:140380000	open sea	PNPLA7	PNPLA7	Body		2.632	0.020	0.010	0.030	4.144	8.24E-05	0.087030996	*	+
287	cg08118241	chr8:63160127	N shore	NKAIN3	NKAIN3	TSS1500		-1.264	0.022	0.011	0.032	4.142	8.29E-05	0.087030996	*	+
288	cg02256089	chr3:125800871	N shore	SLC41A3	SLC41A3	5'UTR		1.037	0.009	0.005	0.014	4.142	8.30E-05	0.087030996	*	+
289	cg15170445	chr5:102676936	open sea		MACIR			-0.178	0.034	0.017	0.050	4.142	8.32E-05	0.087030996	*	+
290	cg22357078	chr19:10348903	open sea		S1PR2			1.643	0.016	0.008	0.024	4.139	8.39E-05	0.087030996	*	+
291	cg24391385	chr2:65526509	open sea		SPRED2			0.731	0.013	0.007	0.019	4.137	8.46E-05	0.087030996	*	+
292	cg19091893	chr2:223906444	open sea		KCNE4			1.333	0.034	0.018	0.050	4.137	8.47E-05	0.087030996	*	+
293	cg06694435	chr13:25788013	open sea		MTMR6			2.052	0.018	0.009	0.027	4.134	8.53E-05	0.087030996	*	+
294	cg09144398	chr11:118550485	open sea	TREH	TREH	TSS200		1.858	0.012	0.006	0.017	4.134	8.53E-05	0.087030996	*	+
295	cg19909717	chr6:107924415	open sea	SOBP	SOBP	Body		2.625	0.014	0.007	0.021	4.134	8.53E-05	0.087030996	*	+
296	cg15868443	chr11:4002513	open sea	STIM1	STIM1	Body		2.123	0.019	0.010	0.027	4.133	8.56E-05	0.087030996	*	+
297	cg25058019	chr14:77542701	open sea	LOC102724190	CIPC	TSS200		-1.179	-0.028	-0.041	-0.014	-4.133	8.57E-05	0.087030996	*	-
298	cg03718241	chr6:3623988	open sea	RP1-223B1.1	PXDC1			-1.509	0.018	0.010	0.027	4.132	8.59E-05	0.087030996	*	+
299	cg03698343	chr9:139921892	N shore	ABCA2	ABCA2	Body	promoter-linked cell specific	-0.354	0.009	0.005	0.013	4.131	8.63E-05	0.087030996	*	+
300	cg23904570	chr11:67811721	open sea	TCIRG1	TCIRG1	TSS1500	gene-linked cell specific	2.325	0.014	0.007	0.021	4.131	8.64E-05	0.087030996	*	+
301	cg16350622	chr3:132631089	open sea		NPHP3			0.737	0.032	0.017	0.048	4.131	8.65E-05	0.087030996	*	+
302	cg05697866	chr1:2437200	S shore		PLCH2			2.057	0.028	0.014	0.041	4.128	8.71E-05	0.087030996	*	+
303	cg03758021	chr20:62208140	N shore		HELZ2			2.233	0.018	0.009	0.026	4.126	8.77E-05	0.087030996	*	+
304	cg22881573	chr3:49638532	open sea	BSN	BSN	Body		3.005	0.016	0.008	0.024	4.126	8.78E-05	0.087030996	*	+
305	cg15429959	chr1:233927450	open sea		SLC35F3			2.409	0.019	0.010	0.028	4.126	8.78E-05	0.087030996	*	+

AAGS: FEELINGS OF GUILT

rank	cpg	cpg.position	region	annotation	gene	gene.group	gene.feature	AveExpr	logFC	CI.L	CI.R	t	P.Value	adj.P.Val	fdr	sign
1	cg20866785	chr4:148733880	open sea	ARHGAP10	ARHGAP10	Body		2.683	0.052	0.036	0.068	6.643	3.23E-09	0.000987992	***	+
2	cg09618380	chr4:123839727	N shelf	NUDT6	NUDT6	Body		1.600	-0.047	-0.064	-0.030	-5.515	4.02E-07	0.061500196	*	-
3	cg18770231	chr1:102119095	open sea		OLFM3			-2.060	0.062	0.039	0.085	5.313	9.22E-07	0.078745135	*	+
4	cg15363887	chr11:57414557	island	YPEL4	YPEL4	Body		-2.877	0.049	0.031	0.067	5.286	1.03E-06	0.078745135	*	+
5	cg12608633	chr10:101468959	open sea	ENTPD7	ENTPD7	3'UTR		0.239	0.044	0.027	0.061	5.196	1.48E-06	0.0904457	*	+
6	cg13055772	chr2:134349024	open sea		NCKAP5			-2.717	-0.047	-0.066	-0.029	-5.048	2.68E-06	0.136373661	ns	-
7	cg06470943	chr1:40839093	N shore	SMAP2	SMAP2	TSS1500		-1.240	-0.037	-0.052	-0.022	-4.840	6.07E-06	0.249506031	ns	-
8	cg07298547	chr16:85585356	N shore		GSE1		unknown cellspecific	2.090	0.033	0.019	0.047	4.818	6.64E-06	0.249506031	ns	+
9	cg15623260	chr9:140392492	N shelf	PNPLA7	PNPLA7	Body		2.856	0.043	0.025	0.061	4.792	7.34E-06	0.249506031	ns	+
10	cg02860470	chr6:75649222	open sea		COL12A1			0.303	-0.040	-0.056	-0.023	-4.753	8.54E-06	0.261340687	ns	-
11	cg13994408	chrX:151307730	open sea	MAGEA10	MAGEA10	TSS1500		1.626	-0.032	-0.045	-0.018	-4.704	1.03E-05	0.286899999	ns	-
12	cg25797454	chr6:150327115	S shore	RAET1K	RAET1L	TSS1500	promoter-linked	1.972	0.059	0.034	0.084	4.668	1.19E-05	0.302069872	ns	+
13	cg10996596	chr10:102998762	S shelf	LBX1	LBX1			-3.694	-0.055	-0.079	-0.031	-4.627	1.39E-05	0.326152808	ns	-
14	cg14118991	chr17:26740915	open sea		SLC46A1			1.645	0.042	0.023	0.060	4.577	1.68E-05	0.354617995	ns	+
15	cg16883805	chr17:52946	N shelf		RPH3AL			2.913	0.046	0.026	0.066	4.550	1.86E-05	0.354617995	ns	+
16	cg06192238	chr22:37633583	open sea	RAC2	RAC2	Body		2.610	-0.038	-0.055	-0.021	-4.542	1.91E-05	0.354617995	ns	-
17	cg24170700	chr11:72295438	island	PDE2A	PDE2A	Body		-2.245	-0.041	-0.059	-0.023	-4.534	1.97E-05	0.354617995	ns	-
18	cg26969486	chr4:24982066	open sea	CCDC149	CCDC149	TSS1500		-2.220	0.059	0.033	0.085	4.502	2.23E-05	0.378257956	ns	+
19	cg16361396	chrX:150069224	S shore		CD99L2			2.847	0.031	0.017	0.045	4.476	2.45E-05	0.393041322	ns	+
20	cg00508023	chr14:74296957	open sea	RP5-1021I20.2	PTGR2		unknown cellspecific	-0.631	0.050	0.028	0.073	4.452	2.68E-05	0.393041322	ns	+
21	cg01212329	chr12:104965274	open sea	CHST11	CHST11	Body		2.441	0.022	0.012	0.031	4.439	2.81E-05	0.393041322	ns	+
22	cg22813648	chr12:988989	open sea	WNK1	WNK1	Body		2.584	0.030	0.017	0.044	4.438	2.83E-05	0.393041322	ns	+
23	cg25144039	chr10:18597677	open sea	CACNB2	CACNB2	Body		1.264	0.027	0.015	0.039	4.402	3.23E-05	0.430157268	ns	+
24	cg09959420	chr10:123495675	open sea	RP11-78A18.2	ATE1			-1.290	-0.037	-0.054	-0.020	-4.364	3.72E-05	0.445670835	ns	-
25	cg22103164	chr4:155702409	island	RBM46	RBM46	TSS200		1.130	0.045	0.024	0.065	4.357	3.83E-05	0.445670835	ns	+
26	cg04913132	chr5:153418977	S shore	MFAP3	MFAP3	5'UTR		-2.342	-0.024	-0.035	-0.013	-4.349	3.93E-05	0.445670835	ns	-
27	cg23895495	chr7:47539837	open sea	TNS3	TNS3	5'UTR		0.083	0.039	0.021	0.057	4.346	3.98E-05	0.445670835	ns	+
28	cg01483219	chr16:89075851	open sea		CBFA2T3		unknown cellspecific	2.351	0.060	0.032	0.087	4.328	4.25E-05	0.445670835	ns	+
29	cg08596773	chr12:119510333	open sea	SRRM4	SRRM4	Body		1.563	0.033	0.018	0.048	4.321	4.37E-05	0.445670835	ns	+
30	cg09550624	chr9:100935192	open sea	CORO2A	CORO2A	TSS200		1.988	0.048	0.026	0.070	4.321	4.37E-05	0.445670835	ns	+
31	cg10909179	chrX:71934336	S shore	PHKA1	PHKA1	TSS1500		-1.877	-0.039	-0.057	-0.021	-4.276	5.15E-05	0.46734164	ns	-
32	cg24948887	chr18:74859463	open sea		MBP			0.417	0.052	0.028	0.076	4.275	5.17E-05	0.46734164	ns	+
33	cg07779313	chr2:192731534	open sea	AC098617.1	CAVIN2			1.404	0.029	0.016	0.043	4.275	5.17E-05	0.46734164	ns	+
34	cg22599539	chr20:54089465	open sea	LINC01440	CBLN4			1.662	0.039	0.021	0.057	4.266	5.35E-05	0.46734164	ns	+
35	cg14966336	chr5:137464502	open sea	NME5	NME5	Body		-1.055	-0.038	-0.056	-0.020	-4.258	5.50E-05	0.46734164	ns	-
36	cg06611788	chr5:137495598	open sea	BRD8	BRD8	Body		2.751	0.029	0.015	0.043	4.258	5.50E-05	0.46734164	ns	+
37	cg07855319	chr16:69123283	open sea		TANGO6			-2.381	-0.024	-0.035	-0.013	-4.243	5.81E-05	0.480695114	ns	-
38	cg27196467	chr4:21305490	open sea	KCNIP4	KCNIP4	Body		-1.641	0.044	0.023	0.065	4.226	6.18E-05	0.485547786	ns	+
39	cg13840922	chr7:98258974	open sea	NPTX2	NPTX2	3'UTR		0.848	0.073	0.038	0.107	4.223	6.30E-05	0.485547786	ns	+
40	cg11212234	chr5:74353351	S shelf		GCNT4		unknown cellspecific	0.965	0.041	0.022	0.060	4.214	6.46E-05	0.485547786	ns	+
41	cg20930968	chr1:8611868	open sea	RERE	RERE	Body		0.792	-0.034	-0.050	-0.018	-4.205	6.66E-05	0.485547786	ns	-
42	cg19392133	chr5:1443434	N shore	SLC6A3	SLC6A3	5'UTR		2.598	0.036	0.019	0.053	4.205	6.67E-05	0.485547786	ns	+
43	cg13044268	chr11:59539527	open sea	STX3	STX3	Body		2.421	0.027	0.014	0.040	4.156	7.97E-05	0.566777575	ns	+
44	cg23582054	chr3:3152201	open sea	IL5RA	IL5RA	TSS200		2.932	0.024	0.013	0.036	4.146	8.25E-05	0.573526284	ns	+
45	cg17771238	chr3:97633644	open sea	CRYBG3	CRYBG3	Body		1.452	-0.027	-0.039	-0.014	-4.135	8.58E-05	0.583154994	ns	-
46	cg05607423	chr20:13864214	open sea	SEL1L2	SEL1L2	Body		1.214	0.039	0.020	0.057	4.127	8.85E-05	0.584403788	ns	+
47	cg20308540	chr1:242612849	open sea	PLD5	PLD5	5'UTR		0.187	0.023	0.012	0.034	4.123	8.98E-05	0.584403788	ns	+
48	cg14171882	chr7:48019441	S shore	HUS1	HUS1	TSS200	promoter-linked	-1.727	0.043	0.022	0.064	4.115	9.24E-05	0.588599728	ns	+
49	cg00111778	chr2:3833127	N shore	DCDC2C	DCDC2C			1.769	-0.056	-0.083	-0.029	-4.099	9.75E-05	0.608775308	ns	-
50	cg14545670	chr9:140064693	island	LRRC26	LRRC26	TSS1500		-0.488	-0.027	-0.040	-0.014	-4.094	9.95E-05	0.608922204	ns	-
51	cg11858450	chr19:15132184	island	CCDC105	CCDC105	Body		2.260	0.054	0.028	0.080	4.086	0.000102493	0.614695235	ns	+
52	cg09723313	chr7:142420303	open sea	TRBC2	TRBC2			-1.591	0.052	0.027	0.078	4.066	0.000109936	0.626413277	ns	+
53	cg24038905	chr4:76813684	open sea	PPEF2	PPEF2	Body		2.455	0.027	0.014	0.041	4.064	0.000110605	0.626413277	ns	+
54	cg17499345	chr1:86048585	S shore	CCN1	CCN1	Body		2.750	0.026	0.013	0.039	4.060	0.00011211	0.626413277	ns	+
55	cg17569844	chrX:100446220	N shore	GHc-210E9.2	CENPI-DRP2			2.055	0.026	0.013	0.038	4.049	0.000116927	0.626413277	ns	+
56	cg09615821	chr16:70749564	open sea	VAC14	VAC14	Body		1.751	0.034	0.017	0.050	4.045	0.000118346	0.626413277	ns	+
57	cg20930482	chr6:106520549	open sea		PRDM1			2.764	-0.029	-0.044	-0.015	-4.039	0.000120876	0.626413277	ns	-
58	cg14086205	chr18:31603693	open sea	NOL4	NOL4	5'UTR		1.263	0.023	0.012	0.034	4.038	0.000121346	0.626413277	ns	+
59	cg14844141	chr3:131245672	N shore	BCL2L12P1	CPNE4			-1.173	0.025	0.013	0.038	4.037	0.000121761	0.626413277	ns	+
60	cg23331961	chr15:72564179	N shore	PARP6	PARP6	TSS1500		1.603	-0.042	-0.062	-0.021	-4.034	0.000123254	0.626413277	ns	-
61	cg19219672	chr17:79804113	N shore	P4HB	P4HB	Body		1.781	-0.021	-0.031	-0.010	-4.030	0.000124927	0.626413277	ns	-
62	cg03812891	chr9:84386734	open sea	LOC101927502	TLE1	Body		1.037	-0.093	-0.139	-0.047	-4.029	0.000128083	0.63187795	ns	-
63	cg04858631	chr10:74035570	S shore	DDIT4	DDIT4	3'UTR		0.877	-0.035	-0.052	-0.018	-4.012	0.000132979	0.635034067	ns	-
64	cg25956753	chr8:145230086	open sea	MROH1	MROH1	Body		3.184	-0.028	-0.042	-0.014	-4.005	0.000136377	0.635034067	ns	-
65	cg20066216	chr4:80573486	open sea		GK2			2.280	-0.028	-0.041	-0.014	-4.003	0.000137554	0.635034067	ns	-
66	cg00354641	chr6:30071503	S shore	TRIM31	TRIM31	Body		-2.246	0.043	0.022	0.064	4.001	0.000138135	0.635034067	ns	+
67	cg26894820	chr12:91505265	open sea	LUM	LUM	1stExon		1.865	0.030	0.015	0.044	4.000	0.000139103	0.635034067	ns	+
68	cg17295489	chr12:57998192	open sea	DTX3	DTX3	TSS1500	promoter-linked	-0.691	-0.033	-0.049	-0.016	-3.994	0.000141738	0.637544595	ns	-
69	cg11959215	chr7:151722222	N shore	GALNT11	GALNT11	TSS1500	promoter-linked	-0.654	0.056	0.028	0.085	3.977	0.000150485	0.649431927	ns	+
70	cg16852323	chr17:6348225	S shore	PIMREG	PIMREG	5'UTR		-3.230	-0.033	-0.049	-0.016	-3.973	0.000152449	0.649431927	ns	-
71	cg02178433	chr2:121427490	open sea		GLI2		unknown cellspecific	-1.523	-0.030	-0.045	-0.015	-3.972	0.000153492	0.649431927	ns	-
72	cg24237115	chr1:25885648	open sea	LDLRAP1	LDLRAP1	Body		0.918	0.025	0.012	0.037	3.968	0.00015541	0.649431927	ns	+
73	cg04375253	chr5:66241308	open sea	MAST4	MAST4	Body		2.330	-0.032	-0.048	-0.016	-3.960	0.000159857	0.649431927	ns	-
74	cg09957624	chr17:63317229	open sea		RGS9		non-gene-linked	1.126	0.026	0.013	0.040	3.958	0.000161102	0.649431927	ns	+
75	cg11138035	chr5:74244655	open sea	CTD-2503O16.4	FAM169A			2.437	-0.042	-0.064	-0.021	-3.955	0.000162898	0.649431927	ns	-

76	cg21505509	chr8:37605717	open sea	ERLIN2	ERLIN2	TSS200	promoter-linked cell specific	0.937	0.034	0.017	0.051	3.949	0.000166397	0.649431927	ns	+
77	cg17062083	chr2:217592637	open sea	AC007563.5	IGFBP5			3.200	-0.040	-0.060	-0.020	-3.947	0.000167548	0.649431927	ns	-
78	cg01591592	chr11:49399704	open sea	LOC729960	FOLH1			0.538	0.032	0.016	0.048	3.944	0.000168793	0.649431927	ns	+
79	cg08474396	chr16:27237656	open sea	NSMCE1	NSMCE1	Body		2.197	-0.024	-0.037	-0.012	-3.943	0.000169682	0.649431927	ns	-
80	cg10076009	chrX:128656725	N shore	SMARCA1	SMARCA1	Body	unknown cell specific	-0.901	-0.046	-0.069	-0.023	-3.942	0.000169983	0.649431927	ns	-
81	cg19787469	chr16:86811993	open sea		FOXL1			0.587	0.027	0.013	0.040	3.939	0.000172164	0.649431927	ns	+
82	cg19471655	chr19:51487109	open sea	KLK7	KLK7	5'UTR		-1.504	0.049	0.024	0.073	3.936	0.000174106	0.649431927	ns	+
83	cg07851597	chr8:28360385	open sea	FZD3	FZD3	5'UTR		-0.075	-0.040	-0.060	-0.020	-3.929	0.000178119	0.656397283	ns	-
84	cg02553047	chrX:64855503	open sea	MSN	MSN			-0.090	0.036	0.018	0.054	3.915	0.000187237	0.666490433	ns	+
85	cg16249711	chr14:55907501	S shore	TBPL2	TBPL2	TSS1500		2.911	0.037	0.018	0.056	3.903	0.000195091	0.666490433	ns	+
86	cg01284438	chr5:175250977	open sea	CPLX2	CPLX2	5'UTR		1.664	-0.044	-0.066	-0.021	-3.902	0.000195869	0.666490433	ns	-
87	cg23616046	chrX:133931641	S shore	PABIR2	PABIR2	TSS1500	promoter-linked	-1.286	-0.043	-0.065	-0.021	-3.902	0.000195962	0.666490433	ns	-
88	cg02558132	chr3:123411198	open sea	MYLK	MYLK	Body		2.184	0.028	0.014	0.042	3.901	0.000196195	0.666490433	ns	+
89	cg00780871	chr8:47917029	open sea		SPIDR			1.407	0.029	0.014	0.043	3.900	0.000196926	0.666490433	ns	+
90	cg27247689	chr8:73445394	N shelf		KCNB2			2.273	0.027	0.013	0.041	3.897	0.000199016	0.666490433	ns	+
91	cg06084843	chr14:58595679	open sea	ARMH4	ARMH4	Body		2.766	0.022	0.011	0.034	3.897	0.000199071	0.666490433	ns	+
92	cg23899779	chr2:241190395	open sea	AC124861.1	OTOS		unknown cell specific	2.665	0.028	0.014	0.043	3.892	0.000202379	0.666490433	ns	+
93	cg14426373	chr19:51220781	S shore	SHANK1	SHANK1	TSS1500		2.161	0.026	0.013	0.039	3.892	0.000202648	0.666490433	ns	+
94	cg17321385	chr2:132429793	N shore		C2orf27A			0.819	0.021	0.010	0.032	3.874	0.000215893	0.690835532	ns	+
95	cg03434624	chr12:123380883	island	VPS37B	VPS37B	TSS200	promoter-linked	-3.642	0.072	0.035	0.109	3.871	0.00021988	0.690835532	ns	+
96	cg01900358	chr5:1476090	open sea	LPCAT1	LPCAT1	Body		2.131	0.023	0.011	0.034	3.867	0.000220806	0.690835532	ns	+
97	cg23813603	chr20:859405	open sea	ANGPT4	ANGPT4	Body		2.177	0.035	0.017	0.053	3.861	0.000225714	0.690835532	ns	+
98	cg07533824	chr19:13363864	N shelf	CACNA1A	CACNA1A	Body		2.962	0.025	0.012	0.037	3.860	0.000226565	0.690835532	ns	+
99	cg05449108	chr17:114319	island	RPH3AL	RPH3AL	Body		1.440	0.104	0.050	0.157	3.865	0.000227564	0.690835532	ns	+
100	cg25483073	chr20:15026122	open sea	MACROD2	MACROD2	Body		1.847	0.032	0.016	0.049	3.857	0.000229101	0.690835532	ns	+
101	cg10805837	chrX:134618613	open sea		INTS6L		unknown cell specific	1.422	0.040	0.019	0.061	3.850	0.000234457	0.690835532	ns	+
102	cg00416243	chr6:143857467	N shore	PHACTR2	PHACTR2			1.732	0.032	0.015	0.049	3.849	0.00023521	0.690835532	ns	+
103	cg10205002	chr11:134748929	open sea		B3GAT1			2.945	0.031	0.015	0.048	3.843	0.000239778	0.690835532	ns	+
104	cg00737905	chr6:52198683	open sea		PAQR8			-2.126	-0.047	-0.071	-0.022	-3.840	0.000242157	0.690835532	ns	-
105	cg09137533	chr22:46469091	S shore	MIRLET7BHG	PPARA			-1.694	0.032	0.015	0.049	3.840	0.000242201	0.690835532	ns	+
106	cg04215950	chr1:7125137	S shelf	CAMTA1	CAMTA1	Body		2.268	0.022	0.010	0.033	3.838	0.000243846	0.690835532	ns	+
107	cg14255617	chr6:32729118	N shore	HLA-DQB2	HLA-DQB2	Body		2.858	0.066	0.032	0.100	3.839	0.00024489	0.690835532	ns	+
108	cg21006539	chr8:6793506	open sea	DEFA4	DEFA4	3'UTR		1.635	0.056	0.027	0.085	3.834	0.000248087	0.690835532	ns	+
109	cg14544289	chr14:78051204	open sea	SPTLC2	SPTLC2	Body		0.912	0.026	0.012	0.039	3.833	0.000248832	0.690835532	ns	+
110	cg06537110	chr7:1250500	N shore		UNCX			1.411	0.032	0.015	0.048	3.830	0.000250752	0.690835532	ns	+
111	cg18745424	chr3:100401200	open sea	ADGRG7	ADGRG7	Body		2.921	0.033	0.016	0.051	3.830	0.000251179	0.690835532	ns	+
112	cg16479923	chr6:18491607	open sea		RNF144B			3.064	-0.039	-0.059	-0.019	-3.826	0.000254597	0.690835532	ns	-
113	cg04123469	chr17:38694432	open sea		CCR7			2.393	0.032	0.015	0.048	3.823	0.00025736	0.690835532	ns	+
114	cg20955567	chr9:102629999	open sea		NR4A3			0.151	0.040	0.019	0.061	3.823	0.000257481	0.690835532	ns	+
115	cg20580788	chr17:189410	open sea	RPH3AL	RPH3AL	5'UTR		2.405	0.032	0.016	0.049	3.814	0.000265465	0.692625122	ns	+
116	cg22633390	chr7:100964966	island	IFT22	IFT22	1stExon		-0.935	-0.037	-0.056	-0.017	-3.812	0.00026708	0.692625122	ns	-
117	cg26701826	chr4:108814604	open sea	SGMS2	SGMS2	5'UTR		-4.179	0.097	0.047	0.148	3.816	0.000268555	0.692625122	ns	+
118	cg22317649	chr14:36225113	open sea	RALGAPA1	RALGAPA1	Body		1.877	-0.026	-0.040	-0.013	-3.808	0.000270448	0.692625122	ns	-
119	cg20761098	chr9:124479719	open sea	DAB2IP	DAB2IP	Body		2.480	0.024	0.012	0.037	3.806	0.000272645	0.692625122	ns	+
120	cg16126759	chrX:55743906	open sea	RRAGB	RRAGB	TSS1500	promoter-linked	0.358	0.027	0.013	0.041	3.804	0.000274635	0.692625122	ns	+
121	cg12786198	chr14:55741714	S shelf	FBXO34	FBXO34	5'UTR		1.589	-0.025	-0.039	-0.012	-3.801	0.0002774	0.692625122	ns	-
122	cg05318071	chr2:118845276	N shore	INSIG2	INSIG2	TSS1500		-1.187	0.045	0.021	0.069	3.797	0.000281556	0.692625122	ns	+
123	cg03202564	chr10:50977130	island		OGDHL			-0.437	0.055	0.026	0.084	3.794	0.000284299	0.692625122	ns	+
124	cg14646540	chr20:44944775	S shelf		CDH22			2.384	0.028	0.014	0.043	3.793	0.000284549	0.692625122	ns	+
125	cg13215982	chr13:24078497	open sea		TNFRSF19			2.865	-0.023	-0.034	-0.011	-3.793	0.000284608	0.692625122	ns	-
126	cg18387316	chr2:227002371	open sea		NYAP2			1.734	-0.030	-0.046	-0.014	-3.793	0.000285322	0.692625122	ns	-
127	cg18665594	chr5:101119420	open sea		SLCO4C1			0.103	0.053	0.025	0.080	3.789	0.000289242	0.696613773	ns	+
128	cg07186032	chr3:56950340	open sea	ARHGEF3	ARHGEF3	Body	unknown cell specific	-0.606	-0.024	-0.037	-0.011	-3.783	0.000294699	0.704209898	ns	-
129	cg01907347	chr20:36298468	open sea		CTNNBL1			0.701	0.026	0.012	0.039	3.778	0.000299706	0.709184916	ns	+
130	cg08523025	chr2:35591060	open sea		CRIM1			2.141	0.026	0.012	0.040	3.775	0.000302751	0.709184916	ns	+
131	cg10502220	chr4:176733966	open sea	GPM6A	GPM6A	1stExon		-0.091	0.039	0.019	0.060	3.774	0.000303759	0.709184916	ns	+
132	cg06921345	chr8:60506639	open sea	RP11-379I19.1	TOX			2.645	0.025	0.012	0.039	3.768	0.000309919	0.709184916	ns	+
133	cg10440011	chr17:1933183	island	DPH1	DPH1	TSS1500	promoter-linked	-0.374	-0.021	-0.033	-0.010	-3.768	0.000310106	0.709184916	ns	-
134	cg07089660	chr8:6795162	open sea	DEFA4	DEFA4	5'UTR		1.635	0.029	0.014	0.045	3.766	0.00031294	0.709184916	ns	+
135	cg08805287	chr2:3802050	open sea	DCDC2C	DCDC2C	Body		0.521	-0.077	-0.118	-0.037	-3.769	0.000313481	0.709184916	ns	-
136	cg10451752	chr20:62873536	open sea	MYT1	MYT1	3'UTR		1.833	0.026	0.012	0.040	3.759	0.0003198	0.709184916	ns	+
137	cg08219213	chr14:94641222	island	PPP4R4	PPP4R4	Body		-2.668	0.039	0.018	0.059	3.759	0.00032053	0.709184916	ns	+
138	cg22395913	chr14:77844147	S shore	TMED8	TMED8	TSS1500	promoter-linked	2.419	0.038	0.018	0.058	3.757	0.000321859	0.709184916	ns	+
139	cg02525435	chr16:88977768	S shore	CBFA2T3	CBFA2T3	5'UTR		1.113	0.020	0.009	0.031	3.757	0.000322641	0.709184916	ns	+
140	cg05988605	chr8:28173887	open sea	PNOC	PNOC	TSS1500		2.741	0.031	0.014	0.047	3.748	0.000332648	0.709184916	ns	+
141	cg05777357	chr9:126776999	island	LHX2	LHX2	Body		-0.561	-0.021	-0.033	-0.010	-3.747	0.000333507	0.709184916	ns	-
142	cg01056004	chr10:98948259	S shelf	SLIT1	SLIT1		unknown cell specific	-1.151	0.061	0.029	0.093	3.747	0.000334186	0.709184916	ns	+
143	cg09267188	chr8:97340225	open sea	PTDSS1	PTDSS1	Body	gene-linked	-0.558	0.041	0.019	0.062	3.743	0.000337568	0.709184916	ns	+
144	cg18091385	chr14:99502466	open sea		BCL11B			-2.526	-0.050	-0.076	-0.023	-3.742	0.000338997	0.709184916	ns	-
145	cg03157512	chr22:19223165	open sea	CLTCL1	CLTCL1	Body		1.278	0.033	0.016	0.051	3.742	0.000339416	0.709184916	ns	+
146	cg23784400	chr19:19576195	open sea	GATAD2A	GATAD2A	1stExon		2.809	0.036	0.017	0.055	3.739	0.000342418	0.709184916	ns	+
147	cg07342674	chr17:22020985	S shelf	MTRNR2L1	MTRNR2L1	TSS1500		1.336	-0.026	-0.039	-0.012	-3.739	0.0003431	0.709184916	ns	-
148	cg27525524	chr1:16468313	S shelf	EPHA2	EPHA2	Body		-1.642	-0.034	-0.052	-0.016	-3.738	0.000343152	0.709184916	ns	-
149	cg18792689	chr1:109254098	open sea	FNDC7	FNDC7	TSS1500		1.727	-0.042	-0.065	-0.020	-3.731	0.000352117	0.722828472	ns	-
150	cg15429715	chr3:46905584	open sea	MYL3	MYL3	TSS1500		0.222	-0.043	-0.067	-0.020	-3.727	0.000357122	0.726118397	ns	-
151	cg18152496	chrX:48490674	open sea		WDR13			2.168	0.028	0.013	0.043	3.722	0.000363145	0.726118397	ns	+
152	cg17001328	chr19:2495886	S shore		GNG7			-0.162	-0.032	-0.050	-0.015	-3.722	0.000363145	0.726118397	ns	-

153	cg03983645	chr11:20181887	island	DBX1	DBX1	TSS200		-3.638	-0.051	-0.078	-0.024	-3.722	0.000363216	0.726118397	ns	-
154	cg01496203	chr4:121843035	N shore	PRDM5	PRDM5	Body		-2.204	-0.032	-0.049	-0.015	-3.719	0.000366375	0.727678366	ns	-
155	cg11388130	chr12:56225508	S shore	TMEM198B	DNAJC14	Body		-3.718	-0.036	-0.056	-0.017	-3.716	0.000369994	0.728539814	ns	-
156	cg01752072	chr1:38356296	open sea	INPP5B	INPP5B	Body	gene-linked	-1.993	0.037	0.017	0.057	3.715	0.000371573	0.728539814	ns	+
157	cg14366292	chr10:124328787	open sea	DMBT1	DMBT1	Body		0.950	-0.033	-0.051	-0.015	-3.712	0.000375017	0.730610413	ns	-
158	cg13039925	chr18:2889850	open sea	EMILIN2	EMILIN2	Body	unknown cellspecific	1.285	0.034	0.016	0.053	3.709	0.000378511	0.732749131	ns	+
159	cg00518055	chr2:28589359	open sea		FOSL2			-1.042	-0.034	-0.052	-0.016	-3.707	0.000381628	0.732833195	ns	-
160	cg08063492	chr14:90324658	open sea	EFCAB11	EFCAB11	Body		2.234	0.036	0.017	0.056	3.706	0.000383346	0.732833195	ns	+
161	cg14288579	chr2:237527328	open sea		ACKR3			2.642	0.036	0.017	0.056	3.704	0.000385944	0.733215949	ns	+
162	cg14335069	chr22:41956810	open sea	CSDC2	CSDC2	TSS1500		-2.198	0.036	0.016	0.055	3.699	0.000392194	0.740491259	ns	+
163	cg23075688	chr2:217561731	S shelf	IGFBP5	IGFBP5	TSS1500		-1.154	0.057	0.026	0.088	3.687	0.000409382	0.741444696	ns	+
164	cg23275355	chr8:73152431	open sea	LOC392232	TRPA1	Body		1.711	0.035	0.016	0.054	3.683	0.000414185	0.741444696	ns	+
165	cg26708484	chr1:6142608	open sea	KCNAB2	KCNAB2	Body	gene-linked cellspecific	-1.734	-0.034	-0.053	-0.016	-3.682	0.000415328	0.741444696	ns	-
166	cg24640561	chr13:108979138	open sea		TNFSF13B			2.670	0.028	0.013	0.044	3.680	0.000417984	0.741444696	ns	+
167	cg15656203	chr17:49442591	open sea		UTP18			-2.066	-0.057	-0.087	-0.026	-3.679	0.000419693	0.741444696	ns	-
168	cg02943412	chr10:120356034	S shore	PRLHR	PRLHR	TSS1500		-3.481	-0.043	-0.066	-0.020	-3.679	0.00041975	0.741444696	ns	-
169	cg11993160	chr18:12404041	N shelf		PRELID3A			-1.356	-0.036	-0.055	-0.016	-3.679	0.000419956	0.741444696	ns	-
170	cg02238229	chr14:106373139	open sea	IGHD2-8	IGHD2-8			0.997	-0.052	-0.081	-0.024	-3.678	0.00042116	0.741444696	ns	-
171	cg02014416	chr1:231376203	N shore	GNPAT	GNPAT	TSS1500		0.721	-0.044	-0.068	-0.020	-3.674	0.000425994	0.741444696	ns	-
172	cg03913499	chr18:55892629	open sea	NEDD4L	NEDD4L	Body		2.814	0.027	0.012	0.042	3.673	0.00042803	0.741444696	ns	+
173	cg22548031	chr14:32686880	open sea		ARHGAP5			-0.318	-0.049	-0.075	-0.022	-3.671	0.000430278	0.741444696	ns	-
174	cg03298319	chr14:33403060	island	NPAS3	NPAS3		unknown cellspecific	-3.736	0.041	0.019	0.063	3.670	0.000431651	0.741444696	ns	+
175	cg14818192	chr1:111423791	open sea	CD53	CD53	5'UTR	promoter-linked	0.378	-0.024	-0.037	-0.011	-3.668	0.000434953	0.741444696	ns	-
176	cg21442003	chr2:158454243	S shore	ACVR1C	ACVR1C	TSS200	unknown cellspecific	-3.049	0.030	0.014	0.046	3.667	0.000435909	0.741444696	ns	+
177	cg01966791	chr20:62572875	S shore	UCKL1	UCKL1	Body	gene-linked	-1.336	-0.065	-0.100	-0.030	-3.664	0.000443506	0.741444696	ns	-
178	cg10404643	chr11:8718658	open sea	DENND2B	DENND2B	Body		1.427	-0.041	-0.064	-0.019	-3.660	0.000447111	0.741444696	ns	-
179	cg04049812	chr20:13251901	open sea	ISM1	ISM1	Body		0.928	-0.029	-0.044	-0.013	-3.658	0.000449861	0.741444696	ns	-
180	cg05571129	chrX:6626831	open sea	RP11-1M18.1	VCX3A			1.610	0.026	0.012	0.040	3.658	0.000450407	0.741444696	ns	+
181	cg24409566	chr11:2933967	S shelf	SLC22A18	SLC22A18	Body	unknown cellspecific	0.021	-0.035	-0.054	-0.016	-3.657	0.000451601	0.741444696	ns	-
182	cg13304359	chr6:166823219	N shelf	RPS6KA2	RPS6KA2	3'UTR		0.915	0.052	0.024	0.080	3.656	0.000453089	0.741444696	ns	+
183	cg18014500	chr10:114074843	open sea	GUCY2GP	TECTB	Body	unknown cellspecific	-2.192	0.023	0.011	0.036	3.656	0.000453224	0.741444696	ns	+
184	cg05280527	chr14:80328450	S shore	NRXN3	NRXN3	3'UTR		1.004	-0.036	-0.055	-0.016	-3.652	0.000459548	0.741444696	ns	-
185	cg20546782	chr17:3505636	open sea	TRPV1	TRPV1	5'UTR		0.423	-0.021	-0.033	-0.010	-3.650	0.000462934	0.741444696	ns	-
186	cg22680451	chr1:160992367	S shore	F11R	F11R	TSS1500		2.133	-0.021	-0.032	-0.009	-3.648	0.000464623	0.741444696	ns	-
187	cg20051530	chr11:65816935	S shore	GAL3ST3	GAL3ST3	TSS1500	unknown cellspecific	0.361	-0.047	-0.073	-0.021	-3.646	0.000468159	0.741444696	ns	-
188	cg23765231	chr8:81539016	open sea		ZNF704			0.151	0.019	0.009	0.029	3.645	0.000469925	0.741444696	ns	+
189	cg25247998	chr8:26108217	open sea		PPP2R2A			2.222	-0.026	-0.041	-0.012	-3.645	0.000470066	0.741444696	ns	-
190	cg24894657	chr2:242714413	S shelf		GAL3ST2			-2.829	-0.189	-0.292	-0.086	-3.653	0.00047126	0.741444696	ns	-
191	cg12324588	chr20:58632413	open sea	C20orf197	CDH26	Body	promoter-linked	-2.735	-0.038	-0.059	-0.017	-3.642	0.000475032	0.741444696	ns	-
192	cg03125498	chr19:34112111	N shore	CHST8	CHST8	TSS1500		0.989	0.033	0.015	0.050	3.641	0.000475915	0.741444696	ns	+
193	cg19153196	chr8:58663328	open sea	RP11-388G22.1	FAM110B			2.441	-0.022	-0.034	-0.010	-3.641	0.000476619	0.741444696	ns	-
194	cg24122929	chr19:38893252	N shore	FAM98C	FAM98C	TSS1500		1.023	0.042	0.019	0.065	3.640	0.000478591	0.741444696	ns	+
195	cg14360579	chr1:209542809	open sea	RP11-372M18.2	CAMK1G			-2.066	0.037	0.017	0.057	3.638	0.00048089	0.741444696	ns	+
196	cg06425026	chr16:2577404	N shore	AMDHD2	AMDHD2	Body		2.110	0.024	0.011	0.037	3.638	0.000481031	0.741444696	ns	+
197	cg12719030	chr12:132102811	open sea		SFSWAP			2.433	0.039	0.018	0.061	3.638	0.000481241	0.741444696	ns	+
198	cg04327001	chr18:67729695	open sea	RTTN	RTTN	Body		0.997	0.029	0.013	0.044	3.637	0.000483404	0.741444696	ns	+
199	cg23656322	chr1:153533922	open sea	S100A2	S100A2	Body	unknown cellspecific	0.278	-0.066	-0.103	-0.030	-3.637	0.000486032	0.741444696	ns	-
200	cg11912608	chr7:103393558	open sea	RELN	RELN	Body		1.435	0.028	0.013	0.043	3.633	0.000489494	0.741444696	ns	+
201	cg09207457	chr14:101369855	open sea	MEG8	RTL1	Body		3.582	0.035	0.016	0.054	3.632	0.000491325	0.741444696	ns	+
202	cg11518764	chr15:31751448	open sea		OTUD7A			1.661	0.023	0.010	0.036	3.630	0.000493316	0.741444696	ns	+
203	cg21718051	chr6:12071462	open sea	HIVEP1	HIVEP1	Body		0.001	0.025	0.011	0.038	3.629	0.000495	0.741444696	ns	+
204	cg26533311	chr6:27206911	open sea		PRSS16			0.209	0.059	0.027	0.092	3.629	0.000497087	0.741444696	ns	+
205	cg07832738	chr17:11142923	N shore		SHISA6			-3.049	0.041	0.019	0.064	3.626	0.000500222	0.741444696	ns	+
206	cg17374364	chr5:76929064	N shelf	OTP	OTP	Body		0.046	-0.033	-0.051	-0.015	-3.626	0.000500402	0.741444696	ns	-
207	cg12017635	chr12:72343656	open sea	TPH2	TPH2	Body		1.484	0.029	0.013	0.044	3.625	0.000501782	0.741444696	ns	+
208	cg09535924	chr2:66671659	N shore	MEIS1	MEIS1	Body		-2.878	-0.037	-0.058	-0.017	-3.616	0.000517062	0.745712596	ns	-
209	cg23162571	chr7:65196762	open sea	LOC441242	VKORC1L1			-2.181	-0.061	-0.095	-0.028	-3.617	0.000519024	0.745712596	ns	-
210	cg21637886	chr12:25007140	open sea	BCAT1	BCAT1	Body		1.482	0.022	0.010	0.035	3.614	0.000520247	0.745712596	ns	+
211	cg01235114	chr11:120504209	open sea	GRIK4	GRIK4	5'UTR		0.171	-0.019	-0.030	-0.009	-3.614	0.000520961	0.745712596	ns	-
212	cg19360943	chr12:6762431	open sea	ING4	ING4	Body		3.430	0.043	0.019	0.067	3.614	0.000521276	0.745712596	ns	+
213	cg16004722	chr15:95674587	open sea	RP11-255M2.2	MCTP2			2.675	-0.044	-0.069	-0.020	-3.614	0.000521799	0.745712596	ns	-
214	cg22172358	chr20:4910667	open sea	SLC23A2	SLC23A2	Body		1.408	-0.037	-0.058	-0.017	-3.613	0.000522777	0.745712596	ns	-
215	cg23093692	chr1:234367145	island	SLC35F3	SLC35F3	Body		3.004	0.065	0.029	0.101	3.614	0.000524175	0.745712596	ns	+
216	cg08723064	chrX:68761037	S shelf		FAM155B			0.003	-0.019	-0.029	-0.008	-3.610	0.000527837	0.747446896	ns	-
217	cg00408210	chr22:21401992	S shore	LRRC74B	LRRC74B	Body		2.598	0.024	0.011	0.038	3.607	0.000532999	0.748497052	ns	+
218	cg06804581	chr2:148602526	island	ACVR2A	ACVR2A	5'UTR		-3.573	0.041	0.019	0.064	3.607	0.000533473	0.748497052	ns	+
219	cg00004963	chr6:147124996	open sea	ADGB	ADGB	TSS200		2.789	0.033	0.015	0.051	3.605	0.000537136	0.750194607	ns	+
220	cg06506352	chr6:7468781	open sea		RIOK1			1.704	0.041	0.018	0.063	3.601	0.00054449	0.756562792	ns	+
221	cg03060661	chr16:88934005	N shelf	PABPN1L	PABPN1L	TSS1500		3.210	-0.031	-0.048	-0.014	-3.600	0.000546642	0.756562792	ns	-
222	cg05403241	chr2:121776604	open sea		GLI2			0.615	0.040	0.018	0.063	3.595	0.000554187	0.761875419	ns	+
223	cg26439975	chr1:84873633	open sea	DNASE2B	DNASE2B	TSS1500		2.422	-0.024	-0.037	-0.011	-3.594	0.00055615	0.761875419	ns	-
224	cg03878654	chr16:73081124	N shore	ZFHX3	ZFHX3	5'UTR		0.567	-0.035	-0.054	-0.016	-3.593	0.000557953	0.761875419	ns	-
225	cg00910015	chr2:3826621	open sea	DCDC2C	DCDC2C			2.791	0.047	0.021	0.073	3.592	0.000560697	0.762218423	ns	+
226	cg12130976	chrX:57312244	open sea	FAAH2	FAAH2	TSS1500		2.614	0.050	0.022	0.078	3.584	0.000575313	0.778627963	ns	+
227	cg01300852	chr6:166955885	open sea	RPS6KA2	RPS6KA2	Body	promoter-linked	2.227	0.028	0.013	0.044	3.581	0.000581311	0.782597132	ns	+
228	cg18359044	chr6:71900580	open sea		OGFRL1			-2.091	0.041	0.018	0.065	3.580	0.000583454	0.782597132	ns	+
229	cg17782495	chr2:163466280	open sea	KCNH7	KCNH7	Body		1.509	0.030	0.013	0.047	3.579	0.000585922	0.782597132	ns	+

230	cg00835825	chr16:85608058	open sea		GSE1			2.439	0.074	0.033	0.115	3.575	0.000598245	0.793796706	ns	+
231	cg14060535	chr7:127811715	S shelf		LEP			1.901	0.020	0.009	0.031	3.572	0.000599497	0.793796706	ns	+
232	cg08626131	chr5:42812509	open sea	SEPP1	SEPP1	TSS1500		1.280	-0.028	-0.044	-0.012	-3.568	0.000606991	0.794652739	ns	-
233	cg17935021	chrX:101853962	N shore	ARMCX5	ARMCX5	TSS1500		0.750	0.019	0.008	0.030	3.567	0.000609262	0.794652739	ns	+
234	cg26562532	chr19:55864365	island	COX6B2	COX6B2	3'UTR		2.530	0.026	0.012	0.041	3.562	0.000619555	0.794652739	ns	+
235	cg25434864	chr8:83353753	open sea		SNX16			3.053	0.114	0.050	0.177	3.568	0.000620235	0.794652739	ns	+
236	cg06087739	chr18:928069	open sea	RP11-672L10.1	ADCYAP1			1.868	0.031	0.014	0.048	3.561	0.000621492	0.794652739	ns	+
237	cg01438174	chr9:130640301	S shore	AK1	AK1	TSS1500		-0.434	-0.021	-0.032	-0.009	-3.559	0.00062415	0.794652739	ns	-
238	cg06657041	chr9:121501743	open sea	LINC02578	BRINP1			2.125	0.029	0.013	0.046	3.558	0.000627959	0.794652739	ns	+
239	cg05506600	chr15:72447967	open sea	RP11-2I17.4	GRAMD2			-0.445	-0.035	-0.055	-0.016	-3.557	0.000629277	0.794652739	ns	-
240	cg10143067	chr10:118892423	island	VAX1	VAX1	Body		-3.176	0.043	0.019	0.068	3.556	0.000630536	0.794652739	ns	+
241	cg06717302	chr16:2278224	open sea	E4F1	E4F1	Body		2.736	0.028	0.012	0.044	3.555	0.000634217	0.794652739	ns	+
242	cg02146740	chr8:3890512	open sea	CSMD1	CSMD1	Body		2.022	-0.021	-0.033	-0.009	-3.553	0.000637174	0.794652739	ns	-
243	cg10998146	chr13:27447706	open sea		GPR12			2.273	0.044	0.019	0.069	3.551	0.000640548	0.794652739	ns	+
244	cg07915730	chr12:3371890	open sea	TSPAN9	TSPAN9	Body		2.078	0.040	0.018	0.063	3.551	0.000642038	0.794652739	ns	+
245	cg19624318	chr7:62514926	island	RP11-196D18.1	ZNF733P			-1.305	-0.039	-0.061	-0.017	-3.551	0.000642638	0.794652739	ns	-
246	cg24976802	chr18:29027614	open sea	DSG3	DSG3	TSS200		1.303	-0.023	-0.036	-0.010	-3.543	0.000659022	0.794652739	ns	-
247	cg17196675	chr10:129936356	open sea		MKI67			-0.394	0.036	0.016	0.056	3.542	0.000661022	0.794652739	ns	+
248	cg12566136	chr9:124857977	S shelf		TTLL11			-0.481	-0.025	-0.040	-0.011	-3.542	0.000661201	0.794652739	ns	-
249	cg17904704	chr2:43804362	open sea	THADA	THADA	ExonBnd	gene-linked	0.045	-0.032	-0.050	-0.014	-3.541	0.000663811	0.794652739	ns	-
250	cg06667833	chrX:130611460	open sea		IGSF1			-1.693	-0.037	-0.057	-0.016	-3.538	0.000670433	0.794652739	ns	-
251	cg05545777	chr5:101119128	open sea		SLCO4C1			-0.500	0.057	0.025	0.089	3.537	0.000674219	0.794652739	ns	+
252	cg02352904	chr1:223566705	island	CCDC185	CCDC185	TSS200	unknown cell specific	-0.626	-0.041	-0.065	-0.018	-3.535	0.000675744	0.794652739	ns	-
253	cg12193731	chr12:52300911	island	ACVRL1	ACVRL1	TSS1500	unknown cell specific	-3.397	-0.081	-0.126	-0.035	-3.539	0.000675914	0.794652739	ns	-
254	cg13041518	chr20:12339571	open sea		BTBD3			2.606	0.029	0.013	0.046	3.531	0.000684535	0.794652739	ns	+
255	cg19002763	chr8:21910390	N shelf	DMTN	DMTN	TSS1500		1.561	0.019	0.008	0.029	3.531	0.00068603	0.794652739	ns	+
256	cg11426075	chr1:8086959	S shore	ERRFI1	ERRFI1	TSS1500		-0.509	0.025	0.011	0.038	3.531	0.00068614	0.794652739	ns	+
257	cg08846760	chr16:88922618	N shore	TRAPPC2L	TRAPPC2L	TSS1500		1.430	0.018	0.008	0.028	3.527	0.000694683	0.794652739	ns	+
258	cg14711616	chr4:2802197	open sea	SH3BP2	SH3BP2	5'UTR		-2.762	0.033	0.015	0.052	3.526	0.000695323	0.794652739	ns	+
259	cg19512055	chr13:98082182	N shelf		RAP2A			2.410	-0.024	-0.037	-0.010	-3.526	0.000696574	0.794652739	ns	-
260	cg14294077	chr13:111981135	S shelf	TEX29	TEX29	Body		1.589	-0.020	-0.031	-0.009	-3.526	0.000696758	0.794652739	ns	-
261	cg21338239	chr17:55865735	open sea	RN7SKP94	CCDC182			2.624	0.023	0.010	0.036	3.524	0.00070192	0.794652739	ns	+
262	cg25740457	chr11:6292896	S shore	CCKBR	CCKBR	3'UTR		0.454	0.067	0.029	0.105	3.525	0.000703495	0.794652739	ns	+
263	cg26135325	chr1:152595322	open sea	LCE3A	LCE3A	1stExon		-0.613	-0.045	-0.070	-0.020	-3.522	0.00070649	0.794652739	ns	-
264	cg07899100	chr16:48048726	open sea		ABCC12			2.805	0.028	0.012	0.043	3.521	0.000706792	0.794652739	ns	+
265	cg21156793	chr2:71301576	open sea	NAGK	NAGK	Body		2.978	0.046	0.020	0.073	3.518	0.000713872	0.794652739	ns	+
266	cg07047360	chr9:92277266	open sea	UNQ6494	GADD45G	Body		-2.495	-0.090	-0.141	-0.039	-3.522	0.000718186	0.794652739	ns	-
267	cg12726960	chr3:36421703	island	STAC	STAC	TSS1500		-2.290	0.022	0.010	0.035	3.516	0.000719903	0.794652739	ns	+
268	cg01804117	chr16:67783269	open sea	RANBP10	RANBP10	Body		1.698	0.022	0.010	0.034	3.516	0.000720429	0.794652739	ns	+
269	cg04737114	chr7:96654931	S shore	DLX5	DLX5	TSS1500		-2.826	-0.037	-0.059	-0.016	-3.514	0.000723411	0.794652739	ns	-
270	cg24404405	chr4:29328611	open sea		PCDH7			-2.502	-0.029	-0.046	-0.013	-3.514	0.000723704	0.794652739	ns	-
271	cg14294797	chr22:45564417	S shelf	NUP50	NUP50	5'UTR		2.065	-0.025	-0.039	-0.011	-3.514	0.000724089	0.794652739	ns	-
272	cg11238366	chr2:38830691	island	HNRNPLL	HNRNPLL	TSS1500	promoter-linked	-2.269	0.022	0.010	0.035	3.512	0.000729779	0.794652739	ns	+
273	cg01411366	chr3:112012867	open sea	SLC9C1	SLC9C1	5'UTR		-0.447	-0.033	-0.052	-0.014	-3.509	0.000736315	0.794652739	ns	-
274	cg12901910	chr1:35586453	island	EFCAB14P1	ZMYM1			-1.695	0.039	0.017	0.061	3.508	0.000737583	0.794652739	ns	+
275	cg23483530	chr5:176759977	S shore	LMAN2	LMAN2	Body	gene-linked cell specific	0.272	0.052	0.023	0.082	3.507	0.00074231	0.794652739	ns	+
276	cg14824005	chr3:105895856	open sea		CBLB		promoter-linked	-2.033	0.025	0.011	0.040	3.506	0.000742913	0.794652739	ns	+
277	cg19144497	chr15:74836094	S shelf	ARID3B	ARID3B	5'UTR		-2.689	-0.049	-0.076	-0.021	-3.505	0.000746885	0.794652739	ns	-
278	cg24271951	chr19:42989522	open sea	LIPE-AS1	CEACAM1	Body		2.144	-0.018	-0.029	-0.008	-3.502	0.000752356	0.794652739	ns	-
279	cg16352612	chr7:76054035	open sea	ZP3	ZP3	TSS1500		1.684	0.021	0.009	0.034	3.501	0.000754769	0.794652739	ns	+
280	cg01815720	chr1:31277948	N shelf		LAPTM5			0.098	-0.016	-0.026	-0.007	-3.500	0.000757925	0.794652739	ns	-
281	cg21851911	chr8:75825861	open sea		PI15			0.751	0.018	0.008	0.028	3.499	0.00076024	0.794652739	ns	+
282	cg14789272	chr5:54456056	open sea	CDC20B	CDC20B	Body		-1.956	-0.036	-0.057	-0.016	-3.497	0.000764649	0.794652739	ns	-
283	cg05395867	chr8:109569142	open sea		TMEM74			2.833	0.025	0.011	0.039	3.497	0.000766003	0.794652739	ns	+
284	cg18990407	chr3:184297380	N shelf	EPHB3	EPHB3	Body		1.000	-0.040	-0.063	-0.017	-3.496	0.000768966	0.794652739	ns	-
285	cg10693390	chr11:12411772	open sea	PARVA	PARVA	Body		0.453	0.024	0.010	0.037	3.493	0.00077677	0.794652739	ns	+
286	cg10277282	chr1:87620483	S shelf	LINC01140	HS2ST1	Body		2.585	0.028	0.012	0.043	3.492	0.000778698	0.794652739	ns	+
287	cg24181769	chr14:93171393	open sea	LGMN	LGMN	Body		2.001	0.025	0.011	0.039	3.492	0.000778982	0.794652739	ns	+
288	cg21298703	chr12:91573749	open sea	DCN	DCN	5'UTR		1.709	-0.018	-0.028	-0.008	-3.491	0.000780727	0.794652739	ns	-
289	cg23844628	chr22:25706205	open sea		IGLL3P			2.606	0.030	0.013	0.047	3.490	0.000782361	0.794652739	ns	+
290	cg10507591	chr20:31407542	island	MAPRE1	MAPRE1	TSS200	promoter-linked	-2.789	0.028	0.012	0.043	3.490	0.000783855	0.794652739	ns	+
291	cg22363563	chrX:47045482	open sea	RBM10	RBM10	ExonBnd		1.974	0.025	0.011	0.040	3.490	0.000784323	0.794652739	ns	+
292	cg26189066	chr19:12768132	S shore	MAN2B1	MAN2B1	Body		1.824	0.032	0.014	0.051	3.489	0.00078682	0.794652739	ns	+
293	cg08939828	chr5:6111860	open sea	AC026797.1	MED10			2.795	0.023	0.010	0.036	3.488	0.000787801	0.794652739	ns	+
294	cg01789980	chr5:167743144	open sea	WWC1	WWC1	Body		1.244	0.019	0.008	0.030	3.488	0.000788421	0.794652739	ns	+
295	cg10057799	chr6:8108921	open sea		EEF1E1			1.413	-0.028	-0.043	-0.012	-3.486	0.000793406	0.794652739	ns	-
296	cg25215292	chr5:135265753	N shore	FBXL21P	LECT2	TSS1500		-1.417	-0.021	-0.033	-0.009	-3.485	0.000795999	0.794652739	ns	-
297	cg05444524	chr16:88119777	open sea	BANP	BANP			2.214	-0.025	-0.039	-0.011	-3.484	0.000797794	0.794652739	ns	-
298	cg21515402	chr15:89991455	open sea		RHCG			1.981	0.032	0.014	0.051	3.484	0.000799049	0.794652739	ns	+
299	cg22792367	chr5:96267912	N shelf	CTD-2260A17.2	LNPEP			1.181	0.031	0.013	0.048	3.482	0.000804634	0.794652739	ns	+
300	cg14043602	chr17:3301340	open sea	OR1E1	OR1E1	1stExon		-1.408	-0.025	-0.040	-0.011	-3.481	0.000805174	0.794652739	ns	-
301	cg25538883	chr18:48191042	S shore	MAPK4	MAPK4	Body		2.164	-0.024	-0.038	-0.010	-3.481	0.000807393	0.794652739	ns	-
302	cg15906214	chr10:13571646	S shore	BEND7	BEND7			-0.675	0.030	0.013	0.047	3.480	0.000807972	0.794652739	ns	+
303	cg22802766	chr12:14361288	open sea		ATF7IP			2.455	0.022	0.009	0.035	3.480	0.000809299	0.794652739	ns	+
304	cg02002333	chr1:49146361	open sea	AGBL4	AGBL4	Body		2.689	-0.030	-0.047	-0.013	-3.478	0.000813454	0.794652739	ns	-
305	cg25257671	chr22:18531243	island		MICAL3			0.500	0.036	0.016	0.057	3.477	0.0008179	0.794652739	ns	+

SAQ: SOCIAL ACKNOWLEDGEMENT

rank	cpg	cpg.position	region	annotation	gene	gene.group	gene.feature	AveExpr	logFC	CI.L	CI.R	t	P.Value	adj.P.Val	fdr	sign
1	cg08806184	chr2:27068081	N shelf		DPYSL5			0.529	-0.013	-0.017	-0.009	-6.741	0.00000000	0.000639826	***	-
2	cg17104191	chr17:7190166	N shelf	SLC2A4	SLC2A4	3'UTR		-0.385	-0.018	-0.024	-0.012	-5.996	0.00000005	0.008138645	***	-
3	cg06964030	chr22:20073636	island	DGCR8	DGCR8	Body		0.971	0.012	0.008	0.017	5.821	0.00000011	0.010222247	**	+
4	cg07170253	chr12:48119903	open sea	ENDOU	ENDOU	TSS1500		-1.751	-0.021	-0.028	-0.014	-5.779	0.00000013	0.010222247	**	-
5	cg22990871	chr21:43377577	S shelf		C2CD2			-0.986	-0.011	-0.015	-0.007	-5.722	0.00000017	0.010390236	**	-
6	cg19499713	chr9:97767159	island	AOPEP	AOPEP	Body		-2.905	-0.022	-0.029	-0.014	-5.666	0.00000021	0.010947693	**	-
7	cg07699323	chr8:90987606	open sea	NBN	NBN	Body		-1.395	-0.020	-0.028	-0.013	-5.531	0.00000038	0.016479546	**	-
8	cg13335869	chr15:62457698	S shore	C2CD4B	C2CD4B	TSS1500		0.349	-0.013	-0.017	-0.008	-5.437	0.00000056	0.021248916	**	-
9	cg06953601	chr6:52441809	N shore	TRAM2	TRAM2	5'UTR	promoter-linked	-1.958	-0.017	-0.024	-0.011	-5.381	0.00000070	0.021367864	**	-
10	cg08582594	chr7:18330055	open sea	HDAC9	HDAC9	Body		-1.700	-0.021	-0.028	-0.013	-5.358	0.00000077	0.021367864	**	-
11	cg17072368	chr17:56004592	open sea	CUEDC1	CUEDC1			2.420	0.011	0.007	0.016	5.358	0.00000077	0.021367864	**	+
12	cg15507356	chr2:228452382	open sea		C2orf83			-0.602	-0.013	-0.017	-0.008	-5.331	0.00000085	0.021445655	**	-
13	cg20227763	chr1:151763931	island	TDRKH	TDRKH	TSS1500		-2.948	-0.022	-0.030	-0.014	-5.309	0.00000094	0.021445655	**	-
14	cg24969467	chr11:3686556	N shore	Y_RNA	CHRNA10			2.221	0.012	0.007	0.016	5.266	0.00000111	0.021445655	**	+
15	cg14682005	chr6:15337603	open sea	JARID2	JARID2	Body		-2.064	-0.013	-0.019	-0.008	-5.245	0.00000121	0.021445655	**	-
16	cg18616175	chr20:39321473	island		MAFB			-3.119	-0.020	-0.027	-0.012	-5.235	0.00000126	0.021445655	**	-
17	cg23411013	chr15:60868149	open sea	RORA	RORA	Body		-0.833	-0.012	-0.017	-0.008	-5.229	0.00000130	0.021445655	**	-
18	cg03336086	chr5:79331047	island	THBS4	THBS4	1stExon		-2.892	-0.013	-0.017	-0.008	-5.229	0.00000130	0.021445655	**	-
19	cg25673784	chr8:49470468	S shore	RP11-567J20.2	EFCAB1			0.635	-0.017	-0.023	-0.011	-5.213	0.00000138	0.021445655	**	-
20	cg09065654	chr3:183948302	island	VWA5B2	VWA5B2	TSS200		-2.007	-0.019	-0.026	-0.011	-5.209	0.00000140	0.021445655	**	-
21	cg21464724	chr21:46952851	S shore	SLC19A1	SLC19A1	Body	unknown cell specific	-1.459	-0.018	-0.025	-0.011	-5.183	0.00000156	0.021445655	**	-
22	cg09645475	chr20:815316	S shore	FAM110A	FAM110A	TSS1500	promoter-linked	-1.288	-0.017	-0.024	-0.011	-5.170	0.00000164	0.021445655	**	-
23	cg03384701	chr2:12643011	open sea	LOC100506457	TRIB2	Body		-0.815	-0.015	-0.021	-0.009	-5.166	0.00000167	0.021445655	**	-
24	cg22645574	chr7:1258449	open sea		UNCX			1.580	-0.012	-0.016	-0.007	-5.162	0.00000170	0.021445655	**	-
25	cg23478349	chr15:75492982	N shore	C15orf39	C15orf39	TSS1500		0.225	-0.009	-0.012	-0.005	-5.154	0.00000175	0.021445655	**	-
26	cg22654444	chr3:101645577	open sea		NFKBIZ			-3.365	0.021	0.013	0.029	5.138	0.00000187	0.022014422	**	+
27	cg10476247	chr7:100608642	N shore	MUC3A	MUC3A			-1.407	-0.015	-0.022	-0.009	-5.087	0.00000229	0.025943381	**	-
28	cg22112495	chr2:24406369	open sea	FAM228A	FAM228A	ExonBnd		3.133	0.009	0.006	0.013	5.053	0.00000263	0.027759823	**	+
29	cg16755863	chr2:130032641	open sea	AC079586.1	RAB6C			2.022	-0.012	-0.016	-0.007	-5.035	0.00000283	0.027759823	**	-
30	cg15152894	chr8:97174226	S shore	GDF6	GDF6	TSS1500		-0.404	-0.012	-0.016	-0.007	-5.025	0.00000294	0.027759823	**	-
31	cg25757140	chr19:38039469	N shore	CTD-3064H18.4	ZNF571			1.603	-0.027	-0.037	-0.016	-5.021	0.00000299	0.027759823	**	-
32	cg00748938	chr14:94641781	S shore	PPP4R4	PPP4R4	Body		-1.399	-0.024	-0.033	-0.014	-5.016	0.00000304	0.027759823	**	-
33	cg01123739	chr4:2398557	N shelf	ZFYVE28	ZFYVE28	Body	unknown cell specific	2.146	-0.017	-0.024	-0.010	-5.012	0.00000309	0.027759823	**	-
34	cg22153823	chr11:69868190	open sea	RP11-626H12.2	ANO1			0.543	-0.013	-0.019	-0.008	-5.007	0.00000315	0.027759823	**	-
35	cg02013741	chr15:67006698	open sea	SMAD6	SMAD6	Body		-0.425	-0.014	-0.019	-0.008	-5.005	0.00000318	0.027759823	**	-
36	cg13295050	chr15:30339875	S shelf		GOLGA8J			2.626	0.015	0.009	0.021	4.994	0.00000332	0.027942831	**	+
37	cg07313836	chr9:114898273	open sea	SUSD1	SUSD1	Body		2.363	0.014	0.008	0.019	4.987	0.00000341	0.027942831	**	+
38	cg25898963	chr22:27051344	N shore	MIAT	CRYBA4			2.346	0.014	0.009	0.020	4.983	0.00000347	0.027942831	**	+
39	cg18936814	chr15:93254934	N shelf	FAM174B	FAM174B			3.145	-0.017	-0.024	-0.010	-4.964	0.00000374	0.029095374	**	-
40	cg12492034	chr2:71933121	open sea		DYSF			2.026	-0.014	-0.020	-0.008	-4.960	0.00000380	0.029095374	**	-
41	cg07291349	chr4:40964962	open sea	APBB2	APBB2	Body		-0.544	-0.018	-0.025	-0.010	-4.935	0.00000419	0.030952229	**	-
42	cg24516901	chr2:225266801	open sea	FAM124B	FAM124B	TSS200	unknown cell specific	-1.756	-0.012	-0.017	-0.007	-4.932	0.00000425	0.030952229	**	-
43	cg08307612	chr20:62526785	S shore	DNAJC5	DNAJC5	5'UTR		-3.687	-0.024	-0.033	-0.014	-4.922	0.00000441	0.031335688	**	-
44	cg24690951	chr6:33822375	open sea		MLN			2.773	-0.017	-0.024	-0.010	-4.910	0.00000462	0.032114114	**	-
45	cg04386222	chr15:83368665	open sea	AP3B2	AP3B2	Body		1.263	-0.015	-0.020	-0.009	-4.887	0.00000506	0.034423155	**	-
46	cg23449231	chr5:179239690	N shelf	SQSTM1	SQSTM1	5'UTR		0.625	0.011	0.007	0.016	4.874	0.00000533	0.035412849	**	+
47	cg12384499	chr15:89949617	island	LOC105371031	RHCG		unknown cell specific	-2.193	-0.015	-0.021	-0.009	-4.863	0.00000556	0.035412849	**	-
48	cg02548350	chr3:123716746	open sea		ROPN1			2.598	-0.010	-0.014	-0.006	-4.855	0.00000574	0.035412849	**	-
49	cg14415833	chr2:217150663	open sea	MARCHF4	MARCHF4	Body		1.399	0.020	0.012	0.028	4.854	0.00000575	0.035412849	**	+
50	cg14176797	chr20:57426801	island	GNAS	GNAS	TSS1500		0.501	-0.008	-0.011	-0.005	-4.840	0.00000608	0.035412849	**	-
51	cg15808292	chr20:61825349	S shelf		YTHDF1			2.230	0.009	0.005	0.013	4.840	0.00000609	0.035412849	**	+
52	cg13399698	chr12:53443059	S shore	TNS2	TNS2	TSS1500	promoter-linked	2.376	-0.010	-0.014	-0.006	-4.839	0.00000610	0.035412849	**	-
53	cg25629796	chr17:11940467	open sea	MAP2K4	MAP2K4	Body		2.653	0.014	0.008	0.020	4.835	0.00000619	0.035412849	**	+
54	cg10740944	chr5:134367807	S shore	PITX1	PITX1	TSS1500	promoter-linked cell specific	-1.437	0.008	0.005	0.011	4.826	0.00000642	0.035412849	**	+
55	cg20129082	chr11:19736150	S shore	LOC100126784	NAV2	TSS200	unknown cell specific	-3.216	0.024	0.014	0.034	4.822	0.00000652	0.035412849	**	+
56	cg08485187	chr2:25500046	island	DNMT3A	DNMT3A	Body	promoter-linked	-0.041	-0.012	-0.018	-0.007	-4.821	0.00000655	0.035412849	**	-
57	cg04152196	chr6:136172687	open sea	PDE7B	PDE7B	TSS200		-2.908	-0.021	-0.029	-0.012	-4.819	0.00000661	0.035412849	**	-
58	cg20058506	chr2:72013035	island		DYSF			0.919	-0.010	-0.014	-0.006	-4.810	0.00000683	0.035412849	**	-
59	cg15916646	chr7:27225127	island	HOXA11	HOXA11	Body		-2.297	-0.010	-0.014	-0.006	-4.807	0.00000692	0.035412849	**	-
60	cg05196487	chr8:28929078	island	KIF13B	KIF13B	Body		2.257	0.011	0.006	0.015	4.806	0.00000695	0.035412849	**	+
61	cg11358114	chr2:45233390	S shore	SIX2	SIX2	Body		0.681	-0.010	-0.014	-0.006	-4.789	0.00000743	0.037243698	**	-
62	cg23437733	chr5:178017571	island	COL23A1	COL23A1	TSS200	promoter-linked	-3.582	-0.024	-0.034	-0.014	-4.781	0.00000768	0.037877839	**	-
63	cg03035653	chrX:99662932	island	PCDH19	PCDH19	1stExon		0.292	-0.010	-0.013	-0.006	-4.774	0.00000787	0.038218584	**	-
64	cg03730533	chr7:26897685	open sea	SKAP2	SKAP2	Body		-3.195	-0.020	-0.028	-0.012	-4.764	0.00000819	0.038425756	**	-
65	cg00064172	chr5:171602655	open sea	STK10	STK10	Body	promoter-linked	-1.522	-0.011	-0.016	-0.006	-4.763	0.00000819	0.038425756	**	-
66	cg17315014	chr5:138211155	open sea	CTNNA1	CTNNA1	Body		2.262	0.016	0.009	0.023	4.760	0.00000829	0.038425756	**	+
67	cg12869949	chr5:3116683	open sea		IRX2			-1.161	-0.008	-0.011	-0.005	-4.751	0.00000859	0.039214735	**	-
68	cg09451427	chr3:50488230	open sea	CACNA2D2	CACNA2D2	Body	unknown cell specific	-1.364	0.025	0.014	0.035	4.739	0.00000905	0.040714624	**	+
69	cg02242681	chr2:111876361	island	BCL2L11	BCL2L11			-1.198	-0.011	-0.016	-0.007	-4.727	0.00000943	0.041038164	**	-
70	cg11423680	chr5:72712836	N shelf		FOXD1			0.643	-0.011	-0.016	-0.006	-4.727	0.00000943	0.041038164	**	-
71	cg18038912	chr7:113857213	open sea	FOXP2	FOXP2	Body		-1.158	-0.020	-0.028	-0.012	-4.721	0.00000967	0.041038164	**	-
72	cg09376414	chr16:3135879	N shelf	RNU1-22P	ZSCAN10			1.616	-0.009	-0.013	-0.005	-4.719	0.00000973	0.041038164	**	-
73	cg10933827	chr7:92348349	open sea	CDK6	CDK6	Body		-1.430	-0.014	-0.020	-0.008	-4.717	0.00000980	0.041038164	**	-
74	cg12016218	chr8:4923996	open sea		CSMD1			0.937	-0.025	-0.036	-0.015	-4.709	0.00001014	0.041038164	**	-
75	cg19032370	chr1:93089381	open sea	EVI5	EVI5	Body		2.188	0.010	0.006	0.015	4.707	0.00001018	0.041038164	**	+

76	cg12171761	chr8:61910949	open sea		CHD7		unknown cell specific	0.155	0.011	0.006	0.016	4.705	0.00001027	0.041038164	**	+
77	cg24687529	chr1:117529954	open sea	PTGFRN	PTGFRN	3'UTR	unknown cell specific	0.440	-0.017	-0.025	-0.010	-4.703	0.00001036	0.041038164	**	-
78	cg15562860	chr6:131322683	open sea	EPB41L2	EPB41L2	TSS1500		1.930	0.010	0.006	0.014	4.700	0.00001047	0.041038164	**	+
79	cg19445230	chr7:99764904	N shelf	GAL3ST4	GAL3ST4	5'UTR		-2.543	-0.014	-0.020	-0.008	-4.696	0.00001063	0.041167746	**	-
80	cg27264462	chr1:2106365	open sea	PRKCZ	PRKCZ	Body		1.157	-0.014	-0.020	-0.008	-4.691	0.00001083	0.041417347	**	-
81	cg19085476	chr6:28234552	N shore	ZSCAN26	ZSCAN26	TSS1500		-2.559	0.017	0.010	0.024	4.683	0.00001118	0.042011325	**	+
82	cg11777105	chr12:46766728	S shore	SLC38A2	SLC38A2	TSS200	promoter-linked	-2.979	-0.017	-0.025	-0.010	-4.681	0.00001126	0.042011325	**	-
83	cg12673103	chr5:134364717	island	PITX1	PITX1	Body		-2.746	0.019	0.011	0.027	4.655	0.00001244	0.045449649	**	+
84	cg11963464	chr8:63976334	open sea	TTPA	TTPA	Body	unknown cell specific	-0.244	-0.015	-0.022	-0.009	-4.654	0.00001248	0.045449649	**	-
85	cg19464917	chr15:76627666	island	ISL2	ISL2	TSS1500		-3.271	-0.016	-0.023	-0.009	-4.649	0.00001272	0.045772132	**	-
86	cg03275611	chr20:33038001	open sea	ITCH	ITCH	Body		-1.170	-0.014	-0.020	-0.008	-4.617	0.00001440	0.050636825	*	-
87	cg14170976	chr20:826424	S shore	FAM110A	FAM110A	3'UTR	promoter-linked	-2.989	0.010	0.006	0.014	4.617	0.00001440	0.050636825	*	+
88	cg14729980	chr18:43415386	N shelf	SIGLEC15	SIGLEC15	Body		-0.400	-0.013	-0.019	-0.007	-4.597	0.00001553	0.053966133	*	-
89	cg13971556	chr3:177201705	open sea	LINC00578	TBL1XR1	Body		2.488	0.009	0.005	0.014	4.591	0.00001588	0.054022901	*	+
90	cg16543009	chr15:34331514	S shore	AVEN	AVEN	TSS1500		-1.961	-0.012	-0.018	-0.007	-4.591	0.00001590	0.054022901	*	-
91	cg25299676	chr7:116312739	S shore	MET	MET	5'UTR		-2.659	-0.012	-0.018	-0.007	-4.586	0.00001616	0.054331014	*	-
92	cg21064632	chr15:59268225	open sea	RNF111	RNF111			-1.126	-0.014	-0.020	-0.008	-4.575	0.00001688	0.056118145	*	-
93	cg18070753	chr7:73035779	N shore	MLXIPL	MLXIPL	Body		2.056	-0.014	-0.020	-0.008	-4.572	0.00001710	0.056246967	*	-
94	cg09675769	chr2:111977123	N shelf	MIR4435-2HG	BCL2L11		unknown cell specific	0.424	-0.016	-0.023	-0.009	-4.569	0.00001729	0.056271376	*	-
95	cg03190379	chr12:49690636	island	PRPH	PRPH	Body		-2.697	-0.021	-0.030	-0.012	-4.563	0.00001767	0.056900567	*	-
96	cg26655077	chr17:21227099	open sea		MAP2K3		promoter-linked	1.006	-0.010	-0.014	-0.005	-4.549	0.00001865	0.058745152	*	-
97	cg13994279	chr8:128993719	open sea	PVT1	MYC	Body	unknown cell specific	2.625	-0.016	-0.023	-0.009	-4.548	0.00001868	0.058745152	*	-
98	cg21199251	chr1:4828621	N shelf	AJAP1	AJAP1	Body		2.746	0.022	0.013	0.032	4.546	0.00001882	0.058745152	*	+
99	cg12853742	chr14:24780890	island	LTB4R	LTB4R	5'UTR	promoter-linked	0.266	0.011	0.006	0.016	4.540	0.00001924	0.059456845	*	+
100	cg10502220	chr4:176733966	open sea	GPM6A	GPM6A	1stExon		-0.091	-0.014	-0.020	-0.008	-4.533	0.00001975	0.060415518	*	-
101	cg16238026	chr7:30323735	N shore	ZNRF2	ZNRF2	TSS200		-1.780	0.012	0.007	0.017	4.526	0.00002029	0.06066217	*	+
102	cg02551646	chr2:216478293	open sea	LINC00607	FN1			1.559	0.009	0.005	0.013	4.523	0.00002058	0.06066217	*	+
103	cg24407078	chr18:20710886	open sea		CABLES1			-1.457	-0.016	-0.023	-0.009	-4.522	0.00002060	0.06066217	*	-
104	cg23923854	chr11:64660996	N shore	AP001187.9	ATG2A			0.190	-0.011	-0.016	-0.006	-4.521	0.00002073	0.06066217	*	-
105	cg15001981	chr21:45374284	open sea	AGPAT3	AGPAT3	5'UTR		0.529	0.009	0.005	0.013	4.516	0.00002107	0.06066217	*	+
106	cg01132471	chr1:53556482	N shore	SLC1A7	SLC1A7	Body		2.830	-0.012	-0.018	-0.007	-4.515	0.00002122	0.06066217	*	-
107	cg03970849	chr11:79148183	N shore	TENM4	TENM4	5'UTR		-1.751	0.009	0.005	0.012	4.512	0.00002141	0.06066217	*	+
108	cg22786748	chr6:31034042	open sea		HCG22			0.372	-0.011	-0.016	-0.006	-4.506	0.00002193	0.06066217	*	-
109	cg22159557	chr15:65067668	island	RBPMS2	RBPMS2	1stExon		-1.907	-0.023	-0.033	-0.013	-4.506	0.00002196	0.06066217	*	-
110	cg00101932	chr22:45097955	island	PRR5	PRR5	5'UTR	promoter-linked	-3.310	-0.016	-0.024	-0.009	-4.503	0.00002219	0.06066217	*	-
111	cg03525023	chr1:3003609	S shelf	PRDM16	PRDM16	Body		2.868	0.014	0.008	0.021	4.502	0.00002221	0.06066217	*	+
112	cg22729539	chr12:122519119	S shore	MLXIP	MLXIP	Body		-2.066	-0.014	-0.021	-0.008	-4.502	0.00002221	0.06066217	*	-
113	cg11090202	chr12:133021713	N shore		FBRSL1		unknown cell specific	-2.961	-0.017	-0.025	-0.010	-4.500	0.00002243	0.060711208	*	-
114	cg07668358	chr1:101001447	N shelf		GPR88			0.601	-0.013	-0.018	-0.007	-4.485	0.00002370	0.063208388	*	-
115	cg01485548	chr19:18284321	N shore	IFI30	IFI30	TSS1500	promoter-linked	-2.049	-0.014	-0.020	-0.008	-4.484	0.00002377	0.063208388	*	-
116	cg16322681	chr11:73115417	open sea	FAM168A	FAM168A	3'UTR	unknown cell specific	-1.032	-0.018	-0.026	-0.010	-4.481	0.00002405	0.063425674	*	-
117	cg19841369	chr14:64663928	open sea	SYNE2	SYNE2	Body		-1.037	-0.018	-0.027	-0.010	-4.477	0.00002448	0.063987205	*	-
118	cg04825336	chr7:129633291	open sea	RP11-306G20.1	ZC3HC1			2.036	0.013	0.007	0.018	4.471	0.00002497	0.064238301	*	+
119	cg22666015	chr2:233981885	open sea	INPP5D	INPP5D	Body		-1.546	-0.014	-0.021	-0.008	-4.471	0.00002499	0.064238301	*	-
120	cg03720617	chr16:47527034	open sea	PHKB	PHKB	Body	unknown cell specific	-1.460	-0.014	-0.020	-0.008	-4.466	0.00002551	0.064341607	*	-
121	cg06182390	chr11:129838947	open sea	PRDM10	PRDM10	5'UTR		-2.219	-0.013	-0.019	-0.007	-4.464	0.00002567	0.064341607	*	-
122	cg23323745	chrX:47696132	island	ZNF81	ZNF81	TSS200	promoter-linked	-0.308	-0.010	-0.015	-0.006	-4.460	0.00002607	0.064341607	*	-
123	cg15248777	chr18:9334699	N shore	TWSG1	TWSG1	TSS200	promoter-linked	-0.740	-0.045	-0.065	-0.025	-4.470	0.00002618	0.064341607	*	-
124	cg20741677	chr8:97198983	open sea		GDF6		unknown cell specific	1.694	-0.019	-0.027	-0.010	-4.458	0.00002623	0.064341607	*	-
125	cg05785989	chr10:3306812	open sea	RP11-195B3.1	PITRM1			0.544	-0.011	-0.016	-0.006	-4.454	0.00002665	0.064341607	*	-
126	cg12032655	chr19:43856746	open sea	CD177	CD177	TSS1500		-1.614	-0.022	-0.032	-0.012	-4.453	0.00002673	0.064341607	*	-
127	cg16547579	chr20:4954333	open sea	SLC23A2	SLC23A2	5'UTR		-1.578	-0.014	-0.021	-0.008	-4.451	0.00002696	0.064341607	*	-
128	cg19159825	chr4:106395585	S shore	PPA2	PPA2	TSS1500	promoter-linked	2.162	-0.013	-0.018	-0.007	-4.447	0.00002738	0.064341607	*	-
129	cg17863551	chr19:43856594	open sea	CD177	CD177	TSS1500		-1.799	-0.013	-0.019	-0.007	-4.446	0.00002745	0.064341607	*	-
130	cg03475086	chrX:148653650	open sea		MAGEA9B			1.639	-0.015	-0.022	-0.008	-4.446	0.00002748	0.064341607	*	-
131	cg21340500	chr8:81478334	open sea		ZBTB10		unknown cell specific	-1.076	-0.016	-0.022	-0.009	-4.442	0.00002788	0.064341607	*	-
132	cg03920442	chr20:25848969	open sea	FAM182B	ZNF337		unknown cell specific	-3.403	-0.016	-0.023	-0.009	-4.441	0.00002797	0.064341607	*	-
133	cg08070022	chr3:101497478	N shore	NXPE3	NXPE3	TSS1500	promoter-linked	-3.307	-0.011	-0.016	-0.006	-4.439	0.00002813	0.064341607	*	-
134	cg10927461	chr2:122530159	open sea		TSN			-1.982	-0.011	-0.016	-0.006	-4.437	0.00002836	0.064341607	*	-
135	cg09607085	chr5:153667757	open sea	GALNT10	GALNT10	Body		2.746	0.010	0.005	0.014	4.437	0.00002840	0.064341607	*	+
136	cg17854886	chr14:89953623	open sea	FOXN3	FOXN3	5'UTR		-1.169	-0.015	-0.021	-0.008	-4.434	0.00002868	0.064424026	*	-
137	cg11206167	chr5:42924367	island		PRELID3BP6			2.718	-0.032	-0.046	-0.018	-4.436	0.00002904	0.064424026	*	-
138	cg07140290	chr16:3014186	island	KREMEN2	KREMEN2	TSS200		-1.412	-0.017	-0.024	-0.009	-4.431	0.00002907	0.064424026	*	-
139	cg24554720	chr2:48579956	open sea	FOXN2	FOXN2	Body		-0.410	-0.013	-0.019	-0.007	-4.426	0.00002953	0.06484641	*	-
140	cg19086156	chr7:70049420	open sea	AUTS2	AUTS2	Body		2.499	-0.017	-0.025	-0.009	-4.423	0.00002991	0.06484641	*	-
141	cg08474396	chr16:27237656	open sea	NSMCE1	NSMCE1	Body		2.197	0.010	0.005	0.014	4.420	0.00003026	0.06484641	*	+
142	cg12396381	chr13:24472378	open sea	C1QTNF9B	C1QTNF9B	TSS1500		1.158	0.006	0.003	0.009	4.419	0.00003031	0.06484641	*	+
143	cg15827559	chr3:99816799	open sea	FILIP1L	FILIP1L	5'UTR		2.455	0.008	0.004	0.011	4.419	0.00003039	0.06484641	*	+
144	cg14454157	chr7:70384700	open sea		AUTS2			-0.449	-0.009	-0.013	-0.005	-4.417	0.00003053	0.06484641	*	-
145	cg24813180	chr19:49065229	S shelf	SULT2B1	SULT2B1	Body		1.594	0.010	0.006	0.015	4.414	0.00003087	0.06487149	*	+
146	cg10084644	chr7:99775521	island	STAG3	STAG3	TSS200	promoter-linked	-3.504	-0.021	-0.031	-0.012	-4.414	0.00003097	0.06487149	*	-
147	cg13718972	chr15:101734560	open sea	CHSY1	CHSY1	Body		-0.369	-0.013	-0.019	-0.007	-4.404	0.00003207	0.066021352	*	-
148	cg02845661	chr3:192617487	open sea	MB21D2	MB21D2	Body		2.136	-0.013	-0.019	-0.007	-4.404	0.00003209	0.066021352	*	-
149	cg00260448	chrX:30907780	S shore	TAB3	TAB3	TSS1500	promoter-linked	-1.778	-0.016	-0.023	-0.009	-4.403	0.00003219	0.066021352	*	-
150	cg16067079	chr9:133708845	N shore	ABL1	ABL1	Body	unknown cell specific	2.668	0.014	0.008	0.021	4.402	0.00003238	0.066021352	*	+
151	cg21486465	chr7:89841028	island	STEAP2	STEAP2	TSS200		-3.694	-0.024	-0.035	-0.013	-4.399	0.00003285	0.066111125	*	-
152	cg24683984	chr9:71613680	open sea	PIP5K1B	PIP5K1B	Body		-0.233	-0.012	-0.017	-0.006	-4.396	0.00003309	0.066111125	*	-

153	cg24736345	chr19:3093246	N shore	GNA11	GNA11	TSS1500		2.624	0.022	0.012	0.032	4.395	0.00003324	0.066111125	*	+
154	cg24948887	chr18:74859463	open sea		MBP			0.417	-0.018	-0.026	-0.010	-4.394	0.00003329	0.066111125	*	-
155	cg16062531	chr14:94404163	N shore	ASB2	ASB2	Body		2.362	0.013	0.007	0.019	4.390	0.00003378	0.066111125	*	+
156	cg18772205	chr3:183236384	open sea	KLHL6	KLHL6	Body	unknown cell specific	-0.910	-0.011	-0.016	-0.006	-4.390	0.00003378	0.066111125	*	-
157	cg27628372	chr18:20686693	open sea		CABLES1			-1.227	-0.009	-0.013	-0.005	-4.389	0.00003393	0.066111125	*	-
158	cg02354259	chr20:60489114	open sea	CDH4	CDH4	Body		1.751	-0.012	-0.018	-0.007	-4.387	0.00003421	0.066225884	*	-
159	cg08540488	chr2:224403622	open sea		SCG2			-1.845	-0.015	-0.022	-0.008	-4.383	0.00003472	0.066531973	*	-
160	cg07621226	chr3:177149819	open sea		TBL1XR1			0.554	0.010	0.006	0.015	4.382	0.00003481	0.066531973	*	+
161	cg20111726	chr10:101543950	open sea	ABCC2	ABCC2	Body		1.564	-0.016	-0.023	-0.009	-4.379	0.00003525	0.066531973	*	-
162	cg17345450	chr6:166259938	open sea	PDE10A	PDE10A			1.808	-0.014	-0.020	-0.008	-4.377	0.00003542	0.066531973	*	-
163	cg26172504	chr19:51487478	open sea	KLK7	KLK7	TSS1500		-1.225	-0.019	-0.028	-0.011	-4.371	0.00003625	0.066531973	*	-
164	cg08211405	chr4:122589621	open sea	ANXA5	ANXA5	3'UTR		2.134	0.012	0.007	0.018	4.370	0.00003645	0.066531973	*	+
165	cg27049751	chr12:76425578	island	PHLDA1	PHLDA1	TSS200	promoter-linked	-2.816	-0.020	-0.028	-0.011	-4.369	0.00003660	0.066531973	*	-
166	cg22218464	chr15:83329551	open sea	CPEB1	CPEB1	Body		2.169	-0.009	-0.014	-0.005	-4.368	0.00003672	0.066531973	*	-
167	cg13085760	chr14:90871617	open sea	CALM1	CALM1	3'UTR		2.945	-0.008	-0.011	-0.004	-4.368	0.00003674	0.066531973	*	-
168	cg02115488	chr3:138009845	open sea	ARMC8	ARMC8	Body		1.496	0.010	0.006	0.015	4.367	0.00003679	0.066531973	*	+
169	cg22329088	chr10:85945950	open sea		C10orf99			2.483	-0.011	-0.017	-0.006	-4.367	0.00003680	0.066531973	*	-
170	cg23903396	chr11:70487842	open sea	SHANK2	SHANK2	Body		-0.724	-0.012	-0.017	-0.007	-4.366	0.00003698	0.066531973	*	-
171	cg00228496	chr2:85875472	open sea	USP39	USP39	3'UTR	gene-linked	0.062	-0.011	-0.016	-0.006	-4.363	0.00003736	0.066832006	*	-
172	cg19763809	chr2:98703475	island	VWA3B	VWA3B	TSS200		-2.087	-0.011	-0.016	-0.006	-4.360	0.00003773	0.067089724	*	-
173	cg13435432	chr6:108489292	island	NR2E1	NR2E1	Body		-1.701	-0.008	-0.011	-0.004	-4.358	0.00003812	0.067398882	*	-
174	cg07295362	chr2:3061128	N shelf	LINC01250	EIPR1			-1.777	-0.014	-0.020	-0.007	-4.355	0.00003852	0.067705926	*	-
175	cg12954529	chr8:32504114	open sea	NRG1	NRG1	TSS200		-1.419	-0.009	-0.013	-0.005	-4.352	0.00003894	0.06805506	*	-
176	cg15167955	chr11:61309917	open sea	SYT7	SYT7	Body		2.087	-0.013	-0.020	-0.007	-4.348	0.00003949	0.068353414	*	-
177	cg20315150	chr7:28647577	open sea	CREB5	CREB5	Body	unknown cell specific	-0.589	-0.011	-0.016	-0.006	-4.348	0.00003955	0.068353414	*	-
178	cg08535112	chr10:61418311	open sea	SLC16A9	SLC16A9	Body		2.877	0.014	0.008	0.021	4.344	0.00004006	0.068684426	*	+
179	cg22203219	chr12:6665335	island	IFFO1	IFFO1	TSS200	promoter-linked	-1.392	-0.012	-0.018	-0.007	-4.343	0.00004020	0.068684426	*	-
180	cg19953406	chr19:51487360	open sea	KLK7	KLK7	TSS1500		-2.657	-0.027	-0.040	-0.015	-4.343	0.00004072	0.068994995	*	-
181	cg21679510	chr2:136738377	open sea	DARS1	DARS1	5'UTR		-0.847	-0.018	-0.026	-0.010	-4.339	0.00004083	0.068994995	*	-
182	cg05649708	chr10:131771695	island		EBF3			-1.837	-0.018	-0.026	-0.010	-4.336	0.00004131	0.069304639	*	-
183	cg06387516	chr1:239568713	open sea	CHRM3	CHRM3			0.428	-0.021	-0.031	-0.011	-4.335	0.00004146	0.069304639	*	-
184	cg13675721	chr7:140227273	open sea	DENND2A	DENND2A	Body		2.788	0.018	0.010	0.027	4.326	0.00004285	0.071237546	*	+
185	cg21634189	chr10:6743484	open sea		LINP1			3.030	-0.014	-0.020	-0.008	-4.323	0.00004331	0.071586331	*	-
186	cg19944059	chr16:66062646	open sea		CDH5			-1.946	-0.017	-0.024	-0.009	-4.321	0.00004366	0.071586331	*	-
187	cg00411544	chr2:74375128	island	BOLA3	BOLA3	TSS200	promoter-linked	-2.425	-0.020	-0.029	-0.011	-4.320	0.00004377	0.071586331	*	-
188	cg19738386	chr2:136288977	island	R3HDM1	R3HDM1	TSS200	promoter-linked	-3.518	-0.020	-0.030	-0.011	-4.319	0.00004404	0.071635426	*	-
189	cg25987208	chr10:8079618	S shore		GATA3-AS1		unknown cell specific	-2.731	-0.018	-0.026	-0.010	-4.317	0.00004426	0.071635426	*	-
190	cg16308565	chr2:118774529	S shelf	AC009303.1	CCDC93			-0.391	-0.018	-0.027	-0.010	-4.315	0.00004465	0.071873883	*	-
191	cg18174542	chr22:41956861	open sea	CSDC2	CSDC2	TSS200		-1.278	-0.016	-0.024	-0.009	-4.311	0.00004523	0.072385177	*	-
192	cg24158363	chr17:73401717	island	GRB2	GRB2	5'UTR	promoter-linked	-0.806	-0.012	-0.017	-0.006	-4.310	0.00004544	0.072385177	*	-
193	cg03313290	chr5:53448485	open sea	ARL15	ARL15	Body		2.396	0.012	0.006	0.017	4.306	0.00004607	0.07301004	*	+
194	cg20992785	chr6:168533507	S shelf		FRMD1		unknown cell specific	-0.221	-0.023	-0.034	-0.012	-4.304	0.00004654	0.073379007	*	-
195	cg07528595	chr12:54814557	S shore	ITGA5	ITGA5			-1.026	-0.011	-0.015	-0.006	-4.298	0.00004756	0.074380434	*	-
196	cg23761878	chr22:48841452	open sea		TAFA5			-0.130	-0.008	-0.011	-0.004	-4.297	0.00004766	0.074380434	*	-
197	cg13014803	chr13:112212253	island		TEX29			-3.160	-0.012	-0.018	-0.007	-4.289	0.00004905	0.074960187	*	-
198	cg02649547	chr10:95848791	open sea	PLCE1	PLCE1	TSS200		-2.594	-0.014	-0.020	-0.007	-4.289	0.00004905	0.074960187	*	-
199	cg10542197	chr6:151246895	open sea	MTHFD1L	MTHFD1L	Body		-0.586	-0.012	-0.017	-0.006	-4.289	0.00004906	0.074960187	*	-
200	cg19351586	chr17:15141677	open sea	PMP22	PMP22	Body		-1.595	-0.012	-0.018	-0.007	-4.289	0.00004914	0.074960187	*	-
201	cg07736115	chr13:113771937	island	F7	F7	Body		2.763	0.011	0.006	0.016	4.288	0.00004926	0.074960187	*	+
202	cg06305962	chr7:76956986	open sea	GSAP	GSAP	Body		1.416	-0.021	-0.031	-0.011	-4.286	0.00004972	0.075293138	*	-
203	cg17920241	chrX:148586978	island	IDS	IDS	TSS200		-0.362	-0.008	-0.012	-0.004	-4.284	0.00005006	0.075424627	*	-
204	cg13748577	chr7:148787770	island	ZNF786	ZNF786	5'UTR	promoter-linked	-3.691	-0.021	-0.031	-0.011	-4.278	0.00005114	0.076484032	*	-
205	cg01701649	chr16:48589954	open sea	N4BP1	N4BP1	Body		-2.116	-0.016	-0.024	-0.009	-4.277	0.00005126	0.076484032	*	-
206	cg08455440	chr16:32183398	open sea	HERC2P4	TP53TG3D	Body		1.766	-0.050	-0.073	-0.027	-4.286	0.00005159	0.076576403	*	-
207	cg16316054	chr4:185480838	open sea	LOC100129245	CASP3			3.344	0.014	0.008	0.021	4.274	0.00005193	0.076576403	*	+
208	cg03559817	chr5:135468621	island	SMAD5	SMAD5	1stExon	promoter-linked	-2.575	-0.012	-0.018	-0.006	-4.272	0.00005218	0.076576403	*	-
209	cg11918822	chr19:50479865	open sea	VRK3	VRK3	3'UTR		2.046	-0.013	-0.019	-0.007	-4.272	0.00005232	0.076576403	*	-
210	cg02374934	chr15:41228736	island	DLL4	DLL4	Body		1.287	-0.009	-0.014	-0.005	-4.270	0.00005264	0.076597423	*	-
211	cg17215468	chr3:111652058	open sea	PHLDB2	PHLDB2	Body		-1.371	-0.010	-0.015	-0.005	-4.266	0.00005331	0.076597423	*	-
212	cg16181678	chrX:119378716	island	NKAPP1	ZBTB33	Body	promoter-linked	0.960	-0.007	-0.011	-0.004	-4.265	0.00005354	0.076597423	*	-
213	cg22375663	chr12:69725435	open sea	RP11-1143G9.5	LYZ			-2.848	-0.018	-0.026	-0.010	-4.265	0.00005354	0.076597423	*	-
214	cg24607140	chr20:30134973	N shore	HM13	HM13	Body	promoter-linked	1.023	-0.009	-0.013	-0.005	-4.265	0.00005359	0.076597423	*	-
215	cg07036561	chr15:42118869	N shore	JMJD7	JMJD7	TSS1500	unknown cell specific	2.439	0.010	0.005	0.015	4.261	0.00005443	0.076944515	*	+
216	cg14335069	chr22:41956810	open sea	CSDC2	CSDC2	TSS1500		-2.198	-0.014	-0.020	-0.007	-4.260	0.00005457	0.076944515	*	-
217	cg04291946	chr20:60791310	island	HRH3	HRH3	Body		1.342	0.012	0.007	0.018	4.260	0.00005459	0.076944515	*	+
218	cg07107661	chr14:97877297	open sea		VRK1			0.317	-0.014	-0.020	-0.007	-4.257	0.00005526	0.077358156	*	-
219	cg11661234	chrX:73834596	island	RLIM	RLIM	TSS200	promoter-linked	-0.207	-0.009	-0.013	-0.005	-4.256	0.00005539	0.077358156	*	-
220	cg26187342	chr17:65713858	island	NOL11	NOL11	TSS1500	promoter-linked	-0.316	-0.010	-0.015	-0.005	-4.254	0.00005576	0.07752564	*	-
221	cg25303955	chr15:80883267	open sea	ARNT2	ARNT2	Body		0.822	-0.013	-0.019	-0.007	-4.251	0.00005634	0.077968737	*	-
222	cg13610072	chr4:89619085	open sea	NAP1L5	NAP1L5	TSS200	promoter-linked	1.238	-0.009	-0.014	-0.005	-4.250	0.00005665	0.078052862	*	-
223	cg18660278	chr19:6166026	open sea	ACSBG2	ACSBG2	Body	unknown cell specific	0.457	-0.014	-0.021	-0.008	-4.247	0.00005714	0.07836973	*	-
224	cg08562099	chr17:55389809	open sea	MSI2	MSI2	Body		-1.852	-0.013	-0.020	-0.007	-4.242	0.00005829	0.079434567	*	-
225	cg06932104	chr18:13457462	open sea	LDLRAD4	LDLRAD4	Body		-1.017	-0.010	-0.014	-0.005	-4.239	0.00005903	0.079434567	*	-
226	cg18912768	chr2:169887819	open sea	ABCB11	ABCB11	5'UTR		0.742	-0.013	-0.019	-0.007	-4.238	0.00005910	0.079434567	*	-
227	cg12697442	chr11:101981167	island	YAP1	YAP1	TSS200		-0.544	-0.022	-0.032	-0.012	-4.236	0.00005961	0.079434567	*	-
228	cg15255408	chr16:58299645	open sea	CCDC113	CCDC113	Body		2.187	-0.019	-0.028	-0.010	-4.235	0.00005972	0.079434567	*	-
229	cg14011845	chr17:59533635	island	TBX4	TBX4	TSS200		0.956	-0.011	-0.016	-0.006	-4.235	0.00005975	0.079434567	*	-

230	cg19760211	chr10:329101	open sea	MIR7641-2	DIP2C	TSS1500		1.604	-0.010	-0.015	-0.005	-4.234	0.00006007	0.079434567	*	-
231	cg00621608	chr1:160040173	island	KCNJ10	KCNJ10	TSS200		-3.095	-0.010	-0.015	-0.005	-4.233	0.00006027	0.079434567	*	-
232	cg24504194	chr6:31527920	open sea	LOC100287329	NFKBIL1			-1.433	-0.018	-0.027	-0.010	-4.233	0.00006032	0.079434567	*	-
233	cg09503954	chr10:50565220	open sea		DRGX			2.443	0.011	0.006	0.017	4.231	0.00006060	0.079434567	*	+
234	cg02780388	chr5:142985157	open sea	CTB-57H20.1	NR3C1			-2.003	-0.014	-0.020	-0.007	-4.230	0.00006091	0.079434567	*	-
235	cg04755227	chr16:88449254	N shore		ZNF469		unknown cell specific	-2.541	-0.012	-0.017	-0.006	-4.229	0.00006103	0.079434567	*	-
236	cg05085585	chr16:30420623	S shore	ZNF771	ZNF771	Body		-0.874	0.016	0.009	0.024	4.228	0.00006133	0.0794918	*	+
237	cg20536364	chr5:55790102	open sea		RPL26P19			1.712	-0.015	-0.022	-0.008	-4.224	0.00006218	0.0797457	*	-
238	cg06258834	chr19:51487136	open sea	KLK7	KLK7	5'UTR		-1.511	-0.016	-0.023	-0.008	-4.224	0.00006220	0.0797457	*	-
239	cg09357934	chr2:159651918	open sea	DAPL1	DAPL1	1stExon		0.070	0.016	0.009	0.024	4.224	0.00006232	0.0797457	*	+
240	cg03965649	chr8:134576933	open sea	ST3GAL1	ST3GAL1	5'UTR		2.436	0.015	0.008	0.022	4.219	0.00006339	0.0797457	*	+
241	cg01399214	chr16:70524458	open sea	COG4	COG4	Body		-1.411	-0.009	-0.013	-0.005	-4.219	0.00006341	0.0797457	*	-
242	cg09890286	chr17:77088710	open sea	RBFOX3	RBFOX3	Body		2.746	-0.014	-0.021	-0.007	-4.219	0.00006342	0.0797457	*	-
243	cg23581009	chr3:50310506	island	SEMA3B	SEMA3B	Body		-2.930	-0.013	-0.019	-0.007	-4.217	0.00006385	0.0797457	*	-
244	cg06751366	chr16:50280723	S shore	ADCY7	ADCY7		promoter-linked	-2.247	-0.013	-0.019	-0.007	-4.217	0.00006387	0.0797457	*	-
245	cg07938743	chr2:63283939	island	OTX1	OTX1	3'UTR		0.276	-0.015	-0.021	-0.008	-4.216	0.00006413	0.0797457	*	-
246	cg08831034	chr2:242138408	N shore	ANO7	ANO7	Body		1.033	0.007	0.004	0.011	4.213	0.00006488	0.0797457	*	+
247	cg26221928	chr1:27701067	open sea	FCN3	FCN3	Body		2.628	-0.012	-0.018	-0.007	-4.213	0.00006488	0.0797457	*	-
248	cg05018520	chr3:185977298	open sea	DGKG	DGKG	Body		0.749	-0.019	-0.028	-0.010	-4.211	0.00006520	0.0797457	*	-
249	cg02524205	chr6:167559851	open sea	TCP10L2	CCR6			0.383	-0.030	-0.044	-0.016	-4.216	0.00006525	0.0797457	*	-
250	cg16238635	chr10:100932213	open sea	HPSE2	HPSE2	Body		0.508	-0.011	-0.016	-0.006	-4.208	0.00006591	0.0797457	*	-
251	cg00918917	chr17:61819349	island	STRADA	STRADA	TSS200	promoter-linked	-2.973	-0.015	-0.023	-0.008	-4.208	0.00006595	0.0797457	*	-
252	cg05354329	chr8:37854308	open sea		ADRB3			-1.248	-0.011	-0.016	-0.006	-4.208	0.00006602	0.0797457	*	-
253	cg13672743	chr17:33417086	S shore	RFFL	RFFL	TSS1500	promoter-linked	-2.667	-0.013	-0.019	-0.007	-4.206	0.00006645	0.0797457	*	-
254	cg12495996	chr10:132239568	open sea	RP11-540N6.1	GLRX3			3.455	-0.016	-0.023	-0.008	-4.206	0.00006655	0.0797457	*	-
255	cg04438911	chr1:26346675	open sea	EXTL1	EXTL1			2.198	-0.010	-0.015	-0.005	-4.205	0.00006663	0.0797457	*	-
256	cg08732418	chr15:41228676	island	DLL4	DLL4	Body		1.687	-0.008	-0.012	-0.004	-4.205	0.00006674	0.0797457	*	-
257	cg21046355	chr19:52772717	island	ZNF766	ZNF766	TSS200	non-gene-linked	-3.263	-0.014	-0.021	-0.007	-4.200	0.00006790	0.080081883	*	-
258	cg23965861	chr5:10705332	open sea	DAP	DAP	Body		-1.297	-0.011	-0.017	-0.006	-4.199	0.00006824	0.080081883	*	-
259	cg12498181	chr13:113436309	N shelf	ATP11A	ATP11A	Body		1.796	0.010	0.005	0.014	4.198	0.00006849	0.080081883	*	+
260	cg14433497	chr15:76634516	island	ISL2	ISL2	3'UTR		-3.708	-0.017	-0.025	-0.009	-4.197	0.00006870	0.080081883	*	-
261	cg15413430	chr3:122017315	open sea		CASR		unknown cell specific	-1.864	-0.015	-0.022	-0.008	-4.196	0.00006899	0.080081883	*	-
262	cg05006412	chr8:41386263	N shore	GINS4	GINS4	TSS1500	promoter-linked	0.063	-0.008	-0.012	-0.004	-4.195	0.00006905	0.080081883	*	-
263	cg11946445	chr7:37264255	open sea	ELMO1	ELMO1	Body		2.322	0.009	0.005	0.013	4.195	0.00006909	0.080081883	*	+
264	cg13015534	chr17:74639793	open sea	ST6GALNAC1	ST6GALNAC1	5'UTR	unknown cell specific	0.357	-0.018	-0.027	-0.010	-4.194	0.00006935	0.080081883	*	-
265	cg17392047	chr11:65247138	S shore		MALAT1			0.636	0.016	0.008	0.023	4.194	0.00006944	0.080081883	*	+
266	cg05018671	chr8:27146760	S shore	TRIM35	TRIM35	Body		2.554	0.012	0.006	0.018	4.193	0.00006964	0.080081883	*	+
267	cg13614383	chr9:91762376	open sea	SHC3	SHC3	Body		2.633	-0.012	-0.018	-0.006	-4.190	0.00007033	0.080570958	*	-
268	cg08201755	chr14:74960113	island	NPC2	NPC2	TSS200	promoter-linked	-3.379	-0.015	-0.022	-0.008	-4.182	0.00007248	0.082721685	*	-
269	cg20173524	chr6:127439996	island	RSPO3	RSPO3	TSS200		-1.360	-0.016	-0.024	-0.008	-4.178	0.00007361	0.083701065	*	-
270	cg23333125	chr7:2803065	S shore	GNA12	GNA12	TSS1500		1.486	0.011	0.006	0.017	4.176	0.00007407	0.08387698	*	+
271	cg15334372	chr16:73096889	island		ZFHX3			-0.414	-0.012	-0.017	-0.006	-4.175	0.00007432	0.08387698	*	-
272	cg04685228	chr5:172462626	open sea		ATP6V0E1		promoter-linked	-1.357	-0.018	-0.026	-0.009	-4.171	0.00007529	0.084668681	*	-
273	cg03324961	chr8:23605601	open sea	RP11-175E9.1	NKX2-6			-2.036	-0.036	-0.054	-0.019	-4.178	0.00007561	0.084707811	*	-
274	cg15493035	chr10:116659330	open sea	FAM160B1	FAM160B1	3'UTR		2.894	0.011	0.006	0.016	4.169	0.00007589	0.084716564	*	+
275	cg01392867	chr16:30410429	N shore	ZNF48	ZNF48	3'UTR		2.560	0.009	0.005	0.014	4.166	0.00007673	0.085337675	*	+
276	cg15611565	chr1:165880424	open sea	UCK2	UCK2	3'UTR		2.027	0.012	0.006	0.018	4.165	0.00007704	0.085375385	*	+
277	cg01486943	chr3:58621258	open sea	FAM3D	FAM3D	Body		2.899	-0.019	-0.028	-0.010	-4.161	0.00007833	0.086121951	*	-
278	cg18377014	chr7:1095595	N shore	C7orf50	C7orf50	Body		-0.788	-0.009	-0.013	-0.005	-4.158	0.00007907	0.086121951	*	-
279	cg04388383	chrX:64754908	S shore	LAS1L	LAS1L	TSS1500	promoter-linked	-0.169	-0.011	-0.016	-0.006	-4.157	0.00007939	0.086121951	*	-
280	cg03031357	chrX:21959700	island	SMS	SMS	Body	promoter-linked	0.959	-0.014	-0.021	-0.007	-4.155	0.00007976	0.086121951	*	-
281	cg08546514	chr20:25506526	open sea	NINL	NINL	Body		0.937	0.025	0.013	0.037	4.157	0.00007984	0.086121951	*	+
282	cg02966971	chr14:51836760	open sea	RP11-255G12.2	TMX1			0.400	-0.012	-0.018	-0.007	-4.155	0.00007986	0.086121951	*	-
283	cg16654771	chr6:159239474	island	EZR	EZR	TSS200	promoter-linked	-3.043	-0.013	-0.020	-0.007	-4.154	0.00008009	0.086121951	*	-
284	cg05288798	chr2:64829254	open sea		AFTPH			1.647	0.007	0.004	0.011	4.154	0.00008018	0.086121951	*	+
285	cg16246169	chr3:181412965	N shore	SOX2	SOX2	Body		2.025	-0.014	-0.020	-0.007	-4.152	0.00008066	0.086121951	*	-
286	cg26348487	chr19:2488560	island		GADD45B			-4.188	-0.023	-0.033	-0.012	-4.152	0.00008081	0.086121951	*	-
287	cg20797320	chr13:45793491	open sea	GTF2F2	GTF2F2	Body		-0.153	-0.016	-0.024	-0.008	-4.150	0.00008122	0.086121951	*	-
288	cg14880539	chr4:10973873	open sea		CLNK			1.501	0.011	0.005	0.016	4.150	0.00008127	0.086121951	*	+
289	cg20832910	chr22:42792851	open sea	NFAM1	NFAM1	Body	unknown cell specific	0.571	-0.019	-0.027	-0.010	-4.150	0.00008137	0.086121951	*	-
290	cg13359161	chr4:124552897	open sea		SPRY1			1.959	0.013	0.007	0.019	4.147	0.00008234	0.086845421	*	+
291	cg23128435	chr5:110419062	open sea	CTC-551A13.2	TSLP			-1.172	-0.013	-0.019	-0.007	-4.145	0.00008290	0.087139747	*	-
292	cg17060274	chr18:51795712	island	POLI	POLI	TSS200	promoter-linked	-2.672	-0.019	-0.028	-0.010	-4.143	0.00008333	0.087216722	*	-
293	cg08893449	chr6:35310274	island	PPARD	PPARD	TSS200	promoter-linked	-1.768	-0.015	-0.023	-0.008	-4.143	0.00008355	0.087216722	*	-
294	cg11113392	chr17:8860140	open sea	PIK3R5	PIK3R5	5'UTR		2.295	0.016	0.008	0.023	4.141	0.00008401	0.087396658	*	+
295	cg12170419	chr20:23367377	open sea	NAPB	NAPB	Body		1.306	-0.016	-0.023	-0.008	-4.139	0.00008452	0.087537192	*	-
296	cg25880958	chr11:74394337	N shore		CHRDL2			0.181	-0.014	-0.021	-0.007	-4.138	0.00008502	0.087537192	*	-
297	cg17393568	chr4:190861806	island	FRG1	FRG1	TSS200		-2.444	-0.017	-0.026	-0.009	-4.137	0.00008512	0.087537192	*	-
298	cg01243968	chr4:87515433	island	PTPN13	PTPN13	TSS200	promoter-linked	-2.975	-0.015	-0.021	-0.008	-4.137	0.00008529	0.087537192	*	-
299	cg19659215	chr12:21488180	open sea	SLCO1A2	SLCO1A2	5'UTR		1.384	0.008	0.004	0.012	4.136	0.00008568	0.087650544	*	+
300	cg20797151	chr2:228804111	open sea		DAW1			2.636	-0.019	-0.029	-0.010	-4.133	0.00008654	0.08813479	*	-
301	cg27002516	chr12:111835267	S shore	RP3-473L9.4	SH2B3			-4.290	-0.016	-0.024	-0.008	-4.132	0.00008673	0.08813479	*	-
302	cg23076960	chr14:50428577	open sea	RP11-58E21.4	ARF6			2.527	0.014	0.007	0.021	4.131	0.00008702	0.08813479	*	+
303	cg22253329	chr13:108451762	open sea	FAM155A	FAM155A	Body		0.169	-0.013	-0.020	-0.007	-4.126	0.00008860	0.089439384	*	-
304	cg00591421	chr10:106468736	open sea	SORCS3	SORCS3	Body		-0.154	-0.011	-0.016	-0.006	-4.125	0.00008900	0.089456718	*	-
305	cg20011601	chr12:124067800	N shore	TMED2	TMED2	Body		-0.387	-0.016	-0.024	-0.008	-4.124	0.00008920	0.089456718	*	-

control 1: nr. of traumatic events (experienced events)

	logFC	CI.L	CI.R	AveExpr	t	P.Value	adj.P.Val	B
cg00357958	0.007158725	0.004554522	0.009762927	-0.00290854	5.469507411	4.89E-07	0.149643445	4.499067179
cg10712440	-0.009255264	-0.013041009	-0.005469519	0.005602442	-4.864349546	5.56E-06	0.363886083	2.082513924
cg20650753	0.007264314	0.004254563	0.010274065	-0.004147526	4.802322996	7.08E-06	0.363886083	1.869090847
cg13155823	-0.0112443	-0.015947711	-0.006540888	0.007070856	-4.75672735	8.46E-06	0.363886083	1.69272724
cg10201783	-0.006218412	-0.00884122	-0.003595605	0.006213366	-4.717375303	9.84E-06	0.363886083	1.559006992
cg18085862	0.00751557	0.004333453	0.010697687	0.000868409	4.699299545	1.05E-05	0.363886083	1.480108843
cg07383092	0.007783983	0.004472324	0.011095643	-0.007766418	4.676743588	1.15E-05	0.363886083	1.402790959
cg13434525	-0.013545941	-0.019326001	-0.007765881	0.011600396	-4.663143606	1.21E-05	0.363886083	1.362255549
cg19364715	-0.006977976	-0.009961835	-0.003994117	0.003071662	-4.653059172	1.26E-05	0.363886083	1.305775188
cg08764421	-0.010070915	-0.014385141	-0.00575669	0.012864125	-4.644654603	1.30E-05	0.363886083	1.332760127
cg12957132	-0.008358964	-0.011941073	-0.004776855	0.007889205	-4.643024572	1.31E-05	0.363886083	1.26845079
cg01854097	-0.009129266	-0.013070445	-0.005188088	0.002459186	-4.608898063	1.49E-05	0.379924171	1.166647782
cg20190772	-0.012374607	-0.01777423	-0.006974984	0.005086191	-4.560013575	1.80E-05	0.385283905	0.98705249
cg18136026	-0.007854819	-0.011290097	-0.00441954	0.008546578	-4.549478245	1.87E-05	0.385283905	0.946212635
cg15415259	-0.007549224	-0.010853104	-0.004245345	0.006811904	-4.546377284	1.89E-05	0.385283905	0.929626089
cg11884741	-0.007470974	-0.010786192	-0.004155756	0.011060423	-4.483864784	2.39E-05	0.38876501	0.693369284
cg05397490	-0.01214102	-0.017580906	-0.006701134	0.008062197	-4.440844184	2.81E-05	0.38876501	0.537513687
cg11064411	-0.014016715	-0.020307451	-0.00772598	-0.006408055	-4.433500777	2.89E-05	0.38876501	0.495303766
cg18521743	0.007975815	0.00439493	0.011556699	0.002749576	4.431717384	2.91E-05	0.38876501	0.510344989
cg04858318	0.010187135	0.005613178	0.014761091	-0.004816729	4.431468141	2.91E-05	0.38876501	0.486755702
cg06980035	-0.005931871	-0.008595286	-0.003268457	0.003966347	-4.431393113	2.91E-05	0.38876501	0.506486148
cg14396287	-0.017161408	-0.024892024	-0.009430793	0.018572572	-4.417136292	3.08E-05	0.38876501	0.423638499
cg16791476	-0.00765206	-0.011100522	-0.004203598	-0.00120111	-4.415097145	3.09E-05	0.38876501	0.444723347
cg21126183	0.00570049	0.003129144	0.008271836	-0.001256783	4.411020318	3.14E-05	0.38876501	0.432456089
cg20513867	-0.011703157	-0.017007162	-0.006399151	0.012553721	-4.39032631	3.40E-05	0.38876501	0.358600353
cg04197449	-0.004768641	-0.006930876	-0.002606406	0.002474146	-4.388126104	3.42E-05	0.38876501	0.355449892
cg07474266	-0.008761017	-0.012734499	-0.004787534	0.010939974	-4.387029146	3.43E-05	0.38876501	0.365719616
cg07090569	-0.00729761	-0.010639904	-0.003955316	0.003682404	-4.34433517	4.02E-05	0.433621352	0.172431087
cg04186487	0.009798	0.0053013	0.0142947	-0.00339074	4.335416914	4.15E-05	0.433621352	0.153105107
cg09781552	-0.009071964	-0.013248258	-0.00489567	0.006003868	-4.322127484	4.36E-05	0.433621352	0.13029683
cg16162905	-0.007836803	-0.011446603	-0.004227003	0.004639732	-4.319596622	4.40E-05	0.433621352	0.108292506
cg05100867	-0.012285234	-0.01795425	-0.006616219	-0.004090625	-4.311990105	4.54E-05	0.433621352	0.076170827
cg07572966	0.008776817	0.004711388	0.012842246	0.002008925	4.295542603	4.81E-05	0.445767377	0.010947412
cg13932029	0.005300647	0.002839405	0.00776189	-2.37E-05	4.285109284	5.00E-05	0.44952348	-0.016496779
cg01260366	-0.008870711	-0.013006036	-0.004735385	0.003211566	-4.268114716	5.32E-05	0.464697384	-0.071588891
cg00899834	0.008315939	0.004421116	0.012210762	0.006260192	4.248258207	5.72E-05	0.485754091	-0.165672293
cg04095468	-0.006554029	-0.009632024	-0.003476033	-0.001139476	-4.236700303	5.96E-05	0.489921488	-0.205568787
cg02573091	-0.011487575	-0.01688946	-0.006085689	0.002180556	-4.231342583	6.09E-05	0.489921488	-0.156074324
cg22925274	-0.016722389	-0.024628233	-0.008816545	0.006796831	-4.208739342	6.61E-05	0.512429222	-0.288068752
cg18391577	-0.010923566	-0.016093076	-0.005754057	0.004097363	-4.204486234	6.71E-05	0.512429222	-0.325846164
cg26403198	-0.009553983	-0.01408609	-0.005021876	0.005322235	-4.194417298	6.95E-05	0.512429222	-0.330537191
cg23537419	-0.010370516	-0.015297212	-0.005443821	0.01436032	-4.18826917	7.11E-05	0.512429222	-0.335725901
cg07061728	0.01135572	0.005951687	0.016759753	-0.003069018	4.181138903	7.30E-05	0.512429222	-0.364552691
cg09342877	-0.006671105	-0.009853047	-0.003489163	0.001946236	-4.171505966	7.55E-05	0.512429222	-0.430510455
cg16987606	-0.010478369	-0.015483145	-0.005473593	0.006746156	-4.165842078	7.71E-05	0.512429222	-0.446077709
cg25763559	-0.011387208	-0.016828581	-0.005945835	0.006864157	-4.163995139	7.77E-05	0.512429222	-0.456267664
cg14471877	-0.011604485	-0.017154419	-0.006054551	0.006777256	-4.160447381	7.87E-05	0.512429222	-0.46268472
cg14680128	0.009413439	0.004900366	0.013926511	-0.013246849	4.150145536	8.16E-05	0.513798305	-0.489154195
cg15048069	0.020478821	0.010611908	0.030345734	0.000101071	4.129767465	8.79E-05	0.513798305	-0.561791882
cg11220161	0.007806892	0.004036921	0.011576862	0.001130604	4.120287673	9.08E-05	0.513798305	-0.614218998
cg22053920	0.004917575	0.002539371	0.00729578	-0.000791147	4.114236288	9.28E-05	0.513798305	-0.627066784
cg16541026	0.01033749	0.005337737	0.015337244	0.000866679	4.113948866	9.29E-05	0.513798305	-0.609429001
cg03618247	-0.009510685	-0.014129509	-0.00489186	0.0121349	-4.09701638	9.87E-05	0.513798305	-0.666443734
cg09351082	-0.012208041	-0.0181392	-0.006276883	0.008239842	-4.095514218	9.94E-05	0.513798305	-0.666206409
cg06862673	0.00794071	0.004077767	0.011803652	-0.002392154	4.090048306	0.00010118	0.513798305	-0.718861809
cg11258404	0.006971965	0.003569471	0.010374458	-0.007757403	4.077042641	0.000105983	0.513798305	-0.760088781
cg16510350	-0.009994112	-0.014872689	-0.005115535	0.006468811	-4.076071531	0.000106392	0.513798305	-0.744320694
cg05220114	0.008745633	0.004468407	0.01302286	-0.00567816	4.068332022	0.000109323	0.513798305	-0.788183116
cg24018535	-0.004331641	-0.006451372	-0.00221191	0.006124295	-4.065921735	0.000110264	0.513798305	-0.773340941
cg01789980	-0.006609149	-0.009844368	-0.00337393	0.007280752	-4.064706204	0.000110742	0.513798305	-0.801483008
cg26165660	0.008968924	0.004574381	0.013363466	-0.002186404	4.060822453	0.000112282	0.513798305	-0.830667309
cg07370496	-0.012268306	-0.018279417	-0.006257194	0.005945495	-4.060988377	0.000112399	0.513798305	-0.786829499
cg06682073	0.004835647	0.002465549	0.007205745	-0.005034922	4.059528413	0.0001128	0.513798305	-0.802038477
cg17989833	-0.006914203	-0.010309934	-0.003518472	0.00325205	-4.051316499	0.000116139	0.513798305	-0.827705222
cg12609893	-0.0170269	-0.025398842	-0.008654958	0.011141404	-4.046796163	0.000118208	0.513798305	-0.865975657
cg01329836	-0.00533219	-0.007958894	-0.002705485	-0.00045673	-4.039073624	0.000121294	0.513798305	-0.902138494
cg21877774	-0.006213373	-0.009276554	-0.003150193	0.000365481	-4.035916709	0.000122659	0.513798305	-0.896096993
cg15612342	-0.007366583	-0.011004806	-0.003728359	-0.001347951	-4.028691762	0.000125837	0.513798305	-0.913238863
cg07850534	-0.006030403	-0.00900883	-0.003051977	0.001439274	-4.028532021	0.000125908	0.513798305	-0.899373737
cg14803663	-0.005159032	-0.007711414	-0.002606649	0.003776868	-4.021701516	0.000128987	0.513798305	-0.950912471
cg06000353	0.02052502	0.010361617	0.030688423	-0.034600514	4.018337506	0.000130737	0.513798305	-0.955540677
cg06664423	-0.008120713	-0.012142813	-0.004098613	-0.003095421	-4.017247901	0.000131034	0.513798305	-0.957261698
cg22335692	0.004994943	0.002518678	0.007471207	0.000312276	4.013477275	0.000132791	0.513798305	-0.95653668
cg16988709	-0.007068854	-0.010575276	-0.003562432	0.000717536	-4.01117983	0.000133873	0.513798305	-0.968390292
cg05560494	-0.008869411	-0.013273248	-0.004465574	0.003669357	-4.007291455	0.000135722	0.513798305	-0.976515195

cg14789818	-0.012555547	-0.018791557	-0.006319537	-0.00220728	-4.006183067	0.000136467	0.513798305	-0.991167549
cg01548463	-0.007608144	-0.011388578	-0.00382771	0.013461711	-4.004279969	0.000137172	0.513798305	-0.980666714
cg17502495	0.009878576	0.004969286	0.014787866	-0.000711319	4.003754227	0.000137491	0.513798305	-0.996514818
cg11941886	-0.007110067	-0.010643672	-0.003576463	0.005476034	-4.003530925	0.000137535	0.513798305	-1.008329139
cg15728672	0.00945102	0.004751663	0.014150378	-0.010328669	4.001560526	0.000138521	0.513798305	-1.015455555
cg23650331	-0.008555753	-0.012824988	-0.004286518	0.008541647	-3.987452096	0.000145549	0.513798305	-1.042579301
cg15971010	-0.003933183	-0.005896545	-0.001969821	-0.004194189	-3.985943294	0.000146324	0.513798305	-1.051051887
cg08426200	0.007479543	0.003745638	0.011213448	0.004429347	3.985649914	0.000146475	0.513798305	-1.03486054
cg07194984	-0.007979001	-0.011962781	-0.003995222	-0.004954412	-3.985112634	0.000146752	0.513798305	-1.06989028
cg19243842	-0.007931483	-0.011894266	-0.003968699	0.002658727	-3.982367823	0.000148175	0.513798305	-1.054878775
cg04418630	-0.009663782	-0.014495824	-0.004831739	0.00694508	-3.979287255	0.000149813	0.513798305	-1.059402408
cg00635356	-0.006486936	-0.009731991	-0.00324188	0.003863844	-3.977450477	0.000150758	0.513798305	-1.088081699
cg09724304	-0.008998606	-0.013504292	-0.00449292	0.001816477	-3.973760056	0.000152725	0.513798305	-1.096621302
cg26644442	-0.005045559	-0.007573188	-0.00251793	0.001042064	-3.971762458	0.0001538	0.513798305	-1.097491885
cg01788025	0.007414216	0.00369972	0.011128711	-0.001974724	3.971483361	0.000153951	0.513798305	-1.067797418
cg09416149	0.009738898	0.004853306	0.014624491	0.014513768	3.96628079	0.000156844	0.513798305	-1.1171936
cg10311700	0.012426364	0.00618194	0.018670787	-0.021583866	3.959621629	0.000160731	0.513798305	-1.130282638
cg02568761	-0.006182698	-0.009291447	-0.003073948	-0.002227876	-3.957123904	0.000161901	0.513798305	-1.145393754
cg02275985	-0.010210623	-0.015346672	-0.005074574	0.017219518	-3.955630154	0.000162838	0.513798305	-1.124141657
cg11687809	-0.005345509	-0.008034588	-0.002656431	0.007582897	-3.95524088	0.000162972	0.513798305	-1.176952385
cg22539450	0.006034337	0.002998227	0.009070448	-0.009122293	3.954571246	0.000163355	0.513798305	-1.166275106
cg00502901	-0.01428423	-0.021475927	-0.007092534	0.015761966	-3.952095742	0.000165023	0.513798305	-1.176455568
cg09219776	0.007774469	0.003856071	0.011692867	-0.006819319	3.947749335	0.000167302	0.513798305	-1.165928941
cg09435541	-0.008416133	-0.012658616	-0.00417365	0.015603476	-3.947115098	0.000167673	0.513798305	-1.183247266
cg05208416	-0.004671255	-0.007029114	-0.002313397	0.0031002	-3.941878957	0.000170771	0.513798305	-1.223780367
cg03333253	-0.007104732	-0.010692324	-0.00351714	0.00130461	-3.940324157	0.000171702	0.513798305	-1.218023502
cg11991735	0.01433403	0.007094864	0.021573197	-0.004239378	3.939868268	0.00017223	0.513798305	-1.221824379
cg18617760	-0.005717765	-0.008606602	-0.002828928	-0.000368884	-3.938135416	0.00017302	0.513798305	-1.212246636
cg10836779	-0.01015276	-0.015290099	-0.00501542	0.018803695	-3.932228427	0.000176724	0.517293068	-1.206030749
cg05125809	0.012515745	0.006180783	0.018850706	-0.005993611	3.93110543	0.000177579	0.517293068	-1.248029733
cg24395057	-0.006880159	-0.010368501	-0.003391817	0.004964197	-3.924341395	0.000181552	0.519516735	-1.260523907
cg24137216	-0.008173897	-0.012318496	-0.004029298	0.014176916	-3.924045181	0.000181739	0.519516735	-1.245991072
cg02176069	-0.007878384	-0.01187911	-0.003877657	0.003298257	-3.91819131	0.000185484	0.523441099	-1.302377243
cg26826434	-0.010098473	-0.015228475	-0.004968471	0.001760908	-3.916829265	0.000186535	0.523441099	-1.297459808
cg22295925	0.013881595	0.006823183	0.020940007	0.010645434	3.913220624	0.000188995	0.525523153	-1.297278763
cg11193462	-0.009723284	-0.014679867	-0.004766702	0.00732536	-3.903239179	0.000195526	0.538502927	-1.355092832
cg25403475	0.007538476	0.0036931	0.011383851	0.003109653	3.90060603	0.000197184	0.538502927	-1.314565053
cg10620475	-0.006337771	-0.009574397	-0.003101145	0.003729065	-3.896111668	0.000200286	0.541414604	-1.372632465
cg07161062	0.004259665	0.00208211	0.006437221	0.000524095	3.892186059	0.000203033	0.541414604	-1.344680711
cg05592363	-0.005908721	-0.008932219	-0.002885222	0.000577244	-3.88840096	0.000205716	0.541414604	-1.366300156
cg17687564	0.006244391	0.00304705	0.009441732	-0.004357336	3.885871606	0.000207528	0.541414604	-1.391516123
cg24604274	-0.012326681	-0.01863834	-0.006015023	0.011267281	-3.886016298	0.000207717	0.541414604	-1.3795534
cg22706007	-0.01267212	-0.019166022	-0.006178217	0.008902397	-3.882804104	0.000210041	0.541414604	-1.402548943
cg01750221	-0.020042977	-0.030316298	-0.009769655	0.011602525	-3.881980084	0.000210641	0.541414604	-1.432964591
cg20373822	-0.00469111	-0.007097453	-0.002284766	0.006527208	-3.878871369	0.000212622	0.54195209	-1.411627082
cg25213350	0.007512284	0.003652065	0.011372503	0.000913	3.872107351	0.000217658	0.545848468	-1.456061374
cg03607379	-0.019542717	-0.029591249	-0.009494186	-0.002355202	-3.869762598	0.000219735	0.545848468	-1.469568545
cg13864543	-0.011148435	-0.016886204	-0.005410665	0.016734719	-3.866096977	0.000222536	0.545848468	-1.46165256
cg09536738	-0.005896762	-0.008934359	-0.002859165	0.001113924	-3.862520282	0.000224991	0.545848468	-1.486913676
cg15521382	-0.010192858	-0.015443301	-0.004942415	0.012697761	-3.86276406	0.000225031	0.545848468	-1.474416105
cg18844905	-0.015810918	-0.023957653	-0.007664183	0.014003903	-3.861672522	0.000225962	0.545848468	-1.461950705
cg16908949	-0.007624458	-0.011554723	-0.003694193	-0.007976412	-3.859885919	0.000227047	0.545848468	-1.487199731
cg06702849	-0.006512106	-0.009870502	-0.003153709	0.00223435	-3.858130042	0.000228427	0.545848468	-1.494213183
cg09473745	0.005797533	0.002802422	0.008792645	-0.010358417	3.851391539	0.0002338	0.547083373	-1.479479259
cg08799570	-0.009440322	-0.014322665	-0.004557979	0.005592021	-3.847251566	0.000237265	0.547083373	-1.527618014
cg08837558	-0.008863527	-0.013449216	-0.004277838	0.003256762	-3.845833989	0.000238347	0.547083373	-1.548001558
cg24719399	0.014919289	0.007197359	0.022641219	-0.023863824	3.844361481	0.000239859	0.547083373	-1.530425229
cg24545961	0.008280445	0.003992811	0.012568079	-0.008406144	3.842583534	0.000241004	0.547083373	-1.54338534
cg23778149	-0.008904911	-0.013518254	-0.004291569	-0.001785732	-3.840619125	0.00024264	0.547083373	-1.502369555
cg09445132	-0.007968926	-0.012098421	-0.003839432	0.005299345	-3.839637952	0.00024346	0.547083373	-1.566735772
cg07260207	0.008797171	0.004238402	0.01335594	-0.00188636	3.839571645	0.000243516	0.547083373	-1.543003601
cg22863492	-0.007172946	-0.010894745	-0.003451148	-0.00477919	-3.83470583	0.000247627	0.547083373	-1.555741861
cg18707858	0.005851279	0.00281447	0.008888087	0.001187982	3.833722812	0.000248465	0.547083373	-1.578993632
cg03762549	-0.006522998	-0.009908587	-0.003137409	0.005891389	-3.83354311	0.000248619	0.547083373	-1.584793596
cg11343894	-0.010539816	-0.016017803	-0.005061829	0.003727641	-3.828363881	0.000253406	0.550239632	-1.580144266
cg00163541	-0.005941739	-0.00903059	-0.002852888	0.0065835	-3.827401192	0.000253923	0.550239632	-1.586287578
cg27528369	-0.008148504	-0.012387349	-0.003909658	0.010436268	-3.824878522	0.000256133	0.550239632	-1.596166721
cg10706072	0.013389771	0.00642259	0.020356953	-0.007031443	3.824001072	0.000257249	0.550239632	-1.596052505
cg03862987	-0.005792398	-0.00881004	-0.002774756	0.001726713	-3.819249361	0.00026113	0.552260079	-1.620991394
cg14435474	0.006766353	0.003240619	0.010292087	0.000870134	3.818496836	0.000261805	0.552260079	-1.630838835
cg19371706	-0.005483449	-0.008343034	-0.002623864	-0.001449414	-3.815382846	0.000264616	0.55436675	-1.635767857
cg05863683	-0.004865851	-0.007408085	-0.002323617	0.000317864	-3.808295665	0.000271122	0.564132215	-1.636309514
cg21949305	-0.006038031	-0.009198446	-0.002877615	0.009444824	-3.801355598	0.00027764	0.567617012	-1.666323666
cg02192974	-0.008706928	-0.013271958	-0.004141898	0.005449882	-3.794978542	0.000283778	0.567617012	-1.710223841
cg22998101	-0.010656698	-0.016244815	-0.005068582	0.010838634	-3.794540349	0.000284557	0.567617012	-1.699968767
cg22052586	0.00799294	0.003798279	0.012187601	-0.005911307	3.791377539	0.000287275	0.567617012	-1.719551594
cg13508369	-0.008532298	-0.013010127	-0.004054469	0.010223783	-3.791279851	0.000287371	0.567617012	-1.705850044

cg24441440	0.007910394	0.003757042	0.012063747	-0.012668504	3.789541694	0.000289082	0.567617012	-1.657084847
cg08331138	-0.006385217	-0.009739246	-0.003031188	0.00417973	-3.787880005	0.000290727	0.567617012	-1.698975505
cg07801814	0.020231754	0.00960503	0.030858479	-0.031408776	3.788227657	0.000290758	0.567617012	-1.700777596
cg06064115	-0.007866987	-0.012001795	-0.00373218	0.004747632	-3.785650164	0.000292948	0.567617012	-1.71671366
cg18348731	-0.011785329	-0.017979762	-0.005590896	0.007056765	-3.785664387	0.000293313	0.567617012	-1.73634528
cg19254533	0.007454708	0.003533401	0.011376016	-0.004942998	3.782570883	0.000296042	0.567617012	-1.697731989
cg10007041	-0.010003638	-0.015268343	-0.004738933	0.009826578	-3.780783966	0.00029813	0.567617012	-1.738579216
cg03704112	0.012271094	0.005812426	0.018729761	0.010983323	3.780439676	0.000298586	0.567617012	-1.730636787
cg16331492	-0.013305481	-0.020312031	-0.00629893	0.007563403	-3.778576866	0.000300488	0.567617012	-1.759958385
cg00510236	-0.005962742	-0.009102995	-0.002822489	0.002546136	-3.778058841	0.000300633	0.567617012	-1.707477814
cg16728898	0.011373098	0.005377372	0.017368825	-0.012001502	3.774322228	0.000304875	0.568691972	-1.71801337
cg24652615	0.00761979	0.00360244	0.011637139	0.007792706	3.773903147	0.000304921	0.568691972	-1.747973648
cg12194653	0.008797727	0.004155492	0.013439962	0.001382796	3.770775536	0.000308186	0.571298759	-1.740750359
cg00183936	-0.005936134	-0.009076282	-0.002795986	0.007519913	-3.761325501	0.000318257	0.583633005	-1.777758163
cg06496454	-0.008912079	-0.013626923	-0.004197235	-0.005255994	-3.760966544	0.000318656	0.583633005	-1.784647634
cg15090391	-0.007880542	-0.012054025	-0.003707059	0.0112438	-3.757031293	0.000322936	0.584010664	-1.819207011
cg01738984	-0.007568228	-0.011577577	-0.003558878	-0.003164278	-3.755844833	0.00032424	0.584010664	-1.813174803
cg17619116	-0.009634367	-0.014738596	-0.004530139	0.007158625	-3.755652009	0.00032459	0.584010664	-1.78459093
cg06655097	-0.011504487	-0.017614026	-0.005394948	0.000282747	-3.746802395	0.000334765	0.592066341	-1.857178574
cg11912272	-0.008921979	-0.01366555	-0.004178409	-0.007179655	-3.742355492	0.00033948	0.592066341	-1.862735753
cg18586851	-0.005132055	-0.007860835	-0.002403275	0.005715153	-3.74205472	0.000339773	0.592066341	-1.845892705
cg18156543	-0.007806155	-0.011963653	-0.003648658	-0.007066804	-3.735876492	0.00034696	0.592066341	-1.896825919
cg05651489	-0.005361809	-0.008218182	-0.002505437	0.002834239	-3.734941849	0.00034806	0.592066341	-1.849339472
cg08599521	-0.007266296	-0.011137256	-0.003395336	0.000219164	-3.734924012	0.000348081	0.592066341	-1.872893706
cg17158584	-0.010662602	-0.016343286	-0.004981918	0.017983465	-3.734775908	0.000348686	0.592066341	-1.878949599
cg04945518	-0.017049492	-0.026134598	-0.007964387	0.018657388	-3.734078329	0.00034951	0.592066341	-1.89743888
cg11582617	-0.005821589	-0.008924141	-0.002719038	-0.001547576	-3.733446267	0.000349827	0.592066341	-1.900797492
cg08404225	-0.007075513	-0.010847579	-0.003303446	0.00735689	-3.732208633	0.000351295	0.592066341	-1.90244066
cg11678205	-0.004723737	-0.007242639	-0.002204835	-0.002401924	-3.731313647	0.000352361	0.592066341	-1.896068504
cg27321658	0.012378365	0.005777258	0.018979472	-0.00645601	3.731199476	0.000352931	0.592066341	-1.908555555
cg19547189	-0.003990845	-0.00612008	-0.001861611	0.001547402	-3.729312247	0.000354755	0.592066341	-1.879419771
cg15184745	0.00749594	0.003494103	0.011497778	-0.001165468	3.726954038	0.000357595	0.592066341	-1.873947084
cg24682449	-0.006049309	-0.009279624	-0.002818995	0.003943443	-3.72604734	0.000358693	0.592066341	-1.915631333
cg00440448	0.006958021	0.00323924	0.010676803	-0.002909961	3.72282365	0.000362623	0.592066341	-1.914498518
cg00176801	-0.007381325	-0.01133184	-0.003430811	0.005108204	-3.717645813	0.000369021	0.592066341	-1.930004049
cg24736274	0.011458824	0.005325687	0.01759196	-0.000483487	3.717571542	0.000369562	0.592066341	-1.939838893
cg18680626	-0.009900621	-0.015201472	-0.004599769	0.016484914	-3.716336716	0.000370993	0.592066341	-1.943586177
cg17634995	-0.007427636	-0.011404817	-0.003450454	0.015003038	-3.715887433	0.000371218	0.592066341	-1.931054141
cg20646556	-0.011296369	-0.017346896	-0.005245842	0.006171353	-3.714904278	0.000372903	0.592066341	-1.975213715
cg00895186	0.00563178	0.002614606	0.008648954	-0.00158317	3.713921138	0.000373689	0.592066341	-1.976816113
cg23714454	-0.009728434	-0.014945384	-0.004511484	0.000945766	-3.71042742	0.000378442	0.592066341	-1.971090818
cg20747455	0.007286493	0.003377153	0.011195833	0.005526786	3.708535379	0.000380539	0.592066341	-1.982884596
cg00834712	-0.005099095	-0.007835974	-0.002362217	0.005882179	-3.707020814	0.000382487	0.592066341	-1.947189457
cg24512517	-0.008780244	-0.013492942	-0.004067546	-0.003491708	-3.707016959	0.000382501	0.592066341	-1.957490771
cg11043485	-0.009459153	-0.014542368	-0.004375939	0.008631279	-3.7025958	0.000388396	0.592066341	-1.962624924
cg24354272	0.005087206	0.002353352	0.00782106	0.003139419	3.702468828	0.000388398	0.592066341	-2.015023691
cg21750428	-0.006603651	-0.010152803	-0.003054499	0.001380759	-3.702089154	0.000388895	0.592066341	-1.983984382
cg14784877	-0.008416233	-0.012941764	-0.003890702	0.006169386	-3.700287934	0.000391261	0.592066341	-2.01488076
cg00235562	-0.006908208	-0.010625966	-0.003190449	0.007718807	-3.697188245	0.000395365	0.592066341	-2.005153695
cg16570507	-0.008663017	-0.013326979	-0.003999055	-0.001740512	-3.69574087	0.000397295	0.592066341	-1.990890509
cg22705959	0.006920853	0.003194601	0.010647105	-0.001146798	3.695513004	0.0003976	0.592066341	-2.015748011
cg26995819	-0.010000489	-0.015386047	-0.004614932	-0.011630613	-3.694765401	0.000398892	0.592066341	-1.97126107
cg24889693	-0.007270843	-0.011187759	-0.003353928	0.00033583	-3.693413524	0.000400418	0.592066341	-2.030413388
cg05671636	-0.005644205	-0.008686293	-0.002602118	0.003720253	-3.691632104	0.000402823	0.592066341	-2.005109011
cg03056854	-0.008391213	-0.012914813	-0.003867612	-0.005644228	-3.690862397	0.000403867	0.592066341	-2.039739331
cg15303621	-0.006048869	-0.00931231	-0.002785427	0.005329157	-3.687955649	0.000407832	0.592066341	-2.055396254
cg17320698	-0.012733037	-0.019604484	-0.00586159	0.012370208	-3.687107234	0.00040948	0.592066341	-2.065849628
cg09814448	-0.00992238	-0.015277318	-0.004567441	0.011485078	-3.686906926	0.000409717	0.592066341	-2.056385007
cg08573879	-0.009511588	-0.014647454	-0.004375722	0.013250214	-3.684970471	0.000412185	0.592066341	-2.032036193
cg13524311	-0.009592816	-0.014774136	-0.004411495	0.007537003	-3.683857746	0.000413813	0.592066341	-2.055887762
cg01316390	-0.008759202	-0.013491148	-0.004027256	-0.002794458	-3.683107057	0.000414602	0.592066341	-2.059830883
cg12420900	0.00870069	0.003999356	0.013402023	-0.012264189	3.682306862	0.000415643	0.592066341	-2.033345203
cg06133671	-0.006229618	-0.00959608	-0.002863157	0.009003567	-3.681926481	0.000416174	0.592066341	-2.04818297
cg12572807	-0.00978152	-0.015081322	-0.004481719	0.005646089	-3.672369842	0.000430153	0.60409401	-2.099516054
cg16834356	0.005662546	0.002594119	0.008730972	-0.001477729	3.671836	0.0004305	0.60409401	-2.07447528
cg10030658	0.006358686	0.002909892	0.009807481	-0.001261396	3.668490776	0.000435351	0.60409401	-2.120108811
cg18967788	0.005177634	0.002368512	0.007986757	0.002277787	3.667313128	0.000437072	0.60409401	-2.088133919
cg06229084	-0.006116437	-0.009435028	-0.002797846	0.00305516	-3.66717939	0.000437268	0.60409401	-2.120542845
cg10825530	-0.012348471	-0.019049104	-0.005647838	0.010092664	-3.666901922	0.000438182	0.60409401	-2.117908448
cg17094356	0.007574178	0.003461412	0.011686944	-0.005085966	3.664281442	0.000441531	0.60409401	-2.112888402
cg07091346	-0.004942572	-0.007627918	-0.002257225	0.002968212	-3.662182145	0.000444643	0.60409401	-2.087979501
cg02070677	0.00684274	0.00312482	0.01056066	-0.006934523	3.661991488	0.000444927	0.60409401	-2.12648824
cg07516742	-0.006125752	-0.009456905	-0.002794599	-0.001782738	-3.658913709	0.000449531	0.60409401	-2.144050365
cg12623101	-0.005692386	-0.008787948	-0.002596823	0.002297021	-3.658829191	0.000449658	0.60409401	-2.134243471
cg00919534	0.005561601	0.002537091	0.008586111	-0.001405607	3.658745488	0.000449784	0.60409401	-2.109238002
cg12483466	0.006959537	0.003172197	0.010746876	0.002514525	3.656229618	0.000453583	0.60409401	-2.13727253
cg03573137	0.007903884	0.003602111	0.012205657	0.001211743	3.655782835	0.000454261	0.60409401	-2.153002645

cg18466176	-0.006701824	-0.010350069	-0.003053578	-0.003857377	-3.655074909	0.000455337	0.60409401	-2.135282348
cg13389684	0.007496926	0.0034152	0.011578652	0.002768197	3.654489626	0.000456229	0.60409401	-2.16069263
cg03347216	-0.004811585	-0.007433517	-0.002189654	0.004135984	-3.651355805	0.00046103	0.607820371	-2.140061619
cg13807254	-0.008336581	-0.01288373	-0.003789432	0.01230546	-3.647843153	0.000466469	0.608426735	-2.170193512
cg08938597	-0.011182322	-0.017288431	-0.005076213	0.008963054	-3.643924527	0.000473146	0.608426735	-2.155987327
cg03028257	-0.00542865	-0.008393638	-0.002463663	-0.000318963	-3.642976625	0.000474105	0.608426735	-2.186815144
cg00288957	-0.013336761	-0.020621875	-0.006051647	0.004694657	-3.642637584	0.00047518	0.608426735	-2.201082475
cg26723048	-0.007088964	-0.010967494	-0.003210435	-0.001813384	-3.636662654	0.000484189	0.608426735	-2.202609645
cg14725590	-0.009645684	-0.014924917	-0.004366451	0.020886337	-3.635470468	0.000486532	0.608426735	-2.20646418
cg14761693	-0.006081538	-0.009410815	-0.00275226	0.010760698	-3.634550648	0.000487608	0.608426735	-2.191205359
cg14069111	-0.00677991	-0.010491945	-0.003067875	0.0034368	-3.634119206	0.000488309	0.608426735	-2.214303293
cg07243104	-0.004771026	-0.007383447	-0.002158605	-0.004124202	-3.633758317	0.000488896	0.608426735	-2.163076194
cg02958262	-0.009969342	-0.015428075	-0.004510609	0.009336796	-3.633918456	0.000489133	0.608426735	-2.201470453
cg11203714	0.005493285	0.00248506	0.00850151	-0.001423421	3.633366477	0.000489535	0.608426735	-2.220272513
cg23042590	0.009532888	0.004312625	0.014753152	-0.010934762	3.633544041	0.000489659	0.608426735	-2.222807578
cg16176440	-0.006336708	-0.0098083	-0.002865116	0.016202029	-3.631803788	0.000492088	0.608426735	-2.168654221
cg01021483	-0.005216075	-0.008073807	-0.002358342	0.000873949	-3.631696271	0.000492264	0.608426735	-2.212684516
cg05670345	-0.008432905	-0.013053478	-0.003812333	0.011138409	-3.631358099	0.00049283	0.608426735	-2.218072207
cg06053702	-0.005653816	-0.008752613	-0.00255502	0.005016948	-3.630245139	0.000494648	0.608426735	-2.225262513
cg03413202	-0.007749278	-0.011998347	-0.003500209	0.005641886	-3.628731127	0.000497146	0.608426735	-2.209107267
cg13391663	-0.006287267	-0.009736022	-0.002838512	0.000830165	-3.627328845	0.000499471	0.608426735	-2.207886892
cg08650923	-0.006834376	-0.010584184	-0.003084567	0.003379784	-3.626411225	0.000500998	0.608426735	-2.189607877
cg03156273	-0.005245907	-0.008124511	-0.002367303	0.003594942	-3.625985628	0.000501707	0.608426735	-2.245222282
cg12632785	0.011788993	0.005316252	0.018261734	-0.017811553	3.624018663	0.000505563	0.608426735	-2.242109392
cg06068118	-0.004892776	-0.007580358	-0.002205194	0.000592359	-3.622271549	0.000507941	0.608426735	-2.257285728
cg02290969	-0.004378415	-0.006784153	-0.001972676	0.002648797	-3.621226788	0.000509708	0.608426735	-2.257148607
cg18696822	-0.0111975	-0.017350118	-0.005044883	0.021473116	-3.621288499	0.000510171	0.608426735	-2.222411918
cg17823807	-0.003646205	-0.005650415	-0.001641994	-0.002100959	-3.619804552	0.000512122	0.608426735	-2.230491442
cg16422714	0.006419175	0.002889488	0.009948862	-0.001954504	3.618514774	0.000514321	0.608426735	-2.254810707
cg06624212	-0.00685594	-0.010627249	-0.003084632	0.002422768	-3.617114799	0.000516718	0.608426735	-2.275849541
cg13720362	0.00576732	0.002594465	0.008940175	0.002697741	3.616689002	0.000517449	0.608426735	-2.266259339
cg10167834	-0.006537957	-0.010135772	-0.002940143	-0.00258867	-3.615685403	0.000519176	0.608426735	-2.227405453
cg09750940	0.012746759	0.005726484	0.019767034	-0.013739753	3.612830839	0.000524698	0.612550457	-2.266370918
cg18095002	-0.008322624	-0.012908128	-0.003737121	-0.006141422	-3.61127565	0.00052683	0.612701434	-2.271837743
cg01132769	0.00799319	0.003584254	0.012402126	0.005194133	3.607228933	0.000533948	0.613068768	-2.238303449
cg01982748	-0.006786125	-0.010529557	-0.003042694	0.008259241	-3.606943852	0.000534453	0.613068768	-2.301504397
cg02525376	-0.014852138	-0.023044675	-0.006659602	0.012775964	-3.607219243	0.000534552	0.613068768	-2.305388542
cg11020665	-0.008218985	-0.012753329	-0.003684642	0.008725603	-3.606543122	0.000535163	0.613068768	-2.285060626
cg10296382	-0.006072268	-0.009426058	-0.002718477	0.005151431	-3.602486535	0.000542406	0.616831437	-2.327029464
cg17809365	-0.005943213	-0.009227708	-0.002658717	0.006077948	-3.600310319	0.000546329	0.616831437	-2.323674892
cg07151380	-0.009502009	-0.014755786	-0.004248232	0.01198663	-3.598665093	0.000549734	0.616831437	-2.317660764
cg05736785	-0.006637051	-0.010310391	-0.002963711	0.007792876	-3.595020452	0.000555979	0.616831437	-2.30767585
cg03858365	0.010424579	0.004654658	0.0161945	0.002051794	3.594931429	0.000556747	0.616831437	-2.342811899
cg21396456	-0.004476459	-0.006954751	-0.001998166	0.000723444	-3.593928016	0.000557992	0.616831437	-2.303675034
cg07630162	-0.006519256	-0.010128797	-0.002909714	0.006798016	-3.593629883	0.000558543	0.616831437	-2.334107057
cg04966825	-0.008371891	-0.013007663	-0.00373612	0.010794288	-3.593277501	0.00055927	0.616831437	-2.359790042
cg15433297	-0.004691258	-0.007289156	-0.002093361	0.005967311	-3.59297903	0.000559746	0.616831437	-2.303648707
cg18938560	-0.012611065	-0.019597184	-0.005624945	0.01646207	-3.591845779	0.000562457	0.616831437	-2.357404202
cg11590578	-0.00817754	-0.012708066	-0.003647015	0.013130535	-3.591380724	0.000562713	0.616831437	-2.337049032
cg00866179	0.006827918	0.003044938	0.010610898	0.003147609	3.591216543	0.000563018	0.616831437	-2.337191856
cg03159960	-0.007743757	-0.01203521	-0.003452305	0.011784824	-3.59033331	0.000564665	0.616831437	-2.332936261
cg17221584	0.011903837	0.005302373	0.018505302	-0.00733943	3.587968794	0.000569709	0.617881535	-2.340900867
cg22113651	-0.01094304	-0.017012202	-0.004873879	0.005467441	-3.587659641	0.000570291	0.617881535	-2.360966877
cg25866895	-0.006679421	-0.010384897	-0.002973945	0.003623868	-3.58659372	0.000571686	0.617881535	-2.355925461
cg22971145	-0.015976171	-0.02484569	-0.007106652	0.010038361	-3.584054455	0.000577121	0.61997833	-2.374070324
cg03717494	-0.00869359	-0.013520679	-0.0038665	0.014889433	-3.58345528	0.00057768	0.61997833	-2.335878531
cg00015193	0.005884363	0.002615795	0.009152931	-0.002078577	3.582030577	0.000580366	0.620683331	-2.329677219
cg23403388	-0.00912647	-0.014199618	-0.004053323	0.013428365	-3.579435502	0.000585437	0.622012055	-2.313962281
cg17452800	-0.006434531	-0.010013536	-0.002855525	0.00567558	-3.577189103	0.000589712	0.622012055	-2.376918069
cg17514816	-0.003591774	-0.005590016	-0.001593531	0.000116131	-3.576417169	0.000591216	0.622012055	-2.396346867
cg12626242	-0.010081333	-0.015692674	-0.004469991	0.010297338	-3.574812031	0.000594987	0.622012055	-2.413130835
cg18120193	-0.00773389	-0.012041179	-0.003426601	0.00398036	-3.572574912	0.000598752	0.622012055	-2.423891954
cg09353184	-0.007889936	-0.012284149	-0.003495723	-0.000335807	-3.572561778	0.000598778	0.622012055	-2.356271593
cg15095727	-0.008115833	-0.012638064	-0.003593602	0.013380006	-3.570818099	0.000602229	0.622012055	-2.418955217
cg03593896	-0.005124168	-0.007979557	-0.002268779	0.001812386	-3.57063486	0.000602592	0.622012055	-2.417851736
cg23772011	-0.009715304	-0.015128921	-0.004301688	0.001753047	-3.570828166	0.000602763	0.622012055	-2.403517537
cg04077175	-0.012130925	-0.018891131	-0.005370719	0.006186591	-3.570556574	0.000603388	0.622012055	-2.396618289
cg06179485	-0.006675929	-0.010396747	-0.002955111	0.000550325	-3.569937555	0.000603978	0.622012055	-2.410960949
cg10564013	-0.00561173	-0.008744584	-0.002478875	0.005137698	-3.564050879	0.000615799	0.626742421	-2.39947173
cg09167664	0.007090768	0.00313107	0.011050466	-0.006177264	3.563022324	0.000617887	0.626742421	-2.426943889
cg05696848	-0.007737035	-0.012058439	-0.003415632	0.005582887	-3.562354294	0.000619246	0.626742421	-2.431343839
cg08667706	-0.008867532	-0.013820851	-0.003914213	0.003785751	-3.562043586	0.00062012	0.626742421	-2.435674868
cg02931191	-0.0074137	-0.011556907	-0.003270493	0.002731348	-3.560292676	0.00062346	0.626742421	-2.449480723
cg12544923	-0.009112358	-0.014207439	-0.004017276	0.003008207	-3.558551554	0.000627322	0.626742421	-2.429055639
cg05105069	-0.012530296	-0.019536877	-0.005523716	-0.001203575	-3.558419782	0.00062797	0.626742421	-2.46094148
cg19341849	-0.006478367	-0.010101591	-0.002855143	-0.002809917	-3.557604495	0.000628995	0.626742421	-2.465494306

control 2: nr. of traumatic events (perpetrated events)

	logFC	CI.L	CI.R	AveExpr	t	P.Value	adj.P.Val	B
cg07000611	-0.003575793	-0.004710493	-0.002441093	-0.000684099	-6.270162415	1.66E-08	0.005079379	6.888472386
cg10361765	0.004171433	0.002753449	0.005589416	0.004739624	5.853308082	9.86E-08	0.015086759	5.169577107
cg02257517	-0.004202052	-0.005708083	-0.00269602	-0.005507195	-5.551554953	3.49E-07	0.035546579	3.857480998
cg07370496	0.006382239	0.003973661	0.008790817	0.005945495	5.272472533	1.10E-06	0.073213305	2.798729681
cg04094062	-0.003472987	-0.004798783	-0.002147192	-0.003494712	-5.212115116	1.40E-06	0.073213305	2.503134942
cg19320967	0.007805118	0.004822054	0.010788182	-0.008026884	5.206176177	1.44E-06	0.073213305	2.506295769
cg10180440	-0.008558728	-0.01193824	-0.005179216	-0.014678937	-5.039148839	2.80E-06	0.099004067	1.833643282
cg12761144	0.004143392	0.002502446	0.005784339	0.003638398	5.023992296	2.97E-06	0.099004067	1.755243006
cg07599737	0.003477019	0.002096702	0.004857336	0.001589135	5.012052955	3.11E-06	0.099004067	1.716091055
cg25500028	0.006323233	0.003808276	0.008838189	0.003235463	5.002770272	3.24E-06	0.099004067	1.663002043
cg08481406	0.002871119	0.001711696	0.004030543	0.009129018	4.92715884	4.35E-06	0.120910534	1.404055438
cg15821095	0.003232449	0.001917503	0.004547395	0.004802879	4.891152162	5.01E-06	0.127664614	1.28140138
cg17894088	0.006581749	0.003862282	0.009301216	-0.002747643	4.815700923	6.74E-06	0.158673947	0.966702138
cg25659578	-0.004039585	-0.005729353	-0.002349817	-0.001580996	-4.756604204	8.46E-06	0.184766107	0.739157291
cg27348370	0.004639736	0.002677976	0.006601496	0.015339794	4.705912955	1.03E-05	0.193246786	0.541683468
cg05746708	0.003789388	0.00218292	0.005395856	0.011736978	4.693365525	1.08E-05	0.193246786	0.508226714
cg10563898	0.00400253	0.002289605	0.005715454	0.014369795	4.649258791	1.28E-05	0.193246786	0.342077837
cg22053920	-0.002131906	-0.003046583	-0.001217228	-0.000791147	-4.637536839	1.34E-05	0.193246786	0.2948835
cg10836779	0.004535356	0.002589446	0.006481266	0.018803695	4.637474054	1.34E-05	0.193246786	0.31773061
cg23913541	0.002237666	0.001276676	0.003198656	0.002226386	4.633015063	1.36E-05	0.193246786	0.280934915
cg13495336	0.005411536	0.003086776	0.007736295	0.008661639	4.631743331	1.37E-05	0.193246786	0.257320263
cg21852792	-0.004517544	-0.006460035	-0.002575052	-0.014656828	-4.62741999	1.39E-05	0.193246786	0.250391261
cg19907782	-0.004375133	-0.006262098	-0.002488169	-0.008871598	-4.613404335	1.47E-05	0.193246786	0.185562121
cg25504717	-0.003913616	-0.005604809	-0.002222423	-0.004131605	-4.604393054	1.52E-05	0.193246786	0.173140996
cg08439109	-0.003982944	-0.005714311	-0.002251577	-0.012011751	-4.577226443	1.68E-05	0.201080158	0.067736838
cg16771366	-0.002755166	-0.003953985	-0.001556348	-0.004914197	-4.57279419	1.71E-05	0.201080158	0.054050138
cg11809139	0.005360481	0.003023232	0.00769773	0.013866623	4.563528837	1.78E-05	0.201080158	0.017999321
cg04241623	-0.004181122	-0.006012674	-0.002349571	-0.002120614	-4.542175363	1.92E-05	0.208698337	-0.059423324
cg23345697	-0.003347285	-0.004816384	-0.001878186	0.003481488	-4.533450706	1.98E-05	0.208698337	-0.065371022
cg17075545	0.001976323	0.001107342	0.002845304	0.003883588	4.525172052	2.05E-05	0.208698337	-0.126452769
cg13515254	0.004986214	0.002788198	0.007184229	0.003105647	4.513797777	2.14E-05	0.211165224	-0.146079134
cg00440448	-0.003248552	-0.004683471	-0.001813633	-0.002909961	-4.504533441	2.21E-05	0.211165224	-0.174363899
cg23385218	0.002444274	0.001359733	0.003528816	-0.004138877	4.484262027	2.39E-05	0.211165224	-0.217205004
cg15939854	-0.00344281	-0.004975535	-0.001910085	0.002005083	-4.469264964	2.53E-05	0.211165224	-0.331884757
cg25093756	0.004365226	0.002420323	0.00631013	0.010749777	4.465832731	2.56E-05	0.211165224	-0.322572656
cg26627320	0.001984699	0.001100139	0.00286926	-0.000626734	4.46431117	2.57E-05	0.211165224	-0.323421545
cg12632785	-0.005570505	-0.008065547	-0.003075463	-0.017811553	-4.442409693	2.80E-05	0.211165224	-0.414817947
cg26043100	0.003693378	0.002037568	0.005349188	-0.002785162	4.438137018	2.84E-05	0.211165224	-0.424873143
cg17739400	0.002298834	0.001267021	0.003330646	0.002331481	4.432962324	2.89E-05	0.211165224	-0.463916261
cg05265071	0.003810946	0.002098137	0.005523755	0.013762145	4.427017622	2.96E-05	0.211165224	-0.489196711
cg03024212	-0.004385976	-0.006359196	-0.002412756	-0.009589624	-4.422694672	3.01E-05	0.211165224	-0.489152191
cg19107973	-0.005667427	-0.008220664	-0.003114191	-0.007755636	-4.416689304	3.08E-05	0.211165224	-0.51638172
cg09102332	-0.005271233	-0.007652839	-0.002889627	-0.008312432	-4.403969656	3.23E-05	0.211165224	-0.555861429
cg13044649	-0.004141779	-0.006017279	-0.00226628	-0.003439563	-4.394018751	3.35E-05	0.211165224	-0.595443548
cg23515090	-0.002543738	-0.00369591	-0.001391565	-0.004116542	-4.392807133	3.36E-05	0.211165224	-0.586342949
cg23938645	0.003212393	0.00175564	0.004669146	0.009877404	4.387631887	3.42E-05	0.211165224	-0.59534327
cg17602556	0.002156483	0.001178083	0.003134882	0.003783433	4.385478019	3.45E-05	0.211165224	-0.600924574
cg10832763	-0.002990448	-0.004347351	-0.001633545	-0.004441383	-4.385051867	3.46E-05	0.211165224	-0.590120458
cg18199664	0.001841213	0.001005555	0.002676871	0.002605034	4.383921793	3.47E-05	0.211165224	-0.647183646
cg17351081	0.00400849	0.002188954	0.005828026	0.008074094	4.383391418	3.48E-05	0.211165224	-0.640936672
cg10655125	0.00383414	0.002091379	0.005576901	0.007190216	4.377413028	3.56E-05	0.211165224	-0.657307504
cg20513867	0.004361924	0.002378248	0.0063456	0.012553721	4.375273202	3.59E-05	0.211165224	-0.674386587
cg15035421	-0.005841556	-0.008503173	-0.003179938	0.004456699	-4.367016439	3.70E-05	0.211570967	-0.683256234
cg18085862	-0.002665005	-0.003881437	-0.001448572	0.000868409	-4.359105074	3.81E-05	0.211570967	-0.720714115
cg26544530	-0.00504978	-0.007354713	-0.002744847	-0.009692622	-4.35929321	3.81E-05	0.211570967	-0.731788274
cg06413784	0.003737889	0.002026684	0.005449094	0.00370556	4.346219533	3.99E-05	0.211570967	-0.750211886
cg08573879	0.004211522	0.002283496	0.006139548	0.013250214	4.346311758	3.99E-05	0.211570967	-0.769087306
cg06471104	-0.003663258	-0.005341081	-0.001985435	-0.003241408	-4.344189086	4.02E-05	0.211570967	-0.764861461
cg24957609	-0.004513951	-0.006588249	-0.002439652	-0.002376989	-4.329990083	4.25E-05	0.211570967	-0.832842753
cg14680128	-0.003756632	-0.005483329	-0.002029936	-0.013246849	-4.328825027	4.26E-05	0.211570967	-0.818476103
cg25431820	0.003869258	0.002090441	0.005648075	0.011272275	4.327973376	4.27E-05	0.211570967	-0.831887337
cg11883286	0.002696402	0.00145534	0.003937463	0.007989023	4.322935568	4.35E-05	0.211570967	-0.859863648
cg13070581	-0.005311302	-0.00775841	-0.002864194	0.003243338	-4.318667701	4.43E-05	0.211570967	-0.878357849
cg14727987	-0.003892028	-0.005686121	-0.002097934	-0.006414642	-4.31636822	4.46E-05	0.211570967	-0.857311975
cg13896190	-0.004830446	-0.007061765	-0.002599127	-0.011994405	-4.307522514	4.61E-05	0.211570967	-0.90498822
cg14767360	0.002174005	0.001169431	0.00317858	0.005096536	4.305919835	4.63E-05	0.211570967	-0.894493806
cg00692792	0.00373528	0.002008953	0.005461607	0.011632634	4.305141577	4.64E-05	0.211570967	-0.916670524
cg20861365	-0.005518124	-0.008070953	-0.002965294	-0.005626901	-4.301021325	4.72E-05	0.211570967	-0.920901047
cg12193628	0.002970296	0.001594849	0.004345743	-0.003367205	4.296779666	4.79E-05	0.211570967	-0.944361881
cg05863683	0.00209559	0.001124495	0.003066685	0.000317864	4.293701881	4.84E-05	0.211570967	-0.941004817
cg03755052	-0.001640449	-0.002402717	-0.00087818	-0.002555099	-4.281954772	5.05E-05	0.216425608	-0.966933255
cg27060391	0.001802292	0.000964403	0.002640182	0.001811054	4.279821201	5.09E-05	0.216425608	-0.963649885
cg25345365	-0.003504775	-0.005138075	-0.001871476	-0.003661771	-4.269546217	5.29E-05	0.221637272	-1.056914227
cg03669282	-0.004323869	-0.006341879	-0.002305859	-0.016919998	-4.263291539	5.42E-05	0.223947341	-1.03104114
cg12375635	-0.005615217	-0.00823882	-0.002991615	-0.012804267	-4.258636094	5.52E-05	0.224929128	-1.085380233

cg01211727	-0.001847943	-0.002713216	-0.000982669	0.005491185	-4.249350368	5.69E-05	0.227578445	-1.082993606
cg00200230	-0.005225592	-0.007673142	-0.002778042	0.001520115	-4.248208672	5.73E-05	0.227578445	-1.124682199
cg22043224	0.004596034	0.002438145	0.006753924	0.005133627	4.237952347	5.95E-05	0.232282991	-1.150135551
cg17297475	-0.00392214	-0.005764815	-0.002079465	-0.002614348	-4.235100707	6.00E-05	0.232282991	-1.14445973
cg18422544	0.003776838	0.001998102	0.005555574	0.010211651	4.224785249	6.23E-05	0.238103546	-1.1922682
cg00417823	-0.002623932	-0.003863084	-0.00138478	-0.001227269	-4.21323186	6.49E-05	0.243360704	-1.233182705
cg17746846	0.00279911	0.001476486	0.004121734	0.006117109	4.210860527	6.55E-05	0.243360704	-1.244652223
cg18851966	-0.005594395	-0.008239017	-0.002949774	-0.002310391	-4.209122344	6.60E-05	0.243360704	-1.26254492
cg16123389	-0.003644856	-0.005371279	-0.001918434	-0.005540913	-4.200691399	6.80E-05	0.246625984	-1.292583913
cg08046581	0.002397474	0.001260185	0.003534763	0.008100752	4.194406795	6.95E-05	0.246625984	-1.292002023
cg02124012	-0.003849577	-0.005675908	-0.002023246	-0.005850299	-4.193955048	6.97E-05	0.246625984	-1.309610328
cg23157375	0.003746535	0.001968261	0.005524808	0.010166867	4.191984912	7.01E-05	0.246625984	-1.317368339
cg04431935	0.005981686	0.00313849	0.008824882	0.002163951	4.186188505	7.18E-05	0.249405254	-1.316424976
cg08421007	0.003570513	0.001870463	0.005270563	0.011976851	4.178845721	7.36E-05	0.25277836	-1.35772206
cg07646791	-0.002297859	-0.003392793	-0.001202924	-0.009474247	-4.175634878	7.44E-05	0.252885712	-1.356901927
cg09753291	-0.001866135	-0.002756515	-0.000975756	-0.002480617	-4.170185283	7.59E-05	0.255075855	-1.354569464
cg08599521	0.003111335	0.001625344	0.004597327	0.000219164	4.165986519	7.70E-05	0.256154242	-1.382351176
cg11577329	0.002949126	0.001538237	0.004360016	0.007485545	4.158987647	7.90E-05	0.25718871	-1.401701005
cg13851767	0.011239453	0.005862737	0.016616169	0.008706308	4.15939399	7.90E-05	0.25718871	-1.412250601
cg02363950	0.001771088	0.000921522	0.002620655	0.002544835	4.147917239	8.22E-05	0.261917008	-1.460017596
cg26402776	-0.002481809	-0.003672827	-0.001290791	-0.001057719	-4.146075816	8.28E-05	0.261917008	-1.457517945
cg18785598	-0.003636816	-0.005382565	-0.001891068	-0.001155847	-4.145028135	8.31E-05	0.261917008	-1.488383695
cg14140920	-0.005512441	-0.008162166	-0.002862716	-0.013689319	-4.139473765	8.49E-05	0.261917008	-1.505158139
cg25748764	-0.002782103	-0.004119574	-0.001444632	-0.005223964	-4.138816061	8.50E-05	0.261917008	-1.498197309
cg25723645	0.002365137	0.00122737	0.003502904	0.000395366	4.136094173	8.58E-05	0.261917008	-1.509146921
cg01797381	-0.002952835	-0.004375555	-0.001530115	-0.000345609	-4.12959241	8.78E-05	0.261917008	-1.538424202
cg11082424	-0.002109011	-0.003125257	-0.001092765	-0.004686727	-4.129212607	8.79E-05	0.261917008	-1.524853622
cg01413771	-0.002320094	-0.003438959	-0.001201229	0.002364106	-4.12586867	8.90E-05	0.261917008	-1.542826977
cg00183886	0.004668432	0.002416867	0.006919996	0.012618454	4.125613968	8.92E-05	0.261917008	-1.521274296
cg18391577	0.004094912	0.002118908	0.006070916	0.004097363	4.123392575	8.99E-05	0.261917008	-1.565107036
cg03618247	0.003646995	0.001885197	0.005408792	0.0121349	4.118760707	9.13E-05	0.263464262	-1.551486768
cg22646083	0.002409781	0.001240537	0.003579025	0.007133125	4.100715885	9.74E-05	0.278416028	-1.633213944
cg04662532	-0.005637277	-0.00837434	-0.002900214	-0.001589228	-4.098138182	9.85E-05	0.278860633	-1.61109645
cg24090369	-0.002242963	-0.003333698	-0.001152229	0.002491042	-4.091574302	0.00010063	0.282381006	-1.619159031
cg10592521	-0.004671458	-0.006944684	-0.002398232	-0.012200961	-4.088949945	0.000101747	0.282920278	-1.671717087
cg20088302	0.00284519	0.001459613	0.004230768	0.002890164	4.085712804	0.000102757	0.283154992	-1.66098992
cg08329004	0.00294039	0.00150726	0.004373519	0.014342694	4.082318324	0.000104009	0.284045032	-1.693054453
cg01401641	-0.004497288	-0.006690768	-0.002303807	0.001704457	-4.079611532	0.000105192	0.284733429	-1.710434965
cg25934581	0.0033696	0.001723621	0.005015579	0.014526937	4.073253075	0.000107424	0.285356683	-1.727489816
cg10097667	-0.002586497	-0.00385048	-0.001322514	-0.005080465	-4.071534792	0.000108083	0.285356683	-1.728646356
cg01280360	-0.003325327	-0.004950508	-0.001700146	-0.000661004	-4.071176748	0.000108221	0.285356683	-1.740308843
cg14558793	0.003459605	0.001765744	0.005153465	0.012131769	4.063836602	0.000111085	0.289003526	-1.735129725
cg24624576	0.00215418	0.001099202	0.003209158	0.00481454	4.062803713	0.000111494	0.289003526	-1.749118685
cg10296382	0.002608498	0.001329577	0.003887419	0.005151431	4.058206569	0.000113331	0.290697608	-1.792942976
cg19741727	0.003273262	0.001666449	0.004880076	-0.005134629	4.053243473	0.000115347	0.290697608	-1.754664572
cg10311700	-0.004773929	-0.007118083	-0.002429774	-0.021583866	-4.052206814	0.00011596	0.290697608	-1.793082361
cg16343134	0.001983139	0.001007924	0.002958355	0.00422948	4.046132325	0.000118296	0.290697608	-1.800657273
cg18632412	0.001855732	0.000942478	0.002768986	0.001734964	4.043066166	0.000119589	0.290697608	-1.78759077
cg15074095	0.003437564	0.001745597	0.005129531	0.005509963	4.042464882	0.000119845	0.290697608	-1.7888046
cg21524461	-0.002974397	-0.004438456	-0.001510337	0.005425334	-4.04228922	0.000119919	0.290697608	-1.82388972
cg17828445	-0.002397945	-0.003578775	-0.001217114	-0.004795385	-4.040536333	0.000120667	0.290697608	-1.8215996
cg02568347	-0.004708664	-0.007028436	-0.002388892	0.010296461	-4.038818251	0.000121599	0.290697608	-1.781131053
cg10996267	0.002113388	0.001072092	0.003154684	0.00794698	4.038243491	0.000121651	0.290697608	-1.84063198
cg01238669	-0.005798436	-0.008661227	-0.002935646	-0.010377124	-4.03016931	0.00012538	0.295540991	-1.863710327
cg08985303	0.003654174	0.001848996	0.005459351	0.011436534	4.02769929	0.000126287	0.295540991	-1.858011007
cg01674147	0.00388348	0.00196479	0.00580217	0.011432208	4.027277153	0.000126577	0.295540991	-1.891334111
cg09709457	-0.003275273	-0.004896401	-0.001654145	-0.011595352	-4.019921838	0.000129802	0.300773813	-1.874611043
cg19531032	-0.002479619	-0.003708788	-0.00125045	0.002313952	-4.013846303	0.000132618	0.304129122	-1.93651189
cg07482356	-0.005802281	-0.008680189	-0.002924373	-0.026637027	-4.01165735	0.000133857	0.304129122	-1.947680605
cg22644194	0.002485383	0.001251159	0.003719606	-0.00526681	4.006700246	0.000136006	0.304129122	-1.924519781
cg04197449	0.001664552	0.000837892	0.002491213	0.002474146	4.006430451	0.000136135	0.304129122	-1.933113624
cg13075971	0.005006873	0.00251872	0.007495027	0.006010335	4.003975163	0.000137534	0.304129122	-1.977434204
cg00533683	-0.004600026	-0.006888035	-0.002312017	-0.010503901	-4.000409776	0.000139273	0.304129122	-1.955207886
cg25224594	0.002989031	0.00150214	0.004475923	0.006063158	3.999801679	0.000139355	0.304129122	-1.978610583
cg05901679	0.00482852	0.002423393	0.007233647	0.014855966	3.994642623	0.000142131	0.304129122	-1.991671222
cg09660432	0.004668317	0.002341829	0.006994804	0.003816467	3.992652668	0.00014313	0.304129122	-1.961226822
cg09105683	0.002824613	0.001416251	0.004232975	-0.00133912	3.99054376	0.000143974	0.304129122	-1.987358871
cg01947787	-0.003267249	-0.0048965	-0.001637999	-0.011457246	-3.990080825	0.000144209	0.304129122	-2.013054873
cg08824395	0.004087959	0.002048028	0.00612789	0.015057939	3.987407881	0.00014576	0.304129122	-2.017481444
cg26583478	-0.005216017	-0.007818835	-0.002613199	-0.015871042	-3.987468147	0.000145765	0.304129122	-2.010254678
cg17885063	0.004435586	0.002218371	0.006652802	0.007965816	3.980567874	0.000149344	0.304129122	-2.032779043
cg12226826	-0.002555925	-0.003834132	-0.001277718	-9.75E-05	-3.978636661	0.000150131	0.304129122	-2.02543756
cg10023249	0.00331906	0.001658929	0.004979191	0.004569185	3.97795791	0.00015049	0.304129122	-2.049178688
cg18816129	0.003781733	0.00188988	0.005673586	0.014211405	3.977368677	0.00015088	0.304129122	-2.043049582
cg23042590	-0.004016286	-0.006026426	-0.002006146	-0.010934762	-3.975545323	0.000151944	0.304129122	-2.057347261
cg18200075	0.002876229	0.001436367	0.004316091	0.007874613	3.974568507	0.000152292	0.304129122	-2.026903373
cg06850054	-0.004039179	-0.006061313	-0.002017045	-0.015564469	-3.974489689	0.000152508	0.304129122	-2.054438002

cg22171860	0.002997618	0.001496797	0.004498438	0.006996592	3.974063578	0.000152562	0.304129122	-2.065301018
cg13714540	0.003790845	0.001892436	0.005689255	0.010414076	3.973191236	0.000153125	0.304129122	-2.063774467
cg06036236	0.002608422	0.001301409	0.003915435	0.008440618	3.970865894	0.000154285	0.304456335	-2.04545547
cg23507839	0.003376142	0.001682472	0.005069811	0.007414089	3.966242915	0.000156808	0.306172622	-2.091472814
cg18322605	0.002574141	0.001281716	0.003866566	0.006573663	3.962910264	0.000158651	0.306172622	-2.094048266
cg21471396	-0.004403004	-0.006614501	-0.002191506	-0.010344296	-3.961544864	0.000159652	0.306172622	-2.097377485
cg23566322	0.003951179	0.001966553	0.005935806	0.01412338	3.961360573	0.000159665	0.306172622	-2.094054818
cg02668218	0.002618639	0.001302976	0.003934303	0.012151238	3.960210604	0.000160159	0.306172622	-2.082256693
cg18513062	0.002420353	0.001201339	0.003639367	0.010230018	3.950547404	0.000165672	0.314743799	-2.138659461
cg18337525	-0.00296402	-0.004459469	-0.00146857	-0.004009434	-3.943633946	0.000169727	0.319618954	-2.173369635
cg24923412	0.004598532	0.002275394	0.00692167	-0.008953648	3.938638275	0.000172971	0.319618954	-2.139273072
cg06203221	-0.003207379	-0.00482889	-0.001585869	1.24E-05	-3.935663292	0.00017452	0.319618954	-2.197078753
cg22535754	0.003599239	0.001779018	0.00541946	0.008480567	3.934372356	0.000175335	0.319618954	-2.181218274
cg22275570	-0.001725013	-0.002597426	-0.000852599	-0.003109052	-3.934207015	0.00017541	0.319618954	-2.189805348
cg21620743	0.004720719	0.002332736	0.007108702	0.015592204	3.933497745	0.000176103	0.319618954	-2.191343842
cg21949305	0.002361175	0.001165934	0.003556416	0.009444824	3.930611372	0.000177625	0.319618954	-2.208321373
cg15323871	-0.002946995	-0.004438958	-0.001455031	-0.001710941	-3.930143614	0.000177915	0.319618954	-2.208516883
cg12824877	0.002769452	0.001366807	0.004172096	0.009317497	3.928561989	0.000178899	0.319618954	-2.217935304
cg21964252	0.001993352	0.000982882	0.003003822	0.00346563	3.925074338	0.000181089	0.319618954	-2.231092383
cg08904394	-0.002766951	-0.004169671	-0.001364232	-0.004263176	-3.924804829	0.000181259	0.319618954	-2.192663756
cg02897989	-0.003939583	-0.005937099	-0.001942066	-0.013556149	-3.924258919	0.000181794	0.319618954	-2.229344161
cg02497258	-0.002874486	-0.004332659	-0.001416314	-0.002604013	-3.922279979	0.000182861	0.319618954	-2.223835767
cg24889693	0.002954073	0.001454721	0.004453424	0.00033583	3.920171505	0.000184209	0.319618954	-2.246530838
cg25707676	-0.003218047	-0.00485216	-0.001583934	-0.0185689	-3.918298571	0.000185415	0.319618954	-2.254675094
cg16106997	-0.002279903	-0.003437639	-0.001122167	-0.001213083	-3.918268036	0.000185435	0.319618954	-2.204241782
cg11064411	0.004804245	0.002363557	0.007244933	-0.006408055	3.916650652	0.000186753	0.319618954	-2.242937675
cg19147608	-0.003036584	-0.004579736	-0.001493432	-0.012178652	-3.915290039	0.000187368	0.319618954	-2.251025191
cg11619216	-0.002540609	-0.003832116	-0.001249101	-0.003621838	-3.914066582	0.000188168	0.319618954	-2.261929429
cg02958895	0.00221306	0.00108745	0.003338669	0.001552505	3.911946099	0.000189561	0.319618954	-2.280098265
cg03787988	-0.002668048	-0.004025401	-0.001310696	-0.008017988	-3.911006213	0.000190182	0.319618954	-2.233440446
cg09858862	-0.00774155	-0.011685557	-0.003797542	-0.013528328	-3.905639191	0.000194043	0.322646904	-2.303552099
cg18348274	0.002493787	0.001223187	0.003764386	0.008804225	3.905152178	0.000194094	0.322646904	-2.298155521
cg22870710	0.003944662	0.001932469	0.005956856	0.016348651	3.900665846	0.000197362	0.326307066	-2.31247561
cg03159960	0.003129357	0.001530362	0.004728353	0.011784824	3.893993923	0.000201763	0.329832957	-2.336612257
cg13783936	0.003369709	0.001647148	0.00509227	0.004797627	3.892290228	0.00020296	0.329832957	-2.336471218
cg07256732	0.002065494	0.001009592	0.003121396	0.005789681	3.892133351	0.00020307	0.329832957	-2.324560931
cg12778228	0.005615682	0.002740969	0.008490396	-0.00120402	3.886958122	0.00020704	0.329832957	-2.332777431
cg27308932	-0.003581866	-0.005416042	-0.001747691	0.001298525	-3.885596232	0.000207761	0.329832957	-2.339656588
cg09440616	-0.004445994	-0.006723021	-0.002168967	-0.013075134	-3.885103747	0.000208374	0.329832957	-2.34864822
cg10765212	0.002980026	0.00145311	0.004506942	0.002309435	3.88322198	0.000209442	0.329832957	-2.334915757
cg00946492	0.002992046	0.001457893	0.0045262	0.003850184	3.880492569	0.000211432	0.329832957	-2.370852329
cg01562540	0.005430096	0.002645924	0.008214267	0.011183166	3.880730391	0.000211554	0.329832957	-2.371626229
cg06107488	-0.001725339	-0.002610618	-0.00084006	-0.005174724	-3.877767039	0.000213436	0.329832957	-2.376695991
cg18329967	-0.002134358	-0.00322952	-0.001039196	0.000471707	-3.877719048	0.000213472	0.329832957	-2.354837333
cg11799593	-0.003681271	-0.00557144	-0.001791103	-0.00802469	-3.87516093	0.000215474	0.329832957	-2.385080162
cg06167953	0.002172818	0.001056969	0.003288667	0.003513426	3.874407769	0.000215932	0.329832957	-2.38313416
cg05163329	-0.005545564	-0.008393621	-0.002697507	-0.028918347	-3.874351059	0.000216277	0.329832957	-2.375707952
cg13693582	0.005437751	0.002642968	0.008232534	0.011357021	3.871446309	0.00021846	0.329832957	-2.407509835
cg26620655	-0.008839067	-0.013384526	-0.004293608	-0.024352716	-3.869284589	0.000220099	0.329832957	-2.421359875
cg04425546	-0.004619088	-0.006994705	-0.002243471	-0.00948861	-3.868849129	0.00022043	0.329832957	-2.41010242
cg22706007	0.004843788	0.002352372	0.007335205	0.008902397	3.868483312	0.000220709	0.329832957	-2.404471908
cg13730689	0.002656194	0.001289814	0.004022574	0.00944618	3.867904141	0.000220844	0.329832957	-2.409752407
cg09277532	-0.003747126	-0.005676204	-0.001818047	-0.006100081	-3.864943947	0.000223275	0.329832957	-2.42592749
cg08153213	-0.003710629	-0.005621419	-0.001799839	-0.008981338	-3.863889038	0.000223988	0.329832957	-2.383193661
cg21229153	0.004633647	0.002246937	0.007020358	0.002962712	3.863004172	0.000224926	0.329832957	-2.427542274
cg12571016	-0.003125145	-0.004734994	-0.001515297	0.008507831	-3.862536782	0.000224978	0.329832957	-2.351981819
cg14571669	-0.00298796	-0.004527678	-0.001448243	-0.004693393	-3.861189159	0.000226028	0.329832957	-2.432058412
cg27624684	0.002844727	0.001378036	0.004311418	0.010917076	3.859128923	0.000227641	0.329832957	-2.425076246
cg01844657	0.004071448	0.001971045	0.006171851	0.009782418	3.856987664	0.000229645	0.329832957	-2.453380079
cg09023643	0.001697209	0.000821545	0.002572872	0.001036111	3.856428989	0.000229772	0.329832957	-2.424407394
cg26332488	0.002424361	0.001172491	0.003676232	0.009351185	3.853233504	0.000232319	0.329832957	-2.405002238
cg13638229	-0.003571539	-0.005416214	-0.001726864	0.002589302	-3.852331897	0.000233059	0.329832957	-2.428317901
cg08284564	0.003291522	0.001591398	0.004991646	0.008630374	3.852152743	0.000233187	0.329832957	-2.4513527
cg08643154	0.003131886	0.001510265	0.004753506	0.007071711	3.842766014	0.000240853	0.329832957	-2.472613775
cg03862353	-0.003552725	-0.005392667	-0.001712782	-0.008587183	-3.841912956	0.000241626	0.329832957	-2.495759173
cg10288541	0.002221387	0.001070823	0.003371951	-0.000905721	3.841500246	0.000241905	0.329832957	-2.47258169
cg25579393	-0.002467191	-0.003745185	-0.001189196	0.001723321	-3.841147388	0.000242199	0.329832957	-2.515129692
cg07675216	-0.007224429	-0.01097003	-0.003478827	-0.033977838	-3.837813669	0.000245325	0.329832957	-2.511242526
cg08042370	-0.004009464	-0.006089238	-0.00192969	-0.011246798	-3.835942779	0.000246909	0.329832957	-2.530107554
cg14196263	-0.006243871	-0.0094827	-0.003005042	-0.025966138	-3.835905365	0.000246941	0.329832957	-2.518123412
cg16415116	0.0047233	0.002272307	0.007174292	0.012640945	3.834470835	0.000248162	0.329832957	-2.532308377
cg07481886	0.002334463	0.001122349	0.003546577	0.005724999	3.832048112	0.0002499	0.329832957	-2.54449802
cg12796916	-0.002937213	-0.004462759	-0.001411667	0.000972934	-3.830869664	0.000250915	0.329832957	-2.550277008
cg13513346	-0.005565518	-0.008456652	-0.002674384	-0.022560815	-3.830357385	0.000251695	0.329832957	-2.539424201
cg15892950	0.004395373	0.002110912	0.006679833	0.017920049	3.828370695	0.000253418	0.329832957	-2.544771088
cg08233217	-0.002473984	-0.003759961	-0.001188007	0.00059909	-3.82781622	0.000253561	0.329832957	-2.561947876
cg19047340	0.003797867	0.001823599	0.005772135	0.009278445	3.82763382	0.000253937	0.329832957	-2.54590819

cg15830564	0.00375858	0.00180458	0.00571258	0.006836994	3.827331164	0.000254203	0.329832957	-2.558484628
cg06863289	0.003346283	0.00160616	0.005086405	0.01287643	3.826221857	0.000254954	0.329832957	-2.555382707
cg02336104	-0.002639483	-0.004012983	-0.001265984	-0.001231041	-3.823647153	0.000257218	0.329832957	-2.522549452
cg02733596	-0.003956004	-0.00601533	-0.001896679	0.002037401	-3.822376865	0.000258682	0.329832957	-2.581472481
cg14855378	0.00345869	0.001657922	0.005259459	-0.001083677	3.821582678	0.000259098	0.329832957	-2.573894264
cg12671990	-0.004691204	-0.007134078	-0.002248331	0.002336716	-3.821073119	0.000259847	0.329832957	-2.578774232
cg11844835	0.004011152	0.001922136	0.006100167	0.004570982	3.820580132	0.000260287	0.329832957	-2.575619224
cg18329418	-0.003524958	-0.00536107	-0.001688847	-0.003776494	-3.819843488	0.000260676	0.329832957	-2.574873897
cg21125095	0.001907542	0.000913765	0.00290132	0.00494543	3.819198556	0.000261175	0.329832957	-2.570201368
cg11684572	-0.002096072	-0.003188577	-0.001003566	0.003311866	-3.817419557	0.000262774	0.329832957	-2.576124199
cg00120481	0.002017922	0.000965963	0.00306988	0.004915592	3.81674362	0.000263384	0.329832957	-2.580726958
cg17906803	0.003578305	0.001711236	0.005445374	0.010773926	3.813360752	0.000266542	0.329832957	-2.581805005
cg21043558	-0.006849565	-0.010424369	-0.00327476	-0.007862794	-3.812524164	0.000267576	0.329832957	-2.562927961
cg19517936	0.003112605	0.001487655	0.004737554	0.008426502	3.811285685	0.000268359	0.329832957	-2.611318492
cg10949345	-0.003343058	-0.005088916	-0.001597199	-0.005293477	-3.809978807	0.000269572	0.329832957	-2.615487678
cg16746274	-0.003786844	-0.005764406	-0.001809282	-0.013695239	-3.810159665	0.000269606	0.329832957	-2.592684683
cg12978573	0.002336268	0.00111591	0.003556626	0.007197954	3.809103737	0.000270372	0.329832957	-2.585532687
cg05100432	0.00191812	0.000916051	0.002920189	0.006391188	3.808601536	0.000270838	0.329832957	-2.612074274
cg11135661	0.004708459	0.002247678	0.00716924	0.008997159	3.807218436	0.000272482	0.329832957	-2.621256323
cg12417071	-0.003389713	-0.005162199	-0.001617226	-0.007157762	-3.805110869	0.000274095	0.329832957	-2.603356387
cg17226870	-0.00317172	-0.004830363	-0.001513076	-0.002074303	-3.804775609	0.000274409	0.329832957	-2.58852593
cg22521522	0.002043506	0.000974376	0.003112635	0.002525584	3.803058156	0.000276027	0.329832957	-2.613034968
cg17601661	0.00291471	0.001389575	0.004439844	0.011760073	3.802546654	0.000276511	0.329832957	-2.626976668
cg16987606	0.00368862	0.001758475	0.005618765	0.006746156	3.802486629	0.000276727	0.329832957	-2.619665575
cg13249833	0.005449988	0.002598079	0.008301896	0.016438612	3.802435402	0.000276979	0.329832957	-2.634375738
cg08749443	0.001956296	0.000932465	0.002980127	0.002379237	3.801837511	0.000277183	0.329832957	-2.611037427
cg27311376	0.002490258	0.001186863	0.003793654	-0.001363751	3.801504958	0.000277498	0.329832957	-2.639357988
cg08562586	0.005526631	0.002633921	0.008419342	-0.008000704	3.801521851	0.000277846	0.329832957	-2.585730183
cg14872074	-0.003616165	-0.005509291	-0.001723039	0.002788452	-3.800666959	0.000278391	0.329832957	-2.614485054
cg06330917	0.002456721	0.001170241	0.0037432	0.005535033	3.799620694	0.000279293	0.329832957	-2.632150387
cg21372885	0.003034087	0.001444458	0.004623717	0.005641459	3.797690566	0.000281143	0.330740425	-2.612369935
cg19388312	0.001705955	0.000811414	0.002600496	0.007943261	3.794502035	0.000284224	0.333084287	-2.649654577
cg25058019	-0.004500576	-0.006862064	-0.002139089	-0.01172989	-3.792140372	0.0002869	0.334936576	-2.650067794
cg24719399	-0.005605956	-0.008551963	-0.002659948	-0.023863824	-3.786323634	0.000292654	0.336819418	-2.680569583
cg26088680	0.002533736	0.001201963	0.003865508	0.011660927	3.785459654	0.000293139	0.336819418	-2.673577277
cg13520102	0.003714358	0.001760807	0.00566791	0.011470857	3.783162269	0.00029567	0.336819418	-2.692788115
cg13633856	-0.00289296	-0.004415514	-0.001370407	-0.005501312	-3.780569573	0.00029807	0.336819418	-2.680505983
cg02670082	0.002541214	0.001203644	0.003878784	0.003398123	3.780175865	0.000298471	0.336819418	-2.700538077
cg09821769	-0.004248785	-0.006487638	-0.002009931	0.007858469	-3.776078589	0.000303057	0.336819418	-2.704790178
cg07107420	-0.004797341	-0.007325381	-0.002269301	-0.009644422	-3.775883757	0.000303258	0.336819418	-2.738287969
cg21467935	0.00337574	0.001596726	0.005154754	0.007821622	3.775529401	0.000303262	0.336819418	-2.719029352
cg06250076	-0.003135634	-0.004788214	-0.001483055	-0.002350674	-3.775290989	0.000303482	0.336819418	-2.696380584
cg22982242	0.003090589	0.001461214	0.004719964	0.008648207	3.774049755	0.000304768	0.336819418	-2.721347984
cg14128411	0.002228534	0.001053541	0.003403528	0.005883762	3.773733605	0.000305097	0.336819418	-2.724803843
cg18325841	-0.004448914	-0.006794825	-0.002103004	-0.006603993	-3.773501887	0.000305728	0.336819418	-2.739118955
cg03888064	0.001964527	0.000928502	0.003000552	0.006989711	3.772897265	0.000305967	0.336819418	-2.708709598
cg01252023	-0.0033471	-0.005112453	-0.001581748	-0.003197155	-3.772460719	0.000306423	0.336819418	-2.708477225
cg10453977	0.00183903	0.000869033	0.002809027	0.001302892	3.772297455	0.000306593	0.336819418	-2.731452449
cg19766489	-0.003390976	-0.005179641	-0.001602312	-0.012614908	-3.772118236	0.000306836	0.336819418	-2.741270678
cg18095555	0.002419799	0.001142712	0.003696886	0.004512994	3.770041139	0.000308958	0.336819418	-2.745004485
cg18607411	0.003636754	0.001717036	0.005556472	0.010701802	3.769379511	0.000309818	0.336819418	-2.728524596
cg19035457	0.002563955	0.001210285	0.003917625	0.004811319	3.768642177	0.000310433	0.336819418	-2.669507191
cg16711721	0.002847995	0.001344148	0.004351842	0.007333466	3.76810407	0.000311002	0.336819418	-2.72344844
cg27257453	0.003702076	0.001746959	0.005657194	0.004302611	3.767583938	0.000311637	0.336819418	-2.680748386
cg14551255	0.002362458	0.001114139	0.003610778	0.001175562	3.76552704	0.000313741	0.337899218	-2.722477379
cg19975218	0.00219037	0.001032397	0.003348344	0.00528855	3.763624774	0.000315778	0.338815962	-2.742810377
cg15882149	-0.002662696	-0.004072714	-0.001252679	0.003407608	-3.757374433	0.00032256	0.338815962	-2.771276836
cg07733606	0.004000994	0.001882297	0.006119692	0.011043707	3.757517243	0.00032281	0.338815962	-2.768656177
cg09473207	0.004724831	0.002221072	0.00722859	0.018842772	3.754877508	0.000325716	0.338815962	-2.780883729
cg22616933	0.003633931	0.001706253	0.005561609	0.012103257	3.75090256	0.000329906	0.338815962	-2.78749014
cg03225444	0.003203002	0.001503865	0.004902139	-0.00590335	3.750733027	0.000329917	0.338815962	-2.763378582
cg21566086	0.002986226	0.001401943	0.004570508	0.008492748	3.750397351	0.000330294	0.338815962	-2.811137138
cg13366903	-0.004739348	-0.007253894	-0.002224802	0.008849415	-3.750256899	0.000330865	0.338815962	-2.81136617
cg17014186	0.002345437	0.001100935	0.003589938	0.007405261	3.749863984	0.000330892	0.338815962	-2.807685293
cg04211581	-0.005796658	-0.008873539	-0.002719777	-0.013970462	-3.748598747	0.000332731	0.338815962	-2.807321881
cg18225895	-0.005405327	-0.008275192	-0.002535462	-0.007949963	-3.747679613	0.00033377	0.338815962	-2.807512134
cg14810029	-0.004658147	-0.007132239	-0.002184056	-0.015770527	-3.746273052	0.000335366	0.338815962	-2.779944536
cg13932029	-0.001795414	-0.00274915	-0.000841679	-2.37E-05	-3.745625422	0.000335685	0.338815962	-2.787883075
cg11914650	0.002313435	0.00108444	0.00354243	0.004348973	3.745368372	0.000335977	0.338815962	-2.801195624
cg11839940	-0.005069814	-0.00776301	-0.002376619	-0.00277906	-3.745640565	0.000336086	0.338815962	-2.800965114
cg17300125	0.002521204	0.001181714	0.003860694	0.009421145	3.745035572	0.000336357	0.338815962	-2.785820078
cg11007962	-0.006211734	-0.009511834	-0.002911634	-0.013554881	-3.745309229	0.000336464	0.338815962	-2.824517673
cg06675538	-0.002375053	-0.003636939	-0.001113166	-0.004257856	-3.74490112	0.00033651	0.338815962	-2.807870626
cg12671033	0.003739035	0.001752131	0.005725939	0.010832585	3.74436964	0.000337368	0.338815962	-2.802999472
cg15740385	0.003394824	0.001589808	0.00519984	0.007977401	3.742180585	0.000339663	0.338815962	-2.815255903
cg12769599	-0.004069101	-0.006233272	-0.001904931	-0.001980794	-3.741182852	0.000341202	0.338815962	-2.816552244
