# Supplementary material for: DNA methylation changes following narrative exposure therapy in a randomized controlled trial with female former child soldiers
Source: Sci Rep. 2021 Sep 16;11:18493. doi: 10.1038/s41598-021-98067-9 (PMC8445994; doi:10.1038/s41598-021-98067-9)
Supplement: Supplementary file 3 — Supplementary Information 3. [file 41598_2021_98067_MOESM3_ESM.pdf]

DNA methylation changes following Narrative Exposure Therapy in a randomized controlled trial with female former child soldiers

Samuel Carleial, Daniel Nätt, Eva Unternährer, Thomas Elbert, Katy Robjant, Sarah Wilker, Vanja Vukojevic, Iris-Tatjana Kolassa, Anja C. Zeller, and Anke Koebach

---

Supplement S3

Intersection (overlap) of CpGs and genes associated with clinical/social outcomes (PTSD, PHQ, AAS, CVB, AAGS and SAQ) and treatment

---

Intersection of significant associations (FDR=0.05)

For CpG ids

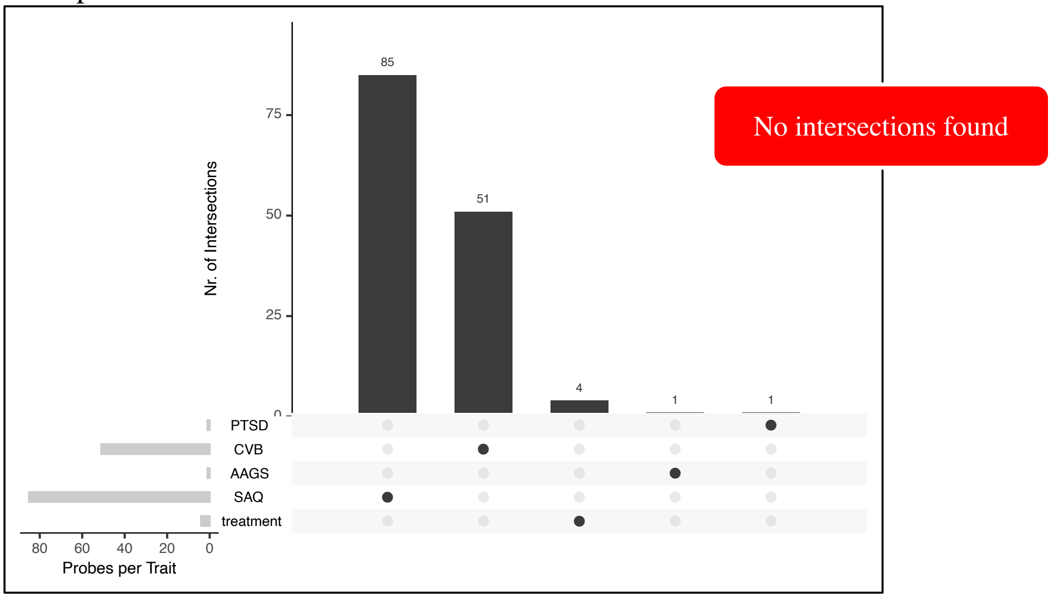

No intersections found

For genes related to associated CpGs

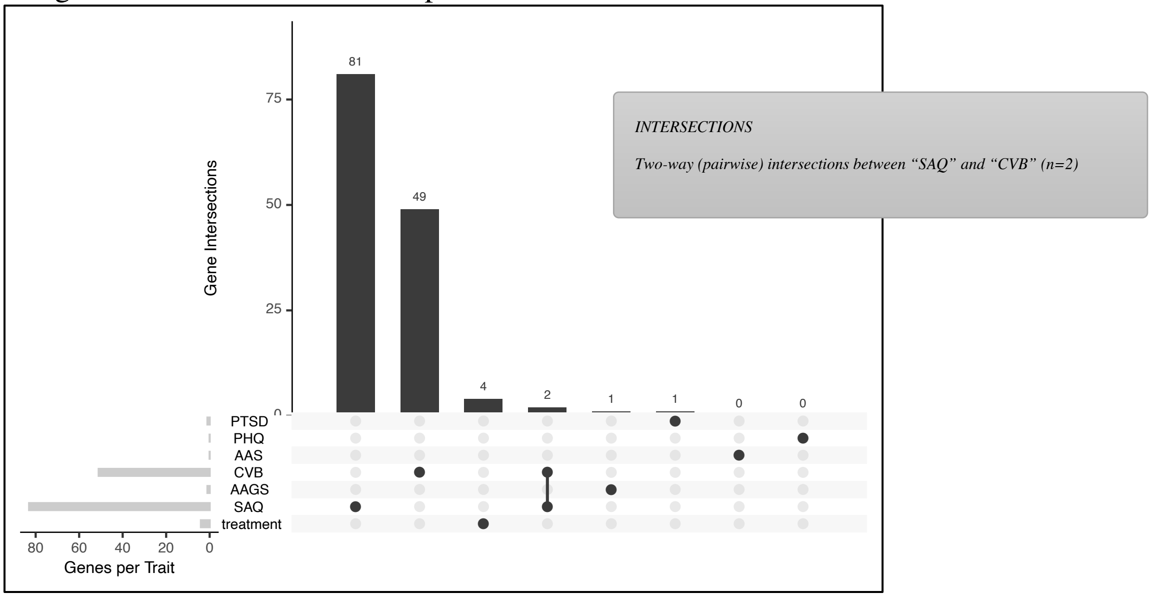

INTERSECTIONS

Two-way (pairwise) intersections between "SAQ" and "CVB" (n=2)

Intersection of top associations (n=305 per association)

For CpG ids

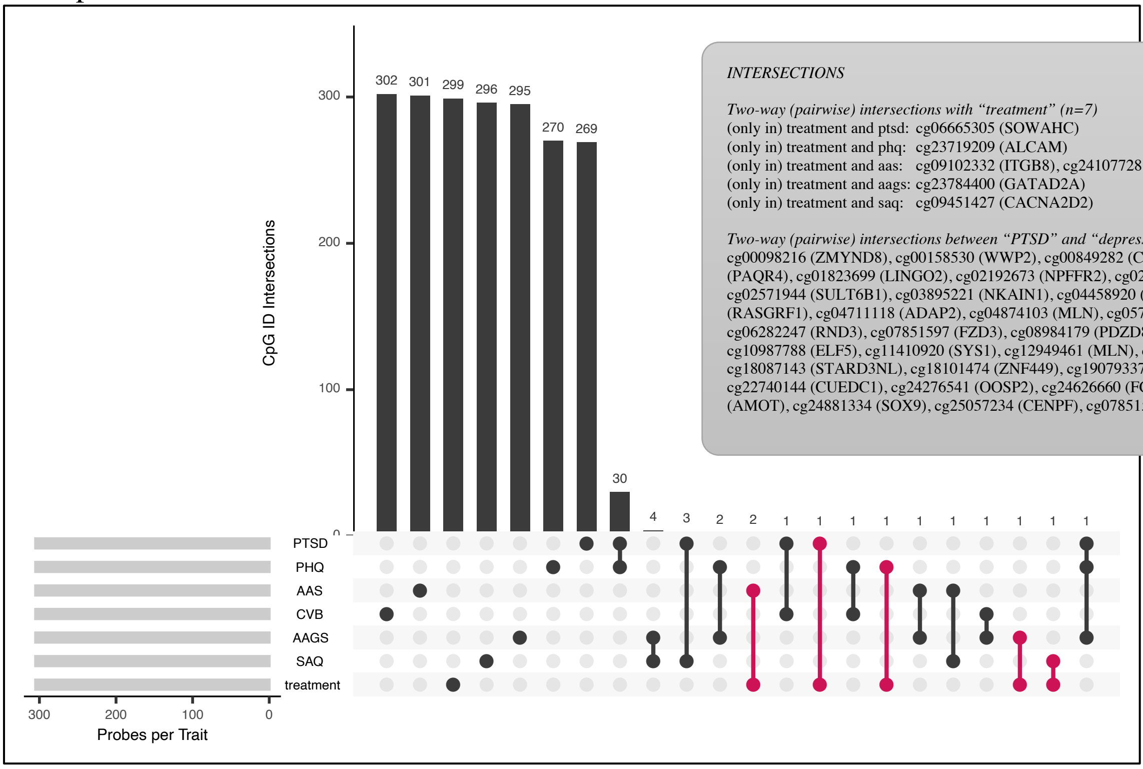

INTERSECTIONS

Two-way (pairwise) intersections with "treatment" (n=7)
(only in) treatment and ptsd: cg06665305 (SOWAHC)
(only in) treatment and phq: cg23719209 (ALCAM)
(only in) treatment and aas: cg09102332 (ITGB8), cg24107728 (LRP8)
(only in) treatment and aags: cg23784400 (GATAD2A)
(only in) treatment and saq: cg09451427 (CACNA2D2)

Two-way (pairwise) intersections between "PTSD" and "depression" (n=31)
cg00098216 (ZMYND8), cg00158530 (WWP2), cg00849282 (CHRDL2), cg01461733 (PAQR4), cg01823699 (LINGO2), cg02192673 (NPFFR2), cg02255004 (ANTXR2), cg02571944 (SULT6B1), cg03895221 (NKAIN1), cg04458920 (TSPYL6), cg04585717 (RASGRF1), cg04711118 (ADAP2), cg04874103 (MLN), cg05773155 (LMOD1), cg06282247 (RND3), cg07851597 (FZD3), cg08984179 (PDZD8), cg09428856 (XKR3), cg10987788 (ELF5), cg11410920 (SYS1), cg12949461 (MLN), cg17351862 (BHLHA9), cg18087143 (STARD3NL), cg18101474 (ZNF449), cg19079337 (EXOSC5), cg22740144 (CUEDC1), cg24276541 (OOSP2), cg24626660 (FGD4), cg24642789 (AMOT), cg24881334 (SOX9), cg25057234 (CENPF), cg07851597 (FZD3)

For genes related to associated CpGs

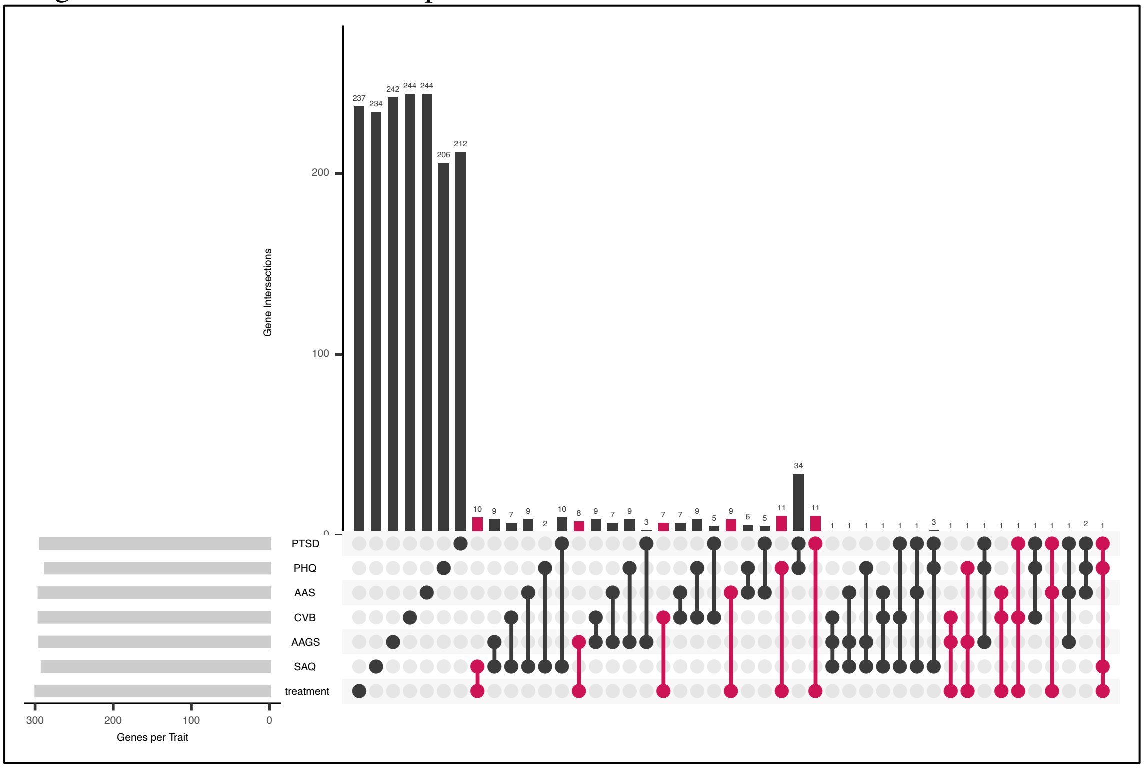

List of genes related with top CpGs after EWAS, in which intersections were found taking treatment into account (previous figure).

Genes found in **intersections with treatment** (n=62):
ABTB2, ACTG1, ADGRD1, ALCAM, ARHGAP10, ASXL2, BOLA3, C1orf87, C9orf47, CACNA2D2, CDH5, CELF1, CFAP46, CHRNA10, COX6B2, CPLX2, DICER1, DPF3, EGFR, EPSTI1, EVI5, EYA1, GATAD2A, GK, GNAS, GPX5, GRID1, IGSF21, INPP5A, IRF8, ITGB8, KIF20B, LBX1, LRP8, LRRC28, LY6H, MAP2K4, MED13L, MICALL2, MTRNR2L1, NKX2-5, PCDHGA4, PNPLA7, PPP2R2A, PTPRN2, RELN, SDHAF3, SHANK2, SIX2, SLCO1A2, SLIT1, SOWAHC, SP9, STK33, SYT7, TBC1D22A, THBS2, TRERF1, TRIB1, WDPCP, WSCD1, ZNF263

with SAQ, PHQ, PTSD: SHANK2
with AAGS, PHQ: SLIT1
with AAGS, CVB: PNPLA7
with AAS, PTSD: ADGRD1
with CVB, PTSD: MICALL2
with AAS, CVB: CFAP46

with SAQ: BOLA3, CACNA2D2, CDH5, CHRNA10, EVI5, GNAS, MAP2K4, SHANK2, SIX2, SLCO1A, SYT7
with AAGS: ARHGAP10, COX6B2, CPLX2, GATAD2A, LBX1, MTRNR2L1, PNPLA7, PPP2R2A, RELN, SLIT1

with CVB: CELF1, CFAP46, DICER1, IGSF21, IRF8, LY6H, MICALL2, PNPLA7, TBC1D22A, ZNF263

with AAS: ADGRD1, CFAP46, DPF3, GK, GPX5, ITGB8, LRP8, PCDHGA4, SP9, TRIB1, WDPCP

with PHQ: ABTB2, ALCAM, C1orf87, EGFR, EPSTI1, EYA1, GRID1, LRRC28, NKX2-5, SHANK2, SLIT1, TRERF1, WSCD1

with PTSD: ACTG1, ADGRD1, ASXL2, C9orf47, INPP5A, KIF20B, MED13L, MICALL2, PTPRN2, SDHAF3, SHANK2, SOWAHC, STK33, THBS2
