# Supplementary material for: DNA methylation changes following narrative exposure therapy in a randomized controlled trial with female former child soldiers
Source: Sci Rep. 2021 Sep 16;11:18493. doi: 10.1038/s41598-021-98067-9 (PMC8445994; doi:10.1038/s41598-021-98067-9)
Supplement: Supplementary file 4 — Supplementary Information 4. [file 41598_2021_98067_MOESM4_ESM.pdf]

DNA methylation changes following Narrative Exposure Therapy in a randomized controlled trial with female former child soldiers

Samuel Carleial, Daniel Nätt, Eva Unternährer, Thomas Elbert, Katy Robjant, Sarah Wilker, Vanja Vukojevic, Iris-Tatjana Kolassa, Anja C. Zeller, and Anke Koebach

---

Supplement S4

Gene networks and gene ontology enrichments for genes found in association with clinical/social outcomes, treatment effect, and the intersection between treatment and outcomes using STRING v11.

Main webtool used: STRING[1]:

1 Szklarczyk, D. *et al*. STRING v11: protein-protein association networks with increased coverage, supporting functional discovery in genome-wide experimental datasets. *Nucleic Acids Research* **47**, D607-D613 (2019).

Content:

Clinical outcomes
- PSS-I (PTSD)
- PHQ-9
- AAS

Social outcomes
- CVB
- AAGS
- SAQ

Treatment effect
- Treatment

⇒ *Intersection: treatment vs. outcomes*

Association with PTSD

Gene list

ABLIM3, ACTG1, ADAP2, ADGRD1, ADRB2, AKIRIN2, ALG10, AMOT, AMPD3, ANKRD44, ANTXR2, ARFGAP3, ARMC10, ARSG, ASIC2, ASXL2, ATP8B1, BABAM1, BHLHA9, BOLA1, BTN3A2, C11orf96, C1QTNF8, C2orf66, C2orf69, C2orf88, C4BPA, C9orf47, CACNG2, CAMK1G, CASP7, CASZ1, CDH13, CEMIP, CENPF, CEP55, CFDP1, CHRD, CHRDL2, COL16A1, COL1A1, CORO7, COX7A1, CPLANE2, CPN2, CSPP1, CTNNA2, CTSE, CUEDC1, CYP1B1, DAAM1, DHRS3, DIS3L2, DOCK9, DOK6, DPRX, DSCAM, DYSF, DZIP1L, E2F3, EGR3, EIF4E, ELF2, ELF5, ELOVL5, ERC2, ERICH1, EVA1A, EXOSC5, F7, FAIM, FAM155A, FAM241B, FAM53A, FAM76B, FAM89A, FBXO27, FBXW7, FCRL6, FGD4, FGF13, FHDC1, FNDC3A, FOXB2, FOXC1, FOXF1, FOXP4, FRG1, FZD3, GABRB3, GAK, GAL3ST1, GALM, GALR1, GLCE, GMDS, GOLGA4, GPR143, GPR6, GRB10, GSDMC, GTF2IRD1, GTPBP1, GYPC, H2BC13, H3C10, HBS1L, HCN1, HLX, HMGN5, HS3ST4, ICA1L, ICAM5, IFNGR1, IFTAP, IGL, IGLL1, IL16, IMMT, INPP5A, ITGAE, KCNF1, KIF20B, KIF26B, KLF6, L3MBTL3, LECT2, LGI1, LHX5, LINGO2, LMBRD1, LMOD1, LRRC14B, LSM3, LTBP3, LYZ, MACROH2A1, MAPK10, MCM3, ME3, MED13L, MFSD14A, MGAT5B, MGRN1, MICALL2, MID1, MIER2, MLN, MOGAT3, MPDU1, MPP7, MRPS5, MRPS7, MSX2, MTCL1, MTUS1, MTX1, MYCBPAP, NBPF12, NCL, NCOR2, NDRG1, NDST2, NDUFAF8, NDUFB8, NDUFC1, NEDD4, NENF, NFIB, NHS, NHSL2, NID1, NKAIN1, NOX5, NPFFR2, NR2F2, OCA2, OLIG2, OOSP2, OSBPL9, P2RY1, PAK1, PAQR4, PCDH7, PDGFA, PDZD8, PDZRN3, PITPNA, PLAC1, PLEKHG4B, PLPP3, POLR1E, PPP1R32, PPP1R9A, PPP2R2C, PSTPIP1, PTPN23, PTPN3, PTPRN2, RAB11FIP1, RAB6C, RAI14, RASGRF1, RET, RGS12, RINL, RNASE10, RND3, RNF115, RP9, RPS6KA2, SATB1, SCPEP1, SDHAF3, SDK1, SEMA6B, SFRP5, SH3PXD2A, SHANK2, SKA1, SLC16A3, SLC16A9, SLC30A2, SLC35A3, SLC5A9, SLC6A20, SLIT2, SND1, SNX7, SOWAHC, SOX13, SOX21, SOX9, SP5, SPACA1, SPNS3, SSU72, STAMBPL1, STARD3NL, STC2, STK3, STK33, STON2, STXBP6, SUGCT, SULT6B1, SUSD1, SV2B, SYNE3, SYS1, TAC4, TAFA5, TATDN3, TCERG1L, TCP11, TENM4, TEX101, THBS2, THSD4, TLR3, TMEM220, TMEM271, TNKS2, TRAPPC12, TRIP13, TSHZ2, TSPYL6, UBASH3B, UHRF1BP1, USP12, VTI1A, WDR27, WDR45B, WDR75, WWP2, XKR3, ZBBX, ZCCHC13, ZDHHC19, ZEB1, ZFHX4, ZFP3, ZG16B, ZMYND8, ZNF185, ZNF221, ZNF449, ZNF496, ZNF627, ZNF648, ZNF766, ZNF782, ZNRF2

Network

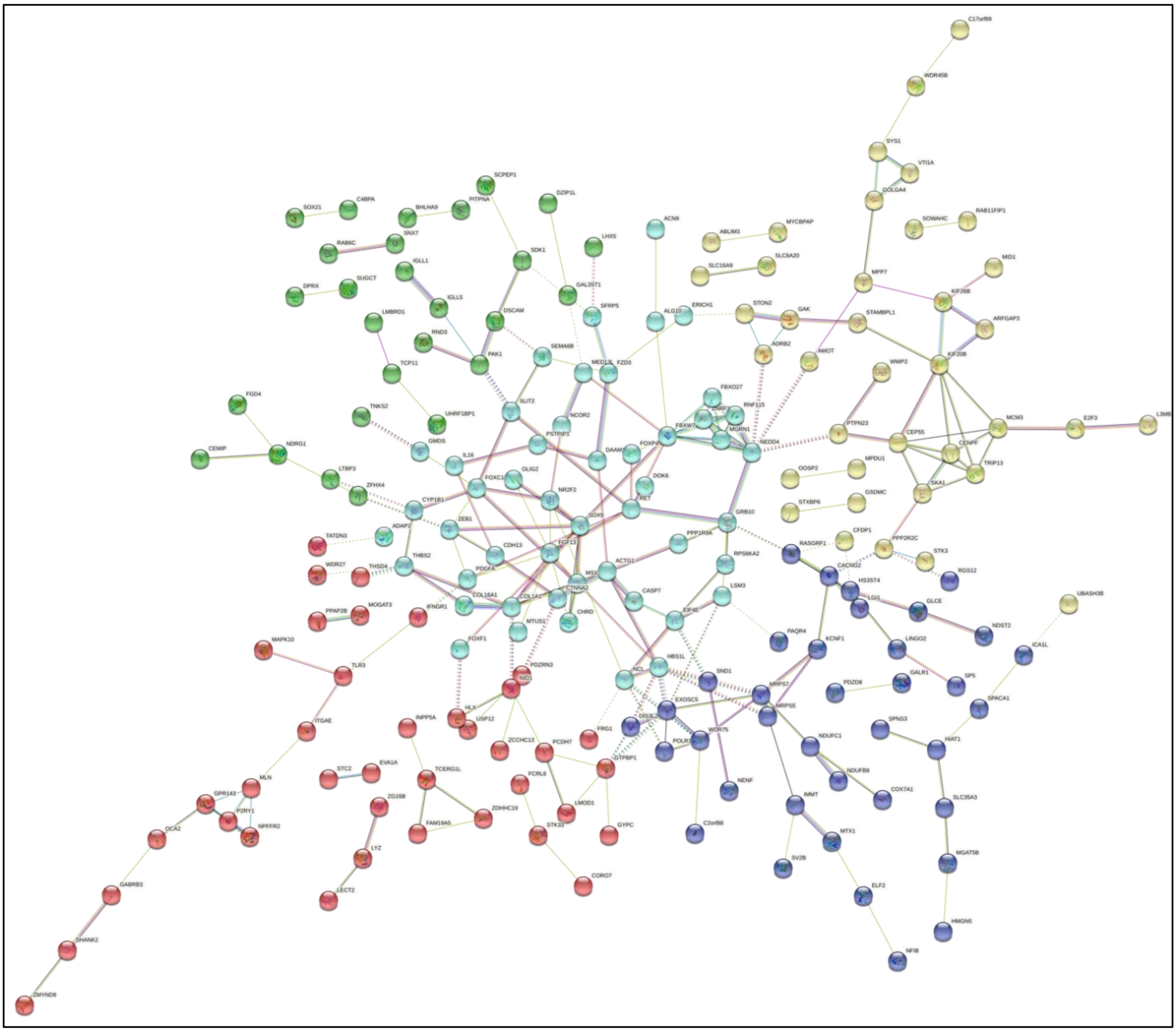

PTSD (PSS-I)

Enrichment

No significant enrichment found

Network Stats

number of nodes:	288	expected number of edges:	203
number of edges:	244	PPI enrichment p-value:	0.00284
average node degree:	1.69	*your network has significantly more interactions than expected (what does that mean?)*	
avg. local clustering coefficient:	0.349		

PTSD (PSS-I)

Mapping

#	queryItem	stringId	preferredName	annotation
1	ABLIM3	9606.ENSP00000425394	ABLIM3	Actin-binding LIM protein 3; May act as scaffold protein. May stimulate ABRA activity and ABRA-dependent SRF transcriptional activity; LIM domain containing
2	ACTG1	9606.ENSP00000458162	ACTG1	Actin, cytoplasmic 2; Actins are highly conserved proteins that are involved in various types of cell motility and are ubiquitously expressed in all eukaryotic cells; Belongs to the actin family
3	ADAP2	9606.ENSP00000329468	ADAP2	Arf-GAP with dual PH domain-containing protein 2; GTPase-activating protein for the ADP ribosylation factor family (Potential). Binds phosphatidylinositol 3,4,5- trisphosphate (PtdInsP3) and inositol 1,3,4,5-tetrakisphosphate (InsP4). Possesses a stoichiometry of two binding sites for InsP4 with identical affinity; ArfGAPs
4	ADGRD1	9606.ENSP00000261654	GPR133	Adhesion G-protein coupled receptor D1; Orphan receptor. Signals via G(s)-alpha family of G- proteins. Has protumorigenic function especially in glioblastoma
5	ADRB2	9606.ENSP00000305372	ADRB2	Beta-2 adrenergic receptor; Beta-adrenergic receptors mediate the catecholamine- induced activation of adenylate cyclase through the action of G proteins. The beta-2-adrenergic receptor binds epinephrine with an approximately 30-fold greater affinity than it does norepinephrine; Belongs to the G-protein coupled receptor 1 family. Adrenergic receptor subfamily. ADRB2 sub-subfamily
6	AKIRIN2	9606.ENSP00000257787	AKIRIN2	Akirin-2; Required for the innate immune response. Downstream effector of the Toll-like receptor (TLR), TNF and IL-1 beta signaling pathways leading to the production of IL-6. Forms a complex with YWHAB that acts to repress transcription of DUSP1 (By similarity); Belongs to the akirin family
7	ALG10	9606.ENSP00000266483	ALG10	Dol-P-Glc:Glc(2)Man(9)GlcNAc(2)-PP-Dol alpha-1,2-glucosyltransferase; Adds the third glucose residue to the lipid-linked oligosaccharide precursor for N-linked glycosylation. Transfers glucose from dolichyl phosphate glucose (Dol-P-Glc) onto the lipid-linked oligosaccharide Glc(2)Man(9)GlcNAc(2)-PP-Dol; Belongs to the ALG10 glucosyltransferase family
8	AMOT	9606.ENSP00000361027	AMOT	Angiomotin; Plays a central role in tight junction maintenance via the complex formed with ARHGAP17, which acts by regulating the uptake of polarity proteins at tight junctions. Appears to regulate endothelial cell migration and tube formation. May also play a role in the assembly of endothelial cell-cell junctions; Belongs to the angiomotin family
9	AMPD3	9606.ENSP00000379802	AMPD3	AMP deaminase 3; AMP deaminase plays a critical role in energy metabolism; Belongs to the metallo-dependent hydrolases superfamily. Adenosine and AMP deaminases family
10	ANKRD44	9606.ENSP00000282272	ANKRD44	Serine/threonine-protein phosphatase 6 regulatory ankyrin repeat subunit B; Putative regulatory subunit of protein phosphatase 6 (PP6) that may be involved in the recognition of phosphoprotein substrates; Ankyrin repeat domain containing
11	ANTXR2	9606.ENSP00000306185	ANTXR2	Anthrax toxin receptor 2; Necessary for cellular interactions with laminin and the extracellular matrix
12	ARFGAP3	9606.ENSP00000263245	ARFGAP3	ADP-ribosylation factor GTPase-activating protein 3; GTPase-activating protein (GAP) for ADP ribosylation factor 1 (ARF1). Hydrolysis of ARF1-bound GTP may lead to dissociation of coatomer from Golgi-derived membranes to allow fusion with target membranes; ArfGAPs
13	ARMC10	9606.ENSP00000319412	ARMC10	Armadillo repeat-containing protein 10; May play a role in cell survival and cell growth. May suppress the transcriptional activity of p53/TP53; Armadillo repeat containing
14	ARSG	9606.ENSP00000407193	ARSG	Arylsulfatase G; Displays arylsulfatase activity at acidic pH with pseudosubstrates, such as p-nitrocatechol sulfate and also, but with lower activity, p-nitrophenyl sulfate and 4- methylumbelliferyl sulfate; Sulfatases
15	ASIC2	9606.ENSP00000225823	ASIC2	Acid sensing ion channel subunit 2
16	ASXL2	9606.ENSP00000383920	ASXL2	Putative Polycomb group protein ASXL2; Putative Polycomb group (PcG) protein. PcG proteins act by forming multiprotein complexes, which are required to maintain the transcriptionally repressive state of homeotic genes throughout development. PcG proteins are not required to initiate repression, but to maintain it during later stages of development. They probably act via methylation of histones, rendering chromatin heritably changed in its expressibility (By similarity). Involved in transcriptional regulation mediated by ligand-bound nuclear hormone receptors, such as peroxisome prolife [...]
17	ATP8B1	9606.ENSP00000445359	ATP8B1	Phospholipid-transporting ATPase IC; Catalytic component of a P4-ATPase flippase complex which catalyzes the hydrolysis of ATP coupled to the transport of aminophospholipids from the outer to the inner leaflet of various membranes and ensures the maintenance of asymmetric distribution of phospholipids. Phospholipid translocation seems also to be implicated in vesicle formation and in uptake of lipid signaling molecules. May play a role in asymmetric distribution of phospholipids in the canicular membrane. May have a role in transport of bile acids into the canaliculus, uptake of bile a [...]
18	BABAM1	9606.ENSP00000352408	BABAM1	BRISC and BRCA1-A complex member 1; Component of the BRCA1-A complex, a complex that specifically recognizes 'Lys-63'-linked ubiquitinated histones H2A and H2AX at DNA lesions sites, leading to target the BRCA1-BARD1 heterodimer to sites of DNA damage at double-strand breaks (DSBs). The BRCA1-A complex also possesses deubiquitinase activity that specifically removes 'Lys-63'-linked ubiquitin on histones H2A and H2AX. In the BRCA1-A complex, it is required for the complex integrity and its localization at DSBs. Component of the BRISC complex, a multiprotein complex that specifically cle [...]
19	BHLHA9	9606.ENSP00000375248	BHLHA9	Class A basic helix-loop-helix protein 9; Transcription factor, which play a role in limb development. Is an essential player in the regulatory network governing transcription of genes implicated in limb morphogenesis; Basic helix-loop-helix proteins
20	BOLA1	9606.ENSP00000358149	BOLA1	BolA-like protein 1; Acts as a mitochondrial iron-sulfur (Fe-S) cluster assembly factor that facilitates (Fe-S) cluster insertion into a subset of mitochondrial proteins (By similarity). Probably acts together with the monothiol glutaredoxin GLRX5. May protect cells against oxidative stress
21	BTN3A2	9606.ENSP00000348751	BTN3A2	Butyrophilin subfamily 3 member A2; Plays a role in T-cell responses in the adaptive immune response. Inhibits the release of IFNG from activated T-cells; Belongs to the immunoglobulin superfamily. BTN/MOG family
22	C11orf96	9606.ENSP00000479976	C11orf96	Uncharacterized protein C11orf96; Chromosome 11 open reading frame 96
23	C1QTNF8	9606.ENSP00000330426	C1QTNF8	Complement C1q tumor necrosis factor-related protein 8; C1q and TNF related 8
24	C2orf66	9606.ENSP00000339384	C2orf66	Uncharacterized protein C2orf66; Chromosome 2 open reading frame 66
25	C2orf69	9606.ENSP00000312770	C2orf69	UPF0565 protein C2orf69; Chromosome 2 open reading frame 69
26	C2orf88	9606.ENSP00000345107	C2orf88	Small membrane A-kinase anchor protein; Binds to type I regulatory subunits of protein kinase A (PKA-RI) and may anchor/target them to the plasma membrane; Belongs to the small membrane AKAP family
27	C4BPA	9606.ENSP00000356037	C4BPA	C4b-binding protein alpha chain; Controls the classical pathway of complement activation. It binds as a cofactor to C3b/C4b inactivator (C3bINA), which then hydrolyzes the complement fragment C4b. It also accelerates the degradation of the C4bC2a complex (C3 convertase) by dissociating the complement fragment C2a. Alpha chain binds C4b. It interacts also with anticoagulant protein S and with serum amyloid P component; Sushi domain containing
28	C9orf47	9606.ENSP00000335616	C9orf47	Uncharacterized protein C9orf47; Chromosome 9 open reading frame 47
29	CACNG2	9606.ENSP00000300105	CACNG2	Voltage-dependent calcium channel gamma-2 subunit; Regulates the trafficking and gating properties of AMPA- selective glutamate receptors (AMPARs). Promotes their targeting to the cell membrane and synapses and modulates their gating properties by slowing their rates of activation, deactivation and desensitization. Does not show subunit-specific AMPA receptor regulation and regulates all AMPAR subunits. Thought to stabilize the calcium channel in an inactivated (closed) state; Belongs to the PMP-22/EMP/MP20 family. CACNG subfamily
30	CAMK1G	9606.ENSP00000009105	CAMK1G	Calcium/calmodulin-dependent protein kinase type 1G; Calcium/calmodulin-dependent protein kinase belonging to a proposed calcium-triggered signaling cascade. In vitro phosphorylates transcription factor CREB1 (By similarity); Belongs to the protein kinase superfamily. CAMK Ser/Thr protein kinase family. CaMK subfamily
31	CASP7	9606.ENSP00000358327	CASP7	Caspase-7; Involved in the activation cascade of caspases responsible for apoptosis execution. Cleaves and activates sterol regulatory element binding proteins (SREBPs). Proteolytically cleaves poly(ADP-ribose) polymerase (PARP) at a '216-Asp-|-Gly- 217' bond. Overexpression promotes programmed cell death
32	CASZ1	9606.ENSP00000366221	CASZ1	Zinc finger protein castor homolog 1; Transcriptional activator. Involved in vascular assembly and morphogenesis through direct transcriptional regulation of EGFL7; Zinc fingers C2H2-type
33	CDH13	9606.ENSP00000268613	CDH13	Cadherin-13; Cadherins are calcium-dependent cell adhesion proteins. They preferentially interact with themselves in a homophilic manner in connecting cells; cadherins may thus contribute to the sorting of heterogeneous cell types. May act as a negative regulator of neural cell growth
34	CEMIP	9606.ENSP00000378177	CEMIP	Cell migration-inducing and hyaluronan-binding protein; Mediates depolymerization of hyaluronic acid (HA) via the cell membrane-associated clathrin-coated pit endocytic pathway. Binds to hyaluronic acid. Hydrolyzes high molecular weight hyaluronic acid to produce an intermediate-sized product, a process that may occur through rapid vesicle endocytosis and recycling without intracytoplasmic accumulation or digestion in lysosomes. Involved in hyaluronan catabolism in the dermis of the skin and arthritic synovium. Positively regulates epithelial- mesenchymal transition (EMT), and hence tu [...]
35	CENPF	9606.ENSP00000355922	CENPF	Centromere protein F; Required for kinetochore function and chromosome segregation in mitosis. Required for kinetochore localization of dynein, LIS1, NDE1 and NDEL1. Regulates recycling of the plasma membrane by acting as a link between recycling vesicles and the microtubule network though its association with STX4 and SNAP25. Acts as a potential inhibitor of pocket protein-mediated cellular processes during development by regulating the activity of RB proteins during cell division and proliferation. May play a regulatory or permissive role in the normal embryonic cardiomyocyte cell cy [...]
36	CEP55	9606.ENSP00000360540	CEP55	Centrosomal protein of 55 kDa; Plays a role in mitotic exit and cytokinesis. Recruits PDCD6IP and TSG101 to midbody during cytokinesis. Required for successful completion of cytokinesis. Not required for microtubule nucleation. Plays a role in the development of the brain and kidney
37	CFDP1	9606.ENSP00000283882	CFDP1	Craniofacial development protein 1; May play a role during embryogenesis
38	CHRD	9606.ENSP00000204604	CHRD	Chordin; Dorsalizing factor. Key developmental protein that dorsalizes early vertebrate embryonic tissues by binding to ventralizing TGF-beta family bone morphogenetic proteins (BMPs) and sequestering them in latent complexes (By similarity)
39	CHRDL2	9606.ENSP00000263671	CHRDL2	Chordin-related protein 2 variant III; Chordin like 2
40	COL16A1	9606.ENSP00000362776	COL16A1	Collagen alpha-1(XVI) chain; Involved in mediating cell attachment and inducing integrin-mediated cellular reactions, such as cell spreading and alterations in cell morphology; Collagens
41	COL1A1	9606.ENSP00000225964	COL1A1	Collagen alpha-1(I) chain; Type I collagen is a member of group I collagen (fibrillar forming collagen); Collagens
42	CORO7	9606.ENSP00000251166	CORO7	Coronin-7; F-actin regulator involved in anterograde Golgi to endosome transport: upon ubiquitination via 'Lys-33'-linked ubiquitin chains by the BCR(KLHL20) E3 ubiquitin ligase complex, interacts with EPS15 and localizes to the trans-Golgi network, where it promotes actin polymerization, thereby facilitating post- Golgi trafficking. May play a role in the maintenance of the Golgi apparatus morphology; Belongs to the WD repeat coronin family
43	COX7A1	9606.ENSP00000292907	COX7A1	Cytochrome c oxidase subunit 7A1, mitochondrial; This protein is one of the nuclear-coded polypeptide chains of cytochrome c oxidase, the terminal oxidase in mitochondrial electron transport
45	CPN2	9606.ENSP00000319464	CPN2	Carboxypeptidase N subunit 2; The 83 kDa subunit binds and stabilizes the catalytic subunit at 37 degrees Celsius and keeps it in circulation. Under some circumstances it may be an allosteric modifier of the catalytic subunit; M14 carboxypeptidases
46	CSPP1	9606.ENSP00000262210	CSPP1	Centrosome and spindle pole-associated protein 1; May play a role in cell-cycle-dependent microtubule organization
47	CTNNA2	9606.ENSP00000384638	CTNNA2	Catenin alpha-2; May function as a linker between cadherin adhesion receptors and the cytoskeleton to regulate cell-cell adhesion and differentiation in the nervous system. Regulates morphological plasticity of synapses and cerebellar and hippocampal lamination during development. Functions in the control of startle modulation; Alpha catenins
48	CTSE	9606.ENSP00000350911	CTSE	Cathepsin E; May have a role in immune function. Probably involved in the processing of antigenic peptides during MHC class II-mediated antigen presentation. May play a role in activation-induced lymphocyte depletion in the thymus, and in neuronal degeneration and glial cell activation in the brain; Cathepsins
49	CUEDC1	9606.ENSP00000462717	CUEDC1	CUE domain containing 1
50	CYP1B1	9606.ENSP00000478561	CYP1B1	Cytochrome P450 1B1; Cytochromes P450 are a group of heme-thiolate monooxygenases. In liver microsomes, this enzyme is involved in an NADPH-dependent electron transport pathway. It oxidizes a variety of structurally unrelated compounds, including steroids, fatty acids, retinoid and xenobiotics. Preferentially oxidizes 17beta- estradiol to the carcinogenic 4-hydroxy derivative, and a variety of procarcinogenic compounds to their activated forms, including polycyclic aromatic hydrocarbons. Promotes angiogenesis by removing cellular oxygenation products, thereby decreasing oxidative stres [...]
51	DAAM1	9606.ENSP00000378557	DAAM1	Disheveled-associated activator of morphogenesis 1; Binds to disheveled (Dvl) and Rho, and mediates Wnt- induced Dvl-Rho complex formation. May play a role as a scaffolding protein to recruit Rho-GDP and Rho-GEF, thereby enhancing Rho-GTP formation. Can direct

#	queryItem	stringId	preferredName	annotation
				nucleation and elongation of new actin filaments. Involved in building functional cilia. Involved in the organization of the subapical actin network in multiciliated epithelial cells (By similarity); Belongs to the formin homology family
52	DHRS3	9606.ENSP00000480439	DHRS3	Short-chain dehydrogenase/reductase 3; Catalyzes the reduction of all-trans-retinal to all- trans-retinol in the presence of NADPH; Short chain dehydrogenase/reductase superfamily
53	DIS3L2	9606.ENSP00000315569	DIS3L2	DIS3-like exonuclease 2; 3'-5'-exoribonuclease that specifically recognizes RNAs polyuridylated at their 3' end and mediates their degradation. Component of an exosome-independent RNA degradation pathway that mediates degradation of both mRNAs and miRNAs that have been polyuridylated by a terminal uridylyltransferase, such as ZCCHC11/TUT4. Mediates degradation of cytoplasmic mRNAs that have been deadenylated and subsequently uridylated at their 3'. Mediates degradation of uridylated pre-let-7 miRNAs, contributing to the maintenance of embryonic stem (ES) cells. Essential for correct mi [...]
54	DOCK9	9606.ENSP00000365643	DOCK9	Dedicator of cytokinesis protein 9; Guanine nucleotide-exchange factor (GEF) that activates CDC42 by exchanging bound GDP for free GTP. Overexpression induces filopodia formation; Belongs to the DOCK family
55	DOK6	9606.ENSP00000372160	DOK6	Docking protein 6; DOK proteins are enzymatically inert adaptor or scaffolding proteins. They provide a docking platform for the assembly of multimolecular signaling complexes. DOK6 promotes Ret- mediated neurite growth. May have a role in brain development and/or maintenance
56	DPRX	9606.ENSP00000365838	DPRX	Divergent paired-related homeobox; Putative transcription factor; PRD class homeoboxes and pseudogenes
57	DSCAM	9606.ENSP00000383303	DSCAM	Down syndrome cell adhesion molecule; Cell adhesion molecule that plays a role in neuronal self-avoidance. Promotes repulsion between specific neuronal processes of either the same cell or the same subtype of cells. Mediates within retinal amacrine and ganglion cell subtypes both isoneuronal self-avoidance for creating an orderly dendritic arborization and heteroneuronal self-avoidance to maintain the mosaic spacing between amacrine and ganglion cell bodies. Receptor for netrin required for axon guidance independently of and in collaboration with the receptor DCC. In spinal chord devel [...]
58	DYSF	9606.ENSP00000386881	DYSF	Dysferlin; Key calcium ion sensor involved in the Ca(2+)-triggered synaptic vesicle-plasma membrane fusion. Plays a role in the sarcolemma repair mechanism of both skeletal muscle and cardiomyocytes that permits rapid resealing of membranes disrupted by mechanical stress (By similarity); Ferlin family
59	DZIP1L	9606.ENSP00000332148	DZIP1L	Zinc finger protein DZIP1L; Involved in primary cilium formation. Probably acts as a transition zone protein required for localization of PKD1/PC1 and PKD2/PC2 to the ciliary membrane; Belongs to the DZIP C2H2-type zinc-finger protein family
60	E2F3	9606.ENSP00000262904	E2F3	Transcription factor E2F3; Transcription activator that binds DNA cooperatively with DP proteins through the E2 recognition site, 5'-TTTC[CG]CGC- 3' found in the promoter region of a number of genes whose products are involved in cell cycle regulation or in DNA replication. The DRTF1/E2F complex functions in the control of cell-cycle progression from G1 to S phase. E2F3 binds specifically to RB1 in a cell-cycle dependent manner. Inhibits adipogenesis, probably through the repression of CEBPA binding to its target gene promoters (By similarity)
61	EGR3	9606.ENSP00000318057	EGR3	Early growth response protein 3; Probable transcription factor involved in muscle spindle development; Belongs to the EGR C2H2-type zinc-finger protein family
62	EIF4E	9606.ENSP00000425561	EIF4E	Eukaryotic translation initiation factor 4E
63	ELF2	9606.ENSP00000377782	ELF2	ETS-related transcription factor Elf-2; Isoform 1 transcriptionally activates the LYN and BLK promoters and acts synergistically with RUNX1 to transactivate the BLK promoter; ETS transcription factor family
64	ELF5	9606.ENSP00000311010	ELF5	ETS-related transcription factor Elf-5; Transcriptionally activator that may play a role in regulating the later stages of keratinocytes terminal differentiation; Belongs to the ETS family
65	ELOVL5	9606.ENSP00000359956	ELOVL5	Elongation of very long chain fatty acids protein 5; Catalyzes the first and rate-limiting reaction of the four that constitute the long-chain fatty acids elongation cycle. This endoplasmic reticulum-bound enzymatic process, allows the addition of 2 carbons to the chain of long- and very long-chain fatty acids/VLCFAs per cycle. Condensing enzyme that acts specifically toward polyunsaturated acyl-CoA with the higher activity toward C18:3(n-6) acyl-CoA. May participate in the production of monounsaturated and of polyunsaturated VLCFAs of different chain lengths that are involved in multi [...]
66	ERC2	9606.ENSP00000288221	ERC2	ERC protein 2; Thought to be involved in the organization of the cytomatrix at the nerve terminals active zone (CAZ) which regulates neurotransmitter release. Seems to act together with BSN. May recruit liprin-alpha proteins to the CAZ
67	ERICH1	9606.ENSP00000262109	ERICH1	Glutamate-rich protein 1; Glutamate rich 1
68	EVA1A	9606.ENSP00000233712	EVA1A	Protein eva-1 homolog A; Acts as a regulator of programmed cell death, mediating both autophagy and apoptosis
69	EXOSC5	9606.ENSP00000221233	EXOSC5	Exosome complex component RRP46; Non-catalytic component of the RNA exosome complex which has 3'->5' exoribonuclease activity and participates in a multitude of cellular RNA processing and degradation events. In the nucleus, the RNA exosome complex is involved in proper maturation of stable RNA species such as rRNA, snRNA and snoRNA, in the elimination of RNA processing by-products and non-coding 'pervasive' transcripts, such as antisense RNA species and promoter-upstream transcripts (PROMPTs), and of mRNAs with processing defects, thereby limiting or excluding their export to the cyto [...]
70	F7	9606.ENSP00000364731	F7	Coagulation factor VII; Initiates the extrinsic pathway of blood coagulation. Serine protease that circulates in the blood in a zymogen form. Factor VII is converted to factor VIIa by factor Xa, factor XIIa, factor IXa, or thrombin by minor proteolysis. In the presence of tissue factor and calcium ions, factor VIIa then converts factor X to factor Xa by limited proteolysis. Factor VIIa will also convert factor IX to factor IXa in the presence of tissue factor and calcium; Gla domain containing
71	FAIM	9606.ENSP00000342805	FAIM	Fas apoptotic inhibitory molecule 1; Plays a role as an inducible effector molecule that mediates Fas resistance produced by surface Ig engagement in B cells; Belongs to the FAIM1 family
72	FAM155A	9606.ENSP00000365080	FAM155A	Transmembrane protein FAM155A; Family with sequence similarity 155 member A
73	FAM241B	9606.ENSP00000362376	C10orf35	Uncharacterized protein FAM241B; Chromosome 10 open reading frame 35
74	FAM53A	9606.ENSP00000310057	FAM53A	Protein FAM53A; May play an important role in neural development; the dorsomedial roof of the third ventricle
75	FAM76B	9606.ENSP00000351631	FAM76B	Protein FAM76B; Family with sequence similarity 76 member B; Belongs to the FAM76 family
76	FAM89A	9606.ENSP00000355614	FAM89A	Protein FAM89A; Family with sequence similarity 89 member A; Belongs to the FAM89 family
77	FBXO27	9606.ENSP00000292853	FBXO27	F-box only protein 27; Substrate-recognition component of the SCF (SKP1-CUL1-F- box protein)-type E3 ubiquitin ligase complex. Able to recognize and bind denatured glycoproteins, which are modified with complex- type oligosaccharides; F-boxes other
78	FBXW7	9606.ENSP00000281708	FBXW7	F-box/WD repeat-containing protein 7; Substrate recognition component of a SCF (SKP1-CUL1-F- box protein) E3 ubiquitin-protein ligase complex which mediates the ubiquitination and subsequent proteasomal degradation of target proteins. Recognizes and binds phosphorylated sites/phosphodegrons within target proteins and thereafter bring them to the SCF complex for ubiquitination. Identified substrates include cyclin-E (CCNE1 or CCNE2), JUN, MYC, NOTCH1 released notch intracellular domain (NICD), and probably PSEN1. Acts as a negative regulator of JNK signaling by binding to phosphorylated [...]
79	FCRL6	9606.ENSP00000357086	FCRL6	Fc receptor-like protein 6; Acts as a MHC class II receptor. When stimulated on its own, does not play a role in cytokine production or the release of cytotoxic granules by NK cells and cytotoxic CD8(+) T cells. Does not act as an Fc receptor; Immunoglobulin like domain containing
80	FGD4	9606.ENSP00000394487	FGD4	FYVE, RhoGEF and PH domain-containing protein 4; Activates CDC42, a member of the Ras-like family of Rho- and Rac proteins, by exchanging bound GDP for free GTP. Plays a role in regulating the actin cytoskeleton and cell shape. Activates MAPK8 (By similarity); Pleckstrin homology domain containing
81	FGF13	9606.ENSP00000322390	FGF13	Fibroblast growth factor 13; Microtubule-binding protein which directly binds tubulin and is involved in both polymerization and stabilization of microtubules. Through its action on microtubules, may participate to the refinement of axons by negatively regulating axonal and leading processes branching. Plays a crucial role in neuron polarization and migration in the cerebral cortex and the hippocampus; Belongs to the heparin-binding growth factors family
82	FHDC1	9606.ENSP00000427567	FHDC1	FH2 domain containing 1
83	FNDC3A	9606.ENSP00000441831	FNDC3A	Fibronectin type-III domain-containing protein 3A; Mediates spermatid-Sertoli adhesion during spermatogenesis; Belongs to the FNDC3 family
84	FOXB2	9606.ENSP00000365898	FOXB2	Forkhead box protein B2; Transcription factor; Forkhead boxes
85	FOXC1	9606.ENSP00000370256	FOXC1	Forkhead box protein C1; DNA-binding transcriptional factor that plays a role in a broad range of cellular and developmental processes such as eye, bones, cardiovascular, kidney and skin development. Acts either as a transcriptional activator or repressor. Binds to the consensus binding site 5'-[G/C][A/T]AAA[T/C]AA[A/C]-3' in promoter of target genes. Upon DNA-binding, promotes DNA bending. Acts as a transcriptional coactivator. Stimulates Indian hedgehog (Ihh)- induced target gene expression mediated by the transcription factor GLI2, and hence regulates endochondral ossification (By s [...]
86	FOXF1	9606.ENSP00000262426	FOXF1	Forkhead box protein F1; Probable transcription activator for a number of lung- specific genes; Forkhead boxes
87	FOXP4	9606.ENSP00000362151	FOXP4	Forkhead box protein P4; Transcriptional repressor that represses lung-specific expression; Forkhead boxes
88	FRG1	9606.ENSP00000226798	FRG1	Protein FRG1; Binds to mRNA in a sequence-independent manner. May play a role in regulation of pre-mRNA splicing or in the assembly of rRNA into ribosomal subunits. May be involved in mRNA transport. May be involved in epigenetic regulation of muscle differentiation through regulation of activity of the histone-lysine N- methyltransferase KMT5B; Belongs to the FRG1 family
89	FZD3	9606.ENSP00000437489	FZD3	Frizzled-3; Receptor for Wnt proteins. Most of frizzled receptors are coupled to the beta-catenin canonical signaling pathway, which leads to the activation of disheveled proteins, inhibition of GSK- 3 kinase, nuclear accumulation of beta-catenin and activation of Wnt target genes. A second signaling pathway involving PKC and calcium fluxes has been seen for some family members, but it is not yet clear if it represents a distinct pathway or if it can be integrated in the canonical pathway, as PKC seems to be required for Wnt-mediated inactivation of GSK-3 kinase. Both pathways seem to [...]
90	GABRB3	9606.ENSP00000308725	GABRB3	Gamma-aminobutyric acid receptor subunit beta-3; Component of the heteropentameric receptor for GABA, the major inhibitory neurotransmitter in the vertebrate brain. Functions also as histamine receptor and mediates cellular responses to histamine. Functions as receptor for diazepines and various anesthetics, such as pentobarbital; these are bound at a separate allosteric effector binding site. Functions as ligand-gated chloride channel; Belongs to the ligand-gated ion channel (TC 1.A.9) family. Gamma-aminobutyric acid receptor (TC 1.A.9.5) subfamily. GABRB3 sub-subfamily
91	GAK	9606.ENSP00000314499	GAK	Cyclin-G-associated kinase; Associates with cyclin G and CDK5. Seems to act as an auxilin homolog that is involved in the uncoating of clathrin- coated vesicles by Hsc70 in non-neuronal cells. Expression oscillates slightly during the cell cycle, peaking at G1; Belongs to the protein kinase superfamily. Ser/Thr protein kinase family
92	GAL3ST1	9606.ENSP00000385735	GAL3ST1	Galactosylceramide sulfotransferase; Catalyzes the sulfation of membrane glycolipids. Seems to prefer beta-glycosides at the non-reducing termini of sugar chains attached to a lipid moiety. Catalyzes the synthesis of galactosylceramide sulfate (sulfatide), a major lipid component of the myelin sheath and of monogalactosylalkylacylglycerol sulfate (seminolipid), present in spermatocytes (By similarity). Also acts on lactosylceramide, galactosyl 1-alkyl-2-sn-glycerol and galactosyl diacylglycerol (in vitro); Belongs to the galactose-3-O-sulfotransferase family
93	GALM	9606.ENSP00000272252	GALM	Aldose 1-epimerase; Mutarotase converts alpha-aldose to the beta-anomer. It is active on D-glucose, L-arabinose, D-xylose, D-galactose, maltose and lactose (By similarity)
94	GALR1	9606.ENSP00000299727	GALR1	Galanin receptor type 1; Receptor for the hormone galanin. The activity of this receptor is mediated by G proteins that inhibit adenylate cyclase activity; Belongs to the G-protein coupled receptor 1 family
95	GLCE	9606.ENSP00000261858	GLCE	D-glucuronyl C5-epimerase; Converts D-glucuronic acid residues adjacent to N- sulfate sugar residues to L-iduronic acid residues, both in maturing heparan sulfate (HS) and heparin chains. This is important for further modifications that determine the specificity of interactions between these glycosaminoglycans and proteins; Belongs to the D-glucuronyl C5-epimerase family
96	GMDS	9606.ENSP00000370194	GMDS	GDP-mannose 4,6 dehydratase; Catalyzes the conversion of GDP-D-mannose to GDP-4- dehydro-6-deoxy-D-mannose; Belongs to the NAD(P)-dependent epimerase/dehydratase family. GDP-mannose 4,6-dehydratase subfamily
97	GOLGA4	9606.ENSP00000349305	GOLGA4	Golgin subfamily A member 4; May play a role in delivery of transport vesicles containing GPI-linked proteins from the trans-Golgi network through its interaction with MACF1
98	GPR143	9606.ENSP00000417161	GPR143	G-protein coupled receptor 143; Receptor for tyrosine, L-DOPA and dopamine. After binding to L-DOPA, stimulates Ca(2+) influx into the cytoplasm, increases secretion of the neurotrophic factor SERPINF1 and relocalizes beta arrestin at the plasma membrane; this ligand-dependent signaling occurs through a G(q)-mediated pathway in melanocytic cells. Its activity is mediated by G proteins which activate the phosphoinositide signaling pathway. Plays also a role as an intracellular G protein-coupled receptor involved in melanosome biogenesis, organization and transport; 7TM orphan receptors
99	GPR6	9606.ENSP00000406986	GPR6	G-protein coupled receptor 6; Orphan receptor with constitutive G(s) signaling activity that activate cyclic AMP. Promotes neurite outgrowth and blocks myelin inhibition in neurons (By similarity); G protein-coupled receptors, Class A orphans

PTSD (PSS-I)

#	queryItem	stringId	preferredName	annotation
100	GRB10	9606.ENSP00000381793	GRB10	Growth factor receptor-bound protein 10; Adapter protein which modulates coupling of a number of cell surface receptor kinases with specific signaling pathways. Binds to, and suppress signals from, activated receptors tyrosine kinases, including the insulin (INSR) and insulin-like growth factor (IGF1R) receptors. The inhibitory effect can be achieved by 2 mechanisms: interference with the signaling pathway and increased receptor degradation. Delays and reduces AKT1 phosphorylation in response to insulin stimulation. Blocks association between INSR and IRS1 and IRS2 and prevents insulin [...]
101	GSDMC	9606.ENSP00000276708	GSDMC	Gasdermin-C; The N-terminal moiety promotes pyroptosis. May be acting by homooligomerizing within the membrane and forming pores. The physiological relevance of this observation is unknown (Probable); Gasdermins
102	GTF2IRD1	9606.ENSP00000397566	GTF2IRD1	General transcription factor II-I repeat domain-containing protein 1; May be a transcription regulator involved in cell-cycle progression and skeletal muscle differentiation. May repress GTF2I transcriptional functions, by preventing its nuclear residency, or by inhibiting its transcriptional activation. May contribute to slow-twitch fiber type specificity during myogenesis and in regenerating muscles. Binds troponin I slow-muscle fiber enhancer (USE B1). Binds specifically and with high affinity to the EFG sequences derived from the early enhancer of HOXC8 (By similarity)
103	GTPBP1	9606.ENSP00000216044	GTPBP1	GTP-binding protein 1; Promotes degradation of target mRNA species. Plays a role in the regulation of circadian mRNA stability. Binds GTP and has GTPase activity (By similarity)
104	GYPC	9606.ENSP00000259254	GYPC	Glycophorin-C; This protein is a minor sialoglycoprotein in human erythrocyte membranes. The blood group Gerbich antigens and receptors for Plasmodium falciparum merozoites are most likely located within the extracellular domain. Glycophorin-C plays an important role in regulating the stability of red cells
107	HBS1L	9606.ENSP00000356811	HBS1L	HBS1-like protein; HBS1 like translational GTPase; Belongs to the TRAFAC class translation factor GTPase superfamily. Classic translation factor GTPase family
108	HCN1	9606.ENSP00000307342	HCN1	Potassium/sodium hyperpolarization-activated cyclic nucleotide-gated channel 1; Hyperpolarization-activated ion channel exhibiting weak selectivity for potassium over sodium ions. Contributes to the native pacemaker currents in heart (If) and in neurons (Ih). May mediate responses to sour stimuli
109	HLX	9606.ENSP00000355870	HLX	H2.0-like homeobox protein; Transcription factor required for TBX21/T-bet-dependent maturation of Th1 cells as well as maintenance of Th1-specific gene expression. Involved in embryogenesis and hematopoiesis (By similarity); Belongs to the H2.0 homeobox family
110	HMGN5	9606.ENSP00000350848	HMGN5	High mobility group nucleosome-binding domain-containing protein 5; Preferentially binds to euchromatin and modulates cellular transcription by counteracting linker histone-mediated chromatin compaction; Belongs to the HMGN family
111	HS3ST4	9606.ENSP00000330606	HS3ST4	Heparan sulfate glucosamine 3-O-sulfotransferase 4; Sulfotransferase that utilizes 3'-phospho-5'-adenylyl sulfate (PAPS) to catalyze the transfer of a sulfo group to an N- unsubstituted glucosamine linked to a 2-O-sulfo iduronic acid unit on heparan sulfate. Unlike 3-OST-1, does not convert non- anticoagulant heparan sulfate to anticoagulant heparan sulfate (By similarity)
112	ICA1L	9606.ENSP00000478645	ICA1L	Islet cell autoantigen 1-like protein; Classical BAR domain containing
113	ICAM5	9606.ENSP00000221980	ICAM5	Intercellular adhesion molecule 5; ICAM proteins are ligands for the leukocyte adhesion protein LFA-1 (integrin alpha-L/beta-2); Ig-like cell adhesion molecule family
114	IFNGR1	9606.ENSP00000356713	IFNGR1	Interferon gamma receptor 1; Associates with IFNGR2 to form a receptor for the cytokine interferon gamma (IFNG). Ligand binding stimulates activation of the JAK/STAT signaling pathway; CD molecules
116	IGL	9606.ENSP00000431254	IGLL5	Immunoglobulin lambda like polypeptide 5; C1-set domain containing
117	IGLL1	9606.ENSP00000329312	IGLL1	Immunoglobulin lambda-like polypeptide 1; Critical for B-cell development; C1-set domain containing
118	IL16	9606.ENSP00000302935	IL16	Pro-interleukin-16; Interleukin-16 stimulates a migratory response in CD4+ lymphocytes, monocytes, and eosinophils. Primes CD4+ T-cells for IL-2 and IL-15 responsiveness. Also induces T-lymphocyte expression of interleukin 2 receptor. Ligand for CD4; Interleukins
119	IMMT	9606.ENSP00000387262	IMMT	MICOS complex subunit MIC60; Component of the MICOS complex, a large protein complex of the mitochondrial inner membrane that plays crucial roles in the maintenance of crista junctions, inner membrane architecture, and formation of contact sites to the outer membrane. Plays an important role in the maintenance of the MICOS complex stability and the mitochondrial cristae morphology
120	INPP5A	9606.ENSP00000357583	INPP5A	Type I inositol 1,4,5-trisphosphate 5-phosphatase; Major isoenzyme hydrolyzing the calcium-mobilizing second messenger Ins(1,4,5)P3, this is a signal-terminating reaction; Belongs to the inositol 1,4,5-trisphosphate 5- phosphatase type I family
121	ITGAE	9606.ENSP00000263087	ITGAE	Integrin alpha-E; Integrin alpha-E/beta-7 is a receptor for E-cadherin. It mediates adhesion of intra-epithelial T-lymphocytes to epithelial cell monolayers
122	KCNF1	9606.ENSP00000295082	KCNF1	Potassium voltage-gated channel subfamily F member 1; Putative voltage-gated potassium channel; Belongs to the potassium channel family. F (TC 1.A.1.2) subfamily. Kv5.1/KCNF1 sub-subfamily
123	KIF20B	9606.ENSP00000360793	KIF20B	Kinesin-like protein KIF20B; Plus-end-directed motor enzyme that is required for completion of cytokinesis. Required for proper midbody organization and abscission in polarized cortical stem cells. Plays a role in the regulation of neuronal polarization by mediating the transport of specific cargos. Participates in the mobilization of SHTN1 and in the accumulation of PIP3 in the growth cone of primary hippocampal neurons in a tubulin and actin-dependent manner. In the developing telencephalon, cooperates with SHTN1 to promote both the transition from the multipolar to the bipolar stage [...]
124	KIF26B	9606.ENSP00000385545	KIF26B	Kinesin-like protein KIF26B; Essential for embryonic kidney development. Plays an important role in the compact adhesion between mesenchymal cells adjacent to the ureteric buds, possibly by interacting with MYH10. This could lead to the establishment of the basolateral integrity of the mesenchyme and the polarized expression of ITGA8, which maintains the GDNF expression required for further ureteric bud attraction. Although it seems to lack ATPase activity it is constitutively associated with microtubules (By similarity); Belongs to the TRAFAC class myosin-kinesin ATPase superfamily. K [...]
125	KLF6	9606.ENSP00000419923	KLF6	Krueppel-like factor 6; Transcriptional activator (By similarity). Binds a GC box motif. Could play a role in B-cell growth and development; Belongs to the krueppel C2H2-type zinc-finger protein family
126	L3MBTL3	9606.ENSP00000431962	L3MBTL3	Lethal(3)malignant brain tumor-like protein 3; Putative Polycomb group (PcG) protein. PcG proteins maintain the transcriptionally repressive state of genes, probably via a modification of chromatin, rendering it heritably changed in its expressibility. Required for normal maturation of myeloid progenitor cells (By similarity); MBT domain containing
127	LECT2	9606.ENSP00000274507	LECT2	Leukocyte cell-derived chemotaxin-2; Has a neutrophil chemotactic activity. Also a positive regulator of chondrocyte proliferation. Does not show metalloendopeptidase activity; Belongs to the LECT2/MIM-1 family
128	LGI1	9606.ENSP00000360472	LGI1	Leucine-rich glioma-inactivated protein 1; Regulates voltage-gated potassium channels assembled from KCNA1, KCNA4 and KCNAB1. It slows down channel inactivation by precluding channel closure mediated by the KCNAB1 subunit. Ligand for ADAM22 that positively regulates synaptic transmission mediated by AMPA-type glutamate receptors (By similarity). Plays a role in suppressing the production of MMP1/3 through the phosphatidylinositol 3-kinase/ERK pathway. May play a role in the control of neuroblastoma cell survival
129	LHX5	9606.ENSP00000261731	LHX5	LIM/homeobox protein Lhx5; Plays an essential role in the regulation of neuronal differentiation and migration during development of the central nervous system; LIM class homeoboxes
130	LINGO2	9606.ENSP00000369328	LINGO2	Leucine-rich repeat and immunoglobulin-like domain-containing nogo receptor-interacting protein 2; Leucine rich repeat and Ig domain containing 2
131	LMBRD1	9606.ENSP00000359609	LMBRD1	Probable lysosomal cobalamin transporter; Probable lysosomal cobalamin transporter. Required to export cobalamin from lysosomes allowing its conversion to cofactors. Isoform 3 may play a role in the assembly of hepatitis delta virus (HDV)
132	LMOD1	9606.ENSP00000356257	LMOD1	Leiomodin-1; Mediates nucleation of actin filaments; Belongs to the tropomodulin family
133	LRRC14B	9606.ENSP00000327675	LRRC14B	Leucine-rich repeat-containing protein 14B; Leucine rich repeat containing 14B; Belongs to the PRAME family. LRRC14 subfamily
134	LSM3	9606.ENSP00000302160	LSM3	U6 snRNA-associated Sm-like protein LSm3; Binds specifically to the 3'-terminal U-tract of U6 snRNA; Belongs to the snRNP Sm proteins family
135	LTBP3	9606.ENSP00000301873	LTBP3	Latent-transforming growth factor beta-binding protein 3; May be involved in the assembly, secretion and targeting of TGFB1 to sites at which it is stored and/or activated. May play critical roles in controlling and directing the activity of TGFB1. May have a structural role in the extracellular matrix (ECM); Belongs to the LTBP family
136	LYZ	9606.ENSP00000261267	LYZ	Lysozyme C; Lysozymes have primarily a bacteriolytic function; those in tissues and body fluids are associated with the monocyte- macrophage system and enhance the activity of immunoagents; Lysozymes, c-type
137	MACROH2A1	9606.ENSP00000423563	H2AFY	Core histone macro-H2A.1; Variant histone H2A which replaces conventional H2A in a subset of nucleosomes where it represses transcription. Nucleosomes wrap and compact DNA into chromatin, limiting DNA accessibility to the cellular machineries which require DNA as a template. Histones thereby play a central role in transcription regulation, DNA repair, DNA replication and chromosomal stability. DNA accessibility is regulated via a complex set of post-translational modifications of histones, also called histone code, and nucleosome remodeling. Involved in stable X chromosome inactivation [...]
138	MAPK10	9606.ENSP00000352157	MAPK10	Mitogen-activated protein kinase 10; Serine/threonine-protein kinase involved in various processes such as neuronal proliferation, differentiation, migration and programmed cell death. Extracellular stimuli such as proinflammatory cytokines or physical stress stimulate the stress- activated protein kinase/c-Jun N-terminal kinase (SAP/JNK) signaling pathway. In this cascade, two dual specificity kinases MAP2K4/MKK4 and MAP2K7/MKK7 phosphorylate and activate MAPK10/JNK3. In turn, MAPK10/JNK3 phosphorylates a number of transcription factors, primarily components of AP-1 such as JUN and AT [...]
139	MCM3	9606.ENSP00000480987	MCM3	DNA replication licensing factor MCM3; Acts as component of the MCM2-7 complex (MCM complex) which is the putative replicative helicase essential for 'once per cell cycle' DNA replication initiation and elongation in eukaryotic cells. The active ATPase sites in the MCM2-7 ring are formed through the interaction surfaces of two neighboring subunits such that a critical structure of a conserved arginine finger motif is provided in trans relative to the ATP-binding site of the Walker A box of the adjacent subunit. The six ATPase active sites, however, are likely to contribute differential [...]
140	ME3	9606.ENSP00000440246	ME3	NADP-dependent malic enzyme, mitochondrial; Malic enzyme 3; Belongs to the malic enzymes family
141	MED13L	9606.ENSP00000281928	MED13L	Mediator of RNA polymerase II transcription subunit 13-like; Component of the Mediator complex, a coactivator involved in the regulated transcription of nearly all RNA polymerase II-dependent genes. Mediator functions as a bridge to convey information from gene-specific regulatory proteins to the basal RNA polymerase II transcription machinery. Mediator is recruited to promoters by direct interactions with regulatory proteins and serves as a scaffold for the assembly of a functional preinitiation complex with RNA polymerase II and the general transcription factors. This subunit may spe [...]
142	MFSD14A	9606.ENSP00000359171	HIAT1	Hippocampus abundant transcript 1
143	MGAT5B	9606.ENSP00000391227	MGAT5B	Alpha-1,6-mannosylglycoprotein 6-beta-N-acetylglucosaminyltransferase B; Glycosyltransferase that acts on alpha-linked mannose of N-glycans and O-mannosyl glycans. Catalyzes the transfer of N- acetylglucosamine (GlcNAc) to the beta 1-6 linkage of the mannose residue of GlcNAcbeta1,2-Manalpha on both the alpha1,3- and alpha1,6-linked mannose arms in the core structure of N-glycan. Also acts on the GlcNAcbeta1,2-Manalpha1-Ser/Thr moiety, forming a 2,6-branched structure in brain O-mannosyl glycan. Plays an active role in modulating integrin and laminin-dependent adhesion and migration of [...]
144	MGRN1	9606.ENSP00000262370	MGRN1	E3 ubiquitin-protein ligase MGRN1; E3 ubiquitin-protein ligase. Mediates monoubiquitination at multiple sites of TSG101 in the presence of UBE2D1, but not of UBE2G1, nor UBE2H. Plays a role in the regulation of endosome-to- lysosome trafficking. Impairs MC1R- and MC4R-signaling by competing with GNAS-binding to MCRs and inhibiting agonist-induced cAMP production. Does not inhibit ADRB2-signaling. Does not promote MC1R ubiquitination; Ring finger proteins
145	MICALL2	9606.ENSP00000297508	MICALL2	MICAL-like protein 2; Effector of small Rab GTPases which is involved in junctional complexes assembly through the regulation of cell adhesion molecules transport to the plasma membrane and actin cytoskeleton reorganization. Regulates the endocytic recycling of occludins, claudins and E-cadherin to the plasma membrane and may thereby regulate the establishment of tight junctions and adherens junctions. In parallel, may regulate actin cytoskeleton reorganization directly through interaction with F-actin or indirectly through actinins and filamins. Most probably involved in the processes [...]
146	MID1	9606.ENSP00000312678	MID1	E3 ubiquitin-protein ligase Midline-1; Has E3 ubiquitin ligase activity towards IGBP1, promoting its monoubiquitination, which results in deprotection of the catalytic subunit of protein phosphatase PP2A, and its subsequent degradation by polyubiquitination; Belongs to the TRIM/RBCC family
147	MIER2	9606.ENSP00000264819	MIER2	Mesoderm induction early response protein 2; Transcriptional repressor; Myb/SANT domain containing
148	MLN	9606.ENSP00000388825	MLN	Promotilin; Plays an important role in the regulation of interdigestive gastrointestinal motility and indirectly causes rhythmic contraction of duodenal and colonic smooth muscle; Endogenous ligands

PTSD (PSS-I)

#	queryItem	stringId	preferredName	annotation
149	MOGAT3	9606.ENSP00000223114	MOGAT3	2-acylglycerol O-acyltransferase 3; Catalyzes the formation of diacylglycerol from 2- monoacylglycerol and fatty acyl-CoA. Also able to catalyze the terminal step in triacylglycerol synthesis by using diacylglycerol and fatty acyl-CoA as substrates. Has a preference toward palmitoyl-CoA and oleoyl-CoA. May be involved in absorption of dietary fat in the small intestine by catalyzing the resynthesis of triacylglycerol in enterocytes
150	MPDU1	9606.ENSP00000250124	MPDU1	Mannose-P-dolichol utilization defect 1 protein; Required for normal utilization of mannose-dolichol phosphate (Dol-P-Man) in the synthesis of N-linked and O-linked oligosaccharides and GPI anchors; Belongs to the MPDU1 (TC 2.A.43.3) family
151	MPP7	9606.ENSP00000337907	MPP7	MAGUK p55 subfamily member 7; Acts as an important adapter that promotes epithelial cell polarity and tight junction formation via its interaction with DLG1. Involved in the assembly of protein complexes at sites of cell-cell contact; Belongs to the MAGUK family
152	MRPS5	9606.ENSP00000272418	MRPS5	Mitochondrial ribosomal protein S5
153	MRPS7	9606.ENSP00000245539	MRPS7	Mitochondrial ribosomal protein S7; Belongs to the universal ribosomal protein uS7 family
154	MSX2	9606.ENSP00000239243	MSX2	Homeobox protein MSX-2; Acts as a transcriptional regulator in bone development. Represses the ALPL promoter activity and antogonizes the stimulatory effect of DLX5 on ALPL expression during osteoblast differentiation. Probable morphogenetic role. May play a role in limb-pattern formation. In osteoblasts, suppresses transcription driven by the osteocalcin FGF response element (OCFRE). Binds to the homeodomain-response element of the ALPL promoter; NKL subclass homeoboxes and pseudogenes
155	MTCL1	9606.ENSP00000352927	MTCL1	Microtubule cross-linking factor 1; Microtubule-associated factor involved in the late phase of epithelial polarization and microtubule dynamics regulation. Plays a role in the development and maintenance of non-centrosomal microtubule bundles at the lateral membrane in polarized epithelial cells; Belongs to the SOGA family
156	MTUS1	9606.ENSP00000262102	MTUS1	Microtubule-associated tumor suppressor 1; Cooperates with AGTR2 to inhibit ERK2 activation and cell proliferation. May be required for AGTR2 cell surface expression. Together with PTPN6, induces UBE2V2 expression upon angiotensin-II stimulation. Isoform 1 inhibits breast cancer cell proliferation, delays the progression of mitosis by prolonging metaphase and reduces tumor growth; Belongs to the MTUS1 family
157	MTX1	9606.ENSP00000357360	MTX1	Metaxin-1; Involved in transport of proteins into the mitochondrion. Essential for embryonic development (By similarity)
158	MYCBPAP	9606.ENSP00000323184	MYCBPAP	MYCBP-associated protein; May play a role in spermatogenesis. May be involved in synaptic processes (By similarity)
159	NBPF12	9606.ENSP00000478609	NBPF12	Neuroblastoma breakpoint family member 1; NBPF member 12
160	NCL	9606.ENSP00000318195	NCL	Nucleolin; Nucleolin is the major nucleolar protein of growing eukaryotic cells. It is found associated with intranucleolar chromatin and pre-ribosomal particles. It induces chromatin decondensation by binding to histone H1. It is thought to play a role in pre-rRNA transcription and ribosome assembly. May play a role in the process of transcriptional elongation. Binds RNA oligonucleotides with 5'-UUAGGG-3' repeats more tightly than the telomeric single-stranded DNA 5'-TTAGGG-3' repeats
161	NCOR2	9606.ENSP00000384018	NCOR2	Nuclear receptor corepressor 2; Transcriptional corepressor. Mediates the transcriptional repression activity of some nuclear receptors by promoting chromatin condensation, thus preventing access of the basal transcription. Isoform 1 and isoform 5 have different affinities for different nuclear receptors. Involved in the regulation BCL6-dependent of the germinal center (GC) reactions, mainly through the control of the GC B-cells proliferation and survival; Myb/SANT domain containing
162	NDRG1	9606.ENSP00000404854	NDRG1	Protein NDRG1; Stress-responsive protein involved in hormone responses, cell growth, and differentiation. Acts as a tumor suppressor in many cell types. Necessary but not sufficient for p53/TP53- mediated caspase activation and apoptosis. Has a role in cell trafficking, notably of the Schwann cell, and is necessary for the maintenance and development of the peripheral nerve myelin sheath. Required for vesicular recycling of CDH1 and TF. May also function in lipid trafficking. Protects cells from spindle disruption damage. Functions in p53/TP53-dependent mitotic spindle checkpoint. Regu [...]
163	NDST2	9606.ENSP00000310657	NDST2	Bifunctional heparan sulfate N-deacetylase/N-sulfotransferase 2; Essential bifunctional enzyme that catalyzes both the N- deacetylation and the N-sulfation of glucosamine (GlcNAc) of the glycosaminoglycan in heparan sulfate. Modifies the GlcNAc-GlcA disaccharide repeating sugar backbone to make N-sulfated heparosan, a prerequisite substrate for later modifications in heparin biosynthesis. Plays a role in determining the extent and pattern of sulfation of heparan sulfate. Required for the exosomal release of SDCBP, CD63 and syndecan; Sulfotransferases, membrane bound
164	NDUFAF8	9606.ENSP00000400184	C17orf89	NADH dehydrogenase [ubiquinone] 1 alpha subcomplex assembly factor 8; Involved in the assembly of mitochondrial NADH:ubiquinone oxidoreductase complex (complex I, MT-ND1). Required to stabilize NDUFAF5
165	NDUFB8	9606.ENSP00000299166	NDUFB8	NADH dehydrogenase [ubiquinone] 1 beta subcomplex subunit 8, mitochondrial; Accessory subunit of the mitochondrial membrane respiratory chain NADH dehydrogenase (Complex I), that is believed not to be involved in catalysis. Complex I functions in the transfer of electrons from NADH to the respiratory chain. The immediate electron acceptor for the enzyme is believed to be ubiquinone
166	NDUFC1	9606.ENSP00000441126	NDUFC1	NADH dehydrogenase [ubiquinone] 1 subunit C1, mitochondrial; Accessory subunit of the mitochondrial membrane respiratory chain NADH dehydrogenase (Complex I), that is believed not to be involved in catalysis. Complex I functions in the transfer of electrons from NADH to the respiratory chain. The immediate electron acceptor for the enzyme is believed to be ubiquinone; NADH:ubiquinone oxidoreductase supernumerary subunits
167	NEDD4	9606.ENSP00000424827	NEDD4	E3 ubiquitin-protein ligase NEDD4; E3 ubiquitin-protein ligase which accepts ubiquitin from an E2 ubiquitin-conjugating enzyme in the form of a thioester and then directly transfers the ubiquitin to targeted substrates. Specifically ubiquitinates 'Lys-63' in target proteins. Involved in the pathway leading to the degradation of VEGFR-2/KDFR, independently of its ubiquitin-ligase activity. Monoubiquitinates IGF1R at multiple sites, thus leading to receptor internalization and degradation in lysosomes. Ubiquitinates FGFR1, leading to receptor internalization and degradation in lysosomes. [...]
168	NENF	9606.ENSP00000355955	NENF	Neudesin; Acts as a neurotrophic factor in postnatal mature neurons enhancing neuronal survival. Promotes cell proliferation and neurogenesis in undifferentiated neural pro-genitor cells at the embryonic stage and inhibits differentiation of astrocyte. Its neurotrophic activity is exerted via MAPK1/ERK2, MAPK3/ERK1 and AKT1/AKT pathways. Neurotrophic activity is enhanced by binding to heme. Acts also as an anorexigenic neurotrophic factor that contributes to energy balance (By similarity). Plays a role in the human tumorigenesis; Belongs to the cytochrome b5 family. MAPR subfamily
169	NFIB	9606.ENSP00000370340	NFIB	Nuclear factor 1 B-type; Recognizes and binds the palindromic sequence 5'- TTGGCNNNNNGCCAA-3' present in viral and cellular promoters and in the origin of replication of adenovirus type 2. These proteins are individually capable of activating transcription and replication; Belongs to the CTF/NF-I family
170	NHS	9606.ENSP00000369400	NHS	Nance-Horan syndrome protein; May function in cell morphology by maintaining the integrity of the circumferential actin ring and controlling lamellipod formation. Involved in the regulation eye, tooth, brain and craniofacial development; Belongs to the NHS family
171	NHSL2	9606.ENSP00000424079	NHSL2	NHS-like protein 2; NHS like 2
172	NID1	9606.ENSP00000264187	NID1	Nidogen-1; Sulfated glycoprotein widely distributed in basement membranes and tightly associated with laminin. Also binds to collagen IV and perlecan. It probably has a role in cell- extracellular matrix interactions
173	NKAIN1	9606.ENSP00000362841	NKAIN1	Sodium/potassium-transporting ATPase subunit beta-1-interacting protein 1; Sodium/potassium transporting ATPase interacting 1
174	NOX5	9606.ENSP00000373518	NOX5	NADPH oxidase 5; Calcium-dependent NADPH oxidase that generates superoxide. Also functions as a calcium-dependent proton channel and may regulate redox-dependent processes in lymphocytes and spermatozoa. May play a role in cell growth and apoptosis. Isoform v2 and isoform v5 are involved in endothelial generation of reactive oxygen species (ROS), proliferation and angiogenesis and contribute to endothelial response to thrombin; EF-hand domain containing
175	NPFFR2	9606.ENSP00000307822	NPFFR2	Neuropeptide FF receptor 2; Receptor for NPAF (A-18-F-amide) and NPFF (F-8-F-amide) neuropeptides, also known as morphine-modulating peptides. Can also be activated by a variety of naturally occurring or synthetic FMRF-amide like ligands. This receptor mediates its action by association with G proteins that activate a phosphatidylinositol- calcium second messenger system
176	NR2F2	9606.ENSP00000377721	NR2F2	COUP transcription factor 2; Ligand-activated transcription factor. Activated by high concentrations of 9-cis-retinoic acid and all-trans-retinoic acid, but not by dexamethasone, cortisol or progesterone (in vitro). Regulation of the apolipoprotein A-I gene transcription. Binds to DNA site A; Nuclear hormone receptors
177	OCA2	9606.ENSP00000346659	OCA2	P protein; Could be involved in the transport of tyrosine, the precursor to melanin synthesis, within the melanocyte. Regulates the pH of melanosome and the melanosome maturation. One of the components of the mammalian pigmentary system. Seems to regulate the post-translational processing of tyrosinase, which catalyzes the limiting reaction in melanin synthesis. May serve as a key control point at which ethnic skin color variation is determined. Major determinant of brown and/or blue eye color; Belongs to the CitM (TC 2.A.11) transporter family
178	OLIG2	9606.ENSP00000331040	OLIG2	Oligodendrocyte transcription factor 2; Required for oligodendrocyte and motor neuron specification in the spinal cord, as well as for the development of somatic motor neurons in the hindbrain. Cooperates with OLIG1 to establish the pMN domain of the embryonic neural tube. Antagonist of V2 interneuron and of NKX2-2-induced V3 interneuron development (By similarity); Basic helix-loop-helix proteins
179	OOSP2	9606.ENSP00000278855	OOSP2	Oocyte secreted protein 2
180	OSBPL9	9606.ENSP00000412733	OSBPL9	Oxysterol-binding protein-related protein 9; Pleckstrin homology domain containing; Belongs to the OSBP family
181	P2RY1	9606.ENSP00000304767	P2RY1	P2Y purinoceptor 1; Receptor for extracellular adenine nucleotides such as ATP and ADP. In platelets binding to ADP leads to mobilization of intracellular calcium ions via activation of phospholipase C, a change in platelet shape, and probably to platelet aggregation; Belongs to the G-protein coupled receptor 1 family
182	PAK1	9606.ENSP00000278568	PAK1	Serine/threonine-protein kinase PAK 1; Protein kinase involved in intracellular signaling pathways downstream of integrins and receptor-type kinases that plays an important role in cytoskeleton dynamics, in cell adhesion, migration, proliferation, apoptosis, mitosis, and in vesicle-mediated transport processes. Can directly phosphorylate BAD and protects cells against apoptosis. Activated by interaction with CDC42 and RAC1. Functions as GTPase effector that links the Rho-related GTPases CDC42 and RAC1 to the JNK MAP kinase pathway. Phosphorylates and activates MAP2K1, and thereby media [...]
183	PAQR4	9606.ENSP00000321804	PAQR4	Progestin and adipoQ receptor family member 4
184	PCDH7	9606.ENSP00000441802	PCDH7	Protocadherin-7; Protein phosphatase 1 regulatory subunits; Non-clustered protocadherins
185	PDGFA	9606.ENSP00000346508	PDGFA	Platelet-derived growth factor subunit A; Growth factor that plays an essential role in the regulation of embryonic development, cell proliferation, cell migration, survival and chemotaxis. Potent mitogen for cells of mesenchymal origin. Required for normal lung alveolar septum formation during embryogenesis, normal development of the gastrointestinal tract, normal development of Leydig cells and spermatogenesis. Required for normal oligodendrocyte development and normal myelination in the spinal cord and cerebellum. Plays an important role in wound healing. Signaling is modulated by t [...]
186	PDZD8	9606.ENSP00000334642	PDZD8	PDZ domain-containing protein 8; Molecular tethering protein that connects endoplasmic reticulum and mitochondria membranes. PDZD8-dependent endoplasmic reticulum-mitochondria membrane tethering is essential for endoplasmic reticulum-mitochondria Ca(2+) transfer. In neurons, involved in the regulation of dendritic Ca(2+) dynamics by regulating mitochondrial Ca(2+) uptake in neurons. Plays an indirect role in the regulation of cell morphology and cytoskeletal organization. May inhibit herpes simplex virus 1 infection at an early stage; PDZ domain containing
187	PDZRN3	9606.ENSP00000263666	PDZRN3	E3 ubiquitin-protein ligase PDZRN3; E3 ubiquitin-protein ligase. Plays an important role in regulating the surface level of MUSK on myotubes. Mediates the ubiquitination of MUSK, promoting its endocytosis and lysosomal degradation. Might contribute to terminal myogenic differentiation; PDZ domain containing
188	PITPNA	9606.ENSP00000316809	PITPNA	Phosphatidylinositol transfer protein alpha isoform; Catalyzes the transfer of PtdIns and phosphatidylcholine between membranes; Phosphatidylinositol transfer proteins
189	PLAC1	9606.ENSP00000352173	PLAC1	Placenta-specific protein 1; May play a role in placental development; Belongs to the PLAC1 family
190	PLEKHG4B	9606.ENSP00000283426	PLEKHG4B	Pleckstrin homology and RhoGEF domain containing G4B
191	PLPP3	9606.ENSP00000360296	PPAP2B	Phospholipid phosphatase 3; Catalyzes the conversion of phosphatidic acid (PA) to diacylglycerol (DG). In addition it hydrolyzes lysophosphatidic acid (LPA), ceramide-1-phosphate (C-1-P) and sphingosine-1- phosphate (S-1-P). The relative catalytic efficiency is LPA = PA > C-1-P > S-1-P. May be involved in cell adhesion and in cell-cell interactions
192	POLR1E	9606.ENSP00000367029	POLR1E	DNA-directed RNA polymerase I subunit RPA49; DNA-dependent RNA polymerase catalyzes the transcription of DNA into RNA using the four ribonucleoside triphosphates as substrates. Component of RNA polymerase I which synthesizes ribosomal RNA precursors. Appears to be involved in the formation of the initiation complex at the promoter by mediating the interaction between Pol I and UBTF/UBF (By similarity); Belongs to the eukaryotic RPA49/POLR1E RNA polymerase subunit family
193	PPP1R32	9606.ENSP00000344140	PPP1R32	Protein phosphatase 1 regulatory subunit 32
194	PPP1R9A	9606.ENSP00000405514	PPP1R9A	Neurabin-1; Binds to actin filaments (F-actin) and shows cross- linking activity. Binds along the sides of the F-actin. May be involved in neurite formation. Inhibits protein phosphatase 1- alpha activity (By similarity); PDZ domain containing

PTSD (PSS-I)

#	queryItem	stringId	preferredName	annotation
195	PPP2R2C	9606.ENSP00000335083	PPP2R2C	Serine/threonine-protein phosphatase 2A 55 kDa regulatory subunit B gamma isoform; The B regulatory subunit might modulate substrate selectivity and catalytic activity, and also might direct the localization of the catalytic enzyme to a particular subcellular compartment
196	PSTPIP1	9606.ENSP00000452746	PSTPIP1	Proline-serine-threonine phosphatase-interacting protein 1; Involved in regulation of the actin cytoskeleton. May regulate WAS actin-bundling activity. Bridges the interaction between ABL1 and PTPN18 leading to ABL1 dephosphorylation. May play a role as a scaffold protein between PTPN12 and WAS and allow PTPN12 to dephosphorylate WAS. Has the potential to physically couple CD2 and CD2AP to WAS. Acts downstream of CD2 and CD2AP to recruit WAS to the T-cell:APC contact site so as to promote the actin polymerization required for synapse induction during T-cell activation (By similarity). [...]
197	PTPN23	9606.ENSP00000265562	PTPN23	Tyrosine-protein phosphatase non-receptor type 23; Plays a role in sorting of endocytic ubiquitinated cargos into multivesicular bodies (MVBs) via its interaction with the ESCRT-I complex (endosomal sorting complex required for transport I), and possibly also other ESCRT complexes. May act as a negative regulator of Ras-mediated mitogenic activity. Plays a role in ciliogenesis; Protein tyrosine phosphatases, non-receptor type
198	PTPN3	9606.ENSP00000363667	PTPN3	Tyrosine-protein phosphatase non-receptor type 3; May act at junctions between the membrane and the cytoskeleton. Possesses tyrosine phosphatase activity; FERM domain containing
199	PTPRN2	9606.ENSP00000374069	PTPRN2	Receptor-type tyrosine-protein phosphatase N2; Plays a role in vesicle-mediated secretory processes. Required for normal accumulation of secretory vesicles in hippocampus, pituitary and pancreatic islets. Required for the accumulation of normal levels of insulin-containing vesicles and preventing their degradation. Plays a role in insulin secretion in response to glucose stimuli. Required for normal accumulation of the neurotransmitters norepinephrine, dopamine and serotonin in the brain. In females, but not in males, required for normal accumulation and secretion of pituitary hormones [...]
200	RAB11FIP1	9606.ENSP00000331342	RAB11FIP1	Rab11 family-interacting protein 1; A Rab11 effector protein involved in the endosomal recycling process. Also involved in controlling membrane trafficking along the phagocytic pathway and in phagocytosis; C2 domain containing
201	RAB6C	9606.ENSP00000387307	RAB6C	Ras-related protein Rab-6C; May be involved in the regulation of centrosome duplication and cell cycle progression; RAB, member RAS oncogene GTPases
202	RAI14	9606.ENSP00000427123	RAI14	Ankycorbin; Plays a role in actin regulation at the ectoplasmic specialization, a type of cell junction specific to testis. Important for establishment of sperm polarity and normal spermatid adhesion. May also promote integrity of Sertoli cell tight junctions at the blood-testis barrier; Ankyrin repeat domain containing
203	RASGRF1	9606.ENSP00000405963	RASGRF1	Ras-specific guanine nucleotide-releasing factor 1; Promotes the exchange of Ras-bound GDP by GTP; Pleckstrin homology domain containing
204	RET	9606.ENSP00000347942	RET	Proto-oncogene tyrosine-protein kinase receptor Ret; Receptor tyrosine-protein kinase involved in numerous cellular mechanisms including cell proliferation, neuronal navigation, cell migration, and cell differentiation upon binding with glial cell derived neurotrophic factor family ligands. Phosphorylates PTK2/FAK1. Regulates both cell death/survival balance and positional information. Required for the molecular mechanisms orchestration during intestine organogenesis; involved in the development of enteric nervous system and renal organogenesis during embryonic life, and promotes the f [...]
205	RGS12	9606.ENSP00000339381	RGS12	Regulator of G-protein signaling 12; Regulates G protein-coupled receptor signaling cascades. Inhibits signal transduction by increasing the GTPase activity of G protein alpha subunits, thereby driving them into their inactive GDP-bound form; PDZ domain containing
206	RINL	9606.ENSP00000467107	RINL	Ras and Rab interactor-like protein; Guanine nucleotide exchange factor (GEF) for RAB5A and RAB22A that activates RAB5A and RAB22A by exchanging bound GDP for free GTP. Plays a role in endocytosis via its role in activating Rab family members (By similarity); VPS9 domain containing
207	RNASE10	9606.ENSP00000333358	RNASE10	Inactive ribonuclease-like protein 10; Secreted proximal epididymal protein required for post- testicular sperm maturation and male fertility. May be involved in sperm adhesion to the egg zona pellucida. Does not have ribonuclease activity (By similarity)
208	RND3	9606.ENSP00000364886	RND3	Rho-related GTP-binding protein RhoE; Binds GTP but lacks intrinsic GTPase activity and is resistant to Rho-specific GTPase-activating proteins; Rho family GTPases
209	RNF115	9606.ENSP00000463650	RNF115	E3 ubiquitin-protein ligase RNF115; E3 ubiquitin-protein ligase that mediates E2-dependent, 'Lys-48'- and/or 'Lys-63'-linked polyubiquitination of substrates and may play a role in diverse biological processes. Through their polyubiquitination, may play a role in the endosomal trafficking and degradation of membrane receptors including EGFR, FLT3, MET and CXCR4; Ring finger proteins
210	RP9	9606.ENSP00000297157	RP9	Retinitis pigmentosa 9 protein; Is thought to be a target protein for the PIM1 kinase. May play some roles in B-cell proliferation in association with PIM1 (By similarity)
211	RPS6KA2	9606.ENSP00000427015	RPS6KA2	Ribosomal protein S6 kinase alpha-2; Serine/threonine-protein kinase that acts downstream of ERK (MAPK1/ERK2 and MAPK3/ERK1) signaling and mediates mitogenic and stress-induced activation of transcription factors, regulates translation, and mediates cellular proliferation, survival, and differentiation. May function as tumor suppressor in epithelial ovarian cancer cells; Mitogen-activated protein kinase-activated protein kinases
212	SATB1	9606.ENSP00000399518	SATB1	DNA-binding protein SATB1; Crucial silencing factor contributing to the initiation of X inactivation mediated by Xist RNA that occurs during embryogenesis and in lymphoma (By similarity). Binds to DNA at special AT-rich sequences, the consensus SATB1-binding sequence (CSBS), at nuclear matrix- or scaffold-associated regions. Thought to recognize the sugar-phosphate structure of double-stranded DNA. Transcriptional repressor controlling nuclear and viral gene expression in a phosphorylated and acetylated status-dependent manner, by binding to matrix attachment regions (MARs) of DNA and [...]
213	SCPEP1	9606.ENSP00000262288	SCPEP1	Retinoid-inducible serine carboxypeptidase; May be involved in vascular wall and kidney homeostasis; M14 carboxypeptidases
214	SDHAF3	9606.ENSP00000414066	ACN9	Succinate dehydrogenase assembly factor 3, mitochondrial; Plays an essential role in the assembly of succinate dehydrogenase (SDH), an enzyme complex (also referred to as respiratory complex II) that is a component of both the tricarboxylic acid (TCA) cycle and the mitochondrial electron transport chain, and which couples the oxidation of succinate to fumarate with the reduction of ubiquinone (coenzyme Q) to ubiquinol. Promotes maturation of the iron-sulfur protein subunit SDHB of the SDH catalytic dimer, protecting it from the deleterious effects of oxidants. May act together with SDHAF1
215	SDK1	9606.ENSP00000385899	SDK1	Protein sidekick-1; Adhesion molecule that promotes lamina-specific synaptic connections in the retina. Expressed in specific subsets of interneurons and retinal ganglion cells (RGCs) and promotes synaptic connectivity via homophilic interactions; Fibronectin type III domain containing
216	SEMA6B	9606.ENSP00000467290	SEMA6B	Semaphorin-6B; May play a role in both peripheral and central nervous system development; Semaphorins
217	SFRP5	9606.ENSP00000266066	SFRP5	Secreted frizzled-related protein 5; Soluble frizzled-related proteins (sFRPS) function as modulators of Wnt signaling through direct interaction with Wnts. They have a role in regulating cell growth and differentiation in specific cell types. SFRP5 may be involved in determining the polarity of photoreceptor, and perhaps, other cells in the retina
218	SH3PXD2A	9606.ENSP00000348215	SH3PXD2A	SH3 and PX domain-containing protein 2A; Adapter protein involved in invadopodia and podosome formation, extracellular matrix degradation and invasiveness of some cancer cells. Binds matrix metalloproteinases (ADAMs), NADPH oxidases (NOXs) and phosphoinositides. Acts as an organizer protein that allows NOX1- or NOX3-dependent reactive oxygen species (ROS) generation and ROS localization. In association with ADAM12, mediates the neurotoxic effect of amyloid-beta peptide
219	SHANK2	9606.ENSP00000469689	SHANK2	SH3 and multiple ankyrin repeat domains protein 2; Seems to be an adapter protein in the postsynaptic density (PSD) of excitatory synapses that interconnects receptors of the postsynaptic membrane including NMDA-type and metabotropic glutamate receptors, and the actin-based cytoskeleton. May play a role in the structural and functional organization of the dendritic spine and synaptic junction; Belongs to the SHANK family
220	SKA1	9606.ENSP00000285116	SKA1	Spindle and kinetochore-associated protein 1; Component of the SKA1 complex, a microtubule-binding subcomplex of the outer kinetochore that is essential for proper chromosome segregation. Required for timely anaphase onset during mitosis, when chromosomes undergo bipolar attachment on spindle microtubules leading to silencing of the spindle checkpoint. The SKA1 complex is a direct component of the kinetochore-microtubule interface and directly associates with microtubules as oligomeric assemblies. The complex facilitates the processive movement of microspheres along a microtubule in a [...]
221	SLC16A3	9606.ENSP00000463978	SLC16A3	Monocarboxylate transporter 4; Proton-linked monocarboxylate transporter. Catalyzes the rapid transport across the plasma membrane of many monocarboxylates such as lactate, pyruvate, branched-chain oxo acids derived from leucine, valine and isoleucine, and the ketone bodies acetoacetate, beta-hydroxybutyrate and acetate (By similarity); Belongs to the major facilitator superfamily. Monocarboxylate porter (TC 2.A.1.13) family
222	SLC16A9	9606.ENSP00000378757	SLC16A9	Monocarboxylate transporter 9; Proton-linked monocarboxylate transporter. May catalyze the transport of monocarboxylates across the plasma membrane; Solute carriers
223	SLC30A2	9606.ENSP00000363394	SLC30A2	Solute carrier family 30 member 2
224	SLC35A3	9606.ENSP00000359172	SLC35A3	UDP-N-acetylglucosamine transporter; Uridine diphosphate-N-acetylglucosamine (UDP-GlcNAc) transporter in the Golgi apparatus. May supply UDP-GlcNAc as substrate for Golgi-resident glycosyltransferases that generate branching of diantennary oligosaccharides; Belongs to the nucleotide-sugar transporter family. SLC35A subfamily
225	SLC5A9	9606.ENSP00000236495	SLC5A9	Sodium/glucose cotransporter 4; Involved in sodium-dependent transport of D-mannose, D- glucose and D-fructose; Belongs to the sodium:solute symporter (SSF) (TC 2.A.21) family
226	SLC6A20	9606.ENSP00000346298	SLC6A20	Sodium- and chloride-dependent transporter XTRP3; Mediates the calcium-dependent uptake of imino acids such as L-proline, N-methyl-L-proline and pipecolate as well as N- methylated amino acids. Involved in the transport of glycine; Solute carriers
227	SLIT2	9606.ENSP00000422591	SLIT2	Slit homolog 2 protein; Thought to act as molecular guidance cue in cellular migration, and function appears to be mediated by interaction with roundabout homolog receptors. During neural development involved in axonal navigation at the ventral midline of the neural tube and projection of axons to different regions. SLIT1 and SLIT2 seem to be essential for midline guidance in the forebrain by acting as repulsive signal preventing inappropriate midline crossing by axons projecting from the olfactory bulb. In spinal chord development may play a role in guiding commissural axons once they [...]
228	SND1	9606.ENSP00000346762	SND1	Staphylococcal nuclease domain-containing protein 1; Functions as a bridging factor between STAT6 and the basal transcription factor. Plays a role in PIM1 regulation of MYB activity. Functions as a transcriptional coactivator for the Epstein-Barr virus nuclear antigen 2 (EBNA2); Tudor domain containing
229	SNX7	9606.ENSP00000304429	SNX7	Sorting nexin-7; May be involved in several stages of intracellular trafficking; Belongs to the sorting nexin family
230	SOWAHC	9606.ENSP00000365830	SOWAHC	Ankyrin repeat domain-containing protein SOWAHC; Sosondowah ankyrin repeat domain family member C; Belongs to the SOWAH family
231	SOX13	9606.ENSP00000356172	SOX13	Transcription factor SOX-13; Binds to the sequence 5'-AACAAT-3'; SRY-boxes
232	SOX21	9606.ENSP00000366144	SOX21	Transcription factor SOX-21; May play a role as an activator of transcription of OPRM1; SRY-boxes
233	SOX9	9606.ENSP00000245479	SOX9	Transcription factor SOX-9; Transcriptional regulator. Binds to the COL2A1 promoter and activates COL2A1 expression, as part of a complex with ZNF219 (By similarity). Plays a role in chondrocyte differentiation (By similarity). Important for normal skeletal development; SRY-boxes
234	SP5	9606.ENSP00000364430	SP5	Transcription factor Sp5; Binds to GC boxes promoters elements. Probable transcriptional activator that has a role in the coordination of changes in transcription required to generate pattern in the developing embryo (By similarity); Sp transcription factors
235	SPACA1	9606.ENSP00000237201	SPACA1	Sperm acrosome membrane-associated protein 1; May be involved in sperm-egg fusion
236	SPNS3	9606.ENSP00000347721	SPNS3	Protein spinster homolog 3; Sphingolipid transporter
237	SSU72	9606.ENSP00000291386	SSU72	RNA polymerase II subunit A C-terminal domain phosphatase SSU72; Protein phosphatase that catalyzes the dephosphorylation of the C-terminal domain of RNA polymerase II. Plays a role in RNA processing and termination. Plays a role in pre-mRNA polyadenylation via its interaction with SYMPK; Belongs to the SSU72 phosphatase family
238	STAMBPL1	9606.ENSP00000360994	STAMBPL1	AMSH-like protease; Zinc metalloprotease that specifically cleaves 'Lys-63'- linked polyubiquitin chains. Does not cleave 'Lys-48'-linked polyubiquitin chains; Belongs to the peptidase M67C family
239	STARD3NL	9606.ENSP00000009041	STARD3NL	STARD3 N-terminal-like protein; Tethering protein that creates contact site between the endoplasmic reticulum and late endosomes: localizes to late endosome membranes and contacts the endoplasmic reticulum via interaction with VAPA and VAPB; Belongs to the STARD3 family
240	STC2	9606.ENSP00000265087	STC2	Stanniocalcin-2; Has an anti-hypocalcemic action on calcium and phosphate homeostasis; Belongs to the stanniocalcin family
241	STK3	9606.ENSP00000429744	STK3	Serine/threonine-protein kinase 3; Stress-activated, pro-apoptotic kinase which, following caspase-cleavage, enters the nucleus and induces chromatin condensation followed by internucleosomal DNA fragmentation. Key component of the Hippo signaling pathway which plays a pivotal role in organ size control and tumor suppression by restricting proliferation and promoting apoptosis. The core of this pathway is composed of a kinase cascade wherein STK3/MST2 and STK4/MST1, in complex with its regulatory protein SAV1, phosphorylates and activates LATS1/2 in complex with its regulatory protein [...]

#	queryItem	stringId	preferredName	annotation
242	STK33	9606.ENSP00000416750	STK33	Serine/threonine-protein kinase 33; Serine/threonine protein kinase which phosphorylates VIME. May play a specific role in the dynamic behavior of the intermediate filament cytoskeleton by phosphorylation of VIME (By similarity). Not essential for the survival of KRAS-dependent AML cell lines
243	STON2	9606.ENSP00000450857	STON2	Stonin-2; Adapter protein involved in endocytic machinery. Involved in the synaptic vesicle recycling. May facilitate clathrin-coated vesicle uncoating; Belongs to the Stoned B family
244	STXBP6	9606.ENSP00000324302	STXBP6	Syntaxin-binding protein 6; Forms non-fusogenic complexes with SNAP25 and STX1A and may thereby modulate the formation of functional SNARE complexes and exocytosis
245	SUGCT	9606.ENSP00000338475	SUGCT	Succinate--hydroxymethylglutarate CoA-transferase; Catalyzes the succinyl-CoA-dependent conversion of glutarate to glutaryl-CoA. Can use different dicarboxylic acids as CoA acceptors, the preferred ones are glutarate, succinate, adipate, and 3-hydroxymethylglutarate; Belongs to the CaiB/BaiF CoA-transferase family
246	SULT6B1	9606.ENSP00000384950	SULT6B1	Sulfotransferase 6B1; Sulfotransferase that utilizes 3'-phospho-5'-adenylyl sulfate (PAPS) as sulfonate donor to catalyze the sulfate conjugation of thyroxine. Involved in the metabolism of thyroxine (By similarity); Sulfotransferases, cytosolic
247	SUSD1	9606.ENSP00000363382	SUSD1	Sushi domain containing 1
248	SV2B	9606.ENSP00000377779	SV2B	Synaptic vesicle glycoprotein 2B; Probably plays a role in the control of regulated secretion in neural and endocrine cells
249	SYNE3	9606.ENSP00000334308	SYNE3	Nesprin-3; As a component of the LINC (LInker of Nucleoskeleton and Cytoskeleton) complex involved in the connection between the nuclear lamina and the cytoskeleton. The nucleocytoplasmic interactions established by the LINC complex play an important role in the transmission of mechanical forces across the nuclear envelope and in nuclear movement and positioning. Probable anchoring protein which tethers the nucleus to the cytoskeleton by binding PLEC which can associate with the intermediate filament system. Plays a role in the regulation of aortic epithelial cell morphology, and is re [...]
250	SYS1	9606.ENSP00000243918	SYS1	Protein SYS1 homolog; Involved in protein trafficking. May serve as a receptor for ARFRP1; Belongs to the SYS1 family
251	TAC4	9606.ENSP00000334042	TAC4	Tachykinin-4; Tachykinins are active peptides which excite neurons, evoke behavioral responses, are potent vasodilators and secretagogues, and contract (directly or indirectly) many smooth muscles. Endokinin-A induces thermal hyperalgesia and pain-related behavior such as scratching following intrathecal administration in rats. These effects are suppressed by treatment with endokinin- C. Endokinin-A/B reduces arterial blood pressure and increases sperm motility
252	TAFA5	9606.ENSP00000383933	FAM19A5	Protein FAM19A5; Family with sequence similarity 19 member A5, C-C motif chemokine like; Belongs to the FAM19/TAFA family
253	TATDN3	9606.ENSP00000431376	TATDN3	Putative deoxyribonuclease TATDN3; Putative deoxyribonuclease; Belongs to the metallo-dependent hydrolases superfamily. TatD-type hydrolase family
254	TCERG1L	9606.ENSP00000357631	TCERG1L	Transcription elongation regulator 1 like
255	TCP11	9606.ENSP00000308708	TCP11	T-complex protein 11 homolog; Plays a role in the process of sperm capacitation and acrosome reactions. Probable receptor for the putative fertilization-promoting peptide (FPP) at the sperm membrane that may modulate the activity of the adenylyl cyclase cAMP pathway; Belongs to the TCP11 family
256	TENM4	9606.ENSP00000278550	TENM4	Teneurin-4; Involved in neural development, regulating the establishment of proper connectivity within the nervous system. Plays a role in the establishment of the anterior-posterior axis during gastrulation. Regulates the differentiation and cellular process formation of oligodendrocytes and myelination of small- diameter axons in the central nervous system (CNS). Promotes activation of focal adhesion kinase. May function as a cellular signal transducer (By similarity); Belongs to the tenascin family. Teneurin subfamily
257	TEX101	9606.ENSP00000472308	TEX101	Testis-expressed protein 101; Plays a role in fertilization by controlling binding of sperm to zona pellucida and migration of spermatozoa into the oviduct (By similarity). May play a role in signal transduction and promote protein tyrosine phosphorylation (By similarity); LY6/PLAUR domain containing
258	THBS2	9606.ENSP00000355751	THBS2	Thrombospondin-2; Adhesive glycoprotein that mediates cell-to-cell and cell-to-matrix interactions. Ligand for CD36 mediating antiangiogenic properties
259	THSD4	9606.ENSP00000347484	THSD4	Thrombospondin type-1 domain-containing protein 4; Promotes FBN1 matrix assembly. Attenuates TGFB signaling, possibly by accelerating the sequestration of large latent complexes of TGFB or active TGFB by FBN1 microfibril assembly, thereby negatively regulating the expression of TGFB regulatory targets, such as POSTN (By similarity); ADAMTS like
260	TLR3	9606.ENSP00000296795	TLR3	Toll-like receptor 3; Key component of innate and adaptive immunity. TLRs (Toll-like receptors) control host immune response against pathogens through recognition of molecular patterns specific to microorganisms. TLR3 is a nucleotide-sensing TLR which is activated by double-stranded RNA, a sign of viral infection. Acts via the adapter TRIF/TICAM1, leading to NF-kappa-B activation, IRF3 nuclear translocation, cytokine secretion and the inflammatory response; CD molecules
261	TMEM220	9606.ENSP00000339830	TMEM220	Transmembrane protein 220
263	TNKS2	9606.ENSP00000360689	TNKS2	Tankyrase-2; Poly-ADP-ribosyltransferase involved in various processes such as Wnt signaling pathway, telomere length and vesicle trafficking. Acts as an activator of the Wnt signaling pathway by mediating poly-ADP-ribosylation of AXIN1 and AXIN2, 2 key components of the beta-catenin destruction complex: poly-ADP- ribosylated target proteins are recognized by RNF146, which mediates their ubiquitination and subsequent degradation. Also mediates poly-ADP-ribosylation of BLZF1 and CASC3, followed by recruitment of RNF146 and subsequent ubiquitination. Mediates poly-ADP-ribosylation of TER [...]
264	TRAPPC12	9606.ENSP00000324318	TRAPPC12	Trafficking protein particle complex subunit 12; Plays an important role in chromosome congression, kinetochore assembly and stability and controls the recruitment of CENPE to the kinetochores. May be involved in endoplasmic reticulum to Golgi apparatus trafficking at a very early stage; Tetratricopeptide repeat domain containing
265	TRIP13	9606.ENSP00000166345	TRIP13	Pachytene checkpoint protein 2 homolog; Plays a key role in chromosome recombination and chromosome structure development during meiosis. Required at early steps in meiotic recombination that leads to non-crossovers pathways. Also needed for efficient completion of homologous synapsis by influencing crossover distribution along the chromosomes affecting both crossovers and non-crossovers pathways. Also required for development of higher-order chromosome structures and is needed for synaptonemal-complex formation. In males, required for efficient synapsis of the sex chromosomes and for [...]
266	TSHZ2	9606.ENSP00000360552	TSHZ2	Teashirt homolog 2; Probable transcriptional regulator involved in developmental processes. May act as a transcriptional repressor (Potential); ZF class homeoboxes and pseudogenes
267	TSPYL6	9606.ENSP00000417919	TSPYL6	Testis-specific Y-encoded-like protein 6; TSPY like 6
268	UBASH3B	9606.ENSP00000284273	UBASH3B	Ubiquitin-associated and SH3 domain-containing protein B; Interferes with CBL-mediated down-regulation and degradation of receptor-type tyrosine kinases. Promotes accumulation of activated target receptors, such as T-cell receptors and EGFR, on the cell surface. Exhibits tyrosine phosphatase activity toward several substrates including EGFR, FAK, SYK, and ZAP70. Down-regulates proteins that are dually modified by both protein tyrosine phosphorylation and ubiquitination
269	UHRF1BP1	9606.ENSP00000192788	UHRF1BP1	UHRF1-binding protein 1; May act as a negative regulator of cell growth
270	USP12	9606.ENSP00000282344	USP12	Ubiquitin carboxyl-terminal hydrolase 12; Deubiquitinating enzyme. Has almost no deubiquitinating activity by itself and requires the interaction with WDR20 and WDR48 to have a high activity. Not involved in deubiquitination of monoubiquitinated FANCD2. In complex with WDR48, acts as a potential tumor suppressor by positively regulating PHLPP1 stability; Belongs to the peptidase C19 family. USP12/USP46 subfamily
271	VTI1A	9606.ENSP00000376792	VTI1A	Vesicle transport through interaction with t-SNAREs homolog 1A; V-SNARE that mediates vesicle transport pathways through interactions with t-SNAREs on the target membrane. These interactions are proposed to mediate aspects of the specificity of vesicle trafficking and to promote fusion of the lipid bilayers. Involved in vesicular transport from the late endosomes to the trans-Golgi network. Along with VAMP7, involved in an non- conventional RAB1-dependent traffic route to the cell surface used by KCNIP1 and KCND2. May be involved in increased cytokine secretion associated with cellular [...]
272	WDR27	9606.ENSP00000416289	WDR27	WD repeat-containing protein 27; WD repeat domain containing
273	WDR45B	9606.ENSP00000376139	WDR45B	WD repeat domain containing
274	WDR75	9606.ENSP00000314193	WDR75	WD repeat-containing protein 75; Ribosome biogenesis factor. Involved in nucleolar processing of pre-18S ribosomal RNA. Required for optimal pre- ribosomal RNA transcription by RNA polymerase I; UTPa subcomplex
275	WWP2	9606.ENSP00000352069	WWP2	NEDD4-like E3 ubiquitin-protein ligase WWP2; E3 ubiquitin-protein ligase which accepts ubiquitin from an E2 ubiquitin-conjugating enzyme in the form of a thioester and then directly transfers the ubiquitin to targeted substrates. Polyubiquitinates POU5F1 by 'Lys-63'-linked conjugation and promotes it to proteasomal degradation; in embryonic stem cells (ESCs) the ubiquitination is proposed to regulate POU5F1 protein level. Ubiquitinates EGR2 and promotes it to proteasomal degradation; in T-cells the ubiquitination inhibits activation- induced cell death. Ubiquitinates SLC11A2; the ubiqu [...]
276	XKR3	9606.ENSP00000331704	XKR3	XK-related protein 3; XK related family; Belongs to the XK family
277	ZBBX	9606.ENSP00000390232	ZBBX	Zinc finger B-box domain containing
278	ZCCHC13	9606.ENSP00000345633	ZCCHC13	Zinc finger CCHC-type containing 13
279	ZDHHC19	9606.ENSP00000296326	ZDHHC19	Probable palmitoyltransferase ZDHHC19; Zinc finger DHHC-type containing 19; Belongs to the DHHC palmitoyltransferase family
280	ZEB1	9606.ENSP00000354487	ZEB1	Zinc finger E-box-binding homeobox 1; Acts as a transcriptional repressor. Inhibits interleukin-2 (IL-2) gene expression. Enhances or represses the promoter activity of the ATP1A1 gene depending on the quantity of cDNA and on the cell type. Represses E-cadherin promoter and induces an epithelial-mesenchymal transition (EMT) by recruiting SMARCA4/BRG1. Represses BCL6 transcription in the presence of the corepressor CTBP1. Positively regulates neuronal differentiation. Represses RCOR1 transcription activation during neurogenesis. Represses transcription by binding to the E box (5'-CANNTG [...]
281	ZFHX4	9606.ENSP00000430497	ZFHX4	Zinc finger homeobox protein 4; May play a role in neural and muscle differentiation (By similarity). May be involved in transcriptional regulation; ZF class homeoboxes and pseudogenes
282	ZFP3	9606.ENSP00000320347	ZFP3	Zinc finger protein 3 homolog; May be involved in transcriptional regulation; Zinc fingers C2H2-type
283	ZG16B	9606.ENSP00000371715	ZG16B	Zymogen granule protein 16B; Belongs to the jacalin lectin family
284	ZMYND8	9606.ENSP00000420095	ZMYND8	Protein kinase C-binding protein 1; May act as a transcriptional corepressor for KDM5D. Required for KDM5D-mediated down-regulation of diverse metastasis- associated genes; the function seems to involve the recognition of the dual histone signature H3K4me1-H3K14ac. Suppresses prostate cancer cell invasion; Bromodomain containing
285	ZNF185	9606.ENSP00000440847	ZNF185	Zinc finger protein 185; May be involved in the regulation of cellular proliferation and/or differentiation; LIM domain containing
286	ZNF221	9606.ENSP00000251269	ZNF221	Zinc finger protein 221; May be involved in transcriptional regulation; Zinc fingers C2H2-type
287	ZNF449	9606.ENSP00000339585	ZNF449	Zinc finger protein 449; May be involved in transcriptional regulation; Belongs to the krueppel C2H2-type zinc-finger protein family
288	ZNF496	9606.ENSP00000294753	ZNF496	Zinc finger protein 496; DNA-binding transcription factor that can both act as an activator and a repressor; Belongs to the krueppel C2H2-type zinc-finger protein family
289	ZNF627	9606.ENSP00000354414	ZNF627	Zinc finger protein 627; May be involved in transcriptional regulation; Zinc fingers C2H2-type
290	ZNF648	9606.ENSP00000344129	ZNF648	Zinc finger protein 648; May be involved in transcriptional regulation; Zinc fingers C2H2-type
291	ZNF766	9606.ENSP00000409652	ZNF766	Zinc finger protein 766; May be involved in transcriptional regulation; Zinc fingers C2H2-type
292	ZNF782	9606.ENSP00000419397	ZNF782	Zinc finger protein 782; May be involved in transcriptional regulation; Zinc fingers C2H2-type
293	ZNRF2	9606.ENSP00000323879	ZNRF2	E3 ubiquitin-protein ligase ZNRF2; May play a role in the establishment and maintenance of neuronal transmission and plasticity via its ubiquitin ligase activity. E3 ubiquitin ligases accept ubiquitin from an E2 ubiquitin-conjugating enzyme in the form of a thioester and then directly transfer the ubiquitin to targeted substrates; Ring finger proteins

PTSD (PSS-I)

Association with Depression

Gene list

ABI1, ABTB2, ACSBG1, ADAP2, ADGRG4, AGBL4, AKAP7, ALCAM, ALDH7A1, ALDH9A1, AMOT, ANKRD11, ANKRD44, ANKRD6, ANTXR1, ANTXR2, AP1G1, AP1M2, ARFGEF3, ARL4D, ASPG, B3GAT1, BHLHA9, BICC1, BIK, BPNT2, C11orf80, C14orf180, C1orf53, C1orf87, C20orf203, C21orf91, C2CD4D, C8orf34, CACNA2D1, CACNG5, CBLN2, CCDC158, CCDC85A, CCNYL2, CD300LG, CD99L2, CDH17, CEACAM21, CELF4, CENPF, CFHR4, CHRDL1, CHRDL2, CHRNA9, CLEC3A, CLSTN1, CNKSR3, CNTN5, CNTROB, COL12A1, COX7C, CPXM2, CSRP1, CSTF3, CUEDC1, CXCL8, CYP39A1, DCTN1, DIABLO, DNA2, DNAI3, DPH1, DSC3, DST, DUSP2, DYNC2I1, EAF1, ECE1, EGFR, EHMT1, ELF5, EPHA4, EPSTI1, ERBB4, ERCC6L2, ERICH1, ESYT3, EXOSC5, EYA1, FADD, FAM207A, FAM53B, FAM53C, FAM83D, FAT4, FGD4, FGF12, FNDC3A, FOSB, FOXF1, FOXN3, FRK, FTL, FXYD3, FYB2, FZD3, GALNT9, GGA3, GPM6A, GPR39, GRID1, GRIP2, H2BC13, HACD3, HBQ1, HDDC2, HTR1D, IFFO2, IGF2, IGFALS, IGSF11, IL13RA1, INPP4A, IQGAP2, KBTBD11, KCNIP1, KCNMB2, KDM4A, KIAA2013, KIR3DL2, KLK4, KMT5B, LAMB4, LARP1, LEP, LINC00163, LINGO2, LMOD1, LNPK, LRATD1, LRCH1, LRP5L, LRRC15, LRRC28, MAMSTR, MAP4K1, MAPK8, MARK4, MDGA2, ME3, METRNL, MGP, MGRN1, MLN, MROH5, MTCL1, MTX2, NBPF1, NFE2L3, NFIX, NHS, NKAIN1, NKX2-5, NMI, NOD1, NOL4L, NONO, NOX4, NPFFR2, NR4A2, NXN, OLIG3, OOSP2, OR4X1, OSBP2, OSTM1, PAK1, PAQR4, PARP15, PCDH9, PCDHB12, PCDHB3, PDZD8, PGK1, PIMREG, PISD, PKDCC, PLCB3, PLEKHM3, PLEKHO2, POLL, PPDPFL, PPFIA1, PPP2R3A, PPP2R5A, PRKCQ, PRRT1, PTCD2, QSER1, RAB3C, RAB6A, RABGEF1, RAD18, RAD50, RAP2A, RASGRF1, RCC1, REEP3, RESF1, RHOH, RND3, RNMT, RPRM, RTL8A, RUNX1T1, SCTR, SDK2, SEMA6A, SERINC5, SERPINC1, SFR, SH3RF1, SHANK2, SIM2, SLC15A4, SLC16A12, SLC26A10, SLC39A14, SLC49A4, SLC52A1, SLIT1, SNRPN, SNX1, SOX2, SOX9, SPNS2, SPTBN4, SRBD1, STARD3NL, STK19, SULT6B1, SYBU, SYNE3, SYS1, TAF4, TBC1D31, TEX13C, TFAP2A, TFAP2E, TGFBI, TIAM2, TIE1, TLL1, TMEM120B, TMEM52B, TMIGD3, TMSB4X, TOM1L1, TOMM5, TP63, TPTE2, TRERF1, TRIM42, TRIM46, TRIM54, TRPV4, TSC1, TSPYL6, TTLL11, TTLL7, USP31, VAV2, VWA5B1, WAS, WDR45, WDR59, WFIKKN2, WSCD1, WWP2, XAGE5, XKR3, YPEL2, ZMYND8, ZNF233, ZNF331, ZNF423, ZNF449, ZNF503, ZNF536, ZNF710, ZNF77

Network

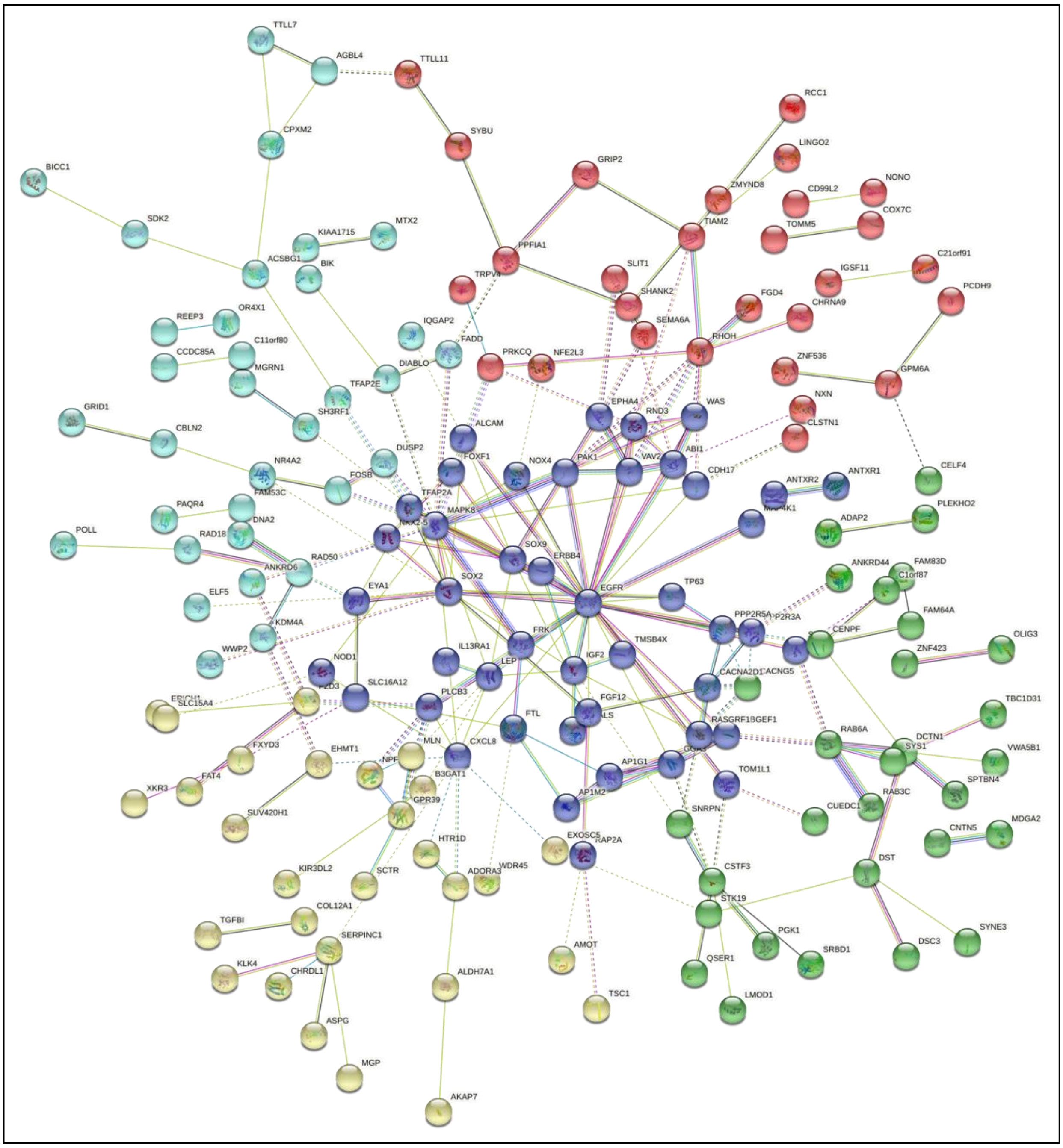

Depression (PHQ-9)

Enrichments found

Network Stats

number of nodes:	274	expected number of edges:	200
number of edges:	231	PPI enrichment p-value:	0.0186
average node degree:	1.69		
avg. local clustering coefficient:	0.367		

your network has significantly more interactions than expected (what does that mean?)

Enrichment Table Columns

Count In Network:
The first number indicates how many proteins in your network are annotated with a particular term. The second number indicates how many proteins in total (in your network and in the background) have this term assigned.

Strength:
Log10(observed / expected). This measure describes how large the enrichment effect is. It's the ratio between i) the number of proteins in your network that are annotated with a term and ii) the number of proteins that we expect to be annotated with this term in a random network of the same size.

False Discovery Rate:
This measure describes how significant the enrichment is. Shown are p-values corrected for multiple testing within each category using the Benjamini–Hochberg procedure.

Functional enrichments in your network

explain columns

Biological Process (Gene Ontology)

GO-term	description	count in network	strength	false discovery rate
GO:1905475	regulation of protein localization to membrane	10 of 163	0.64	0.0481
GO:0001501	skeletal system development	20 of 457	0.49	0.0197
GO:0022610	biological adhesion	29 of 849	0.39	0.0197
GO:0007155	cell adhesion	28 of 843	0.38	0.0197
GO:0009790	embryo development	28 of 890	0.35	0.0293
GO:0009653	anatomical structure morphogenesis	49 of 1992	0.24	0.0294
GO:0048731	system development	91 of 4144	0.2	0.0119
GO:0030154	cell differentiation	76 of 3457	0.2	0.0197
GO:0048869	cellular developmental process	77 of 3533	0.19	0.0197
GO:0007275	multicellular organism development	97 of 4726	0.17	0.0197
GO:0048856	anatomical structure development	104 of 5085	0.16	0.0197
GO:0032502	developmental process	109 of 5401	0.16	0.0197

(less ...)

Cellular Component (Gene Ontology)

GO-term	description	count in network	strength	false discovery rate
GO:0030175	filopodium	9 of 96	0.83	0.0034
GO:0030027	lamellipodium	13 of 185	0.7	0.0018
GO:0031252	cell leading edge	15 of 371	0.46	0.0494

Annotated Keywords (UniProt)

keyword	description	count in network	strength	false discovery rate
KW-0130	Cell adhesion	18 of 476	0.43	0.0256
KW-0025	Alternative splicing	193 of 10225	0.13	2.32e-07

Depression (PHQ-9)

Mapping

#	queryItem	stringId	preferredName	annotation
1	ABI1	9606.ENSP00000365312	ABI1	Abl interactor 1; May act in negative regulation of cell growth and transformation by interacting with nonreceptor tyrosine kinases ABL1 and/or ABL2. May play a role in regulation of EGF-induced Erk pathway activation. Involved in cytoskeletal reorganization and EGFR signaling. Together with EPS8 participates in transduction of signals from Ras to Rac. In vitro, a trimeric complex of ABI1, EPS8 and SOS1 exhibits Rac specific guanine nucleotide exchange factor (GEF) activity and ABI1 seems to act as an adapter in the complex. Regulates ABL1/c-Abl-mediated phosphorylation of ENAH. Recrui [...]
2	ABTB2	9606.ENSP00000410157	ABTB2	Ankyrin repeat and BTB/POZ domain-containing protein 2; May be involved in the initiation of hepatocyte growth; Ankyrin repeat domain containing
3	ACSBG1	9606.ENSP00000258873	ACSBG1	Long-chain-fatty-acid--CoA ligase ACSBG1; Mediates activation of long-chain fatty acids for both synthesis of cellular lipids, and degradation via beta-oxidation. Able to activate long-chain fatty acids. Also able to activate very long-chain fatty acids; however, the relevance of such activity is unclear in vivo. Can activate diverse saturated, monosaturated and polyunsaturated fatty acids; Belongs to the ATP-dependent AMP-binding enzyme family. Bubblegum subfamily
4	ADAP2	9606.ENSP00000329468	ADAP2	Arf-GAP with dual PH domain-containing protein 2; GTPase-activating protein for the ADP ribosylation factor family (Potential). Binds phosphatidylinositol 3,4,5-trisphosphate (PtdInsP3) and inositol 1,3,4,5-tetrakisphosphate (InsP4). Possesses a stoichiometry of two binding sites for InsP4 with identical affinity; ArfGAPs
5	ADGRG4	9606.ENSP00000377699	GPR112	Adhesion G-protein coupled receptor G4; Orphan receptor; Belongs to the G-protein coupled receptor 2 family. Adhesion G-protein coupled receptor (ADGR) subfamily
6	AGBL4	9606.ENSP00000360905	AGBL4	Cytosolic carboxypeptidase 6; Metallocarboxypeptidase that mediates deglutamylation of target proteins. Catalyzes the deglutamylation of polyglutamate side chains generated by post-translational polyglutamylation in proteins such as tubulins. Also removes polyglutamates from the carboxy-terminus of target proteins such as MYLK. Mediates deglutamylation of CGAS/MB21D1, regulating the antiviral activity of CGAS/MB21D1. Acts as a long-chain deglutamylase and specifically shortens long polyglutamate chains, while it is not able to remove the branching point glutamate, a process catalyzed b [...]
7	AKAP7	9606.ENSP00000405252	AKAP7	A-kinase anchor protein 7 isoform gamma; Probably targets cAMP-dependent protein kinase (PKA) to the cellular membrane or cytoskeletal structures. The membrane-associated form reduces epithelial sodium channel (ENaC) activity, whereas the free cytoplasmic form may negatively regulate ENaC channel feedback inhibition by intracellular sodium; A-kinase anchoring proteins
8	ALCAM	9606.ENSP00000305988	ALCAM	CD166 antigen; Cell adhesion molecule that mediates both heterotypic cell-cell contacts via its interaction with CD6, as well as homotypic cell-cell contacts. Promotes T-cell activation and proliferation via its interactions with CD6. Contributes to the formation and maturation of the immunological synapse via its interactions with CD6. Mediates homotypic interactions with cells that express ALCAM. Required for normal hematopoietic stem cell engraftment in the bone marrow. Mediates attachment of dendritic cells onto endothelial cells via homotypic interaction. Inhibits endothelial cell [...]
9	ALDH7A1	9606.ENSP00000387123	ALDH7A1	Alpha-aminoadipic semialdehyde dehydrogenase; Multifunctional enzyme mediating important protective effects. Metabolizes betaine aldehyde to betaine, an important cellular osmolyte and methyl donor. Protects cells from oxidative stress by metabolizing a number of lipid peroxidation-derived aldehydes. Involved in lysine catabolism
10	ALDH9A1	9606.ENSP00000346827	ALDH9A1	4-trimethylaminobutyraldehyde dehydrogenase; Converts gamma-trimethylaminobutyraldehyde into gamma- butyrobetaine. Catalyzes the irreversible oxidation of a broad range of aldehydes to the corresponding acids in an NAD-dependent reaction
11	AMOT	9606.ENSP00000361027	AMOT	Angiomotin; Plays a central role in tight junction maintenance via the complex formed with ARHGAP17, which acts by regulating the uptake of polarity proteins at tight junctions. Appears to regulate endothelial cell migration and tube formation. May also play a role in the assembly of endothelial cell-cell junctions; Belongs to the angiomotin family
12	ANKRD11	9606.ENSP00000301030	ANKRD11	Ankyrin repeat domain-containing protein 11; Chromatin regulator which modulates histone acetylation and gene expression in neural precursor cells (By similarity). May recruit histone deacetylases (HDACs) to the p160 coactivators/nuclear receptor complex to inhibit ligand-dependent transactivation. Has a role in proliferation and development of cortical neural precursors. May also regulate bone homeostasis (By similarity); Ankyrin repeat domain containing
13	ANKRD44	9606.ENSP00000282272	ANKRD44	Serine/threonine-protein phosphatase 6 regulatory ankyrin repeat subunit B; Putative regulatory subunit of protein phosphatase 6 (PP6) that may be involved in the recognition of phosphoprotein substrates; Ankyrin repeat domain containing
14	ANKRD6	9606.ENSP00000430985	ANKRD6	Ankyrin repeat domain-containing protein 6; Recruits CKI-epsilon to the beta-catenin degradation complex that consists of AXN1 or AXN2 and GSK3-beta and allows efficient phosphorylation of beta-catenin, thereby inhibiting beta-catenin/Tcf signals; Ankyrin repeat domain containing
15	ANTXR1	9606.ENSP00000301945	ANTXR1	Anthrax toxin receptor 1; Plays a role in cell attachment and migration. Interacts with extracellular matrix proteins and with the actin cytoskeleton. Mediates adhesion of cells to type 1 collagen and gelatin, reorganization of the actin cytoskeleton and promotes cell spreading. Plays a role in the angiogenic response of cultured umbilical vein endothelial cells
16	ANTXR2	9606.ENSP00000306185	ANTXR2	Anthrax toxin receptor 2; Necessary for cellular interactions with laminin and the extracellular matrix
17	AP1G1	9606.ENSP00000377148	AP1G1	AP-1 complex subunit gamma-1; Subunit of clathrin-associated adaptor protein complex 1 that plays a role in protein sorting in the late-Golgi/trans-Golgi network (TGN) and/or endosomes. The AP complexes mediate both the recruitment of clathrin to membranes and the recognition of sorting signals within the cytosolic tails of transmembrane cargo molecules
18	AP1M2	9606.ENSP00000250244	AP1M2	AP-1 complex subunit mu-2; Subunit of clathrin-associated adaptor protein complex 1 that plays a role in protein sorting in the trans-Golgi network (TGN) and endosomes. The AP complexes mediate the recruitment of clathrin to membranes and the recognition of sorting signals within the cytosolic tails of transmembrane cargo molecules
19	ARFGEF3	9606.ENSP00000251691	KIAA1244	Brefeldin A-inhibited guanine nucleotide-exchange protein 3; Participates in the regulation of systemic glucose homeostasis, where it negatively regulates insulin granule biogenesis in pancreatic islet beta cells (By similarity). Also regulates glucagon granule production in pancreatic alpha cells (By similarity). Inhibits nuclear translocation of the transcriptional coregulator PHB2 and may enhance estrogen receptor alpha (ESR1) transcriptional activity in breast cancer cells
20	ARL4D	9606.ENSP00000322628	ARL4D	ADP-ribosylation factor-like protein 4D; Small GTP-binding protein which cycles between an inactive GDP-bound and an active GTP-bound form, and the rate of cycling is regulated by guanine nucleotide exchange factors (GEF) and GTPase-activating proteins (GAP). GTP-binding protein that does not act as an allosteric activator of the cholera toxin catalytic subunit. Recruits CYTH1, CYTH2, CYTH3 and CYTH4 to the plasma membrane in GDP-bound form
21	ASPG	9606.ENSP00000450040	ASPG	60 kDa lysophospholipase; Exhibits lysophospholipase, transacylase, PAF acetylhydrolase and asparaginase activities; In the N-terminal section; belongs to the asparaginase 1 family
22	B3GAT1	9606.ENSP00000433847	B3GAT1	Galactosylgalactosylxylosylprotein 3-beta-glucuronosyltransferase 1; Involved in the biosynthesis of L2/HNK-1 carbohydrate epitope on glycoproteins. Can also play a role in glycosaminoglycan biosynthesis. Substrates include asialo- orosomucoid (ASOR), asialo-fetuin, and asialo-neural cell adhesion molecule. Requires sphingomyelin for activity: stearoyl- sphingomyelin was the most effective, followed by palmitoyl- sphingomyelin and lignoceroyl-sphingomyelin. Activity was demonstrated only for sphingomyelin with a saturated fatty acid and not for that with an unsaturated fatty acid, rega [...]
23	BHLHA9	9606.ENSP00000375248	BHLHA9	Class A basic helix-loop-helix protein 9; Transcription factor, which play a role in limb development. Is an essential player in the regulatory network governing transcription of genes implicated in limb morphogenesis; Basic helix-loop-helix proteins
24	BICC1	9606.ENSP00000362993	BICC1	Protein bicaudal C homolog 1; Putative RNA-binding protein. Acts as a negative regulator of Wnt signaling. May be involved in regulating gene expression during embryonic development; Sterile alpha motif domain containing
25	BIK	9606.ENSP00000216115	BIK	Bcl-2-interacting killer; Accelerates programmed cell death. Association to the apoptosis repressors Bcl-X(L), BHRF1, Bcl-2 or its adenovirus homolog E1B 19k protein suppresses this death-promoting activity. Does not interact with BAX; BCL2 homology region 3 only
27	C11orf80	9606.ENSP00000354227	C11orf80	Type 2 DNA topoisomerase 6 subunit B-like; Isoform 3: Component of a topoisomerase 6 complex specifically required for meiotic recombination. Together with SPO11, mediates DNA cleavage that forms the double-strand breaks (DSB) that initiate meiotic recombination. The complex promotes relaxation of negative and positive supercoiled DNA and DNA decatenation through cleavage and ligation cycles
28	C14orf180	9606.ENSP00000333041	C14orf180	Nutritionally-regulated adipose and cardiac enriched protein homolog; Chromosome 14 open reading frame 180
29	C1orf53	9606.ENSP00000356363	C1orf53	Uncharacterized protein C1orf53; Chromosome 1 open reading frame 53
30	C1orf87	9606.ENSP00000360244	C1orf87	Uncharacterized protein C1orf87; Chromosome 1 open reading frame 87
32	C21orf91	9606.ENSP00000284881	C21orf91	Protein EURL homolog; Plays a role in cortical progenitor cell proliferation and differentiation. Promotes dendritic spine development of post- migratory cortical projection neurons by modulating the beta- catenin signaling pathway; Belongs to the EURL family
33	C2CD4D	9606.ENSP00000389554	C2CD4D	C2 calcium-dependent domain-containing protein 4D; C2 calcium dependent domain containing 4D
34	C8orf34	9606.ENSP00000427820	C8orf34	Uncharacterized protein C8orf34; Chromosome 8 open reading frame 34
35	CACNA2D1	9606.ENSP00000349320	CACNA2D1	Voltage-dependent calcium channel subunit alpha-2/delta-1; The alpha-2/delta subunit of voltage-dependent calcium channels regulates calcium current density and activation/inactivation kinetics of the calcium channel. Plays an important role in excitation-contraction coupling (By similarity)
36	CACNG5	9606.ENSP00000436836	CACNG5	Voltage-dependent calcium channel gamma-5 subunit; Regulates the gating properties of AMPA-selective glutamate receptors (AMPARs). Modulates their gating properties by accelerating their rates of activation, deactivation and desensitization. Displays subunit-specific AMPA receptor regulation. Shows specificity for GRIA1, GRIA4 and the long isoform of GRIA2. Thought to stabilize the calcium channel in an inactivated (closed) state (By similarity); Calcium channel auxiliary gamma subunits
37	CBLN2	9606.ENSP00000269503	CBLN2	Cerebellin-2; May play role in synaptogenesis induction
38	CCDC158	9606.ENSP00000373566	CCDC158	Coiled-coil domain containing 158
39	CCDC85A	9606.ENSP00000384040	CCDC85A	Coiled-coil domain containing 85A; Belongs to the CCDC85 family
41	CD300LG	9606.ENSP00000321005	CD300LG	CMRF35-like molecule 9; Receptor which may mediate L-selectin-dependent lymphocyte rollings. Binds SELL in a calcium dependent manner. Binds lymphocyte (By similarity); V-set domain containing
42	CD99L2	9606.ENSP00000480322	CD99L2	CD99 antigen-like protein 2; Plays a role in a late step of leukocyte extravasation helping cells to overcome the endothelial basement membrane. Acts at the same site as, but independently of, PECAM1 (By similarity). Homophilic adhesion molecule, but these interactions may not be required for cell aggregation (By similarity); Belongs to the CD99 family
43	CDH17	9606.ENSP00000027335	CDH17	Cadherin-17; Cadherins are calcium-dependent cell adhesion proteins. They preferentially interact with themselves in a homophilic manner in connecting cells; cadherins may thus contribute to the sorting of heterogeneous cell types. LI-cadherin may have a role in the morphological organization of liver and intestine. Involved in intestinal peptide transport
44	CEACAM21	9606.ENSP00000385739	CEACAM21	Carcinoembryonic antigen related cell adhesion molecule family; Belongs to the immunoglobulin superfamily. CEA family
45	CELF4	9606.ENSP00000410584	CELF4	CUGBP Elav-like family member 4; RNA-binding protein implicated in the regulation of pre- mRNA alternative splicing. Mediates exon inclusion and/or exclusion in pre-mRNA that are subject to tissue-specific and developmentally regulated alternative splicing. Specifically activates exon 5 inclusion of cardiac isoforms of TNNT2 during heart remodeling at the juvenile to adult transition. Promotes exclusion of both the smooth muscle (SM) and non-muscle (NM) exons in actinin pre-mRNAs. Activates the splicing of MAPT/Tau exon 10. Binds to muscle-specific splicing enhancer (MSE) intronic site [...]
46	CENPF	9606.ENSP00000355922	CENPF	Centromere protein F; Required for kinetochore function and chromosome segregation in mitosis. Required for kinetochore localization of dynein, LIS1, NDE1 and NDEL1. Regulates recycling of the plasma membrane by acting as a link between recycling vesicles and the microtubule network though its association with STX4 and SNAP25. Acts as a potential inhibitor of pocket protein-mediated cellular processes during development by regulating the activity of RB proteins during cell division and proliferation. May play a regulatory or permissive role in the normal embryonic cardiomyocyte cell cy [...]
47	CFHR4	9606.ENSP00000356386	CFHR4	Complement factor H-related protein 4; Involved in complement regulation. Can associate with lipoproteins and may play a role in lipid metabolism
48	CHRDL1	9606.ENSP00000361112	CHRDL1	Chordin-like protein 1; Antagonizes the function of BMP4 by binding to it and preventing its interaction with receptors. Alters the fate commitment of neural stem cells from gliogenesis to neurogenesis. Contributes to neuronal differentiation of neural stem cells in the brain by preventing the adoption of a glial fate. May play a crucial role in dorsoventral axis formation. May play a role in embryonic bone formation (By similarity). May also play an important role in regulating retinal angiogenesis through modulation of BMP4 actions in endothelial cells. Plays a role during anterior s [...]
49	CHRDL2	9606.ENSP00000263671	CHRDL2	Chordin-related protein 2 variant III; Chordin like 2
50	CHRNA9	9606.ENSP00000312663	CHRNA9	Neuronal acetylcholine receptor subunit alpha-9; Ionotropic receptor with a probable role in the modulation of auditory stimuli. Agonist binding induces a conformation change that leads to the opening of an ion-conducting channel across the plasma membrane. The channel is permeable to a range of divalent cations including calcium,

#	queryItem	stringId	preferredName	annotation
				the influx of which may activate a potassium current which hyperpolarizes the cell membrane. In the ear, this may lead to a reduction in basilar membrane motion, altering the activity of auditory nerve fibers and reducing the range of dynamic hearing. This ma [...]
51	CLEC3A	9606.ENSP0000029 9642	CLEC3A	C-type lectin domain family 3 member A; Promotes cell adhesion to laminin-332 and fibronectin; C-type lectin domain containing
52	CLSTN1	9606.ENSP0000036 6513	CLSTN1	Calsyntenin-1; Induces KLC1 association with vesicles and functions as a cargo in axonal anterograde transport. Complex formation with APBA2 and APP, stabilizes APP metabolism and enhances APBA2- mediated suppression of beta-APP40 secretion, due to the retardation of intracellular APP maturation. In complex with APBA2 and C99, a C-terminal APP fragment, abolishes C99 interaction with PSEN1 and thus APP C99 cleavage by gamma-secretase, most probably through stabilization of the direct interaction between APBA2 and APP. The intracellular fragment AlcICD suppresses APBB1-dependent transac [...]
53	CNKSR3	9606.ENSP0000047 5915	CNKSR3	Connector enhancer of kinase suppressor of ras 3; Involved in transepithelial sodium transport. Regulates aldosterone-induced and epithelial sodium channel (ENaC)-mediated sodium transport through regulation of ENaC cell surface expression. Acts as a scaffold protein coordinating the assembly of an ENaC-regulatory complex (ERC); PDZ domain containing
54	CNTN5	9606.ENSP0000043 5637	CNTN5	Contactin-5; Contactins mediate cell surface interactions during nervous system development. Has some neurite outgrowth-promoting activity in the cerebral cortical neurons but not in hippocampal neurons. Probably involved in neuronal activity in the auditory system (By similarity); Fibronectin type III domain containing
55	CNTROB	9606.ENSP0000036 9614	CNTROB	Centrobin; Required for centriole duplication. Inhibition of centriole duplication leading to defects in cytokinesis
56	COL12A1	9606.ENSP0000032 5146	COL12A1	Collagen alpha-1(XII) chain; Type XII collagen interacts with type I collagen- containing fibrils, the COL1 domain could be associated with the surface of the fibrils, and the COL2 and NC3 domains may be localized in the perifibrillar matrix; Belongs to the fibril-associated collagens with interrupted helices (FACIT) family
57	COX7C	9606.ENSP0000042 5759	COX7C	Cytochrome c oxidase subunit 7C, mitochondrial; This protein is one of the nuclear-coded polypeptide chains of cytochrome c oxidase, the terminal oxidase in mitochondrial electron transport
58	CPXM2	9606.ENSP0000024 1305	CPXM2	Inactive carboxypeptidase-like protein X2; May be involved in cell-cell interactions; M14 carboxypeptidases
59	CSRP1	9606.ENSP0000035 6275	CSRP1	Cysteine and glycine-rich protein 1; Could play a role in neuronal development; LIM domain containing
60	CSTF3	9606.ENSP0000031 5791	CSTF3	Cleavage stimulation factor subunit 3; One of the multiple factors required for polyadenylation and 3'-end cleavage of mammalian pre-mRNAs
61	CUEDC1	9606.ENSP0000046 2717	CUEDC1	CUE domain containing 1
62	CXCL8	9606.ENSP0000030 6512	CXCL8	Interleukin-8; IL-8 is a chemotactic factor that attracts neutrophils, basophils, and T-cells, but not monocytes. It is also involved in neutrophil activation. It is released from several cell types in response to an inflammatory stimulus. IL-8(6-77) has a 5-10-fold higher activity on neutrophil activation, IL-8(5-77) has increased activity on neutrophil activation and IL-8(7-77) has a higher affinity to receptors CXCR1 and CXCR2 as compared to IL-8(1-77), respectively; Chemokine ligands
63	CYP39A1	9606.ENSP0000027 5016	CYP39A1	24-hydroxycholesterol 7-alpha-hydroxylase; Involved in the bile acid metabolism. Has a preference for 24-hydroxycholesterol, and converts it into a 7-alpha-hydroxylated product; Cytochrome P450 family 39
64	DCTN1	9606.ENSP0000035 4791	DCTN1	Dynactin subunit 1; Plays a key role in dynein-mediated retrograde transport of vesicles and organelles along microtubules by recruiting and tethering dynein to microtubules. Binds to both dynein and microtubules providing a link between specific cargos, microtubules and dynein. Essential for targeting dynein to microtubule plus ends, recruiting dynein to membranous cargos and enhancing dynein processivity (the ability to move along a microtubule for a long distance without falling off the track). Can also act as a brake to slow the dynein motor during motility along the microtubule. C [...]
65	DIABLO	9606.ENSP0000039 8495	DIABLO	Diablo homolog, mitochondrial; Promotes apoptosis by activating caspases in the cytochrome c/Apaf-1/caspase-9 pathway. Acts by opposing the inhibitory activity of inhibitor of apoptosis proteins (IAP). Inhibits the activity of BIRC6/bruce by inhibiting its binding to caspases. Isoform 3 attenuates the stability and apoptosis-inhibiting activity of XIAP/BIRC4 by promoting XIAP/BIRC4 ubiquitination and degradation through the ubiquitin-proteasome pathway. Isoform 3 also disrupts XIAP/BIRC4 interacting with processed caspase-9 and promotes caspase-3 activation. Isoform 1 is defective in [...]
66	DNA2	9606.ENSP0000035 1185	DNA2	DNA replication ATP-dependent helicase/nuclease DNA2; Key enzyme involved in DNA replication and DNA repair in nucleus and mitochondrion. Involved in Okazaki fragments processing by cleaving long flaps that escape FEN1: flaps that are longer than 27 nucleotides are coated by replication protein A complex (RPA), leading to recruit DNA2 which cleaves the flap until it is too short to bind RPA and becomes a substrate for FEN1. Also involved in 5'-end resection of DNA during double- strand break (DSB) repair: recruited by BLM and mediates the cleavage of 5'-ssDNA, while the 3'-ssDNA cleava [...]
68	DPH1	9606.ENSP0000026 3083	DPH1	2-(3-amino-3-carboxypropyl)histidine synthase subunit 1; Required for the first step in the synthesis of diphthamide, a post-translational modification of histidine which occurs in translation elongation factor 2 (EEF2). When overexpressed, suppresses colony formation ability and growth rate of ovarian cancer cells. Acts also as a tumor suppressor in lung and breast cancers (By similarity). Plays a role in embryonic growth, organogenesis and postnatal survival (By similarity); Minor histocompatibility antigens
69	DSC3	9606.ENSP0000035 3608	DSC3	Desmocollin-3; Component of intercellular desmosome junctions. Involved in the interaction of plaque proteins and intermediate filaments mediating cell-cell adhesion. May contribute to epidermal cell positioning (stratification) by mediating differential adhesiveness between cells that express different isoforms; Desmosomal cadherins
70	DST	9606.ENSP0000030 7959	DST	Dystonin; Cytoskeletal linker protein. Acts as an integrator of intermediate filaments, actin and microtubule cytoskeleton networks. Required for anchoring either intermediate filaments to the actin cytoskeleton in neural and muscle cells or keratin- containing intermediate filaments to hemidesmosomes in epithelial cells. The proteins may self-aggregate to form filaments or a two- dimensional mesh. Regulates the organization and stability of the microtubule network of sensory neurons to allow axonal transport. Mediates docking of the dynein/dynactin motor complex to vesicle cargos for [...]
71	DUSP2	9606.ENSP0000028 8943	DUSP2	Dual specificity protein phosphatase 2; Regulates mitogenic signal transduction by dephosphorylating both Thr and Tyr residues on MAP kinases ERK1 and ERK2; Belongs to the protein-tyrosine phosphatase family. Non-receptor class dual specificity subfamily
73	EAF1	9606.ENSP0000038 0054	EAF1	ELL-associated factor 1; Acts as a transcriptional transactivator of ELL and ELL2 elongation activities; Belongs to the EAF family
74	ECE1	9606.ENSP0000036 4028	ECE1	Endothelin-converting enzyme 1; Converts big endothelin-1 to endothelin-1; Belongs to the peptidase M13 family
75	EGFR	9606.ENSP0000027 5493	EGFR	Epidermal growth factor receptor; Receptor tyrosine kinase binding ligands of the EGF family and activating several signaling cascades to convert extracellular cues into appropriate cellular responses. Known ligands include EGF, TGFA/TGF-alpha, amphiregulin, epigen/EPGN, BTC/betacellulin, epiregulin/EREG and HBEGF/heparin-binding EGF. Ligand binding triggers receptor homo- and/or heterodimerization and autophosphorylation on key cytoplasmic residues. The phosphorylated receptor recruits adapter proteins like GRB2 which in turn activates complex downstream signaling cascades. Activates [...]
76	EHMT1	9606.ENSP0000041 7980	EHMT1	Histone-lysine N-methyltransferase EHMT1; Histone methyltransferase that specifically mono- and dimethylates 'Lys-9' of histone H3 (H3K9me1 and H3K9me2, respectively) in euchromatin. H3K9me represents a specific tag for epigenetic transcriptional repression by recruiting HP1 proteins to methylated histones. Also weakly methylates 'Lys-27' of histone H3 (H3K27me). Also required for DNA methylation, the histone methyltransferase activity is not required for DNA methylation, suggesting that these 2 activities function independently. Probably targeted to histone H3 by different DNA-binding [...]
77	ELF5	9606.ENSP0000031 1010	ELF5	ETS-related transcription factor Elf-5; Transcriptionally activator that may play a role in regulating the later stages of keratinocytes terminal differentiation; Belongs to the ETS family
78	EPHA4	9606.ENSP0000028 1821	EPHA4	Ephrin type-A receptor 4; Receptor tyrosine kinase which binds membrane-bound ephrin family ligands residing on adjacent cells, leading to contact-dependent bidirectional signaling into neighboring cells. The signaling pathway downstream of the receptor is referred to as forward signaling while the signaling pathway downstream of the ephrin ligand is referred to as reverse signaling. Highly promiscuous, it has the unique property among Eph receptors to bind and to be physiologically activated by both GPI-anchored ephrin-A and transmembrane ephrin-B ligands including EFNA1 and EFNB3. Up [...]
79	EPSTI1	9606.ENSP0000031 8982	EPSTI1	Epithelial stromal interaction 1
80	ERBB4	9606.ENSP0000034 2235	ERBB4	Receptor tyrosine-protein kinase erbB-4; Tyrosine-protein kinase that plays an essential role as cell surface receptor for neuregulins and EGF family members and regulates development of the heart, the central nervous system and the mammary gland, gene transcription, cell proliferation, differentiation, migration and apoptosis. Required for normal cardiac muscle differentiation during embryonic development, and for postnatal cardiomyocyte proliferation. Required for normal development of the embryonic central nervous system, especially for normal neural crest cell migration and normal [...]
81	ERCC6L2	9606.ENSP0000028 8985	ERCC6L2	DNA excision repair protein ERCC-6-like 2; May be involved in early DNA damage response
82	ERICH1	9606.ENSP0000026 2109	ERICH1	Glutamate-rich protein 1; Glutamate rich 1
83	ESYT3	9606.ENSP0000037 4218	ESYT3	Extended synaptotagmin-3; Binds glycerophospholipids in a barrel-like domain and may play a role in cellular lipid transport (By similarity). Tethers the endoplasmic reticulum to the cell membrane and promotes the formation of appositions between the endoplasmic reticulum and the cell membrane; Extended synaptotagmins
84	EXOSC5	9606.ENSP0000022 1233	EXOSC5	Exosome complex component RRP46; Non-catalytic component of the RNA exosome complex which has 3'->5' exoribonuclease activity and participates in a multitude of cellular RNA processing and degradation events. In the nucleus, the RNA exosome complex is involved in proper maturation of stable RNA species such as rRNA, snRNA and snoRNA, in the elimination of RNA processing by-products and non-coding 'pervasive' transcripts, such as antisense RNA species and promoter-upstream transcripts (PROMPTs), and of mRNAs with processing defects, thereby limiting or excluding their export to the cyto [...]
85	EYA1	9606.ENSP0000034 2626	EYA1	Eyes absent homolog 1; Functions both as protein phosphatase and as transcriptional coactivator for SIX1, and probably also for SIX2, SIX4 and SIX5 (By similarity). Tyrosine phosphatase that dephosphorylates 'Tyr-142' of histone H2AX (H2AXY142ph) and promotes efficient DNA repair via the recruitment of DNA repair complexes containing MDC1. 'Tyr-142' phosphorylation of histone H2AX plays a central role in DNA repair and acts as a mark that distinguishes between apoptotic and repair responses to genotoxic stress. Its function as histone phosphatase may contribute to its function in trans [...]
86	FADD	9606.ENSP0000030 1838	FADD	FAS-associated death domain protein; Apoptotic adaptor molecule that recruits caspase-8 or caspase-10 to the activated Fas (CD95) or TNFR-1 receptors. The resulting aggregate called the death-inducing signaling complex (DISC) performs caspase-8 proteolytic activation. Active caspase-8 initiates the subsequent cascade of caspases mediating apoptosis. Involved in interferon-mediated antiviral immune response, playing a role in the positive regulation of interferon signaling; Death effector domain containing
87	FAM207A	9606.ENSP0000029 1634	FAM207A	Protein FAM207A; Family with sequence similarity 207 member A
88	FAM53B	9606.ENSP0000033 8532	FAM53B	Protein FAM53B; Acts as a regulator of Wnt signaling pathway by regulating beta-catenin (CTNNB1) nuclear localization
89	FAM53C	9606.ENSP0000023 9906	FAM53C	Protein FAM53C; Family with sequence similarity 53 member C
90	FAM83D	9606.ENSP0000048 1110	FAM83D	Protein FAM83D; Probable proto-oncogene that regulates cell proliferation, growth, migration and epithelial to mesenchymal transition. Through the degradation of FBXW7, may act indirectly on the expression and downstream signaling of MTOR, JUN and MYC. May play also a role in cell proliferation through activation of the ERK1/ERK2 signaling cascade. May also be important for proper chromosome congression and alignment during mitosis through its interaction with KIF22
91	FAT4	9606.ENSP0000037 7862	FAT4	Protocadherin Fat 4; Cadherins are calcium-dependent cell adhesion proteins. FAT4 plays a role in the maintenance of planar cell polarity as well as in inhibition of YAP1-mediated neuroprogenitor cell proliferation and differentiation (By similarity)
92	FGD4	9606.ENSP0000039 4487	FGD4	FYVE, RhoGEF and PH domain-containing protein 4; Activates CDC42, a member of the Ras-like family of Rho- and Rac proteins, by exchanging bound GDP for free GTP. Plays a role in regulating the actin cytoskeleton and cell shape. Activates MAPK8 (By similarity); Pleckstrin homology domain containing
93	FGF12	9606.ENSP0000041 3496	FGF12	Fibroblast growth factor 12; Involved in nervous system development and function. Involved in the positive regulation of voltage-gated sodium channel activity. Promotes neuronal excitability by elevating the voltage dependence of neuronal sodium channel SCN8A fast inactivation
94	FNDC3A	9606.ENSP0000044 1831	FNDC3A	Fibronectin type-III domain-containing protein 3A; Mediates spermatid-Sertoli adhesion during spermatogenesis; Belongs to the FNDC3 family
95	FOSB	9606.ENSP0000024 5919	FOSB	Protein fosB; FosB interacts with Jun proteins enhancing their DNA binding activity; Belongs to the bZIP family. Fos subfamily
96	FOXF1	9606.ENSP0000026 2426	FOXF1	Forkhead box protein F1; Probable transcription activator for a number of lung- specific genes; Forkhead boxes
97	FOXN3	9606.ENSP0000034 3288	FOXN3	Forkhead box protein N3; Acts as a transcriptional repressor. May be involved in DNA damage-inducible cell cycle arrests (checkpoints); Forkhead boxes
98	FRK	9606.ENSP0000047 6145	FRK	Tyrosine-protein kinase FRK; Non-receptor tyrosine-protein kinase that negatively regulates cell proliferation. Positively regulates PTEN protein stability through phosphorylation of PTEN on 'Tyr-336', which in turn prevents its ubiquitination and degradation, possibly by reducing its binding to NEDD4. May function as a tumor suppressor; SH2 domain containing

#	queryItem	stringId	preferredName	annotation
99	FTL	9606.ENSP00000366525	FTL	Ferritin light chain; Stores iron in a soluble, non-toxic, readily available form. Important for iron homeostasis. Iron is taken up in the ferrous form and deposited as ferric hydroxides after oxidation. Also plays a role in delivery of iron to cells. Mediates iron uptake in capsule cells of the developing kidney (By similarity); Belongs to the ferritin family
100	FXYD3	9606.ENSP00000473929	FXYD3	FXYD domain-containing ion transport regulator 3; Associates with and regulates the activity of the sodium/potassium-transporting ATPase (NKA) which transports Na(+) out of the cell and K(+) into the cell. Reduces glutathionylation of the NKA beta-1 subunit ATP1B1, thus reversing glutathionylation-mediated inhibition of ATP1B1. Induces a hyperpolarization-activated chloride current when expressed in Xenopus oocytes
101	FYB2	9606.ENSP00000345972	C1orf168	FYN-binding protein 2; Adapter protein that plays a role in T-cell receptor (TCR)-mediated activation of signaling pathways. Required for T- cell activation and integrin-mediated T-cell adhesion in response to TCR stimulation
102	FZD3	9606.ENSP00000437489	FZD3	Frizzled-3; Receptor for Wnt proteins. Most of frizzled receptors are coupled to the beta-catenin canonical signaling pathway, which leads to the activation of disheveled proteins, inhibition of GSK- 3 kinase, nuclear accumulation of beta-catenin and activation of Wnt target genes. A second signaling pathway involving PKC and calcium fluxes has been seen for some family members, but it is not yet clear if it represents a distinct pathway or if it can be integrated in the canonical pathway, as PKC seems to be required for Wnt-mediated inactivation of GSK-3 kinase. Both pathways seem to [...]
103	GALNT9	9606.ENSP00000380488	GALNT9	Polypeptide N-acetylgalactosaminyltransferase 9; Catalyzes the initial reaction in O-linked oligosaccharide biosynthesis, the transfer of an N-acetyl-D- galactosamine residue to a serine or threonine residue on the protein receptor. Does not glycosylate apomucin or SDC3; Polypeptide N-acetylgalactosaminyltransferases
104	GGA3	9606.ENSP00000438085	GGA3	ADP-ribosylation factor-binding protein GGA3; Plays a role in protein sorting and trafficking between the trans-Golgi network (TGN) and endosomes. Mediates the ARF- dependent recruitment of clathrin to the TGN and binds ubiquitinated proteins and membrane cargo molecules with a cytosolic acidic cluster-dileucine (AC-LL) motif
105	GPM6A	9606.ENSP00000280187	GPM6A	Neuronal membrane glycoprotein M6-a; Involved in neuronal differentiation, including differentiation and migration of neuronal stem cells. Plays a role in neuronal plasticity and is involved in neurite and filopodia outgrowth, filopodia motility and probably synapse formation. GPM6A-induced filopodia formation involves mitogen-activated protein kinase (MAPK) and Src signaling pathways. May be involved in neuronal NGF-dependent Ca(2+) influx. May be involved in regulation of endocytosis and intracellular trafficking of G- protein-coupled receptors (GPCRs); enhances internalization and r [...]
106	GPR39	9606.ENSP00000327417	GPR39	G-protein coupled receptor 39; Zn(2+) acts as an agonist. This receptor mediates its action by association with G proteins that activate a phosphatidylinositol-calcium second messenger system. Its effect is mediated mainly through G(q)-alpha and G(12)/G(13) proteins. Involved in regulation of body weight, gastrointestinal mobility, hormone secretion and cell death (By similarity); G protein-coupled receptors, Class A orphans
107	GRID1	9606.ENSP00000330148	GRID1	Glutamate receptor ionotropic, delta-1; Receptor for glutamate. L-glutamate acts as an excitatory neurotransmitter at many synapses in the central nervous system. The postsynaptic actions of Glu are mediated by a variety of receptors that are named according to their selective agonists; Belongs to the glutamate-gated ion channel (TC 1.A.10.1) family. GRID1 subfamily
108	GRIP2	9606.ENSP00000480660	GRIP2	Glutamate receptor-interacting protein 2; May play a role as a localized scaffold for the assembly of a multiprotein signaling complex and as mediator of the trafficking of its binding partners at specific subcellular location in neurons; PDZ domain containing
110	HACD3	9606.ENSP00000261875	PTPLAD1	Very-long-chain (3R)-3-hydroxyacyl-CoA dehydratase 3; Catalyzes the third of the four reactions of the long- chain fatty acids elongation cycle. This endoplasmic reticulum- bound enzymatic process, allows the addition of two carbons to the chain of long- and very long-chain fatty acids/VLCFAs per cycle. This enzyme catalyzes the dehydration of the 3-hydroxyacyl-CoA intermediate into trans-2,3-enoyl-CoA, within each cycle of fatty acid elongation. Thereby, it participates in the production of VLCFAs of different chain lengths that are involved in multiple biological processes as precurs [...]
111	HBQ1	9606.ENSP00000199708	HBQ1	Hemoglobin subunit theta 1
112	HDDC2	9606.ENSP00000381220	HDDC2	HD domain containing 2; Belongs to the HDDC2 family
113	HTR1D	9606.ENSP00000363748	HTR1D	5-hydroxytryptamine receptor 1D; G-protein coupled receptor for 5-hydroxytryptamine (serotonin). Also functions as a receptor for ergot alkaloid derivatives, various anxiolytic and antidepressant drugs and other psychoactive substances. Ligand binding causes a conformation change that triggers signaling via guanine nucleotide-binding proteins (G proteins) and modulates the activity of down-stream effectors, such as adenylate cyclase. Signaling inhibits adenylate cyclase activity. Regulates the release of 5-hydroxytryptamine in the brain, and thereby affects neural activity. May also pl [...]
114	IFFO2	9606.ENSP00000387941	IFFO2	Intermediate filament family orphan 2; Belongs to the intermediate filament family
115	IGF2	9606.ENSP00000391826	IGF2	Insulin-like growth factor II; The insulin-like growth factors possess growth-promoting activity. Major fetal growth hormone in mammals. Plays a key role in regulating fetoplacental development. IGF-II is influenced by placental lactogen. Also involved in tissue differentiation. Positively regulates myogenic transcription factor MYOD1 function by facilitating the recruitment of transcriptional coactivators, thereby controlling muscle terminal differentiation (By similarity). In adults, involved in glucose metabolism in adipose tissue, skeletal muscle and liver (Probable)
116	IGFALS	9606.ENSP00000416683	IGFALS	Insulin-like growth factor-binding protein complex acid labile subunit; Involved in protein-protein interactions that result in protein complexes, receptor-ligand binding or cell adhesion
117	IGSF11	9606.ENSP00000377370	IGSF11	Immunoglobulin superfamily member 11; Functions as a cell adhesion molecule through homophilic interaction. Stimulates cell growth; I-set domain containing
118	IL13RA1	9606.ENSP00000360730	IL13RA1	Interleukin-13 receptor subunit alpha-1; Binds with low affinity to interleukin-13 (IL13). Together with IL4RA can form a functional receptor for IL13. Also serves as an alternate accessory protein to the common cytokine receptor gamma chain for interleukin-4 (IL4) signaling, but cannot replace the function of IL2RG in allowing enhanced interleukin-2 (IL2) binding activity; Belongs to the type I cytokine receptor family. Type 5 subfamily
119	INPP4A	9606.ENSP00000074304	INPP4A	Type I inositol 3,4-bisphosphate 4-phosphatase; Catalyzes the hydrolysis of the 4-position phosphate of phosphatidylinositol 3,4-bisphosphate, inositol 1,3,4-trisphosphate and inositol 3,4-bisphosphate
120	IQGAP2	9606.ENSP00000274364	IQGAP2	Ras GTPase-activating-like protein IQGAP2; Binds to activated CDC42 and RAC1 but does not seem to stimulate their GTPase activity. Associates with calmodulin
121	KBTBD11	9606.ENSP00000321544	KBTBD11	Kelch repeat and BTB domain containing 11
122	KCNIP1	9606.ENSP00000414886	KCNIP1	Potassium voltage-gated channel interacting protein 1; EF-hand domain containing
123	KCNMB2	9606.ENSP00000319370	KCNMB3	Calcium-activated potassium channel subunit beta-3; Regulatory subunit of the calcium activated potassium KCNMA1 (maxiK) channel. Modulates the calcium sensitivity and gating kinetics of KCNMA1, thereby contributing to KCNMA1 channel diversity. Alters the functional properties of the current expressed by the KCNMA1 channel. Isoform 2, isoform 3 and isoform 4 partially inactivate the current of KCNBMA. Isoform 4 induces a fast and incomplete inactivation of KCNMA1 channel that is detectable only at large depolarizations. In contrast, isoform 1 does not induce detectable inactivation of [...]
124	KDM4A	9606.ENSP00000361473	KDM4A	Lysine-specific demethylase 4A; Histone demethylase that specifically demethylates 'Lys- 9' and 'Lys-36' residues of histone H3, thereby playing a central role in histone code. Does not demethylate histone H3 'Lys-4', H3 'Lys-27' nor H4 'Lys-20'. Demethylates trimethylated H3 'Lys-9' and H3 'Lys-36' residue, while it has no activity on mono- and dimethylated residues. Demethylation of Lys residue generates formaldehyde and succinate. Participates in transcriptional repression of ASCL2 and E2F-responsive promoters via the recruitment of histone deacetylases and NCOR1, respectively; Lysi [...]
125	KIAA2013	9606.ENSP00000365756	KIAA2013	Uncharacterized protein KIAA2013; KIAA2013
126	KIR3DL2	9606.ENSP00000325525	KIR3DL2	Killer cell immunoglobulin-like receptor 3DL2; Receptor on natural killer (NK) cells for HLA-A alleles. Inhibits the activity of NK cells thus preventing cell lysis; CD molecules
127	KLK4	9606.ENSP00000326159	KLK4	Kallikrein-4; Has a major role in enamel formation. Required during the maturation stage of tooth development for clearance of enamel proteins and normal structural patterning of the crystalline matrix (By similarity); Kallikreins
128	KMT5B	9606.ENSP00000305899	SUV420H1	Histone-lysine N-methyltransferase KMT5B; Histone methyltransferase that specifically trimethylates 'Lys-20' of histone H4. H4 'Lys-20' trimethylation represents a specific tag for epigenetic transcriptional repression. Mainly functions in pericentric heterochromatin regions, thereby playing a central role in the establishment of constitutive heterochromatin in these regions. KMT5B is targeted to histone H3 via its interaction with RB1 family proteins (RB1, RBL1 and RBL2) (By similarity). Plays a role in myogenesis by regulating the expression of target genes, such as EID3
129	LAMB4	9606.ENSP00000373433	LAMB4	Laminin subunit beta-4; Binding to cells via a high affinity receptor, laminin is thought to mediate the attachment, migration and organization of cells into tissues during embryonic development by interacting with other extracellular matrix components
130	LARP1	9606.ENSP00000336721	LARP1	La-related protein 1; RNA-binding protein that promotes translation of specific classes of mRNAs downstream of the mTORC1 complex. Associates with the mRNA 5'cap in an MTOR-dependent manner and associates with mRNAs containing a 5' terminal oligopyrimidine (5'TOP) motif, which is present in mRNAs encoding for ribosomal proteins and several components of the translation machinery. Associates with actively translating ribosomes via interaction with PABPC1/PABP and stimulates translation of mRNAs containing a 5'TOP, thereby regulating cell growth and proliferation. Positively regulates th [...]
131	LEP	9606.ENSP00000312652	LEP	Leptin; Key player in the regulation of energy balance and body weight control. Once released into the circulation, has central and peripheral effects by binding LEPR, found in many tissues, which results in the activation of several major signaling pathways. In the hypothalamus, acts as an appetite-regulating factor that induces a decrease in food intake and an increase in energy consumption by inducing anorexinogenic factors and suppressing orexigenic neuropeptides, also regulates bone mass and secretion of hypothalamo-pituitary-adrenal hormones. In the periphery, increases basal met [...]
133	LINGO2	9606.ENSP00000369328	LINGO2	Leucine-rich repeat and immunoglobulin-like domain-containing nogo receptor-interacting protein 2; Leucine rich repeat and Ig domain containing 2
134	LMOD1	9606.ENSP00000356257	LMOD1	Leiomodin-1; Mediates nucleation of actin filaments; Belongs to the tropomodulin family
135	LNPK	9606.ENSP00000272748	KIAA1715	Endoplasmic reticulum junction formation protein lunapark; Endoplasmic reticulum (ER)-shaping membrane protein that plays a role in determining ER morphology. Involved in the stabilization of nascent three-way ER tubular junctions within the ER network. May also play a role as a curvature-stabilizing protein within the three-way ER tubular junction network. May be involved in limb and central nervous system development (By similarity)
137	LRCH1	9606.ENSP00000374447	LRCH1	Leucine-rich repeat and calponin homology domain-containing protein 1; Acts as a negative regulator of GTPase CDC42 by sequestering CDC42-guanine exchange factor DOCK8. Probably by preventing CDC42 activation, negatively regulates CD4(+) T-cell migration
138	LRP5L	9606.ENSP00000482378	LRP5L	Low-density lipoprotein receptor-related protein 5-like protein; LDL receptor related protein 5 like
139	LRRC15	9606.ENSP00000413707	LRRC15	Leucine-rich repeat-containing protein 15; Leucine rich repeat containing 15
140	LRRC28	9606.ENSP00000304923	LRRC28	Leucine-rich repeat-containing protein 28; Leucine rich repeat containing 28
141	MAMSTR	9606.ENSP00000324175	MAMSTR	MEF2-activating motif and SAP domain-containing transcriptional regulator; Transcriptional coactivator. Stimulates the transcriptional activity of MEF2C. Stimulates MYOD1 activity in part via MEF2, resulting in an enhancement of skeletal muscle differentiation (By similarity); Myocardin family
142	MAP4K1	9606.ENSP00000465039	MAP4K1	Mitogen-activated protein kinase kinase kinase kinase 1; Serine/threonine-protein kinase, which may play a role in the response to environmental stress. Appears to act upstream of the JUN N-terminal pathway. May play a role in hematopoietic lineage decisions and growth regulation. Able to autophosphorylate
143	MAPK8	9606.ENSP00000378974	MAPK8	Mitogen-activated protein kinase 8; Serine/threonine-protein kinase involved in various processes such as cell proliferation, differentiation, migration, transformation and programmed cell death. Extracellular stimuli such as proinflammatory cytokines or physical stress stimulate the stress-activated protein kinase/c-Jun N-terminal kinase (SAP/JNK) signaling pathway. In this cascade, two dual specificity kinases MAP2K4/MKK4 and MAP2K7/MKK7 phosphorylate and activate MAPK8/JNK1. In turn, MAPK8/JNK1 phosphorylates a number of transcription factors, primarily components of AP-1 such as JU [...]
144	MARK4	9606.ENSP00000262891	MARK4	MAP/microtubule affinity-regulating kinase 4; Serine/threonine-protein kinase. Phosphorylates the microtubule-associated protein MAPT. Also phosphorylates the microtubule-associated proteins MAP2 and MAP4. Involved in regulation of the microtubule network, causing reorganization of microtubules into bundles. Required for the initiation of axoneme extension during cilium assembly. Regulates the centrosomal location of ODF2 and phosphorylates ODF2 in vitro. Plays a role in cell cycle progression, specifically in the G1/S checkpoint. Reduces neuronal cell survival. Plays a role in energy [...]
145	MDGA2	9606.ENSP00000382178	MDGA2	MAM domain-containing glycosylphosphatidylinositol anchor protein 2; May be involved in cell-cell interactions; I-set domain containing
146	ME3	9606.ENSP00000440246	ME3	NADP-dependent malic enzyme, mitochondrial; Malic enzyme 3; Belongs to the malic enzymes family
147	METRNL	9606.ENSP00000315731	METRNL	Meteorin-like protein; Hormone induced following exercise or cold exposure that promotes energy expenditure. Induced either in the skeletal muscle after exercise or in adipose tissue following cold exposure and is present in the circulation. Able to stimulate energy expenditure associated with the browning of the white fat depots and improves glucose tolerance. Does not promote an increase in a thermogenic gene program via direct action on adipocytes, but acts by stimulating several immune cell subtypes to enter the adipose tissue and activate their prothermogenic actions. Stimulates a [...]

Depression (PHQ-9)

#	queryItem	stringId	preferredName	annotation
148	MGP	9606.ENSP00000228938	MGP	Matrix Gla protein; Gla domain containing
149	MGRN1	9606.ENSP00000262370	MGRN1	E3 ubiquitin-protein ligase MGRN1; E3 ubiquitin-protein ligase. Mediates monoubiquitination at multiple sites of TSG101 in the presence of UBE2D1, but not of UBE2G1, nor UBE2H. Plays a role in the regulation of endosome-to- lysosome trafficking. Impairs MC1R- and MC4R-signaling by competing with GNAS-binding to MCRs and inhibiting agonist-induced cAMP production. Does not inhibit ADRB2-signaling. Does not promote MC1R ubiquitination; Ring finger proteins
150	MLN	9606.ENSP00000388825	MLN	Promotilin; Plays an important role in the regulation of interdigestive gastrointestinal motility and indirectly causes rhythmic contraction of duodenal and colonic smooth muscle; Endogenous ligands
152	MTCL1	9606.ENSP00000352927	MTCL1	Microtubule cross-linking factor 1; Microtubule-associated factor involved in the late phase of epithelial polarization and microtubule dynamics regulation. Plays a role in the development and maintenance of non-centrosomal microtubule bundles at the lateral membrane in polarized epithelial cells; Belongs to the SOGA family
153	MTX2	9606.ENSP00000249442	MTX2	Metaxin-2; Involved in transport of proteins into the mitochondrion
154	NBPF1	9606.ENSP00000474456	NBPF1	Neuroblastoma breakpoint family member 1; NBPF member 1
155	NFE2L3	9606.ENSP00000056233	NFE2L3	Nuclear factor erythroid 2-related factor 3; Activates erythroid-specific, globin gene expression; Basic leucine zipper proteins
156	NFIX	9606.ENSP00000380781	NFIX	Nuclear factor 1 X-type; Recognizes and binds the palindromic sequence 5'- TTGGCNNNNNGCCAA-3' present in viral and cellular promoters and in the origin of replication of adenovirus type 2. These proteins are individually capable of activating transcription and replication
157	NHS	9606.ENSP00000369400	NHS	Nance-Horan syndrome protein; May function in cell morphology by maintaining the integrity of the circumferential actin ring and controlling lamellipod formation. Involved in the regulation eye, tooth, brain and craniofacial development; Belongs to the NHS family
158	NKAIN1	9606.ENSP00000362841	NKAIN1	Sodium/potassium-transporting ATPase subunit beta-1-interacting protein 1; Sodium/potassium transporting ATPase interacting 1
159	NKX2-5	9606.ENSP00000327758	NKX2-5	Homeobox protein Nkx-2.5; Implicated in commitment to and/or differentiation of the myocardial lineage. Acts as a transcriptional activator of ANF in cooperation with GATA4 (By similarity). Binds to the core DNA motif of NPPA promoter. It is transcriptionally controlled by PBX1 and acts as a transcriptional repressor of CDKN2B (By similarity). It is required for spleen development; NKL subclass homeoboxes and pseudogenes
160	NMI	9606.ENSP00000243346	NMI	N-myc-interactor; May be involved in augmenting coactivator protein recruitment to a group of sequence-specific transcription factors. Augments cytokine-mediated STAT transcription. Enhances CBP/p300 coactivator protein recruitment to STAT1 and STAT5; Belongs to the NMI family
161	NOD1	9606.ENSP00000222823	NOD1	Nucleotide-binding oligomerization domain-containing protein 1; Enhances caspase-9-mediated apoptosis. Induces NF-kappa- B activity via RIPK2 and IKK-gamma. Confers responsiveness to intracellular bacterial lipopolysaccharides (LPS). Forms an intracellular sensing system along with ARHGEF2 for the detection of microbial effectors during cell invasion by pathogens. Required for RHOA and RIPK2 dependent NF-kappa-B signaling pathway activation upon S.flexneri cell invasion. Involved not only in sensing peptidoglycan (PGN)-derived muropeptides but also in the activation of NF-kappa-B by Sh [...]
162	NOL4L	9606.ENSP00000483523	NOL4L	Nucleolar protein 4 like
163	NONO	9606.ENSP00000276079	NONO	Non-POU domain-containing octamer-binding protein; DNA- and RNA binding protein, involved in several nuclear processes. Binds the conventional octamer sequence in double-stranded DNA. Also binds single-stranded DNA and RNA at a site independent of the duplex site. Involved in pre-mRNA splicing, probably as a heterodimer with SFPQ. Interacts with U5 snRNA, probably by binding to a purine-rich sequence located on the 3' side of U5 snRNA stem 1b. Together with PSPC1, required for the formation of nuclear paraspeckles. The SFPQ-NONO heteromer associated with MATR3 may play a role in nuclea [...]
164	NOX4	9606.ENSP00000263317	NOX4	NADPH oxidase 4; Constitutive NADPH oxidase which generates superoxide intracellularly upon formation of a complex with CYBA/p22phox. Regulates signaling cascades probably through phosphatases inhibition. May function as an oxygen sensor regulating the KCNK3/TASK-1 potassium channel and HIF1A activity. May regulate insulin signaling cascade. May play a role in apoptosis, bone resorption and lipolysaccharide-mediated activation of NFKB. May produce superoxide in the nucleus and play a role in regulating gene expression upon cell stimulation. Isoform 3 is not functional. Isoform 5 and is [...]
165	NPFFR2	9606.ENSP00000307822	NPFFR2	Neuropeptide FF receptor 2; Receptor for NPAF (A-18-F-amide) and NPFF (F-8-F-amide) neuropeptides, also known as morphine-modulating peptides. Can also be activated by a variety of naturally occurring or synthetic FMRF-amide like ligands. This receptor mediates its action by association with G proteins that activate a phosphatidylinositol- calcium second messenger system
166	NR4A2	9606.ENSP00000344479	NR4A2	Nuclear receptor subfamily 4 group A member 2; Transcriptional regulator which is important for the differentiation and maintenance of meso-diencephalic dopaminergic (mdDA) neurons during development. It is crucial for expression of a set of genes such as SLC6A3, SLC18A2, TH and DRD2 which are essential for development of mdDA neurons (By similarity); Belongs to the nuclear hormone receptor family. NR4 subfamily
167	NXN	9606.ENSP00000337443	NXN	Nucleoredoxin; Functions as a redox-dependent negative regulator of the Wnt signaling pathway, possibly by preventing ubiquitination of DVL3 by the BCR(KLHL12) complex. May also function as a transcriptional regulator act as a regulator of protein phosphatase 2A (PP2A) (By similarity); Nucleoredoxin family
168	OLIG3	9606.ENSP00000356708	OLIG3	Oligodendrocyte transcription factor 3; May determine the distinct specification program of class A neurons in the dorsal part of the spinal cord and suppress specification of class B neurons; Basic helix-loop-helix proteins
169	OOSP2	9606.ENSP00000278855	OOSP2	Oocyte secreted protein 2
170	OR4X1	9606.ENSP00000321506	OR4X1	Olfactory receptor 4X1; Odorant receptor; Olfactory receptors, family 4
171	OSBP2	9606.ENSP00000332576	OSBP2	Oxysterol-binding protein 2; Binds 7-ketocholesterol; Oxysterol binding proteins
172	OSTM1	9606.ENSP00000193322	OSTM1	Osteopetrosis-associated transmembrane protein 1; Required for osteoclast and melanocyte maturation and function
173	PAK1	9606.ENSP00000278568	PAK1	Serine/threonine-protein kinase PAK 1; Protein kinase involved in intracellular signaling pathways downstream of integrins and receptor-type kinases that plays an important role in cytoskeleton dynamics, in cell adhesion, migration, proliferation, apoptosis, mitosis, and in vesicle-mediated transport processes. Can directly phosphorylate BAD and protects cells against apoptosis. Activated by interaction with CDC42 and RAC1. Functions as GTPase effector that links the Rho-related GTPases CDC42 and RAC1 to the JNK MAP kinase pathway. Phosphorylates and activates MAP2K1, and thereby media [...]
174	PAQR4	9606.ENSP00000321804	PAQR4	Progestin and adipoQ receptor family member 4
175	PARP15	9606.ENSP00000417214	PARP15	Poly [ADP-ribose] polymerase 15; Possesses ADP-ribosyltransferase activity. Transcriptional repressor; Poly(ADP-ribose) polymerases
176	PCDH9	9606.ENSP00000367096	PCDH9	Protocadherin-9; Potential calcium-dependent cell-adhesion protein; Non-clustered protocadherins
177	PCDHB12	9606.ENSP00000239450	PCDHB12	Protocadherin beta-12; Potential calcium-dependent cell-adhesion protein. May be involved in the establishment and maintenance of specific neuronal connections in the brain; Clustered protocadherins
178	PCDHB3	9606.ENSP00000231130	PCDHB3	Protocadherin beta-3; Potential calcium-dependent cell-adhesion protein. May be involved in the establishment and maintenance of specific neuronal connections in the brain; Clustered protocadherins
179	PDZD8	9606.ENSP00000334642	PDZD8	PDZ domain-containing protein 8; Molecular tethering protein that connects endoplasmic reticulum and mitochondria membranes. PDZD8- dependent endoplasmic reticulum-mitochondria membrane tethering is essential for endoplasmic reticulum-mitochondria Ca(2+) transfer. In neurons, involved in the regulation of dendritic Ca(2+) dynamics by regulating mitochondrial Ca(2+) uptake in neurons. Plays an indirect role in the regulation of cell morphology and cytoskeletal organization. May inhibit herpes simplex virus 1 infection at an early stage; PDZ domain containing
180	PGK1	9606.ENSP00000362413	PGK1	Phosphoglycerate kinase 1; In addition to its role as a glycolytic enzyme, it seems that PGK-1 acts as a polymerase alpha cofactor protein (primer recognition protein). May play a role in sperm motility
181	PIMREG	9606.ENSP00000250056	FAM64A	Protein PIMREG; During mitosis, may play a role in the control of metaphase-to-anaphase transition
182	PISD	9606.ENSP00000371586	PISD	Phosphatidylserine decarboxylase proenzyme, mitochondrial; Catalyzes the formation of phosphatidylethanolamine (PtdEtn) from phosphatidylserine (PtdSer). Plays a central role in phospholipid metabolism and in the interorganelle trafficking of phosphatidylserine; Belongs to the phosphatidylserine decarboxylase family. PSD-B subfamily. Eukaryotic type I sub-subfamily
183	PKDCC	9606.ENSP00000294964	PKDCC	Extracellular tyrosine-protein kinase PKDCC; Secreted tyrosine-protein kinase that mediates phosphorylation of extracellular proteins and endogenous proteins in the secretory pathway, which is essential for patterning at organogenesis stages. Mediates phosphorylation of MMP1, MMP13, MMP14, MMP19 and ERP29. Probably plays a role in platelets: rapidly and quantitatively secreted from platelets in response to stimulation of platelet degranulation. May also have serine/threonine protein kinase activity. Required for longitudinal bone growth through regulation of chondrocyte differentiation [...]
184	PLCB3	9606.ENSP00000443631	PLCB3	1-phosphatidylinositol 4,5-bisphosphate phosphodiesterase beta-3; The production of the second messenger molecules diacylglycerol (DAG) and inositol 1,4,5-trisphosphate (IP3) is mediated by activated phosphatidylinositol-specific phospholipase C enzymes; C2 domain containing phospholipases
185	PLEKHM3	9606.ENSP00000417003	PLEKHM3	Pleckstrin homology domain containing M3
186	PLEKHO2	9606.ENSP00000326706	PLEKHO2	Pleckstrin homology domain containing O2
187	POLL	9606.ENSP00000359181	POLL	DNA polymerase lambda; DNA polymerase that functions in several pathways of DNA repair. Involved in base excision repair (BER) responsible for repair of lesions that give rise to abasic (AP) sites in DNA. Also contributes to DNA double-strand break repair by non-homologous end joining and homologous recombination. Has both template-dependent and template-independent (terminal transferase) DNA polymerase activities. Has also a 5'-deoxyribose-5- phosphate lyase (dRP lyase) activity; Belongs to the DNA polymerase type-X family
188	PPDPFL	9606.ENSP00000304926	C8orf22	Pancreatic progenitor cell differentiation and proliferation factor-like protein; Chromosome 8 open reading frame 22
189	PPFIA1	9606.ENSP00000253925	PPFIA1	Liprin-alpha-1; May regulate the disassembly of focal adhesions. May localize receptor-like tyrosine phosphatases type 2A at specific sites on the plasma membrane, possibly regulating their interaction with the extracellular environment and their association with substrates; Sterile alpha motif domain containing
190	PPP2R3A	9606.ENSP00000264977	PPP2R3A	Serine/threonine-protein phosphatase 2A regulatory subunit B'' subunit alpha; The B regulatory subunit might modulate substrate selectivity and catalytic activity, and also might direct the localization of the catalytic enzyme to a particular subcellular compartment; EF-hand domain containing
191	PPP2R5A	9606.ENSP00000261461	PPP2R5A	Serine/threonine-protein phosphatase 2A 56 kDa regulatory subunit alpha isoform; The B regulatory subunit might modulate substrate selectivity and catalytic activity, and also might direct the localization of the catalytic enzyme to a particular subcellular compartment; Armadillo-like helical domain containing
192	PRKCQ	9606.ENSP00000263125	PRKCQ	Protein kinase C theta type; Calcium-independent, phospholipid- and diacylglycerol (DAG)-dependent serine/threonine-protein kinase that mediates non- redundant functions in T-cell receptor (TCR) signaling, including T-cells activation, proliferation, differentiation and survival, by mediating activation of multiple transcription factors such as NF-kappa-B, JUN, NFATC1 and NFATC2. In TCR-CD3/CD28-co-stimulated T-cells, is required for the activation of NF-kappa-B and JUN, which in turn are essential for IL2 production, and participates in the calcium-dependent NFATC1 and NFATC2 transact [...]
193	PRRT1	9606.ENSP00000211413	PRRT1	Interferon induced transmembrane protein domain containing; Belongs to the CD225/Dispanin family
194	PTCD2	9606.ENSP00000370013	PTCD2	Pentatricopeptide repeat-containing protein 2, mitochondrial; Involved in mitochondrial RNA maturation and mitochondrial respiratory chain function
195	QSER1	9606.ENSP00000382241	QSER1	Glutamine and serine rich 1
196	RAB3C	9606.ENSP00000282878	RAB3C	Ras-related protein Rab-3C; Protein transport. Probably involved in vesicular traffic (By similarity); Belongs to the small GTPase superfamily. Rab family
197	RAB6A	9606.ENSP00000311449	RAB6A	Ras-related protein Rab-6A; Protein transport. Regulator of membrane traffic from the Golgi apparatus towards the endoplasmic reticulum (ER). Has a low GTPase activity. Involved in COPI-independent retrograde transport from the Golgi to the ER; RAB, member RAS oncogene GTPases
198	RABGEF1	9606.ENSP00000370208	RABGEF1	RAB guanine nucleotide exchange factor 1; VPS9 domain containing
199	RAD18	9606.ENSP00000264926	RAD18	E3 ubiquitin-protein ligase RAD18; E3 ubiquitin-protein ligase involved in postreplication repair of UV-damaged DNA. Postreplication repair functions in gap- filling of a daughter strand on replication of damaged DNA. Associates to the E2 ubiquitin conjugating enzyme UBE2B to form the UBE2B-RAD18 ubiquitin ligase complex involved in mono- ubiquitination of DNA-associated PCNA on 'Lys-164'. Has ssDNA binding activity

Depression (PHQ-9)

#	queryItem	stringId	preferredName	annotation
200	RAD50	9606.ENSP00000368100	RAD50	DNA repair protein RAD50; Component of the MRN complex, which plays a central role in double-strand break (DSB) repair, DNA recombination, maintenance of telomere integrity and meiosis. The complex possesses single-strand endonuclease activity and double-strand- specific 3'-5' exonuclease activity, which are provided by MRE11. RAD50 may be required to bind DNA ends and hold them in close proximity. This could facilitate searches for short or long regions of sequence homology in the recombining DNA templates, and may also stimulate the activity of DNA ligases and/or restrict the nucleas [...]
201	RAP2A	9606.ENSP00000245304	RAP2A	Ras-related protein Rap-2a; Small GTP-binding protein which cycles between a GDP- bound inactive and a GTP-bound active form. In its active form interacts with and regulates several effectors including MAP4K4, MINK1 and TNIK. Part of a signaling complex composed of NEDD4, RAP2A and TNIK which regulates neuronal dendrite extension and arborization during development. More generally, it is part of several signaling cascades and may regulate cytoskeletal rearrangements, cell migration, cell adhesion and cell spreading; RAS type GTPase family
202	RASGRF1	9606.ENSP00000405963	RASGRF1	Ras-specific guanine nucleotide-releasing factor 1; Promotes the exchange of Ras-bound GDP by GTP; Pleckstrin homology domain containing
203	RCC1	9606.ENSP00000362937	RCC1	Regulator of chromosome condensation; Guanine-nucleotide releasing factor that promotes the exchange of Ran-bound GDP by GTP. Involved in the regulation of onset of chromosome condensation in the S phase. Binds both to the nucleosomes and double-stranded DNA. RCC1-Ran complex (together with other proteins) acts as a component of a signal transmission pathway that detects unreplicated DNA. Plays a key role in nucleo- cytoplasmic transport, mitosis and nuclear-envelope assembly
204	REEP3	9606.ENSP00000362863	REEP3	Receptor expression-enhancing protein 3; Microtubule-binding protein required to ensure proper cell division and nuclear envelope reassembly by sequestering the endoplasmic reticulum away from chromosomes during mitosis. Probably acts by clearing the endoplasmic reticulum membrane from metaphase chromosomes; Belongs to the DP1 family
206	RHOH	9606.ENSP00000371219	RHOH	Rho-related GTP-binding protein RhoH; Negative regulator of hematopoietic progenitor cell proliferation, survival and migration. Critical regulator of thymocyte development and T-cell antigen receptor (TCR) signaling by mediating recruitment and activation of ZAP70. Required for phosphorylation of CD3Z, membrane translocation of ZAP70 and subsequent activation of the ZAP70-mediated pathways. Essential for efficient beta-selection and positive selection by promoting the ZAP70-dependent phosphorylation of the LAT signalosome during pre-TCR and TCR signaling. Crucial for thymocyte maturat [...]
207	RND3	9606.ENSP00000364886	RND3	Rho-related GTP-binding protein RhoE; Binds GTP but lacks intrinsic GTPase activity and is resistant to Rho-specific GTPase-activating proteins; Rho family GTPases
208	RNMT	9606.ENSP00000372804	RNMT	mRNA cap guanine-N7 methyltransferase; Catalytic subunit of the mRNA-capping methyltransferase RNMT:RAM/FAM103A1 complex that methylates the N7 position of the added guanosine to the 5'-cap structure of mRNAs. Binds RNA containing 5'-terminal GpppC
209	RPRM	9606.ENSP00000314946	RPRM	Protein reprimo; May be involved in the regulation of p53-dependent G2 arrest of the cell cycle. Seems to induce cell cycle arrest by inhibiting CDK1 activity and nuclear translocation of the CDC2 cyclin B1 complex (By similarity)
210	RTL8A	9606.ENSP00000375267	FAM127B	Retrotransposon Gag-like protein 8A; Family with sequence similarity 127, member B; Belongs to the FAM127 family
211	RUNX1T1	9606.ENSP00000402257	RUNX1T1	Protein CBFA2T1; Transcriptional corepressor which facilitates transcriptional repression via its association with DNA-binding transcription factors and recruitment of other corepressors and histone-modifying enzymes. Can repress the expression of MMP7 in a ZBTB33-dependent manner. Can repress transactivation mediated by TCF12. Acts as a negative regulator of adipogenesis (By similarity). The AML1-MTG8/ETO fusion protein frequently found in leukemic cells is involved in leukemogenesis and contributes to hematopoietic stem/progenitor cell self-renewal; Zinc fingers MYND-type
212	SCTR	9606.ENSP00000019103	SCTR	Secretin receptor; This is a receptor for secretin. The activity of this receptor is mediated by G proteins which activate adenylyl cyclase; Glucagon receptor family
213	SDK2	9606.ENSP00000376421	SDK2	Protein sidekick-2; Adhesion molecule that promotes lamina-specific synaptic connections in the retina and is specifically required for the formation of neuronal circuits that detect motion. Acts by promoting formation of synapses between two specific retinal cell types: the retinal ganglion cells W3B-RGCs and the excitatory amacrine cells VG3-ACs. Formation of synapses between these two cells plays a key role in detection of motion. Promotes synaptic connectivity via homophilic interactions; Fibronectin type III domain containing
214	SEMA6A	9606.ENSP00000257414	SEMA6A	Semaphorin-6A; Cell surface receptor for PLXNA2 that plays an important role in cell-cell signaling. Required for normal granule cell migration in the developing cerebellum. Promotes reorganization of the actin cytoskeleton and plays an important role in axon guidance in the developing central nervous system. Can act as repulsive axon guidance cue. Has repulsive action towards migrating granular neurons. May play a role in channeling sympathetic axons into the sympathetic chains and controlling the temporal sequence of sympathetic target innervation (By similarity); Belongs to the sema [...]
215	SERINC5	9606.ENSP00000426237	SERINC5	Serine incorporator 5; Restriction factor required to restrict infectivity of lentiviruses, such as HIV-1: acts by inhibiting an early step of viral infection. Impairs the penetration of the viral particle into the cytoplasm. Enhances the incorporation of serine into phosphatidylserine and sphingolipids. May play a role in providing serine molecules for the formation of myelin glycosphingolipids in oligodendrocytes (By similarity)
216	SERPINC1	9606.ENSP00000356671	SERPINC1	Antithrombin-III; Most important serine protease inhibitor in plasma that regulates the blood coagulation cascade. AT-III inhibits thrombin, matriptase-3/TMPRSS7, as well as factors IXa, Xa and XIa. Its inhibitory activity is greatly enhanced in the presence of heparin; Serpin peptidase inhibitors
218	SH3RF1	9606.ENSP00000284637	SH3RF1	E3 ubiquitin-protein ligase SH3RF1; Acts as a scaffold protein, contributes to Rac-induced signal transduction such as JNKs (MAPK8 and MAPK9) activation and induces apoptosis. Within a signaling complex, it probably recruits protein kinases such as MAP3K10 or MAP3K11 which are in turn activated leading to the sequential activation of MAP2K4, MAP2K7 and JNKs (MAPK8 and MAPK9) (By similarity). May be involved in targeting of HIV-1 GAG and GAG-POL polyproteins to the plasma membrane; Belongs to the SH3RF family
219	SHANK2	9606.ENSP00000469689	SHANK2	SH3 and multiple ankyrin repeat domains protein 2; Seems to be an adapter protein in the postsynaptic density (PSD) of excitatory synapses that interconnects receptors of the postsynaptic membrane including NMDA-type and metabotropic glutamate receptors, and the actin-based cytoskeleton. May play a role in the structural and functional organization of the dendritic spine and synaptic junction; Belongs to the SHANK family
220	SIM2	9606.ENSP00000290399	SIM2	Single-minded homolog 2; Transcription factor that may be a master gene of CNS development in cooperation with Arnt. It may have pleiotropic effects in the tissues expressed during development; Basic helix-loop-helix proteins
221	SLC15A4	9606.ENSP00000266771	SLC15A4	Solute carrier family 15 member 4; Proton oligopeptide cotransporter. Transports free histidine and certain di- and tripeptides; Solute carriers
222	SLC16A12	9606.ENSP00000360855	SLC16A12	Monocarboxylate transporter 12; Proton-linked monocarboxylate transporter that mediates creatine transport across the plasma membrane; Belongs to the major facilitator superfamily. Monocarboxylate porter (TC 2.A.1.13) family
223	SLC26A10	9606.ENSP00000320217	SLC26A10	Solute carrier family 26 member 10; Chloride/bicarbonate exchanger; Solute carriers
224	SLC39A14	9606.ENSP00000352779	SLC39A14	Zinc transporter ZIP14; Broad-scope metal ion transporter with a preference for zinc uptake. Also mediates cellular uptake of nontransferrin-bound iron; Belongs to the ZIP transporter (TC 2.A.5) family
226	SLC52A1	9606.ENSP00000399979	SLC52A1	Solute carrier family 52, riboflavin transporter, member 1; Riboflavin transporter. Riboflavin transport is Na(+)- independent but moderately pH-sensitive. Activity is strongly inhibited by riboflavin analogs, such as lumiflavin. Weakly inhibited by flavin adenine dinucleotide (FAD). In case of infection by retroviruses, acts as a cell receptor to retroviral envelopes similar to the porcine endogenous retrovirus (PERV-A)
227	SLIT1	9606.ENSP00000266058	SLIT1	Slit homolog 1 protein; Thought to act as molecular guidance cue in cellular migration, and function appears to be mediated by interaction with roundabout homolog receptors. During neural development involved in axonal navigation at the ventral midline of the neural tube and projection of axons to different regions (By similarity). SLIT1 and SLIT2 together seem to be essential for midline guidance in the forebrain by acting as repulsive signal preventing inappropriate midline crossing by axons projecting from the olfactory bulb
228	SNRPN	9606.ENSP00000382972	SNRPN	Small nuclear ribonucleoprotein-associated protein N; May be involved in tissue-specific alternative RNA processing events; Sm spliceosomal proteins
229	SNX1	9606.ENSP00000261889	SNX1	Sorting nexin-1; Involved in several stages of intracellular trafficking. Interacts with membranes containing phosphatidylinositol 3- phosphate (PtdIns(3P)) or phosphatidylinositol 3,5-bisphosphate (PtdIns(3,5)P2). Acts in part as component of the retromer membrane-deforming SNX-BAR subcomplex. The SNX-BAR retromer mediates retrograde transport of cargo proteins from endosomes to the trans-Golgi network (TGN) and is involved in endosome-to-plasma membrane transport for cargo protein recycling. The SNX-BAR subcomplex functions to deform the donor membrane into a tubular profile called e [...]
230	SOX2	9606.ENSP00000323588	SOX2	Transcription factor SOX-2; Transcription factor that forms a trimeric complex with OCT4 on DNA and controls the expression of a number of genes involved in embryonic development such as YES1, FGF4, UTF1 and ZFP206 (By similarity). Critical for early embryogenesis and for embryonic stem cell pluripotency. May function as a switch in neuronal development. Downstream SRRT target that mediates the promotion of neural stem cell self-renewal (By similarity). Keeps neural cells undifferentiated by counteracting the activity of proneural proteins and suppresses neuronal differentiation (By si [...]
231	SOX9	9606.ENSP00000245479	SOX9	Transcription factor SOX-9; Transcriptional regulator. Binds to the COL2A1 promoter and activates COL2A1 expression, as part of a complex with ZNF219 (By similarity). Plays a role in chondrocyte differentiation (By similarity). Important for normal skeletal development; SRY-boxes
232	SPNS2	9606.ENSP00000333292	SPNS2	Protein spinster homolog 2; Sphingolipid transporter required for migration of myocardial precursors. Transports sphingosine 1-phosphate (S1P), a secreted lipid mediator that plays critical roles in cardiovascular, immunological, and neural development and function. Mediates the export of S1P from cells in the extraembryonic yolk syncytial layer (YSL), thereby regulating myocardial precursor migration
233	SPTBN4	9606.ENSP00000263373	SPTBN4	Spectrin beta chain, non-erythrocytic 4; Pleckstrin homology domain containing
234	SRBD1	9606.ENSP00000263736	SRBD1	S1 RNA-binding domain-containing protein 1; S1 RNA binding domain 1
235	STARD3NL	9606.ENSP00000009041	STARD3NL	STARD3 N-terminal-like protein; Tethering protein that creates contact site between the endoplasmic reticulum and late endosomes: localizes to late endosome membranes and contacts the endoplasmic reticulum via interaction with VAPA and VAPB; Belongs to the STARD3 family
236	STK19	9606.ENSP00000364482	STK19	Serine/threonine-protein kinase 19; Seems to be a protein kinase. In vitro it can phosphorylate casein-alpha on serine and threonine residues and histones on serine residues
237	SULT6B1	9606.ENSP00000384950	SULT6B1	Sulfotransferase 6B1; Sulfotransferase that utilizes 3'-phospho-5'-adenylyl sulfate (PAPS) as sulfonate donor to catalyze the sulfate conjugation of thyroxine. Involved in the metabolism of thyroxine (By similarity); Sulfotransferases, cytosolic
238	SYBU	9606.ENSP00000407118	SYBU	Syntabulin; Part of a kinesin motor-adapter complex that is critical for the anterograde axonal transport of active zone components and contributes to activity-dependent presynaptic assembly during neuronal development
239	SYNE3	9606.ENSP00000334308	SYNE3	Nesprin-3; As a component of the LINC (LInker of Nucleoskeleton and Cytoskeleton) complex involved in the connection between the nuclear lamina and the cytoskeleton. The nucleocytoplasmic interactions established by the LINC complex play an important role in the transmission of mechanical forces across the nuclear envelope and in nuclear movement and positioning. Probable anchoring protein which tethers the nucleus to the cytoskeleton by binding PLEC which can associate with the intermediate filament system. Plays a role in the regulation of aortic epithelial cell morphology, and is re [...]
240	SYS1	9606.ENSP00000243918	SYS1	Protein SYS1 homolog; Involved in protein trafficking. May serve as a receptor for ARFRP1; Belongs to the SYS1 family
241	TAF4	9606.ENSP00000252996	TAF4	Transcription initiation factor TFIID subunit 4; Part of the TFIID complex, a multimeric protein complex that plays a central role in mediating promoter responses to various activators and repressors. Potentiates transcriptional activation by the AF-2S of the retinoic acid, vitamin D3 and thyroid hormone; Belongs to the TAF4 family
242	TBC1D31	9606.ENSP00000287380	TBC1D31	TBC1 domain family member 31; WD repeat domain containing
244	TFAP2A	9606.ENSP00000368924	TFAP2A	Transcription factor AP-2-alpha; Sequence-specific DNA-binding protein that interacts with inducible viral and cellular enhancer elements to regulate transcription of selected genes. AP-2 factors bind to the consensus sequence 5'-GCCNNNGGC-3' and activate genes involved in a large spectrum of important biological functions including proper eye, face, body wall, limb and neural tube development. They also suppress a number of genes including MCAM/MUC18, C/EBP alpha and MYC. AP-2-alpha is the only AP-2 protein required for early morphogenesis of the lens vesicle. Together with the CITED2 [...]
245	TFAP2E	9606.ENSP00000362332	TFAP2E	Transcription factor AP-2-epsilon; Sequence-specific DNA-binding protein that interacts with inducible viral and cellular enhancer elements to regulate transcription of selected genes. AP-2 factors bind to the consensus sequence 5'-GCCNNNGGC-3' and activate genes involved in a large spectrum of important biological functions including proper eye, face, body wall, limb and neural tube development. They also suppress a number of genes including MCAM/MUC18, C/EBP alpha and MYC. AP-2-epsilon may play a role in the development of the CNS and in cartilage differentiation (By similarity)
246	TGFBI	9606.ENSP00000416330	TGFBI	Transforming growth factor-beta-induced protein ig-h3; Plays a role in cell adhesion. May play a role in cell-collagen interactions (By similarity)
247	TIAM2	9606.ENSP00000437188	TIAM2	T-lymphoma invasion and metastasis-inducing protein 2; Modulates the activity of RHO-like proteins and connects extracellular signals to cytoskeletal activities. Acts as a GDP- dissociation stimulator protein that stimulates the GDP-GTP exchange activity of RHO-like GTPases and activates them. Mediates extracellular laminin signals to activate Rac1, contributing to neurite growth. Involved in lamellipodial formation and advancement of the growth cone of embryonic hippocampal neurons. Promotes migration of neurons in the cerebral cortex. When overexpressed, induces membrane ruffling acc [...]
248	TIE1	9606.ENSP00000361554	TIE1	Tyrosine-protein kinase receptor Tie-1; Transmembrane tyrosine-protein kinase that may modulate TEK/TIE2 activity and contribute to the regulation of angiogenesis
249	TLL1	9606.ENSP00000061240	TLL1	Tolloid-like protein 1; Protease which processes procollagen C-propeptides, such as chordin, pro-biglycan and pro-lysyl oxidase. Required for the embryonic development. Predominant protease, which in the development, influences dorsal-ventral patterning and skeletogenesis; Astacins

Depression (PHQ-9)

#	queryItem	stringId	preferredName	annotation
250	TMEM120B	9606.ENSP00000404991	TMEM120B	Transmembrane protein 120B; Necessary for efficient adipogenesis; Belongs to the TMEM120 family
251	TMEM52B	9606.ENSP00000371348	TMEM52B	Transmembrane protein 52B
252	TMIGD3	9606.ENSP00000358730	ADORA3	Transmembrane domain-containing protein TMIGD3; Isoform 1: Plays a suppressive role in osteosarcoma malignancy by inhibiting NF-kappa-B activity; Adenosine receptors
253	TMSB4X	9606.ENSP00000370010	TMSB4X	Thymosin beta-4; Plays an important role in the organization of the cytoskeleton (By similarity). Binds to and sequesters actin monomers (G actin) and therefore inhibits actin polymerization
254	TOM1L1	9606.ENSP00000460823	TOM1L1	TOM1-like protein 1; Probable adapter protein involved in signaling pathways. Interacts with the SH2 and SH3 domains of various signaling proteins when it is phosphorylated. May promote FYN activation, possibly by disrupting intramolecular SH3-dependent interactions (By similarity); Belongs to the TOM1 family
255	TOMM5	9606.ENSP00000438204	TOMM5	Mitochondrial import receptor subunit TOM5 homolog; Translocase of outer mitochondrial membrane complex
256	TP63	9606.ENSP00000264731	TP63	Tumor protein 63; Acts as a sequence specific DNA binding transcriptional activator or repressor. The isoforms contain a varying set of transactivation and auto-regulating transactivation inhibiting domains thus showing an isoform specific activity. Isoform 2 activates RIPK4 transcription. May be required in conjunction with TP73/p73 for initiation of p53/TP53 dependent apoptosis in response to genotoxic insults and the presence of activated oncogenes. Involved in Notch signaling by probably inducing JAG1 and JAG2. Plays a role in the regulation of epithelial morphogenesis. The ratio o [...]
257	TPTE2	9606.ENSP00000383089	TPTE2	Phosphatidylinositol 3,4,5-trisphosphate 3-phosphatase TPTE2; Transmembrane phosphoinositide 3-phosphatase and tensin homolog 2; C2 tensin-type domain containing
258	TRERF1	9606.ENSP00000439689	TRERF1	Transcriptional-regulating factor 1; Binds DNA and activates transcription of CYP11A1. Interaction with CREBBP and EP300 results in a synergistic transcriptional activation of CYP11A1; Myb/SANT domain containing
259	TRIM42	9606.ENSP00000286349	TRIM42	Tripartite motif-containing protein 42; Protein phosphatase 1 regulatory subunits; Belongs to the TRIM/RBCC family
260	TRIM46	9606.ENSP00000334657	TRIM46	Tripartite motif-containing protein 46; Microtubule-associated protein that is involved in the formation of parallel microtubule bundles linked by cross-bridges in the proximal axon. Required for the uniform orientation and maintenance of the parallel microtubule fascicles, which are important for efficient cargo delivery and trafficking in axons. Thereby also required for proper axon specification, the establishment of neuronal polarity and proper neuronal migration; Belongs to the TRIM/RBCC family
261	TRIM54	9606.ENSP00000296098	TRIM54	Tripartite motif containing 54; Ring finger proteins
262	TRPV4	9606.ENSP00000406191	TRPV4	Transient receptor potential cation channel subfamily V member 4; Non-selective calcium permeant cation channel involved in osmotic sensitivity and mechanosensitivity. Activation by exposure to hypotonicity within the physiological range exhibits an outward rectification. Also activated by heat, low pH, citrate and phorbol esters. Increase of intracellular Ca(2+) potentiates currents. Channel activity seems to be regulated by a calmodulin-dependent mechanism with a negative feedback mechanism. Promotes cell-cell junction formation in skin keratinocytes and plays an important role in th [...]
263	TSC1	9606.ENSP00000298552	TSC1	Hamartin; In complex with TSC2, inhibits the nutrient-mediated or growth factor-stimulated phosphorylation of S6K1 and EIF4EBP1 by negatively regulating mTORC1 signaling. Seems not to be required for TSC2 GAP activity towards RHEB. Implicated as a tumor suppressor. Involved in microtubule-mediated protein transport, but this seems to be due to unregulated mTOR signaling; Armadillo-like helical domain containing
264	TSPYL6	9606.ENSP00000417919	TSPYL6	Testis-specific Y-encoded-like protein 6; TSPY like 6
265	TTLL11	9606.ENSP00000321346	TTLL11	Tubulin polyglutamylase TTLL11; Polyglutamase which preferentially modifies alpha- tubulin. Involved in the side-chain elongation step of the polyglutamylation reaction rather than in the initiation step (By similarity). Required for CCSAP localization to both spindle and cilia microtubules. Generates long side-chains (By similarity); Belongs to the tubulin--tyrosine ligase family
266	TTLL7	9606.ENSP00000260505	TTLL7	Tubulin polyglutamylase TTLL7; Polyglutamylase which preferentially modifies beta- tubulin. Mediates both ATP-dependent initiation and elongation of polyglutamylation of microtubules. Required for neurite growth; responsible for the strong increase in tubulin polyglutamylation during postnatal neuronal maturation (By similarity); Belongs to the tubulin--tyrosine ligase family
267	USP31	9606.ENSP00000219689	USP31	Ubiquitin carboxyl-terminal hydrolase 31; May recognize and hydrolyze the peptide bond at the C- terminal Gly of ubiquitin. Involved in the processing of poly-ubiquitin precursors as well as that of ubiquitinated proteins (By similarity); Belongs to the peptidase C19 family
268	VAV2	9606.ENSP00000360916	VAV2	Guanine nucleotide exchange factor VAV2; Guanine nucleotide exchange factor for the Rho family of Ras-related GTPases. Plays an important role in angiogenesis. Its recruitment by phosphorylated EPHA2 is critical for EFNA1-induced RAC1 GTPase activation and vascular endothelial cell migration and assembly (By similarity); Pleckstrin homology domain containing
269	VWA5B1	9606.ENSP00000364220	VWA5B1	Von Willebrand factor A domain containing 5B1
270	WAS	9606.ENSP00000365891	WAS	Wiskott-Aldrich syndrome protein; Effector protein for Rho-type GTPases. Regulates actin filament reorganization via its interaction with the Arp2/3 complex. Important for efficient actin polymerization. Possible regulator of lymphocyte and platelet function. Mediates actin filament reorganization and the formation of actin pedestals upon infection by pathogenic bacteria; Wiskott-Aldrich Syndrome protein family
271	WDR45	9606.ENSP00000348848	WDR45	WD repeat domain phosphoinositide-interacting protein 4; Plays an important role in the autophagy pathway, which is the major intracellular degradation system by which cytoplasmic materials are packaged into autophagosomes and delivered to lysosomes for degradation; Belongs to the WD repeat SVP1 family
272	WDR59	9606.ENSP00000262144	WDR59	GATOR complex protein WDR59; As a component of the GATOR subcomplex GATOR2, functions within the amino acid-sensing branch of the TORC1 signaling pathway. Indirectly activates mTORC1 and the TORC1 signaling pathway through the inhibition of the GATOR1 subcomplex. It is negatively regulated by the upstream amino acid sensors SESN2 and CASTOR1; Belongs to the WD repeat WDR59 family
273	WFIKKN2	9606.ENSP00000311184	WFIKKN2	WAP, Kazal, immunoglobulin, Kunitz and NTR domain-containing protein 2; Protease-inhibitor that contains multiple distinct protease inhibitor domains. Probably has serine protease- and metalloprotease-inhibitor activity. Inhibits the biological activity of mature myostatin, but not activin (By similarity); Belongs to the WFIKKN family
274	WSCD1	9606.ENSP00000460825	WSCD1	WSC domain containing 1
275	WWP2	9606.ENSP00000352069	WWP2	NEDD4-like E3 ubiquitin-protein ligase WWP2; E3 ubiquitin-protein ligase which accepts ubiquitin from an E2 ubiquitin-conjugating enzyme in the form of a thioester and then directly transfers the ubiquitin to targeted substrates. Polyubiquitinates POU5F1 by 'Lys-63'-linked conjugation and promotes it to proteasomal degradation; in embryonic stem cells (ESCs) the ubiquitination is proposed to regulate POU5F1 protein level. Ubiquitinates EGR2 and promotes it to proteasomal degradation; in T-cells the ubiquitination inhibits activation- induced cell death. Ubiquitinates SLC11A2; the ubiqu [...]
276	XAGE5	9606.ENSP00000342240	XAGE5	X antigen family member 5; Belongs to the GAGE family
277	XKR3	9606.ENSP00000331704	XKR3	XK-related protein 3; XK related family; Belongs to the XK family
278	YPEL2	9606.ENSP00000312272	YPEL2	Protein yippee-like 2; Yippee like family
279	ZMYND8	9606.ENSP00000420095	ZMYND8	Protein kinase C-binding protein 1; May act as a transcriptional corepressor for KDM5D. Required for KDM5D-mediated down-regulation of diverse metastasis-associated genes; the function seems to involve the recognition of the dual histone signature H3K4me1-H3K14ac. Suppresses prostate cancer cell invasion; Bromodomain containing
280	ZNF233	9606.ENSP00000375820	ZNF233	Zinc finger protein 233; May be involved in transcriptional regulation; Zinc fingers C2H2-type
281	ZNF331	9606.ENSP00000253144	ZNF331	Zinc finger protein 331; May be involved in transcriptional regulation. May play a role in spermatogenesis; Zinc fingers C2H2-type
282	ZNF423	9606.ENSP00000455426	ZNF423	Zinc finger protein 423; Transcription factor that can both act as an activator or a repressor depending on the context. Plays a central role in BMP signaling and olfactory neurogenesis. Associates with SMADs in response to BMP2 leading to activate transcription of BMP target genes. Acts as a transcriptional repressor via its interaction with EBF1, a transcription factor involved in terminal olfactory receptor neurons differentiation; this interaction preventing EBF1 to bind DNA and activate olfactory-specific genes. Involved in olfactory neurogenesis by participating in a developmenta [...]
283	ZNF449	9606.ENSP00000339585	ZNF449	Zinc finger protein 449; May be involved in transcriptional regulation; Belongs to the krueppel C2H2-type zinc-finger protein family
284	ZNF503	9606.ENSP00000361602	ZNF503	Zinc finger protein 503; May function as a transcriptional repressor; Zinc fingers C2H2-type
285	ZNF536	9606.ENSP00000347730	ZNF536	Zinc finger protein 536; May be involved in transcriptional regulation. Recognizes and binds 2 copies of the core DNA sequence 5'-CCCCCA- 3'; Zinc fingers C2H2-type
286	ZNF710	9606.ENSP00000268154	ZNF710	Zinc finger protein 710; May be involved in transcriptional regulation; Zinc fingers C2H2-type
287	ZNF77	9606.ENSP00000319053	ZNF77	Zinc finger protein 77; May be involved in transcriptional regulation; Zinc fingers C2H2-type

Association with Appetitive Aggression

Gene list

ABCC11, ACP6, ACSL5, ACTN2, ADAMDEC1, ADGRD1, ADGRG6, ADK, ADRA1A, ADSS2, ADTRP, AKR1C3, ALOX5, AMACR, ANK3, ANKDD1A, ANKRD12, ARHGAP26, ARID5B, ARMC3, ATP1A4, ATXN1L, ATXN2, B3GLCT, BCOR, BLVRA, BOC, C1orf174, C1orf61, C1orf94, C20orf85, C3orf52, C6orf99, CAMKK1, CAPSL, CBLIF, CBLN1, CBR1, CCDC113, CCDC170, CCL3, CCNQ, CCNYL3, CD200R1, CD58, CDRT4, CELF2, CFAP46, CFAP77, CHD5, CHST10, CLUL1, CMTM2, COG1, COL22A1, CREB3, CTNNBL1, CUBN, DAGLB, DAOA, DCDC2C, DCTD, DDX51, DENND2B, DEUP1, DIAPH2, DIP2C, DIXDC1, DLGAP2, DLK1, DMBT1, DMD, DNAJA2, DNAJB11, DNHD1, DPF3, DUSP1, DUSP4, EDEM1, ELFN1, ELMO2, EN1, EPAS1, EPHA4, FAM160A2, FAM207A, FAM72C, FBN1, FBRSL1, FGF13, FIBCD1, FIGNL2, FKBP6, FOXD2, FOXP1, FRG1, FZD10, GALNT9, GCNT2, GIGYF2, GK, GLOD5, GLRX3, GLTP, GMDS, GPRASP2, GPX3, GPX5, HDAC4-AS1, HERPUD2, HMGB4, HNRNPA3P6, HS3ST3B1, IGFL4, IKZF4, IL17D, IL9, ILDR2, INSC, IQSEC2, IRX1, ITGB8, ITPRID1, JADE1, JAM3, KAT6B, KCNA4, KCNMA1, KIF7, KIRREL2, KLF6, KRT33A, KTN1, LAMA2, LAMA4, LAMC2, LGI4, LMX1B, LRATD1, LRP1, LRP8, LY6K, LZTS1, LZTS3, MAML1, MAX, MBOAT1, MBP, MCTS1, MEG9, MEIS2, METTL4, MEX3C, MFAP3L, MIR3937, MOB1A, MRPL23, MSI2, MSL3, MTCL1, MTERF, MX2, MYO18A, MYO7A, NCAM1, NCKAP5, NEBL-AS1, NKAPL, NMT2, NPAS2, NTM, NUFIP1, ONECUT1, OR5AU1, OSBP, OTX2, PCAT29, PCDH7, PCDH9, PCDHA1, PCDHA6, PCDHB16, PCDHGA2, PCDHGA4, PCDHGA5, PDE2A, PDXK, PEX12, PFKP, PGBD1, PGRMC1, PITX1, PLA2G12B, PLET1, PMEPA1, PNKD, PPARGC1A, PPP2R5C, PPT1, PRDM5, PRKCH, PRMT2, PTPRT, PTPRU, PXDN, PXDNL, RARA, RBFOX3, RGN, RHOBTB2, RIC8B, RIPK4, RNF125, RNF182, ROR1, RPH3A, RRP15, RTN4, RTP5, RUBCN, RUNX1, SDC3, SDR42E2, SEMA3D, SEMA3F, SEMA4A, SEMA6D, SERP2, SH3BGRL, SHISA2, SLC28A2, SLC39A4, SLC5A1, SLC6A14, SLC8A2, SMIM41, SMPD3, SNX12, SORCS1, SOX9, SP9, SPATA5, SPDL1, SPNS2, SRGAP3, SSBP3, ST3GAL1, STUM, SUCLG1, SULT2B1, SYTL4, TAF7, TBX1, TCEAL7, TCF3, TENT5D, THAP1, TLCD5, TLX1, TMEM154, TMEM200A, TMEM229B, TNFSF4, TP73, TPO, TRIB1, TRIM36, TRPC7, TRPM5, TSNAX, TTC27, TTC7B, TVP23C, UACA, UBE2F, UNC5CL, UPK1B, USP12, USP24, UTS2R, VAX2, VRK1, VSIG1, WAPL, WDPCP, WDR27, WDR81, WT1, XXYLT1, YTHDF2, ZC3H12B, ZC3H18, ZEB2, ZNF696, ZNF763

Network

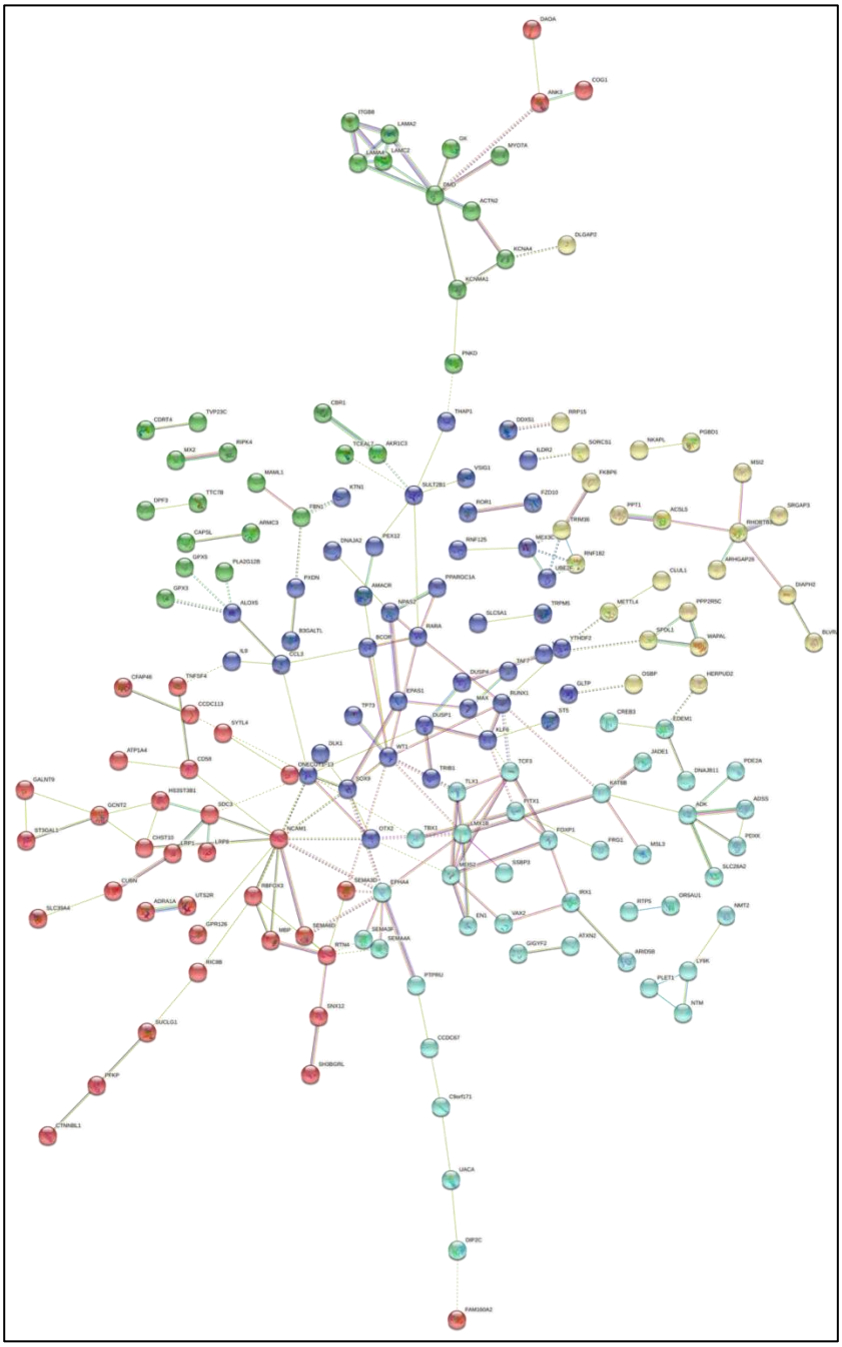

Appetitive aggression (AAS)

Enrichments found

Network Stats

number of nodes:	281	expected number of edges:	165
number of edges:	203	PPI enrichment p-value:	0.00227
average node degree:	1.44		
avg. local clustering coefficient:	0.348		

your network has significantly more interactions than expected (what does that mean?)

Enrichment Table Columns

Count In Network:
The first number indicates how many proteins in your network are annotated with a particular term. The second number indicates how many proteins in total (in your network and in the background) have this term assigned.

Strength:
Log10(observed / expected). This measure describes how large the enrichment effect is. It's the ratio between i) the number of proteins in your network that are annotated with a term and ii) the number of proteins that we expect to be annotated with this term in a random network of the same size.

False Discovery Rate:
This measure describes how significant the enrichment is. Shown are p-values corrected for multiple testing within each category using the Benjamini–Hochberg procedure.

Functional enrichments in your network

explain columns

Biological Process (Gene Ontology)

GO-term	description	count in network	strength	false discovery rate
GO:0007417	central nervous system development	28 of 861	0.35	0.0298
GO:0007155	cell adhesion	27 of 843	0.35	0.0463
GO:0048468	cell development	42 of 1493	0.29	0.0280
GO:0007399	nervous system development	60 of 2206	0.28	0.0044
GO:0030154	cell differentiation	78 of 3457	0.2	0.0280
GO:0048856	anatomical structure development	107 of 5085	0.17	0.0135
GO:0048731	system development	88 of 4144	0.17	0.0280
GO:0007275	multicellular organism development	98 of 4726	0.16	0.0280
GO:0032502	developmental process	110 of 5401	0.15	0.0280

(less ...)

local network cluster (STRING)

cluster	description	count in network	strength	false discovery rate
CL:20970	mixed, incl. NACHT domain binding, and RNA-binding protein M...	3 of 5	1.62	0.0378
CL:17749	mixed, incl. L-fuconate dehydratase, and DNA methylation on a...	3 of 7	1.47	0.0445
CL:619	mixed, incl. Neural cell adhesion, and Dullard phosphatase dom...	4 of 19	1.17	0.0445
CL:618	mixed, incl. Axonal growth inhibition (RHOA activation), and Ne...	5 of 28	1.09	0.0318
CL:32171	mixed, incl. Cadherin-like, and Domain in the Doublecortin (DCX...	8 of 74	0.88	0.0107
CL:615	mixed, incl. Axonal growth inhibition (RHOA activation), and Pho...	6 of 57	0.87	0.0445
CL:32170	mixed, incl. Cadherin-like, and Transmembrane protein 132	10 of 104	0.83	0.0052

(less ...)

Annotated Keywords (UniProt)

keyword	description	count in network	strength	false discovery rate
KW-0376	Hydrogen peroxide	3 of 9	1.37	0.0273
KW-0575	Peroxidase	5 of 25	1.14	0.0085
KW-0130	Cell adhesion	18 of 476	0.42	0.0195
KW-0393	Immunoglobulin domain	17 of 448	0.42	0.0212
KW-0106	Calcium	27 of 872	0.33	0.0195
KW-0025	Alternative splicing	181 of 10225	0.09	0.0084

(less ...)

Protein Domains (Pfam)

domain	description	count in network	strength	false discovery rate
PF08266	Cadherin-like	8 of 63	0.95	0.0028

Protein Domains and Features (InterPro)

domain	description	count in network	strength	false discovery rate
IPR013164	Cadherin, N-terminal	8 of 63	0.95	0.0050

Appetitive aggression (AAS)

Mapping

#	queryItem	stringId	preferredName	annotation
1	ABCC11	9606.ENSP00000378230	ABCC11	ATP-binding cassette sub-family C member 11; Participates in physiological processes involving bile acids, conjugated steroids and cyclic nucleotides. Enhances the cellular extrusion of cAMP and cGMP. Stimulates the ATP-dependent uptake of a range of physiological and synthetic lipophilic anions, including the glutathione S- conjugates leukotriene C4 and dinitrophenyl S-glutathione, steroid sulfates such as dehydroepiandrosterone 3-sulfate (DHEAS) and estrone 3-sulfate, glucuronides such as estradiol 17-beta-D- glucuronide (E(2)17betaG), the monoanionic bile acids glycocholate and taur [...]
2	ACP6	9606.ENSP00000463574	ACP6	Lysophosphatidic acid phosphatase type 6; Hydrolyzes lysophosphatidic acid (LPA) containing a medium length fatty acid chain to the corresponding monoacylglycerol. Has highest activity with lysophosphatidic acid containing myristate (C14:0), monounsaturated oleate (C18:1) or palmitate (C16:0), and lower activity with C18:0 and C6:0 lysophosphatidic acid
3	ACSL5	9606.ENSP00000348429	ACSL5	Long-chain-fatty-acid--CoA ligase 5; Acyl-CoA synthetases (ACSL) activate long-chain fatty acids for both synthesis of cellular lipids, and degradation via beta-oxidation. ACSL5 may activate fatty acids from exogenous sources for the synthesis of triacylglycerol destined for intracellular storage (By similarity). Utilizes a wide range of saturated fatty acids with a preference for C16-C18 unsaturated fatty acids (By similarity). It was suggested that it may also stimulate fatty acid oxidation (By similarity). At the villus tip of the crypt-villus axis of the small intestine may sensiti [...]
4	ACTN2	9606.ENSP00000443495	ACTN2	Alpha-actinin-2; F-actin cross-linking protein which is thought to anchor actin to a variety of intracellular structures. This is a bundling protein; Actinins
5	ADAMDEC1	9606.ENSP00000256412	ADAMDEC1	ADAM DEC1; May play an important role in the control of the immune response and during pregnancy
6	ADGRD1	9606.ENSP00000261654	GPR133	Adhesion G-protein coupled receptor D1; Orphan receptor. Signals via G(s)-alpha family of G- proteins. Has protumorigenic function especially in glioblastoma
7	ADGRG6	9606.ENSP00000356581	GPR126	Adhesion G-protein coupled receptor G6; G-protein coupled receptor which is activated by type IV collagen, a major constituent of the basement membrane (By similarity). Couples to G(i)-proteins as well as G(s)-proteins. Essential for normal differentiation of promyelinating Schwann cells and for normal myelination of axons. Regulates neural, cardiac and ear development via G-protein- and/or N-terminus-dependent signaling (By similarity). May act as a receptor for PRNP which may promote myelin homeostasis (By similarity)
8	ADK	9606.ENSP00000286621	ADK	Adenosine kinase; ATP dependent phosphorylation of adenosine and other related nucleoside analogs to monophosphate derivatives. Serves as a potential regulator of concentrations of extracellular adenosine and intracellular adenine nucleotides; Belongs to the carbohydrate kinase PfkB family
9	ADRA1A	9606.ENSP00000369960	ADRA1A	Alpha-1A adrenergic receptor; This alpha-adrenergic receptor mediates its action by association with G proteins that activate a phosphatidylinositol- calcium second messenger system. Its effect is mediated by G(q) and G(11) proteins. Nuclear ADRA1A-ADRA1B heterooligomers regulate phenylephrine(PE)-stimulated ERK signaling in cardiac myocytes; Belongs to the G-protein coupled receptor 1 family. Adrenergic receptor subfamily. ADRA1A sub-subfamily
10	ADSS2	9606.ENSP00000355493	ADSS	Adenylosuccinate synthetase isozyme 2; Plays an important role in the de novo pathway and in the salvage pathway of purine nucleotide biosynthesis. Catalyzes the first committed step in the biosynthesis of AMP from IMP
11	ADTRP	9606.ENSP00000229583	ADTRP	Androgen-dependent TFPI-regulating protein; Regulates the expression and the cell-associated anticoagulant activity of the inhibitor TFPI in endothelial cells (in vitro)
12	AKR1C3	9606.ENSP00000369927	AKR1C3	Aldo-keto reductase family 1 member C3; Catalyzes the conversion of aldehydes and ketones to alcohols. Catalyzes the reduction of prostaglandin (PG) D2, PGH2 and phenanthrenequinone (PQ) and the oxidation of 9-alpha,11-beta- PGF2 to PGD2. Functions as a bi-directional 3-alpha-, 17-beta- and 20-alpha HSD. Can interconvert active androgens, estrogens and progestins with their cognate inactive metabolites. Preferentially transforms androstenedione (4-dione) to testosterone; Belongs to the aldo/keto reductase family
13	ALOX5	9606.ENSP00000363512	ALOX5	Arachidonate 5-lipoxygenase; Catalyzes the first step in leukotriene biosynthesis, and thereby plays a role in inflammatory processes; Belongs to the lipoxygenase family
14	AMACR	9606.ENSP00000371517	AMACR	Alpha-methylacyl-CoA racemase; Racemization of 2-methyl-branched fatty acid CoA esters. Responsible for the conversion of pristanoyl-CoA and C27-bile acyl-CoAs to their (S)-stereoisomers; Belongs to the CaiB/BaiF CoA-transferase family
15	ANK3	9606.ENSP00000280772	ANK3	Ankyrin-3; In skeletal muscle, required for costamere localization of DMD and betaDAG1 (By similarity). Membrane-cytoskeleton linker. May participate in the maintenance/targeting of ion channels and cell adhesion molecules at the nodes of Ranvier and axonal initial segments. Regulates KCNA1 channel activity in function of dietary Mg(2+) levels, and thereby contributes to the regulation of renal Mg(2+) reabsorption; Ankyrin repeat domain containing
16	ANKDD1A	9606.ENSP00000325895	ANKDD1A	Ankyrin repeat and death domain containing 1A
17	ANKRD12	9606.ENSP00000262126	ANKRD12	Ankyrin repeat domain-containing protein 12; May recruit HDACs to the p160 coactivators/nuclear receptor complex to inhibit ligand-dependent transactivation; Ankyrin repeat domain containing
18	ARHGAP26	9606.ENSP00000274498	ARHGAP26	Rho GTPase-activating protein 26; GTPase-activating protein for RHOA and CDC42
19	ARID5B	9606.ENSP00000279873	ARID5B	AT-rich interactive domain-containing protein 5B; Transcription coactivator that binds to the 5'-AATA[CT]- 3' core sequence and plays a key role in adipogenesis and liver development. Acts by forming a complex with phosphorylated PHF2, which mediates demethylation at Lys-336, leading to target the PHF2-ARID5B complex to target promoters, where PHF2 mediates demethylation of dimethylated 'Lys-9' of histone H3 (H3K9me2), followed by transcription activation of target genes. The PHF2- ARID5B complex acts as a coactivator of HNF4A in liver. Required for adipogenesis: regulates triglyceride [...]
20	ARMC3	9606.ENSP00000298032	ARMC3	Armadillo repeat containing 3
21	ATP1A4	9606.ENSP00000357060	ATP1A4	Sodium/potassium-transporting ATPase subunit alpha-4; This is the catalytic component of the active enzyme, which catalyzes the hydrolysis of ATP coupled with the exchange of sodium and potassium ions across the plasma membrane. This action creates the electrochemical gradient of sodium and potassium ions, providing the energy for active transport of various nutrients. Plays a role in sperm motility; ATPase Na+/K+ transporting subunits
22	ATXN1L	9606.ENSP00000415822	ATXN1L	Ataxin-1-like; Chromatin-binding factor that repress Notch signaling in the absence of Notch intracellular domain by acting as a CBF1 corepressor. Binds to the HEY promoter and might assist, along with NCOR2, RBPJ-mediated repression. Can suppress ATXN1 cytotoxicity in spinocerebellar ataxia type 1 (SCA1). In concert with CIC and ATXN1, involved in brain development (By similarity)
23	ATXN2	9606.ENSP00000366843	ATXN2	Ataxin-2; Involved in EGFR trafficking, acting as negative regulator of endocytic EGFR internalization at the plasma membrane; Ataxins
24	B3GLCT	9606.ENSP00000343002	B3GALTL	Beta-1,3-glucosyltransferase; O-glucosyltransferase that transfers glucose toward fucose with a beta-1,3 linkage. Specifically glucosylates O-linked fucosylglycan on TSP type-1 domains of proteins, thereby contributing to elongation of O-fucosylglycan
25	BCOR	9606.ENSP00000367705	BCOR	BCL-6 corepressor; Transcriptional corepressor. May specifically inhibit gene expression when recruited to promoter regions by sequence-specific DNA-binding proteins such as BCL6 and MLLT3. This repression may be mediated at least in part by histone deacetylase activities which can associate with this corepressor. Involved in the repression of TFAP2A; impairs binding of BCL6 and KDM2B to TFAP2A promoter regions. Via repression of TFAP2A acts as a negative regulator of osteo-dentiogenic capacity in adult stem cells; the function implies inhibition of methylation on histone H3 'Lys-4' ([...]
26	BLVRA	9606.ENSP00000385757	BLVRA	Biliverdin reductase A; Reduces the gamma-methene bridge of the open tetrapyrrole, biliverdin IX alpha, to bilirubin with the concomitant oxidation of a NADH or NADPH cofactor; Belongs to the Gfo/Idh/MocA family. Biliverdin reductase subfamily
27	BOC	9606.ENSP00000418663	BOC	Brother of CDO; Component of a cell-surface receptor complex that mediates cell-cell interactions between muscle precursor cells. Promotes differentiation of myogenic cells; Fibronectin type III domain containing
28	C1orf174	9606.ENSP00000355306	C1orf174	UPF0688 protein C1orf174; Chromosome 1 open reading frame 174; Belongs to the UPF0688 family
29	C1orf61	9606.ENSP00000357226	C1orf61	Protein CROC-4; May play a role in FOS signaling pathways involved in development and remodeling of neurons. Promotes transcription of the FOS promoter
30	C1orf94	9606.ENSP00000435634	C1orf94	Uncharacterized protein C1orf94; Chromosome 1 open reading frame 94
31	C20orf85	9606.ENSP00000360210	C20orf85	Uncharacterized protein C20orf85; Chromosome 20 open reading frame 85
32	C3orf52	9606.ENSP00000399392	C3orf52	TPA-induced transmembrane protein; Chromosome 3 open reading frame 52
33	C6orf99	9606.ENSP00000476986	C6orf99	Putative uncharacterized protein C6orf99; Chromosome 6 open reading frame 99
34	CAMKK1	9606.ENSP00000158166	CAMKK1	Calcium/calmodulin-dependent protein kinase kinase 1; Calcium/calmodulin-dependent protein kinase that belongs to a proposed calcium-triggered signaling cascade involved in a number of cellular processes. Phosphorylates CAMK1, CAMK1D, CAMK1G and CAMK4. Involved in regulating cell apoptosis. Promotes cell survival by phosphorylating AKT1/PKB that inhibits pro-apoptotic BAD/Bcl2-antagonist of cell death
35	CAPSL	9606.ENSP00000380524	CAPSL	Calcyphosin-like protein; EF-hand domain containing
37	CBLN1	9606.ENSP00000219197	CBLN1	Cerebellin-1; Required for synapse integrity and synaptic plasticity. During cerebellar synapse formation, essential for the matching and maintenance of pre- and post-synaptic elements at parallel fiber-Purkinje cell synapses, the establishment of the proper pattern of climbing fiber-Purkinje cell innervation, and induction of long-term depression at parallel fiber-Purkinje cell synapses. Plays a role as a synaptic organizer that acts bidirectionally on both pre- and post-synaptic components. On the one hand induces accumulation of synaptic vesicles in the pre-synaptic part by binding [...]
38	CBR1	9606.ENSP00000290349	CBR1	Carbonyl reductase [NADPH] 1; NADPH-dependent reductase with broad substrate specificity. Catalyzes the reduction of a wide variety of carbonyl compounds including quinones, prostaglandins, menadione, plus various xenobiotics. Catalyzes the reduction of the antitumor anthracyclines doxorubicin and daunorubicin to the cardiotoxic compounds doxorubicinol and daunorubicinol. Can convert prostaglandin E2 to prostaglandin F2-alpha. Can bind glutathione, which explains its higher affinity for glutathione-conjugated substrates. Catalyzes the reduction of S-nitrosoglutathione; Short chain dehy [...]
39	CCDC113	9606.ENSP00000219299	CCDC113	Coiled-coil domain-containing protein 113; Component of centriolar satellites contributing to primary cilium formation
40	CCDC170	9606.ENSP00000239374	CCDC170	Coiled-coil domain containing 170
41	CCL3	9606.ENSP00000477908	CCL3	C-C motif chemokine 3; Monokine with inflammatory and chemokinetic properties. Binds to CCR1, CCR4 and CCR5. One of the major HIV-suppressive factors produced by CD8+ T-cells. Recombinant MIP-1-alpha induces a dose-dependent inhibition of different strains of HIV-1, HIV-2, and simian immunodeficiency virus (SIV); Belongs to the intercrine beta (chemokine CC) family
42	CCNQ	9606.ENSP00000402949	FAM58A	Cyclin-Q; Activating cyclin for the cyclin-associated kinase CDK10; Belongs to the cyclin family. Cyclin-like FAM58 subfamily
44	CD200R1	9606.ENSP00000311035	CD200R1	Cell surface glycoprotein CD200 receptor 1; Inhibitory receptor for the CD200/OX2 cell surface glycoprotein. Limits inflammation by inhibiting the expression of proinflammatory molecules including TNF-alpha, interferons, and inducible nitric oxide synthase (iNOS) in response to selected stimuli. Also binds to HHV-8 K14 viral CD200 homolog with identical affinity and kinetics as the host CD200; Belongs to the CD200R family
45	CD58	9606.ENSP00000358501	CD58	Lymphocyte function-associated antigen 3; Ligand of the T-lymphocyte CD2 glycoprotein. This interaction is important in mediating thymocyte interactions with thymic epithelial cells, antigen-independent and -dependent interactions of T-lymphocytes with target cells and antigen- presenting cells and the T-lymphocyte rosetting with erythrocytes. In addition, the LFA-3/CD2 interaction may prime response by both the CD2+ and LFA-3+ cells
46	CDRT4	9606.ENSP00000482523	CDRT4	CMT1A duplicated region transcript 4
47	CELF2	9606.ENSP00000443926	CELF2	CUGBP Elav-like family member 2; RNA-binding protein implicated in the regulation of several post-transcriptional events. Involved in pre-mRNA alternative splicing, mRNA translation and stability. Mediates exon inclusion and/or exclusion in pre-mRNA that are subject to tissue-specific and developmentally regulated alternative splicing. Specifically activates exon 5 inclusion of TNNT2 in embryonic, but not adult, skeletal muscle. Activates TNNT2 exon 5 inclusion by antagonizing the repressive effect of PTB. Acts as both an activator and repressor of a pair of coregulated exons: promotes [...]
48	CFAP46	9606.ENSP00000357575	CFAP46	Cilia- and flagella-associated protein 46; As part of the central apparatus of the cilium axoneme plays a role in cilium movement; Cilia and flagella associated
49	CFAP77	9606.ENSP00000343290	C9orf171	Cilia- and flagella-associated protein 77; Chromosome 9 open reading frame 171
50	CHD5	9606.ENSP00000262450	CHD5	Chromodomain-helicase-DNA-binding protein 5; Chromatin-remodeling protein that binds DNA through histones and regulates gene transcription. May specifically recognize and bind trimethylated 'Lys-27' (H3K27me3) and non- methylated 'Lys-4' of histone H3. Plays a role in the development of the nervous system by activating the expression of genes promoting neuron terminal differentiation. In parallel, it may

#	queryItem	stringId	preferredName	annotation
				also positively regulate the trimethylation of histone H3 at 'Lys- 27' thereby specifically repressing genes that promote the differentiation into non-neuronal cell lineages. Tumor sup [...]
51	CHST10	9606.ENSP00000264249	CHST10	Carbohydrate sulfotransferase 10; Catalyzes the transfer of sulfate to position 3 of terminal glucuronic acid of both protein- and lipid-linked oligosaccharides. Participates in biosynthesis of HNK-1 carbohydrate structure, a sulfated glucuronyl-lactosaminyl residue carried by many neural recognition molecules, which is involved in cell interactions during ontogenetic development and in synaptic plasticity in the adult. May be indirectly involved in synapse plasticity of the hippocampus, via its role in HNK-1 biosynthesis; Sulfotransferases, membrane bound
52	CLUL1	9606.ENSP00000441726	CLUL1	Clusterin-like protein 1; Clusterin like 1
53	CMTM2	9606.ENSP00000268595	CMTM2	CKLF like MARVEL transmembrane domain containing 2; Belongs to the chemokine-like factor family
54	COG1	9606.ENSP00000299886	COG1	Conserved oligomeric Golgi complex subunit 1; Required for normal Golgi function; Components of oligomeric golgi complex
55	COL22A1	9606.ENSP00000303153	COL22A1	Collagen alpha-1(XXII) chain; Acts as a cell adhesion ligand for skin epithelial cells and fibroblasts; Belongs to the fibril-associated collagens with interrupted helices (FACIT) family
56	CREB3	9606.ENSP00000342136	CREB3	Cyclic AMP-responsive element-binding protein 3; Endoplasmic reticulum (ER)-bound transcription factor that plays a role in the unfolded protein response (UPR). Involved in cell proliferation and migration, tumor suppression and inflammatory gene expression. Plays also a role in the human immunodeficiency virus type 1 (HIV-1) virus protein expression and in the herpes simplex virus-1 (HSV-1) latent infection and reactivation from latency. Isoform 2 plays a role in the unfolded protein response (UPR). Isoform 2 acts as a positive regulator of LKN-1/CCL15-induced chemotaxis signaling of [...]
57	CTNNBL1	9606.ENSP00000355050	CTNNBL1	Beta-catenin-like protein 1; Component of the PRP19-CDC5L complex that forms an integral part of the spliceosome and is required for activating pre-mRNA splicing. Participates in AID/AICDA-mediated Ig class switching recombination (CSR). May induce apoptosis; Armadillo-like helical domain containing
58	CUBN	9606.ENSP00000367064	CUBN	Cubilin; Cotransporter which plays a role in lipoprotein, vitamin and iron metabolism, by facilitating their uptake. Binds to ALB, MB, Kappa and lambda-light chains, TF, hemoglobin, GC, SCGB1A1, APOA1, high density lipoprotein, and the GIF-cobalamin complex. The binding of all ligands requires calcium. Serves as important transporter in several absorptive epithelia, including intestine, renal proximal tubules and embryonic yolk sac. Interaction with LRP2 mediates its trafficking throughout vesicles and facilitates the uptake of specific ligands like GC, hemoglobin, ALB, TF and SCGB1A1. [...]
59	DAGLB	9606.ENSP00000297056	DAGLB	Sn1-specific diacylglycerol lipase beta; Catalyzes the hydrolysis of diacylglycerol (DAG) to 2- arachidonoyl-glycerol (2-AG), the most abundant endocannabinoid in tissues. Required for axonal growth during development and for retrograde synaptic signaling at mature synapses; Belongs to the AB hydrolase superfamily. Lipase family
60	DAOA	9606.ENSP00000483757	DAOA	D-amino acid oxidase activator; Seems to activate D-amino acid oxidase
61	DCDC2C	9606.ENSP00000382097	DCDC2C	Doublecortin domain containing 2C
62	DCTD	9606.ENSP00000349576	DCTD	Deoxycytidylate deaminase; Supplies the nucleotide substrate for thymidylate synthetase
63	DDX51	9606.ENSP00000380495	DDX51	ATP-dependent RNA helicase DDX51; ATP-binding RNA helicase involved in the biogenesis of 60S ribosomal subunits; Belongs to the DEAD box helicase family. DDX51/DBP6 subfamily
64	DENND2B	9606.ENSP00000433528	ST5	Suppression of tumorigenicity 5 protein; Guanine nucleotide exchange factor (GEF) which may activate RAB9A and RAB9B. Promotes the exchange of GDP to GTP, converting inactive GDP-bound Rab proteins into their active GTP- bound form. May be involved in cytoskeletal organization and tumorogenicity. Isoform 1 seems to be involved in a signaling transduction pathway leading to activation of MAPK1/ERK2. Isoform 3 may block ERK2 activation stimulated by ABL1. Isoform 3 may alter cell morphology and cell growth; DENN/MADD domain containing
65	DEUP1	9606.ENSP00000298050	CCDC67	Deuterosome assembly protein 1; Key structural component of the deuterosome, a structure that promotes de novo centriole amplification in multiciliated cells. Deuterosome-mediated centriole amplification occurs in terminally differentiated multiciliated cells and can generate more than 100 centrioles. Probably sufficient for the specification and formation of the deuterosome inner core. Interacts with CEP152 and recruits PLK4 to activate centriole biogenesis (By similarity); Belongs to the CEP63 family
66	DIAPH2	9606.ENSP00000321348	DIAPH2	Protein diaphanous homolog 2; Could be involved in oogenesis. Involved in the regulation of endosome dynamics. Implicated in a novel signal transduction pathway, in which isoform 3 and CSK are sequentially activated by RHOD to regulate the motility of early endosomes through interactions with the actin cytoskeleton; Belongs to the formin homology family. Diaphanous subfamily
67	DIP2C	9606.ENSP00000280886	DIP2C	Disco interacting protein 2 homolog C
68	DIXDC1	9606.ENSP00000394352	DIXDC1	Dixin; Positive effector of the Wnt signaling pathway; activates WNT3A signaling via DVL2. Regulates JNK activation by AXIN1 and DVL2; Belongs to the DIXDC1 family
69	DLGAP2	9606.ENSP00000400258	DLGAP2	Disks large-associated protein 2; May play a role in the molecular organization of synapses and neuronal cell signaling. Could be an adapter protein linking ion channel to the subsynaptic cytoskeleton. May induce enrichment of PSD-95/SAP90 at the plasma membrane; Belongs to the SAPAP family
70	DLK1	9606.ENSP00000340292	DLK1	Protein delta homolog 1; May have a role in neuroendocrine differentiation
71	DMBT1	9606.ENSP00000357905	DMBT1	Deleted in malignant brain tumors 1 protein; May be considered as a candidate tumor suppressor gene for brain, lung, esophageal, gastric, and colorectal cancers. May play roles in mucosal defense system, cellular immune defense and epithelial differentiation. May play a role as an opsonin receptor for SFTPD and SPAR in macrophage tissues throughout the body, including epithelial cells lining the gastrointestinal tract. May play a role in liver regeneration. May be an important factor in fate decision and differentiation of transit-amplifying ductular (oval) cells within the hepatic lin [...]
72	DMD	9606.ENSP00000354923	DMD	Dystrophin; Anchors the extracellular matrix to the cytoskeleton via F-actin. Ligand for dystroglycan. Component of the dystrophin- associated glycoprotein complex which accumulates at the neuromuscular junction (NMJ) and at a variety of synapses in the peripheral and central nervous systems and has a structural function in stabilizing the sarcolemma. Also implicated in signaling events and synaptic transmission; X-linked mental retardation
73	DNAJA2	9606.ENSP00000314030	DNAJA2	DnaJ homolog subfamily A member 2; Co-chaperone of Hsc70. Stimulates ATP hydrolysis and the folding of unfolded proteins mediated by HSPA1A/B (in vitro); DNAJ heat shock proteins
74	DNAJB11	9606.ENSP00000414398	DNAJB11	DnaJ homolog subfamily B member 11; Serves as a co-chaperone for HSPA5. Binds directly to both unfolded proteins that are substrates for ERAD and nascent unfolded peptide chains, but dissociates from the HSPA5-unfolded protein complex before folding is completed. May help recruiting HSPA5 and other chaperones to the substrate. Stimulates HSPA5 ATPase activity; DNAJ heat shock proteins
75	DNHD1	9606.ENSP00000254579	DNHD1	Dynein heavy chain domain 1
76	DPF3	9606.ENSP00000479526	DPF3	Zinc finger protein DPF3; Belongs to the neuron-specific chromatin remodeling complex (nBAF complex). During neural development a switch from a stem/progenitor to a post-mitotic chromatin remodeling mechanism occurs as neurons exit the cell cycle and become committed to their adult state. The transition from proliferating neural stem/progenitor cells to post-mitotic neurons requires a switch in subunit composition of the npBAF and nBAF complexes. As neural progenitors exit mitosis and differentiate into neurons, npBAF complexes which contain ACTL6A/BAF53A and PHF10/BAF45A, are exchange [...]
77	DUSP1	9606.ENSP00000239223	DUSP1	Dual specificity protein phosphatase 1; Dual specificity phosphatase that dephosphorylates MAP kinase MAPK1/ERK2 on both 'Thr-183' and 'Tyr-185', regulating its activity during the meiotic cell cycle; Belongs to the protein-tyrosine phosphatase family. Non-receptor class dual specificity subfamily
78	DUSP4	9606.ENSP00000240100	DUSP4	Dual specificity protein phosphatase 4; Regulates mitogenic signal transduction by dephosphorylating both Thr and Tyr residues on MAP kinases ERK1 and ERK2
79	EDEM1	9606.ENSP00000256497	EDEM1	ER degradation-enhancing alpha-mannosidase-like protein 1; Extracts misfolded glycoproteins, but not glycoproteins undergoing productive folding, from the calnexin cycle. It is directly involved in endoplasmic reticulum-associated degradation (ERAD) and targets misfolded glycoproteins for degradation in an N-glycan-independent manner, probably by forming a complex with SEL1L. It has low mannosidase activity, catalyzing mannose trimming from Man8GlcNAc2 to Man7GlcNAc2; Belongs to the glycosyl hydrolase 47 family
80	ELFN1	9606.ENSP00000456548	ELFN1	Protein ELFN1; Postsynaptic protein that regulates circuit dynamics in the central nervous system by modulating the temporal dynamics of interneuron recruitment. Specifically present in excitatory synapses onto oriens-lacunosum molecular (OLM) interneurons and acts as a regulator of presynaptic release probability to direct the formation of highly facilitating pyramidal-OLM synapses (By similarity). Inhibits phosphatase activity of protein phosphatase 1 (PP1) complexes; Fibronectin type III domain containing
81	ELMO2	9606.ENSP00000290246	ELMO2	Engulfment and cell motility protein 2; Involved in cytoskeletal rearrangements required for phagocytosis of apoptotic cells and cell motility. Acts in association with DOCK1 and CRK. Was initially proposed to be required in complex with DOCK1 to activate Rac Rho small GTPases. May enhance the guanine nucleotide exchange factor (GEF) activity of DOCK1
82	EN1	9606.ENSP00000295206	EN1	Homeobox protein engrailed-1; NKL subclass homeoboxes and pseudogenes
83	EPAS1	9606.ENSP00000263734	EPAS1	Endothelial PAS domain-containing protein 1; Transcription factor involved in the induction of oxygen regulated genes. Binds to core DNA sequence 5'-[AG]CGTG-3' within the hypoxia response element (HRE) of target gene promoters. Regulates the vascular endothelial growth factor (VEGF) expression and seems to be implicated in the development of blood vessels and the tubular system of lung. May also play a role in the formation of the endothelium that gives rise to the blood brain barrier. Potent activator of the Tie-2 tyrosine kinase expression. Activation seems to require recruitment of [...]
84	EPHA4	9606.ENSP00000281821	EPHA4	Ephrin type-A receptor 4; Receptor tyrosine kinase which binds membrane-bound ephrin family ligands residing on adjacent cells, leading to contact-dependent bidirectional signaling into neighboring cells. The signaling pathway downstream of the receptor is referred to as forward signaling while the signaling pathway downstream of the ephrin ligand is referred to as reverse signaling. Highly promiscuous, it has the unique property among Eph receptors to bind and to be physiologically activated by both GPI-anchored ephrin-A and transmembrane ephrin-B ligands including EFNA1 and EFNB3. Up [...]
85	FAM160A2	9606.ENSP00000265978	FAM160A2	FTS and Hook-interacting protein; Component of the FTS/Hook/FHIP complex (FHF complex). The FHF complex may function to promote vesicle trafficking and/or fusion via the homotypic vesicular protein sorting complex (the HOPS complex); Belongs to the UPF0518 family
86	FAM207A	9606.ENSP00000291634	FAM207A	Protein FAM207A; Family with sequence similarity 207 member A
87	FAM72C	9606.ENSP00000463032	FAM72C	Protein FAM72C; Family with sequence similarity 72 member C; Belongs to the FAM72 family
88	FBN1	9606.ENSP00000325527	FBN1	Fibrillin-1; Fibrillin-1: Structural component of the 10-12 nm diameter microfibrils of the extracellular matrix, which conveys both structural and regulatory properties to load-bearing connective tissues. Fibrillin-1- containing microfibrils provide long-term force bearing structural support. In tissues such as the lung, blood vessels and skin, microfibrils form the periphery of the elastic fiber, acting as a scaffold for the deposition of elastin. In addition, microfibrils can occur as elastin-independent networks in tissues such as the ciliary zonule, tendon, cornea and glomerulus w [...]
89	FBRSL1	9606.ENSP00000396160	FBRSL1	Fibrosin-1-like protein; Fibrosin like 1; Belongs to the AUTS2 family
90	FGF13	9606.ENSP00000322390	FGF13	Fibroblast growth factor 13; Microtubule-binding protein which directly binds tubulin and is involved in both polymerization and stabilization of microtubules. Through its action on microtubules, may participate to the refinement of axons by negatively regulating axonal and leading processes branching. Plays a crucial role in neuron polarization and migration in the cerebral cortex and the hippocampus; Belongs to the heparin-binding growth factors family
91	FIBCD1	9606.ENSP00000361413	FIBCD1	Fibrinogen C domain-containing protein 1; Acetyl group-binding receptor which shows a high- affinity and calcium-dependent binding to acetylated structures such as chitin, some N-acetylated carbohydrates, and amino acids, but not to their non-acetylated counterparts. Can facilitate the endocytosis of acetylated components; Fibrinogen C domain containing
93	FKBP6	9606.ENSP00000252037	FKBP6	Inactive peptidyl-prolyl cis-trans isomerase FKBP6; Co-chaperone required during spermatogenesis to repress transposable elements and prevent their mobilization, which is essential for the germline integrity. Acts via the piRNA metabolic process, which mediates the repression of transposable elements during meiosis by forming complexes composed of piRNAs and Piwi proteins and govern the methylation and subsequent repression of transposons. Acts as a co-chaperone via its interaction with HSP90 and is required for the piRNA amplification process, the secondary piRNA biogenesis. May be re [...]
94	FOXD2	9606.ENSP00000335493	FOXD2	Forkhead box protein D2; Probable transcription factor involved in embryogenesis and somatogenesis; Forkhead boxes
95	FOXP1	9606.ENSP00000484803	FOXP1	Forkhead box protein P1; Transcriptional repressor. Can act with CTBP1 to synergistically repress transcription but CTPBP1 is not essential (By similarity). Plays an important role in the specification and differentiation of lung epithelium. Acts cooperatively with FOXP4 to regulate lung secretory epithelial cell fate and regeneration by restricting the goblet cell lineage program; the function may involve regulation of AGR2. Essential transcriptional regulator of B-cell development. Involved in regulation of cardiac muscle cell proliferation. Involved in the columnar organization of s [...]

#	queryItem	stringId	preferredName	annotation
96	FRG1	9606.ENSP00000226798	FRG1	Protein FRG1; Binds to mRNA in a sequence-independent manner. May play a role in regulation of pre-mRNA splicing or in the assembly of rRNA into ribosomal subunits. May be involved in mRNA transport. May be involved in epigenetic regulation of muscle differentiation through regulation of activity of the histone-lysine N- methyltransferase KMT5B; Belongs to the FRG1 family
97	FZD10	9606.ENSP00000229030	FZD10	Frizzled-10; Receptor for Wnt proteins. Most of frizzled receptors are coupled to the beta-catenin canonical signaling pathway, which leads to the activation of disheveled proteins, inhibition of GSK- 3 kinase, nuclear accumulation of beta-catenin and activation of Wnt target genes. A second signaling pathway involving PKC and calcium fluxes has been seen for some family members, but it is not yet clear if it represents a distinct pathway or if it can be integrated in the canonical pathway, as PKC seems to be required for Wnt-mediated inactivation of GSK-3 kinase. Both pathways seem to [...]
98	GALNT9	9606.ENSP00000380488	GALNT9	Polypeptide N-acetylgalactosaminyltransferase 9; Catalyzes the initial reaction in O-linked oligosaccharide biosynthesis, the transfer of an N-acetyl-D- galactosamine residue to a serine or threonine residue on the protein receptor. Does not glycosylate apomucin or SDC3; Polypeptide N-acetylgalactosaminyltransferases
99	GCNT2	9606.ENSP00000368917	GCNT2	N-acetyllactosaminide beta-1,6-N-acetylglucosaminyl-transferase; Branching enzyme that converts linear into branched poly-N-acetyllactosaminoglycans. Introduces the blood group I antigen during embryonic development. It is closely associated with the development and maturation of erythroid cells; Belongs to the glycosyltransferase 14 family
100	GIGYF2	9606.ENSP00000387170	GIGYF2	GRB10-interacting GYF protein 2; Key component of the 4EHP-GYF2 complex, a multiprotein complex that acts as a repressor of translation initiation. In 4EHP-GYF2 the complex, acts as a factor that bridges EIF4E2 to ZFP36/TTP, linking translation repression with mRNA decay (By similarity). May act cooperatively with GRB10 to regulate tyrosine kinase receptor signaling, including IGF1 and insulin receptors; Belongs to the GIGYF family
101	GK	9606.ENSP00000401720	GK	Glycerol kinase; Key enzyme in the regulation of glycerol uptake and metabolism; Belongs to the FGGY kinase family
102	GLOD5	9606.ENSP00000302552	GLOD5	Glyoxalase domain containing 5; Belongs to the glyoxalase I family
103	GLRX3	9606.ENSP00000357633	GLRX3	Glutaredoxin-3; Together with BOLA2, acts as a cytosolic iron-sulfur (Fe-S) cluster assembly factor that facilitates [2Fe-2S] cluster insertion into a subset of cytosolic proteins. Acts as a critical negative regulator of cardiac hypertrophy and a positive inotropic regulator (By similarity). Required for hemoglobin maturation. Does not possess any thyoredoxin activity since it lacks the conserved motif that is essential for catalytic activity; Glutaredoxin domain containing
104	GLTP	9606.ENSP00000315263	GLTP	Glycolipid transfer protein; Accelerates the intermembrane transfer of various glycolipids. Catalyzes the transfer of various glycosphingolipids between membranes but does not catalyze the transfer of phospholipids. May be involved in the intracellular translocation of glucosylceramides
105	GMDS	9606.ENSP00000370194	GMDS	GDP-mannose 4,6 dehydratase; Catalyzes the conversion of GDP-D-mannose to GDP-4- dehydro-6-deoxy-D-mannose; Belongs to the NAD(P)-dependent epimerase/dehydratase family. GDP-mannose 4,6-dehydratase subfamily
106	GPRASP2	9606.ENSP00000437872	GPRASP2	G-protein coupled receptor-associated sorting protein 2; May play a role in regulation of a variety of G-protein coupled receptors; Belongs to the GPRASP family
107	GPX3	9606.ENSP00000373477	GPX3	Glutathione peroxidase 3; Protects cells and enzymes from oxidative damage, by catalyzing the reduction of hydrogen peroxide, lipid peroxides and organic hydroperoxide, by glutathione; Selenoproteins
108	GPX5	9606.ENSP00000392398	GPX5	Epididymal secretory glutathione peroxidase; Protects cells and enzymes from oxidative damage, by catalyzing the reduction of hydrogen peroxide, lipid peroxides and organic hydroperoxide, by glutathione. May constitute a glutathione peroxidase-like protective system against peroxide damage in sperm membrane lipids
110	HERPUD2	9606.ENSP00000379390	HERPUD2	Homocysteine-responsive endoplasmic reticulum-resident ubiquitin-like domain member 2 protein; Could be involved in the unfolded protein response (UPR) pathway
111	HMGB4	9606.ENSP00000430919	HMGB4	Canonical high mobility group; Belongs to the HMGB family
113	HS3ST3B1	9606.ENSP00000354213	HS3ST3B1	Heparan sulfate glucosamine 3-O-sulfotransferase 3B1; Sulfotransferase that utilizes 3'-phospho-5'-adenylyl sulfate (PAPS) to catalyze the transfer of a sulfo group to an N- unsubstituted glucosamine linked to a 2-O-sulfo iduronic acid unit on heparan sulfate. Catalyzes the O-sulfation of glucosamine in IdoUA2S-GlcNS and also in IdoUA2S-GlcNH2. The substrate-specific O-sulfation generates an enzyme-modified heparan sulfate which acts as a binding receptor to Herpes simplex virus-1 (HSV-1) and permits its entry. Unlike 3-OST-1, does not convert non- anticoagulant heparan sulfate to anti [...]
114	IGFL4	9606.ENSP00000366926	IGFL4	IGF like family member 4; Belongs to the IGFL family
115	IKZF4	9606.ENSP00000262032	IKZF4	Zinc finger protein Eos; DNA-binding protein that binds to the 5'GGGAATRCC-3' Ikaros-binding sequence. Transcriptional repressor. Interacts with SPI1 and MITF to repress transcription of the CTSK and ACP5 promoters via recruitment of corepressors SIN3A and CTBP2. May be involved in the development of central and peripheral nervous systems. Essential for the inhibitory function of regulatory T- cells (Treg). Mediates FOXP3-mediated gene silencing in regulatory T-cells (Treg) via recruitment of corepressor CTBP1 (By similarity)
116	IL17D	9606.ENSP00000302924	IL17D	Interleukin-17D; Induces expression of IL6, CXCL8/IL8, and CSF2/GM-CSF from endothelial cells; Interleukins
117	IL9	9606.ENSP00000274520	IL9	Interleukin-9; Supports IL-2 independent and IL-4 independent growth of helper T-cells; Interleukins
118	ILDR2	9606.ENSP00000271417	ILDR2	Immunoglobulin-like domain-containing receptor 2; May be involved in lipid homeostasis and ER stress pathways
119	INSC	9606.ENSP00000368872	INSC	Protein inscuteable homolog; May function as an adapter linking the Par3 complex to the GPSM1/GPSM2 complex. Involved in spindle orientation during mitosis. May regulate cell proliferation and differentiation in the developing nervous system. May play a role in the asymmetric division of fibroblasts and participate in the process of stratification of the squamous epithelium (By similarity); Armadillo-like helical domain containing
120	IQSEC2	9606.ENSP00000379712	IQSEC2	IQ motif and SEC7 domain-containing protein 2; Is a guanine nucleotide exchange factor for the ARF GTP- binding proteins; X-linked mental retardation
121	IRX1	9606.ENSP00000305244	IRX1	Iroquois-class homeodomain protein IRX-1; TALE class homeoboxes and pseudogenes
122	ITGB8	9606.ENSP00000222573	ITGB8	Integrin beta-8; Integrin alpha-V/beta-8 is a receptor for fibronectin
124	JADE1	9606.ENSP00000226319	JADE1	Protein Jade-1; Component of the HBO1 complex which has a histone H4- specific acetyltransferase activity, a reduced activity toward histone H3 and is responsible for the bulk of histone H4 acetylation in vivo. Transcriptional coactivator, it may also promote acetylation of nucleosomal histone H4 by KAT5. Promotes apoptosis. May act as a renal tumor suppressor. Negatively regulates canonical Wnt signaling; at least in part, cooperates with NPHP4 in this function; PHD finger proteins
125	JAM3	9606.ENSP00000299106	JAM3	Junctional adhesion molecule C; Participates in cell-cell adhesion. It is a counter- receptor for ITGAM, mediating leukocyte-platelet interactions and is involved in the regulation of transepithelial migration of polymorphonuclear neutrophils (PMN). The soluble form is a mediator of angiogenesis; Belongs to the immunoglobulin superfamily
126	KAT6B	9606.ENSP00000287239	KAT6B	Histone acetyltransferase KAT6B; Histone acetyltransferase which may be involved in both positive and negative regulation of transcription. Required for RUNX2-dependent transcriptional activation. May be involved in cerebral cortex development. Component of the MOZ/MORF complex which has a histone H3 acetyltransferase activity; Belongs to the MYST (SAS/MOZ) family
127	KCNA4	9606.ENSP00000328511	KCNA4	Potassium voltage-gated channel subfamily A member 4; Voltage-gated potassium channel that mediates transmembrane potassium transport in excitable membranes. Forms tetrameric potassium-selective channels through which potassium ions pass in accordance with their electrochemical gradient. The channel alternates between opened and closed conformations in response to the voltage difference across the membrane. Can form functional homotetrameric channels and heterotetrameric channels that contain variable proportions of KCNA1, KCNA2, KCNA4, KCNA5, and possibly other family members as well; [...]
128	KCNMA1	9606.ENSP00000286628	KCNMA1	Calcium-activated potassium channel subunit alpha-1; Potassium channel activated by both membrane depolarization or increase in cytosolic Ca(2+) that mediates export of K(+). It is also activated by the concentration of cytosolic Mg(2+). Its activation dampens the excitatory events that elevate the cytosolic Ca(2+) concentration and/or depolarize the cell membrane. It therefore contributes to repolarization of the membrane potential. Plays a key role in controlling excitability in a number of systems, such as regulation of the contraction of smooth muscle, the tuning of hair cells in t [...]
129	KIF7	9606.ENSP00000377934	KIF7	Kinesin-like protein KIF7; Essential for hedgehog signaling regulation: acts as both a negative and positive regulator of sonic hedgehog (Shh) and Indian hedgehog (Ihh) pathways, acting downstream of SMO, through both SUFU-dependent and -independent mechanisms. Involved in the regulation of microtubular dynamics. Required for proper organization of the ciliary tip and control of ciliary localization of SUFU-GLI2 complexes (By similarity). Required for localization of GLI3 to cilia in response to Shh. Negatively regulates Shh signaling by preventing inappropriate activation of the trans [...]
130	KIRREL2	9606.ENSP00000353331	KIRREL2	Kin of IRRE-like protein 2; Kirre like nephrin family adhesion molecule 2; C2-set domain containing
131	KLF6	9606.ENSP00000419923	KLF6	Krueppel-like factor 6; Transcriptional activator (By similarity). Binds a GC box motif. Could play a role in B-cell growth and development; Belongs to the krueppel C2H2-type zinc-finger protein family
132	KRT33A	9606.ENSP00000007735	KRT33A	Keratin, type I cuticular Ha3-I; Keratins, type I; Belongs to the intermediate filament family
133	KTN1	9606.ENSP00000378725	KTN1	Kinectin; Receptor for kinesin thus involved in kinesin-driven vesicle motility. Accumulates in integrin-based adhesion complexes (IAC) upon integrin aggregation by fibronectin; Belongs to the kinectin family
134	LAMA2	9606.ENSP00000400365	LAMA2	Laminin subunit alpha-2; Binding to cells via a high affinity receptor, laminin is thought to mediate the attachment, migration and organization of cells into tissues during embryonic development by interacting with other extracellular matrix components
135	LAMA4	9606.ENSP00000230538	LAMA4	Laminin subunit alpha-4; Binding to cells via a high affinity receptor, laminin is thought to mediate the attachment, migration and organization of cells into tissues during embryonic development by interacting with other extracellular matrix components
136	LAMC2	9606.ENSP00000264144	LAMC2	Laminin subunit gamma-2; Binding to cells via a high affinity receptor, laminin is thought to mediate the attachment, migration and organization of cells into tissues during embryonic development by interacting with other extracellular matrix components. Ladsin exerts cell-scattering activity toward a wide variety of cells, including epithelial, endothelial, and fibroblastic cells
137	LGI4	9606.ENSP00000312273	LGI4	Leucine rich repeat LGI family member 4
138	LMX1B	9606.ENSP00000347684	LMX1B	LIM homeobox transcription factor 1-beta; Essential for the specification of dorsal limb fate at both the zeugopodal and autopodal levels; LIM class homeoboxes
140	LRP1	9606.ENSP00000243077	LRP1	Prolow-density lipoprotein receptor-related protein 1; Endocytic receptor involved in endocytosis and in phagocytosis of apoptotic cells. Required for early embryonic development. Involved in cellular lipid homeostasis. Involved in the plasma clearance of chylomicron remnants and activated LRPAP1 (alpha 2-macroglobulin), as well as the local metabolism of complexes between plasminogen activators and their endogenous inhibitors. May modulate cellular events, such as APP metabolism, kinase-dependent intracellular signaling, neuronal calcium signaling as well as neurotransmission. Acts as [...]
141	LRP8	9606.ENSP00000303634	LRP8	Low-density lipoprotein receptor-related protein 8; Cell surface receptor for Reelin (RELN) and apolipoprotein E (apoE)-containing ligands. LRP8 participates in transmitting the extracellular Reelin signal to intracellular signaling processes, by binding to DAB1 on its cytoplasmic tail. Reelin acts via both the VLDL receptor (VLDLR) and LRP8 to regulate DAB1 tyrosine phosphorylation and microtubule function in neurons. LRP8 has higher affinity for Reelin than VLDLR. LRP8 is thus a key component of the Reelin pathway which governs neuronal layering of the forebrain during embryonic brai [...]
142	LY6K	9606.ENSP00000292430	LY6K	Lymphocyte antigen 6K; Required for sperm migration into the oviduct and male fertility by controlling binding of sperm to zona pellucida (By similarity). May play a role in cell growth; LY6/PLAUR domain containing
143	LZTS1	9606.ENSP00000370981	LZTS1	Leucine zipper putative tumor suppressor 1; Involved in the regulation of cell growth. May stabilize the active CDC2-cyclin B1 complex and thereby contribute to the regulation of the cell cycle and the prevention of uncontrolled cell proliferation. May act as a tumor suppressor; Leucine zipper tumor suppressor family
144	LZTS3	9606.ENSP00000353496	LZTS3	Leucine zipper putative tumor suppressor 3; May be involved in promoting the maturation of dendritic spines, probably via regulating SIPA1L1 levels at the postsynaptic density of synapses; Belongs to the LZTS3 family
145	MAML1	9606.ENSP00000292599	MAML1	Mastermind-like protein 1; Acts as a transcriptional coactivator for NOTCH proteins. Has been shown to amplify NOTCH-induced transcription of HES1. Enhances phosphorylation and proteolytic turnover of the NOTCH intracellular domain in the nucleus through interaction with CDK8. Binds to CREBBP/CBP which promotes nucleosome acetylation at NOTCH enhancers and activates transcription. Induces phosphorylation and localization of CREBBP to nuclear foci. Plays a role in hematopoietic development by regulating NOTCH-mediated lymphoid cell fate decisions; Belongs to the mastermind family
146	MAX	9606.ENSP00000351490	MAX	Protein max; Transcription regulator. Forms a sequence-specific DNA- binding protein complex with MYC or MAD which recognizes the core sequence 5'-CAC[GA]TG-3'. The MYC:MAX complex is a transcriptional activator, whereas the MAD:MAX complex is a repressor. May repress transcription via the recruitment of a chromatin remodeling complex containing H3 'Lys-9' histone methyltransferase activity. Represses MYC transcriptional activity from E-box elements

#	queryItem	stringId	preferredName	annotation
147	MBOAT1	9606.ENSP00000324944	MBOAT1	Lysophospholipid acyltransferase 1; Acyltransferase which mediates the conversion of lysophosphatidylserine (1-acyl-2-hydroxy-sn-glycero-3-phospho-L- serine or LPS) into phosphatidylserine (1,2-diacyl-sn-glycero-3- phospho-L-serine or PS) (LPSAT activity). Prefers oleoyl-CoA as the acyl donor. Lysophospholipid acyltransferases (LPLATs) catalyze the reacylation step of the phospholipid remodeling pathway also known as the Lands cycle; Membrane bound O-acyltransferases
148	MBP	9606.ENSP00000380958	MBP	Myelin basic protein; The classic group of MBP isoforms (isoform 4-isoform 14) are with PLP the most abundant protein components of the myelin membrane in the CNS. They have a role in both its formation and stabilization. The smaller isoforms might have an important role in remyelination of denuded axons in multiple sclerosis. The non- classic group of MBP isoforms (isoform 1-isoform 3/Golli-MBPs) may preferentially have a role in the early developing brain long before myelination, maybe as components of transcriptional complexes, and may also be involved in signaling pathways in T- ce [...]
149	MCTS1	9606.ENSP00000360365	MCTS1	Malignant T-cell-amplified sequence 1; Anti-oncogene that plays a role in cell cycle regulation; decreases cell doubling time and anchorage-dependent growth; shortens the duration of G1 transit time and G1/S transition. When constitutively expressed, increases CDK4 and CDK6 kinases activity and CCND1/cyclin D1 protein level, as well as G1 cyclin/CDK complex formation. Involved in translation initiation; promotes recruitment of aminoacetyled initiator tRNA to P site of 40S ribosomes. Can promote release of deacylated tRNA and mRNA from recycled 40S subunits following ABCE1-mediated diss [...]
151	MEIS2	9606.ENSP00000453793	MEIS2	Homeobox protein Meis2; Involved in transcriptional regulation. Binds to HOX or PBX proteins to form dimers, or to a DNA-bound dimer of PBX and HOX proteins and thought to have a role in stabilization of the homeoprotein-DNA complex. Isoform 3 is required for the activity of a PDX1:PBX1b:MEIS2b complex in pancreatic acinar cells involved in the transcriptional activation of the ELA1 enhancer; the complex binds to the enhancer B element and cooperates with the transcription factor 1 complex (PTF1) bound to the enhancer A element; MEIS2 is not involved in complex DNA-binding. Probably in [...]
152	METTL4	9606.ENSP00000458290	METTL4	Methyltransferase-like protein 4; Probable methyltransferase
153	MEX3C	9606.ENSP00000385610	MEX3C	RNA-binding E3 ubiquitin-protein ligase MEX3C; E3 ubiquitin ligase responsible for the post- transcriptional regulation of common HLA-A allotypes. Binds to the 3' UTR of HLA-A2 mRNA, and regulates its levels by promoting mRNA decay. RNA binding is sufficient to prevent translation, but ubiquitin ligase activity is required for mRNA degradation; Ring finger proteins
154	MFAP3L	9606.ENSP00000354583	MFAP3L	Microfibrillar-associated protein 3-like; May participate in the nuclear signaling of EGFR and MAPK1/ERK2. May a have a role in metastasis; I-set domain containing
156	MOB1A	9606.ENSP00000379364	MOB1A	MOB kinase activator 1A; Activator of LATS1/2 in the Hippo signaling pathway which plays a pivotal role in organ size control and tumor suppression by restricting proliferation and promoting apoptosis. The core of this pathway is composed of a kinase cascade wherein STK3/MST2 and STK4/MST1, in complex with its regulatory protein SAV1, phosphorylates and activates LATS1/2 in complex with its regulatory protein MOB1, which in turn phosphorylates and inactivates YAP1 oncoprotein and WWTR1/TAZ. Phosphorylation of YAP1 by LATS1/2 inhibits its translocation into the nucleus to regulate cellu [...]
157	MRPL23	9606.ENSP00000380466	RPL23L	Mitochondrial ribosomal protein L23
158	MSI2	9606.ENSP00000284073	MSI2	RNA-binding protein Musashi homolog 2; RNA binding protein that regulates the expression of target mRNAs at the translation level. May play a role in the proliferation and maintenance of stem cells in the central nervous system (By similarity); Belongs to the Musashi family
159	MSL3	9606.ENSP00000312244	MSL3	Male-specific lethal 3 homolog; May be involved in chromatin remodeling and transcriptional regulation. May have a role in X inactivation. Component of the MSL complex which is responsible for the majority of histone H4 acetylation at 'Lys-16' which is implicated in the formation of higher-order chromatin structure. Specifically recognizes histone H4 monomethylated at 'Lys-20' (H4K20Me1) in a DNA-dependent manner and is proposed to be involved in chromosomal targeting of the MSL complex
160	MTCL1	9606.ENSP00000352927	MTCL1	Microtubule cross-linking factor 1; Microtubule-associated factor involved in the late phase of epithelial polarization and microtubule dynamics regulation. Plays a role in the development and maintenance of non-centrosomal microtubule bundles at the lateral membrane in polarized epithelial cells; Belongs to the SOGA family
161	MTERF	9606.ENSP00000248643	MTERF1	Transcription termination factor 1, mitochondrial; Transcription termination factor. Binds to a 28 bp region within the tRNA(Leu(uur)) gene at a position immediately adjacent to and downstream of the 16S rRNA gene; this region comprises a tridecamer sequence critical for directing accurate termination. Binds DNA along the major grove and promotes DNA bending and partial unwinding. Promotes base flipping. Transcription termination activity appears to be polarized with highest specificity for transcripts initiated on the light strand; Belongs to the mTERF family
162	MX2	9606.ENSP00000333657	MX2	Interferon-induced GTP-binding protein Mx2; Interferon-induced dynamin-like GTPase with potent antiviral activity against human immunodeficiency virus type 1 (HIV-1). Acts by targeting the viral capsid and affects the nuclear uptake and/or stability of the HIV-1 replication complex and the subsequent chromosomal integration of the proviral DNA. Exhibits antiviral activity also against simian immunodeficiency virus (SIV-mnd). May play a role in regulating nucleocytoplasmic transport and cell-cycle progression
163	MYO18A	9606.ENSP00000437073	MYO18A	Unconventional myosin-XVIIIa; May link Golgi membranes to the cytoskeleton and participate in the tensile force required for vesicle budding from the Golgi. Thereby, may play a role in Golgi membrane trafficking and could indirectly give its flattened shape to the Golgi apparatus. Alternatively, in concert with LURAP1 and CDC42BPA/CDC42BPB, has been involved in modulating lamellar actomyosin retrograde flow that is crucial to cell protrusion and migration. May be involved in the maintenance of the stromal cell architectures required for cell to cell contact (By similarity). Regulates t [...]
164	MYO7A	9606.ENSP00000386331	MYO7A	Unconventional myosin-VIIa; Myosins are actin-based motor molecules with ATPase activity. Unconventional myosins serve in intracellular movements. Their highly divergent tails bind to membranous compartments, which are then moved relative to actin filaments. In the retina, plays an important role in the renewal of the outer photoreceptor disks. Plays an important role in the distribution and migration of retinal pigment epithelial (RPE) melanosomes and phagosomes, and in the regulation of opsin transport in retinal photoreceptors. In the inner ear, plays an important role in differenti [...]
165	NCAM1	9606.ENSP00000480132	NCAM1	Neural cell adhesion molecule 1; This protein is a cell adhesion molecule involved in neuron-neuron adhesion, neurite fasciculation, outgrowth of neurites, etc; CD molecules
166	NCKAP5	9606.ENSP00000387128	NCKAP5	NCK associated protein 5
168	NKAPL	9606.ENSP00000345716	NKAPL	NKAP-like protein; Transcriptional repressor of Notch-mediated signaling. Required for spermatogenesis
169	NMT2	9606.ENSP00000367407	NMT2	Glycylpeptide N-tetradecanoyltransferase 2; Adds a myristoyl group to the N-terminal glycine residue of certain cellular and viral proteins
170	NPAS2	9606.ENSP00000338283	NPAS2	Neuronal PAS domain-containing protein 2; Transcriptional activator which forms a core component of the circadian clock. The circadian clock, an internal time- keeping system, regulates various physiological processes through the generation of approximately 24 hour circadian rhythms in gene expression, which are translated into rhythms in metabolism and behavior. It is derived from the Latin roots 'circa' (about) and 'diem' (day) and acts as an important regulator of a wide array of physiological functions including metabolism, sleep, body temperature, blood pressure, endocrine, immune [...]
171	NTM	9606.ENSP00000396722	NTM	Neurotrimin; Neural cell adhesion molecule; Belongs to the immunoglobulin superfamily. IgLON family
172	NUFIP1	9606.ENSP00000368459	NUFIP1	Nuclear fragile X mental retardation-interacting protein 1; Binds RNA
173	ONECUT1	9606.ENSP00000302630	ONECUT1	Hepatocyte nuclear factor 6; Transcriptional activator. Binds the consensus sequence 5'-DHWATTGAYTWWD-3' on a variety of gene promoters such as those of HNF3B and TTR. Important for liver genes transcription; Belongs to the CUT homeobox family
174	OR5AU1	9606.ENSP00000302057	OR5AU1	Olfactory receptor 5AU1; Odorant receptor; Olfactory receptors, family 5
175	OSBP	9606.ENSP00000263847	OSBP	Oxysterol-binding protein 1; Lipid transporter involved in lipid countertransport between the Golgi complex and membranes of the endoplasmic reticulum: specifically exchanges sterol with phosphatidylinositol 4-phosphate (PI4P), delivering sterol to the Golgi in exchange for PI4P, which is degraded by the SAC1/SACM1L phosphatase in the endoplasmic reticulum. Binds cholesterol and a range of oxysterols including 25-hydroxycholesterol. Cholesterol binding promotes the formation of a complex with PP2A and a tyrosine phosphatase which dephosphorylates ERK1/2, whereas 25-hydroxycholesterol c [...]
176	OTX2	9606.ENSP00000343819	OTX2	Homeobox protein OTX2; Transcription factor probably involved in the development of the brain and the sense organs. Can bind to the bicoid/BCD target sequence (BTS): 5'-TCTAATCCC-3'; Belongs to the paired homeobox family. Bicoid subfamily
178	PCDH7	9606.ENSP00000441802	PCDH7	Protocadherin-7; Protein phosphatase 1 regulatory subunits; Non-clustered protocadherins
179	PCDH9	9606.ENSP00000367096	PCDH9	Protocadherin-9; Potential calcium-dependent cell-adhesion protein; Non-clustered protocadherins
180	PCDHA1	9606.ENSP00000420840	PCDHA1	Protocadherin alpha-1; Potential calcium-dependent cell-adhesion protein. May be involved in the establishment and maintenance of specific neuronal connections in the brain; Clustered protocadherins
181	PCDHA6	9606.ENSP00000433378	PCDHA6	Protocadherin alpha-6; Potential calcium-dependent cell-adhesion protein. May be involved in the establishment and maintenance of specific neuronal connections in the brain; Clustered protocadherins
182	PCDHB16	9606.ENSP00000477314	PCDHB16	Protocadherin beta-16; Potential calcium-dependent cell-adhesion protein. May be involved in the establishment and maintenance of specific neuronal connections in the brain; Clustered protocadherins
183	PCDHGA2	9606.ENSP00000378077	PCDHGA2	Protocadherin gamma-A2; Potential calcium-dependent cell-adhesion protein. May be involved in the establishment and maintenance of specific neuronal connections in the brain; Clustered protocadherins
184	PCDHGA4	9606.ENSP00000458570	PCDHGA4	Protocadherin gamma-A4; Potential calcium-dependent cell-adhesion protein. May be involved in the establishment and maintenance of specific neuronal connections in the brain; Clustered protocadherins
185	PCDHGA5	9606.ENSP00000429834	PCDHGA5	Protocadherin gamma-A5; Potential calcium-dependent cell-adhesion protein. May be involved in the establishment and maintenance of specific neuronal connections in the brain; Clustered protocadherins
186	PDE2A	9606.ENSP00000334910	PDE2A	cGMP-dependent 3',5'-cyclic phosphodiesterase; Cyclic nucleotide phosphodiesterase with a dual- specificity for the second messengers cAMP and cGMP, which are key regulators of many important physiological processes. Plays an important role in growth and invasion of malignant melanoma cells (e.g. pseudomyxoma peritonei (PMP) cell line)
187	PDXK	9606.ENSP00000291565	PDXK	Pyridoxal kinase; Required for synthesis of pyridoxal-5-phosphate from vitamin B6; Belongs to the pyridoxine kinase family
188	PEX12	9606.ENSP00000482609	PEX12	Peroxisome assembly protein 12; Required for protein import into peroxisomes; Peroxins
189	PFKP	9606.ENSP00000370517	PFKP	ATP-dependent 6-phosphofructokinase, platelet type; Catalyzes the phosphorylation of D-fructose 6-phosphate to fructose 1,6-bisphosphate by ATP, the first committing step of glycolysis; Belongs to the phosphofructokinase type A (PFKA) family. ATP-dependent PFK group I subfamily. Eukaryotic two domain clade "E" sub-subfamily
190	PGBD1	9606.ENSP00000259883	PGBD1	PiggyBac transposable element-derived protein 1; Scavenger receptor cysteine rich domain containing; DNA transposon derived genes
191	PGRMC1	9606.ENSP00000217971	PGRMC1	Membrane-associated progesterone receptor component 1; Component of a progesterone-binding protein complex. Binds progesterone. Has many reported cellular functions (heme homeostasis, interaction with CYPs); Belongs to the cytochrome b5 family. MAPR subfamily
192	PITX1	9606.ENSP00000265340	PITX1	Pituitary homeobox 1; Sequence-specific transcription factor that binds gene promoters and activates their transcription. May play a role in the development of anterior structures, and in particular, the brain and facies and in specifying the identity or structure of hindlimb; Belongs to the paired homeobox family. Bicoid subfamily
193	PLA2G12B	9606.ENSP00000362123	PLA2G12B	Group XIIB secretory phospholipase A2-like protein; Not known; does not seem to have catalytic activity; Phospholipases
194	PLET1	9606.ENSP00000341412	PLET1	Placenta-expressed transcript 1 protein; Modulates leading keratinocyte migration and cellular adhesion to matrix proteins during a wound-healing response and promotes wound repair. May play a role during trichilemmal differentiation of the hair follicle (By similarity)
195	PMEPA1	9606.ENSP00000345826	PMEPA1	Protein TMEPAI; Functions as a negative regulator of TGF-beta signaling and thereby probably plays a role in cell proliferation, differentiation, apoptosis, motility, extracellular matrix production and immunosuppression. In the canonical TGF-beta pathway, ZFYVE9/SARA recruits the intracellular signal transducer and transcriptional modulators SMAD2 and SMAD3 to the TGF-beta receptor. Phosphorylated by the receptor, SMAD2 and SMAD3 then form a heteromeric complex with SMAD4 that translocates to the nucleus to regulate transcription. Through interaction with SMAD2 and SMAD3, LDLRAD4 may [...]
196	PNKD	9606.ENSP00000273077	PNKD	Probable hydrolase PNKD; Probable hydrolase that plays an aggravative role in the development of cardiac hypertrophy via activation of the NF-kappa- B signaling pathway; Belongs to the metallo-beta-lactamase superfamily. Glyoxalase II family
197	PPARGC1A	9606.ENSP00000264867	PPARGC1A	Peroxisome proliferator-activated receptor gamma coactivator 1-alpha; Transcriptional coactivator for steroid receptors and nuclear receptors. Greatly increases the transcriptional activity of PPARG and thyroid hormone receptor on the uncoupling protein promoter. Can regulate key mitochondrial genes that contribute to the program of adaptive thermogenesis. Plays an essential role in metabolic reprogramming in response to dietary availability through coordination of the expression of a wide array of genes involved in glucose and fatty acid metabolism. Induces the expression of PERM1 in [...]
198	PPP2R5C	9606.ENSP00000412324	PPP2R5C	Serine/threonine-protein phosphatase 2A 56 kDa regulatory subunit gamma isoform; The B regulatory subunit might modulate substrate selectivity and catalytic activity, and also might direct the localization of the catalytic enzyme to a particular subcellular compartment. The

#	queryItem	stringId	preferredName	annotation
				PP2A-PPP2R5C holoenzyme may specifically dephosphorylate and activate TP53 and play a role in DNA damage- induced inhibition of cell proliferation. PP2A-PPP2R5C may also regulate the ERK signaling pathway through ERK dephosphorylation
199	PPT1	9606.ENSP00000394863	PPT1	Palmitoyl-protein thioesterase 1; Removes thioester-linked fatty acyl groups such as palmitate from modified cysteine residues in proteins or peptides during lysosomal degradation. Prefers acyl chain lengths of 14 to 18 carbons; Belongs to the palmitoyl-protein thioesterase family
200	PRDM5	9606.ENSP00000264808	PRDM5	PR domain zinc finger protein 5; Sequence-specific DNA-binding transcription factor. Represses transcription at least in part by recruitment of the histone methyltransferase EHMT2/G9A and histone deacetylases such as HDAC1. Regulates hematopoiesis-associated protein-coding and microRNA (miRNA) genes. May regulate the expression of proteins involved in extracellular matrix development and maintenance, including fibrillar collagens, such as COL4A1 and COL11A1, connective tissue components, such as HAPLN1, and molecules regulating cell migration and adhesion, including EDIL3 and TGFB2. Ma [...]
201	PRKCH	9606.ENSP00000329127	PRKCH	Protein kinase C eta type; Calcium-independent, phospholipid- and diacylglycerol (DAG)-dependent serine/threonine-protein kinase that is involved in the regulation of cell differentiation in keratinocytes and pre-B cell receptor, mediates regulation of epithelial tight junction integrity and foam cell formation, and is required for glioblastoma proliferation and apoptosis prevention in MCF-7 cells. In keratinocytes, binds and activates the tyrosine kinase FYN, which in turn blocks epidermal growth factor receptor (EGFR) signaling and leads to keratinocyte growth arrest and differentiat [...]
202	PRMT2	9606.ENSP00000380759	PRMT2	Protein arginine N-methyltransferase 2; Arginine methyltransferase that methylates the guanidino nitrogens of arginyl residues in proteins such as STAT3, FBL, histone H4. Acts as a coactivator (with NCOA2) of the androgen receptor (AR)-mediated transactivation. Acts as a coactivator (with estrogen) of estrogen receptor (ER)-mediated transactivation. Enhances PGR, PPARG, RARA-mediated transactivation. May inhibit NF-kappa-B transcription and promote apoptosis. Represses E2F1 transcriptional activity (in a RB1- dependent manner). May be involved in growth regulation; Belongs to the class [...]
203	PTPRT	9606.ENSP00000362294	PTPRT	Receptor-type tyrosine-protein phosphatase T; May be involved in both signal transduction and cellular adhesion in the CNS; Belongs to the protein-tyrosine phosphatase family. Receptor class 2B subfamily
204	PTPRU	9606.ENSP00000334941	PTPRU	Receptor-type tyrosine-protein phosphatase U; Tyrosine-protein phosphatase which dephosphorylates CTNNB1. Regulates CTNNB1 function both in cell adhesion and signaling. May function in cell proliferation and migration and play a role in the maintenance of epithelial integrity. May play a role in megakaryocytopoiesis; Belongs to the protein-tyrosine phosphatase family. Receptor class 2B subfamily
205	PXDN	9606.ENSP00000252804	PXDN	Peroxidasin homolog; Displays low peroxidase activity and is likely to participate in H(2)O(2) metabolism and peroxidative reactions in the cardiovascular system. Plays a role in extracellular matrix formation; I-set domain containing
206	PXDNL	9606.ENSP00000348645	PXDNL	Peroxidasin-like protein; Isoform PMR1: Endonuclease selectively degrading some target mRNAs while they are engaged by translating ribosomes, among which albumin and beta-globin mRNAs; I-set domain containing
207	RARA	9606.ENSP00000254066	RARA	Retinoic acid receptor alpha; Receptor for retinoic acid. Retinoic acid receptors bind as heterodimers to their target response elements in response to their ligands, all-trans or 9-cis retinoic acid, and regulate gene expression in various biological processes. The RXR/RAR heterodimers bind to the retinoic acid response elements (RARE) composed of tandem 5'-AGGTCA-3' sites known as DR1-DR5. In the absence of ligand, the RXR-RAR heterodimers associate with a multiprotein complex containing transcription corepressors that induce histone acetylation, chromatin condensation and transcript [...]
208	RBFOX3	9606.ENSP00000463653	RBFOX3	RNA binding protein fox-1 homolog 3; Pre-mRNA alternative splicing regulator. Regulates alternative splicing of RBFOX2 to enhance the production of mRNA species that are targeted for nonsense-mediated decay (NMD); RNA binding motif containing
209	RGN	9606.ENSP00000380365	RGN	Regucalcin; Gluconolactonase with low activity towards other sugar lactones, including gulonolactone and galactonolactone. Can also hydrolyze diisopropyl phosphorofluoridate and phenylacetate (in vitro). Calcium-binding protein. Modulates Ca(2+) signaling, and Ca(2+)-dependent cellular processes and enzyme activities (By similarity)
210	RHOBTB2	9606.ENSP00000427926	RHOBTB2	Rho-related BTB domain-containing protein 2; Rho related BTB domain containing 2
211	RIC8B	9606.ENSP00000376583	RIC8B	Synembryn-B; Guanine nucleotide exchange factor (GEF), which can activate some, but not all, G-alpha proteins by exchanging bound GDP for free GTP. Able to potentiate G(olf)-alpha-dependent cAMP accumulation suggesting that it may be an important component for odorant signal transduction; Belongs to the synembryn family
212	RIPK4	9606.ENSP00000332454	RIPK4	Receptor-interacting serine/threonine-protein kinase 4; Involved in stratified epithelial development. It is a direct transcriptional target of TP63. Plays a role in NF-kappa-B activation; Belongs to the protein kinase superfamily. TKL Ser/Thr protein kinase family
213	RNF125	9606.ENSP00000217740	RNF125	E3 ubiquitin-protein ligase RNF125; E3 ubiquitin-protein ligase that mediates ubiquitination and subsequent proteasomal degradation of target proteins, such as DDX58/RIG-I, MAVS/IPS1, IFIH1/MDA5, JAK1 and p53/TP53. Acts as a negative regulator of type I interferon production by mediating ubiquitination of DDX58/RIG-I at 'Lys-181', leading to DDX58/RIG-I degradation. Mediates ubiquitination and subsequent degradation of p53/TP53. Mediates ubiquitination and subsequent degradation of JAK1. Acts as a positive regulator of T-cell activation; Ring finger proteins
214	RNF182	9606.ENSP00000420465	RNF182	E3 ubiquitin-protein ligase RNF182; E3 ubiquitin-protein ligase that mediates the ubiquitination of ATP6V0C and targets it to degradation via the ubiquitin-proteasome pathway; Ring finger proteins
215	ROR1	9606.ENSP00000360120	ROR1	Inactive tyrosine-protein kinase transmembrane receptor ROR1; Has very low kinase activity in vitro and is unlikely to function as a tyrosine kinase in vivo. Receptor for ligand WNT5A which activate downstream NFkB signaling pathway and may result in the inhibition of WNT3A-mediated signaling. In inner ear, crucial for spiral ganglion neurons to innervate auditory hair cells; I-set domain containing
216	RPH3A	9606.ENSP00000374036	RPH3A	Rabphilin-3A; Protein transport. Probably involved with Ras-related protein Rab-3A in synaptic vesicle traffic and/or synaptic vesicle fusion. Could play a role in neurotransmitter release by regulating membrane flow in the nerve terminal (By similarity); Synaptotagmin like tandem C2 proteins
217	RRP15	9606.ENSP00000355899	RRP15	RRP15-like protein; Ribosomal RNA processing 15 homolog
218	RTN4	9606.ENSP00000337838	RTN4	Reticulon-4; Developmental neurite growth regulatory factor with a role as a negative regulator of axon-axon adhesion and growth, and as a facilitator of neurite branching. Regulates neurite fasciculation, branching and extension in the developing nervous system. Involved in down-regulation of growth, stabilization of wiring and restriction of plasticity in the adult CNS. Regulates the radial migration of cortical neurons via an RTN4R-LINGO1 containing receptor complex (By similarity). Isoform 2 reduces the anti-apoptotic activity of Bcl-xl and Bcl-2. This is likely consecutive to thei [...]
219	RTP5	9606.ENSP00000345374	RTP5	Receptor transporter protein 5
220	RUBCN	9606.ENSP00000296343	KIAA0226	Run domain Beclin-1-interacting and cysteine-rich domain-containing protein; Inhibits PIK3C3 activity; under basal conditions negatively regulates PI3K complex II (PI3KC3-C2) function in autophagy. Negatively regulates endosome maturation and degradative endocytic trafficking and impairs autophagosome maturation process. Can sequester UVRAG from association with a class C Vps complex (possibly the HOPS complex) and negatively regulates Rab7 activation
221	RUNX1	9606.ENSP00000300305	RUNX1	Runt-related transcription factor 1; CBF binds to the core site, 5'-PYGPYGGT-3', of a number of enhancers and promoters, including murine leukemia virus, polyomavirus enhancer, T-cell receptor enhancers, LCK, IL-3 and GM-CSF promoters. The alpha subunit binds DNA and appears to have a role in the development of normal hematopoiesis. Isoform AML-1L interferes with the transactivation activity of RUNX1. Acts synergistically with ELF4 to transactivate the IL-3 promoter and with ELF2 to transactivate the mouse BLK promoter. Inhibits KAT6B-dependent transcriptional activation. Controls the [...]
222	SDC3	9606.ENSP00000344468	SDC3	Syndecan-3; Cell surface proteoglycan that may bear heparan sulfate (By similarity). May have a role in the organization of cell shape by affecting the actin cytoskeleton, possibly by transferring signals from the cell surface in a sugar-dependent mechanism
223	SDR42E2	9606.ENSP00000473474	SDR42E2	Short chain dehydrogenase/reductase family 42E, member 2; Belongs to the 3-beta-HSD family
224	SEMA3D	9606.ENSP00000284136	SEMA3D	Semaphorin-3D; Induces the collapse and paralysis of neuronal growth cones. Could potentially act as repulsive cues toward specific neuronal populations. Binds to neuropilin (By similarity); Semaphorins
225	SEMA3F	9606.ENSP00000002829	SEMA3F	Semaphorin-3F; May play a role in cell motility and cell adhesion; Immunoglobulin like domain containing
226	SEMA4A	9606.ENSP00000357268	SEMA4A	Semaphorin-4A; Cell surface receptor for PLXNB1, PLXNB2, PLXNB3 and PLXND1 that plays an important role in cell-cell signaling. Plays a role in priming antigen-specific T-cells, promotes differentiation of Th1 T-helper cells, and thereby contributes to adaptive immunity. Promotes phosphorylation of TIMD2. Inhibits angiogenesis. Promotes axon growth cone collapse. Inhibits axonal extension by providing local signals to specify territories inaccessible for growing axons (By similarity); Semaphorins
227	SEMA6D	9606.ENSP00000324857	SEMA6D	Semaphorin-6D; Shows growth cone collapsing activity on dorsal root ganglion (DRG) neurons in vitro. May be a stop signal for the DRG neurons in their target areas, and possibly also for other neurons. May also be involved in the maintenance and remodeling of neuronal connections; Belongs to the semaphorin family
228	SERP2	9606.ENSP00000368477	SERP2	Stress-associated endoplasmic reticulum protein 2; May interact with target proteins during translocation into the lumen of the endoplasmic reticulum. May protect unfolded target proteins against degradation and facilitate correct glycosylation (Potential)
229	SH3BGRL	9606.ENSP00000362308	SH3BGRL	SH3 domain-binding glutamic acid-rich-like protein; SH3 domain binding glutamate rich protein like
230	SHISA2	9606.ENSP00000313079	SHISA2	Protein shisa-2 homolog; Plays an essential role in the maturation of presomitic mesoderm cells by individual attenuation of both FGF and WNT signaling; Shisa family members
231	SLC28A2	9606.ENSP00000315006	SLC28A2	Sodium/nucleoside cotransporter 2; Sodium-dependent and purine-selective transporter. Exhibits the transport characteristics of the nucleoside transport system cif or N1 subtype (N1/cif) (selective for purine nucleosides and uridine). Plays a critical role in specific uptake and salvage of purine nucleosides in kidney and other tissues; Belongs to the concentrative nucleoside transporter (CNT) (TC 2.A.41) family
232	SLC39A4	9606.ENSP00000301305	SLC39A4	Zinc transporter ZIP4; Plays an important role in cellular zinc homeostasis as a zinc transporter. Regulated in response to zinc availability (By similarity); Solute carriers
233	SLC5A1	9606.ENSP00000266088	SLC5A1	Sodium/glucose cotransporter 1; Actively transports glucose into cells by Na(+) cotransport with a Na(+) to glucose coupling ratio of 2:1. Efficient substrate transport in mammalian kidney is provided by the concerted action of a low affinity high capacity and a high affinity low capacity Na(+)/glucose cotransporter arranged in series along kidney proximal tubules; Solute carriers
234	SLC6A14	9606.ENSP00000470801	SLC6A14	Sodium- and chloride-dependent neutral and basic amino acid transporter B(0+); Mediates the uptake of a broad range of neutral and cationic amino acids (with the exception of proline) in a Na(+)/Cl(-)-dependent manner; Solute carriers
235	SLC8A2	9606.ENSP00000236877	SLC8A2	Sodium/calcium exchanger 2; Mediates the electrogenic exchange of Ca(2+) against Na(+) ions across the cell membrane, and thereby contributes to the regulation of cytoplasmic Ca(2+) levels and Ca(2+)-dependent cellular processes. Contributes to cellular Ca(2+) homeostasis in excitable cells. Contributes to the rapid decrease of cytoplasmic Ca(2+) levels back to baseline after neuronal activation, and thereby contributes to modulate synaptic plasticity, learning and memory. Plays a role in regulating urinary Ca(2+) and Na(+) excretion; Belongs to the Ca(2+):cation antiporter (CaCA) (TC [...]
237	SMPD3	9606.ENSP00000219334	SMPD3	Sphingomyelin phosphodiesterase 3; Catalyzes the hydrolysis of sphingomyelin to form ceramide and phosphocholine. Ceramide mediates numerous cellular functions, such as apoptosis and growth arrest, and is capable of regulating these 2 cellular events independently. Also hydrolyzes sphingosylphosphocholine. Regulates the cell cycle by acting as a growth suppressor in confluent cells. Probably acts as a regulator of postnatal development and participates in bone and dentin mineralization; Belongs to the neutral sphingomyelinase family
238	SNX12	9606.ENSP00000481314	SNX12	Sorting nexin-12; May be involved in several stages of intracellular trafficking; Sorting nexins
239	SORCS1	9606.ENSP00000263054	SORCS1	Sortilin related VPS10 domain containing receptor 1; Belongs to the VPS10-related sortilin family. SORCS subfamily
240	SOX9	9606.ENSP00000245479	SOX9	Transcription factor SOX-9; Transcriptional regulator. Binds to the COL2A1 promoter and activates COL2A1 expression, as part of a complex with ZNF219 (By similarity). Plays a role in chondrocyte differentiation (By similarity). Important for normal skeletal development; SRY-boxes
241	SP9	9606.ENSP00000378418	SP9	Transcription factor Sp9; Transcription factor which plays a key role in limb development. Positively regulates FGF8 expression in the apical ectodermal ridge (AER) and contributes to limb outgrowth in embryos (By similarity); Sp transcription factors
242	SPATA5	9606.ENSP00000274008	SPATA5	Spermatogenesis-associated protein 5; May be involved in morphological and functional mitochondrial transformations during spermatogenesis; Belongs to the AAA ATPase family. AFG2 subfamily
243	SPDL1	9606.ENSP00000265295	SPDL1	Protein Spindly; Required for the localization of dynein and dynactin to the mitotic kintochore. Dynein is believed to control the initial lateral interaction between the kinetochore and spindle microtubules and to facilitate the subsequent formation of end-on kinetochore-microtubule attachments mediated by the NDC80 complex. Also required for correct spindle orientation. Does not appear to be required for the removal of spindle assembly checkpoint (SAC) proteins from the kinetochore upon bipolar spindle attachment. Acts as an adapter protein linking the dynein motor complex to various [...]

Appetitive aggression (AAS)

#	queryItem	stringId	preferredName	annotation
244	SPNS2	9606.ENSP00000333292	SPNS2	Protein spinster homolog 2; Sphingolipid transporter required for migration of myocardial precursors. Transports sphingosine 1-phosphate (S1P), a secreted lipid mediator that plays critical roles in cardiovascular, immunological, and neural development and function. Mediates the export of S1P from cells in the extraembryonic yolk syncytial layer (YSL), thereby regulating myocardial precursor migration
245	SRGAP3	9606.ENSP00000373347	SRGAP3	SLIT-ROBO Rho GTPase-activating protein 3; GTPase-activating protein for RAC1 and perhaps Cdc42, but not for RhoA small GTPase. May attenuate RAC1 signaling in neurons; F-BAR domain containing
246	SSBP3	9606.ENSP00000360371	SSBP3	Single-stranded DNA-binding protein 3; May be involved in transcription regulation of the alpha 2(I) collagen gene where it binds to the single-stranded polypyrimidine sequences in the promoter region
247	ST3GAL1	9606.ENSP00000428540	ST3GAL1	CMP-N-acetylneuraminate-beta-galactosamide-alpha-2,3-sialyltransferase 1; Responsible for the synthesis of the sequence NeuAc- alpha-2,3-Gal-beta-1,3-GalNAc- found on sugar chains O-linked to Thr or Ser and also as a terminal sequence on certain gangliosides. SIAT4A and SIAT4B sialylate the same acceptor substrates but exhibit different Km values; Belongs to the glycosyltransferase 29 family
248	STUM	9606.ENSP00000355752	C1orf95	Protein stum homolog; Chromosome 1 open reading frame 95
249	SUCLG1	9606.ENSP00000377446	SUCLG1	Succinate--CoA ligase [ADP/GDP-forming] subunit alpha, mitochondrial; Succinyl-CoA synthetase functions in the citric acid cycle (TCA), coupling the hydrolysis of succinyl-CoA to the synthesis of either ATP or GTP and thus represents the only step of substrate-level phosphorylation in the TCA. The alpha subunit of the enzyme binds the substrates coenzyme A and phosphate, while succinate binding and specificity for either ATP or GTP is provided by different beta subunits
250	SULT2B1	9606.ENSP00000201586	SULT2B1	Sulfotransferase family cytosolic 2B member 1; Sulfotransferase that utilizes 3'-phospho-5'-adenylyl sulfate (PAPS) as sulfonate donor to catalyze the sulfate conjugation of many hormones, neurotransmitters, drugs and xenobiotic compounds. Sulfonation increases the water solubility of most compounds, and therefore their renal excretion, but it can also result in bioactivation to form active metabolites. Sulfates hydroxysteroids like DHEA. Isoform 1 preferentially sulfonates cholesterol, and isoform 2 avidly sulfonates pregnenolone but not cholesterol. Plays a role in epidermal choleste [...]
251	SYTL4	9606.ENSP00000362080	SYTL4	Synaptotagmin-like protein 4; Modulates exocytosis of dense-core granules and secretion of hormones in the pancreas and the pituitary. Interacts with vesicles containing negatively charged phospholipids in a Ca(2+)-independent manner (By similarity); Synaptotagmin like tandem C2 proteins
252	TAF7	9606.ENSP00000312709	TAF7	Transcription initiation factor TFIID subunit 7; Functions as a component of the DNA-binding general transcription factor complex TFIID, a multimeric protein complex that plays a central role in mediating promoter responses to various activators and repressors. Present in both of the previously described TFIID species which either lack or contain TAFII30 (TFIID alpha and TFIID beta respectively); Belongs to the TAF7 family
253	TBX1	9606.ENSP00000331791	TBX1	T-box transcription factor TBX1; Probable transcriptional regulator involved in developmental processes. Is required for normal development of the pharyngeal arch arteries (By similarity); T-boxes
254	TCEAL7	9606.ENSP00000329794	TCEAL7	Transcription elongation factor A protein-like 7; Plays a role in the negative regulation of NF-kappa-B signaling at the basal level by modulating transcriptional activity of NF-kappa-B on its target gene promoters. Associates with cyclin D1 promoter containing Myc E-box sequence and transcriptionally represses cyclin D1 expression. Regulates telomerase reverse transcriptase expression and telomerase activity in both ALT (alternative lengthening of telomeres)and telomerase-positive cell lines; Transcription elongation factor A like family
255	TCF3	9606.ENSP00000262965	TCF3	Transcription factor E2-alpha; Transcriptional regulator. Involved in the initiation of neuronal differentiation. Heterodimers between TCF3 and tissue- specific basic helix-loop-helix (bHLH) proteins play major roles in determining tissue-specific cell fate during embryogenesis, like muscle or early B-cell differentiation. Dimers bind DNA on E- box motifs: 5'-CANNTG-3'. Binds to the kappa-E2 site in the kappa immunoglobulin gene enhancer. Binds to IEB1 and IEB2, which are short DNA sequences in the insulin gene transcription control region
257	THAP1	9606.ENSP00000254250	THAP1	THAP domain-containing protein 1; DNA-binding transcription regulator that regulates endothelial cell proliferation and G1/S cell-cycle progression. Specifically binds the 5'-[AT]NTNN[GT]GGCA[AGT]-3' core DNA sequence and acts by modulating expression of pRB-E2F cell-cycle target genes, including RRM1. Component of a THAP1/THAP3-HCFC1-OGT complex that is required for the regulation of the transcriptional activity of RRM1. May also have pro-apoptopic activity by potentiating both serum-withdrawal and TNF-induced apoptosis; THAP domain containing
259	TLX1	9606.ENSP00000359215	TLX1	T-cell leukemia homeobox protein 1; Controls the genesis of the spleen. Binds to the DNA sequence 5'-GGCGGTAAGTGG-3'; NKL subclass homeoboxes and pseudogenes
260	TMEM154	9606.ENSP00000302144	TMEM154	Transmembrane protein 154
261	TMEM200A	9606.ENSP00000376224	TMEM200A	Transmembrane protein 200A; Belongs to the TMEM200 family
262	TMEM229B	9606.ENSP00000350050	TMEM229B	Transmembrane protein 229B
263	TNFSF4	9606.ENSP00000281834	TNFSF4	Tumor necrosis factor ligand superfamily member 4; Cytokine that binds to TNFRSF4. Co-stimulates T-cell proliferation and cytokine production; CD molecules
264	TP73	9606.ENSP00000367545	TP73	Tumor protein p73; Participates in the apoptotic response to DNA damage. Isoforms containing the transactivation domain are pro-apoptotic, isoforms lacking the domain are anti-apoptotic and block the function of p53 and transactivating p73 isoforms. May be a tumor suppressor protein
265	TPO	9606.ENSP00000318820	TPO	Thyroid peroxidase; Iodination and coupling of the hormonogenic tyrosines in thyroglobulin to yield the thyroid hormones T(3) and T(4); Belongs to the peroxidase family. XPO subfamily
266	TRIB1	9606.ENSP00000312150	TRIB1	Tribbles homolog 1; Adapter protein involved in protein degradation by interacting with RFWD2/COP1 ubiquitin ligase. The RFWD2-binding motif is masked by autoinhibitory interactions with the protein kinase domain. Serves to alter RFWD2 substrate specificity by directing the activity of RFWD2 toward CEBPA. Binds selectively the recognition sequence of CEBPA. Regulates myeloid cell differentiation by altering the expression of CEBPA in a RFWD2- dependent manner (By similarity). Controls macrophage, eosinophil and neutrophil differentiation via the COP1-binding domain (By similarity). Int [...]
267	TRIM36	9606.ENSP00000282369	TRIM36	E3 ubiquitin-protein ligase TRIM36; E3 ubiquitin-protein ligase which mediates ubiquitination and subsequent proteasomal degradation of target proteins. Involved in chromosome segregation and cell cycle regulation. May play a role in the acrosome reaction and fertilization; Belongs to the TRIM/RBCC family
268	TRPC7	9606.ENSP00000426070	TRPC7	Short transient receptor potential channel 7; Thought to form a receptor-activated non-selective calcium permeant cation channel. Probably is operated by a phosphatidylinositol second messenger system activated by receptor tyrosine kinases or G-protein coupled receptors. Activated by diacylglycerol (DAG) (By similarity). May also be activated by intracellular calcium store depletion; Transient receptor potential cation channels
269	TRPM5	9606.ENSP00000155858	TRPM5	Transient receptor potential cation channel subfamily M member 5; Voltage-modulated Ca(2+)-activated, monovalent cation channel (VCAM) that mediates a transient membrane depolarization and plays a central role in taste transduction. Monovalent- specific, non-selective cation channel that mediates the transport of Na(+), K(+) and Cs(+) ions equally well. Activated directly by increases in intracellular Ca(2+), but is impermeable to it. Gating is voltage-dependent and displays rapid activation and deactivation kinetics upon channel stimulation even during sustained elevations in Ca(2+). [...]
270	TSNAX	9606.ENSP00000355599	TSNAX	Translin-associated protein X; Acts in combination with TSN as an endonuclease involved in the activation of the RNA-induced silencing complex (RISC). Possible role in spermatogenesis
271	TTC27	9606.ENSP00000313953	TTC27	Tetratricopeptide repeat domain containing; Belongs to the TTC27 family
272	TTC7B	9606.ENSP00000336127	TTC7B	Tetratricopeptide repeat protein 7B; Component of a complex required to localize phosphatidylinositol 4-kinase (PI4K) to the plasma membrane. The complex acts as a regulator of phosphatidylinositol 4-phosphate (PtdIns(4)P) synthesis. In the complex, plays a central role in bridging PI4KA to EFR3B and FAM126A, via direct interactions
273	TVP23C	9606.ENSP00000225576	TVP23C	Trans-golgi network vesicle protein 23 homolog C; Belongs to the TVP23 family
274	UACA	9606.ENSP00000314556	UACA	Uveal autoantigen with coiled-coil domains and ankyrin repeats; Regulates APAF1 expression and plays an important role in the regulation of stress-induced apoptosis. Promotes apoptosis by regulating three pathways, apoptosome up-regulation, LGALS3/galectin-3 down-regulation and NF-kappa-B inactivation. Regulates the redistribution of APAF1 into the nucleus after proapoptotic stress. Down-regulates the expression of LGALS3 by inhibiting NFKB1 (By similarity); Ankyrin repeat domain containing
275	UBE2F	9606.ENSP00000478474	UBE2F	NEDD8-conjugating enzyme UBE2F; Accepts the ubiquitin-like protein NEDD8 from the UBA3- NAE1 E1 complex and catalyzes its covalent attachment to other proteins. The specific interaction with the E3 ubiquitin ligase RBX2, but not RBX1, suggests that the RBX2-UBE2F complex neddylates specific target proteins, such as CUL5
276	UNC5CL	9606.ENSP00000244565	UNC5CL	UNC5C-like protein; Inhibits NF-kappa-B-dependent transcription by impairing NF-kappa-B binding to its targets; Belongs to the unc-5 family
277	UPK1B	9606.ENSP00000264234	UPK1B	Uroplakin-1b; Component of the asymmetric unit membrane (AUM); a highly specialized biomembrane elaborated by terminally differentiated urothelial cells. May play an important role in normal bladder epithelial physiology, possibly in regulating membrane permeability of superficial umbrella cells or in stabilizing the apical membrane through AUM/cytoskeletal interactions (By similarity); Belongs to the tetraspanin (TM4SF) family
278	USP12	9606.ENSP00000282344	USP12	Ubiquitin carboxyl-terminal hydrolase 12; Deubiquitinating enzyme. Has almost no deubiquitinating activity by itself and requires the interaction with WDR20 and WDR48 to have a high activity. Not involved in deubiquitination of monoubiquitinated FANCD2. In complex with WDR48, acts as a potential tumor suppressor by positively regulating PHLPP1 stability; Belongs to the peptidase C19 family. USP12/USP46 subfamily
279	USP24	9606.ENSP00000294383	USP24	Ubiquitin carboxyl-terminal hydrolase 24; Protease that can remove conjugated ubiquitin from target proteins and polyubiquitin chains. Deubiquitinates DDB2, preventing its proteasomal degradation; Ubiquitin specific peptidases
280	UTS2R	9606.ENSP00000323516	UTS2R	Urotensin-2 receptor; High affinity receptor for urotensin-2 and urotensin-2B. The activity of this receptor is mediated by a G-protein that activate a phosphatidylinositol-calcium second messenger system
281	VAX2	9606.ENSP00000234392	VAX2	Ventral anterior homeobox 2; Transcription factor that may function in dorsoventral specification of the forebrain. Regulates the expression of Wnt signaling antagonists including the expression of a truncated TCF7L2 isoform that cannot bind CTNNB1 and acts therefore as a potent dominant-negative Wnt antagonist. Plays a crucial role in eye development and, in particular, in the specification of the ventral optic vesicle (By similarity). May be a regulator of axial polarization in the retina; NKL subclass homeoboxes and pseudogenes
282	VRK1	9606.ENSP00000216639	VRK1	Serine/threonine-protein kinase VRK1; Serine/threonine kinase involved in Golgi disassembly during the cell cycle: following phosphorylation by PLK3 during mitosis, required to induce Golgi fragmentation. Acts by mediating phosphorylation of downstream target protein. Phosphorylates 'Thr- 18' of p53/TP53 and may thereby prevent the interaction between p53/TP53 and MDM2. Phosphorylates casein and histone H3. Phosphorylates BANF1: disrupts its ability to bind DNA, reduces its binding to LEM domain-containing proteins and causes its relocalization from the nucleus to the cytoplasm. Phosph [...]
283	VSIG1	9606.ENSP00000402219	VSIG1	V-set and immunoglobulin domain containing 1; IgCAM CXADR-related subfamily
284	WAPL	9606.ENSP00000298767	WAPAL	Wings apart-like protein homolog; Regulator of sister chromatid cohesion in mitosis which negatively regulates cohesin association with chromatin. Involved in both sister chromatid cohesion during interphase and sister- chromatid resolution during early stages of mitosis. Couples DNA replication to sister chromatid cohesion. Cohesion ensures that chromosome partitioning is accurate in both meiotic and mitotic cells and plays an important role in DNA repair
285	WDPCP	9606.ENSP00000272321	WDPCP	WD repeat-containing and planar cell polarity effector protein fritz homolog; Probable effector of the planar cell polarity signaling pathway which regulates the septin cytoskeleton in both ciliogenesis and collective cell movements. Together with FUZ and WDPCP proposed to function as core component of the CPLANE (ciliogenesis and planar polarity effectors) complex involved in the recruitment of peripheral IFT-A proteins to basal bodies (By similarity); Bardet-Biedl syndrome associated
286	WDR27	9606.ENSP00000416289	WDR27	WD repeat-containing protein 27; WD repeat domain containing
287	WDR81	9606.ENSP00000386609	WDR81	WD repeat-containing protein 81; Functions as a negative regulator of the PI3 kinase/PI3K activity associated with endosomal membranes via BECN1, a core subunit of the PI3K complex. By modifying the phosphatidylinositol 3-phosphate/PtdInsP3 content of endosomal membranes may regulate endosome fusion, recycling, sorting and early to late endosome transport. It is for instance, required for the delivery of cargos like BST2/tetherin from early to late endosome and thereby participates indirectly to their degradation by the lysosome. May also play a role in aggrephagy, the macroautophagic [...]
288	WT1	9606.ENSP00000331327	WT1	Wilms tumor protein; Transcription factor that plays an important role in cellular development and cell survival. Recognizes and binds to the DNA sequence 5'-GCG(T/G)GGGCG-3'. Regulates the expression of numerous target genes, including EPO. Plays an essential role for development of the urogenital system. It has a tumor suppressor as well as an oncogenic role in tumor formation. Function may be isoform-

Appetitive aggression (AAS)

#	queryItem	stringId	preferredName	annotation
				specific: isoforms lacking the KTS motif may act as transcription factors. Isoforms containing the KTS motif may bind mRNA and play a role in mRNA metabolism or splicing. Isoform 1 has [...]
289	XXYLT1	9606.ENSP00000309640	XXYLT1	Xyloside xylosyltransferase 1; Alpha-1,3-xylosyltransferase, which elongates the O- linked xylose-glucose disaccharide attached to EGF-like repeats in the extracellular domain of target proteins by catalyzing the addition of the second xylose. Known targets include Notch proteins and coagulation factors, such as F9; Glycosyltransferase family 8
290	YTHDF2	9606.ENSP00000362918	YTHDF2	YTH domain-containing family protein 2; Specifically recognizes and binds N6-methyladenosine (m6A)-containing RNAs, and regulates mRNA stability. M6A is a modification present at internal sites of mRNAs and some non- coding RNAs and plays a role in mRNA stability and processing. Acts as a regulator of mRNA stability: binding to m6A-containing mRNAs results in the localization to mRNA decay sites, such as processing bodies (P-bodies), leading to mRNA degradation. Required maternally to regulate oocyte maturation: probably acts by binding to m6A-containing mRNAs, thereby regulating mater [...]
291	ZC3H12B	9606.ENSP00000340839	ZC3H12B	Probable ribonuclease ZC3H12B; May function as RNase and regulate the levels of target RNA species; Zinc fingers CCCH-type
292	ZC3H18	9606.ENSP00000416951	ZC3H18	Zinc finger CCCH-type containing 18
293	ZEB2	9606.ENSP00000454157	ZEB2	Zinc finger E-box-binding homeobox 2; Transcriptional inhibitor that binds to DNA sequence 5'- CACCT-3' in different promoters. Represses transcription of E- cadherin; ZF class homeoboxes and pseudogenes
294	ZNF696	9606.ENSP00000328515	ZNF696	Zinc finger protein 696; May be involved in transcriptional regulation; Zinc fingers C2H2-type
295	ZNF763	9606.ENSP00000369774	ENSG00000197054	Zinc finger protein 763; May be involved in transcriptional regulation; Zinc fingers C2H2-type

Appetitive aggression (AAS)

Association with Current Violent Behaviour

Gene list

AGO2, AATBC, ABCA2, ABCC5, ACOT7, ACSF3, ADARB1, ADCY10, ADCYAP1, AFF3, AIM2, AKNAD1, AKT1, AKT3, ALDH3A2, ANGPTL6, ANK2, ANKK1, ANKRD9, ANO1, ANP32BP2, ANP32E, ANXA9, ARAP1, ARHGAP44, ARHGEF7, ASAP1, ASNSP1, ATOX1, ATP2C1, AVPR1B, AZU1P1, B3GNTL1, BAHD1, BAIAP2, BMPR1B, BRD4, BSN, BTK, CACNA2D1, CACNB2, CACNG4, CARD11, CARNS1, CASK, CCND1, CCPG1, CD200R1, CDC14B, CDC42EP4, CDH26, CELF1, CELF4, CERS6, CFAP46, CIITA, CIPC, CLDN5, CNPY3, COL15A1, CPLX1, CRABP2, CREB3L1, CRIP2, CTIF, CXADR, CYP2D7, DAOA, DAXX, DICER1, DIO3, DLGAP2, DLX2-DT, DNAJB6, DNHD1, DUSP10, DUSP2, DYSF, ECHDC3, ECM1P2, EIF3G, EXD3, EXTL3, FAM131A, FAM163B, FAM169B, FAM174B, FAM221A, FAM53B, FBXL16, FKBP10, FMNL1, FOXP3, GALE, GLI2, GNB1L, GNG7, GOT2P1, GPR17, GPR173, GPR68, GPSM1, GRB14, GRHL1, GSAP, GTF2I, GTF3C1, GTSF1, GUCY2D, H2AFY, HDAC10, HELZ, HELZ2, HMGN1P19, HOXB9, HSPA12B, HSPD1P5, ID2-AS1, IGF1, IGLV5-37, IGSF21, IL34, IRF2BP2, IRF8, IRX1, JADE1, JAKMIP1, KAT2A, KAZN, KCNE4, KCNG2, KCNK17, KIF21A, KIFC3, KLRG2, KRT7, LBH, LGALS14, LGALS16, LHPP, LMO1, LPAR2, LPCAT4, LRFN3, LY6H, LY6L, MACIR, MACROH2A1, MAFK, MAGEA8, MAML2, MAP3K11, MAP3K7CL, MAP4K1, MAPKAPK5P1, MARCKS, ME3, MEDAG, MEF2C, MEOX1, MICALL2, MID1, MIDEAS, MINAR1, MROH5, MTA1, MTMR6, MTSS1, MTUS2, MTX2, MYBPC3, MYC, MYL3, MYO1H, MYO3B, NDRG2, NKAIN3, NPHP3, NR2F2, NRDE2, NSUN7, NT5DC2, NWD1, OTOG, OTUB1, P2RY2, PAPPA2, PDE4DIP, PGAP1, PGPEP1L, PHGR1, PITHD1, PIWIL2, PKD1, PLAC1, PLCH2, PLEC, PLPPR2, PNKP, PNPLA7, POLA1, PPL, PRDM16, PRDM2, PRKAG2, PRKCB, PRKCZ, PROK2, PRORP, PSEN1, PTCD3, PTPRF, PXDC1, PXN, QKI, RAB3IL1, RAPGEF1, RAPGEFL1, RBFOX3, REEP2, RHCG, RHEB, RPN1, S1PR2, SCRT1, SEC22C, SEMA4C, SEMA6A, SEMA6D, SEPTIN10, SETD1B, SH3BP1, SH3KBP1, SH3PXD2A, SHISA9, SKIDA1, SLC16A2, SLC25A26, SLC2A4RG, SLC35F3, SLC38A5, SLC41A3, SLCO4A1, SMYD3, SNTG1, SOBP, SPACA7, SPEM2, SPIDR, SPRED2, SSTR5-AS1, ST3GAL2, STEAP1B, STIM1, STK10, STX2, TALDO1, TBC1D22A, TCIRG1, TENT5A, TK2, TMED10, TMEM92, TNFRSF6B, TNNT3, TOMM20, TRBC2, TREH, TRIM59, TSNARE1, TTC21B, TTC39C, TTLL4, TUBGCP3, TXLNA, UBAC2, UBE2G2, UBL4A, UTP14A, WARS1, WDR45, WDR5, XYLT2, ZAP70, ZBED9, ZBTB7C, ZC3H11A, ZDBF2, ZMIZ1, ZMYM3, ZNF263, ZNF273, ZNF335, ZNF516, ZNF518A

Network

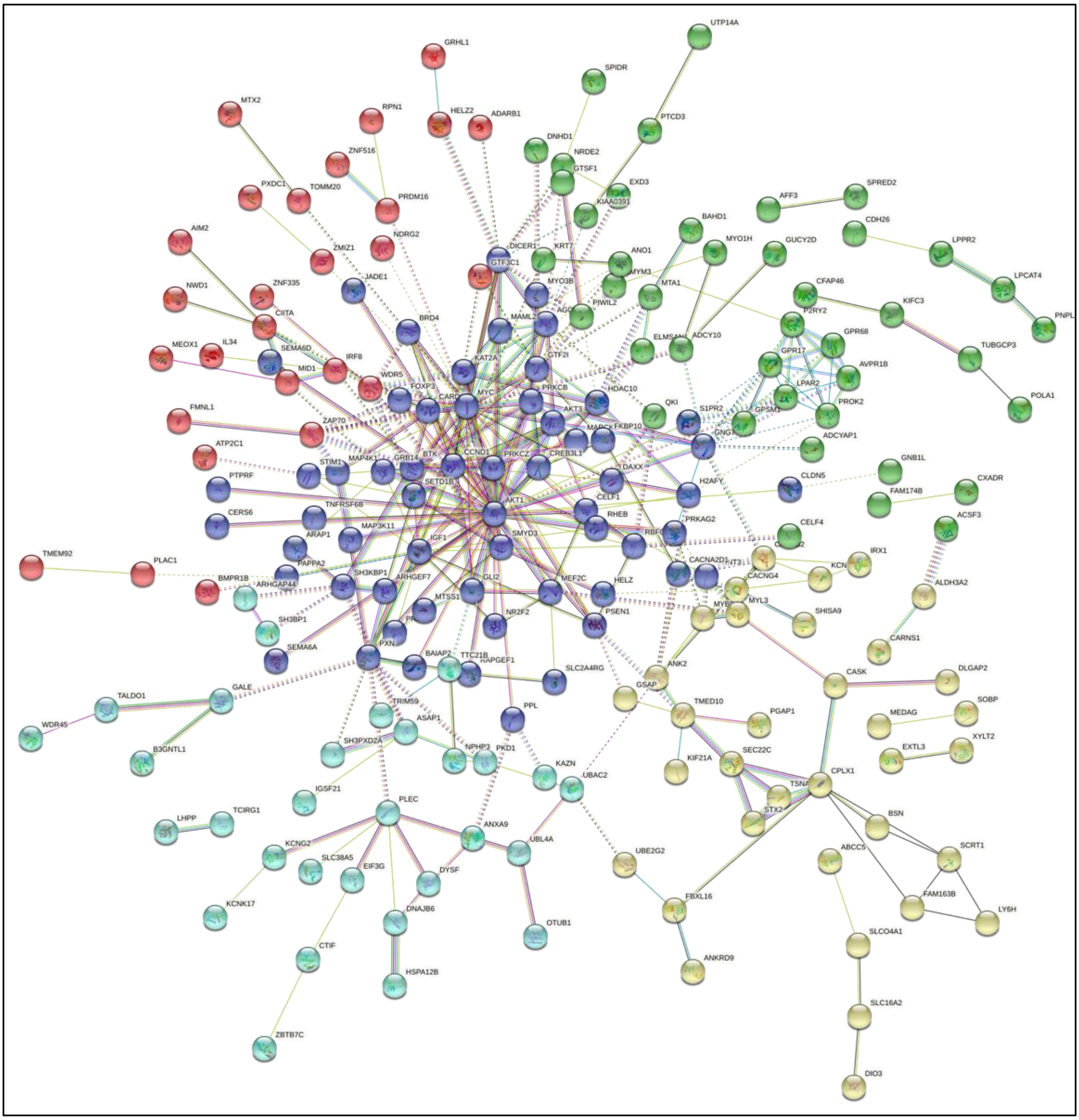

Current Violent Behaviour (CVB)

Enrichments found

Network Stats

number of nodes:	273	expected number of edges:	303
number of edges:	359	PPI enrichment p-value:	0.00099
average node degree:	2.63		
avg. local clustering coefficient:	0.391		

your network has significantly more interactions than expected (what does that mean?)

Enrichment Table Columns

Count In Network:
The first number indicates how many proteins in your network are annotated with a particular term. The second number indicates how many proteins in total (in your network and in the background) have this term assigned.

Strength:
Log10(observed / expected). This measure describes how large the enrichment effect is. It's the ratio between i) the number of proteins in your network that are annotated with a term and ii) the number of proteins that we expect to be annotated with this term in a random network of the same size.

False Discovery Rate:
This measure describes how significant the enrichment is. Shown are p-values corrected for multiple testing within each category using the Benjamini–Hochberg procedure.

Functional enrichments in your network

explain columns

Biological Process (Gene Ontology)

GO-term	description	count in network	strength	false discovery rate
GO:0016246	RNA interference	4 of 10	1.46	0.0326
GO:1902105	regulation of leukocyte differentiation	13 of 261	0.55	0.0326
GO:1903706	regulation of hemopoiesis	17 of 412	0.47	0.0326
GO:0071900	regulation of protein serine/threonine kinase activity	19 of 488	0.45	0.0326
GO:0045597	positive regulation of cell differentiation	28 of 908	0.34	0.0326
GO:0051094	positive regulation of developmental process	37 of 1286	0.31	0.0326
GO:2000026	regulation of multicellular organismal development	50 of 1876	0.28	0.0326
GO:0045595	regulation of cell differentiation	45 of 1695	0.28	0.0326
GO:0044093	positive regulation of molecular function	45 of 1713	0.27	0.0326
GO:0050793	regulation of developmental process	58 of 2416	0.24	0.0326
GO:0051239	regulation of multicellular organismal process	65 of 2788	0.22	0.0326
GO:0048731	system development	85 of 4144	0.17	0.0326
GO:0007275	multicellular organism development	98 of 4726	0.17	0.0326
GO:0048856	anatomical structure development	102 of 5085	0.16	0.0326
GO:0032502	developmental process	105 of 5401	0.14	0.0326

(less ...)

Molecular Function (Gene Ontology)

GO-term	description	count in network	strength	false discovery rate
GO:0019901	protein kinase binding	21 of 599	0.4	0.0336
GO:0005515	protein binding	125 of 6607	0.13	0.0202
GO:0005488	binding	197 of 11878	0.08	0.0202

Cellular Component (Gene Ontology)

GO-term	description	count in network	strength	false discovery rate
GO:0005856	cytoskeleton	50 of 2068	0.24	0.0479

KEGG Pathways

pathway	description	count in network	strength	false discovery rate
hsa05410	Hypertrophic cardiomyopathy (HCM)	7 of 81	0.79	0.0157
hsa04919	Thyroid hormone signaling pathway	9 of 115	0.75	0.0123
hsa04931	Insulin resistance	7 of 107	0.67	0.0452
hsa04371	Apelin signaling pathway	8 of 133	0.63	0.0422
hsa04010	MAPK signaling pathway	14 of 293	0.53	0.0123

Annotated Keywords (UniProt)

keyword	description	count in network	strength	false discovery rate
KW-0728	SH3 domain	11 of 221	0.55	0.0272
KW-0010	Activator	23 of 670	0.39	0.0126
KW-1017	Isopeptide bond	40 of 1715	0.22	0.0495
KW-0963	Cytoplasm	95 of 4979	0.14	0.0272
KW-0025	Alternative splicing	185 of 10225	0.11	4.83e-05
KW-0597	Phosphoprotein	144 of 8067	0.11	0.0126

Current Violent Behaviour (CVB)

Mapping

#	queryItem	stringId	preferredName	annotation
1	AGO2	9606.ENSP00000220592	AGO2	Protein argonaute-2; Required for RNA-mediated gene silencing (RNAi) by the RNA-induced silencing complex (RISC). The 'minimal RISC' appears to include AGO2 bound to a short guide RNA such as a microRNA (miRNA) or short interfering RNA (siRNA). These guide RNAs direct RISC to complementary mRNAs that are targets for RISC-mediated gene silencing. The precise mechanism of gene silencing depends on the degree of complementarity between the miRNA or siRNA and its target. Binding of RISC to a perfectly complementary mRNA generally results in silencing due to endonucleolytic cleavage of the [...]
3	ABCA2	9606.ENSP00000344155	ABCA2	ATP-binding cassette sub-family A member 2; Probable transporter, its natural substrate has not been found yet. May have a role in macrophage lipid metabolism and neural development; Belongs to the ABC transporter superfamily. ABCA family
4	ABCC5	9606.ENSP00000333926	ABCC5	Multidrug resistance-associated protein 5; Acts as a multispecific organic anion pump which can transport nucleotide analogs; Belongs to the ABC transporter superfamily. ABCC family. Conjugate transporter (TC 3.A.1.208) subfamily
5	ACOT7	9606.ENSP00000367086	ACOT7	Cytosolic acyl coenzyme A thioester hydrolase; Acyl-CoA thioesterases are a group of enzymes that catalyze the hydrolysis of acyl-CoAs to the free fatty acid and coenzyme A (CoASH), providing the potential to regulate intracellular levels of acyl-CoAs, free fatty acids and CoASH. May play an important physiological function in brain. May play a regulatory role by modulating the cellular levels of fatty acyl- CoA ligands for certain transcription factors as well as the substrates for fatty acid metabolizing enzymes, contributing to lipid homeostasis. Has broad specificity, active toward [...]
6	ACSF3	9606.ENSP00000479130	ACSF3	Acyl-CoA synthetase family member 3, mitochondrial; Catalyzes the initial reaction in intramitochondrial fatty acid synthesis, by activating malonate and methylmalonate, but not acetate, into their respective CoA thioester. May have some preference toward very-long-chain substrates; Acyl-CoA synthetase family
7	ADARB1	9606.ENSP00000353920	ADARB1	Double-stranded RNA-specific editase 1; Catalyzes the hydrolytic deamination of adenosine to inosine in double-stranded RNA (dsRNA) referred to as A-to-I RNA editing. This may affect gene expression and function in a number of ways that include mRNA translation by changing codons and hence the amino acid sequence of proteins; pre-mRNA splicing by altering splice site recognition sequences; RNA stability by changing sequences involved in nuclease recognition; genetic stability in the case of RNA virus genomes by changing sequences during viral RNA replication; and RNA structure-dependen [...]
8	ADCY10	9606.ENSP00000356825	ADCY10	Adenylate cyclase type 10; Catalyzes the formation of the signaling molecule cAMP. May function as sensor that mediates responses to changes in cellular bicarbonate and CO(2) levels. Has a critical role in mammalian spermatogenesis by producing the cAMP which regulates cAMP-responsive nuclear factors indispensable for sperm maturation in the epididymis. Induces capacitation, the maturational process that sperm undergo prior to fertilization (By similarity). Involved in ciliary beat regulation; Adenylate cyclases
9	ADCYAP1	9606.ENSP00000462647	ADCYAP1	Pituitary adenylate cyclase-activating polypeptide; Binding to its receptor activates G proteins and stimulates adenylate cyclase in pituitary cells. Promotes neuron projection development through the RAPGEF2/Rap1/B-Raf/ERK pathway. In chromaffin cells, induces long-lasting increase of intracellular calcium concentrations and neuroendocrine secretion (By similarity). Involved in the control of glucose homeostasis, induces insulin secretion by pancreatic beta cells (By similarity); Belongs to the glucagon family
10	AFF3	9606.ENSP00000386834	AFF3	AF4/FMR2 family member 3; Putative transcription activator that may function in lymphoid development and oncogenesis. Binds, in vitro, to double- stranded DNA; Belongs to the AF4 family
11	AIM2	9606.ENSP00000357112	AIM2	Interferon-inducible protein AIM2; Involved in innate immune response by recognizing cytosolic double-stranded DNA and inducing caspase-1-activating inflammasome formation in macrophages. Upon binding to DNA is thought to undergo oligomerization and to associate with PYCARD initiating the recruitment of caspase-1 precusrsor and processing of interleukin-1 beta and interleukin-18. Detects cytosolic dsDNA of viral and bacterial origin in a non-sequence-specific manner. Can also trigger PYCARD-dependent, caspase-1-independent cell death that involves caspase-8 (By similarity). Tumor suppr [...]
12	AKNAD1	9606.ENSP00000359018	AKNAD1	Protein AKNAD1; AKNA domain containing 1
13	AKT1	9606.ENSP00000451828	AKT1	RAC-alpha serine/threonine-protein kinase; AKT1 is one of 3 closely related serine/threonine- protein kinases (AKT1, AKT2 and AKT3) called the AKT kinase, and which regulate many processes including metabolism, proliferation, cell survival, growth and angiogenesis. This is mediated through serine and/or threonine phosphorylation of a range of downstream substrates. Over 100 substrate candidates have been reported so far, but for most of them, no isoform specificity has been reported. AKT is responsible of the regulation of glucose uptake by mediating insulin-induced translocation of th [...]
14	AKT3	9606.ENSP00000263826	AKT3	RAC-gamma serine/threonine-protein kinase; AKT3 is one of 3 closely related serine/threonine- protein kinases (AKT1, AKT2 and AKT3) called the AKT kinase, and which regulate many processes including metabolism, proliferation, cell survival, growth and angiogenesis. This is mediated through serine and/or threonine phosphorylation of a range of downstream substrates. Over 100 substrate candidates have been reported so far, but for most of them, no isoform specificity has been reported. AKT3 is the least studied AKT isoform. It plays an important role in brain development and is crucial f [...]
15	ALDH3A2	9606.ENSP00000345774	ALDH3A2	Fatty aldehyde dehydrogenase; Catalyzes the oxidation of long-chain aliphatic aldehydes to fatty acids. Active on a variety of saturated and unsaturated aliphatic aldehydes between 6 and 24 carbons in length. Responsible for conversion of the sphingosine 1-phosphate (S1P) degradation product hexadecenal to hexadecenoic acid
16	ANGPTL6	9606.ENSP00000253109	ANGPTL6	Angiopoietin-related protein 6; May play a role in the wound healing process. May promote epidermal proliferation, remodeling and regeneration. May promote the chemotactic activity of endothelial cells and induce neovascularization. May counteract high-fat diet-induced obesity and related insulin resistance through increased energy expenditure; Angiopoietin like
17	ANK2	9606.ENSP00000349588	ANK2	Ankyrin-2; In skeletal muscle, required for proper localization of DMD and DCTN4 and for the formation and/or stability of a special subset of microtubules associated with costameres and neuromuscular junctions. Attaches integral membrane proteins to cytoskeletal elements. Also binds to cytoskeletal proteins. Required for coordinate assembly of Na/Ca exchanger, Na/K ATPase and InsP3 receptor at sarcoplasmic reticulum sites in cardiomyocytes. Required for the coordinated expression of the Na/K ATPase, Na/Ca exchanger and beta-2-spectrin (SPTBN1) in the inner segment of rod photoreceptor [...]
18	ANKK1	9606.ENSP00000306678	ANKK1	Ankyrin repeat and kinase domain containing 1; Belongs to the protein kinase superfamily. TKL Ser/Thr protein kinase family
19	ANKRD9	9606.ENSP00000286918	ANKRD9	Ankyrin repeat domain-containing protein 9; Ankyrin repeat domain containing
20	ANO1	9606.ENSP00000347454	ANO1	Anoctamin-1; Calcium-activated chloride channel (CaCC) which plays a role in transepithelial anion transport and smooth muscle contraction. Required for the normal functioning of the interstitial cells of Cajal (ICCs) which generate electrical pacemaker activity in gastrointestinal smooth muscles. Acts as a major contributor to basal and stimulated chloride conductance in airway epithelial cells and plays an important role in tracheal cartilage development; Belongs to the anoctamin family
22	ANP32E	9606.ENSP00000463154	ANP32E	Acidic leucine-rich nuclear phosphoprotein 32 family member E; Histone chaperone that specifically mediates the genome- wide removal of histone H2A.Z/H2AFZ from the nucleosome: removes H2A.Z/H2AFZ from its normal sites of deposition, especially from enhancer and insulator regions. Not involved in deposition of H2A.Z/H2AFZ in the nucleosome. May stabilize the evicted H2A.Z/H2AFZ-H2B dimer, thus shifting the equilibrium towards dissociation and the off-chromatin state. Inhibits activity of protein phosphatase 2A (PP2A). Does not inhibit protein phosphatase 1. May play a role in cerebella [...]
23	ANXA9	9606.ENSP00000357943	ANXA9	Annexin A9; Low affinity receptor for acetylcholine known to be targeted by disease-causing pemphigus vulgaris antibodies in keratinocytes; Belongs to the annexin family
24	ARAP1	9606.ENSP00000377233	ARAP1	Arf-GAP with Rho-GAP domain, ANK repeat and PH domain-containing protein 1; Phosphatidylinositol 3,4,5-trisphosphate-dependent GTPase-activating protein that modulates actin cytoskeleton remodeling by regulating ARF and RHO family members. Is activated by phosphatidylinositol 3,4,5-trisphosphate (PtdIns(3,4,5)P3) binding. Can be activated by phosphatidylinositol 3,4-bisphosphate (PtdIns(3,4,5)P2) binding, albeit with lower efficiency. Has a preference for ARF1 and ARF5 (By similarity); Ankyrin repeat domain containing
25	ARHGAP44	9606.ENSP00000368994	ARHGAP44	Rho GTPase-activating protein 44; GTPase-activating protein (GAP) that stimulates the GTPase activity of Rho-type GTPases. Thereby, controls Rho-type GTPases cycling between their active GTP-bound and inactive GDP- bound states. May act as a GAP for CDC42 and RAC1. Endosomal recycling protein which, in association with SHANK3, is involved in synaptic plasticity. Promotes GRIA1 exocytosis from recycling endosomes and spine morphological changes associated to long-term potentiation; N-BAR domain containing
26	ARHGEF7	9606.ENSP00000364893	ARHGEF7	Rho guanine nucleotide exchange factor 7; Acts as a RAC1 guanine nucleotide exchange factor (GEF) and can induce membrane ruffling. Functions in cell migration, attachment and cell spreading. Promotes targeting of RAC1 to focal adhesions (By similarity). May function as a positive regulator of apoptosis. Downstream of NMDA receptors and CaMKK-CaMK1 signaling cascade, promotes the formation of spines and synapses in hippocampal neurons; Pleckstrin homology domain containing
27	ASAP1	9606.ENSP00000429900	ASAP1	Arf-GAP with SH3 domain, ANK repeat and PH domain-containing protein 1; Possesses phosphatidylinositol 4,5-bisphosphate- dependent GTPase-activating protein activity for ARF1 (ADP ribosylation factor 1) and ARF5 and a lesser activity towards ARF6. May coordinate membrane trafficking with cell growth or actin cytoskeleton remodeling by binding to both SRC and PIP2. May function as a signal transduction protein involved in the differentiation of fibroblasts into adipocytes and possibly other cell types (By similarity). Plays a role in ciliogenesis; Ankyrin repeat domain containing
29	ATOX1	9606.ENSP00000430598	ATOX1	Copper transport protein ATOX1; Binds and deliver cytosolic copper to the copper ATPase proteins. May be important in cellular antioxidant defense; Belongs to the ATX1 family
30	ATP2C1	9606.ENSP00000421326	ATP2C1	Calcium-transporting ATPase type 2C member 1; This magnesium-dependent enzyme catalyzes the hydrolysis of ATP coupled with the transport of the calcium; Belongs to the cation transport ATPase (P-type) (TC 3.A.3) family. Type IIA subfamily
31	AVPR1B	9606.ENSP00000356094	AVPR1B	Vasopressin V1b receptor; Receptor for arginine vasopressin. The activity of this receptor is mediated by G proteins which activate a phosphatidyl- inositol-calcium second messenger system; Arginine vasopressin and oxytocin receptors
33	B3GNTL1	9606.ENSP00000319979	B3GNTL1	UDP-GlcNAc:betaGal beta-1,3-N-acetylglucosaminyltransferase-like protein 1; Putative glycosyltransferase; Glycosyltransferase family 2
34	BAHD1	9606.ENSP00000396976	BAHD1	Bromo adjacent homology domain-containing 1 protein; Heterochromatin protein that acts as a transcription repressor and has the ability to promote the formation of large heterochromatic domains. May act by recruiting heterochromatin proteins such as CBX5 (HP1 alpha), HDAC5 and MBD1. Represses IGF2 expression by binding to its CpG-rich P3 promoter and recruiting heterochromatin proteins. At specific stages of Listeria infection, in complex with TRIM28, corepresses interferon- stimulated genes, including IFNL1, IFNL2 and IFNL3
35	BAIAP2	9606.ENSP00000316338	BAIAP2	Brain-specific angiogenesis inhibitor 1-associated protein 2; Adapter protein that links membrane-bound small G- proteins to cytoplasmic effector proteins. Necessary for CDC42-mediated reorganization of the actin cytoskeleton and for RAC1- mediated membrane ruffling. Involved in the regulation of the actin cytoskeleton by WASF family members and the Arp2/3 complex. Plays a role in neurite growth. Acts syngeristically with ENAH to promote filipodia formation. Plays a role in the reorganization of the actin cytoskeleton in response to bacterial infection. Participates in actin bundling [...]
36	BMPR1B	9606.ENSP00000401907	BMPR1B	Bone morphogenetic protein receptor type-1B; On ligand binding, forms a receptor complex consisting of two type II and two type I transmembrane serine/threonine kinases. Type II receptors phosphorylate and activate type I receptors which autophosphorylate, then bind and activate SMAD transcriptional regulators. Receptor for BMP7/OP-1 and GDF5. Positively regulates chondrocyte differentiation through GDF5 interaction; Belongs to the protein kinase superfamily. TKL Ser/Thr protein kinase family. TGFB receptor subfamily
37	BRD4	9606.ENSP00000263377	BRD4	Bromodomain-containing protein 4; Chromatin reader protein that recognizes and binds acetylated histones and plays a key role in transmission of epigenetic memory across cell divisions and transcription regulation. Remains associated with acetylated chromatin throughout the entire cell cycle and provides epigenetic memory for postmitotic G1 gene transcription by preserving acetylated chromatin status and maintaining high-order chromatin structure. During interphase, plays a key role in regulating the transcription of signal-inducible genes by associating with the P- TEFb complex and re [...]
38	BSN	9606.ENSP00000296452	BSN	Protein bassoon; Is thought to be involved in the organization of the cytomatrix at the nerve terminals active zone (CAZ) which regulates neurotransmitter release. Seems to act through binding to ERC2/CAST1. Essential in regulated neurotransmitter release from a subset of brain glutamatergic synapses. Involved in the formation of the retinal photoreceptor ribbon synapses (By similarity); Zinc fingers
39	BTK	9606.ENSP00000483570	BTK	Tyrosine-protein kinase BTK; Non-receptor tyrosine kinase indispensable for B lymphocyte development, differentiation and signaling. Binding of antigen to the B-cell antigen receptor (BCR) triggers signaling that ultimately leads to B-cell activation. After BCR engagement and activation at the plasma membrane, phosphorylates PLCG2 at several sites, igniting the downstream signaling pathway through calcium mobilization, followed by activation of the protein kinase C (PKC) family members. PLCG2 phosphorylation is performed in close cooperation with the adapter protein B-cell linker prote [...]
40	CACNA2D1	9606.ENSP00000349320	CACNA2D1	Voltage-dependent calcium channel subunit alpha-2/delta-1; The alpha-2/delta subunit of voltage-dependent calcium channels regulates calcium current density and activation/inactivation kinetics of the calcium channel. Plays an important role in excitation-contraction coupling (By similarity)
41	CACNB2	9606.ENSP00000320025	CACNB2	Voltage-dependent L-type calcium channel subunit beta-2; The beta subunit of voltage-dependent calcium channels contributes to the function of the calcium channel by increasing peak calcium current, shifting the voltage dependencies of activation and inactivation, modulating G protein inhibition and controlling the alpha-1 subunit membrane targeting
42	CACNG4	9606.ENSP00000262138	CACNG4	Voltage-dependent calcium channel gamma-4 subunit; Regulates the trafficking and gating properties of AMPA- selective glutamate receptors (AMPARs). Promotes their targeting to the cell membrane and synapses and modulates their gating properties by slowing their rates of activation, deactivation and desensitization and by mediating their resensitization. Does not show subunit-specific AMPA receptor regulation and regulates all AMPAR subunits. Thought to stabilize the calcium channel in an inactivated (closed) state
43	CARD11	9606.ENSP00000380150	CARD11	Caspase recruitment domain-containing protein 11; Involved in the costimulatory signal essential for T- cell receptor (TCR)-mediated T-cell activation. Its binding to DPP4 induces T-cell proliferation and NF-kappa-B activation in a T-cell receptor/CD3-dependent manner. Activates NF-kappa-B via BCL10 and IKK. Stimulates the phosphorylation of BCL10. Also activates the TORC1 signaling pathway; CBM complex
44	CARNS1	9606.ENSP00000389009	CARNS1	Carnosine synthase 1; Catalyzes the synthesis of carnosine and homocarnosine. Carnosine is synthesized more efficiently than homocarnosine
45	CASK	9606.ENSP00000367408	CASK	Peripheral plasma membrane protein CASK; Multidomain scaffolding protein with a role in synaptic transmembrane protein anchoring and ion channel trafficking. Contributes to neural development and regulation of gene expression via interaction with the transcription factor TBR1. Binds to cell-surface proteins, including amyloid precursor protein, neurexins and syndecans. May mediate a link between the extracellular matrix and the actin cytoskeleton via its interaction with syndecan and with the actin/spectrin-binding protein 4.1; In the N-terminal section; belongs to the protein kinase s [...]
46	CCND1	9606.ENSP00000227507	CCND1	G1/S-specific cyclin-D1; Regulatory component of the cyclin D1-CDK4 (DC) complex that phosphorylates and inhibits members of the retinoblastoma (RB) protein family including RB1 and regulates the cell-cycle during G(1)/S transition. Phosphorylation of RB1 allows dissociation of the transcription factor E2F from the RB/E2F complex and the subsequent transcription of E2F target genes which are responsible for the progression through the G(1) phase. Hypophosphorylates RB1 in early G(1) phase. Cyclin D-CDK4 complexes are major integrators of various mitogenenic and antimitogenic signals. A [...]
47	CCPG1	9606.ENSP00000403400	CCPG1	Cell cycle progression protein 1; Acts as an assembly platform for Rho protein signaling complexes. Limits guanine nucleotide exchange activity of MCF2L toward RHOA, which results in an inhibition of both its transcriptional activation ability and its transforming activity. Does not inhibit activity of MCF2L toward CDC42, or activity of MCF2 toward either RHOA or CDC42 (By similarity). May be involved in cell cycle regulation; Belongs to the CCPG1 family

#	queryItem	stringId	preferredName	annotation
48	CD200R1	9606.ENSP00000311035	CD200R1	Cell surface glycoprotein CD200 receptor 1; Inhibitory receptor for the CD200/OX2 cell surface glycoprotein. Limits inflammation by inhibiting the expression of proinflammatory molecules including TNF-alpha, interferons, and inducible nitric oxide synthase (iNOS) in response to selected stimuli. Also binds to HHV-8 K14 viral CD200 homolog with identical affinity and kinetics as the host CD200; Belongs to the CD200R family
49	CDC14B	9606.ENSP00000364389	CDC14B	Dual specificity protein phosphatase CDC14B; Dual-specificity phosphatase involved in DNA damage response. Essential regulator of the G2 DNA damage checkpoint: following DNA damage, translocates to the nucleus and dephosphorylates FZR1/CDH1, a key activator of the anaphase promoting complex/cyclosome (APC/C). Dephosphorylates SIRT2 around early anaphase. Dephosphorylation of FZR1/CDH1 activates the APC/C, leading to the ubiquitination of PLK1, preventing entry into mitosis. Preferentially dephosphorylates proteins modified by proline-directed kinases; Belongs to the protein-tyrosine ph [...]
50	CDC42EP4	9606.ENSP00000338258	CDC42EP4	Cdc42 effector protein 4; Probably involved in the organization of the actin cytoskeleton. May act downstream of CDC42 to induce actin filament assembly leading to cell shape changes. Induces pseudopodia formation, when overexpressed in fibroblasts; Belongs to the BORG/CEP family
51	CDH26	9606.ENSP00000339390	CDH26	Cadherin-like protein 26; Cadherins are calcium-dependent cell adhesion proteins. They preferentially interact with themselves in a homophilic manner in connecting cells; cadherins may thus contribute to the sorting of heterogeneous cell types
52	CELF1	9606.ENSP00000435926	CELF1	CUGBP Elav-like family member 1; RNA-binding protein implicated in the regulation of several post-transcriptional events. Involved in pre-mRNA alternative splicing, mRNA translation and stability. Mediates exon inclusion and/or exclusion in pre-mRNA that are subject to tissue-specific and developmentally regulated alternative splicing. Specifically activates exon 5 inclusion of cardiac isoforms of TNNT2 during heart remodeling at the juvenile to adult transition. Acts as both an activator and repressor of a pair of coregulated exons: promotes inclusion of the smooth muscle (SM) exon bu [...]
53	CELF4	9606.ENSP00000410584	CELF4	CUGBP Elav-like family member 4; RNA-binding protein implicated in the regulation of pre- mRNA alternative splicing. Mediates exon inclusion and/or exclusion in pre-mRNA that are subject to tissue-specific and developmentally regulated alternative splicing. Specifically activates exon 5 inclusion of cardiac isoforms of TNNT2 during heart remodeling at the juvenile to adult transition. Promotes exclusion of both the smooth muscle (SM) and non-muscle (NM) exons in actinin pre-mRNAs. Activates the splicing of MAPT/Tau exon 10. Binds to muscle-specific splicing enhancer (MSE) intronic site [...]
54	CERS6	9606.ENSP00000376453	CERS6	Ceramide synthase 6; May be involved in sphingolipid synthesis or its regulation; CERS class homeoboxes
55	CFAP46	9606.ENSP00000357575	CFAP46	Cilia- and flagella-associated protein 46; As part of the central apparatus of the cilium axoneme plays a role in cilium movement; Cilia and flagella associated
56	CIITA	9606.ENSP00000485010	CIITA	MHC class II transactivator; Essential for transcriptional activity of the HLA class II promoter; activation is via the proximal promoter. No DNA binding of in vitro translated CIITA was detected. May act in a coactivator-like fashion through protein-protein interactions by contacting factors binding to the proximal MHC class II promoter, to elements of the transcription machinery, or both. Alternatively it may activate HLA class II transcription by modifying proteins that bind to the MHC class II promoter. Also mediates enhanced MHC class I transcription; the promoter element requirem [...]
57	CIPC	9606.ENSP00000355319	CIPC	CLOCK-interacting pacemaker; Transcriptional repressor which may act as a negative- feedback regulator of CLOCK-ARNTL/BMAL1 transcriptional activity in the circadian-clock mechanism. May stimulate ARNTL/BMAL1- dependent phosphorylation of CLOCK. However, the physiogical relevance of these observations is unsure, since experiments in an animal model showed that CIPC is not critially required for basic circadian clock
58	CLDN5	9606.ENSP00000385477	CLDN5	Claudin-5; Plays a major role in tight junction-specific obliteration of the intercellular space; Claudins
59	CNPY3	9606.ENSP00000361926	CNPY3	Protein canopy homolog 3; Toll-like receptor (TLR)-specific co-chaperone for HSP90B1. Required for proper TLR folding, except that of TLR3, and hence controls TLR exit from the endoplasmic reticulum. Consequently, required for both innate and adaptive immune responses (By similarity); Belongs to the canopy family
60	COL15A1	9606.ENSP00000364140	COL15A1	Collagen alpha-1(XV) chain; Structural protein that stabilizes microvessels and muscle cells, both in heart and in skeletal muscle; Collagen proteoglycans
61	CPLX1	9606.ENSP00000305613	CPLX1	Complexin-1; Positively regulates a late step in exocytosis of various cytoplasmic vesicles, such as synaptic vesicles and other secretory vesicles. Organizes the SNAREs into a cross-linked zigzag topology that, when interposed between the vesicle and plasma membranes, is incompatible with fusion, thereby preventing SNAREs from releasing neurotransmitters until an action potential arrives at the synapse. Also involved in glucose-induced secretion of insulin by pancreatic beta-cells. Essential for motor behavior
62	CRABP2	9606.ENSP00000482841	CRABP2	Cellular retinoic acid-binding protein 2; Transports retinoic acid to the nucleus. Regulates the access of retinoic acid to the nuclear retinoic acid receptors; Fatty acid binding protein family
63	CREB3L1	9606.ENSP00000481956	CREB3L1	Cyclic AMP-responsive element-binding protein 3-like protein 1; Transcription factor involved in unfolded protein response (UPR). Binds the DNA consensus sequence 5'-GTGXGCXGC-3'. In the absence of endoplasmic reticulum (ER) stress, inserted into ER membranes, with N-terminal DNA-binding and transcription activation domains oriented toward the cytosolic face of the membrane. In response to ER stress, transported to the Golgi, where it is cleaved in a site-specific manner by resident proteases S1P/MBTPS1 and S2P/MBTPS2. The released N-terminal cytosolic domain is translocated to the nuc [...]
64	CRIP2	9606.ENSP00000426119	CRIP2	Cysteine rich protein 2; LIM domain containing
65	CTIF	9606.ENSP00000372459	CTIF	CBP80/20-dependent translation initiation factor; Specifically required for the pioneer round of mRNA translation mediated by the cap-binding complex (CBC), that takes place during or right after mRNA export via the nuclear pore complex (NPC). Acts via its interaction with the NCBP1/CBP80 component of the CBC complex and recruits the 40S small subunit of the ribosome via eIF3. In contrast, it is not involved in steady state translation, that takes place when the CBC complex is replaced by cytoplasmic cap-binding protein eIF4E. Also required for nonsense-mediated mRNA decay (NMD), the p [...]
66	CXADR	9606.ENSP00000284878	CXADR	Coxsackievirus and adenovirus receptor; Component of the epithelial apical junction complex that may function as a homophilic cell adhesion molecule and is essential for tight junction integrity. Also involved in transepithelial migration of leukocytes through adhesive interactions with JAML a transmembrane protein of the plasma membrane of leukocytes. The interaction between both receptors also mediates the activation of gamma-delta T-cells, a subpopulation of T-cells residing in epithelia and involved in tissue homeostasis and repair. Upon epithelial CXADR-binding, JAML induces downs [...]
68	DAOA	9606.ENSP00000483757	DAOA	D-amino acid oxidase activator; Seems to activate D-amino acid oxidase
69	DAXX	9606.ENSP00000363668	DAXX	Death domain-associated protein 6; Transcription corepressor known to repress transcriptional potential of several sumoylated transcription factors. Down-regulates basal and activated transcription. Its transcription repressor activity is modulated by recruiting it to subnuclear compartments like the nucleolus or PML/POD/ND10 nuclear bodies through interactions with MCSR1 and PML, respectively. Seems to regulate transcription in PML/POD/ND10 nuclear bodies together with PML and may influence TNFRSF6-dependent apoptosis thereby. Inhibits transcriptional activation of PAX3 and ETS1 throu [...]
70	DICER1	9606.ENSP00000437256	DICER1	Endoribonuclease Dicer; Double-stranded RNA (dsRNA) endoribonuclease playing a central role in short dsRNA-mediated post-transcriptional gene silencing. Cleaves naturally occurring long dsRNAs and short hairpin pre-microRNAs (miRNA) into fragments of twenty-one to twenty-three nucleotides with 3' overhang of two nucleotides, producing respectively short interfering RNAs (siRNA) and mature microRNAs. SiRNAs and miRNAs serve as guide to direct the RNA- induced silencing complex (RISC) to complementary RNAs to degrade them or prevent their translation. Gene silencing mediated by siRNAs, a [...]
71	DIO3	9606.ENSP00000427336	DIO3	Thyroxine 5-deiodinase; Responsible for the deiodination of T4 (3,5,3',5'- tetraiodothyronine) into RT3 (3,3',5'-triiodothyronine) and of T3 (3,5,3'-triiodothyronine) into T2 (3,3'-diiodothyronine). RT3 and T2 are inactive metabolites. May play a role in preventing premature exposure of developing fetal tissues to adult levels of thyroid hormones. Can regulate circulating fetal thyroid hormone concentrations throughout gestation. Essential role for regulation of thyroid hormone inactivation during embryological development
72	DLGAP2	9606.ENSP00000400258	DLGAP2	Disks large-associated protein 2; May play a role in the molecular organization of synapses and neuronal cell signaling. Could be an adapter protein linking ion channel to the subsynaptic cytoskeleton. May induce enrichment of PSD-95/SAP90 at the plasma membrane; Belongs to the SAPAP family
74	DNAJB6	9606.ENSP00000262177	DNAJB6	DnaJ homolog subfamily B member 6; Plays an indispensable role in the organization of KRT8/KRT18 filaments. Acts as an endogenous molecular chaperone for neuronal proteins including huntingtin. Suppresses aggregation and toxicity of polyglutamine-containing, aggregation-prone proteins. Isoform B but not isoform A inhibits huntingtin aggregation. Has a stimulatory effect on the ATPase activity of HSP70 in a dose-dependent and time-dependent manner and hence acts as a co-chaperone of HSP70. Also reduces cellular toxicity and caspase-3 activity; DNAJ heat shock proteins
75	DNHD1	9606.ENSP00000254579	DNHD1	Dynein heavy chain domain 1
76	DUSP10	9606.ENSP00000355866	DUSP10	Dual specificity protein phosphatase 10; Protein phosphatase involved in the inactivation of MAP kinases. Has a specificity for the MAPK11/MAPK12/MAPK13/MAPK14 subfamily. It preferably dephosphorylates p38
77	DUSP2	9606.ENSP00000288943	DUSP2	Dual specificity protein phosphatase 2; Regulates mitogenic signal transduction by dephosphorylating both Thr and Tyr residues on MAP kinases ERK1 and ERK2; Belongs to the protein-tyrosine phosphatase family. Non-receptor class dual specificity subfamily
78	DYSF	9606.ENSP00000386881	DYSF	Dysferlin; Key calcium ion sensor involved in the Ca(2+)-triggered synaptic vesicle-plasma membrane fusion. Plays a role in the sarcolemma repair mechanism of both skeletal muscle and cardiomyocytes that permits rapid resealing of membranes disrupted by mechanical stress (By similarity); Ferlin family
79	ECHDC3	9606.ENSP00000368517	ECHDC3	Enoyl-CoA hydratase domain-containing protein 3, mitochondrial; enoyl-CoA hydratase domain containing 3; Belongs to the enoyl-CoA hydratase/isomerase family
81	EIF3G	9606.ENSP00000253108	EIF3G	Eukaryotic translation initiation factor 3 subunit G; RNA-binding component of the eukaryotic translation initiation factor 3 (eIF-3) complex, which is required for several steps in the initiation of protein synthesis. The eIF-3 complex associates with the 40S ribosome and facilitates the recruitment of eIF-1, eIF-1A, eIF-2:GTP:methionyl-tRNAi and eIF-5 to form the 43S pre- initiation complex (43S PIC). The eIF-3 complex stimulates mRNA recruitment to the 43S PIC and scanning of the mRNA for AUG recognition. The eIF-3 complex is also required for disassembly and recycling of post-termi [...]
82	EXD3	9606.ENSP00000340474	EXD3	Exonuclease mut-7 homolog; Possesses 3'-5' exoribonuclease activity. Required for 3'-end trimming of AGO1-bound miRNAs (By similarity)
83	EXTL3	9606.ENSP00000220562	EXTL3	Exostosin-like 3; Glycosyltransferase which regulates the biosynthesis of heparan sulfate (HS). Important for both skeletal development and hematopoiesis, through the formation of HS proteoglycans (HSPGs). Required for the function of REG3A in regulating keratinocyte proliferation and differentiation
84	FAM131A	9606.ENSP00000373360	FAM131A	Protein FAM131A; Family with sequence similarity 131 member A; Belongs to the FAM131 family
85	FAM163B	9606.ENSP00000349336	FAM163B	Protein FAM163B; Family with sequence similarity 163 member B
86	FAM169B	9606.ENSP00000453554	FAM169B	Protein FAM169B; Family with sequence similarity 169 member B; Belongs to the FAM169 family
87	FAM174B	9606.ENSP00000329040	FAM174B	Membrane protein FAM174B; Family with sequence similarity 174 member B
88	FAM221A	9606.ENSP00000342576	FAM221A	Protein FAM221A; Family with sequence similarity 221 member A; Belongs to the FAM221 family
89	FAM53B	9606.ENSP00000338532	FAM53B	Protein FAM53B; Acts as a regulator of Wnt signaling pathway by regulating beta-catenin (CTNNB1) nuclear localization
90	FBXL16	9606.ENSP00000380746	FBXL16	F-box/LRR-repeat protein 16; Substrate-recognition component of the SCF (SKP1-CUL1-F- box protein)-type E3 ubiquitin ligase complex; F-box and leucine rich repeat proteins
91	FKBP10	9606.ENSP00000317232	FKBP10	Peptidyl-prolyl cis-trans isomerase FKBP10; PPIases accelerate the folding of proteins during protein synthesis; EF-hand domain containing
92	FMNL1	9606.ENSP00000329219	FMNL1	Formin-like protein 1; May play a role in the control of cell motility and survival of macrophages (By similarity). Plays a role in the regulation of cell morphology and cytoskeletal organization. Required in the cortical actin filament dynamics and cell shape; Armadillo-like helical domain containing
93	FOXP3	9606.ENSP00000365380	FOXP3	Forkhead box protein P3; Transcriptional regulator which is crucial for the development and inhibitory function of regulatory T-cells (Treg). Plays an essential role in maintaining homeostasis of the immune system by allowing the acquisition of full suppressive function and stability of the Treg lineage, and by directly modulating the expansion and function of conventional T-cells. Can act either as a transcriptional repressor or a transcriptional activator depending on its interactions with other transcription factors, histone acetylases and deacetylases. The suppressive activity of T [...]
94	GALE	9606.ENSP00000483375	GALE	UDP-glucose 4-epimerase; Catalyzes two distinct but analogous reactions: the reversible epimerization of UDP-glucose to UDP-galactose and the reversible epimerization of UDP-N-acetylglucosamine to UDP-N- acetylgalactosamine. The reaction with UDP-Gal plays a critical role in the Leloir pathway of galactose catabolism in which galactose is converted to the glycolytic intermediate glucose 6- phosphate. It contributes to the catabolism of dietary galactose and enables the endogenous biosynthesis of both UDP-Gal and UDP- GalNAc when exogenous sources are limited. Both UDP-sugar interconver [...]
95	GLI2	9606.ENSP00000390436	GLI2	Zinc finger protein GLI2; Functions as transcription regulator in the hedgehog (Hh) pathway. Functions as transcriptional activator. May also function as transcriptional repressor (By similarity). Requires STK36 for full transcriptional activator activity. Required for normal embryonic development; Zinc fingers C2H2-type
96	GNB1L	9606.ENSP00000331313	GNB1L	G protein subunit beta 1 like; WD repeat domain containing
97	GNG7	9606.ENSP00000371594	GNG7	Guanine nucleotide-binding protein G(I)/G(S)/G(O) subunit gamma-7; Guanine nucleotide-binding proteins (G proteins) are involved as a modulator or transducer in various transmembrane signaling systems. The beta and gamma chains are required for the GTPase activity, for replacement of GDP by GTP, and for G protein- effector interaction. Plays a role in the regulation of adenylyl cyclase signaling in certain regions of the brain. Plays a role in the formation or stabilzation of a G protein heterotrimer (G(olf) subunit alpha-beta-gamma-7) that is required for adenylyl cyclase activity in [...]
99	GPR17	9606.ENSP00000442982	GPR17	Uracil nucleotide/cysteinyl leukotriene receptor; Dual specificity receptor for uracil nucleotides and cysteinyl leukotrienes (CysLTs). Signals through G(i) and inhibition of adenylyl cyclase. May mediate brain damage by nucleotides and CysLTs following ischemia; Belongs to the G-protein coupled receptor 1 family
100	GPR173	9606.ENSP00000331600	GPR173	Probable G-protein coupled receptor 173; Orphan receptor; G protein-coupled receptors, Class A orphans
101	GPR68	9606.ENSP00000434045	GPR68	Ovarian cancer G-protein coupled receptor 1; Proton-sensing receptor involved in pH homeostasis. May represents an osteoblastic pH sensor regulating cell-mediated responses to acidosis in bone. Mediates its action by association with G proteins that stimulates inositol phosphate (IP) production or Ca(2+) mobilization. The receptor is almost silent at pH 7.8 but fully activated at pH 6.8. Function also as a metastasis suppressor gene in prostate cancer (By similarity); Belongs to the G-protein coupled receptor 1 family
102	GPSM1	9606.ENSP00000392828	GPSM1	G-protein-signaling modulator 1; Guanine nucleotide dissociation inhibitor (GDI) which functions as a receptor-independent activator of heterotrimeric G- protein signaling. Keeps G(i/o) alpha subunit in its GDP-bound form thus uncoupling heterotrimeric G-proteins signaling from G protein-coupled receptors. Controls spindle orientation and asymmetric cell fate of cerebral cortical progenitors. May also be involved in macroautophagy in intestinal cells. May play a role in drug addiction; Belongs to the GPSM family
103	GRB14	9606.ENSP00000263915	GRB14	Growth factor receptor-bound protein 14; Adapter protein which modulates coupling of cell surface receptor kinases with specific signaling pathways. Binds to, and suppresses signals from, the activated insulin receptor (INSR). Potent inhibitor of insulin-stimulated MAPK3 phosphorylation. Plays a critical role regulating PDPK1 membrane translocation in response to insulin stimulation and serves as an adapter protein to recruit PDPK1 to activated insulin receptor, thus promoting PKB/AKT1 phosphorylation and transduction of the insulin signal; Pleckstrin homology domain containing
104	GRHL1	9606.ENSP00000324693	GRHL1	Grainyhead-like protein 1 homolog; Transcription factor involved in epithelial development. Binds directly to the consensus DNA sequence 5'-AACCGGTT-3'. Important regulator of DSG1 in the context of hair anchorage and epidermal differentiation, participates in the maintenance of the skin barrier. There is no genetic interaction with GRHL3, no functional cooperativity due to diverse target gene selectivity during epithelia development (By similarity). Isoform 1 may function as an activator and isoform 2 as a repressor in tissues where both forms are expressed

Current Violent Behaviour (CVB)

#	queryItem	stringId	preferredName	annotation
105	GSAP	9606.ENSP00000257626	GSAP	Gamma-secretase-activating protein; Regulator of gamma-secretase activity, which specifically activates the production of amyloid-beta protein (amyloid-beta protein 40 and amyloid-beta protein 42), without affecting the cleavage of other gamma-secretase targets such has Notch. The gamma-secretase complex is an endoprotease complex that catalyzes the intramembrane cleavage of integral membrane proteins such as Notch receptors and APP (amyloid-beta precursor protein). Specifically promotes the gamma-cleavage of APP CTF-alpha (also named APP-CTF) by the gamma-secretase complex to generate [...]
106	GTF2I	9606.ENSP00000460070	GTF2I	General transcription factor II-I; Interacts with the basal transcription machinery by coordinating the formation of a multiprotein complex at the C-FOS promoter, and linking specific signal responsive activator complexes. Promotes the formation of stable high-order complexes of SRF and PHOX1 and interacts cooperatively with PHOX1 to promote serum-inducible transcription of a reporter gene deriven by the C- FOS serum response element (SRE). Acts as a coregulator for USF1 by binding independently two promoter elements, a pyrimidine-rich initiator (Inr) and an upstream E-box. Required fo [...]
107	GTF3C1	9606.ENSP00000348510	GTF3C1	General transcription factor 3C polypeptide 1; Required for RNA polymerase III-mediated transcription. Component of TFIIIC that initiates transcription complex assembly on tRNA and is required for transcription of 5S rRNA and other stable nuclear and cytoplasmic RNAs. Binds to the box B promoter element; General transcription factors
108	GTSF1	9606.ENSP00000446485	GTSF1	Gametocyte-specific factor 1; Required for spermatogenesis and is involved in the suppression of retrotransposon transcription in male germ cells
109	GUCY2D	9606.ENSP00000254854	GUCY2D	Retinal guanylyl cyclase 1; Probably plays a specific functional role in the rods and/or cones of photoreceptors. It may be the enzyme involved in the resynthesis of cGMP required for recovery of the dark state after phototransduction; Guanylate cyclase receptors
110	H2AFY	9606.ENSP00000423563	H2AFY	Core histone macro-H2A.1; Variant histone H2A which replaces conventional H2A in a subset of nucleosomes where it represses transcription. Nucleosomes wrap and compact DNA into chromatin, limiting DNA accessibility to the cellular machineries which require DNA as a template. Histones thereby play a central role in transcription regulation, DNA repair, DNA replication and chromosomal stability. DNA accessibility is regulated via a complex set of post-translational modifications of histones, also called histone code, and nucleosome remodeling. Involved in stable X chromosome inactivation [...]
111	HDAC10	9606.ENSP00000216271	HDAC10	Histone deacetylase 10; Responsible for the deacetylation of lysine residues on the N-terminal part of the core histones (H2A, H2B, H3 and H4). Histone deacetylation gives a tag for epigenetic repression and plays an important role in transcriptional regulation, cell cycle progression and developmental events. Histone deacetylases act via the formation of large multiprotein complexes
112	HELZ	9606.ENSP00000351524	HELZ	Probable helicase with zinc finger domain; May act as a helicase that plays a role in RNA metabolism in multiple tissues and organs within the developing embryo; Belongs to the DNA2/NAM7 helicase family
113	HELZ2	9606.ENSP00000417401	HELZ2	Helicase with zinc finger domain 2; Helicase that acts as a transcriptional coactivator for a number of nuclear receptors including PPARA, PPARG, THRA, THRB and RXRA; Belongs to the DNA2/NAM7 helicase family
115	HOXB9	9606.ENSP00000309439	HOXB9	Homeobox protein Hox-B9; Sequence-specific transcription factor which is part of a developmental regulatory system that provides cells with specific positional identities on the anterior-posterior axis; Belongs to the Abd-B homeobox family
116	HSPA12B	9606.ENSP00000254963	HSPA12B	Heat shock protein family A member 12B; Belongs to the heat shock protein 70 family
119	IGF1	9606.ENSP00000302665	IGF1	Insulin-like growth factor I; The insulin-like growth factors, isolated from plasma, are structurally and functionally related to insulin but have a much higher growth-promoting activity. May be a physiological regulator of [1-14C]-2-deoxy-D-glucose (2DG) transport and glycogen synthesis in osteoblasts. Stimulates glucose transport in bone-derived osteoblastic (PyMS) cells and is effective at much lower concentrations than insulin, not only regarding glycogen and DNA synthesis but also with regard to enhancing glucose uptake. May play a role in synapse maturation. Ca(2+)-dependent exoc [...]
121	IGSF21	9606.ENSP00000251296	IGSF21	Immunoglobulin superfamily member 21; Immunoglobulin like domain containing
122	IL34	9606.ENSP00000397863	IL34	Interleukin-34; Cytokine that promotes the proliferation, survival and differentiation of monocytes and macrophages. Promotes the release of proinflammatory chemokines, and thereby plays an important role in innate immunity and in inflammatory processes. Plays an important role in the regulation of osteoclast proliferation and differentiation, and in the regulation of bone resorption. Signaling via CSF1R and its downstream effectors stimulates phosphorylation of MAPK1/ERK2 AND MAPK3/ERK1; Belongs to the IL-34 family
123	IRF2BP2	9606.ENSP00000355568	IRF2BP2	Interferon regulatory factor 2-binding protein 2; Acts as a transcriptional corepressor in a IRF2- dependent manner; this repression is not mediated by histone deacetylase activities. Represses the NFAT1-dependent transactivation of NFAT-responsive promoters. Acts as a coactivator of VEGFA expression in cardiac and skeletal muscle; Belongs to the IRF2BP family
124	IRF8	9606.ENSP00000268638	IRF8	Interferon regulatory factor 8; Plays a role as a transcriptional activator or repressor. Specifically binds to the upstream regulatory region of type I IFN and IFN-inducible MHC class I genes (the interferon consensus sequence (ICS)). Plays a negative regulatory role in cells of the immune system. Involved in CD8(+) dendritic cell differentiation by forming a complex with the BATF-JUNB heterodimer in immune cells, leading to recognition of AICE sequence (5'-TGAnTCA/GAAA-3'), an immune-specific regulatory element, followed by cooperative binding of BATF and IRF8 and activation of genes [...]
125	IRX1	9606.ENSP00000305244	IRX1	Iroquois-class homeodomain protein IRX-1; TALE class homeoboxes and pseudogenes
126	JADE1	9606.ENSP00000226319	JADE1	Protein Jade-1; Component of the HBO1 complex which has a histone H4- specific acetyltransferase activity, a reduced activity toward histone H3 and is responsible for the bulk of histone H4 acetylation in vivo. Transcriptional coactivator, it may also promote acetylation of nucleosomal histone H4 by KAT5. Promotes apoptosis. May act as a renal tumor suppressor. Negatively regulates canonical Wnt signaling; at least in part, cooperates with NPHP4 in this function; PHD finger proteins
127	JAKMIP1	9606.ENSP00000386711	JAKMIP1	Janus kinase and microtubule-interacting protein 1; Associates with microtubules and may play a role in the microtubule-dependent transport of the GABA-B receptor. May play a role in JAK1 signaling and regulate microtubule cytoskeleton rearrangements; Belongs to the JAKMIP family
128	KAT2A	9606.ENSP00000225916	KAT2A	Histone acetyltransferase KAT2A; Functions as a histone acetyltransferase (HAT) to promote transcriptional activation. Acetylation of histones gives a specific tag for epigenetic transcription activation. Has significant histone acetyltransferase activity with core histones, but not with nucleosome core particles. Also acetylates non- histone proteins, such as CEBPB. Component of the ATAC complex, a complex with histone acetyltransferase activity on histones H3 and H4. In case of HIV-1 infection, it is recruited by the viral protein Tat. Regulates Tat's transactivating activity and may [...]
129	KAZN	9606.ENSP00000365198	KAZN	Kazrin; Component of the cornified envelope of keratinocytes. May be involved in the interplay between adherens junctions and desmosomes. The function in the nucleus is not known; Sterile alpha motif domain containing
130	KCNE4	9606.ENSP00000281830	KCNE4	Potassium voltage-gated channel subfamily E member 4; Ancillary protein that assembles as a beta subunit with a voltage-gated potassium channel complex of pore-forming alpha subunits. Modulates the gating kinetics and enhances stability of the channel complex. May associate with KCNQ1/KVLTQ1 and inhibit potassium current; Belongs to the potassium channel KCNE family
131	KCNG2	9606.ENSP00000315654	KCNG2	Potassium voltage-gated channel subfamily G member 2; Potassium channel subunit. Modulates channel activity by shifting the threshold and the half-maximal activation to more negative values; Belongs to the potassium channel family. G (TC 1.A.1.2) subfamily. Kv6.2/KCNG2 sub-subfamily
132	KCNK17	9606.ENSP00000362328	KCNK17	Potassium channel subfamily K member 17; Outward rectifying potassium channel. Produces rapidly activating and non-inactivating outward rectifier K(+) currents; Potassium two pore domain channel subfamily K
133	KIF21A	9606.ENSP00000354878	KIF21A	Kinesin-like protein KIF21A; Microtubule-binding motor protein probably involved in neuronal axonal transport. In vitro, has a plus-end directed motor activity (By similarity); Kinesins
134	KIFC3	9606.ENSP00000368976	KIFC3	Kinesin-like protein KIFC3; Minus-end microtubule-dependent motor protein. Involved in apically targeted transport (By similarity). Required for zonula adherens maintenance; Belongs to the TRAFAC class myosin-kinesin ATPase superfamily. Kinesin family
135	KLRG2	9606.ENSP00000339356	KLRG2	Killer cell lectin like receptor G2; C-type lectin domain containing
136	KRT7	9606.ENSP00000329243	KRT7	Keratin, type II cytoskeletal 7; Blocks interferon-dependent interphase and stimulates DNA synthesis in cells. Involved in the translational regulation of the human papillomavirus type 16 E7 mRNA (HPV16 E7); Keratins, type II
137	LBH	9606.ENSP00000378733	LBH	Protein LBH; Transcriptional activator which may act in mitogen- activated protein kinase signaling pathway; Belongs to the LBH family
138	LGALS14	9606.ENSP00000353893	LGALS14	Placental protein 13-like; Binds beta-galactoside and lactose. Strong inducer of T- cell apoptosis; Galectins
139	LGALS16	9606.ENSP00000375904	LGALS16	Galectin-16; Binds lactose with high affinity. Strong inducer of T- cell apoptosis; Galectins
140	LHPP	9606.ENSP00000357835	LHPP	Phospholysine phosphohistidine inorganic pyrophosphate phosphatase; Phosphatase that hydrolyzes imidodiphosphate, 3- phosphohistidine and 6-phospholysine. Has broad substrate specificity and can also hydrolyze inorganic diphosphate, but with lower efficiency (By similarity); Belongs to the HAD-like hydrolase superfamily
141	LMO1	9606.ENSP00000338207	LMO1	Rhombotin-1; May be involved in gene regulation within neural lineage cells potentially by direct DNA binding or by binding to other transcription factors; LIM domain containing
142	LPAR2	9606.ENSP00000443256	LPAR2	Lysophosphatidic acid receptor 2; Receptor for lysophosphatidic acid (LPA), a mediator of diverse cellular activities. Seems to be coupled to the G(i)/G(o), G(12)/G(13), and G(q) families of heteromeric G proteins. Plays a key role in phospholipase C-beta (PLC-beta) signaling pathway. Stimulates phospholipase C (PLC) activity in a manner that is independent of RALA activation
143	LPCAT4	9606.ENSP00000317300	LPCAT4	Lysophospholipid acyltransferase LPCAT4; Displays acyl-CoA-dependent lysophospholipid acyltransferase activity with a subset of lysophospholipids as substrates; converts lysophosphatidylethanolamine to phosphatidylethanolamine, lysophosphatidylcholine to phosphatidycholine, 1-alkenyl-lysophatidylethanolamine to 1- alkenyl-phosphatidylethanolamine, lysophosphatidylglycerol and alkyl-lysophosphatidylcholine to phosphatidylglycerol and alkyl- phosphatidylcholine, respectively. In contrast, has no lysophosphatidylinositol, glycerol-3-phosphate, diacylglycerol or lysophosphatidic acid acylt [...]
144	LRFN3	9606.ENSP00000466989	LRFN3	Leucine-rich repeat and fibronectin type-III domain-containing protein 3; Cell adhesion molecule that mediates homophilic cell- cell adhesion in a Ca(2+)-independent manner. Promotes neurite outgrowth in hippocampal neurons (By similarity); Fibronectin type III domain containing
145	LY6H	9606.ENSP00000399485	LY6H	Lymphocyte antigen 6H; Believed to act as a modulator of nicotinic acetylcholine receptors (nAChRs) activity. In vitro inhibits alpha-3:beta-4-containing nAChRs maximum response. May play a role in the intracellular trafficking of alpha-7-containing nAChRs and may inhibit their expression at the cell surface. Seems to inhibit alpha-7/CHRNA7 signaling in hippocampal neurons; LY6/PLAUR domain containing
146	LY6L	9606.ENSP00000455811	LY6L	Lymphocyte antigen 6L; Protein LOC101928108
148	MACROH2A1	9606.ENSP00000423563	H2AFY	Core histone macro-H2A.1; Variant histone H2A which replaces conventional H2A in a subset of nucleosomes where it represses transcription. Nucleosomes wrap and compact DNA into chromatin, limiting DNA accessibility to the cellular machineries which require DNA as a template. Histones thereby play a central role in transcription regulation, DNA repair, DNA replication and chromosomal stability. DNA accessibility is regulated via a complex set of post-translational modifications of histones, also called histone code, and nucleosome remodeling. Involved in stable X chromosome inactivation [...]
149	MAFK	9606.ENSP00000344903	MAFK	Transcription factor MafK; Since they lack a putative transactivation domain, the small Mafs behave as transcriptional repressors when they dimerize among themselves. However, they seem to serve as transcriptional activators by dimerizing with other (usually larger) basic-zipper proteins and recruiting them to specific DNA-binding sites. Small Maf proteins heterodimerize with Fos and may act as competitive repressors of the NF-E2 transcription factor; Belongs to the bZIP family. Maf subfamily
150	MAGEA8	9606.ENSP00000438293	MAGEA8	Melanoma-associated antigen 8; Not known, though may play a role in embryonal development and tumor transformation or aspects of tumor progression; MAGE family
151	MAML2	9606.ENSP00000434552	MAML2	Mastermind-like protein 2; Acts as a transcriptional coactivator for NOTCH proteins. Has been shown to amplify NOTCH-induced transcription of HES1. Potentiates activation by NOTCH3 and NOTCH4 more efficiently than MAML1 or MAML3; Belongs to the mastermind family
152	MAP3K11	9606.ENSP00000309597	MAP3K11	Mitogen-activated protein kinase kinase kinase 11; Activates the JUN N-terminal pathway. Required for serum-stimulated cell proliferation and for mitogen and cytokine activation of MAPK14 (p38), MAPK3 (ERK) and MAPK8 (JNK1) through phosphorylation and activation of MAP2K4/MKK4 and MAP2K7/MKK7. Plays a role in mitogen-stimulated phosphorylation and activation of BRAF, but does not phosphorylate BRAF directly. Influences microtubule organization during the cell cycle; Belongs to the protein kinase superfamily. STE Ser/Thr protein kinase family. MAP kinase kinase kinase subfamily
153	MAP3K7CL	9606.ENSP00000382828	MAP3K7CL	MAP3K7 C-terminal-like protein; MAP3K7 C-terminal like
154	MAP4K1	9606.ENSP00000465039	MAP4K1	Mitogen-activated protein kinase kinase kinase kinase 1; Serine/threonine-protein kinase, which may play a role in the response to environmental stress. Appears to act upstream of the JUN N-terminal pathway. May play a role in hematopoietic lineage decisions and growth regulation. Able to autophosphorylate
156	MARCKS	9606.ENSP00000478061	MARCKS	Myristoylated alanine-rich C-kinase substrate; MARCKS is the most prominent cellular substrate for protein kinase C. This protein binds calmodulin, actin, and synapsin. MARCKS is a filamentous (F) actin cross-linking protein
157	ME3	9606.ENSP00000440246	ME3	NADP-dependent malic enzyme, mitochondrial; Malic enzyme 3; Belongs to the malic enzymes family
158	MEDAG	9606.ENSP00000369849	MEDAG	Mesenteric estrogen-dependent adipogenesis protein; Involved in processes that promote adipocyte differentiation, lipid accumulation, and glucose uptake in mature adipocytes
159	MEF2C	9606.ENSP00000340874	MEF2C	Myocyte-specific enhancer factor 2C; Transcription activator which binds specifically to the MEF2 element present in the regulatory regions of many muscle- specific genes. Controls cardiac morphogenesis and myogenesis, and is also involved in vascular development. Plays an essential role in hippocampal-dependent learning and memory by suppressing the number of excitatory synapses and thus regulating basal and evoked synaptic transmission. Crucial for normal neuronal development, distribution, and electrical activity in the neocortex. Necessary for proper development of megakaryocytes a [...]
160	MEOX1	9606.ENSP00000321684	MEOX1	Homeobox protein MOX-1; Mesodermal transcription factor that plays a key role in somitogenesis and is specifically required for sclerotome development. Required for maintenance of the sclerotome polarity and formation of the cranio-cervical joints. Binds specifically to the promoter of target genes and regulates their expression. Activates expression of NKX3-2 in the sclerotome. Activates expression of CDKN1A and CDKN2A in endothelial cells, acting as a regulator of vascular cell proliferation. While it activates CDKN1A in a DNA-dependent manner, it activates CDKN2A in a DNA-independen [...]
161	MICALL2	9606.ENSP00000297508	MICALL2	MICAL-like protein 2; Effector of small Rab GTPases which is involved in junctional complexes assembly through the regulation of cell adhesion molecules transport to the plasma membrane and actin cytoskeleton reorganization. Regulates the endocytic recycling of occludins, claudins and E-cadherin to the plasma membrane and may thereby regulate the establishment of tight junctions and adherens junctions. In parallel, may regulate actin cytoskeleton reorganization directly through interaction with F-actin or indirectly through actinins and filamins. Most probably involved in the processes [...]
162	MID1	9606.ENSP00000312678	MID1	E3 ubiquitin-protein ligase Midline-1; Has E3 ubiquitin ligase activity towards IGBP1, promoting its monoubiquitination, which results in deprotection of the catalytic subunit of protein phosphatase PP2A, and its subsequent degradation by polyubiquitination; Belongs to the TRIM/RBCC family
163	MIDEAS	9606.ENSP00000286523	ELMSAN1	ELM2 and Myb/SANT domain containing 1
166	MTA1	9606.ENSP00000333633	MTA1	Metastasis-associated protein MTA1; Transcriptional coregulator which can act as both a transcriptional corepressor and coactivator. As a part of the histone-deacetylase multiprotein complex (NuRD), regulates transcription of its targets by modifying the acetylation status of the target chromatin and cofactor accessibility to the target DNA. In conjunction with

Current Violent Behaviour (CVB)

#	queryItem	stringId	preferredName	annotation
				other components of NuRD, acts as a transcriptional corepressor of BRCA1, ESR1, TFF1 and CDKN1A. Acts as a transcriptional coactivator of BCAS3, PAX5 and SUMO2, independent of the NuRD complex. Stimulates the expression of WNT1 [...]
167	MTMR6	9606.ENSP00000371221	MTMR6	Myotubularin-related protein 6; Phosphatase that acts on lipids with a phosphoinositol headgroup. Acts as a negative regulator of KCNN4/KCa3.1 channel activity in CD4+ T-cells possibly by decreasing intracellular levels of phosphatidylinositol 3 phosphatase. Negatively regulates proliferation of reactivated CD4+ T-cells; Belongs to the protein-tyrosine phosphatase family. Non-receptor class myotubularin subfamily
168	MTSS1	9606.ENSP00000322804	MTSS1	Metastasis suppressor protein 1; May be related to cancer progression or tumor metastasis in a variety of organ sites, most likely through an interaction with the actin cytoskeleton; I-BAR domain containing
169	MTUS2	9606.ENSP00000483729	MTUS2	Microtubule-associated tumor suppressor candidate 2; Binds microtubules. Together with MAPRE1 may target the microtubule depolymerase KIF2C to the plus-end of microtubules. May regulate the dynamics of microtubules at their growing distal tip; In the C-terminal section; belongs to the MTUS1 family
170	MTX2	9606.ENSP00000249442	MTX2	Metaxin-2; Involved in transport of proteins into the mitochondrion
171	MYBPC3	9606.ENSP00000442795	MYBPC3	Myosin-binding protein C, cardiac-type; Thick filament-associated protein located in the crossbridge region of vertebrate striated muscle a bands. In vitro it binds MHC, F-actin and native thin filaments, and modifies the activity of actin-activated myosin ATPase. It may modulate muscle contraction or may play a more structural role; Fibronectin type III domain containing
172	MYC	9606.ENSP00000479618	MYC	Myc proto-oncogene protein; Transcription factor that binds DNA in a non-specific manner, yet also specifically recognizes the core sequence 5'- CAC[GA]TG-3'. Activates the transcription of growth-related genes. Binds to the VEGFA promoter, promoting VEGFA production and subsequent sprouting angiogenesis; Basic helix-loop-helix proteins
173	MYL3	9606.ENSP00000379210	MYL3	Myosin light chain 3; Regulatory light chain of myosin. Does not bind calcium; EF-hand domain containing
174	MYO1H	9606.ENSP00000439182	MYO1H	Unconventional myosin-Ih; Myosins are actin-based motor molecules with ATPase activity. Unconventional myosins serve in intracellular movements. Their highly divergent tails are presumed to bind to membranous compartments, which would be moved relative to actin filaments (By similarity); Myosins, class I
175	MYO3B	9606.ENSP00000386213	MYO3B	Myosin-IIIb; Probable actin-based motor with a protein kinase activity. Required for normal cochlear hair bundle development and hearing. Plays an important role in the early steps of cochlear hair bundle morphogenesis. Influences the number and lengths of stereocilia to be produced and limits the growth of microvilli within the forming auditory hair bundles thereby contributing to the architecture of the hair bundle, including its staircase pattern. Involved in the elongation of actin in stereocilia tips by transporting the actin regulatory factor ESPN to the plus ends of actin filame [...]
176	NDRG2	9606.ENSP00000451712	NDRG2	Protein NDRG2; Contributes to the regulation of the Wnt signaling pathway. Down-regulates CTNNB1-mediated transcriptional activation of target genes, such as CCND1, and may thereby act as tumor suppressor. May be involved in dendritic cell and neuron differentiation
177	NKAIN3	9606.ENSP00000429073	NKAIN3	Sodium/potassium-transporting ATPase subunit beta-1-interacting protein 3; Sodium/potassium transporting ATPase interacting 3; Belongs to the NKAIN family
178	NPHP3	9606.ENSP00000338766	NPHP3	Nephrocystin-3; Required for normal ciliary development and function. Inhibits disheveled-1-induced canonical Wnt-signaling activity and may also play a role in the control of non-canonical Wnt signaling which regulates planar cell polarity. Probably acts as a molecular switch between different Wnt signaling pathways. Required for proper convergent extension cell movements
179	NR2F2	9606.ENSP00000377721	NR2F2	COUP transcription factor 2; Ligand-activated transcription factor. Activated by high concentrations of 9-cis-retinoic acid and all-trans-retinoic acid, but not by dexamethasone, cortisol or progesterone (in vitro). Regulation of the apolipoprotein A-I gene transcription. Binds to DNA site A; Nuclear hormone receptors
180	NRDE2	9606.ENSP00000346335	NRDE2	Protein NRDE2 homolog; NRDE-2, necessary for RNA interference, domain containing; Belongs to the NRDE2 family
181	NSUN7	9606.ENSP00000371201	NSUN7	Putative methyltransferase NSUN7; May have S-adenosyl-L-methionine-dependent methyl- transferase activity; NOP2/Sun RNA methyltransferase family
182	NT5DC2	9606.ENSP00000406933	NT5DC2	5'-nucleotidase domain containing 2
183	NWD1	9606.ENSP00000428579	NWD1	NACHT domain- and WD repeat-containing protein 1; May play a role in the control of androgen receptor (AR) protein steady-state levels; WD repeat domain containing
184	OTOG	9606.ENSP00000382323	OTOG	Otogelin; Glycoprotein specific to acellular membranes of the inner ear. May be required for the anchoring of the otoconial membranes and cupulae to the underlying neuroepithelia in the vestibule. May be involved in the organization and/or stabilization of the fibrillar network that compose the tectorial membrane in the cochlea. May play a role in mechanotransduction processes (By similarity)
185	OTUB1	9606.ENSP00000444357	OTUB1	Ubiquitin thioesterase OTUB1; Hydrolase that can specifically remove 'Lys-48'-linked conjugated ubiquitin from proteins and plays an important regulatory role at the level of protein turnover by preventing degradation. Regulator of T-cell anergy, a phenomenon that occurs when T-cells are rendered unresponsive to antigen rechallenge and no longer respond to their cognate antigen. Acts via its interaction with RNF128/GRAIL, a crucial inductor of CD4 T-cell anergy. Isoform 1 destabilizes RNF128, leading to prevent anergy. In contrast, isoform 2 stabilizes RNF128 and promotes anergy. Surpr [...]
186	P2RY2	9606.ENSP00000310305	P2RY2	P2Y purinoceptor 2; Receptor for ATP and UTP coupled to G-proteins that activate a phosphatidylinositol-calcium second messenger system. The affinity range is UTP = ATP > ATP-gamma-S >> 2-methylthio-ATP = ADP; P2Y receptors
187	PAPPA2	9606.ENSP00000356634	PAPPA2	Pappalysin-2; Metalloproteinase which specifically cleaves insulin- like growth factor binding protein (IGFBP)-5 at the '163-Ser-|- Lys-164' bond. Shows limited proteolysis toward IGFBP-3; Belongs to the peptidase M43B family
188	PDE4DIP	9606.ENSP00000358363	PDE4DIP	Myomegalin; Functions as an anchor sequestering components of the cAMP-dependent pathway to Golgi and/or centrosomes (By similarity). Forms a complex with AKAP9: the complex recruits CAMSAP2 to the Golgi apparatus, leading to tether non-centrosomal minus-end microtubules to the Golgi, an important step for polarized cell movement
189	PGAP1	9606.ENSP00000346809	PGAP1	GPI inositol-deacylase; Involved in inositol deacylation of GPI-anchored proteins. GPI inositol deacylation may important for efficient transport of GPI-anchored proteins from the endoplasmic reticulum to the Golgi (By similarity)
190	PGPEP1L	9606.ENSP00000368199	PGPEP1L	Pyroglutamyl-peptidase 1-like protein; Pyroglutamyl-peptidase I like
191	PHGR1	9606.ENSP00000410024	PHGR1	Proline, histidine and glycine rich 1
192	PITHD1	9606.ENSP00000246151	PITHD1	PITH domain containing 1
193	PIWIL2	9606.ENSP00000349208	PIWIL2	Piwi-like protein 2; Endoribonuclease that plays a central role during spermatogenesis by repressing transposable elements and preventing their mobilization, which is essential for the germline integrity (By similarity). Plays an essential role in meiotic differentiation of spermatocytes, germ cell differentiation and in self-renewal of spermatogonial stem cells (By similarity). Acts via the piRNA metabolic process, which mediates the repression of transposable elements during meiosis by forming complexes composed of piRNAs and Piwi proteins and govern the methylation and subsequent re [...]
194	PKD1	9606.ENSP00000262304	PKD1	Polycystin-1; Involved in renal tubulogenesis. Involved in fluid-flow mechanosensation by the primary cilium in renal epithelium (By similarity). Acts as a regulator of cilium length, together with PKD2 (By similarity). The dynamic control of cilium length is essential in the regulation of mechanotransductive signaling (By similarity). The cilium length response creates a negative feedback loop whereby fluid shear- mediated deflection of the primary cilium, which decreases intracellular cAMP, leads to cilium shortening and thus decreases flow-induced signaling (By similarity). May be a [...]
195	PLAC1	9606.ENSP00000352173	PLAC1	Placenta-specific protein 1; May play a role in placental development; Belongs to the PLAC1 family
196	PLCH2	9606.ENSP00000367747	PLCH2	1-phosphatidylinositol 4,5-bisphosphate phosphodiesterase eta-2; The production of the second messenger molecules diacylglycerol (DAG) and inositol 1,4,5-trisphosphate (IP3) is mediated by activated phosphatidylinositol-specific phospholipase C enzymes. This phospholipase activity is very sensitive to calcium. May be important for formation and maintenance of the neuronal network in the postnatal brain (By similarity); EF-hand domain containing
197	PLEC	9606.ENSP00000323856	PLEC	Plectin; Interlinks intermediate filaments with microtubules and microfilaments and anchors intermediate filaments to desmosomes or hemidesmosomes. Could also bind muscle proteins such as actin to membrane complexes in muscle. May be involved not only in the filaments network, but also in the regulation of their dynamics. Structural component of muscle. Isoform 9 plays a major role in the maintenance of myofiber integrity; Plakins
198	PLPPR2	9606.ENSP00000466898	LPPR2	Lipid phosphate phosphatase-related protein type 2
199	PNKP	9606.ENSP00000323511	PNKP	Bifunctional polynucleotide phosphatase/kinase; Plays a key role in the repair of DNA damage, functioning as part of both the non-homologous end-joining (NHEJ) and base excision repair (BER) pathways. Through its two catalytic activities, PNK ensures that DNA termini are compatible with extension and ligation by either removing 3'-phosphates from, or by phosphorylating 5'-hydroxyl groups on, the ribose sugar of the DNA backbone; HAD Asp-based non-protein phosphatases
200	PNPLA7	9606.ENSP00000384610	PNPLA7	Patatin-like phospholipase domain-containing protein 7; Serine hydrolase, whose specific chemical modification by certain organophosphorus (OP) compounds leads to distal axonopathy; Patatin like phospholipase domain containing
201	POLA1	9606.ENSP00000368349	POLA1	DNA polymerase alpha catalytic subunit; Plays an essential role in the initiation of DNA replication. During the S phase of the cell cycle, the DNA polymerase alpha complex (composed of a catalytic subunit POLA1/p180, a regulatory subunit POLA2/p70 and two primase subunits PRIM1/p49 and PRIM2/p58) is recruited to DNA at the replicative forks via direct interactions with MCM10 and WDHD1. The primase subunit of the polymerase alpha complex initiates DNA synthesis by oligomerising short RNA primers on both leading and lagging strands. These primers are initially extended by the polymerase [...]
202	PPL	9606.ENSP00000340510	PPL	Periplakin; Component of the cornified envelope of keratinocytes. May link the cornified envelope to desmosomes and intermediate filaments. May act as a localization signal in PKB/AKT-mediated signaling; Belongs to the plakin or cytolinker family
203	PRDM16	9606.ENSP00000270722	PRDM16	PR domain zinc finger protein 16; Binds DNA and functions as a transcriptional regulator. Functions in the differentiation of brown adipose tissue (BAT) which is specialized in dissipating chemical energy in the form of heat in response to cold or excess feeding while white adipose tissue (WAT) is specialized in the storage of excess energy and the control of systemic metabolism. Together with CEBPB, regulates the differentiation of myoblastic precursors into brown adipose cells. Functions also as a repressor of TGF-beta signaling. Isoform 4 may regulate granulocytes differentiation; L [...]
204	PRDM2	9606.ENSP00000235372	PRDM2	PR domain zinc finger protein 2; S-adenosyl-L-methionine-dependent histone methyltransferase that specifically methylates 'Lys-9' of histone H3. May function as a DNA-binding transcription factor. Binds to the macrophage-specific TPA-responsive element (MTE) of the HMOX1 (heme oxygenase 1) gene and may act as a transcriptional activator of this gene; Belongs to the class V-like SAM-binding methyltransferase superfamily
205	PRKAG2	9606.ENSP00000287878	PRKAG2	5'-AMP-activated protein kinase subunit gamma-2; AMP/ATP-binding subunit of AMP-activated protein kinase (AMPK), an energy sensor protein kinase that plays a key role in regulating cellular energy metabolism. In response to reduction of intracellular ATP levels, AMPK activates energy-producing pathways and inhibits energy-consuming processes: inhibits protein, carbohydrate and lipid biosynthesis, as well as cell growth and proliferation. AMPK acts via direct phosphorylation of metabolic enzymes, and by longer-term effects via phosphorylation of transcription regulators. Also acts as a [...]
206	PRKCB	9606.ENSP00000305355	PRKCB	Protein kinase C beta type; Calcium-activated, phospholipid- and diacylglycerol (DAG)-dependent serine/threonine-protein kinase involved in various cellular processes such as regulation of the B-cell receptor (BCR) signalosome, oxidative stress-induced apoptosis, androgen receptor-dependent transcription regulation, insulin signaling and endothelial cells proliferation. Plays a key role in B-cell activation by regulating BCR-induced NF-kappa-B activation. Mediates the activation of the canonical NF-kappa-B pathway (NFKB1) by direct phosphorylation of CARD11/CARMA1 at 'Ser-559', 'Ser-64 [...]
207	PRKCZ	9606.ENSP00000367830	PRKCZ	Protein kinase C zeta type; Calcium- and diacylglycerol-independent serine/threonine-protein kinase that functions in phosphatidylinositol 3-kinase (PI3K) pathway and mitogen-activated protein (MAP) kinase cascade, and is involved in NF-kappa-B activation, mitogenic signaling, cell proliferation, cell polarity, inflammatory response and maintenance of long-term potentiation (LTP). Upon lipopolysaccharide (LPS) treatment in macrophages, or following mitogenic stimuli, functions downstream of PI3K to activate MAP2K1/MEK1-MAPK1/ERK2 signaling cascade independently of RAF1 activation. Requ [...]
208	PROK2	9606.ENSP00000295619	PROK2	Prokineticin-2; May function as an output molecule from the suprachiasmatic nucleus (SCN) that transmits behavioral circadian rhythm. May also function locally within the SCN to synchronize output. Potently contracts gastrointestinal (GI) smooth muscle; Endogenous ligands
209	PRORP	9606.ENSP00000440915	KIAA0391	Mitochondrial ribonuclease P catalytic subunit; Catalytic ribonuclease component of mitochondrial ribonuclease P, a complex composed of TRMT10C/MRPP1, HSD17B10/MRPP2 and MRPP3, which cleaves tRNA molecules in their 5'-ends. The presence of TRMT10C/MRPP1, HSD17B10/MRPP2 is required to catalyze tRNA molecules in their 5'-ends
210	PSEN1	9606.ENSP00000326366	PSEN1	Presenilin-1; Catalytic subunit of the gamma-secretase complex, an endoprotease complex that catalyzes the intramembrane cleavage of integral membrane proteins such as Notch receptors and APP (amyloid-beta precursor protein). Requires the presence of the other members of the gamma-secretase complex for protease activity. Plays a role in Notch and Wnt signaling cascades and regulation of downstream processes via its role in processing key regulatory proteins, and by regulating cytosolic CTNNB1 levels. Stimulates cell-cell adhesion via its interaction with CDH1; this stabilizes the compl [...]
211	PTCD3	9606.ENSP00000254630	PTCD3	Pentatricopeptide repeat domain-containing protein 3, mitochondrial; Mitochondrial RNA-binding protein that has a role in mitochondrial translation
212	PTPRF	9606.ENSP00000353030	PTPRF	Receptor-type tyrosine-protein phosphatase F; Possible cell adhesion receptor. It possesses an intrinsic protein tyrosine phosphatase activity (PTPase) and dephosphorylates EPHA2 regulating its activity; Fibronectin type III domain containing
213	PXDC1	9606.ENSP00000369636	PXDC1	PX domain containing 1
214	PXN	9606.ENSP00000267257	PXN	Paxillin; Cytoskeletal protein involved in actin-membrane attachment at sites of cell adhesion to the extracellular matrix (focal adhesion); Belongs to the paxillin family
215	QKI	9606.ENSP00000355094	QKI	Protein quaking; RNA-binding protein that plays a central role in myelinization. Binds to the 5'-NACUAAY-N(1,20)- UAAY-3' RNA core sequence. Regulates target mRNA stability. In addition, acts by regulating pre-mRNA splicing, mRNA export and protein translation. Required to protect and promote stability of mRNAs such as MBP and CDKN1B. Regulator of oligodendrocyte differentiation and maturation in the brain that may play a role in myelin and oligodendrocyte dysfunction in schizophrenia. Participates in mRNA transport by regulating the nuclear export of MBP mRNA. Also involved in regulat [...]
216	RAB3IL1	9606.ENSP00000378313	RAB3IL1	Guanine nucleotide exchange factor for Rab-3A; Guanine nucleotide exchange factor (GEF) which may activate RAB3A, a GTPase that regulates synaptic vesicle exocytosis. Promotes the exchange of GDP to GTP, converting inactive GDP-bound Rab proteins into their active GTP-bound form. May also activate RAB8A and RAB8B; Belongs to the SEC2 family
217	RAPGEF1	9606.ENSP00000361263	RAPGEF1	Rap guanine nucleotide exchange factor 1; Guanine nucleotide-releasing protein that binds to SH3 domain of CRK and GRB2/ASH. Transduces signals from CRK to activate RAS. Plays a role in the establishment of basal endothelial barrier function. Plays a role in nerve growth factor (NGF)-induced sustained activation of Rap1 and neurite outgrowth
218	RAPGEFL1	9606.ENSP00000264644	RAPGEFL1	Rap guanine nucleotide exchange factor-like 1; Probable guanine nucleotide exchange factor (GEF)
219	RBFOX3	9606.ENSP00000463653	RBFOX3	RNA binding protein fox-1 homolog 3; Pre-mRNA alternative splicing regulator. Regulates alternative splicing of RBFOX2 to enhance the production of mRNA species that are targeted for nonsense-mediated decay (NMD); RNA binding motif containing
220	REEP2	9606.ENSP00000367590	REEP2	Receptor expression-enhancing protein 2; Required for endoplasmic reticulum (ER) network formation, shaping and remodeling. May enhance the cell surface expression of odorant receptors (By similarity); Belongs to the DP1 family

Current Violent Behaviour (CVB)

#	queryItem	stringId	preferredName	annotation
221	RHCG	9606.ENSP00000268122	RHCG	Ammonium transporter Rh type C; Functions as an electroneutral and bidirectional ammonium transporter. May regulate transepithelial ammonia secretion; Solute carriers
222	RHEB	9606.ENSP00000262187	RHEB	GTP-binding protein Rheb; Activates the protein kinase activity of mTORC1, and thereby plays a role in the regulation of apoptosis. Stimulates the phosphorylation of S6K1 and EIF4EBP1 through activation of mTORC1 signaling. Has low intrinsic GTPase activity; RAS type GTPase family
223	RPN1	9606.ENSP00000296255	RPN1	Dolichyl-diphosphooligosaccharide--protein glycosyltransferase subunit 1; Essential subunit of the N-oligosaccharyl transferase (OST) complex which catalyzes the transfer of a high mannose oligosaccharide from a lipid-linked oligosaccharide donor to an asparagine residue within an Asn-X-Ser/Thr consensus motif in nascent polypeptide chains; Belongs to the OST1 family
224	S1PR2	9606.ENSP00000466933	S1PR2	Sphingosine 1-phosphate receptor 2; Receptor for the lysosphingolipid sphingosine 1- phosphate (S1P). S1P is a bioactive lysophospholipid that elicits diverse physiological effect on most types of cells and tissues. When expressed in rat HTC4 hepatoma cells, is capable of mediating S1P-induced cell proliferation and suppression of apoptosis
225	SCRT1	9606.ENSP00000455711	SCRT1	Transcriptional repressor scratch 1; Transcriptional repressor that binds E-box motif CAGGTG. Can modulate the action of basic helix-loop-helix (bHLH) transcription factors, critical for neuronal differentiation; SNAG transcriptional repressors
226	SEC22C	9606.ENSP00000264454	SEC22C	Vesicle-trafficking protein SEC22c; May be involved in vesicle transport between the ER and the Golgi complex; SNAREs
227	SEMA4C	9606.ENSP00000306844	SEMA4C	Semaphorin-4C; Cell surface receptor for PLXNB2 that plays an important role in cell-cell signaling. PLXNB2 binding promotes downstream activation of RHOA and phosphorylation of ERBB2 at 'Tyr-1248'. Required for normal brain development, axon guidance and cell migration (By similarity). Probable signaling receptor which may play a role in myogenic differentiation through activation of the stress-activated MAPK cascade; Belongs to the semaphorin family
228	SEMA6A	9606.ENSP00000257414	SEMA6A	Semaphorin-6A; Cell surface receptor for PLXNA2 that plays an important role in cell-cell signaling. Required for normal granule cell migration in the developing cerebellum. Promotes reorganization of the actin cytoskeleton and plays an important role in axon guidance in the developing central nervous system. Can act as repulsive axon guidance cue. Has repulsive action towards migrating granular neurons. May play a role in channeling sympathetic axons into the sympathetic chains and controlling the temporal sequence of sympathetic target innervation (By similarity); Belongs to the sema [...]
229	SEMA6D	9606.ENSP00000324857	SEMA6D	Semaphorin-6D; Shows growth cone collapsing activity on dorsal root ganglion (DRG) neurons in vitro. May be a stop signal for the DRG neurons in their target areas, and possibly also for other neurons. May also be involved in the maintenance and remodeling of neuronal connections; Belongs to the semaphorin family
231	SETD1B	9606.ENSP00000442924	SETD1B	Histone-lysine N-methyltransferase SETD1B; Histone methyltransferase that specifically methylates 'Lys-4' of histone H3, when part of the SET1 histone methyltransferase (HMT) complex, but not if the neighboring 'Lys- 9' residue is already methylated. H3 'Lys-4' methylation represents a specific tag for epigenetic transcriptional activation. The non-overlapping localization with SETD1A suggests that SETD1A and SETD1B make non-redundant contributions to the epigenetic control of chromatin structure and gene expression. Specifically tri-methylates 'Lys-4' of histone H3 in vitro; Belongs t [...]
232	SH3BP1	9606.ENSP00000350018	SH3BP1	SH3 domain-binding protein 1; GTPase activating protein (GAP) which specifically converts GTP-bound Rho-type GTPases including RAC1 and CDC42 in their inactive GDP-bound form. By specifically inactivating RAC1 at the leading edge of migrating cells, it regulates the spatiotemporal organization of cell protrusions which is important for proper cell migration. Also negatively regulates CDC42 in the process of actin remodeling and the formation of epithelial cell junctions. Through its GAP activity toward RAC1 and/or CDC42 plays a specific role in phagocytosis of large particles. Specific [...]
233	SH3KBP1	9606.ENSP00000380921	SH3KBP1	SH3 domain-containing kinase-binding protein 1; Adapter protein involved in regulating diverse signal transduction pathways. Involved in the regulation of endocytosis and lysosomal degradation of ligand-induced receptor tyrosine kinases, including EGFR and MET/hepatocyte growth factor receptor, through a association with CBL and endophilins. The association with CBL, and thus the receptor internalization, may inhibited by an interaction with PDCD6IP and/or SPRY2. Involved in regulation of ligand-dependent endocytosis of the IgE receptor. Attenuates phosphatidylinositol 3-kinase activit [...]
234	SH3PXD2A	9606.ENSP00000348215	SH3PXD2A	SH3 and PX domain-containing protein 2A; Adapter protein involved in invadopodia and podosome formation, extracellular matrix degradation and invasiveness of some cancer cells. Binds matrix metalloproteinases (ADAMs), NADPH oxidases (NOXs) and phosphoinositides. Acts as an organizer protein that allows NOX1- or NOX3-dependent reactive oxygen species (ROS) generation and ROS localization. In association with ADAM12, mediates the neurotoxic effect of amyloid-beta peptide
235	SHISA9	9606.ENSP00000454014	SHISA9	Protein shisa-9; Regulator of short-term neuronal synaptic plasticity in the dentate gyrus. Associates with AMPA receptors (ionotropic glutamate receptors) in synaptic spines and promotes AMPA receptor desensitization at excitatory synapses (By similarity); Belongs to the shisa family. SHISA9 subfamily
236	SKIDA1	9606.ENSP00000410041	SKIDA1	SKI/DACH domain containing 1
237	SLC16A2	9606.ENSP00000465734	SLC16A2	Monocarboxylate transporter 8; Very active and specific thyroid hormone transporter. Stimulates cellular uptake of thyroxine (T4), triiodothyronine (T3), reverse triiodothyronine (rT3) and diiodothyronine. Does not transport Leu, Phe, Trp or Tyr; Belongs to the major facilitator superfamily. Monocarboxylate porter (TC 2.A.1.13) family
238	SLC25A26	9606.ENSP00000346955	SLC25A26	S-adenosylmethionine mitochondrial carrier protein; Mitochondrial solute carriers shuttle metabolites, nucleotides, and cofactors through the mitochondrial inner membrane. Specifically mediates the transport of S- adenosylmethionine (SAM) into the mitochondria
239	SLC2A4RG	9606.ENSP00000266077	SLC2A4RG	SLC2A4 regulator; Transcription factor involved in SLC2A4 and HD gene transactivation. Binds to the consensus sequence 5'-GCCGGCG-3'
240	SLC35F3	9606.ENSP00000355577	SLC35F3	Putative thiamine transporter SLC35F3; May be a thiamine transporter; Belongs to the SLC35F solute transporter family
241	SLC38A5	9606.ENSP00000471683	SLC38A5	Sodium-coupled neutral amino acid transporter 5; Functions as a sodium-dependent amino acid transporter which countertransport protons. Mediates the saturable, pH- sensitive, and electrogenic cotransport of several neutral amino acids including glycine, asparagine, alanine, serine, glutamine and histidine with sodium
242	SLC41A3	9606.ENSP00000326070	SLC41A3	Solute carrier family 41 member 3
243	SLCO4A1	9606.ENSP00000217159	SLCO4A1	Solute carrier organic anion transporter family member 4A1; Mediates the Na(+)-independent transport of organic anions such as the thyroid hormones T3 (triiodo-L-thyronine), T4 (thyroxine) and rT3, and of estrone-3-sulfate and taurocholate; Belongs to the organo anion transporter (TC 2.A.60) family
244	SMYD3	9606.ENSP00000419184	SMYD3	Histone-lysine N-methyltransferase SMYD3; Histone methyltransferase. Specifically methylates 'Lys- 4' of histone H3, inducing di- and tri-methylation, but not monomethylation. Also methylates 'Lys-5' of histone H4. Plays an important role in transcriptional activation as a member of an RNA polymerase complex. Binds DNA containing 5'- CCCTCC-3' or 5'-GAGGGG-3' sequences; Belongs to the class V-like SAM-binding methyltransferase superfamily. Histone-lysine methyltransferase family
245	SNTG1	9606.ENSP00000429842	SNTG1	Gamma-1-syntrophin; Adapter protein that binds to and probably organizes the subcellular localization of a variety of proteins. May link various receptors to the actin cytoskeleton and the dystrophin glycoprotein complex (By similarity). May participate in regulating the subcellular location of diacylglycerol kinase-zeta to ensure that diacylglycerol is rapidly inactivated following receptor activation; Belongs to the syntrophin family
246	SOBP	9606.ENSP00000318900	SOBP	Sine oculis-binding protein homolog; Implicated in development of the cochlea
247	SPACA7	9606.ENSP00000283550	SPACA7	Sperm acrosome-associated protein 7; Involved in fertilization. Seems not to play a direct role in sperm-egg binding or gamete fusion
248	SPEM2	9606.ENSP00000328061	C17orf74	Uncharacterized protein SPEM2; Chromosome 17 open reading frame 74
249	SPIDR	9606.ENSP00000297423	SPIDR	DNA repair-scaffolding protein; Plays a role in DNA double-strand break (DBS) repair via homologous recombination (HR). Serves as a scaffolding protein that helps to promote the recruitment of DNA-processing enzymes like the helicase BLM and recombinase RAD51 to site of DNA damage, and hence contributes to maintain genomic integrity
250	SPRED2	9606.ENSP00000348753	SPRED2	Sprouty-related, EVH1 domain-containing protein 2; Negatively regulates Ras signaling pathways and downstream activation of MAP kinases
252	ST3GAL2	9606.ENSP00000377257	ST3GAL2	CMP-N-acetylneuraminate-beta-galactosamide-alpha-2,3-sialyltransferase 2; Responsible for the synthesis of the sequence NeuAc- alpha-2,3-Gal-beta-1,3-GalNAc- found in terminal carbohydrate groups of certain glycoproteins, oligosaccharides and glycolipids. SIAT4A and SIAT4B sialylate the same acceptor substrates but exhibit different Km values; Belongs to the glycosyltransferase 29 family
253	STEAP1B	9606.ENSP00000384370	STEAP1B	STEAP family member 1B; Belongs to the STEAP family
254	STIM1	9606.ENSP00000478059	STIM1	Stromal interaction molecule 1; Plays a role in mediating store-operated Ca(2+) entry (SOCE), a Ca(2+) influx following depletion of intracellular Ca(2+) stores. Acts as Ca(2+) sensor in the endoplasmic reticulum via its EF-hand domain. Upon Ca(2+) depletion, translocates from the endoplasmic reticulum to the plasma membrane where it activates the Ca(2+) release-activated Ca(2+) (CRAC) channel subunit ORAI1. Involved in enamel formation. Activated following interaction with STIMATE, leading to promote STIM1 conformational switch; Sterile alpha motif domain containing
255	STK10	9606.ENSP00000176763	STK10	Serine/threonine-protein kinase 10; Serine/threonine-protein kinase involved in regulation of lymphocyte migration. Phosphorylates MSN, and possibly PLK1. Involved in regulation of lymphocyte migration by mediating phosphorylation of ERM proteins such as MSN. Acts as a negative regulator of MAP3K1/MEKK1. May also act as a cell cycle regulator by acting as a polo kinase kinase: mediates phosphorylation of PLK1 in vitro; however such data require additional evidences in vivo; Belongs to the protein kinase superfamily. STE Ser/Thr protein kinase family. STE20 subfamily
256	STX2	9606.ENSP00000376178	STX2	Syntaxin-2; Essential for epithelial morphogenesis. May mediate Ca(2+)-regulation of exocytosis acrosomal reaction in sperm; Syntaxins
257	TALDO1	9606.ENSP00000321259	TALDO1	Transaldolase; Transaldolase is important for the balance of metabolites in the pentose-phosphate pathway
258	TBC1D22A	9606.ENSP00000336724	TBC1D22A	TBC1 domain family member 22A; May act as a GTPase-activating protein for Rab family protein(s)
259	TCIRG1	9606.ENSP00000265686	TCIRG1	V-type proton ATPase 116 kDa subunit a isoform 3; Part of the proton channel of V-ATPases (By similarity). Seems to be directly involved in T-cell activation; Belongs to the V-ATPase 116 kDa subunit family
261	TK2	9606.ENSP00000299697	TK2	Thymidine kinase 2, mitochondrial; Phosphorylates thymidine, deoxycytidine, and deoxyuridine in the mitochondrial matrix. In non-replicating cells, where cytosolic dNTP synthesis is down-regulated, mtDNA synthesis depends solely on TK2 and DGUOK. Widely used as target of antiviral and chemotherapeutic agents; Belongs to the DCK/DGK family
262	TMED10	9606.ENSP00000303145	TMED10	Transmembrane emp24 domain-containing protein 10; Involved in vesicular protein trafficking. Mainly functions in the early secretory pathway. Thought to act as cargo receptor at the lumenal side for incorporation of secretory cargo molecules into transport vesicles and to be involved in vesicle coat formation at the cytoplasmic side. In COPII vesicle-mediated anterograde transport involved in the transport of GPI-anchored proteins and proposed to act together with TMED2 as their cargo receptor; the function specifically implies SEC24C and SEC24D of the COPII vesicle coat and lipid raft [...]
263	TMEM92	9606.ENSP00000300433	TMEM92	Transmembrane protein 92
264	TNFRSF6B	9606.ENSP00000359013	TNFRSF6B	Tumor necrosis factor receptor superfamily, member 6b, decoy; Decoy receptor that can neutralize the cytotoxic ligands TNFS14/LIGHT, TNFSF15 and TNFSF6/FASL. Protects against apoptosis; Tumor necrosis factor receptor superfamily
265	TNNT3	9606.ENSP00000278317	TNNT3	Troponin T, fast skeletal muscle; Troponin T is the tropomyosin-binding subunit of troponin, the thin filament regulatory complex which confers calcium-sensitivity to striated muscle actomyosin ATPase activity
266	TOMM20	9606.ENSP00000355566	TOMM20	Mitochondrial import receptor subunit TOM20 homolog; Central component of the receptor complex responsible for the recognition and translocation of cytosolically synthesized mitochondrial preproteins. Together with TOM22 functions as the transit peptide receptor at the surface of the mitochondrion outer membrane and facilitates the movement of preproteins into the TOM40 translocation pore (By similarity); Belongs to the Tom20 family
268	TREH	9606.ENSP00000264029	TREH	Trehalase; Intestinal trehalase is probably involved in the hydrolysis of ingested trehalose; Belongs to the glycosyl hydrolase 37 family
269	TRIM59	9606.ENSP00000311219	TRIM59	Tripartite motif-containing protein 59; May serve as a multifunctional regulator for innate immune signaling pathways; Ring finger proteins
270	TSNARE1	9606.ENSP00000303437	TSNARE1	t-SNARE domain containing 1
271	TTC21B	9606.ENSP00000243344	TTC21B	Tetratricopeptide repeat protein 21B; Component of the IFT complex A (IFT-A), a complex required for retrograde ciliary transport. Negatively modulates the SHH signal transduction (By similarity); Intraflagellar transport proteins
272	TTC39C	9606.ENSP00000323645	TTC39C	Tetratricopeptide repeat domain containing
273	TTLL4	9606.ENSP00000375951	TTLL4	Tubulin polyglutamylase TTLL4; Glutamylase which preferentially modifies beta-tubulin and non-tubulin proteins, such as NAP1L1, NAP1L4 and CGAS/MB21D1. Involved in the side-chain initiation step of the polyglutamylation reaction rather than in the elongation step. Involved in formation of short side-chains. Mediates initiation of polyglutamylation of nucleosome assembly proteins NAP1L1 and NAP1L4. Also acts as a monoglutamylase: generates monoglutamylation of CGAS/MB21D1, leading to impair the nucleotidyltransferase activity of CGAS/MB21D1; Tubulin tyrosine ligase family
274	TUBGCP3	9606.ENSP00000261965	TUBGCP3	Gamma-tubulin complex component 3; Gamma-tubulin complex is necessary for microtubule nucleation at the centrosome
275	TXLNA	9606.ENSP00000362711	TXLNA	Alpha-taxilin; May be involved in intracellular vesicle traffic and potentially in calcium-dependent exocytosis in neuroendocrine cells; Belongs to the taxilin family
276	UBAC2	9606.ENSP00000383911	UBAC2	Ubiquitin-associated domain-containing protein 2; Restricts trafficking of FAF2 from the endoplasmic reticulum to lipid droplets; Rhomboid family
277	UBE2G2	9606.ENSP00000338348	UBE2G2	Ubiquitin-conjugating enzyme E2 G2; Accepts ubiquitin from the E1 complex and catalyzes its covalent attachment to other proteins. In vitro catalyzes 'Lys- 48'-linked polyubiquitination. Involved in endoplasmic reticulum- associated degradation (ERAD); Ubiquitin conjugating enzymes E2
278	UBL4A	9606.ENSP00000358674	UBL4A	Ubiquitin-like protein 4A; As part of a cytosolic protein quality control complex, the BAG6/BAT3 complex, maintains misfolded and hydrophobic patches-containing proteins in a soluble state and participates to their proper delivery to the endoplasmic reticulum or alternatively can promote their sorting to the proteasome where they undergo degradation. The BAG6/BAT3 complex is involved in the post-translational delivery of tail-anchored/type II transmembrane proteins to the endoplasmic reticulum membrane. Recruited to ribosomes, it interacts with the transmembrane region of newly synthes [...]
279	UTP14A	9606.ENSP00000377944	UTP14A	U3 small nucleolar RNA-associated protein 14 homolog A; May be required for ribosome biogenesis
281	WDR45	9606.ENSP00000348848	WDR45	WD repeat domain phosphoinositide-interacting protein 4; Plays an important role in the autophagy pathway, which is the major intracellular degradation system by which cytoplasmic materials are packaged into autophagosomes and delivered to lysosomes for degradation; Belongs to the WD repeat SVP1 family
282	WDR5	9606.ENSP00000351446	WDR5	WD repeat-containing protein 5; Contributes to histone modification. May position the N- terminus of histone H3 for efficient trimethylation at 'Lys-4'. As part of the MLL1/MLL complex it is involved in methylation and dimethylation at 'Lys-4' of histone H3. H3 'Lys-4' methylation represents a specific tag for epigenetic transcriptional activation. As part of the NSL complex it may be involved in acetylation of nucleosomal histone H4 on several lysine residues. May regulate osteoblasts differentiation; Belongs to the WD repeat WDR5/wds family
283	XYLT2	9606.ENSP00000017003	XYLT2	Xylosyltransferase 2; Involved in the formation of heparan sulfate and chondroitin sulfate proteoglycans. Probably catalyzes the first step in biosynthesis of glycosaminoglycan. Transfers D-xylose from UDP-D-xylose to specific serine residues of the core protein. Initial enzyme in the biosynthesis of chondroitin sulfate and dermatan sulfate proteoglycans in fibroblasts and chondrocytes (By similarity). Its enzyme activity has not been demonstrated; Belongs to the glycosyltransferase 14 family. XylT subfamily
284	ZAP70	9606.ENSP00000264972	ZAP70	Tyrosine-protein kinase ZAP-70; Tyrosine kinase that plays an essential role in regulation of the adaptive immune response. Regulates motility, adhesion and cytokine expression of mature T-cells, as well as thymocyte development. Contributes also to the development and activation of primary B-lymphocytes. When antigen presenting cells (APC) activate T-cell receptor (TCR), a serie of phosphorylations lead to the recruitment of ZAP70 to the doubly phosphorylated TCR component CD247/CD3Z through ITAM motif at the plasma membrane. This recruitment serves to localization to the stimulated T [...]

Current Violent Behaviour (CVB)

#	queryItem	stringId	preferredName	annotation
285	ZBED9	9606.ENSP00000395259	ZBED9	SCAN domain-containing protein 3; Zinc finger BED-type containing 9; SCAN domain containing
286	ZBTB7C	9606.ENSP00000468782	ZBTB7C	Zinc finger and BTB domain-containing protein 7C; May be a tumor suppressor gene; BTB domain containing
287	ZC3H11A	9606.ENSP00000438527	ZC3H11A	Zinc finger CCCH domain-containing protein 11A; Involved in nuclear mRNA export; probably mediated by assoociation with the TREX complex; Zinc fingers CCCH-type
288	ZDBF2	9606.ENSP00000363545	ZDBF2	DBF4-type zinc finger-containing protein 2; Zinc finger DBF-type containing 2
289	ZMIZ1	9606.ENSP00000334474	ZMIZ1	Zinc finger MIZ domain-containing protein 1; Acts as transcriptional coactivator. Increases ligand- dependent transcriptional activity of AR and promotes AR sumoylation. The stimulation of AR activity is dependent upon sumoylation. Involved in transcriptional activation of a subset of NOTCH1 target genes including MYC. Involved in thymocyte and T cell development (By similarity); Zinc fingers MIZ-type
290	ZMYM3	9606.ENSP00000322845	ZMYM3	Zinc finger MYM-type protein 3; Plays a role in the regulation of cell morphology and cytoskeletal organization; Zinc fingers MYM-type
291	ZNF263	9606.ENSP00000219069	ZNF263	Zinc finger protein 263; Might play an important role in basic cellular processes as a transcriptional repressor; Belongs to the krueppel C2H2-type zinc-finger protein family
292	ZNF273	9606.ENSP00000418719	ZNF273	Zinc finger protein 273; May be involved in transcriptional regulation; Belongs to the krueppel C2H2-type zinc-finger protein family
293	ZNF335	9606.ENSP00000325326	ZNF335	Zinc finger protein 335; Component or associated component of some histone methyltransferase complexes may regulate transcription through recruitment of those complexes on gene promoters. Enhances ligand- dependent transcriptional activation by nuclear hormone receptors. Plays an important role in neural progenitor cell proliferation and self-renewal through the regulation of specific genes involved brain development, including REST. Also controls the expression of genes involved in somatic development and regulates, for instance, lymphoblast proliferation; Zinc fingers C2H2-type
294	ZNF516	9606.ENSP00000394757	ZNF516	Zinc finger protein 516; Transcriptional regulator that binds to the promoter and activates the transcription of genes promoting brown adipose tissue (BAT) differentiation. Among brown adipose tissue-specific genes, binds the proximal region of the promoter of the UCP1 gene to activate its transcription and thereby regulate thermogenesis (By similarity). May also play a role in the cellular response to replication stress; Zinc fingers C2H2-type
295	ZNF518A	9606.ENSP00000485614	ZNF518A	Zinc finger protein 518A; May be involved in transcriptional regulation; Zinc fingers C2H2-type

Current Violent Behaviour (CVB)

Association with Feelings of Guilt

Gene list

ABCC12, ACKR3, ACVR1C, ACVR2A, ACVRL1, ADCYAP1, ADGB, ADGRG7, AGBL4, AK1, AMDHD2, ANGPT4, ARHGAP10, ARHGAP5, ARHGEF3, ARID3B, ARMCX5, ARMH4, ATE1, ATF7IP, B3GAT1, BANP, BCAT1, BCL11B, BEND7, BRD8, BRINP1, BTBD3, C2orf27A, CACNA1A, CACNB2, CAMK1G, CAMTA1, CAVIN2, CBFA2T3, CBLB, CBLN4, CCDC105, CCDC149, CCDC182, CCDC185, CCKBR, CCN1, CCR7, CD53, CD99L2, CDC20B, CDH22, CDH26, CEACAM1, CENPI-DRP2, CHST11, CHST8, CLTCL1, COL12A1, CORO2A, COX6B2, CPLX2, CPNE4, CRIM1, CRYBG3, CSDC2, CSMD1, CTNNBL1, DAB2IP, DBX1, DCDC2C, DCN, DDIT4, DEFA4, DENND2B, DLX5, DMBT1, DMTN, DNAJC14, DNASE2B, DPH1, DSG3, DTX3, E4F1, EEF1E1, EFCAB11, EMILIN2, ENTPD7, EPHA2, EPHB3, ERLIN2, ERRFI1, F11R, FAAH2, FAM110B, FAM155B, FAM169A, FAM98C, FBXO34, FNDC7, FOLH1, FOSL2, FOXL1, FZD3, GADD45G, GAL3ST2, GAL3ST3, GALNT11, GATAD2A, GCNT4, GK2, GLI2, GNG7, GNPAT, GPM6A, GPR12, GRAMD2, GRIK4, GSE1, HIVEP1, HLA-DQB2, HNRNPLL, HS2ST1, HUS1, IFT22, IGFBP5, IGHD2-8, IGLL3P, IGSF1, IL5RA, ING4, INPP5B, INSIG2, INTS6L, ISM1, KCNAB2, KCNB2, KCNH7, KCNIP4, KLK7, LAPTM5, LBX1, LCE3A, LDLRAP1, LECT2, LEP, LGMN, LHX2, LMAN2, LNPEP, LPCAT1, LRRC26, LRRC74B, LUM, MACROD2, MAGEA10, MAN2B1, MAPK4, MAPRE1, MAST4, MBP, MCTP2, MED10, MEIS1, MFAP3, MICAL3, MKI67, MROH1, MSN, MTRNR2L1, MYL3, MYLK, MYT1, NAGK, NCKAP5, NEDD4L, NME5, NOL4, NPAS3, NPTX2, NR4A3, NRXN3, NSMCE1, NUDT6, NUP50, NYAP2, OGDHL, OGFRL1, OLFM3, OR1E1, OTOS, OTP, OTUD7A, P4HB, PABIR2, PABPN1L, PAQR8, PARP6, PARVA, PCDH7, PDE2A, PHACTR2, PHKA1, PI15, PIMREG, PLD5, PNOC, PNPLA7, PPARA, PPEF2, PPP2R2A, PPP4R4, PRDM1, PRDM5, PRELID3A, PRLHR, PRSS16, PTDSS1, PTGR2, RAC2, RAET1L, RALGAPA1, RANBP10, RAP2A, RBM10, RBM46, RELN, RERE, RGS9, RHCG, RIOK1, RNF144B, RPH3AL, RPS6KA2, RRAGB, RTL1, RTTN, S100A2, SEL1L2, SEPP1, SFSWAP, SGMS2, SH3BP2, SHANK1, SHISA6, SLC22A18, SLC23A2, SLC35F3, SLC46A1, SLC6A3, SLC9C1, SLCO4C1, SLIT1, SMAP2, SMARCA1, SNX16, SPIDR, SPTLC2, SRRM4, STAC, STX3, TANGO6, TBPL2, TECTB, TEX29, THADA, TLE1, TMED8, TMEM74, TNFRSF19, TNFSF13B, TNS3, TOX, TPH2, TRAPPC2L, TRBC2, TRIM31, TRPA1, TRPV1, TSPAN9, TTLL11, UCKL1, UNCX, UTP18, VAC14, VAX1, VCX3A, VKORC1L1, VPS37B, WDR13, WNK1, WWC1, YPEL4, ZFHX3, ZMYM1, ZNF704, ZNF733P, ZP3

Network

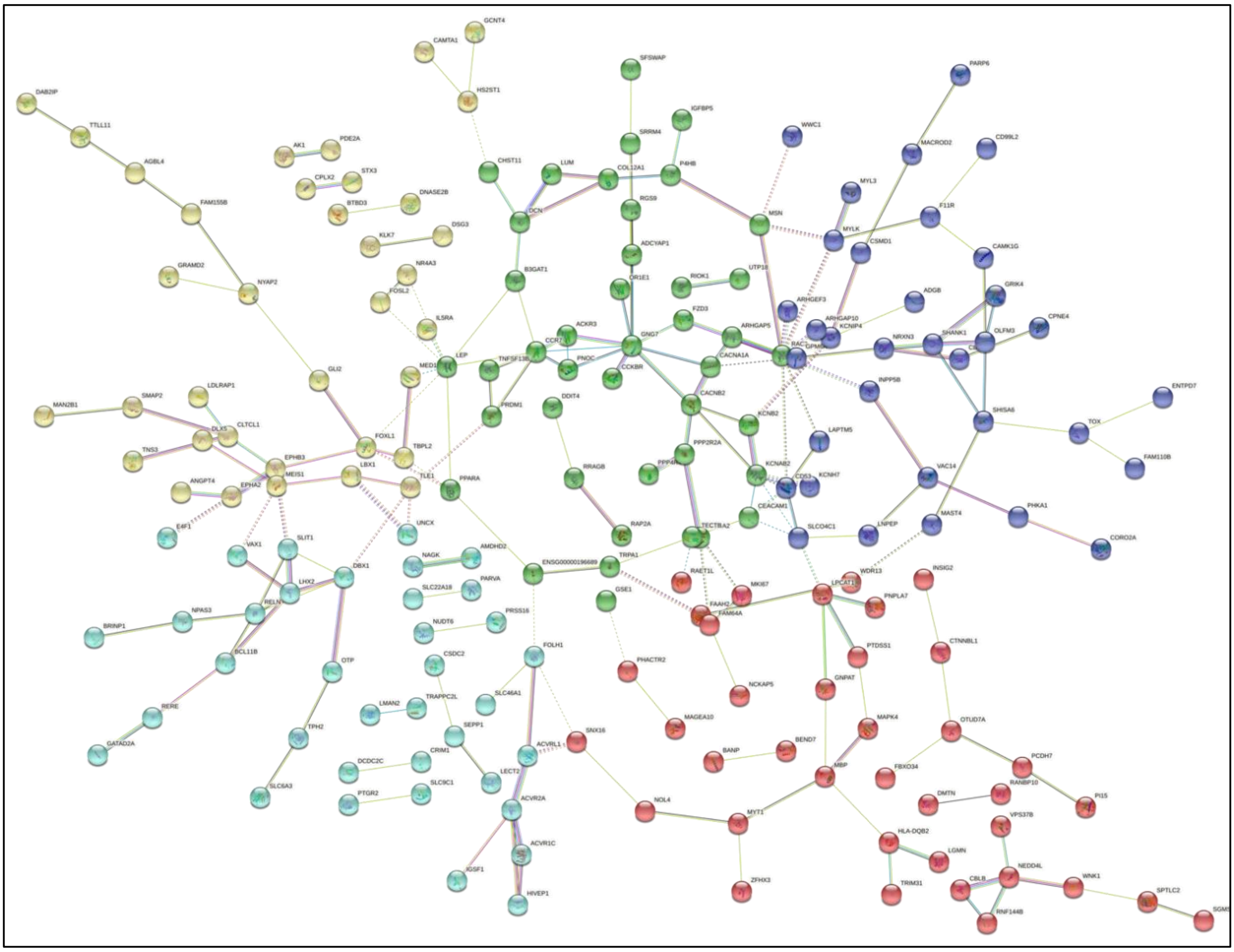

Feelings of Guilt (AAGS)

Enrichments found

Network Stats

number of nodes:	287	expected number of edges:	190
number of edges:	207	PPI enrichment p-value:	0.119
average node degree:	1.44		
avg. local clustering coefficient:	0.352		

*your network does **not** have significantly more interactions than expected (what does that mean?)*

Functional enrichments in your network

explain columns

Enrichment Table Columns

Count In Network:
The first number indicates how many proteins in your network are annotated with a particular term. The second number indicates how many proteins in total (in your network and in the background) have this term assigned.

Strength:
Log10(observed / expected). This measure describes how large the enrichment effect is. It's the ratio between i) the number of proteins in your network that are annotated with a term and ii) the number of proteins that we expect to be annotated with this term in a random network of the same size.

False Discovery Rate:
This measure describes how significant the enrichment is. Shown are p-values corrected for multiple testing within each category using the Benjamini–Hochberg procedure.

Biological Process (Gene Ontology)

GO-term	description	count in network	strength	false discovery rate
GO:0007420	brain development	25 of 650	0.42	0.0150
GO:0007417	central nervous system development	30 of 861	0.38	0.0150
GO:0040011	locomotion	34 of 1144	0.31	0.0405
GO:0048513	animal organ development	67 of 2926	0.19	0.0494
GO:0007275	multicellular organism development	105 of 4726	0.18	0.0079
GO:0048731	system development	89 of 4144	0.17	0.0339
GO:0048856	anatomical structure development	109 of 5085	0.16	0.0124
GO:0032502	developmental process	114 of 5401	0.16	0.0124
GO:0032501	multicellular organismal process	129 of 6507	0.13	0.0182

(less ...)

Feelings of Guilt (AAGS)

Mapping

#	queryItem	stringId	preferredName	annotation
1	ABCC12	9606.ENSP00000311030	ABCC12	Multidrug resistance-associated protein 9; Probable transporter; ATP binding cassette subfamily C
2	ACKR3	9606.ENSP00000272928	ACKR3	Atypical chemokine receptor 3; Atypical chemokine receptor that controls chemokine levels and localization via high-affinity chemokine binding that is uncoupled from classic ligand-driven signal transduction cascades, resulting instead in chemokine sequestration, degradation, or transcytosis. Also known as interceptor (internalizing receptor) or chemokine-scavenging receptor or chemokine decoy receptor. Acts as a receptor for chemokines CXCL11 and CXCL12/SDF1. Chemokine binding does not activate G-protein- mediated signal transduction but instead induces beta-arrestin recruitment, lead [...]
3	ACVR1C	9606.ENSP00000243349	ACVR1C	Activin receptor type-1C; Serine/threonine protein kinase which forms a receptor complex on ligand binding. The receptor complex consisting of 2 type II and 2 type I transmembrane serine/threonine kinases. Type II receptors phosphorylate and activate type I receptors which autophosphorylate, then bind and activate SMAD transcriptional regulators, SMAD2 and SMAD3. Receptor for activin AB, activin B and NODAL. Plays a role in cell differentiation, growth arrest and apoptosis
4	ACVR2A	9606.ENSP00000241416	ACVR2A	Activin receptor type-2A; On ligand binding, forms a receptor complex consisting of two type II and two type I transmembrane serine/threonine kinases. Type II receptors phosphorylate and activate type I receptors which autophosphorylate, then bind and activate SMAD transcriptional regulators. Receptor for activin A, activin B and inhibin A. Mediates induction of adipogenesis by GDF6 (By similarity); Belongs to the protein kinase superfamily. TKL Ser/Thr protein kinase family. TGFB receptor subfamily
5	ACVRL1	9606.ENSP00000373574	ACVRL1	Serine/threonine-protein kinase receptor R3; Type I receptor for TGF-beta family ligands BMP9/GDF2 and BMP10 and important regulator of normal blood vessel development. On ligand binding, forms a receptor complex consisting of two type II and two type I transmembrane serine/threonine kinases. Type II receptors phosphorylate and activate type I receptors which autophosphorylate, then bind and activate SMAD transcriptional regulators. May bind activin as well; Belongs to the protein kinase superfamily. TKL Ser/Thr protein kinase family. TGFB receptor subfamily
6	ADCYAP1	9606.ENSP00000462647	ADCYAP1	Pituitary adenylate cyclase-activating polypeptide; Binding to its receptor activates G proteins and stimulates adenylate cyclase in pituitary cells. Promotes neuron projection development through the RAPGEF2/Rap1/B-Raf/ERK pathway. In chromaffin cells, induces long-lasting increase of intracellular calcium concentrations and neuroendocrine secretion (By similarity). Involved in the control of glucose homeostasis, induces insulin secretion by pancreatic beta cells (By similarity); Belongs to the glucagon family
7	ADGB	9606.ENSP00000381036	ADGB	Androglobin; Calpains
8	ADGRG7	9606.ENSP00000273352	GPR128	Adhesion G-protein coupled receptor G7; Orphan receptor
9	AGBL4	9606.ENSP00000360905	AGBL4	Cytosolic carboxypeptidase 6; Metallocarboxypeptidase that mediates deglutamylation of target proteins. Catalyzes the deglutamylation of polyglutamate side chains generated by post-translational polyglutamylation in proteins such as tubulins. Also removes polyglutamates from the carboxy-terminus of target proteins such as MYLK. Mediates deglutamylation of CGAS/MB21D1, regulating the antiviral activity of CGAS/MB21D1. Acts as a long-chain deglutamylase and specifically shortens long polyglutamate chains, while it is not able to remove the branching point glutamate, a process catalyzed b [...]
10	AK1	9606.ENSP00000362271	AK1	Adenylate kinase isoenzyme 1; Catalyzes the reversible transfer of the terminal phosphate group between ATP and AMP. Also displays broad nucleoside diphosphate kinase activity. Plays an important role in cellular energy homeostasis and in adenine nucleotide metabolism; Belongs to the adenylate kinase family. AK1 subfamily
11	AMDHD2	9606.ENSP00000391596	AMDHD2	N-acetylglucosamine-6-phosphate deacetylase; Hydrolyzes the N-glycolyl group from N- glycolylglucosamine 6-phosphate (GlcNGc-6-P) in the N-glycolylneuraminic acid (Neu5Gc) degradation pathway. Although human is not able to catalyze formation of Neu5Gc due to the inactive CMAHP enzyme, Neu5Gc is present in food and must be degraded; Belongs to the metallo-dependent hydrolases superfamily. NagA family
12	ANGPT4	9606.ENSP00000371347	ANGPT4	Angiopoietin-4; Binds to TEK/TIE2, modulating ANGPT1 signaling. Can induce tyrosine phosphorylation of TEK/TIE2. Promotes endothelial cell survival, migration and angiogenesis; Fibrinogen C domain containing
13	ARHGAP10	9606.ENSP00000336923	ARHGAP10	Rho GTPase-activating protein 10; GTPase activator for the small GTPases RhoA and Cdc42 by converting them to an inactive GDP-bound state. Essential for PTKB2 regulation of cytoskeletal organization via Rho family GTPases. Inhibits PAK2 proteolytic fragment PAK-2p34 kinase activity and changes its localization from the nucleus to the perinuclear region. Stabilizes PAK-2p34 thereby increasing stimulation of cell death (By similarity)
14	ARHGAP5	9606.ENSP00000371897	ARHGAP5	Rho GTPase-activating protein 5; GTPase-activating protein for Rho family members. May play a role in the reduction of the p21rasGTPase-activating potential of RASA1/p120GAP
15	ARHGEF3	9606.ENSP00000341071	ARHGEF3	Rho guanine nucleotide exchange factor 3; Acts as guanine nucleotide exchange factor (GEF) for RhoA and RhoB GTPases
16	ARID3B	9606.ENSP00000343126	ARID3B	AT-rich interactive domain-containing protein 3B; Transcription factor which may be involved in neuroblastoma growth and malignant transformation. Favors nuclear targeting of ARID3A; AT-rich interaction domain containing
17	ARMCX5	9606.ENSP00000474720	ARMCX5	Armadillo repeat containing, X-linked 5
19	ATE1	9606.ENSP00000224652	ATE1	Arginyl-tRNA--protein transferase 1; Involved in the post-translational conjugation of arginine to the N-terminal aspartate or glutamate of a protein. This arginylation is required for degradation of the protein via the ubiquitin pathway. Does not arginylate cysteine residues (By similarity)
20	ATF7IP	9606.ENSP00000440440	ATF7IP	Activating transcription factor 7-interacting protein 1; Recruiter that couples transcriptional factors to general transcription apparatus and thereby modulates transcription regulation and chromatin formation. Can both act as an activator or a repressor depending on the context. Mediates MBD1-dependent transcriptional repression, probably by recruiting complexes containing SETDB1. Required to stimulate histone methyltransferase activity of SETDB1 and facilitate the conversion of dimethylated to trimethylated H3 'Lys-9' (H3K9me3). The complex formed with MBD1 and SETDB1 represses trans [...]
21	B3GAT1	9606.ENSP00000433847	B3GAT1	Galactosylgalactosylxylosylprotein 3-beta-glucuronosyltransferase 1; Involved in the biosynthesis of L2/HNK-1 carbohydrate epitope on glycoproteins. Can also play a role in glycosaminoglycan biosynthesis. Substrates include asialo- orosomucoid (ASOR), asialo-fetuin, and asialo-neural cell adhesion molecule. Requires sphingomyelin for activity: stearoyl- sphingomyelin was the most effective, followed by palmitoyl- sphingomyelin and lignoceroyl-sphingomyelin. Activity was demonstrated only for sphingomyelin with a saturated fatty acid and not for that with an unsaturated fatty acid, rega [...]
22	BANP	9606.ENSP00000376902	BANP	Protein BANP; Controls V(D)J recombination during T-cell development by repressing T-cell receptor (TCR) beta enhancer function. Binds to scaffold/matrix attachment region beta (S/MARbeta), an ATC-rich DNA sequence located upstream of the TCR beta enhancer. Represses cyclin D1 transcription by recruiting HDAC1 to its promoter, thereby diminishing H3K9ac, H3S10ph and H4K8ac levels. Promotes TP53 'Ser-15' phosphorylation and nuclear accumulation, which causes cell cycle arrest (By similarity); Belongs to the BANP/SMAR1 family
23	BCAT1	9606.ENSP00000443459	BCAT1	Branched-chain-amino-acid aminotransferase, cytosolic; Catalyzes the first reaction in the catabolism of the essential branched chain amino acids leucine, isoleucine, and valine
24	BCL11B	9606.ENSP00000349723	BCL11B	B-cell lymphoma/leukemia 11B; Key regulator of both differentiation and survival of T- lymphocytes during thymocyte development in mammals. Essential in controlling the responsiveness of hematopoietic stem cells to chemotactic signals by modulating the expression of the receptors CCR7 and CCR9, which direct the movement of progenitor cells from the bone marrow to the thymus. Is a regulator of IL2 promoter and enhances IL2 expression in activated CD4(+) T- lymphocytes. Tumor-suppressor that represses transcription through direct, TFCOUP2-independent binding to a GC- rich response elemen [...]
25	BEND7	9606.ENSP00000345773	BEND7	BEN domain containing 7
26	BRD8	9606.ENSP00000254900	BRD8	Bromodomain-containing protein 8; May act as a coactivator during transcriptional activation by hormone-activated nuclear receptors (NR). Isoform 2 stimulates transcriptional activation by AR/DHTR, ESR1/NR3A1, RXRA/NR2B1 and THRB/ERBA2. At least isoform 1 and isoform 2 are components of the NuA4 histone acetyltransferase (HAT) complex which is involved in transcriptional activation of select genes principally by acetylation of nucleosomal histones H4 and H2A. This modification may both alter nucleosome - DNA interactions and promote interaction of the modified histones with other prote [...]
27	BRINP1	9606.ENSP00000265922	BRINP1	BMP/retinoic acid-inducible neural-specific protein 1; Inhibits cell proliferation by negative regulation of the G1/S transition. Mediates cell death which is not of the classical apoptotic type and regulates expression of components of the plasminogen pathway; Belongs to the BRINP family
28	BTBD3	9606.ENSP00000384545	BTBD3	BTB/POZ domain-containing protein 3; Acts as a key regulator of dendritic field orientation during development of sensory cortex. Also directs dendrites toward active axon terminals when ectopically expressed (By similarity); BTB domain containing
29	C2orf27A	9606.ENSP00000347298	C2orf27A	Uncharacterized protein C2orf27; Chromosome 2 open reading frame 27A
30	CACNA1A	9606.ENSP00000353362	CACNA1A	Voltage-dependent P/Q-type calcium channel subunit alpha-1A; Voltage-sensitive calcium channels (VSCC) mediate the entry of calcium ions into excitable cells and are also involved in a variety of calcium-dependent processes, including muscle contraction, hormone or neurotransmitter release, gene expression, cell motility, cell division and cell death. The isoform alpha-1A gives rise to P and/or Q-type calcium currents. P/Q-type calcium channels belong to the 'high-voltage activated' (HVA) group and are blocked by the funnel toxin (Ftx) and by the omega-agatoxin- IVA (omega-Aga-IVA). Th [...]
31	CACNB2	9606.ENSP00000320025	CACNB2	Voltage-dependent L-type calcium channel subunit beta-2; The beta subunit of voltage-dependent calcium channels contributes to the function of the calcium channel by increasing peak calcium current, shifting the voltage dependencies of activation and inactivation, modulating G protein inhibition and controlling the alpha-1 subunit membrane targeting
32	CAMK1G	9606.ENSP00000009105	CAMK1G	Calcium/calmodulin-dependent protein kinase type 1G; Calcium/calmodulin-dependent protein kinase belonging to a proposed calcium-triggered signaling cascade. In vitro phosphorylates transcription factor CREB1 (By similarity); Belongs to the protein kinase superfamily. CAMK Ser/Thr protein kinase family. CaMK subfamily
33	CAMTA1	9606.ENSP00000306522	CAMTA1	Calmodulin-binding transcription activator 1; Transcriptional activator. May act as a tumor suppressor; Belongs to the CAMTA family
34	CAVIN2	9606.ENSP00000305675	SDPR	Caveolae-associated protein 2; Plays an important role in caveolar biogenesis and morphology. Regulates caveolae morphology by inducing membrane curvature within caveolae. Plays a role in caveola formation in a tissue-specific manner. Required for the formation of caveolae in the lung and fat endothelia but not in the heart endothelia. Negatively regulates the size or stability of CAVIN complexes in the lung endothelial cells. May play a role in targeting PRKCA to caveolae (By similarity)
35	CBFA2T3	9606.ENSP00000268679	CBFA2T3	Protein CBFA2T3; Transcriptional corepressor which facilitates transcriptional repression via its association with DNA-binding transcription factors and recruitment of other corepressors and histone-modifying enzymes. Can repress the expression of MMP7 in a ZBTB33-dependent manner. Reduces the protein levels and stability of the transcriptinal regulator HIF1A; interacts with EGLN1 and promotes the HIF1A prolyl hydroxylation-dependent ubiquitination and proteasomal degradation pathway. Contributes to inhibition of glycolysis and stimulation of mitochondrial respiration by down-regulatin [...]
36	CBLB	9606.ENSP00000264122	CBLB	E3 ubiquitin-protein ligase CBL-B; E3 ubiquitin-protein ligase which accepts ubiquitin from specific E2 ubiquitin-conjugating enzymes, and transfers it to substrates, generally promoting their degradation by the proteasome. Negatively regulates TCR (T-cell receptor), BCR (B- cell receptor) and FCER1 (high affinity immunoglobulin epsilon receptor) signal transduction pathways. In naive T-cells, inhibits VAV1 activation upon TCR engagement and imposes a requirement for CD28 costimulation for proliferation and IL-2 production. Also acts by promoting PIK3R1/p85 ubiquitination, which impair [...]
37	CBLN4	9606.ENSP00000064571	CBLN4	Cerebellin-4; May be involved in synaptic functions in the CNS. May play a role in CBLN3 export from the endoplasmic reticulum and secretion (By similarity)
38	CCDC105	9606.ENSP00000292574	CCDC105	Coiled-coil domain containing 105
39	CCDC149	9606.ENSP00000425715	CCDC149	Coiled-coil domain containing 149
40	CCDC182	9606.ENSP00000299415	CCDC182	Coiled-coil domain containing 182
41	CCDC185	9606.ENSP00000355840	CCDC185	Coiled-coil domain containing 185
42	CCKBR	9606.ENSP00000335544	CCKBR	Gastrin/cholecystokinin type B receptor; Receptor for gastrin and cholecystokinin. The CKK-B receptors occur throughout the central nervous system where they modulate anxiety, analgesia, arousal, and neuroleptic activity. This receptor mediates its action by association with G proteins that activate a phosphatidylinositol-calcium second messenger system; Belongs to the G-protein coupled receptor 1 family
43	CCN1	9606.ENSP00000274026	CCNA2	Cyclin-A2; Cyclin which controls both the G1/S and the G2/M transition phases of the cell cycle. Functions through the formation of specific serine/threonine protein kinase holoenzyme complexes with the cyclin-dependent protein kinases CDK1 or CDK2. The cyclin subunit confers the substrate specificity of these complexes and differentially interacts with and activates CDK1 and CDK2 throughout the cell cycle; Cyclins
44	CCR7	9606.ENSP00000246657	CCR7	C-C chemokine receptor type 7; Receptor for the MIP-3-beta chemokine. Probable mediator of EBV effects on B-lymphocytes or of normal lymphocyte functions; Belongs to the G-protein coupled receptor 1 family
45	CD53	9606.ENSP00000271324	CD53	Leukocyte surface antigen CD53; Required for efficient formation of myofibers in regenerating muscle at the level of cell fusion. May be involved in growth regulation in hematopoietic cells (By similarity); CD molecules
46	CD99L2	9606.ENSP00000480322	CD99L2	CD99 antigen-like protein 2; Plays a role in a late step of leukocyte extravasation helping cells to overcome the endothelial basement membrane. Acts at the same site as, but independently of, PECAM1 (By similarity). Homophilic adhesion molecule, but these interactions may not be required for cell aggregation (By similarity); Belongs to the CD99 family
47	CDC20B	9606.ENSP00000370781	CDC20B	Cell division cycle protein 20 homolog B; WD repeat domain containing; Belongs to the WD repeat CDC20/Fizzy family

#	queryItem	stringId	preferredName	annotation
48	CDH22	9606.ENSP00000437790	CDH22	Cadherin-22; Cadherins are calcium-dependent cell adhesion proteins. They preferentially interact with themselves in a homophilic manner in connecting cells; cadherins may thus contribute to the sorting of heterogeneous cell types. PB-cadherins may have a role in the morphological organization of pituitary gland and brain tissues (By similarity)
49	CDH26	9606.ENSP00000339390	CDH26	Cadherin-like protein 26; Cadherins are calcium-dependent cell adhesion proteins. They preferentially interact with themselves in a homophilic manner in connecting cells; cadherins may thus contribute to the sorting of heterogeneous cell types
50	CEACAM1	9606.ENSP00000161559	CEACAM1	Carcinoembryonic antigen-related cell adhesion molecule 1; Isoform 1: Cell adhesion protein that mediates homophilic cell adhesion in a calcium-independent manner (By similarity). Plays a role as coinhibitory receptor in immune response, insulin action and functions also as an activator during angiogenesis. Its coinhibitory receptor function is phosphorylation- and PTPN6 -dependent, which in turn, suppress signal transduction of associated receptors by dephosphorylation of their downstream effectors. Plays a role in immune response, of T cells, natural killer (NK) and neutrophils. Upon [...]
52	CHST11	9606.ENSP00000305725	CHST11	Carbohydrate sulfotransferase 11; Catalyzes the transfer of sulfate to position 4 of the N-acetylgalactosamine (GalNAc) residue of chondroitin. Chondroitin sulfate constitutes the predominant proteoglycan present in cartilage and is distributed on the surfaces of many cells and extracellular matrices. Can also sulfate Gal residues in desulfated dermatan sulfate. Preferentially sulfates in GlcA->GalNAc unit than in IdoA->GalNAc unit. Does not form 4, 6- di-O-sulfated GalNAc when chondroitin sulfate C is used as an acceptor; Belongs to the sulfotransferase 2 family
53	CHST8	9606.ENSP00000262622	CHST8	Carbohydrate sulfotransferase 8; Catalyzes the transfer of sulfate to position 4 of non- reducing N-acetylgalactosamine (GalNAc) residues in both N-glycans and O-glycans. Required for biosynthesis of glycoprotein hormones lutropin and thyrotropin, by mediating sulfation of their carbohydrate structures. Only active against terminal GalNAcbeta1,GalNAcbeta. Not active toward chondroitin; Belongs to the sulfotransferase 2 family
54	CLTCL1	9606.ENSP00000441158	CLTCL1	Clathrin heavy chain 2; Clathrin is the major protein of the polyhedral coat of coated pits and vesicles. Two different adapter protein complexes link the clathrin lattice either to the plasma membrane or to the trans-Golgi network (By similarity)
55	COL12A1	9606.ENSP00000325146	COL12A1	Collagen alpha-1(XII) chain; Type XII collagen interacts with type I collagen- containing fibrils, the COL1 domain could be associated with the surface of the fibrils, and the COL2 and NC3 domains may be localized in the perifibrillar matrix; Belongs to the fibril-associated collagens with interrupted helices (FACIT) family
56	CORO2A	9606.ENSP00000343746	CORO2A	Coronin-2A; WD repeat domain containing; Belongs to the WD repeat coronin family
57	COX6B2	9606.ENSP00000467266	COX6B2	Cytochrome c oxidase subunit 6B2; Connects the two COX monomers into the physiological dimeric form; Belongs to the cytochrome c oxidase subunit 6B family
58	CPLX2	9606.ENSP00000352544	CPLX2	Complexin-2; Negatively regulates the formation of synaptic vesicle clustering at active zone to the presynaptic membrane in postmitotic neurons. Positively regulates a late step in exocytosis of various cytoplasmic vesicles, such as synaptic vesicles and other secretory vesicles. Also involved in mast cell exocytosis (By similarity); Belongs to the complexin/synaphin family
59	CPNE4	9606.ENSP00000478878	CPNE4	Copine-4; Probable calcium-dependent phospholipid-binding protein that may play a role in calcium-mediated intracellular processes; Belongs to the copine family
60	CRIM1	9606.ENSP00000280527	CRIM1	Cysteine-rich motor neuron 1 protein; May play a role in CNS development by interacting with growth factors implicated in motor neuron differentiation and survival. May play a role in capillary formation and maintenance during angiogenesis. Modulates BMP activity by affecting its processing and delivery to the cell surface
61	CRYBG3	9606.ENSP00000374273	CRYBG3	Very large A-kinase anchor protein; Isoform vlAKAP: Anchoring protein that mediates the subcellular compartmentation of protein kinase A (PKA); Beta-gamma crystallin domain containing
62	CSDC2	9606.ENSP00000302485	CSDC2	Cold shock domain-containing protein C2; RNA-binding factor which binds specifically to the very 3'-UTR ends of both histone H1 and H3.3 mRNAs, encompassing the polyadenylation signal. Might play a central role in the negative regulation of histone variant synthesis in the developing brain (By similarity)
63	CSMD1	9606.ENSP00000430733	CSMD1	CUB and sushi domain-containing protein 1; Potential suppressor of squamous cell carcinomas; Protein phosphatase 1 regulatory subunits
64	CTNNBL1	9606.ENSP00000355050	CTNNBL1	Beta-catenin-like protein 1; Component of the PRP19-CDC5L complex that forms an integral part of the spliceosome and is required for activating pre-mRNA splicing. Participates in AID/AICDA-mediated Ig class switching recombination (CSR). May induce apoptosis; Armadillo-like helical domain containing
65	DAB2IP	9606.ENSP00000259371	DAB2IP	Disabled homolog 2-interacting protein; Functions as a scaffold protein implicated in the regulation of a large spectrum of both general and specialized signaling pathways. Involved in several processes such as innate immune response, inflammation and cell growth inhibition, apoptosis, cell survival, angiogenesis, cell migration and maturation. Plays also a role in cell cycle checkpoint control; reduces G1 phase cyclin levels resulting in G0/G1 cell cycle arrest. Mediates signal transduction by receptor-mediated inflammatory signals, such as the tumor necrosis factor (TNF), interferon [...]
66	DBX1	9606.ENSP00000436881	DBX1	Homeobox protein DBX1; Could have a role in patterning the central nervous system during embryogenesis. Has a key role in regulating the distinct phenotypic features that distinguish two major classes of ventral interneurons, V0 and V1 neurons. Regulates the transcription factor profile, neurotransmitter phenotype, intraspinal migratory path and axonal trajectory of V0 neurons, features that differentiate them from an adjacent set of V1 neurons (By similarity); NKL subclass homeoboxes and pseudogenes
67	DCDC2C	9606.ENSP00000382097	DCDC2C	Doublecortin domain containing 2C
68	DCN	9606.ENSP00000052754	DCN	Decorin; May affect the rate of fibrils formation; Small leucine rich repeat proteoglycans
69	DDIT4	9606.ENSP00000307305	DDIT4	DNA damage-inducible transcript 4 protein; Regulates cell growth, proliferation and survival via inhibition of the activity of the mammalian target of rapamycin complex 1 (mTORC1). Inhibition of mTORC1 is mediated by a pathway that involves DDIT4/REDD1, AKT1, the TSC1-TSC2 complex and the GTPase RHEB. Plays an important role in responses to cellular energy levels and cellular stress, including responses to hypoxia and DNA damage. Regulates p53/TP53-mediated apoptosis in response to DNA damage via its effect on mTORC1 activity. Its role in the response to hypoxia depends on the cell typ [...]
70	DEFA4	9606.ENSP00000297435	DEFA4	Neutrophil defensin 4; Has antimicrobial activity against Gram-negative bacteria, and to a lesser extent also against Gram-positive bacteria and fungi. Protects blood cells against infection with HIV-1 (in vitro). Inhibits corticotropin (ACTH)-stimulated corticosterone production; Defensins, alpha
71	DENND2B	9606.ENSP00000433528	ST5	Suppression of tumorigenicity 5 protein; Guanine nucleotide exchange factor (GEF) which may activate RAB9A and RAB9B. Promotes the exchange of GDP to GTP, converting inactive GDP-bound Rab proteins into their active GTP- bound form. May be involved in cytoskeletal organization and tumorogenicity. Isoform 1 seems to be involved in a signaling transduction pathway leading to activation of MAPK1/ERK2. Isoform 3 may block ERK2 activation stimulated by ABL1. Isoform 3 may alter cell morphology and cell growth; DENN/MADD domain containing
72	DLX5	9606.ENSP00000222598	DLX5	Homeobox protein DLX-5; Transcriptional factor involved in bone development. Acts as an immediate early BMP-responsive transcriptional activator essential for osteoblast differentiation. Stimulates ALPL promoter activity in a RUNX2-independent manner during osteoblast differentiation. Stimulates SP7 promoter activity during osteoblast differentiation. Promotes cell proliferation by up-regulating MYC promoter activity. Involved as a positive regulator of both chondrogenesis and chondrocyte hypertrophy in the endochondral skeleton. Binds to the homeodomain-response element of the ALPL an [...]
73	DMBT1	9606.ENSP00000357905	DMBT1	Deleted in malignant brain tumors 1 protein; May be considered as a candidate tumor suppressor gene for brain, lung, esophageal, gastric, and colorectal cancers. May play roles in mucosal defense system, cellular immune defense and epithelial differentiation. May play a role as an opsonin receptor for SFTPD and SPAR in macrophage tissues throughout the body, including epithelial cells lining the gastrointestinal tract. May play a role in liver regeneration. May be an important factor in fate decision and differentiation of transit-amplifying ductular (oval) cells within the hepatic lin [...]
74	DMTN	9606.ENSP00000427866	DMTN	Dematin; Membrane-cytoskeleton-associated protein with F-actin- binding activity that induces F-actin bundles formation and stabilization. Its F-actin-bundling activity is reversibly regulated upon its phosphorylation by the cAMP-dependent protein kinase A (PKA). Binds to the erythrocyte membrane glucose transporter-1 SLC2A1/GLUT1, and hence stabilizes and attaches the spectrin-actin network to the erythrocytic plasma membrane. Plays a role in maintaining the functional integrity of PKA-activated erythrocyte shape and the membrane mechanical properties. Plays also a role as a modulator [...]
75	DNAJC14	9606.ENSP00000350223	DNAJC14	DnaJ homolog subfamily C member 14; Regulates the export of target proteins, such as DRD1, from the endoplasmic reticulum to the cell surface; DNAJ heat shock proteins
76	DNASE2B	9606.ENSP00000359699	DNASE2B	Deoxyribonuclease-2-beta; Hydrolyzes DNA under acidic conditions. Does not require divalent cations for activity. Participates in the degradation of nuclear DNA during lens cell differentiation
77	DPH1	9606.ENSP00000263083	DPH1	2-(3-amino-3-carboxypropyl)histidine synthase subunit 1; Required for the first step in the synthesis of diphthamide, a post-translational modification of histidine which occurs in translation elongation factor 2 (EEF2). When overexpressed, suppresses colony formation ability and growth rate of ovarian cancer cells. Acts also as a tumor suppressor in lung and breast cancers (By similarity). Plays a role in embryonic growth, organogenesis and postnatal survival (By similarity); Minor histocompatibility antigens
78	DSG3	9606.ENSP00000257189	DSG3	Desmoglein-3; Component of intercellular desmosome junctions. Involved in the interaction of plaque proteins and intermediate filaments mediating cell-cell adhesion; Desmosomal cadherins
79	DTX3	9606.ENSP00000448696	DTX3	Probable E3 ubiquitin-protein ligase DTX3; Regulator of Notch signaling, a signaling pathway involved in cell-cell communications that regulates a broad spectrum of cell-fate determinations. Probably acts both as a positive and negative regulator of Notch, depending on the developmental and cell context (By similarity). Functions as an ubiquitin ligase protein in vitro, suggesting that it may regulate the Notch pathway via some ubiquitin ligase activity; Ring finger proteins
80	E4F1	9606.ENSP00000301727	E4F1	Transcription factor E4F1; May function as a transcriptional repressor. May also function as a ubiquitin ligase mediating ubiquitination of chromatin-associated TP53. Functions in cell survival and proliferation through control of the cell cycle. Functions in the p53 and pRB tumor suppressor pathways and regulates the cyclin CCNA2 transcription; Zinc fingers C2H2-type
81	EEF1E1	9606.ENSP00000369038	EEF1E1	Eukaryotic translation elongation factor 1 epsilon-1; Positive modulator of ATM response to DNA damage
82	EFCAB11	9606.ENSP00000326267	EFCAB11	EF-hand calcium-binding domain-containing protein 11; EF-hand calcium binding domain 11
83	EMILIN2	9606.ENSP00000254528	EMILIN2	EMILIN-2; May be responsible for anchoring smooth muscle cells to elastic fibers, and may be involved not only in the formation of the elastic fiber, but also in the processes that regulate vessel assembly. Has cell adhesive capacity; EMI domain containing
84	ENTPD7	9606.ENSP00000359520	ENTPD7	Ectonucleoside triphosphate diphosphohydrolase 7; Preferentially hydrolyzes nucleoside 5'-triphosphates. The order of activity with respect to possible substrates is UTP > GTP > CTP
85	EPHA2	9606.ENSP00000351209	EPHA2	Ephrin type-A receptor 2; Receptor tyrosine kinase which binds promiscuously membrane-bound ephrin-A family ligands residing on adjacent cells, leading to contact-dependent bidirectional signaling into neighboring cells. The signaling pathway downstream of the receptor is referred to as forward signaling while the signaling pathway downstream of the ephrin ligand is referred to as reverse signaling. Activated by the ligand ephrin-A1/EFNA1 regulates migration, integrin-mediated adhesion, proliferation and differentiation of cells. Regulates cell adhesion and differentiation through DSG1 [...]
86	EPHB3	9606.ENSP00000332118	EPHB3	Ephrin type-B receptor 3; Receptor tyrosine kinase which binds promiscuously transmembrane ephrin-B family ligands residing on adjacent cells, leading to contact-dependent bidirectional signaling into neighboring cells. The signaling pathway downstream of the receptor is referred to as forward signaling while the signaling pathway downstream of the ephrin ligand is referred to as reverse signaling. Generally has an overlapping and redundant function with EPHB2. Like EPHB2, functions in axon guidance during development regulating for instance the neurons forming the corpus callosum and [...]
87	ERLIN2	9606.ENSP00000276461	ERLIN2	Erlin-2; Component of the ERLIN1/ERLIN2 complex which mediates the endoplasmic reticulum-associated degradation (ERAD) of inositol 1,4,5-trisphosphate receptors (IP3Rs) such as ITPR1. Promotes sterol-accelerated ERAD of HMGCR probably implicating an AMFR/gp78-containing ubiquitin ligase complex. Involved in regulation of cellular cholesterol homeostasis by regulation the SREBP signaling pathway. May promote ER retention of the SCAP-SREBF complex
88	ERRFI1	9606.ENSP00000366702	ERRFI1	ERBB receptor feedback inhibitor 1; Negative regulator of EGFR signaling in skin morphogenesis. Acts as a negative regulator for several EGFR family members, including ERBB2, ERBB3 and ERBB4. Inhibits EGFR catalytic activity by interfering with its dimerization. Inhibits autophosphorylation of EGFR, ERBB2 and ERBB4. Important for normal keratinocyte proliferation and differentiation. Plays a role in modulating the response to steroid hormones in the uterus. Required for normal response to progesterone in the uterus and for fertility. Mediates epithelial estrogen responses in the uterus [...]
89	F11R	9606.ENSP00000357005	F11R	Junctional adhesion molecule A; Seems to play a role in epithelial tight junction formation. Appears early in primordial forms of cell junctions and recruits PARD3. The association of the PARD6- PARD3 complex may prevent the interaction of PARD3 with JAM1, thereby preventing tight junction assembly (By similarity). Plays a role in regulating monocyte transmigration involved in integrity of epithelial barrier (By similarity). Ligand for integrin alpha-L/beta-2 involved in memory T-cell and neutrophil transmigration. Involved in platelet activation; Belongs to the immunoglobulin superfamily
90	FAAH2	9606.ENSP00000364035	FAAH2	Fatty-acid amide hydrolase 2; Degrades bioactive fatty acid amides like oleamide, the endogenous cannabinoid, anandamide and myristic amide to their corresponding acids, thereby serving to terminate the signaling functions of these molecules. Hydrolyzes monounsaturated substrate anandamide preferentially as compared to polyunsaturated substrates
91	FAM110B	9606.ENSP00000355204	FAM110B	Protein FAM110B; May be involved in tumor progression
92	FAM155B	9606.ENSP00000252338	FAM155B	Transmembrane protein FAM155B; Family with sequence similarity 155 member B; Belongs to the FAM155 family
93	FAM169A	9606.ENSP00000373808	FAM169A	Soluble lamin-associated protein of 75 kDa; Family with sequence similarity 169 member A; Belongs to the FAM169 family
94	FAM98C	9606.ENSP00000252530	FAM98C	Protein FAM98C; Family with sequence similarity 98 member C

Feelings of Guilt (AAGS)

#	queryItem	stringId	preferredName	annotation
95	FBXO34	9606.ENSP00000313159	FBXO34	F-box only protein 34; Substrate-recognition component of the SCF (SKP1-CUL1-F- box protein)-type E3 ubiquitin ligase complex; F-boxes other
96	FNDC7	9606.ENSP00000359034	FNDC7	Fibronectin type III domain containing 7
97	FOLH1	9606.ENSP00000256999	FOLH1	Glutamate carboxypeptidase 2; Has both folate hydrolase and N-acetylated-alpha-linked- acidic dipeptidase (NAALADase) activity. Has a preference for tri- alpha-glutamate peptides. In the intestine, required for the uptake of folate. In the brain, modulates excitatory neurotransmission through the hydrolysis of the neuropeptide, N- aceylaspartylglutamate (NAAG), thereby releasing glutamate. Involved in prostate tumor progression
98	FOSL2	9606.ENSP00000264716	FOSL2	Fos-related antigen 2; Controls osteoclast survival and size. As a dimer with JUN, activates LIF transcription. Activates CEBPB transcription in PGE2-activated osteoblasts; Basic leucine zipper proteins
99	FOXL1	9606.ENSP00000326272	FOXL1	Forkhead box protein L1; Transcription factor required for proper proliferation and differentiation in the gastrointestinal epithelium. Target gene of the hedgehog (Hh) signaling pathway via GLI2 AND GLI3 transcription factors (By similarity); Forkhead boxes
100	FZD3	9606.ENSP00000437489	FZD3	Frizzled-3; Receptor for Wnt proteins. Most of frizzled receptors are coupled to the beta-catenin canonical signaling pathway, which leads to the activation of disheveled proteins, inhibition of GSK- 3 kinase, nuclear accumulation of beta-catenin and activation of Wnt target genes. A second signaling pathway involving PKC and calcium fluxes has been seen for some family members, but it is not yet clear if it represents a distinct pathway or if it can be integrated in the canonical pathway, as PKC seems to be required for Wnt-mediated inactivation of GSK-3 kinase. Both pathways seem to [...]
101	GADD45G	9606.ENSP00000252506	GADD45G	Growth arrest and DNA damage-inducible protein GADD45 gamma; Involved in the regulation of growth and apoptosis. Mediates activation of stress-responsive MTK1/MEKK4 MAPKKK; Belongs to the GADD45 family
102	GAL3ST2	9606.ENSP00000192314	GAL3ST2	Galactose-3-O-sulfotransferase 2; Transfers a sulfate group to the hydroxyl group at C3 of non-reducing beta-galactosyl residues. Acts both on type 1 (Gal- beta-1,3-GlcNAc) and type 2 (Gal-beta-1,4-GlcNAc) chains with similar efficiency; Sulfotransferases, membrane bound
103	GAL3ST3	9606.ENSP00000308591	GAL3ST3	Galactose-3-O-sulfotransferase 3; Transfers a sulfate to position 3 of non-reducing beta- galactosyl residues in N-glycans and core2-branched O-glycans. Has high activity towards Gal-beta-1,4-GlcNAc, Gal-beta-1,4(Fuc-alpha- 1,3)GlcNAc and lower activity towards Gal-beta-1,3(Fuc-alpha- 1,4)GlcNAc; Belongs to the galactose-3-O-sulfotransferase family
104	GALNT11	9606.ENSP00000416787	GALNT11	Polypeptide N-acetylgalactosaminyltransferase 11; Polypeptide N-acetylgalactosaminyltransferase that catalyzes the initiation of protein O-linked glycosylation and is involved in left/right asymmetry by mediating O-glycosylation of NOTCH1. O-glycosylation of NOTCH1 promotes activation of NOTCH1, modulating the balance between motile and immotile (sensory) cilia at the left-right organiser (LRO). Polypeptide N-acetylgalactosaminyltransferases catalyze the transfer of an N- acetyl-D-galactosamine residue to a serine or threonine residue on the protein receptor. Displays the same enzyme [...]
105	GATAD2A	9606.ENSP00000353463	GATAD2A	Transcriptional repressor p66-alpha; Transcriptional repressor. Enhances MBD2-mediated repression. Efficient repression requires the presence of GATAD2B; GATA zinc finger domain containing
106	GCNT4	9606.ENSP00000317027	GCNT4	Beta-1,3-galactosyl-O-glycosyl-glycoprotein beta-1,6-N-acetylglucosaminyltransferase 4; Glycosyltransferase that mediates core 2 O-glycan branching, an important step in mucin-type biosynthesis. Does not have core 4 O-glycan or I-branching enzyme activity; Glucosaminyl transferases/xylosyltransferases
107	GK2	9606.ENSP00000351706	GK2	Glycerol kinase 2; Key enzyme in the regulation of glycerol uptake and metabolism; Belongs to the FGGY kinase family
108	GLI2	9606.ENSP00000390436	GLI2	Zinc finger protein GLI2; Functions as transcription regulator in the hedgehog (Hh) pathway. Functions as transcriptional activator. May also function as transcriptional repressor (By similarity). Requires STK36 for full transcriptional activator activity. Required for normal embryonic development; Zinc fingers C2H2-type
109	GNG7	9606.ENSP00000371594	GNG7	Guanine nucleotide-binding protein G(I)/G(S)/G(O) subunit gamma-7; Guanine nucleotide-binding proteins (G proteins) are involved as a modulator or transducer in various transmembrane signaling systems. The beta and gamma chains are required for the GTPase activity, for replacement of GDP by GTP, and for G protein- effector interaction. Plays a role in the regulation of adenylyl cyclase signaling in certain regions of the brain. Plays a role in the formation or stabilzation of a G protein heterotrimer (G(olf) subunit alpha-beta-gamma-7) that is required for adenylyl cyclase activity in [...]
110	GNPAT	9606.ENSP00000355607	GNPAT	Glyceronephosphate O-acyltransferase
111	GPM6A	9606.ENSP00000280187	GPM6A	Neuronal membrane glycoprotein M6-a; Involved in neuronal differentiation, including differentiation and migration of neuronal stem cells. Plays a role in neuronal plasticity and is involved in neurite and filopodia outgrowth, filopodia motility and probably synapse formation. GPM6A-induced filopodia formation involves mitogen-activated protein kinase (MAPK) and Src signaling pathways. May be involved in neuronal NGF-dependent Ca(2+) influx. May be involved in regulation of endocytosis and intracellular trafficking of G- protein-coupled receptors (GPCRs); enhances internalization and r [...]
112	GPR12	9606.ENSP00000384932	GPR12	G-protein coupled receptor 12; Promotes neurite outgrowth and blocks myelin inhibition in neurons (By similarity). Receptor with constitutive G(s) signaling activity that stimulates cyclic AMP production; G protein-coupled receptors, Class A orphans
113	GRAMD2	9606.ENSP00000311657	GRAMD2	GRAM domain-containing protein 2A; GRAM domain containing 2
114	GRIK4	9606.ENSP00000435648	GRIK4	Glutamate receptor ionotropic, kainate 4; Receptor for glutamate. L-glutamate acts as an excitatory neurotransmitter at many synapses in the central nervous system. The postsynaptic actions of Glu are mediated by a variety of receptors that are named according to their selective agonists; Glutamate ionotropic receptor kainate type subunits
115	GSE1	9606.ENSP00000253458	GSE1	Genetic suppressor element 1; Gse1 coiled-coil protein
116	HIVEP1	9606.ENSP00000368698	HIVEP1	Zinc finger protein 40; This protein specifically binds to the DNA sequence 5'- GGGACTTTCC-3' which is found in the enhancer elements of numerous viral promoters such as those of SV40, CMV, or HIV-1. In addition, related sequences are found in the enhancer elements of a number of cellular promoters, including those of the class I MHC, interleukin-2 receptor, and interferon-beta genes. It may act in T-cell activation. Involved in activating HIV-1 gene expression. Isoform 2 and isoform 3 also bind to the IPCS (IRF1 and p53 common sequence) DNA sequence in the promoter region of interfero [...]
117	HLA-DQB2	9606.ENSP00000390431	HLA-DQB2	Major histocompatibility complex, class II, DQ beta 2; Belongs to the MHC class II family
118	HNRNPLL	9606.ENSP00000390625	HNRNPLL	Heterogeneous nuclear ribonucleoprotein L-like; RNA-binding protein that functions as regulator of alternative splicing for multiple target mRNAs, including PTPRC/CD45 and STAT5A. Required for alternative splicing of PTPRC
119	HS2ST1	9606.ENSP00000359581	HS2ST1	Heparan sulfate 2-O-sulfotransferase 1; Catalyzes the transfer of sulfate to the C2-position of selected hexuronic acid residues within the maturing heparan sulfate (HS). 2-O-sulfation within HS, particularly of iduronate residues, is essential for HS to participate in a variety of high- affinity ligand-binding interactions and signaling processes. Mediates 2-O-sulfation of both L-iduronyl and D-glucuronyl residues (By similarity); Sulfotransferases, membrane bound
120	HUS1	9606.ENSP00000258774	HUS1	Checkpoint protein HUS1; Component of the 9-1-1 cell-cycle checkpoint response complex that plays a major role in DNA repair. The 9-1-1 complex is recruited to DNA lesion upon damage by the RAD17-replication factor C (RFC) clamp loader complex. Acts then as a sliding clamp platform on DNA for several proteins involved in long-patch base excision repair (LP-BER). The 9-1-1 complex stimulates DNA polymerase beta (POLB) activity by increasing its affinity for the 3'-OH end of the primer-template and stabilizes POLB to those sites where LP-BER proceeds; endonuclease FEN1 cleavage activity [...]
121	IFT22	9606.ENSP00000320359	IFT22	Intraflagellar transport protein 22 homolog; Small GTPase-like component of the intraflagellar transport (IFT) complex B; Belongs to the small GTPase superfamily. Rab family
122	IGFBP5	9606.ENSP00000233813	IGFBP5	Insulin-like growth factor-binding protein 5; IGF-binding proteins prolong the half-life of the IGFs and have been shown to either inhibit or stimulate the growth promoting effects of the IGFs on cell culture. They alter the interaction of IGFs with their cell surface receptors
125	IGSF1	9606.ENSP00000359940	IGSF1	Immunoglobulin superfamily member 1; Seems to be a coreceptor in inhibin signaling, but seems not to be a high-affinity inhibin receptor. Antagonizes activin A signaling in the presence or absence of inhibin B (By similarity). Necessary to mediate a specific antagonistic effect of inhibin B on activin-stimulated transcription; Immunoglobulin like domain containing
126	IL5RA	9606.ENSP00000412209	IL5RA	Interleukin-5 receptor subunit alpha; This is the receptor for interleukin-5. The alpha chain binds to IL5; Belongs to the type I cytokine receptor family. Type 5 subfamily
127	ING4	9606.ENSP00000380024	ING4	Inhibitor of growth protein 4; Component of the HBO1 complex which has a histone H4- specific acetyltransferase activity, a reduced activity toward histone H3 and is responsible for the bulk of histone H4 acetylation in vivo. Through chromatin acetylation it may function in DNA replication. May inhibit tumor progression by modulating the transcriptional output of signaling pathways which regulate cell proliferation. Can suppress brain tumor angiogenesis through transcriptional repression of RELA/NFKB3 target genes when complexed with RELA. May also specifically suppress loss of contact [...]
128	INPP5B	9606.ENSP00000362115	INPP5B	Type II inositol 1,4,5-trisphosphate 5-phosphatase; Hydrolyzes phosphatidylinositol 4,5-bisphosphate (PtIns(4,5)P2) and the signaling molecule phosphatidylinositol 1,4,5-trisphosphate (PtIns(1,4,5)P3), and thereby modulates cellular signaling events; Phosphoinositide phosphatases
129	INSIG2	9606.ENSP00000245787	INSIG2	Insulin-induced gene 2 protein; Mediates feedback control of cholesterol synthesis by controlling SCAP and HMGCR. Functions by blocking the processing of sterol regulatory element-binding proteins (SREBPs). Capable of retaining the SCAP-SREBF2 complex in the ER thus preventing it from escorting SREBPs to the Golgi. Seems to regulate the ubiquitin-mediated proteasomal degradation of HMGCR
130	INTS6L	9606.ENSP00000359788	DDX26B	Integrator complex subunit 6-like; DEAD/H (Asp-Glu-Ala-Asp/His) box polypeptide 26B
131	ISM1	9606.ENSP00000262487	ISM1	Isthmin-1; Acts as an angiogenesis inhibitor; Belongs to the isthmin family
132	KCNAB2	9606.ENSP00000367323	KCNAB2	Voltage-gated potassium channel subunit beta-2; Cytoplasmic potassium channel subunit that modulates the characteristics of the channel-forming alpha-subunits. Contributes to the regulation of nerve signaling, and prevents neuronal hyperexcitability (By similarity). Promotes expression of the pore-forming alpha subunits at the cell membrane, and thereby increases channel activity (By similarity). Promotes potassium channel closure via a mechanism that does not involve physical obstruction of the channel pore. Promotes KCNA4 channel closure. Modulates the functional properties of KCNA5 [...]
133	KCNB2	9606.ENSP00000430846	KCNB2	Potassium voltage-gated channel subfamily B member 2; Voltage-gated potassium channel that mediates transmembrane potassium transport in excitable membranes, primarily in the brain and smooth muscle cells. Channels open or close in response to the voltage difference across the membrane, letting potassium ions pass in accordance with their electrochemical gradient. Homotetrameric channels mediate a delayed-rectifier voltage-dependent outward potassium current that display rapid activation and slow inactivation in response to membrane depolarization. Can form functional homotetrameric an [...]
134	KCNH7	9606.ENSP00000331727	KCNH7	Potassium voltage-gated channel subfamily H member 7; Pore-forming (alpha) subunit of voltage-gated potassium channel. Channel properties may be modulated by cAMP and subunit assembly; Belongs to the potassium channel family. H (Eag) (TC 1.A.1.20) subfamily. Kv11.3/KCNH7 sub-subfamily
135	KCNIP4	9606.ENSP00000371587	KCNIP4	Kv channel-interacting protein 4; Regulatory subunit of Kv4/D (Shal)-type voltage-gated rapidly inactivating A-type potassium channels. Modulates KCND2 channel density, inactivation kinetics and rate of recovery from inactivation in a calcium-dependent and isoform-specific manner. Modulates KCND3/Kv4.3 currents. Isoform 4 does not increase KCND2 expression at the cell membrane. Isoform 4 retains KCND3 in the endoplasmic reticulum and negatively regulates its expression at the cell membrane; Belongs to the recoverin family
136	KLK7	9606.ENSP00000375683	KLK7	Kallikrein-7; May catalyze the degradation of intercellular cohesive structures in the cornified layer of the skin in the continuous shedding of cells from the skin surface. Specific for amino acid residues with aromatic side chains in the P1 position. Cleaves insulin A chain at '14-Tyr-|-Gln-15' and insulin B chain at '6- Leu-|-Cys-7', '16-Tyr-|-Leu-17', '25-Phe-|-Tyr-26' and '26-Tyr-|- Thr-27'. Could play a role in the activation of precursors to inflammatory cytokines; Kallikreins
137	LAPTM5	9606.ENSP00000294507	LAPTM5	Lysosomal-associated transmembrane protein 5; May have a special functional role during embryogenesis and in adult hematopoietic cells; Belongs to the LAPTM4/LAPTM5 transporter family
138	LBX1	9606.ENSP00000359212	LBX1	Transcription factor LBX1; Transcription factor required for the development of GABAergic interneurons in the dorsal horn of the spinal cord and migration and further development of hypaxial muscle precursor cells for limb muscles, diaphragm and hypoglossal cord; NKL subclass homeoboxes and pseudogenes
139	LCE3A	9606.ENSP00000335006	LCE3A	Late cornified envelope protein 3A; Precursors of the cornified envelope of the stratum corneum; Belongs to the LCE family
140	LDLRAP1	9606.ENSP00000363458	LDLRAP1	Low density lipoprotein receptor adapter protein 1; Adapter protein (clathrin-associated sorting protein (CLASP)) required for efficient endocytosis of the LDL receptor (LDLR) in polarized cells such as hepatocytes and lymphocytes, but not in non-polarized cells (fibroblasts). May be required for LDL binding and internalization but not for receptor clustering in coated pits. May facilitate the endocytocis of LDLR and LDLR-LDL complexes from coated pits by stabilizing the interaction between the receptor and the structural components of the pits. May also be involved in the internalizat [...]
141	LECT2	9606.ENSP00000274507	LECT2	Leukocyte cell-derived chemotaxin-2; Has a neutrophil chemotactic activity. Also a positive regulator of chondrocyte proliferation. Does not show metalloendopeptidase activity; Belongs to the LECT2/MIM-1 family
142	LEP	9606.ENSP00000312652	LEP	Leptin; Key player in the regulation of energy balance and body weight control. Once released into the circulation, has central and peripheral effects by binding LEPR, found in many tissues, which results in the activation of several major signaling pathways. In the hypothalamus, acts as an appetite-regulating factor that induces a decrease in food intake and an increase in energy consumption by inducing anorexinogenic factors and suppressing orexigenic neuropeptides, also regulates bone mass and secretion of hypothalamo-pituitary-adrenal hormones. In the periphery, increases basal met [...]
143	LGMN	9606.ENSP00000376911	LGMN	Legumain; Has a strict specificity for hydrolysis of asparaginyl bonds. Can also cleave aspartyl bonds slowly, especially under acidic conditions. Required for normal lysosomal protein degradation in renal proximal tubules. Required for normal degradation of internalized EGFR. Plays a role in the regulation of cell proliferation via its role in EGFR degradation (By similarity). May be involved in the processing of proteins for MHC class II antigen presentation in the lysosomal/endosomal system; Belongs to the peptidase C13 family

Feelings of Guilt (AAGS)

#	queryItem	stringId	preferredName	annotation
144	LHX2	9606.ENSP00000362717	LHX2	LIM/homeobox protein Lhx2; Acts as a transcriptional activator. Stimulates the promoter of the alpha-glycoprotein gene. Transcriptional regulatory protein involved in the control of cell differentiation in developing lymphoid and neural cell types (By similarity); LIM class homeoboxes
145	LMAN2	9606.ENSP00000303366	LMAN2	Vesicular integral-membrane protein VIP36; Plays a role as an intracellular lectin in the early secretory pathway. Interacts with N-acetyl-D-galactosamine and high-mannose type glycans and may also bind to O-linked glycans. Involved in the transport and sorting of glycoproteins carrying high mannose-type glycans (By similarity)
146	LNPEP	9606.ENSP00000231368	LNPEP	Leucyl-cystinyl aminopeptidase; Release of an N-terminal amino acid, cleaves before cysteine, leucine as well as other amino acids. Degrades peptide hormones such as oxytocin, vasopressin and angiotensin III, and plays a role in maintaining homeostasis during pregnancy. May be involved in the inactivation of neuronal peptides in the brain. Cleaves Met-enkephalin and dynorphin. Binds angiotensin IV and may be the angiotensin IV receptor in the brain; Aminopeptidases
147	LPCAT1	9606.ENSP00000283415	LPCAT1	Lysophosphatidylcholine acyltransferase 1; Possesses both acyltransferase and acetyltransferase activities. Activity is calcium-independent (By similarity). Mediates the conversion of 1- acyl-sn-glycero-3-phosphocholine (LPC) into phosphatidylcholine (PC). Displays a clear preference for saturated fatty acyl-CoAs, and 1-myristoyl or 1-palmitoyl LPC as acyl donors and acceptors, respectively. May synthesize phosphatidylcholine in pulmonary surfactant, thereby playing a pivotal role in respiratory physiology. Involved in the regulation of lipid droplet number and size
148	LRRC26	9606.ENSP00000360597	LRRC26	Leucine-rich repeat-containing protein 26; Auxiliary protein of the large-conductance, voltage and calcium-activated potassium channel (BK alpha). Required for the conversion of BK alpha channels from a high-voltage to a low- voltage activated channel type in non-excitable cells. These are characterized by negative membrane voltages and constant low levels of calcium
149	LRRC74B	9606.ENSP00000394078	LRRC74B	Leucine-rich repeat-containing protein 74B; Leucine rich repeat containing 74B
150	LUM	9606.ENSP00000266718	LUM	Lumican; Small leucine rich repeat proteoglycans; Belongs to the small leucine-rich proteoglycan (SLRP) family. SLRP class II subfamily
151	MACROD2	9606.ENSP00000217246	MACROD2	O-acetyl-ADP-ribose deacetylase MACROD2; Removes ADP-ribose from glutamate residues in proteins bearing a single ADP-ribose moiety. Inactive towards proteins bearing poly-ADP-ribose. Deacetylates O-acetyl-ADP ribose, a signaling molecule generated by the deacetylation of acetylated lysine residues in histones and other proteins
152	MAGEA10	9606.ENSP00000244096	MAGEA10	Melanoma-associated antigen 10; Not known, though may play a role in embryonal development and tumor transformation or aspects of tumor progression; MAGE family
153	MAN2B1	9606.ENSP00000395473	MAN2B1	Lysosomal alpha-mannosidase; Necessary for the catabolism of N-linked carbohydrates released during glycoprotein turnover. Cleaves all known types of alpha-mannosidic linkages; Belongs to the glycosyl hydrolase 38 family
154	MAPK4	9606.ENSP00000383234	MAPK4	Mitogen-activated protein kinase 4; Atypical MAPK protein. Phosphorylates microtubule- associated protein 2 (MAP2) and MAPKAPK5. The precise role of the complex formed with MAPKAPK5 is still unclear, but the complex follows a complex set of phosphorylation events: upon interaction with atypical MAPKAPK5, ERK4/MAPK4 is phosphorylated at Ser-186 and then mediates phosphorylation and activation of MAPKAPK5, which in turn phosphorylates ERK4/MAPK4. May promote entry in the cell cycle (By similarity); Mitogen-activated protein kinases
155	MAPRE1	9606.ENSP00000364721	MAPRE1	Microtubule-associated protein RP/EB family member 1; Plus-end tracking protein (+TIP) that binds to the plus- end of microtubules and regulates the dynamics of the microtubule cytoskeleton. Promotes cytoplasmic microtubule nucleation and elongation. May be involved in spindle function by stabilizing microtubules and anchoring them at centrosomes. Also acts as a regulator of minus-end microtubule organization: interacts with the complex formed by AKAP9 and PDE4DIP, leading to recruit CAMSAP2 to the Golgi apparatus, thereby tethering non-centrosomal minus-end microtubules to the Golgi, [...]
156	MAST4	9606.ENSP00000385727	MAST4	Microtubule associated serine/threonine kinase family member 4; PDZ domain containing
157	MBP	9606.ENSP00000380958	MBP	Myelin basic protein; The classic group of MBP isoforms (isoform 4-isoform 14) are with PLP the most abundant protein components of the myelin membrane in the CNS. They have a role in both its formation and stabilization. The smaller isoforms might have an important role in remyelination of denuded axons in multiple sclerosis. The non- classic group of MBP isoforms (isoform 1-isoform 3/Golli-MBPs) may preferentially have a role in the early developing brain long before myelination, maybe as components of transcriptional complexes, and may also be involved in signaling pathways in T-ce [...]
158	MCTP2	9606.ENSP00000350377	MCTP2	Multiple C2 and transmembrane domain-containing protein 2; Might play a role in the development of cardiac outflow tract; Multiple C2 and transmembrane domain containing
159	MED10	9606.ENSP00000255764	MED10	Mediator of RNA polymerase II transcription subunit 10; Component of the Mediator complex, a coactivator involved in the regulated transcription of nearly all RNA polymerase II-dependent genes. Mediator functions as a bridge to convey information from gene-specific regulatory proteins to the basal RNA polymerase II transcription machinery. Mediator is recruited to promoters by direct interactions with regulatory proteins and serves as a scaffold for the assembly of a functional preinitiation complex with RNA polymerase II and the general transcription factors
160	MEIS1	9606.ENSP00000272369	MEIS1	Homeobox protein Meis1; Acts as a transcriptional regulator of PAX6. Acts as a transcriptional activator of PF4 in complex with PBX1 or PBX2. Required for hematopoiesis, megakaryocyte lineage development and vascular patterning. May function as a cofactor for HOXA7 and HOXA9 in the induction of myeloid leukemias; TALE class homeoboxes and pseudogenes
161	MFAP3	9606.ENSP00000322956	MFAP3	Microfibril-associated glycoprotein 3; Component of the elastin-associated microfibrils; I-set domain containing
162	MICAL3	9606.ENSP00000416015	MICAL3	[F-actin]-monooxygenase MICAL3; Monooxygenase that promotes depolymerization of F-actin by mediating oxidation of specific methionine residues on actin to form methionine-sulfoxide, resulting in actin filament disassembly and preventing repolymerization. In the absence of actin, it also functions as a NADPH oxidase producing H(2)O(2). Seems to act as Rab effector protein and plays a role in vesicle trafficking. Involved in exocytic vesicles tethering and fusion: the monooxygenase activity is required for this process and implicates RAB8A associated with exocytotic vesicles. Required fo [...]
163	MKI67	9606.ENSP00000357643	MKI67	Proliferation marker protein Ki-67; Required to maintain individual mitotic chromosomes dispersed in the cytoplasm following nuclear envelope disassembly. Associates with the surface of the mitotic chromosome, the perichromosomal layer, and covers a substantial fraction of the chromosome surface. Prevents chromosomes from collapsing into a single chromatin mass by forming a steric and electrostatic charge barrier: the protein has a high net electrical charge and acts as a surfactant, dispersing chromosomes and enabling independent chromosome motility. Binds DNA, with a preference for s [...]
164	MROH1	9606.ENSP00000435565	MROH1	Maestro heat like repeat family member 1
165	MSN	9606.ENSP00000353408	MSN	Moesin; Probably involved in connections of major cytoskeletal structures to the plasma membrane. May inhibit herpes simplex virus 1 infection at an early stage. Plays a role in regulating the proliferation, migration, and adhesion of human lymphoid cells and participates in immunologic synapse formation; FERM domain containing
166	MTRNR2L1	9606.ENSP00000439228	MTRNR2L1	Humanin-like 1; Plays a role as a neuroprotective and antiapoptotic factor; Belongs to the humanin family
167	MYL3	9606.ENSP00000379210	MYL3	Myosin light chain 3; Regulatory light chain of myosin. Does not bind calcium; EF-hand domain containing
168	MYLK	9606.ENSP00000353452	MYLK	Myosin light chain kinase, smooth muscle; Calcium/calmodulin-dependent myosin light chain kinase implicated in smooth muscle contraction via phosphorylation of myosin light chains (MLC). Also regulates actin-myosin interaction through a non-kinase activity. Phosphorylates PTK2B/PYK2 and myosin light-chains. Involved in the inflammatory response (e.g. apoptosis, vascular permeability, leukocyte diapedesis), cell motility and morphology, airway hyperreactivity and other activities relevant to asthma. Required for tonic airway smooth muscle contraction that is necessary for physiological [...]
169	MYT1	9606.ENSP00000327465	MYT1	Myelin transcription factor 1; Binds to the promoter region of genes encoding proteolipid proteins of the central nervous system. May play a role in the development of neurons and oligodendroglia in the CNS. May regulate a critical transition point in oligodendrocyte lineage development by modulating oligodendrocyte progenitor proliferation relative to terminal differentiation and up- regulation of myelin gene transcription; Belongs to the MYT1 family
170	NAGK	9606.ENSP00000477639	NAGK	N-acetyl-D-glucosamine kinase; Converts endogenous N-acetylglucosamine (GlcNAc), a major component of complex carbohydrates, from lysosomal degradation or nutritional sources into GlcNAc 6-phosphate. Involved in the N-glycolylneuraminic acid (Neu5Gc) degradation pathway: although human is not able to catalyze formation of Neu5Gc due to the inactive CMAHP enzyme, Neu5Gc is present in food and must be degraded. Also has ManNAc kinase activity
171	NCKAP5	9606.ENSP00000387128	NCKAP5	NCK associated protein 5
172	NEDD4L	9606.ENSP00000383199	NEDD4L	E3 ubiquitin-protein ligase NEDD4-like; E3 ubiquitin-protein ligase which accepts ubiquitin from an E2 ubiquitin-conjugating enzyme in the form of a thioester and then directly transfers the ubiquitin to targeted substrates. Inhibits TGF-beta signaling by triggering SMAD2 and TGFBR1 ubiquitination and proteasome-dependent degradation. Promotes ubiquitination and internalization of various plasma membrane channels such as ENaC, Nav1.2, Nav1.3, Nav1.5, Nav1.7, Nav1.8, Kv1.3, KCNH2, EAAT1 or CLC5. Promotes ubiquitination and degradation of SGK1 and TNK2. Ubiquitinates BRAT1 and this ubiqu [...]
173	NME5	9606.ENSP00000265191	NME5	Nucleoside diphosphate kinase homolog 5; Does not seem to have NDK kinase activity. Confers protection from cell death by Bax and alters the cellular levels of several antioxidant enzymes including Gpx5. May play a role in spermiogenesis by increasing the ability of late-stage spermatids to eliminate reactive oxygen species (By similarity); NME/NM23 family
174	NOL4	9606.ENSP00000261592	NOL4	Nucleolar protein 4
175	NPAS3	9606.ENSP00000348460	NPAS3	Neuronal PAS domain-containing protein 3; May play a broad role in neurogenesis. May control regulatory pathways relevant to schizophrenia and to psychotic illness (By similarity); Basic helix-loop-helix proteins
176	NPTX2	9606.ENSP00000265634	NPTX2	Neuronal pentraxin-2; Likely to play role in the modification of cellular properties that underlie long-term plasticity. Binds to agar matrix in a calcium-dependent manner (By similarity)
177	NR4A3	9606.ENSP00000482027	NR4A3	Nuclear receptor subfamily 4 group A member 3; Transcriptional activator that binds to regulatory elements in promoter regions in a cell- and response element (target)-specific manner. Induces gene expression by binding as monomers to the NR4A1 response element (NBRE) 5'-AAAAGGTCA-3' site and as homodimers to the Nur response element (NurRE) site in the promoter of their regulated target genes (By similarity). Plays a role in the regulation of proliferation, survival and differentiation of many different cell types and also in metabolism and inflammation. Mediates proliferation of vasc [...]
178	NRXN3	9606.ENSP00000451648	NRXN3	Neurexin-3; Neuronal cell surface protein that may be involved in cell recognition and cell adhesion. May mediate intracellular signaling; Neurexins
179	NSMCE1	9606.ENSP00000355077	NSMCE1	Non-structural maintenance of chromosomes element 1 homolog; Component of the SMC5-SMC6 complex, a complex involved in DNA double-strand breaks by homologous recombination. The complex may promote sister chromatid homologous recombination by recruiting the SMC1-SMC3 cohesin complex to double-strand breaks. The complex is required for telomere maintenance via recombination in ALT (alternative lengthening of telomeres) cell lines and mediates sumoylation of shelterin complex (telosome) components which is proposed to lead to shelterin complex disassembly in ALT-associated PML bodies (AP [...]
180	NUDT6	9606.ENSP00000306070	NUDT6	Nucleoside diphosphate-linked moiety X motif 6; May contribute to the regulation of cell proliferation; Belongs to the Nudix hydrolase family
181	NUP50	9606.ENSP00000345895	NUP50	Nuclear pore complex protein Nup50; Component of the nuclear pore complex that has a direct role in nuclear protein import. Actively displaces NLSs from importin-alpha, and facilitates disassembly of the importin-alpha:beta-cargo complex and importin recycling. Interacts with regulatory proteins of cell cycle progression including CDKN1B (By similarity). This interaction is required for correct intracellular transport and degradation of CDKN1B (By similarity); Nucleoporins
182	NYAP2	9606.ENSP00000272907	NYAP2	Neuronal tyrosine-phosphorylated phosphoinositide-3-kinase adapter 2; Activates PI3K and concomitantly recruits the WAVE1 complex to the close vicinity of PI3K and regulates neuronal morphogenesis
183	OGDHL	9606.ENSP00000363216	OGDHL	2-oxoglutarate dehydrogenase-like, mitochondrial; Oxoglutarate dehydrogenase like; Belongs to the alpha-ketoglutarate dehydrogenase family
184	OGFRL1	9606.ENSP00000359464	OGFRL1	Opioid growth factor receptor like 1
185	OLFM3	9606.ENSP00000345192	OLFM3	Noelin-3; Olfactomedin 3
186	OR1E1	9606.ENSP00000313384	OR1E1	Olfactory receptor 1E1; Odorant receptor; Olfactory receptors, family 1
187	OTOS	9606.ENSP00000375849	OTOS	Otospiralin; May be essential for the survival of the neurosensory epithelium of the inner ear
188	OTP	9606.ENSP00000302814	OTP	Homeobox protein orthopedia; Probably involved in the differentiation of hypothalamic neuroendocrine cells; Belongs to the paired homeobox family. Bicoid subfamily
189	OTUD7A	9606.ENSP00000305926	OTUD7A	OTU domain-containing protein 7A; Has deubiquitinating activity towards 'Lys-11'-linked polyubiquitin chains; Belongs to the peptidase C64 family
190	P4HB	9606.ENSP00000327801	P4HB	Protein disulfide-isomerase; This multifunctional protein catalyzes the formation, breakage and rearrangement of disulfide bonds. At the cell surface, seems to act as a reductase that cleaves disulfide bonds of proteins attached to the cell. May therefore cause structural modifications of exofacial proteins. Inside the cell, seems to form/rearrange disulfide bonds of nascent proteins. At high concentrations, functions as a chaperone that inhibits aggregation of misfolded proteins. At low concentrations, facilitates aggregation (anti-chaperone activity). May be involved with other chape [...]
192	PABPN1L	9606.ENSP00000408598	PABPN1L	Embryonic polyadenylate-binding protein 2; Binds the poly(A) tail of mRNA; RNA binding motif containing
193	PAQR8	9606.ENSP00000406197	PAQR8	Membrane progestin receptor beta; Plasma membrane progesterone (P4) receptor coupled to G proteins. Seems to act through a G(i) mediated pathway. May be involved in oocyte maturation (By similarity). Also binds dehydroepiandrosterone (DHEA), pregnanolone, pregnenolone and allopregnanolone; Belongs to the ADIPOR family
194	PARP6	9606.ENSP00000456348	PARP6	poly(ADP-ribose) polymerase family member 6
195	PARVA	9606.ENSP00000334008	PARVA	Alpha-parvin; Plays a role in sarcomere organization and in smooth muscle cell contraction. Required for normal development of the embryonic cardiovascular system, and for normal septation of the heart outflow tract. Plays a role in sprouting angiogenesis and is required for normal adhesion of

#	queryItem	stringId	preferredName	annotation
				vascular smooth muscle cells to endothelial cells during blood vessel development (By similarity). Plays a role in the reorganization of the actin cytoskeleton, formation of lamellipodia and ciliogenesis. Plays a role in the establishement of cell polarity, cell adhesion, cell spreading, and dire [...]
196	PCDH7	9606.ENSP00000441802	PCDH7	Protocadherin-7; Protein phosphatase 1 regulatory subunits; Non-clustered protocadherins
197	PDE2A	9606.ENSP00000334910	PDE2A	cGMP-dependent 3',5'-cyclic phosphodiesterase; Cyclic nucleotide phosphodiesterase with a dual- specificity for the second messengers cAMP and cGMP, which are key regulators of many important physiological processes. Plays an important role in growth and invasion of malignant melanoma cells (e.g. pseudomyxoma peritonei (PMP) cell line)
198	PHACTR2	9606.ENSP00000417038	PHACTR2	Phosphatase and actin regulator 2
199	PHKA1	9606.ENSP00000362643	PHKA1	Phosphorylase b kinase regulatory subunit alpha, skeletal muscle isoform; Phosphorylase b kinase catalyzes the phosphorylation of serine in certain substrates, including troponin I. The alpha chain may bind calmodulin
200	PI15	9606.ENSP00000260113	PI15	Peptidase inhibitor 15; Serine protease inhibitor which displays weak inhibitory activity against trypsin. May play a role in facial patterning during embryonic development (By similarity); Belongs to the CRISP family
201	PIMREG	9606.ENSP00000250056	FAM64A	Protein PIMREG; During mitosis, may play a role in the control of metaphase-to-anaphase transition
202	PLD5	9606.ENSP00000440896	PLD5	Inactive phospholipase D5; Phospholipase D family member 5; Belongs to the phospholipase D family
203	PNOC	9606.ENSP00000301908	PNOC	Prepronociceptin; Nociceptin: Ligand of the opioid receptor-like receptor OPRL1. It may act as a transmitter in the brain by modulating nociceptive and locomotor behavior. May be involved in neuronal differentiation and development; Belongs to the opioid neuropeptide precursor family
204	PNPLA7	9606.ENSP00000384610	PNPLA7	Patatin-like phospholipase domain-containing protein 7; Serine hydrolase, whose specific chemical modification by certain organophosphorus (OP) compounds leads to distal axonopathy; Patatin like phospholipase domain containing
205	PPARA	9606.ENSP00000385523	PPARA	Peroxisome proliferator-activated receptor alpha; Ligand-activated transcription factor. Key regulator of lipid metabolism. Activated by the endogenous ligand 1-palmitoyl- 2-oleoyl-sn-glycerol-3-phosphocholine (16:0/18:1-GPC). Activated by oleylethanolamide, a naturally occurring lipid that regulates satiety. Receptor for peroxisome proliferators such as hypolipidemic drugs and fatty acids. Regulates the peroxisomal beta-oxidation pathway of fatty acids. Functions as transcription activator for the ACOX1 and P450 genes. Transactivation activity requires heterodimerization with RXRA and [...]
206	PPEF2	9606.ENSP00000286719	PPEF2	Serine/threonine-protein phosphatase with EF-hands 2; May play a role in phototransduction. May dephosphorylate photoactivated rhodopsin. May function as a calcium sensing regulator of ionic currents, energy production or synaptic transmission; Belongs to the PPP phosphatase family
207	PPP2R2A	9606.ENSP00000325074	PPP2R2A	Serine/threonine-protein phosphatase 2A 55 kDa regulatory subunit B alpha isoform; The B regulatory subunit might modulate substrate selectivity and catalytic activity, and also might direct the localization of the catalytic enzyme to a particular subcellular compartment; Protein phosphatase 2 regulatory subunits
208	PPP4R4	9606.ENSP00000305924	PPP4R4	Serine/threonine-protein phosphatase 4 regulatory subunit 4; Putative regulatory subunit of serine/threonine-protein phosphatase 4; Armadillo-like helical domain containing
209	PRDM1	9606.ENSP00000358092	PRDM1	PR domain zinc finger protein 1; Transcription factor that mediates a transcriptional program in various innate and adaptive immune tissue-resident lymphocyte T cell types such as tissue-resident memory T (Trm), natural killer (trNK) and natural killer T (NKT) cells and negatively regulates gene expression of proteins that promote the egress of tissue-resident T-cell populations from non-lymphoid organs. Plays a role in the development, retention and long-term establishment of adaptive and innate tissue-resident lymphocyte T cell types in non-lymphoid organs, such as the skin and gut, [...]
210	PRDM5	9606.ENSP00000264808	PRDM5	PR domain zinc finger protein 5; Sequence-specific DNA-binding transcription factor. Represses transcription at least in part by recruitment of the histone methyltransferase EHMT2/G9A and histone deacetylases such as HDAC1. Regulates hematopoiesis-associated protein-coding and microRNA (miRNA) genes. May regulate the expression of proteins involved in extracellular matrix development and maintenance, including fibrillar collagens, such as COL4A1 and COL11A1, connective tissue components, such as HAPLN1, and molecules regulating cell migration and adhesion, including EDIL3 and TGFB2. Ma [...]
211	PRELID3A	9606.ENSP00000404700	SLMO1	PRELI domain containing protein 3A; In vitro, the TRIAP1:PRELID3A complex mediates the transfer of phosphatidic acid (PA) between liposomes and probably functions as a PA transporter across the mitochondrion intermembrane space. Phosphatidic acid import is required for cardiolipin (CL) synthesis in the mitochondrial inner membrane; Belongs to the slowmo family
212	PRLHR	9606.ENSP00000239032	PRLHR	Prolactin-releasing peptide receptor; Receptor for prolactin-releasing peptide (PrRP). Implicated in lactation, regulation of food intake and pain-signal processing; Belongs to the G-protein coupled receptor 1 family
213	PRSS16	9606.ENSP00000230582	PRSS16	Thymus-specific serine protease; Protease that may play a role in T-cell development; Belongs to the peptidase S28 family
214	PTDSS1	9606.ENSP00000430548	PTDSS1	Phosphatidylserine synthase 1; Catalyzes a base-exchange reaction in which the polar head group of phosphatidylethanolamine (PE) or phosphatidylcholine (PC) is replaced by L-serine. In membranes, PTDSS1 catalyzes mainly the conversion of phosphatidylcholine. Also converts, in vitro and to a lesser extent, phosphatidylethanolamine
215	PTGR2	9606.ENSP00000452280	PTGR2	Prostaglandin reductase 2; Functions as 15-oxo-prostaglandin 13-reductase and acts on 15-keto-PGE1, 15-keto-PGE2, 15-keto-PGE1-alpha and 15-keto-PGE2-alpha with highest activity towards 15-keto-PGE2. Overexpression represses transcriptional activity of PPARG and inhibits adipocyte differentiation (By similarity)
216	RAC2	9606.ENSP00000249071	RAC2	Ras-related C3 botulinum toxin substrate 2; Plasma membrane-associated small GTPase which cycles between an active GTP-bound and inactive GDP-bound state. In active state binds to a variety of effector proteins to regulate cellular responses, such as secretory processes, phagocytose of apoptotic cells and epithelial cell polarization. Augments the production of reactive oxygen species (ROS) by NADPH oxidase
217	RAET1L	9606.ENSP00000356310	RAET1L	UL16-binding protein 6; Binds and activates the KLRK1/NKG2D receptor, mediating natural killer cell cytotoxicity
218	RALGAPA1	9606.ENSP00000302647	RALGAPA1	Ral GTPase-activating protein subunit alpha-1; Catalytic subunit of the heterodimeric RalGAP1 complex which acts as a GTPase activator for the Ras-like small GTPases RALA and RALB
219	RANBP10	9606.ENSP00000316589	RANBP10	Ran-binding protein 10; Acts as a guanine nucleotide exchange factor (GEF) for RAN GTPase (By similarity). May play an essential role in hemostasis and in maintaining microtubule dynamics with respect to both platelet shape and function (By similarity). May act as an adapter protein to couple membrane receptors to intracellular signaling pathways. Enhances dihydrotestosterone-induced transactivation activity of AR, as well as dexamethasone-induced transactivation activity of NR3C1, but does not affect estrogen- induced transactivation. In contrast to RANBP9, does not interact with Sos [...]
220	RAP2A	9606.ENSP00000245304	RAP2A	Ras-related protein Rap-2a; Small GTP-binding protein which cycles between a GDP- bound inactive and a GTP-bound active form. In its active form interacts with and regulates several effectors including MAP4K4, MINK1 and TNIK. Part of a signaling complex composed of NEDD4, RAP2A and TNIK which regulates neuronal dendrite extension and arborization during development. More generally, it is part of several signaling cascades and may regulate cytoskeletal rearrangements, cell migration, cell adhesion and cell spreading; RAS type GTPase family
221	RBM10	9606.ENSP00000328848	RBM10	RNA-binding protein 10; May be involved in post-transcriptional processing, most probably in mRNA splicing. Binds to RNA homopolymers, with a preference for poly(G) and poly(U) and little for poly(A) (By similarity). May bind to specific miRNA hairpins; G-patch domain containing
222	RBM46	9606.ENSP00000281722	RBM46	Probable RNA-binding protein 46; RNA binding motif containing
223	RELN	9606.ENSP00000392423	RELN	Reelin; Extracellular matrix serine protease that plays a role in layering of neurons in the cerebral cortex and cerebellum. Regulates microtubule function in neurons and neuronal migration. Affects migration of sympathetic preganglionic neurons in the spinal cord, where it seems to act as a barrier to neuronal migration. Enzymatic activity is important for the modulation of cell adhesion. Binding to the extracellular domains of lipoprotein receptors VLDLR and LRP8/APOER2 induces tyrosine phosphorylation of DAB1 and modulation of TAU phosphorylation (By similarity); Belongs to the reel [...]
224	RERE	9606.ENSP00000338629	RERE	Arginine-glutamic acid dipeptide repeats protein; Plays a role as a transcriptional repressor during development. May play a role in the control of cell survival. Overexpression of RERE recruits BAX to the nucleus particularly to POD and triggers caspase-3 activation, leading to cell death; GATA zinc finger domain containing
225	RGS9	9606.ENSP00000262406	RGS9	Regulator of G-protein signaling 9; Inhibits signal transduction by increasing the GTPase activity of G protein alpha subunits thereby driving them into their inactive GDP-bound form. Binds to G(t)-alpha. Involved in phototransduction; key element in the recovery phase of visual transduction (By similarity); Regulators of G-protein signaling
226	RHCG	9606.ENSP00000268122	RHCG	Ammonium transporter Rh type C; Functions as an electroneutral and bidirectional ammonium transporter. May regulate transepithelial ammonia secretion; Solute carriers
227	RIOK1	9606.ENSP00000369162	RIOK1	Serine/threonine-protein kinase RIO1; Involved in the final steps of cytoplasmic maturation of the 40S ribosomal subunit. Involved in processing of 18S-E pre- rRNA to the mature 18S rRNA. Required for the recycling of NOB1 and PNO1 from the late 40S precursor. The association with the very late 40S subunit intermediate may involve a translation-like checkpoint point cycle preceeding the binding to the 60S ribosomal subunit (By similarity). Despite the protein kinase domain is proposed to act predominantly as an ATPase (By similarity). The catalytic activity regulates its dynamic associ [...]
228	RNF144B	9606.ENSP00000259939	RNF144B	E3 ubiquitin-protein ligase RNF144B; E3 ubiquitin-protein ligase which accepts ubiquitin from E2 ubiquitin-conjugating enzymes UBE2L3 and UBE2L6 in the form of a thioester and then directly transfers the ubiquitin to targeted substrates such as LCMT2, thereby promoting their degradation. Induces apoptosis via a p53/TP53-dependent but caspase-independent mechanism. However, its overexpression also produces a decrease of the ubiquitin-dependent stability of BAX, a pro-apoptotic protein, ultimately leading to protection of cell death; But, it is not an anti-apoptotic protein per se
229	RPH3AL	9606.ENSP00000328977	RPH3AL	Rab effector Noc2; Rab GTPase effector involved in the late steps of regulated exocytosis, both in endocrine and exocrine cells (By similarity). Acts as a potential RAB3B effector protein in epithelial cells
230	RPS6KA2	9606.ENSP00000427015	RPS6KA2	Ribosomal protein S6 kinase alpha-2; Serine/threonine-protein kinase that acts downstream of ERK (MAPK1/ERK2 and MAPK3/ERK1) signaling and mediates mitogenic and stress-induced activation of transcription factors, regulates translation, and mediates cellular proliferation, survival, and differentiation. May function as tumor suppressor in epithelial ovarian cancer cells; Mitogen-activated protein kinase-activated protein kinases
231	RRAGB	9606.ENSP00000262850	RRAGB	Ras-related GTP-binding protein B; Guanine nucleotide-binding protein that plays a crucial role in the cellular response to amino acid availability through regulation of the mTORC1 signaling cascade. Forms heterodimeric Rag complexes with RRAGC or RRAGD and cycles between an inactive GDP-bound and an active GTP-bound form. In its active form participates in the relocalization of mTORC1 to the lysosomes and its subsequent activation by the GTPase RHEB. Involved in the RCC1/Ran-GTPase pathway; Belongs to the GTR/RAG GTP-binding protein family
232	RTL1	9606.ENSP00000435342	RTL1	Retrotransposon-like protein 1; Plays an essential role in capillaries endothelial cells for the maintenance of feto-maternal interface and for development of the placenta; Retrotransposon Gag like
233	RTTN	9606.ENSP00000255674	RTTN	Rotatin; Involved in the genetic cascade that governs left-right specification. Plays a role in the maintenance of a normal ciliary structure. Required for correct asymmetric expression of NODAL, LEFTY and PITX2; Armadillo-like helical domain containing
234	S100A2	9606.ENSP00000357697	S100A2	Protein S100-A2; May function as calcium sensor and modulator, contributing to cellular calcium signaling. May function by interacting with other proteins, such as TPR-containing proteins, and indirectly play a role in many physiological processes. May also play a role in suppressing tumor cell growth; EF-hand domain containing
235	SEL1L2	9606.ENSP00000367312	SEL1L2	Protein sel-1 homolog 2; SEL1L2 ERAD E3 ligase adaptor subunit; Belongs to the sel-1 family
236	SEPP1	9606.ENSP00000420939	SEPP1	Selenoprotein P; Might be responsible for some of the extracellular antioxidant defense properties of selenium or might be involved in the transport of selenium. May supply selenium to tissues such as brain and testis
237	SFSWAP	9606.ENSP00000437738	SFSWAP	Splicing factor, suppressor of white-apricot homolog; Plays a role as an alternative splicing regulator. Regulate its own expression at the level of RNA processing. Also regulates the splicing of fibronectin and CD45 genes. May act, at least in part, by interaction with other R/S-containing splicing factors. Represses the splicing of MAPT/Tau exon 10
238	SGMS2	9606.ENSP00000378176	SGMS2	Phosphatidylcholine:ceramide cholinephosphotransferase 2; Sphingomyelin synthases synthesize the sphingolipid, sphingomyelin, through transfer of the phosphatidyl head group, phosphatidylcholine, on to the primary hydroxyl of ceramide. The reaction is bidirectional depending on the respective levels of the sphingolipid and ceramide. Plasma membrane SMS2 can also convert phosphatidylethanolamine (PE) to ceramide phosphatidylethanolamine (CPE). Major form in liver. Required for cell growth in certain cell types. Regulator of cell surface levels of ceramide, an important mediator of signa [...]
239	SH3BP2	9606.ENSP00000422168	SH3BP2	SH3 domain-binding protein 2; Binds differentially to the SH3 domains of certain proteins of signal transduction pathways. Binds to phosphatidylinositols; linking the hemopoietic tyrosine kinase fes to the cytoplasmic membrane in a phosphorylation dependent mechanism; Pleckstrin homology domain containing
240	SHANK1	9606.ENSP00000293441	SHANK1	SH3 and multiple ankyrin repeat domains protein 1; Seems to be an adapter protein in the postsynaptic density (PSD) of excitatory synapses that interconnects receptors of the postsynaptic membrane including NMDA-type and metabotropic glutamate receptors via complexes with GKAP/PSD-95 and Homer, respectively, and the actin-based cytoskeleton. Plays a role in the structural and functional organization of the dendritic spine and synaptic junction; Ankyrin repeat domain containing
241	SHISA6	9606.ENSP00000390084	SHISA6	Protein shisa-6 homolog; Shisa family member 6
242	SLC22A18	9606.ENSP00000369948	SLC22A18	Solute carrier family 22 member 18; May act as a transporter of organic cations based on a proton efflux antiport mechanism. May play a role in the transport of chloroquine and quinidine-related compounds in kidney; Solute carriers
243	SLC23A2	9606.ENSP00000368637	SLC23A2	Solute carrier family 23 member 2; Sodium/ascorbate cotransporter. Mediates electrogenic uptake of vitamin C, with a stoichiometry of 2 Na(+) for each ascorbate; Solute carriers
244	SLC35F3	9606.ENSP00000355577	SLC35F3	Putative thiamine transporter SLC35F3; May be a thiamine transporter; Belongs to the SLC35F solute transporter family

#	queryItem	stringId	preferredName	annotation
245	SLC46A1	9606.ENSP00000480703	SLC46A1	Proton-coupled folate transporter; Has been shown to act both as an intestinal proton- coupled high-affinity folate transporter and as an intestinal heme transporter which mediates heme uptake from the gut lumen into duodenal epithelial cells. The iron is then released from heme and may be transported into the bloodstream. Dietary heme iron is an important nutritional source of iron. Shows a higher affinity for folate than heme; Solute carriers
246	SLC6A3	9606.ENSP00000270349	SLC6A3	Sodium-dependent dopamine transporter; Amine transporter. Terminates the action of dopamine by its high affinity sodium-dependent reuptake into presynaptic terminals; Solute carriers
247	SLC9C1	9606.ENSP00000306627	SLC9C1	Sodium/hydrogen exchanger 10; Sperm-specific sodium/hydrogen exchanger involved in intracellular pH regulation of spermatozoa. Required for sperm motility and fertility. Involved in sperm cell hyperactivation, a step needed for sperm motility which is essential late in the preparation of sperm for fertilization. Required for the expression and bicarbonate regulation of the soluble adenylyl cyclase (sAC) (By similarity); Solute carriers
248	SLCO4C1	9606.ENSP00000309741	SLCO4C1	Solute carrier organic anion transporter family member 4C1; Organic anion transporter, capable of transporting pharmacological substances such as digoxin, ouabain, thyroxine, methotrexate and cAMP. May participate in the regulation of membrane transport of ouabain. Involved in the uptake of the dipeptidyl peptidase-4 inhibitor sitagliptin and hence may play a role in its transport into and out of renal proximal tubule cells. May be involved in the first step of the transport pathway of digoxin and various compounds into the urine in the kidney. May be involved in sperm maturation by en [...]
249	SLIT1	9606.ENSP00000266058	SLIT1	Slit homolog 1 protein; Thought to act as molecular guidance cue in cellular migration, and function appears to be mediated by interaction with roundabout homolog receptors. During neural development involved in axonal navigation at the ventral midline of the neural tube and projection of axons to different regions (By similarity). SLIT1 and SLIT2 together seem to be essential for midline guidance in the forebrain by acting as repulsive signal preventing inappropriate midline crossing by axons projecting from the olfactory bulb
250	SMAP2	9606.ENSP00000361803	SMAP2	Stromal membrane-associated protein 2; GTPase activating protein that acts on ARF1. Can also activate ARF6 (in vitro). May play a role in clathrin-dependent retrograde transport from early endosomes to the trans-Golgi network (By similarity); ArfGAPs
251	SMARCA1	9606.ENSP00000360162	SMARCA1	Probable global transcription activator SNF2L1; Energy-transducing component of NURF (nucleosome- remodeling factor) and CERF (CECR2-containing-remodeling factor) complexes. Both complexes facilitate the perturbation of chromatin structure in an ATP-dependent manner. Potentiates neurite outgrowth. May be involved in brain development by regulating En-1 and En-2 expression. May be involved in the development of luteal cells; Myb/SANT domain containing
252	SNX16	9606.ENSP00000379621	SNX16	Sorting nexin-16; May be involved in several stages of intracellular trafficking. Plays a role in protein transport from early to late endosomes. Plays a role in protein transport to the lysosome. Promotes degradation of EGFR after EGF signaling. Plays a role in intracellular transport of vesicular stomatitis virus nucleocapsids from the endosome to the cytoplasm; Belongs to the sorting nexin family
253	SPIDR	9606.ENSP00000297423	SPIDR	DNA repair-scaffolding protein; Plays a role in DNA double-strand break (DBS) repair via homologous recombination (HR). Serves as a scaffolding protein that helps to promote the recruitment of DNA-processing enzymes like the helicase BLM and recombinase RAD51 to site of DNA damage, and hence contributes to maintain genomic integrity
254	SPTLC2	9606.ENSP00000216484	SPTLC2	Serine palmitoyltransferase 2; Serine palmitoyltransferase (SPT). The heterodimer formed with LCB1/SPTLC1 constitutes the catalytic core. The composition of the serine palmitoyltransferase (SPT) complex determines the substrate preference. The SPTLC1-SPTLC2-SPTSSA complex shows a strong preference for C16-CoA substrate, while the SPTLC1-SPTLC2-SPTSSB complex displays a preference for C18-CoA substrate; Belongs to the class-II pyridoxal-phosphate-dependent aminotransferase family
255	SRRM4	9606.ENSP00000267260	SRRM4	Serine/arginine repetitive matrix protein 4; Splicing factor specifically required for neural cell differentiation. Acts in conjuction with nPTB/PTBP2 by binding directly to its regulated target transcripts and promotes neural- specific exon inclusion in many genes that function in neural cell differentiation. Required to promote the inclusion of neural- specific exon 10 in nPTB/PTBP2, leading to increased expression of neural-specific nPTB/PTBP2. Also promotes the inclusion of exon 16 in DAAM1 in neuron extracts (By similarity)
256	STAC	9606.ENSP00000273183	STAC	SH3 and cysteine-rich domain-containing protein; Probably involved in a neuron-specific signal transduction
257	STX3	9606.ENSP00000338562	STX3	Syntaxin-3; Potentially involved in docking of synaptic vesicles at presynaptic active zones; Belongs to the syntaxin family
258	TANGO6	9606.ENSP00000261778	TANGO6	Transport and golgi organization 6 homolog; Armadillo-like helical domain containing
259	TBPL2	9606.ENSP00000247219	TBPL2	TATA box-binding protein-like protein 2; Transcription factor required in complex with TAF3 for the differentiation of myoblasts into myocytes. The complex replaces TFIID at specific promoters at an early stage in the differentiation process (By similarity); Belongs to the TBP family
260	TECTB	9606.ENSP00000358430	TECTB	Beta-tectorin; One of the major non-collagenous components of the tectorial membrane (By similarity). The tectorial membrane is an extracellular matrix of the inner ear that covers the neuroepithelium of the cochlea and contacts the stereocilia bundles of specialized sensory hair cells. Sound induces movement of these hair cells relative to the tectorial membrane, deflects the stereocilia and leads to fluctuations in hair-cell membrane potential, transducing sound into electrical signals
261	TEX29	9606.ENSP00000283547	TEX29	Testis-expressed protein 29; Testis expressed 29
262	THADA	9606.ENSP00000385995	THADA	Thyroid adenoma-associated protein; THADA, armadillo repeat containing; Belongs to the THADA family
263	TLE1	9606.ENSP00000365682	TLE1	Transducin-like enhancer protein 1; Transcriptional corepressor that binds to a number of transcription factors. Inhibits NF-kappa-B-regulated gene expression. Inhibits the transcriptional activation mediated by FOXA2, and by CTNNB1 and TCF family members in Wnt signaling. The effects of full-length TLE family members may be modulated by association with dominant-negative AES. Unusual function as coactivator for ESRRG; Belongs to the WD repeat Groucho/TLE family
264	TMED8	9606.ENSP00000216468	TMED8	Protein TMED8; Transmembrane p24 trafficking protein family member 8; GOLD domain containing
265	TMEM74	9606.ENSP00000297459	TMEM74	Transmembrane protein 74; Plays an essential role in autophagy. TMEM74-induced autophagy may involve PI3K signal transduction; Belongs to the TMEM74 family
266	TNFRSF19	9606.ENSP00000371693	TNFRSF19	Tumor necrosis factor receptor superfamily member 19; Can mediate activation of JNK and NF-kappa-B. May promote caspase-independent cell death; Tumor necrosis factor receptor superfamily
267	TNFSF13B	9606.ENSP00000365048	TNFSF13B	Tumor necrosis factor ligand superfamily member 13B; Cytokine that binds to TNFRSF13B/TACI and TNFRSF17/BCMA. TNFSF13/APRIL binds to the same 2 receptors. Together, they form a 2 ligands -2 receptors pathway involved in the stimulation of B- and T-cell function and the regulation of humoral immunity. A third B-cell specific BAFF-receptor (BAFFR/BR3) promotes the survival of mature B-cells and the B-cell response; CD molecules
268	TNS3	9606.ENSP00000312143	TNS3	Tensin-3; May play a role in actin remodeling. Involved in the dissociation of the integrin-tensin-actin complex. EGF activates TNS4 and down-regulates TNS3 which results in capping the tail of ITGB1. Seems to be involved in mammary cell migration. May be involved in cell migration and bone development (By similarity); C2 tensin-type domain containing
269	TOX	9606.ENSP00000354842	TOX	Thymocyte selection-associated high mobility group box protein TOX; May play a role in regulating T-cell development
270	TPH2	9606.ENSP00000329093	TPH2	Tryptophan hydroxylase 2
271	TRAPPC2L	9606.ENSP00000301021	TRAPPC2L	Trafficking protein particle complex subunit 2-like protein; May play a role in vesicular transport from endoplasmic reticulum to Golgi; Belongs to the TRAPP small subunits family. Sedlin subfamily
273	TRIM31	9606.ENSP00000365924	TRIM31	E3 ubiquitin-protein ligase TRIM31; Regulator of Src-induced anchorage independent cell growth (By similarity). May have E3 ubiquitin-protein ligase activity; Ring finger proteins
274	TRPA1	9606.ENSP00000262209	TRPA1	Transient receptor potential cation channel subfamily A member 1; Receptor-activated non-selective cation channel involved in detection of pain and possibly also in cold perception and inner ear function. Has a central role in the pain response to endogenous inflammatory mediators and to a diverse array of volatile irritants, such as mustard oil, cinnamaldehyde, garlic and acrolein, an irritant from tears gas and vehicule exhaust fumes. Is also activated by menthol (in vitro). Acts also as a ionotropic cannabinoid receptor by being activated by delta(9)-tetrahydrocannabinol (THC), the [...]
275	TRPV1	9606.ENSP00000459962	ENSG00000196689	Transient receptor potential cation channel, subfamily V, member 1; Ligand-activated non-selective calcium permeant cation channel involved in detection of noxious chemical and thermal stimuli. Seems to mediate proton influx and may be involved in intracellular acidosis in nociceptive neurons. Involved in mediation of inflammatory pain and hyperalgesia. Sensitized by a phosphatidylinositol second messenger system activated by receptor tyrosine kinases, which involves PKC isozymes and PCL. Activation by vanilloids, like capsaicin, and temperatures higher than 42 degrees Celsius, exhibit [...]
276	TSPAN9	9606.ENSP00000011898	TSPAN9	Tetraspanin-9; Tetraspanin 9; Belongs to the tetraspanin (TM4SF) family
277	TTLL11	9606.ENSP00000321346	TTLL11	Tubulin polyglutamylase TTLL11; Polyglutamase which preferentially modifies alpha- tubulin. Involved in the side-chain elongation step of the polyglutamylation reaction rather than in the initiation step (By similarity). Required for CCSAP localization to both spindle and cilia microtubules. Generates long side-chains (By similarity); Belongs to the tubulin--tyrosine ligase family
278	UCKL1	9606.ENSP00000346155	UCKL1	Uridine-cytidine kinase-like 1; May contribute to UTP accumulation needed for blast transformation and proliferation
279	UNCX	9606.ENSP00000314480	UNCX	Homeobox protein unc-4 homolog; Transcription factor involved in somitogenesis and neurogenesis. Required for the maintenance and differentiation of particular elements of the axial skeleton. May act upstream of PAX9. Plays a role in controlling the development of connections of hypothalamic neurons to pituitary elements, allowing central neurons to reach the peripheral blood circulation and to deliver hormones for control of peripheral functions (By similarity); PRD class homeoboxes and pseudogenes
280	UTP18	9606.ENSP00000225298	UTP18	U3 small nucleolar RNA-associated protein 18 homolog; Involved in nucleolar processing of pre-18S ribosomal RNA; UTPb subcomplex
281	VAC14	9606.ENSP00000261776	VAC14	Protein VAC14 homolog; The PI(3,5)P2 regulatory complex regulates both the synthesis and turnover of phosphatidylinositol 3,5-bisphosphate (PtdIns(3,5)P2). Acts as a positive activator of PIKfyve kinase activity. Also required to maintain normal levels of phosphatidylinositol 3-phosphate (PtdIns(3)P) and phosphatidylinositol 5-phosphate (PtdIns(5)P). Plays a role in the biogenesis of endosome carrier vesicles (ECV) / multivesicular bodies (MVB) transport intermediates from early endosomes; Belongs to the VAC14 family
282	VAX1	9606.ENSP00000358207	VAX1	Ventral anterior homeobox 1; Transcription factor that may function in dorsoventral specification of the forebrain. Required for axon guidance and major tract formation in the developing forebrain. May contribute to the differentiation of the neuroretina, pigmented epithelium and optic stalk (By similarity); Belongs to the EMX homeobox family
283	VCX3A	9606.ENSP00000370479	VCX3A	Variable charge X-linked protein 3; May mediate a process in spermatogenesis or may play a role in sex ratio distortion; Variable charge X/Y family
284	VKORC1L1	9606.ENSP00000403077	VKORC1L1	Vitamin K epoxide reductase complex subunit 1 like 1
285	VPS37B	9606.ENSP00000267202	VPS37B	Vacuolar protein sorting-associated protein 37B; Component of the ESCRT-I complex, a regulator of vesicular trafficking process. Required for the sorting of endocytic ubiquitinated cargos into multivesicular bodies. May be involved in cell growth and differentiation
286	WDR13	9606.ENSP00000365919	WDR13	WD repeat-containing protein 13; WD repeat domain containing
287	WNK1	9606.ENSP00000341292	WNK1	Serine/threonine-protein kinase WNK1; Serine/threonine kinase which plays an important role in the regulation of electrolyte homeostasis, cell signaling, survival, and proliferation. Acts as an activator and inhibitor of sodium-coupled chloride cotransporters and potassium-coupled chloride cotransporters respectively. Activates SCNN1A, SCNN1B, SCNN1D and SGK1. Controls sodium and chloride ion transport by inhibiting the activity of WNK4, by either phosphorylating the kinase or via an interaction between WNK4 and the autoinhibitory domain of WNK1. WNK4 regulates the activity of the thia [...]
288	WWC1	9606.ENSP00000427772	WWC1	Protein KIBRA; Probable regulator of the Hippo/SWH (Sav/Wts/Hpo) signaling pathway, a signaling pathway that plays a pivotal role in tumor suppression by restricting proliferation and promoting apoptosis. Along with NF2 can synergistically induce the phosphorylation of LATS1 and LATS2 and can probably function in the regulation of the Hippo/SWH (Sav/Wts/Hpo) signaling pathway. Acts as a transcriptional coactivator of ESR1 which plays an essential role in DYNLL1-mediated ESR1 transactivation. Regulates collagen-stimulated activation of the ERK/MAPK cascade. Modulates directional migrati [...]
289	YPEL4	9606.ENSP00000432648	YPEL4	Protein yippee-like 4; Yippee like family; Belongs to the yippee family
290	ZFHX3	9606.ENSP00000268489	ZFHX3	Zinc finger homeobox protein 3; Transcriptional regulator which can act as an activator or a repressor. Inhibits the enhancer element of the AFP gene by binding to its AT-rich core sequence. In concert with SMAD- dependent TGF-beta signaling can repress the transcription of AFP via its interaction with SMAD2/3. Regulates the circadian locomotor rhythms via transcriptional activation of neuropeptidergic genes which are essential for intercellular synchrony and rhythm amplitude in the suprachiasmatic nucleus (SCN) of the brain (By similarity). Regulator of myoblasts differentiation throu [...]
291	ZMYM1	9606.ENSP00000482579	ZMYM1	Zinc finger MYM-type containing 1
292	ZNF704	9606.ENSP00000331462	ZNF704	Zinc finger protein 704
294	ZP3	9606.ENSP00000378326	ZP3	Zona pellucida sperm-binding protein 3; The mammalian zona pellucida, which mediates species- specific sperm binding, induction of the acrosome reaction and prevents post-fertilization polyspermy, is composed of three to four glycoproteins, ZP1, ZP2, ZP3, and ZP4. ZP3 is essential for sperm binding and zona matrix formation; Belongs to the ZP domain family. ZPC subfamily

Feelings of Guilt (AAGS)

Gene list

ABCB11, ABCC2, ABL1, ACSBG2, ADCY7, ADRB3, AFTPH, AGPAT3, AJAP1, ANO1, ANO7, ANXA5, AOPEP, AP3B2, APBB2, ARF6, ARL15, ARMC8, ARNT2, ASB2, ATG2A, ATP11A, ATP6V0E1, AUTS2, AVEN, BCL2L11, BOLA3, C10orf99, C15orf39, C1QTNF9B, C2CD2, C2CD4B, C2orf83, C7orf50, CABLES1, CACNA2D2, CALM1, CASP3, CASR, CCDC113, CCDC93, CCR6, CD177, CDH4, CDH5, CDK6, CHD7, CHRDL2, CHRM3, CHRNA10, CHSY1, CLNK, COG4, COL23A1, CPEB1, CREB5, CRYBA4, CSDC2, CSMD1, CTNNA1, CUEDC1, DAP, DAPL1, DARS1, DAW1, DENND2A, DGCR8, DGKG, DIP2C, DLL4, DNAJC5, DNMT3A, DPYSL5, DRGX, DYSF, EBF3, EFCAB1, EIPR1, ELMO1, ENDOU, EPB41L2, EVI5, EXTL1, EZR, F7, FAM110A, FAM124B, FAM155A, FAM160B1, FAM168A, FAM174B, FAM228A, FAM3D, FBRSL1, FCN3, FILIP1L, FN1, FOXD1, FOXN2, FOXN3, FOXP2, FRG1, FRMD1, GADD45B, GAL3ST4, GALNT10, GATA3-AS1, GDF6, GINS4, GLRX3, GNA11, GNA12, GNAS, GOLGA8J, GPM6A, GPR88, GRB2, GSAP, GTF2F2, HCG22, HDAC9, HM13, HOXA11, HPSE2, HRH3, IDS, IFFO1, IFI30, INPP5D, IRX2, ISL2, ITCH, ITGA5, JARID2, JMJD7, KCNJ10, KIF13B, KLHL6, KLK7, KREMEN2, LAS1L, LDLRAD4, LINP1, LTB4R, LYZ, MAFB, MAGEA9B, MALAT1, MAP2K3, MAP2K4, MARCHF4, MB21D2, MBP, MET, MLN, MLXIP, MLXIPL, MSI2, MTHFD1L, MUC3A, MYC, N4BP1, NAP1L5, NAPB, NAV2, NBN, NFAM1, NFKBIL1, NFKBIZ, NINL, NKX2-6, NOL11, NPC2, NR2E1, NR3C1, NRG1, NSMCE1, NXPE3, OTX1, PCDH19, PDE10A, PDE7B, PHKB, PHLDA1, PHLDB2, PIK3R5, PIP5K1B, PITRM1, PITX1, PLCE1, PMP22, POLI, PPA2, PPARD, PPP4R4, PRDM10, PRDM16, PRELID3BP6, PRKCZ, PRPH, PRR5, PTGFRN, PTPN13, R3HDM1, RAB6C, RBFOX3, RBPMS2, RFFL, RHCG, RLIM, RNF111, ROPN1, RORA, RPL26P19, RSPO3, SCG2, SEMA3B, SH2B3, SHANK2, SHC3, SIGLEC15, SIX2, SKAP2, SLC16A9, SLC19A1, SLC1A7, SLC23A2, SLC2A4, SLC38A2, SLCO1A2, SMAD5, SMAD6, SMS, SORCS3, SOX2, SPRY1, SQSTM1, ST3GAL1, ST6GALNAC1, STAG3, STEAP2, STK10, STRADA, SULT2B1, SUSD1, SYNE2, SYT7, TAB3, TAFA5, TBL1XR1, TBX4, TDRKH, TENM4, TEX29, THBS4, TMED2, TMX1, TNS2, TP53TG3D, TRAM2, TRIB2, TRIM35, TSLP, TSN, TTPA, TWSG1, UCK2, UNCX, USP39, VRK1, VRK3, VWA3B, VWA5B2, YAP1, YTHDF1, ZBTB10, ZBTB33, ZC3HC1, ZFHX3, ZFYVE28, ZNF337, ZNF469, ZNF48, ZNF571, ZNF766, ZNF771, ZNF786, ZNF81, ZNRF2, ZSCAN10, ZSCAN26

Network

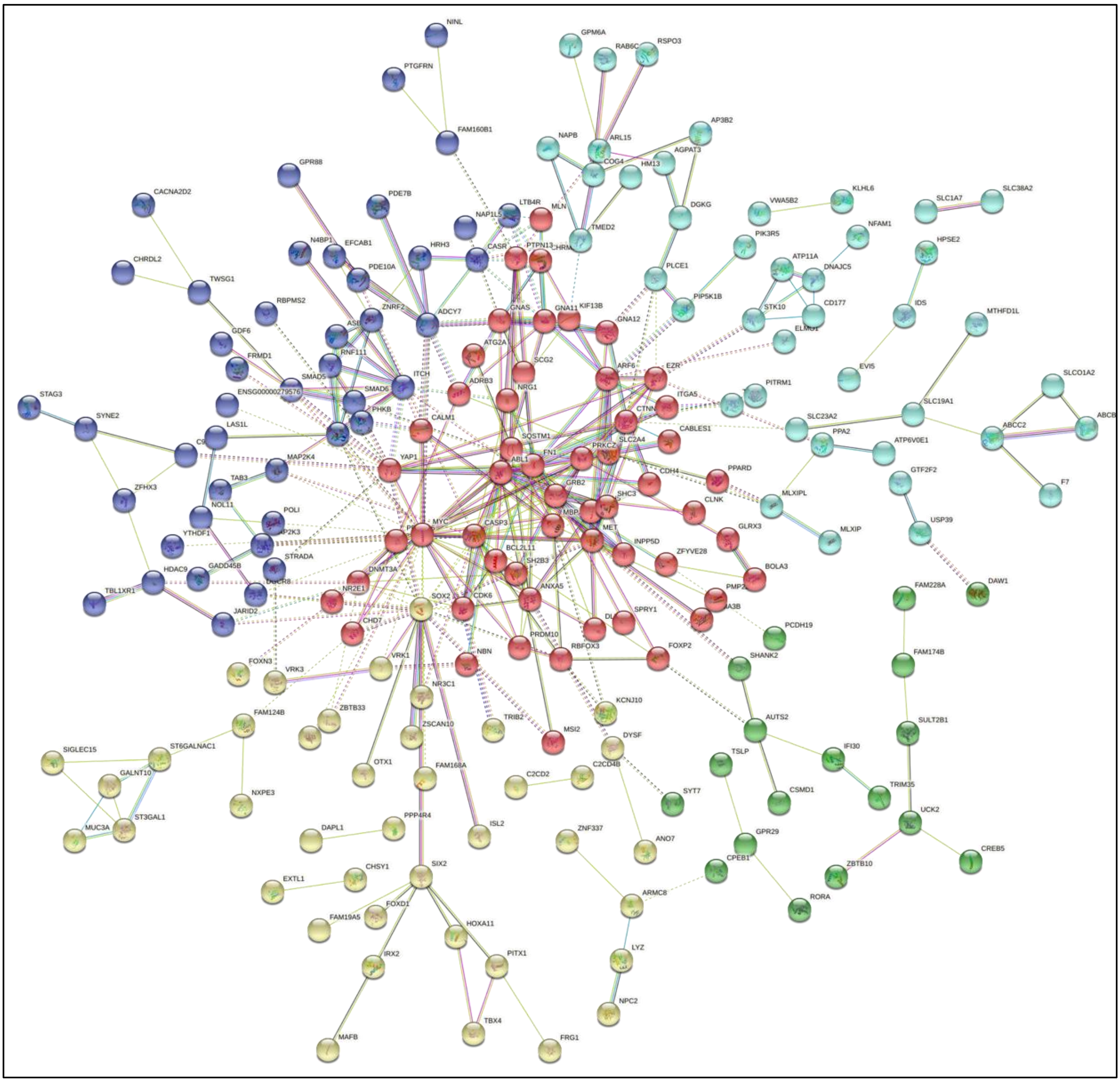

Social Acknowledgement (SAQ)

Enrichments found

Network Stats

number of nodes: 284
number of edges: 359
average node degree: 2.53
avg. local clustering coefficient: 0.394

expected number of edges: 268
PPI enrichment p-value: 6.76e-08
your network has significantly more interactions than expected (what does that mean?)

Functional enrichments in your network

explain columns

Enrichment Table Columns

Count In Network:
The first number indicates how many proteins in your network are annotated with a particular term. The second number indicates how many proteins in total (in your network and in the background) have this term assigned.

Strength:
Log10(observed / expected). This measure describes how large the enrichment effect is. It's the ratio between i) the number of proteins in your network that are annotated with a term and ii) the number of proteins that we expect to be annotated with this term in a random network of the same size.

False Discovery Rate:
This measure describes how significant the enrichment is. Shown are p-values corrected for multiple testing within each category using the Benjamini–Hochberg procedure.

Biological Process (Gene Ontology)

GO-term	description	count in network	strength	false discovery rate
GO:0045656	negative regulation of monocyte differentiation	3 of 7	1.47	0.0256
GO:0035019	somatic stem cell population maintenance	6 of 53	0.89	0.0197
GO:0061448	connective tissue development	12 of 194	0.63	0.0075
GO:0061138	morphogenesis of a branching epithelium	10 of 160	0.63	0.0188
GO:0072073	kidney epithelium development	8 of 128	0.63	0.0443
GO:0045165	cell fate commitment	12 of 230	0.56	0.0197
GO:0048598	embryonic morphogenesis	25 of 545	0.5	0.00082
GO:0040007	growth	16 of 357	0.49	0.0139
GO:0048589	developmental growth	15 of 340	0.48	0.0197
GO:0048638	regulation of developmental growth	13 of 302	0.47	0.0389
GO:0002009	morphogenesis of an epithelium	17 of 414	0.45	0.0188
GO:0001933	negative regulation of protein phosphorylation	17 of 422	0.44	0.0197
GO:0009790	embryo development	35 of 890	0.43	0.00062
GO:0042060	wound healing	18 of 461	0.43	0.0197
GO:0048729	tissue morphogenesis	20 of 522	0.42	0.0150
GO:0048568	embryonic organ development	16 of 417	0.42	0.0336
GO:0009611	response to wounding	20 of 547	0.4	0.0197
GO:0035239	tube morphogenesis	22 of 615	0.39	0.0153
GO:0007417	central nervous system development	29 of 861	0.37	0.0069
GO:0045596	negative regulation of cell differentiation	23 of 683	0.37	0.0197
GO:0031400	negative regulation of protein modification process	20 of 592	0.37	0.0336
GO:0051093	negative regulation of developmental process	30 of 910	0.36	0.0070
GO:0000902	cell morphogenesis	21 of 626	0.36	0.0310
GO:0035295	tube development	26 of 793	0.35	0.0153
GO:0035556	intracellular signal transduction	45 of 1528	0.31	0.0029
GO:0072359	circulatory system development	24 of 807	0.31	0.0496
GO:0009653	anatomical structure morphogenesis	55 of 1992	0.28	0.0018
GO:0001932	regulation of protein phosphorylation	38 of 1370	0.28	0.0150
GO:0045595	regulation of cell differentiation	46 of 1695	0.27	0.0070
GO:0051241	negative regulation of multicellular organismal process	30 of 1098	0.27	0.0451
GO:0071495	cellular response to endogenous stimulus	30 of 1106	0.27	0.0496
GO:0007399	nervous system development	57 of 2206	0.25	0.0035
GO:0048584	positive regulation of response to stimulus	53 of 2054	0.25	0.0069
GO:0009888	tissue development	42 of 1626	0.25	0.0197
GO:0009719	response to endogenous stimulus	35 of 1353	0.25	0.0443
GO:0050793	regulation of developmental process	61 of 2416	0.24	0.0035
GO:0010647	positive regulation of cell communication	41 of 1631	0.24	0.0319
GO:0023056	positive regulation of signaling	41 of 1638	0.24	0.0336
GO:0051239	regulation of multicellular organismal process	69 of 2788	0.23	0.0029
GO:0031399	regulation of protein modification process	43 of 1747	0.23	0.0336
GO:0048513	animal organ development	70 of 2926	0.22	0.0036
GO:0010646	regulation of cell communication	78 of 3327	0.21	0.0029
GO:0006357	regulation of transcription by RNA polymerase II	62 of 2633	0.21	0.0126
GO:0048731	system development	95 of 4144	0.2	0.00095
GO:0023051	regulation of signaling	78 of 3360	0.2	0.0035
GO:0009966	regulation of signal transduction	69 of 3033	0.2	0.0126
GO:0051173	positive regulation of nitrogen compound metabolic process	67 of 2946	0.2	0.0150

GO:0048583	regulation of response to stimulus	88 of 3882	0.19	0.0029
GO:0010604	positive regulation of macromolecule metabolic process	70 of 3081	0.19	0.0121
GO:0031325	positive regulation of cellular metabolic process	69 of 3060	0.19	0.0150
GO:0032502	developmental process	118 of 5401	0.18	0.00072
GO:0048856	anatomical structure development	112 of 5085	0.18	0.00082
GO:0007275	multicellular organism development	105 of 4726	0.18	0.00095
GO:0009893	positive regulation of metabolic process	72 of 3280	0.18	0.0188
GO:0048522	positive regulation of cellular process	105 of 4898	0.17	0.0029
GO:0006950	response to stress	70 of 3267	0.17	0.0310
GO:0048518	positive regulation of biological process	114 of 5459	0.16	0.0029
GO:0065008	regulation of biological quality	75 of 3559	0.16	0.0310
GO:0030154	cell differentiation	73 of 3457	0.16	0.0329
GO:2001141	regulation of RNA biosynthetic process	76 of 3691	0.15	0.0443
GO:0048519	negative regulation of biological process	99 of 4953	0.14	0.0200
GO:0048523	negative regulation of cellular process	90 of 4454	0.14	0.0279
GO:0032501	multicellular organismal process	126 of 6507	0.13	0.0114
GO:0060255	regulation of macromolecule metabolic process	117 of 6072	0.12	0.0197
GO:0051171	regulation of nitrogen compound metabolic process	112 of 5827	0.12	0.0273
GO:0031323	regulation of cellular metabolic process	116 of 6082	0.12	0.0273
GO:0080090	regulation of primary metabolic process	113 of 5982	0.11	0.0390
GO:0019222	regulation of metabolic process	121 of 6516	0.11	0.0425
GO:0050794	regulation of cellular process	186 of 10484	0.09	0.0070
GO:0065007	biological regulation	202 of 11740	0.07	0.0114
GO:0050789	regulation of biological process	190 of 11116	0.07	0.0273

(less ...)

Molecular Function (Gene Ontology)

GO-term	*description*	*count in network*	*strength*	*false discovery rate*
GO:0043565	sequence-specific DNA binding	35 of 1047	0.36	0.0041
GO:0003700	DNA-binding transcription factor activity	46 of 1749	0.26	0.0178
GO:0000981	DNA-binding transcription factor activity, RNA polymerase II-...	43 of 1633	0.26	0.0231
GO:0140110	transcription regulator activity	53 of 2069	0.25	0.0138

Reference publications (PubMed)

publication	*(year) title*	*count in network*	*strength*	*false discovery rate*
PMID:28624794	(2017) Knockdown of HDAC1 expression suppresses invasi...	7 of 22	1.34	0.0394
PMID:25378891	(2012) intracellular signaling by bile acids.	8 of 32	1.24	0.0394

KEGG Pathways

pathway	*description*	*count in network*	*strength*	*false discovery rate*
hsa04971	Gastric acid secretion	6 of 72	0.76	0.0479
hsa04912	GnRH signaling pathway	7 of 88	0.74	0.0311
hsa05220	Chronic myeloid leukemia	6 of 76	0.74	0.0498
hsa04072	Phospholipase D signaling pathway	10 of 145	0.68	0.0176
hsa05206	MicroRNAs in cancer	10 of 149	0.66	0.0176

Annotated Keywords (UniProt)

keyword	*description*	*count in network*	*strength*	*false discovery rate*
KW-0238	DNA-binding	48 of 1952	0.23	0.0356
KW-0539	Nucleus	102 of 5204	0.13	0.0377
KW-0597	Phosphoprotein	151 of 8067	0.11	0.0109

Social Acknowledgement (SAQ)

Mapping

#	queryItem	stringId	preferredName	annotation
1	ABCB11	9606.ENSP00000263817	ABCB11	Bile salt export pump; Involved in the ATP-dependent secretion of bile salts into the canaliculus of hepatocytes; Belongs to the ABC transporter superfamily. ABCB family. Multidrug resistance exporter (TC 3.A.1.201) subfamily
2	ABCC2	9606.ENSP00000359478	ABCC2	Canalicular multispecific organic anion transporter 1; Mediates hepatobiliary excretion of numerous organic anions. May function as a cellular cisplatin transporter; ATP binding cassette subfamily C
3	ABL1	9606.ENSP00000361423	ABL1	Tyrosine-protein kinase ABL1; Non-receptor tyrosine-protein kinase that plays a role in many key processes linked to cell growth and survival such as cytoskeleton remodeling in response to extracellular stimuli, cell motility and adhesion, receptor endocytosis, autophagy, DNA damage response and apoptosis. Coordinates actin remodeling through tyrosine phosphorylation of proteins controlling cytoskeleton dynamics like WASF3 (involved in branch formation); ANXA1 (involved in membrane anchoring); DBN1, DBNL, CTTN, RAPH1 and ENAH (involved in signaling); or MAPT and PXN (microtubule-bindin [...]
4	ACSBG2	9606.ENSP00000465589	ACSBG2	Long-chain-fatty-acid--CoA ligase ACSBG2; Mediates activation of long-chain fatty acids for both synthesis of cellular lipids, and degradation via beta-oxidation. Able to activate long-chain fatty acids. Also able to activate very long-chain fatty acids; however, the relevance of such activity is unclear in vivo. Has increased ability to activate oleic and linoleic acid. May play a role in spermatogenesis; Belongs to the ATP-dependent AMP-binding enzyme family. Bubblegum subfamily
5	ADCY7	9606.ENSP00000378187	ADCY7	Adenylate cyclase type 7; This is a membrane-bound, calcium-inhibitable adenylyl cyclase; Adenylate cyclases
6	ADRB3	9606.ENSP00000343782	ADRB3	Beta-3 adrenergic receptor; Beta-adrenergic receptors mediate the catecholamine- induced activation of adenylate cyclase through the action of G proteins. Beta-3 is involved in the regulation of lipolysis and thermogenesis; Adrenoceptors
7	AFTPH	9606.ENSP00000238855	AFTPH	Aftiphilin; May play a role in membrane trafficking
8	AGPAT3	9606.ENSP00000381140	AGPAT3	1-acyl-sn-glycerol-3-phosphate acyltransferase gamma; Converts lysophosphatidic acid (LPA) into phosphatidic acid by incorporating an acyl moiety at the sn-2 position of the glycerol backbone. Acts on LPA containing saturated or unsaturated fatty acids C16:0-C20:4 at the sn-1 position using C18:1, C20:4 or C18:2-CoA as the acyl donor. Also acts on lysophosphatidylcholine, lysophosphatidylinositol and lysophosphatidylserine using C18:1 or C20:4-CoA. Has a preference for arachidonoyl-CoA as a donor. Has also a modest lysophosphatidylinositol acyltransferase (LPIAT) activity, converts lys [...]
9	AJAP1	9606.ENSP00000367433	AJAP1	Adherens junction-associated protein 1; Plays a role in cell adhesion and cell migration
10	ANO1	9606.ENSP00000347454	ANO1	Anoctamin-1; Calcium-activated chloride channel (CaCC) which plays a role in transepithelial anion transport and smooth muscle contraction. Required for the normal functioning of the interstitial cells of Cajal (ICCs) which generate electrical pacemaker activity in gastrointestinal smooth muscles. Acts as a major contributor to basal and stimulated chloride conductance in airway epithelial cells and plays an important role in tracheal cartilage development; Belongs to the anoctamin family
11	ANO7	9606.ENSP00000274979	ANO7	Anoctamin-7; Has calcium-dependent phospholipid scramblase activity; scrambles phosphatidylserine, phosphatidylcholine and galactosylceramide (By similarity). Does not exhibit calcium- activated chloride channel (CaCC) activity. May play a role in cell-cell interactions; Anoctamins
12	ANXA5	9606.ENSP00000296511	ANXA5	Annexin A5; This protein is an anticoagulant protein that acts as an indirect inhibitor of the thromboplastin-specific complex, which is involved in the blood coagulation cascade; Annexins
13	AOPEP	9606.ENSP00000364464	C9orf3	Aminopeptidase O; Aminopeptidases catalyze the hydrolysis of amino acid residues from the N-terminus of peptide or protein substrates. Able to cleave angiotensin III to generate angiotensin IV, a bioactive peptide of the renin-angiotensin pathway. Not able to cleave angiotensin I and angiotensin II. May play a role in the proteolytic processing of bioactive peptides in tissues such as testis and heart; M1 metallopeptidases
14	AP3B2	9606.ENSP00000440984	AP3B2	AP-3 complex subunit beta-2; Subunit of non-clathrin- and clathrin-associated adaptor protein complex 3 (AP-3) that plays a role in protein sorting in the late-Golgi/trans-Golgi network (TGN) and/or endosomes. The AP complexes mediate both the recruitment of clathrin to membranes and the recognition of sorting signals within the cytosolic tails of transmembrane cargo molecules. AP-3 appears to be involved in the sorting of a subset of transmembrane proteins targeted to lysosomes and lysosome-related organelles. In concert with the BLOC-1 complex, AP-3 is required to target cargos into [...]
15	APBB2	9606.ENSP00000427211	APBB2	Amyloid-beta A4 precursor protein-binding family B member 2; May modulate the internalization of amyloid-beta precursor protein
16	ARF6	9606.ENSP00000298316	ARF6	ADP-ribosylation factor 6; GTP-binding protein involved in protein trafficking that regulates endocytic recycling and cytoskeleton remodeling. Required for normal completion of mitotic cytokinesis. Plays a role in the reorganization of the actin cytoskeleton and the formation of stress fibers. May also modulate vesicle budding and uncoating within the Golgi apparatus. Involved in the regulation of dendritic spine development, contributing to the regulation of dendritic branching and filopodia extension. Involved in epithelial polarization (By similarity).Functions as an allosteric acti [...]
17	ARL15	9606.ENSP00000433427	ARL15	ADP-ribosylation factor-like protein 15; ADP ribosylation factor like GTPase 15; ARF GTPase family
18	ARMC8	9606.ENSP00000420333	ARMC8	Armadillo repeat containing 8
19	ARNT2	9606.ENSP00000307479	ARNT2	Aryl hydrocarbon receptor nuclear translocator 2; Transcription factor that plays a role in the development of the hypothalamo-pituitary axis, postnatal brain growth, and visual and renal function. Specifically recognizes the xenobiotic response element (XRE); Basic helix-loop-helix proteins
20	ASB2	9606.ENSP00000451575	ASB2	Ankyrin repeat and SOCS box protein 2; Substrate-recognition component of a SCF-like ECS (Elongin-Cullin-SOCS-box protein) E3 ubiquitin-protein ligase complex which mediates the ubiquitination and subsequent proteasomal degradation of target proteins; Belongs to the ankyrin SOCS box (ASB) family
21	ATG2A	9606.ENSP00000366475	ATG2A	Autophagy-related protein 2 homolog A; Required for both autophagosome formation and regulation of lipid droplet morphology and dispersion; Belongs to the ATG2 family
22	ATP11A	9606.ENSP00000420387	ATP11A	Probable phospholipid-transporting ATPase IH; Catalytic component of a P4-ATPase flippase complex which catalyzes the hydrolysis of ATP coupled to the transport of aminophospholipids from the outer to the inner leaflet of various membranes and ensures the maintenance of asymmetric distribution of phospholipids. Phospholipid translocation seems also to be implicated in vesicle formation and in uptake of lipid signaling molecules (Probable). May be involved in the uptake of farnesyltransferase inhibitor drugs, such as lonafarnib
23	ATP6V0E1	9606.ENSP00000429690	ATP6V0E1	V-type proton ATPase subunit e 1; Vacuolar ATPase is responsible for acidifying a variety of intracellular compartments in eukaryotic cells; Belongs to the V-ATPase e1/e2 subunit family
24	AUTS2	9606.ENSP00000344087	AUTS2	Autism susceptibility gene 2 protein; Component of a Polycomb group (PcG) multiprotein PRC1- like complex, a complex class required to maintain the transcriptionally repressive state of many genes, including Hox genes, throughout development. PcG PRC1 complex acts via chromatin remodeling and modification of histones; it mediates monoubiquitination of histone H2A 'Lys-119', rendering chromatin heritably changed in its expressibility. The PRC1-like complex that contains PCGF5, RNF2, CSNK2B, RYBP and AUTS2 has decreased histone H2A ubiquitination activity, due to the phosphorylation of R [...]
25	AVEN	9606.ENSP00000306822	AVEN	Cell death regulator Aven; Protects against apoptosis mediated by Apaf-1
26	BCL2L11	9606.ENSP00000376943	BCL2L11	Bcl-2-like protein 11; Induces apoptosis and anoikis. Isoform BimL is more potent than isoform BimEL. Isoform Bim-alpha1, isoform Bim-alpha2 and isoform Bim-alpha3 induce apoptosis, although less potent than isoform BimEL, isoform BimL and isoform BimS. Isoform Bim-gamma induces apoptosis. Isoform Bim-alpha3 induces apoptosis possibly through a caspase-mediated pathway. Isoform BimAC and isoform BimABC lack the ability to induce apoptosis; Belongs to the Bcl-2 family
27	BOLA3	9606.ENSP00000331369	BOLA3	BolA-like protein 3; Acts as a mitochondrial iron-sulfur (Fe-S) cluster assembly factor that facilitates (Fe-S) cluster insertion into a subset of mitochondrial proteins. Probably acts together with NFU1; Belongs to the BolA/IbaG family
28	C10orf99	9606.ENSP00000361199	C10orf99	Protein GPR15L; Chemotactic factor that mediates lymphocytes recruitement to epithelia through binding and activation of the G-protein coupled receptor GPR15. May be a tumor suppressor; together with SUSD2 has a growth inhibitory effect on colon cancer cells which includes G1 cell cycle arrest
29	C15orf39	9606.ENSP00000353854	C15orf39	Uncharacterized protein C15orf39; Chromosome 15 open reading frame 39
30	C1QTNF9B	9606.ENSP00000371572	C1QTNF9B	Complement C1q and tumor necrosis factor-related protein 9B; C1q and TNF related 9B
31	C2CD2	9606.ENSP00000369853	C2CD2	C2 calcium dependent domain containing 2
32	C2CD4B	9606.ENSP00000369755	C2CD4B	C2 calcium-dependent domain-containing protein 4B; May be involved in inflammatory process. May regulate cell architecture and adhesion; Belongs to the C2CD4 family
33	C2orf83	9606.ENSP00000264387	C2orf83	Folate transporter-like protein C2orf83; Chromosome 2 open reading frame 83
34	C7orf50	9606.ENSP00000380286	C7orf50	Uncharacterized protein C7orf50; Chromosome 7 open reading frame 50
35	CABLES1	9606.ENSP00000256925	CABLES1	CDK5 and ABL1 enzyme substrate 1; Cyclin-dependent kinase binding protein. Enhances cyclin-dependent kinase tyrosine phosphorylation by nonreceptor tyrosine kinases, such as that of CDK5 by activated ABL1, which leads to increased CDK5 activity and is critical for neuronal development, and that of CDK2 by WEE1, which leads to decreased CDK2 activity and growth inhibition. Positively affects neuronal outgrowth. Plays a role as a regulator for p53/p73-induced cell death (By similarity)
36	CACNA2D2	9606.ENSP00000418081	CACNA2D2	Voltage-dependent calcium channel subunit alpha-2/delta-2; The alpha-2/delta subunit of voltage-dependent calcium channels regulates calcium current density and activation/inactivation kinetics of the calcium channel. Acts as a regulatory subunit for P/Q-type calcium channel (CACNA1A), N-type (CACNA1B), L-type (CACNA1C OR CACNA1D) and possibly T-type (CACNA1G). Overexpression induces apoptosis
37	CALM1	9606.ENSP00000349467	CALM1	Calmodulin-1; Calmodulin mediates the control of a large number of enzymes, ion channels, aquaporins and other proteins through calcium-binding. Among the enzymes to be stimulated by the calmodulin-calcium complex are a number of protein kinases and phosphatases. Together with CCP110 and centrin, is involved in a genetic pathway that regulates the centrosome cycle and progression through cytokinesis. Mediates calcium-dependent inactivation of CACNA1C. Positively regulates calcium-activated potassium channel activity of KCNN2
38	CASP3	9606.ENSP00000311032	CASP3	Caspase-3; Involved in the activation cascade of caspases responsible for apoptosis execution. At the onset of apoptosis it proteolytically cleaves poly(ADP-ribose) polymerase (PARP) at a '216-Asp-|-Gly-217' bond. Cleaves and activates sterol regulatory element binding proteins (SREBPs) between the basic helix-loop- helix leucine zipper domain and the membrane attachment domain. Cleaves and activates caspase-6, -7 and -9. Involved in the cleavage of huntingtin. Triggers cell adhesion in sympathetic neurons through RET cleavage
39	CASR	9606.ENSP00000420194	CASR	Extracellular calcium-sensing receptor; G-protein-coupled receptor that senses changes in the extracellular concentration of calcium ions and plays a key role in maintaining calcium homeostasis. Senses fluctuations in the circulating calcium concentration and modulates the production of parathyroid hormone (PTH) in parathyroid glands (By similarity). The activity of this receptor is mediated by a G-protein that activates a phosphatidylinositol- calcium second messenger system. The G-protein- coupled receptor activity is activated by a co-agonist mechanism: aromatic amino acids, such as [...]
40	CCDC113	9606.ENSP00000219299	CCDC113	Coiled-coil domain-containing protein 113; Component of centriolar satellites contributing to primary cilium formation
41	CCDC93	9606.ENSP00000365477	CCDC93	Coiled-coil domain-containing protein 93; Involved in copper-dependent ATP7A trafficking between the trans-Golgi network and vesicles in the cell periphery; the function is proposed to depend on its association within the CCC complex and cooperation with the WASH complex on early endosomes and is dependent on its interaction with WASHC2C
42	CCR6	9606.ENSP00000343952	GPR29	Chemokine (C-C motif) receptor 6; Receptor for the C-C type chemokine CCL20. Binds to CCL20 and subsequently transduces a signal by increasing the intracellular calcium ion levels. Although CCL20 is its major ligand it can also act as a receptor for non-chemokine ligands such as beta-defensins. Binds to defensin DEFB1 leading to increase in intracellular calcium ions and cAMP levels. Its binding to DEFB1 is essential for the function of DEFB1 in regulating sperm motility and bactericidal activity. Binds to defensins DEFB4 and DEFB4A/B and mediates their chemotactic effects. The ligand- [...]
43	CD177	9606.ENSP00000479536	CD177	CD177 antigen; In association with beta-2 integrin heterodimer ITGAM/CD11b and ITGB2/CD18, mediates activation of TNF-alpha primed neutrophils including degranulation and superoxide production. In addition, by preventing beta-2 integrin internalization and attenuating chemokine signaling favors adhesion over migration. Heterophilic interaction with PECAM1 on endothelial cells plays a role in neutrophil transendothelial migration in vitro. However, appears to be dispensable for neutrophil recruitment caused by bacterial infection in vivo. Acts as a receptor for the mature form of protea [...]

Social Acknowledgement (SAQ)

44	CDH4	9606.ENSP00000484928	CDH4	Cadherin-4; Cadherins are calcium-dependent cell adhesion proteins. They preferentially interact with themselves in a homophilic manner in connecting cells; cadherins may thus contribute to the sorting of heterogeneous cell types. May play an important role in retinal development
45	CDH5	9606.ENSP00000344115	CDH5	Cadherin-5; Cadherins are calcium-dependent cell adhesion proteins. They preferentially interact with themselves in a homophilic manner in connecting cells; cadherins may thus contribute to the sorting of heterogeneous cell types. This cadherin may play a important role in endothelial cell biology through control of the cohesion and organization of the intercellular junctions. It associates with alpha-catenin forming a link to the cytoskeleton. Acts in concert with KRIT1 to establish and maintain correct endothelial cell polarity and vascular lumen. These effects are mediated by recrui [...]
46	CDK6	9606.ENSP00000265734	CDK6	Cyclin-dependent kinase 6; Serine/threonine-protein kinase involved in the control of the cell cycle and differentiation; promotes G1/S transition. Phosphorylates pRB/RB1 and NPM1. Interacts with D-type G1 cyclins during interphase at G1 to form a pRB/RB1 kinase and controls the entrance into the cell cycle. Involved in initiation and maintenance of cell cycle exit during cell differentiation; prevents cell proliferation and regulates negatively cell differentiation, but is required for the proliferation of specific cell types (e.g. erythroid and hematopoietic cells). Essential for cel [...]
47	CHD7	9606.ENSP00000392028	CHD7	Chromodomain-helicase-DNA-binding protein 7; Probable transcription regulator. Maybe involved in the in 45S precursor rRNA production; DNA helicases
48	CHRDL2	9606.ENSP00000263671	CHRDL2	Chordin-related protein 2 variant III; Chordin like 2
49	CHRM3	9606.ENSP00000255380	CHRM3	Muscarinic acetylcholine receptor M3; The muscarinic acetylcholine receptor mediates various cellular responses, including inhibition of adenylate cyclase, breakdown of phosphoinositides and modulation of potassium channels through the action of G proteins. Primary transducing effect is Pi turnover; Cholinergic receptors muscarinic
50	CHRNA10	9606.ENSP00000250699	CHRNA10	Neuronal acetylcholine receptor subunit alpha-10; Ionotropic receptor with a probable role in the modulation of auditory stimuli. Agonist binding may induce an extensive change in conformation that affects all subunits and leads to opening of an ion-conducting channel across the plasma membrane. The channel is permeable to a range of divalent cations including calcium, the influx of which may activate a potassium current which hyperpolarizes the cell membrane. In the ear, this may lead to a reduction in basilar membrane motion, altering the activity of auditory nerve fibers and reducin [...]
51	CHSY1	9606.ENSP00000254190	CHSY1	Chondroitin sulfate synthase 1; Has both beta-1,3-glucuronic acid and beta-1,4-N- acetylgalactosamine transferase activity. Transfers glucuronic acid (GlcUA) from UDP-GlcUA and N-acetylgalactosamine (GalNAc) from UDP-GalNAc to the non-reducing end of the elongating chondroitin polymer. Involved in the negative control of osteogenesis likely through the modulation of NOTCH signaling
52	CLNK	9606.ENSP00000226951	CLNK	Cytokine-dependent hematopoietic cell linker; Plays a role in the regulation of immunoreceptor signaling, including PLC-gamma-mediated B-cell antigen receptor (BCR) signaling and FC-epsilon R1-mediated mast cell degranulation. Involved in phosphorylation of LAT (By similarity); SH2 domain containing
53	COG4	9606.ENSP00000315775	COG4	Conserved oligomeric Golgi complex subunit 4; Required for normal Golgi function. Plays a role in SNARE-pin assembly and Golgi-to-ER retrograde transport via its interaction with SCFD1; Belongs to the COG4 family
54	COL23A1	9606.ENSP00000375069	COL23A1	Collagen type XXIII alpha 1 chain; Collagens
55	CPEB1	9606.ENSP00000477715	CPEB1	Cytoplasmic polyadenylation element-binding protein 1; Sequence-specific RNA-binding protein that regulates mRNA cytoplasmic polyadenylation and translation initiation during oocyte maturation, early development and at postsynapse sites of neurons. Binds to the cytoplasmic polyadenylation element (CPE), an uridine-rich sequence element (consensus sequence 5'-UUUUUAU- 3') within the mRNA 3'-UTR. RNA binding results in a clear conformational change analogous to the Venus fly trap mechanism. In absence of phosphorylation and in association with TACC3 is also involved as a repressor of tra [...]
56	CREB5	9606.ENSP00000350359	CREB5	Cyclic AMP-responsive element-binding protein 5; Binds to the cAMP response element and activates transcription; Basic leucine zipper proteins
57	CRYBA4	9606.ENSP00000346805	CRYBA4	Beta-crystallin A4; Crystallins are the dominant structural components of the vertebrate eye lens; Belongs to the beta/gamma-crystallin family
58	CSDC2	9606.ENSP00000302485	CSDC2	Cold shock domain-containing protein C2; RNA-binding factor which binds specifically to the very 3'-UTR ends of both histone H1 and H3.3 mRNAs, encompassing the polyadenylation signal. Might play a central role in the negative regulation of histone variant synthesis in the developing brain (By similarity)
59	CSMD1	9606.ENSP00000430733	CSMD1	CUB and sushi domain-containing protein 1; Potential suppressor of squamous cell carcinomas; Protein phosphatase 1 regulatory subunits
60	CTNNA1	9606.ENSP00000304669	CTNNA1	Catenin alpha-1; Associates with the cytoplasmic domain of a variety of cadherins. The association of catenins to cadherins produces a complex which is linked to the actin filament network, and which seems to be of primary importance for cadherins cell-adhesion properties. Can associate with both E- and N-cadherins. Originally believed to be a stable component of E-cadherin/catenin adhesion complexes and to mediate the linkage of cadherins to the actin cytoskeleton at adherens junctions. In contrast, cortical actin was found to be much more dynamic than E-cadherin/catenin complexes and [...]
61	CUEDC1	9606.ENSP00000462717	CUEDC1	CUE domain containing 1
62	DAP	9606.ENSP00000230895	DAP	Death-associated protein 1; Negative regulator of autophagy. Involved in mediating interferon-gamma-induced cell death
63	DAPL1	9606.ENSP00000309538	DAPL1	Death-associated protein-like 1; May play a role in the early stages of epithelial differentiation or in apoptosis
65	DAW1	9606.ENSP00000311899	DAW1	Dynein assembly factor with WDR repeat domains 1; May play a role in axonemal outer row dynein assembly; Belongs to the WD repeat WDR69 family
66	DENND2A	9606.ENSP00000275884	DENND2A	DENN domain-containing protein 2A; Guanine nucleotide exchange factor (GEF) which may activate RAB9A and RAB9B. Promotes the exchange of GDP to GTP, converting inactive GDP-bound Rab proteins into their active GTP- bound form. May play a role in late endosomes back to trans-Golgi network/TGN transport; DENN/MADD domain containing
67	DGCR8	9606.ENSP00000263209	DGCR8	Microprocessor complex subunit DGCR8; Component of the microprocessor complex that acts as a RNA- and heme-binding protein that is involved in the initial step of microRNA (miRNA) biogenesis. Component of the microprocessor complex that is required to process primary miRNA transcripts (pri-miRNAs) to release precursor miRNA (pre-miRNA) in the nucleus. Within the microprocessor complex, DGCR8 function as a molecular anchor necessary for the recognition of pri-miRNA at dsRNA-ssRNA junction and directs DROSHA to cleave 11 bp away form the junction to release hairpin-shaped pre-miRNAs that [...]
68	DGKG	9606.ENSP00000265022	DGKG	Diacylglycerol kinase gamma; Reverses the normal flow of glycerolipid biosynthesis by phosphorylating diacylglycerol back to phosphatidic acid; Belongs to the eukaryotic diacylglycerol kinase family
69	DIP2C	9606.ENSP00000280886	DIP2C	Disco interacting protein 2 homolog C
70	DLL4	9606.ENSP00000249749	DLL4	Delta-like protein 4; Involved in the Notch signaling pathway as Notch ligand. Activates NOTCH1 and NOTCH4. Involved in angiogenesis; negatively regulates endothelial cell proliferation and migration and angiogenic sprouting. Essential for retinal progenitor proliferation. Required for suppressing rod fates in late retinal progenitors as well as for proper generation of other retinal cell types (By similarity). During spinal cord neurogenesis, inhibits V2a interneuron fate
71	DNAJC5	9606.ENSP00000354111	DNAJC5	DnaJ homolog subfamily C member 5; Acts as a general chaperone in regulated exocytosis (By similarity). Acts as a co-chaperone for the SNARE protein SNAP-25 (By similarity). Involved in the calcium-mediated control of a late stage of exocytosis (By similarity). May have an important role in presynaptic function. May be involved in calcium-dependent neurotransmitter release at nerve endings (By similarity); DNAJ heat shock proteins
72	DNMT3A	9606.ENSP00000264709	DNMT3A	DNA (cytosine-5)-methyltransferase 3A; Required for genome-wide de novo methylation and is essential for the establishment of DNA methylation patterns during development. DNA methylation is coordinated with methylation of histones. It modifies DNA in a non-processive manner and also methylates non-CpG sites. May preferentially methylate DNA linker between 2 nucleosomal cores and is inhibited by histone H1. Plays a role in paternal and maternal imprinting. Required for methylation of most imprinted loci in germ cells. Acts as a transcriptional corepressor for ZBTB18. Recruited to trimet [...]
73	DPYSL5	9606.ENSP00000288699	DPYSL5	Dihydropyrimidinase-related protein 5; May have a function in neuronal differentiation and/or axon growth; Belongs to the metallo-dependent hydrolases superfamily. Hydantoinase/dihydropyrimidinase family
74	DRGX	9606.ENSP00000363254	DRGX	Dorsal root ganglia homeobox protein; Transcription factor required for the formation of correct projections from nociceptive sensory neurons to the dorsal horn of the spinal cord and normal perception of pain; PRD class homeoboxes and pseudogenes
75	DYSF	9606.ENSP00000386881	DYSF	Dysferlin; Key calcium ion sensor involved in the Ca(2+)-triggered synaptic vesicle-plasma membrane fusion. Plays a role in the sarcolemma repair mechanism of both skeletal muscle and cardiomyocytes that permits rapid resealing of membranes disrupted by mechanical stress (By similarity); Ferlin family
76	EBF3	9606.ENSP00000357637	EBF3	Transcription factor COE3; Transcriptional activator. Recognizes variations of the palindromic sequence 5'-ATTCCCNNGGGAATT-3' (By similarity); Belongs to the COE family
77	EFCAB1	9606.ENSP00000262103	EFCAB1	EF-hand calcium-binding domain-containing protein 1; EF-hand calcium binding domain 1
78	EIPR1	9606.ENSP00000371559	TSSC1	EARP-interacting protein; Tumor suppressing subtransferable candidate 1
79	ELMO1	9606.ENSP00000312185	ELMO1	Engulfment and cell motility protein 1; Involved in cytoskeletal rearrangements required for phagocytosis of apoptotic cells and cell motility. Acts in association with DOCK1 and CRK. Was initially proposed to be required in complex with DOCK1 to activate Rac Rho small GTPases. May enhance the guanine nucleotide exchange factor (GEF) activity of DOCK1
80	ENDOU	9606.ENSP00000397679	ENDOU	Poly(U)-specific endoribonuclease; Endoribonuclease that cleaves single-stranded RNAs at uridylates and releases products that have 2'-3'-cyclic phosphate termini; Serine proteases
81	EPB41L2	9606.ENSP00000338481	EPB41L2	Band 4.1-like protein 2; Required for dynein-dynactin complex and NUMA1 recruitment at the mitotic cell cortex during anaphase; Erythrocyte membrane protein band 4.1
82	EVI5	9606.ENSP00000359356	EVI5	Ecotropic viral integration site 5 protein homolog; Functions as a regulator of cell cycle progression by stabilizing the FBXO5 protein and promoting cyclin-A accumulation during interphase. May play a role in cytokinesis
83	EXTL1	9606.ENSP00000363398	EXTL1	Exostosin-like 1; Probable glycosyltransferase; Belongs to the glycosyltransferase 47 family
84	EZR	9606.ENSP00000356042	EZR	Ezrin; Probably involved in connections of major cytoskeletal structures to the plasma membrane. In epithelial cells, required for the formation of microvilli and membrane ruffles on the apical pole. Along with PLEKHG6, required for normal macropinocytosis; A-kinase anchoring proteins
85	F7	9606.ENSP00000364731	F7	Coagulation factor VII; Initiates the extrinsic pathway of blood coagulation. Serine protease that circulates in the blood in a zymogen form. Factor VII is converted to factor VIIa by factor Xa, factor XIIa, factor IXa, or thrombin by minor proteolysis. In the presence of tissue factor and calcium ions, factor VIIa then converts factor X to factor Xa by limited proteolysis. Factor VIIa will also convert factor IX to factor IXa in the presence of tissue factor and calcium; Gla domain containing
86	FAM110A	9606.ENSP00000354163	FAM110A	Protein FAM110A; Family with sequence similarity 110 member A; Belongs to the FAM110 family
87	FAM124B	9606.ENSP00000386895	FAM124B	Protein FAM124B; Family with sequence similarity 124 member B; Belongs to the FAM124 family
88	FAM155A	9606.ENSP00000365080	FAM155A	Transmembrane protein FAM155A; Family with sequence similarity 155 member A
89	FAM160B1	9606.ENSP00000358251	FAM160B1	Protein FAM160B1; Family with sequence similarity 160 member B1; Armadillo-like helical domain containing
90	FAM168A	9606.ENSP00000064778	FAM168A	Protein FAM168A; In cancer context, protects cells from induced-DNA damage and apoptosis. Acts, at least in part, through PI3K/AKT/NFKB signaling pathway and by preventing POLB degradation. Decreases POLB ubiquitation and stabilizes its protein levels
91	FAM174B	9606.ENSP00000329040	FAM174B	Membrane protein FAM174B; Family with sequence similarity 174 member B
92	FAM228A	9606.ENSP00000295150	FAM228A	Protein FAM228A; Family with sequence similarity 228 member A; Belongs to the FAM228 family
93	FAM3D	9606.ENSP00000351632	FAM3D	Protein FAM3D; Family with sequence similarity 3 member D; Belongs to the FAM3 family
94	FBRSL1	9606.ENSP00000396160	FBRSL1	Fibrosin-1-like protein; Fibrosin like 1; Belongs to the AUTS2 family
95	FCN3	9606.ENSP00000270879	FCN3	Ficolin-3; May function in innate immunity through activation of the lectin complement pathway. Calcium-dependent and GlcNAc-binding lectin. Has affinity with GalNAc, GlcNAc, D-fucose, as mono/oligosaccharide and lipopolysaccharides from S.typhimurium and S.minnesota; Fibrinogen C domain containing
96	FILIP1L	9606.ENSP00000346560	FILIP1L	Filamin A-interacting protein 1-like; Acts as a regulator of the antiangiogenic activity on endothelial cells. When overexpressed in endothelial cells, leads to inhibition of cell proliferation and migration and an increase in apoptosis. Inhibits melanoma growth When expressed in tumor- associated vasculature
97	FN1	9606.ENSP00000346839	FN1	Fibronectin type III domain containing; Endogenous ligands

98	FOXD1	9606.ENSP00000481581	FOXD1	Forkhead box protein D1; Transcription factor involved in regulation of gene expression in a variety of processes, including formation of positional identity in the developing retina, regionalization of the optic chiasm, morphogenesis of the kidney, and neuralization of ectodermal cells (By similarity). Involved in transcriptional activation of PGF and C3 genes; Forkhead boxes
99	FOXN2	9606.ENSP00000343633	FOXN2	Forkhead box protein N2; Binds to the purine-rich region in HTLV-I LTR; Forkhead boxes
100	FOXN3	9606.ENSP00000343288	FOXN3	Forkhead box protein N3; Acts as a transcriptional repressor. May be involved in DNA damage-inducible cell cycle arrests (checkpoints); Forkhead boxes
101	FOXP2	9606.ENSP00000386200	FOXP2	Forkhead box protein P2; Transcriptional repressor that may play a role in the specification and differentiation of lung epithelium. May also play a role in developing neural, gastrointestinal and cardiovascular tissues. Can act with CTBP1 to synergistically repress transcription but CTPBP1 is not essential. Plays a role in synapse formation by regulating SRPX2 levels. Involved in neural mechanisms mediating the development of speech and language; Forkhead boxes
102	FRG1	9606.ENSP00000226798	FRG1	Protein FRG1; Binds to mRNA in a sequence-independent manner. May play a role in regulation of pre-mRNA splicing or in the assembly of rRNA into ribosomal subunits. May be involved in mRNA transport. May be involved in epigenetic regulation of muscle differentiation through regulation of activity of the histone-lysine N- methyltransferase KMT5B; Belongs to the FRG1 family
103	FRMD1	9606.ENSP00000283309	FRMD1	FERM domain containing 1
104	GADD45B	9606.ENSP00000215631	GADD45B	Growth arrest and DNA damage-inducible protein GADD45 beta; Involved in the regulation of growth and apoptosis. Mediates activation of stress-responsive MTK1/MEKK4 MAPKKK
105	GAL3ST4	9606.ENSP00000353142	GAL3ST4	Galactose-3-O-sulfotransferase 4; Catalyzes the transfer of sulfate to beta-1,3-linked galactose residues in O-linked glycoproteins. Good substrates include asialofetuin, Gal-beta-1,3-GalNAc and Gal-beta-1,3 (GlcNAc-beta-1,6)GalNAc; Sulfotransferases, membrane bound
106	GALNT10	9606.ENSP00000297107	GALNT10	Polypeptide N-acetylgalactosaminyltransferase 10; Catalyzes the initial reaction in O-linked oligosaccharide biosynthesis, the transfer of an N-acetyl-D- galactosamine residue to a serine or threonine residue on the protein receptor. Has activity toward Muc5Ac and EA2 peptide substrates; Polypeptide N-acetylgalactosaminyltransferases
108	GDF6	9606.ENSP00000287020	GDF6	Growth/differentiation factor 6; Growth factor that controls proliferation and cellular differentiation in the retina and bone formation. Plays a key role in regulating apoptosis during retinal development. Establishes dorsal-ventral positional information in the retina and controls the formation of the retinotectal map. Required for normal formation of bones and joints in the limbs, skull, digits and axial skeleton. Plays a key role in establishing boundaries between skeletal elements during development. Regulation of GDF6 expression seems to be a mechanism for evolving species-specif [...]
109	GINS4	9606.ENSP00000276533	GINS4	DNA replication complex GINS protein SLD5; The GINS complex plays an essential role in the initiation of DNA replication, and progression of DNA replication forks. GINS4 is important for GINS complex assembly. GINS complex seems to bind preferentially to single-stranded DNA
110	GLRX3	9606.ENSP00000357633	GLRX3	Glutaredoxin-3; Together with BOLA2, acts as a cytosolic iron-sulfur (Fe-S) cluster assembly factor that facilitates [2Fe-2S] cluster insertion into a subset of cytosolic proteins. Acts as a critical negative regulator of cardiac hypertrophy and a positive inotropic regulator (By similarity). Required for hemoglobin maturation. Does not possess any thyoredoxin activity since it lacks the conserved motif that is essential for catalytic activity; Glutaredoxin domain containing
111	GNA11	9606.ENSP00000078429	GNA11	Guanine nucleotide-binding protein subunit alpha-11; Guanine nucleotide-binding proteins (G proteins) are involved as modulators or transducers in various transmembrane signaling systems. Acts as an activator of phospholipase C; G protein subunits alpha, group q
112	GNA12	9606.ENSP00000275364	GNA12	Guanine nucleotide-binding protein subunit alpha-12; Guanine nucleotide-binding proteins (G proteins) are involved as modulators or transducers in various transmembrane signaling systems. Activates effector molecule RhoA by binding and activating RhoGEFs (ARHGEF12/LARG). GNA12-dependent Rho signaling subsequently regulates transcription factor AP-1 (activating protein-1) (By similarity). GNA12-dependent Rho signaling also regulates protein phosphatese 2A activation causing dephosphorylation of its target proteins. Promotes tumor cell invasion and metastasis by activating RhoA/ROCK sign [...]
113	GNAS	9606.ENSP00000360141	GNAS	Guanine nucleotide-binding protein G(s) subunit alpha isoforms XLas; Guanine nucleotide-binding proteins (G proteins) function as transducers in numerous signaling pathways controlled by G protein-coupled receptors (GPCRs). Signaling involves the activation of adenylyl cyclases, resulting in increased levels of the signaling molecule cAMP. GNAS functions downstream of several GPCRs, including beta-adrenergic receptors. XLas isoforms interact with the same set of receptors as GNAS isoforms (By similarity)
114	GOLGA8J	9606.ENSP00000456401	GOLGA8J	Golgin A8 family member J; Belongs to the GOLGA8 family
115	GPM6A	9606.ENSP00000280187	GPM6A	Neuronal membrane glycoprotein M6-a; Involved in neuronal differentiation, including differentiation and migration of neuronal stem cells. Plays a role in neuronal plasticity and is involved in neurite and filopodia outgrowth, filopodia motility and probably synapse formation. GPM6A-induced filopodia formation involves mitogen-activated protein kinase (MAPK) and Src signaling pathways. May be involved in neuronal NGF-dependent Ca(2+) influx. May be involved in regulation of endocytosis and intracellular trafficking of G-protein-coupled receptors (GPCRs); enhances internalization and r [...]
116	GPR88	9606.ENSP00000314223	GPR88	Probable G-protein coupled receptor 88; Probable G-protein coupled receptor implicated in a large repertoire of behavioral responses that engage motor activities, spatial learning, and emotional processing. May play a role in the regulation of cognitive and motor function
117	GRB2	9606.ENSP00000376345	GRB2	Growth factor receptor-bound protein 2; Adapter protein that provides a critical link between cell surface growth factor receptors and the Ras signaling pathway; SH2 domain containing
118	GSAP	9606.ENSP00000257626	GSAP	Gamma-secretase-activating protein; Regulator of gamma-secretase activity, which specifically activates the production of amyloid-beta protein (amyloid-beta protein 40 and amyloid-beta protein 42), without affecting the cleavage of other gamma-secretase targets such has Notch. The gamma-secretase complex is an endoprotease complex that catalyzes the intramembrane cleavage of integral membrane proteins such as Notch receptors and APP (amyloid-beta precursor protein). Specifically promotes the gamma-cleavage of APP CTF-alpha (also named APP-CTF) by the gamma-secretase complex to generate [...]
119	GTF2F2	9606.ENSP00000340823	GTF2F2	General transcription factor IIF subunit 2; TFIIF is a general transcription initiation factor that binds to RNA polymerase II and helps to recruit it to the initiation complex in collaboration with TFIIB. It promotes transcription elongation. This subunit shows ATP-dependent DNA- helicase activity
121	HDAC9	9606.ENSP00000408617	HDAC9	Histone deacetylase 9; Responsible for the deacetylation of lysine residues on the N-terminal part of the core histones (H2A, H2B, H3 and H4). Histone deacetylation gives a tag for epigenetic repression and plays an important role in transcriptional regulation, cell cycle progression and developmental events. Represses MEF2-dependent transcription; Belongs to the histone deacetylase family. HD type 2 subfamily
122	HM13	9606.ENSP00000381237	HM13	Histocompatibility minor 13
123	HOXA11	9606.ENSP00000006015	HOXA11	Homeobox protein Hox-A11; Sequence-specific transcription factor which is part of a developmental regulatory system that provides cells with specific positional identities on the anterior-posterior axis; HOXL subclass homeoboxes
124	HPSE2	9606.ENSP00000359583	HPSE2	Inactive heparanase-2; Binds heparin and heparan sulfate with high affinity, but lacks heparanase activity. Inhibits HPSE, possibly by competing for its substrates (in vitro)
125	HRH3	9606.ENSP00000342560	HRH3	Histamine H3 receptor; The H3 subclass of histamine receptors could mediate the histamine signals in CNS and peripheral nervous system. Signals through the inhibition of adenylate cyclase and displays high constitutive activity (spontaneous activity in the absence of agonist). Agonist stimulation of isoform 3 neither modified adenylate cyclase activity nor induced intracellular calcium mobilization
126	IDS	9606.ENSP00000339801	IDS	Iduronate 2-sulfatase; Required for the lysosomal degradation of heparan sulfate and dermatan sulfate; Sulfatases
127	IFFO1	9606.ENSP00000482285	IFFO1	Intermediate filament family orphan 1; Belongs to the intermediate filament family
128	IFI30	9606.ENSP00000384886	IFI30	Gamma-interferon-inducible lysosomal thiol reductase; Lysosomal thiol reductase that can reduce protein disulfide bonds. May facilitate the complete unfolding of proteins destined for lysosomal degradation. Plays an important role in antigen processing. Facilitates the generation of MHC class II- restricted epitodes from disulfide bond-containing antigen by the endocytic reduction of disulfide bonds (By similarity). Facilitates also MHC class I-restricted recognition of exogenous antigens containing disulfide bonds by CD8+ T-cells or crosspresentation (By similarity)
129	INPP5D	9606.ENSP00000405338	INPP5D	Phosphatidylinositol 3,4,5-trisphosphate 5-phosphatase 1; Phosphatidylinositol (PtdIns) phosphatase that specifically hydrolyzes the 5-phosphate of phosphatidylinositol- 3,4,5-trisphosphate (PtdIns(3,4,5)P3) to produce PtdIns(3,4)P2, thereby negatively regulating the PI3K (phosphoinositide 3-kinase) pathways. Acts as a negative regulator of B-cell antigen receptor signaling. Mediates signaling from the FC-gamma-RIIB receptor (FCGR2B), playing a central role in terminating signal transduction from activating immune/hematopoietic cell receptor systems. Acts as a negative regulator of mye [...]
130	IRX2	9606.ENSP00000372056	IRX2	Iroquois-class homeodomain protein IRX-2; TALE class homeoboxes and pseudogenes
131	ISL2	9606.ENSP00000290759	ISL2	Insulin gene enhancer protein ISL-2; Transcriptional factor that defines subclasses of motoneurons that segregate into columns in the spinal cord and select distinct axon pathways; LIM class homeoboxes
132	ITCH	9606.ENSP00000480499	ITCH	E3 ubiquitin-protein ligase Itchy homolog; Acts as an E3 ubiquitin-protein ligase which accepts ubiquitin from an E2 ubiquitin-conjugating enzyme in the form of a thioester and then directly transfers the ubiquitin to targeted substrates. Catalyzes 'Lys-29'-, 'Lys-48'- and 'Lys-63'-linked ubiquitin conjugation. Involved in the control of inflammatory signaling pathways. Essential component of a ubiquitin-editing protein complex, comprising also TNFAIP3, TAX1BP1 and RNF11, that ensures the transient nature of inflammatory signaling pathways. Promotes the association of the complex after [...]
133	ITGA5	9606.ENSP00000293379	ITGA5	Integrin alpha-5; Integrin alpha-5/beta-1 is a receptor for fibronectin and fibrinogen. It recognizes the sequence R-G-D in its ligands. ITGA5:ITGB1 binds to PLA2G2A via a site (site 2) which is distinct from the classical ligand-binding site (site 1) and this induces integrin conformational changes and enhanced ligand binding to site 1. ITGA5:ITGB1 acts as a receptor for fibrillin-1 (FBN1) and mediates R-G-D-dependent cell adhesion to FBN1; CD molecules
134	JARID2	9606.ENSP00000341280	JARID2	Protein Jumonji; Regulator of histone methyltransferase complexes that plays an essential role in embryonic development, including heart and liver development, neural tube fusion process and hematopoiesis. Acts by modulating histone methyltransferase activity and promoting the recruitment of histone methyltransferase complexes to their target genes. Binds DNA and mediates the recruitment of the PRC2 complex to target genes in embryonic stem cells. Does not have histone demethylase activity but regulates activity of various histone methyltransferase complexes. In embryonic stem cells, i [...]
135	JMJD7	9606.ENSP00000380467	JMJD7	JmjC domain-containing protein 7; Jumonji domain containing 7
136	KCNJ10	9606.ENSP00000357068	KCNJ10	ATP-sensitive inward rectifier potassium channel 10; May be responsible for potassium buffering action of glial cells in the brain. Inward rectifier potassium channels are characterized by a greater tendency to allow potassium to flow into the cell rather than out of it. Their voltage dependence is regulated by the concentration of extracellular potassium; as external potassium is raised, the voltage range of the channel opening shifts to more positive voltages. The inward rectification is mainly due to the blockage of outward current by internal magnesium. Can be blocked by extracellu [...]
137	KIF13B	9606.ENSP00000427900	KIF13B	Kinesin-like protein KIF13B; Involved in reorganization of the cortical cytoskeleton. Regulates axon formation by promoting the formation of extra axons. May be functionally important for the intracellular trafficking of MAGUKs and associated protein complexes; Kinesins
138	KLHL6	9606.ENSP00000341342	KLHL6	Kelch-like protein 6; Involved in B-lymphocyte antigen receptor signaling and germinal center formation; BTB domain containing
139	KLK7	9606.ENSP00000375683	KLK7	Kallikrein-7; May catalyze the degradation of intercellular cohesive structures in the cornified layer of the skin in the continuous shedding of cells from the skin surface. Specific for amino acid residues with aromatic side chains in the P1 position. Cleaves insulin A chain at '14-Tyr-|-Gln-15' and insulin B chain at '6- Leu-|-Cys-7', '16-Tyr-|-Leu-17', '25-Phe-|-Tyr-26' and '26-Tyr-|- Thr-27'. Could play a role in the activation of precursors to inflammatory cytokines; Kallikreins
140	KREMEN2	9606.ENSP00000304422	KREMEN2	Kremen protein 2; Receptor for Dickkopf proteins. Cooperates with DKK1/2 to inhibit Wnt/beta-catenin signaling by promoting the endocytosis of Wnt receptors LRP5 and LRP6. Plays a role in limb development; attenuates Wnt signaling in the developing limb to allow normal limb patterning and can also negatively regulate bone formation
141	LAS1L	9606.ENSP00000363944	LAS1L	Ribosomal biogenesis protein LAS1L; Involved in the biogenesis of the 60S ribosomal subunit. Required for maturation of the 28S rRNA. Functions as a component of the Five Friends of Methylated CHTOP (5FMC) complex; the 5FMC complex is recruited to ZNF148 by methylated CHTOP, leading to desumoylation of ZNF148 and subsequent transactivation of ZNF148 target genes
142	LDLRAD4	9606.ENSP00000352420	LDLRAD4	Low-density lipoprotein receptor class A domain-containing protein 4; Functions as a negative regulator of TGF-beta signaling and thereby probably plays a role in cell proliferation, differentiation, apoptosis, motility, extracellular matrix production and immunosuppression. In the canonical TGF-beta pathway, ZFYVE9/SARA recruits the intracellular signal transducer and transcriptional

				modulators SMAD2 and SMAD3 to the TGF-beta receptor. Phosphorylated by the receptor, SMAD2 and SMAD3 then form a heteromeric complex with SMAD4 that translocates to the nucleus to regulate transcription. [...]
144	LTB4R	9606.ENSP00000380008	LTB4R	Leukotriene B4 receptor 1; Receptor for extracellular ATP > UTP and ADP. The activity of this receptor is mediated by G proteins which activate a phosphatidylinositol-calcium second messenger system. May be the cardiac P2Y receptor involved in the regulation of cardiac muscle contraction through modulation of L-type calcium currents. Is a receptor for leukotriene B4, a potent chemoattractant involved in inflammation and immune response; Belongs to the G-protein coupled receptor 1 family
145	LYZ	9606.ENSP00000261267	LYZ	Lysozyme C; Lysozymes have primarily a bacteriolytic function; those in tissues and body fluids are associated with the monocyte-macrophage system and enhance the activity of immunoagents; Lysozymes, c-type
146	MAFB	9606.ENSP00000362410	MAFB	Transcription factor MafB; Acts as a transcriptional activator or repressor. Plays a pivotal role in regulating lineage- specific hematopoiesis by repressing ETS1-mediated transcription of erythroid-specific genes in myeloid cells. Required for monocytic, macrophage, osteoclast, podocyte and islet beta cell differentiation. Involved in renal tubule survival and F4/80 maturation. Activates the insulin and glucagon promoters. Together with PAX6, transactivates weakly the glucagon gene promoter through the G1 element. SUMO modification controls its transcriptional activity and ability to [...]
147	MAGEA9B	9606.ENSP00000243314	ENSG00000267978	Melanoma antigen family A, 9B; Not known, though may play a role in embryonal development and tumor transformation or aspects of tumor progression; MAGE family
148	MALAT1	9606.ENSP00000485396	ENSG00000279576	Metastasis-associated lung adenocarcinoma transcript 1
149	MAP2K3	9606.ENSP00000345083	MAP2K3	Dual specificity mitogen-activated protein kinase kinase 3; Dual specificity kinase. Is activated by cytokines and environmental stress in vivo. Catalyzes the concomitant phosphorylation of a threonine and a tyrosine residue in the MAP kinase p38. Part of a signaling cascade that begins with the activation of the adrenergic receptor ADRA1B and leads to the activation of MAPK14; Belongs to the protein kinase superfamily. STE Ser/Thr protein kinase family. MAP kinase kinase subfamily
150	MAP2K4	9606.ENSP00000410402	MAP2K4	Dual specificity mitogen-activated protein kinase kinase 4; Dual specificity protein kinase which acts as an essential component of the MAP kinase signal transduction pathway. Essential component of the stress-activated protein kinase/c-Jun N-terminal kinase (SAP/JNK) signaling pathway. With MAP2K7/MKK7, is the one of the only known kinase to directly activate the stress-activated protein kinase/c-Jun N-terminal kinases MAPK8/JNK1, MAPK9/JNK2 and MAPK10/JNK3. MAP2K4/MKK4 and MAP2K7/MKK7 both activate the JNKs by phosphorylation, but they differ in their preference for the phosphorylati [...]
152	MB21D2	9606.ENSP00000376246	MB21D2	Protein MB21D2; Mab-21 domain containing 2
153	MBP	9606.ENSP00000380958	MBP	Myelin basic protein; The classic group of MBP isoforms (isoform 4-isoform 14) are with PLP the most abundant protein components of the myelin membrane in the CNS. They have a role in both its formation and stabilization. The smaller isoforms might have an important role in remyelination of denuded axons in multiple sclerosis. The non- classic group of MBP isoforms (isoform 1-isoform 3/Golli-MBPs) may preferentially have a role in the early developing brain long before myelination, maybe as components of transcriptional complexes, and may also be involved in signaling pathways in T- ce [...]
154	MET	9606.ENSP00000317272	MET	Hepatocyte growth factor receptor; Receptor tyrosine kinase that transduces signals from the extracellular matrix into the cytoplasm by binding to hepatocyte growth factor/HGF ligand. Regulates many physiological processes including proliferation, scattering, morphogenesis and survival. Ligand binding at the cell surface induces autophosphorylation of MET on its intracellular domain that provides docking sites for downstream signaling molecules. Following activation by ligand, interacts with the PI3-kinase subunit PIK3R1, PLCG1, SRC, GRB2, STAT3 or the adapter GAB1. Recruitment of thes [...]
155	MLN	9606.ENSP00000388825	MLN	Promotilin; Plays an important role in the regulation of interdigestive gastrointestinal motility and indirectly causes rhythmic contraction of duodenal and colonic smooth muscle; Endogenous ligands
156	MLXIP	9606.ENSP00000312834	MLXIP	MLX-interacting protein; Binds DNA as a heterodimer with MLX and activates transcription. Binds to the canonical E box sequence 5'-CACGTG-3'. Plays a role in transcriptional activation of glycolytic target genes. Involved in glucose-responsive gene regulation; Basic helix-loop-helix proteins
157	MLXIPL	9606.ENSP00000320886	MLXIPL	Carbohydrate-responsive element-binding protein; Transcriptional repressor. Binds to the canonical and non-canonical E box sequences 5'-CACGTG-3' (By similarity); Basic helix-loop-helix proteins
158	MSI2	9606.ENSP00000284073	MSI2	RNA-binding protein Musashi homolog 2; RNA binding protein that regulates the expression of target mRNAs at the translation level. May play a role in the proliferation and maintenance of stem cells in the central nervous system (By similarity); Belongs to the Musashi family
159	MTHFD1L	9606.ENSP00000478253	MTHFD1L	Monofunctional C1-tetrahydrofolate synthase, mitochondrial; May provide the missing metabolic reaction required to link the mitochondria and the cytoplasm in the mammalian model of one-carbon folate metabolism in embryonic an transformed cells complementing thus the enzymatic activities of MTHFD2; In the N-terminal section; belongs to the tetrahydrofolate dehydrogenase/cyclohydrolase family
160	MUC3A	9606.ENSP00000368771	MUC3A	Mucin-3A; Major glycoprotein component of a variety of mucus gels. Thought to provide a protective, lubricating barrier against particles and infectious agents at mucosal surfaces. May be involved in ligand binding and intracellular signaling; Mucins
161	MYC	9606.ENSP00000479618	MYC	Myc proto-oncogene protein; Transcription factor that binds DNA in a non-specific manner, yet also specifically recognizes the core sequence 5'- CAC[GA]TG-3'. Activates the transcription of growth-related genes. Binds to the VEGFA promoter, promoting VEGFA production and subsequent sprouting angiogenesis; Basic helix-loop-helix proteins
162	N4BP1	9606.ENSP00000262384	N4BP1	NEDD4-binding protein 1; Inhibitor of the E3 ubiquitin-protein ligase ITCH. Acts by interacting with the second WW domain of ITCH, leading to compete with ITCH's substrates and impairing ubiquitination of substrates (By similarity); Belongs to the N4BP1 family
163	NAP1L5	9606.ENSP00000320488	NAP1L5	Nucleosome assembly protein 1 like 5
164	NAPB	9606.ENSP00000482826	NAPB	Beta-soluble NSF attachment protein; Required for vesicular transport between the endoplasmic reticulum and the Golgi apparatus
165	NAV2	9606.ENSP00000379396	NAV2	Neuron navigator 2; Possesses 3' to 5' helicase activity and exonuclease activity. Involved in neuronal development, specifically in the development of different sensory organs; Belongs to the Nav/unc-53 family
166	NBN	9606.ENSP00000265433	NBN	Nibrin; Component of the MRE11-RAD50-NBN (MRN complex) which plays a critical role in the cellular response to DNA damage and the maintenance of chromosome integrity. The complex is involved in double-strand break (DSB) repair, DNA recombination, maintenance of telomere integrity, cell cycle checkpoint control and meiosis. The complex possesses single-strand endonuclease activity and double-strand-specific 3'-5' exonuclease activity, which are provided by MRE11. RAD50 may be required to bind DNA ends and hold them in close proximity. NBN modulate the DNA damage signal sensing by recrui [...]
167	NFAM1	9606.ENSP00000333680	NFAM1	NFAT activation molecule 1; May function in immune system as a receptor which activates via the calcineurin/NFAT-signaling pathway the downstream cytokine gene promoters. Activates the transcription of IL-13 and TNF-alpha promoters. May be involved in the regulation of B-cell, but not T-cell, development. Overexpression activates downstream effectors without ligand binding or antibody cross- linking
168	NFKBIL1	9606.ENSP00000365318	NFKBIL1	NF-kappa-B inhibitor-like protein 1; Involved in the regulation of innate immune response. Acts as negative regulator of Toll-like receptor and interferon- regulatory factor (IRF) signaling pathways. Contributes to the negative regulation of transcriptional activation of NF-kappa-B target genes in response to endogenous proinflammatory stimuli
169	NFKBIZ	9606.ENSP00000325663	NFKBIZ	NF-kappa-B inhibitor zeta; Involved in regulation of NF-kappa-B transcription factor complexes. Inhibits NF-kappa-B activity without affecting its nuclear translocation upon stimulation. Inhibits DNA-binding of RELA and NFKB1/p50, and of the NF-kappa-B p65-p50 heterodimer and the NF-kappa-B p50-p50 homodimer. Seems also to activate NF- kappa-B-mediated transcription. In vitro, upon association with NFKB1/p50 has transcriptional activation activity and, together with NFKB1/p50 and RELA, is recruited to LCN2 promoters. Promotes transcription of LCN2 and DEFB4. Is recruited to IL-6 promot [...]
170	NINL	9606.ENSP00000278886	NINL	Ninein-like protein; Involved in the microtubule organization in interphase cells. Overexpression induces the fragmentation of the Golgi, and causes lysosomes to disperse toward the cell periphery; it also interferes with mitotic spindle assembly. May play a role in ovarian carcinogenesis; EF-hand domain containing
171	NKX2-6	9606.ENSP00000320089	NKX2-6	Homeobox protein Nkx-2.6; Acts as a transcriptional activator. In conjunction with NKX2-5, may play a role in both pharyngeal and cardiac embryonic development; NKL subclass homeoboxes and pseudogenes
172	NOL11	9606.ENSP00000253247	NOL11	Nucleolar protein 11; Ribosome biogenesis factor. May be required for both optimal rDNA transcription and small subunit (SSU) pre-rRNA processing at sites A', A0, 1 and 2b; UTPa subcomplex
173	NPC2	9606.ENSP00000451112	NPC2	Epididymal secretory protein E1; Intracellular cholesterol transporter which acts in concert with NPC1 and plays an important role in the egress of cholesterol from the endosomal/lysosomal compartment. Both NPC1 and NPC2 function as the cellular 'tag team duo' (TTD) to catalyze the mobilization of cholesterol within the multivesicular environment of the late endosome (LE) to effect egress through the limiting bilayer of the LE. NPC2 binds unesterified cholesterol that has been released from LDLs in the lumen of the late endosomes/lysosomes and transfers it to the cholesterol-binding po [...]
174	NR2E1	9606.ENSP00000357979	NR2E1	Nuclear receptor subfamily 2 group E member 1; Orphan receptor that binds DNA as a monomer to hormone response elements (HRE) containing an extended core motif half- site sequence 5'-AAGGTCA-3' in which the 5' flanking nucleotides participate in determining receptor specificity (By similarity). May be required to pattern anterior brain differentiation. Involved in the regulation of retinal development and essential for vision. During retinogenesis, regulates PTEN-Cyclin D expression via binding to the promoter region of PTEN and suppressing its activity (By similarity). May be involved [...]
175	NR3C1	9606.ENSP00000231509	NR3C1	Glucocorticoid receptor; Isoform Alpha-D3: Has lowest transcriptional activation activity of all isoforms created by alternative initiation. Has transcriptional repression activity; Nuclear hormone receptors
176	NRG1	9606.ENSP00000384620	NRG1	Pro-neuregulin-1, membrane-bound isoform; Direct ligand for ERBB3 and ERBB4 tyrosine kinase receptors. Concomitantly recruits ERBB1 and ERBB2 coreceptors, resulting in ligand-stimulated tyrosine phosphorylation and activation of the ERBB receptors. The multiple isoforms perform diverse functions such as inducing growth and differentiation of epithelial, glial, neuronal, and skeletal muscle cells; inducing expression of acetylcholine receptor in synaptic vesicles during the formation of the neuromuscular junction; stimulating lobuloalveolar budding and milk production in the mammary gla [...]
177	NSMCE1	9606.ENSP00000355077	NSMCE1	Non-structural maintenance of chromosomes element 1 homolog; Component of the SMC5-SMC6 complex, a complex involved in DNA double-strand breaks by homologous recombination. The complex may promote sister chromatid homologous recombination by recruiting the SMC1-SMC3 cohesin complex to double-strand breaks. The complex is required for telomere maintenance via recombination in ALT (alternative lengthening of telomeres) cell lines and mediates sumoylation of shelterin complex (telosome) components which is proposed to lead to shelterin complex disassembly in ALT- associated PML bodies (AP [...]
178	NXPE3	9606.ENSP00000396421	NXPE3	NXPE family member 3; Neurexophilin and PC-esterase domain family member 3
179	OTX1	9606.ENSP00000282549	OTX1	Homeobox protein OTX1; Probably plays a role in the development of the brain and the sense organs. Can bind to the BCD target sequence (BTS): 5'-TCTAATCCC-3'; PRD class homeoboxes and pseudogenes
180	PCDH19	9606.ENSP00000362125	PCDH19	Protocadherin-19; Potential calcium-dependent cell-adhesion protein; Non-clustered protocadherins
181	PDE10A	9606.ENSP00000438284	PDE10A	cAMP and cAMP-inhibited cGMP 3',5'-cyclic phosphodiesterase 10A; Plays a role in signal transduction by regulating the intracellular concentration of cyclic nucleotides. Can hydrolyze both cAMP and cGMP, but has higher affinity for cAMP and is more efficient with cAMP as substrate. May play a critical role in regulating cAMP and cGMP levels in the striatum, a region of the brain that contributes to the control of movement and cognition; Phosphodiesterases
182	PDE7B	9606.ENSP00000310661	PDE7B	cAMP-specific 3',5'-cyclic phosphodiesterase 7B; Hydrolyzes the second messenger cAMP, which is a key regulator of many important physiological processes. May be involved in the control of cAMP-mediated neural activity and cAMP metabolism in the brain; Phosphodiesterases
183	PHKB	9606.ENSP00000313504	PHKB	Phosphorylase b kinase regulatory subunit beta; Phosphorylase b kinase catalyzes the phosphorylation of serine in certain substrates, including troponin I. The beta chain acts as a regulatory unit and modulates the activity of the holoenzyme in response to phosphorylation
184	PHLDA1	9606.ENSP00000266671	PHLDA1	Pleckstrin homology-like domain family A member 1; Seems to be involved in regulation of apoptosis. May be involved in detachment-mediated programmed cell death. May mediate apoptosis during neuronal development. May be involved in regulation of anti-apoptotic effects of IGF1. May be involved in translational regulation
185	PHLDB2	9606.ENSP00000405405	PHLDB2	Pleckstrin homology-like domain family B member 2; Seems to be involved in the assembly of the postsynaptic apparatus. May play a role in acetyl-choline receptor (AChR) aggregation in the postsynaptic membrane (By similarity); Pleckstrin homology domain containing

Social Acknowledgement (SAQ)

186	PIK3R5	9606.ENSP00000392812	PIK3R5	Phosphoinositide 3-kinase regulatory subunit 5; Regulatory subunit of the PI3K gamma complex. Required for recruitment of the catalytic subunit to the plasma membrane via interaction with beta-gamma G protein dimers. Required for G protein-mediated activation of PIK3CG (By similarity)
187	PIP5K1B	9606.ENSP00000265382	PIP5K1B	Phosphatidylinositol 4-phosphate 5-kinase type-1 beta; Participates in the biosynthesis of phosphatidylinositol 4,5-bisphosphate. Mediates RAC1-dependent reorganization of actin filaments. Contributes to the activation of PLD2. Together with PIP5K1A is required after stimulation of G-protein coupled receptors for stable platelet adhesion (By similarity)
188	PITRM1	9606.ENSP00000370377	PITRM1	Presequence protease, mitochondrial; ATP-independent protease that degrades mitochondrial transit peptides after their cleavage. Also degrades other unstructured peptides. Specific for peptides in the range of 10 to 65 residues. Able to degrade amyloid beta A4 (APP) protein when it accumulates in mitochondrion, suggesting a link with Alzheimer disease. Shows a preference for cleavage after small polar residues and before basic residues, but without any positional preference; Belongs to the peptidase M16 family. PreP subfamily
189	PITX1	9606.ENSP00000265340	PITX1	Pituitary homeobox 1; Sequence-specific transcription factor that binds gene promoters and activates their transcription. May play a role in the development of anterior structures, and in particular, the brain and facies and in specifying the identity or structure of hindlimb; Belongs to the paired homeobox family. Bicoid subfamily
190	PLCE1	9606.ENSP00000360431	PLCE1	1-phosphatidylinositol 4,5-bisphosphate phosphodiesterase epsilon-1; The production of the second messenger molecules diacylglycerol (DAG) and inositol 1,4,5-trisphosphate (IP3) is mediated by activated phosphatidylinositol-specific phospholipase C enzymes. PLCE1 is a bifunctional enzyme which also regulates small GTPases of the Ras superfamily through its Ras guanine- exchange factor (RasGEF) activity. As an effector of heterotrimeric and small G-protein, it may play a role in cell survival, cell growth, actin organization and T-cell activation; C2 domain containing phospholipases
191	PMP22	9606.ENSP00000484631	PMP22	Peripheral myelin protein 22; Might be involved in growth regulation, and in myelinization in the peripheral nervous system; Belongs to the PMP-22/EMP/MP20 family
192	POLI	9606.ENSP00000462664	POLI	DNA polymerase iota; Error-prone DNA polymerase specifically involved in DNA repair. Plays an important role in translesion synthesis, where the normal high-fidelity DNA polymerases cannot proceed and DNA synthesis stalls. Favors Hoogsteen base-pairing in the active site. Inserts the correct base with high-fidelity opposite an adenosine template. Exhibits low fidelity and efficiency opposite a thymidine template, where it will preferentially insert guanosine. May play a role in hypermutation of immunogobulin genes. Forms a Schiff base with 5'-deoxyribose phosphate at abasic sites, but [...]
193	PPA2	9606.ENSP00000343885	PPA2	Inorganic pyrophosphatase 2, mitochondrial; Hydrolyzes inorganic pyrophosphate. This activity is essential for correct regulation of mitochondrial membrane potential, and mitochondrial organization and function
194	PPARD	9606.ENSP00000310928	PPARD	Peroxisome proliferator-activated receptor delta; Ligand-activated transcription factor. Receptor that binds peroxisome proliferators such as hypolipidemic drugs and fatty acids. Has a preference for poly-unsaturated fatty acids, such as gamma-linoleic acid and eicosapentanoic acid. Once activated by a ligand, the receptor binds to promoter elements of target genes. Regulates the peroxisomal beta-oxidation pathway of fatty acids. Functions as transcription activator for the acyl-CoA oxidase gene. Decreases expression of NPC1L1 once activated by a ligand; Belongs to the nuclear hormone [...]
195	PPP4R4	9606.ENSP00000305924	PPP4R4	Serine/threonine-protein phosphatase 4 regulatory subunit 4; Putative regulatory subunit of serine/threonine-protein phosphatase 4; Armadillo-like helical domain containing
196	PRDM10	9606.ENSP00000351686	PRDM10	PR domain zinc finger protein 10; May be involved in transcriptional regulation; PR/SET domain family
197	PRDM16	9606.ENSP00000270722	PRDM16	PR domain zinc finger protein 16; Binds DNA and functions as a transcriptional regulator. Functions in the differentiation of brown adipose tissue (BAT) which is specialized in dissipating chemical energy in the form of heat in response to cold or excess feeding while white adipose tissue (WAT) is specialized in the storage of excess energy and the control of systemic metabolism. Together with CEBPB, regulates the differentiation of myoblastic precursors into brown adipose cells. Functions also as a repressor of TGF-beta signaling. Isoform 4 may regulate granulocytes differentiation; L [...]
199	PRKCZ	9606.ENSP00000367830	PRKCZ	Protein kinase C zeta type; Calcium- and diacylglycerol-independent serine/threonine-protein kinase that functions in phosphatidylinositol 3-kinase (PI3K) pathway and mitogen-activated protein (MAP) kinase cascade, and is involved in NF-kappa-B activation, mitogenic signaling, cell proliferation, cell polarity, inflammatory response and maintenance of long-term potentiation (LTP). Upon lipopolysaccharide (LPS) treatment in macrophages, or following mitogenic stimuli, functions downstream of PI3K to activate MAP2K1/MEK1-MAPK1/ERK2 signaling cascade independently of RAF1 activation. Requ [...]
200	PRPH	9606.ENSP00000257860	PRPH	Peripherin; Class-III neuronal intermediate filament protein
201	PRR5	9606.ENSP00000384848	PRR5	Proline-rich protein 5; Subunit of mTORC2, which regulates cell growth and survival in response to hormonal signals. mTORC2 is activated by growth factors, but, in contrast to mTORC1, seems to be nutrient- insensitive. mTORC2 seems to function upstream of Rho GTPases to regulate the actin cytoskeleton, probably by activating one or more Rho-type guanine nucleotide exchange factors. mTORC2 promotes the serum-induced formation of stress-fibers or F-actin. mTORC2 plays a critical role in AKT1 'Ser-473' phosphorylation, which may facilitate the phosphorylation of the activation loop of AKT [...]
202	PTGFRN	9606.ENSP00000376899	PTGFRN	Prostaglandin F2 receptor negative regulator; Inhibits the binding of prostaglandin F2-alpha (PGF2- alpha) to its specific FP receptor, by decreasing the receptor number rather than the affinity constant. Functional coupling with the prostaglandin F2-alpha receptor seems to occur (By similarity); CD molecules
203	PTPN13	9606.ENSP00000394794	PTPN13	Tyrosine-protein phosphatase non-receptor type 13; Tyrosine phosphatase which regulates negatively FAS- induced apoptosis and NGFR-mediated pro-apoptotic signaling. May regulate phosphoinositide 3-kinase (PI3K) signaling through dephosphorylation of PIK3R2; FERM domain containing
204	R3HDM1	9606.ENSP00000387010	R3HDM1	R3H domain containing 1
205	RAB6C	9606.ENSP00000387307	RAB6C	Ras-related protein Rab-6C; May be involved in the regulation of centrosome duplication and cell cycle progression; RAB, member RAS oncogene GTPases
206	RBFOX3	9606.ENSP00000463653	RBFOX3	RNA binding protein fox-1 homolog 3; Pre-mRNA alternative splicing regulator. Regulates alternative splicing of RBFOX2 to enhance the production of mRNA species that are targeted for nonsense-mediated decay (NMD); RNA binding motif containing
207	RBPMS2	9606.ENSP00000300069	RBPMS2	RNA-binding protein with multiple splicing 2; Contributes to the regulation of smooth muscle cell differentiation and proliferation in the gastrointestinal system. Binds NOG mRNA. Mediates an increase of NOG mRNA levels, and thereby contributes to the negative regulation of the BMP signaling pathway. This promotes reversible dedifferentiation of smooth muscle cells and promotes smooth muscle cell proliferation; RNA binding motif containing
208	RFFL	9606.ENSP00000326170	RFFL	E3 ubiquitin-protein ligase rififylin; E3 ubiquitin-protein ligase that regulates several biological processes through the ubiquitin-mediated proteasomal degradation of various target proteins. Mediates 'Lys-48'-linked polyubiquitination of PRR5L and its subsequent proteasomal degradation thereby indirectly regulating cell migration through the mTORC2 complex. Ubiquitinates the caspases CASP8 and CASP10, promoting their proteasomal degradation, to negatively regulate cell death downstream of death domain receptors in the extrinsic pathway of apoptosis. Negatively regulates the tumor ne [...]
209	RHCG	9606.ENSP00000268122	RHCG	Ammonium transporter Rh type C; Functions as an electroneutral and bidirectional ammonium transporter. May regulate transepithelial ammonia secretion; Solute carriers
210	RLIM	9606.ENSP00000328059	RLIM	E3 ubiquitin-protein ligase RLIM; E3 ubiquitin-protein ligase. Acts as a negative coregulator for LIM homeodomain transcription factors by mediating the ubiquitination and subsequent degradation of LIM cofactors LDB1 and LDB2 and by mediating the recruitment the SIN3a/histone deacetylase corepressor complex. Ubiquitination and degradation of LIM cofactors LDB1 and LDB2 allows DNA-bound LIM homeodomain transcription factors to interact with other protein partners such as RLIM. Plays a role in telomere length-mediated growth suppression by mediating the ubiquitination and degradation of [...]
211	RNF111	9606.ENSP00000453872	RNF111	E3 ubiquitin-protein ligase Arkadia; E3 ubiquitin-protein ligase. Required for mesoderm patterning during embryonic development (By similarity). Acts as an enhancer of the transcriptional responses of the SMAD2/SMAD3 effectors, which are activated downstream of BMP. Acts by mediating ubiquitination and degradation of SMAD inhibitors such as SMAD7, inducing their proteasomal degradation and thereby enhancing the transcriptional activity of TGF-beta and BMP. In addition to enhance transcription of SMAD2/SMAD3 effectors, also regulates their turnover by mediating their ubiquitination and [...]
212	ROPN1	9606.ENSP00000184183	ROPN1	Ropporin-1A; Important for male fertility. With ROPN1L, involved in fibrous sheath integrity and sperm motility, plays a role in PKA-dependent signaling processes required for spermatozoa capacitation; Belongs to the ropporin family
213	RORA	9606.ENSP00000261523	RORA	Nuclear receptor ROR-alpha; Nuclear receptor that binds DNA as a monomer to ROR response elements (RORE) containing a single core motif half-site 5'-AGGTCA-3' preceded by a short A-T-rich sequence. Key regulator of embryonic development, cellular differentiation, immunity, circadian rhythm as well as lipid, steroid, xenobiotics and glucose metabolism. Considered to have intrinsic transcriptional activity, have some natural ligands like oxysterols that act as agonists (25-hydroxycholesterol) or inverse agonists (7-oxygenated sterols), enhancing or repressing the transcriptional activity [...]
215	RSPO3	9606.ENSP00000349131	RSPO3	R-spondin-3; Activator of the canonical Wnt signaling pathway by acting as a ligand for LGR4-6 receptors, which acts as a key regulator of angiogenesis. Upon binding to LGR4-6 (LGR4, LGR5 or LGR6), LGR4-6 associate with phosphorylated LRP6 and frizzled receptors that are activated by extracellular Wnt receptors, triggering the canonical Wnt signaling pathway to increase expression of target genes. Also regulates the canonical Wnt/beta- catenin-dependent pathway and non-canonical Wnt signaling by acting as an inhibitor of ZNRF3, an important regulator of the Wnt signaling pathway. Acts [...]
216	SCG2	9606.ENSP00000304133	SCG2	Secretogranin-2; Secretogranin-2 is a neuroendocrine secretory granule protein, which is the precursor for biologically active peptides; Granins
217	SEMA3B	9606.ENSP00000484146	SEMA3B	Semaphorin-3B; Inhibits axonal extension by providing local signals to specify territories inaccessible for growing axons; Belongs to the semaphorin family
218	SH2B3	9606.ENSP00000345492	SH2B3	SH2B adapter protein 3; Links T-cell receptor activation signal to phospholipase C-gamma-1, GRB2 and phosphatidylinositol 3-kinase; Pleckstrin homology domain containing
219	SHANK2	9606.ENSP00000469689	SHANK2	SH3 and multiple ankyrin repeat domains protein 2; Seems to be an adapter protein in the postsynaptic density (PSD) of excitatory synapses that interconnects receptors of the postsynaptic membrane including NMDA-type and metabotropic glutamate receptors, and the actin-based cytoskeleton. May play a role in the structural and functional organization of the dendritic spine and synaptic junction; Belongs to the SHANK family
220	SHC3	9606.ENSP00000364995	SHC3	SHC-transforming protein 3; Signaling adapter that couples activated growth factor receptors to signaling pathway in neurons. Involved in the signal transduction pathways of neurotrophin-activated Trk receptors in cortical neurons; SH2 domain containing
221	SIGLEC15	9606.ENSP00000374125	SIGLEC15	Sialic acid-binding Ig-like lectin 15; Binds sialylated glycoproteins; Sialic acid binding Ig like lectins
222	SIX2	9606.ENSP00000304502	SIX2	Homeobox protein SIX2; Transcription factor that plays an important role in the development of several organs, including kidney, skull and stomach. During kidney development, maintains cap mesenchyme multipotent nephron progenitor cells in an undifferentiated state by opposing the inductive signals emanating from the ureteric bud and cooperates with WNT9B to promote renewing progenitor cells proliferation. Acts through its interaction with TCF7L2 and OSR1 in a canonical Wnt signaling independent manner preventing transcription of differentiation genes in cap mesenchyme such as WNT4. Al [...]
223	SKAP2	9606.ENSP00000005587	SKAP2	Src kinase-associated phosphoprotein 2; May be involved in B-cell and macrophage adhesion processes. In B-cells, may act by coupling the B-cell receptor (BCR) to integrin activation. May play a role in src signaling pathway; Belongs to the SKAP family
224	SLC16A9	9606.ENSP00000378757	SLC16A9	Monocarboxylate transporter 9; Proton-linked monocarboxylate transporter. May catalyze the transport of monocarboxylates across the plasma membrane; Solute carriers
225	SLC19A1	9606.ENSP00000308895	SLC19A1	Folate transporter 1; Transporter for the intake of folate. Uptake of folate in human placental choriocarcinoma cells occurs by a novel mechanism called potocytosis which functionally couples three components, namely the folate receptor, the folate transporter, and a V-type H(+)-pump; Minor histocompatibility antigens
226	SLC1A7	9606.ENSP00000478639	SLC1A7	Amino acid transporter; Solute carrier family 1 member 7
227	SLC23A2	9606.ENSP00000368637	SLC23A2	Solute carrier family 23 member 2; Sodium/ascorbate cotransporter. Mediates electrogenic uptake of vitamin C, with a stoichiometry of 2 Na(+) for each ascorbate; Solute carriers
228	SLC2A4	9606.ENSP00000320935	SLC2A4	Solute carrier family 2, facilitated glucose transporter member 4; Insulin-regulated facilitative glucose transporter; Solute carriers
229	SLC38A2	9606.ENSP00000256689	SLC38A2	Sodium-coupled neutral amino acid transporter 2; Functions as a sodium-dependent amino acid transporter. Mediates the saturable, pH-sensitive and electrogenic cotransport of neutral amino acids and sodium ions with a stoichiometry of 1:1. May function in the transport of amino acids at the blood- brain barrier and in the supply of maternal nutrients to the fetus through the placenta

230	SLCO1A2	9606.ENSP00000305974	SLCO1A2	Solute carrier organic anion transporter family member 1A2; Mediates the Na(+)-independent transport of organic anions such as sulfobromophthalein (BSP) and conjugated (taurocholate) and unconjugated (cholate) bile acids (By similarity). Selectively inhibited by the grapefruit juice component naringin; Solute carriers
231	SMAD5	9606.ENSP00000441954	SMAD5	Mothers against decapentaplegic homolog 5; Transcriptional modulator activated by BMP (bone morphogenetic proteins) type 1 receptor kinase. SMAD5 is a receptor-regulated SMAD (R-SMAD)
232	SMAD6	9606.ENSP00000288840	SMAD6	Mothers against decapentaplegic homolog 6; Acts as a mediator of TGF-beta and BMP antiflammatory activity. Suppresses IL1R-TLR signaling through its direct interaction with PEL1, preventing NF-kappa-B activation, nuclear transport and NF-kappa-B-mediated expression of proinflammatory genes. May block the BMP-SMAD1 signaling pathway by competing with SMAD4 for receptor-activated SMAD1-binding. Binds to regulatory elements in target promoter regions
233	SMS	9606.ENSP00000385746	SMS	Spermine synthase; Catalyzes the production of spermine from spermidine and decarboxylated S-adenosylmethionine (dcSAM)
234	SORCS3	9606.ENSP00000358715	SORCS3	Sortilin related VPS10 domain containing receptor 3; Belongs to the VPS10-related sortilin family. SORCS subfamily
235	SOX2	9606.ENSP00000323588	SOX2	Transcription factor SOX-2; Transcription factor that forms a trimeric complex with OCT4 on DNA and controls the expression of a number of genes involved in embryonic development such as YES1, FGF4, UTF1 and ZFP206 (By similarity). Critical for early embryogenesis and for embryonic stem cell pluripotency. May function as a switch in neuronal development. Downstream SRRT target that mediates the promotion of neural stem cell self-renewal (By similarity). Keeps neural cells undifferentiated by counteracting the activity of proneural proteins and suppresses neuronal differentiation (By si [...]
236	SPRY1	9606.ENSP00000481675	SPRY1	Protein sprouty homolog 1; May function as an antagonist of fibroblast growth factor (FGF) pathways and may negatively modulate respiratory organogenesis
237	SQSTM1	9606.ENSP00000374455	SQSTM1	Sequestosome-1; Autophagy receptor that interacts directly with both the cargo to become degraded and an autophagy modifier of the MAP1 LC3 family. Along with WDFY3, involved in the formation and autophagic degradation of cytoplasmic ubiquitin-containing inclusions (p62 bodies, ALIS/aggresome-like induced structures). Along with SQSTM1, required to recruit ubiquitinated proteins to PML bodies in the nucleus. May regulate the activation of NFKB1 by TNF-alpha, nerve growth factor (NGF) and interleukin-1. May play a role in titin/TTN downstream signaling in muscle cells. May regulate sign [...]
238	ST3GAL1	9606.ENSP00000428540	ST3GAL1	CMP-N-acetylneuraminate-beta-galactosamide-alpha-2,3-sialyltransferase 1; Responsible for the synthesis of the sequence NeuAc-alpha-2,3-Gal-beta-1,3-GalNAc- found on sugar chains O-linked to Thr or Ser and also as a terminal sequence on certain gangliosides. SIAT4A and SIAT4B sialylate the same acceptor substrates but exhibit different Km values; Belongs to the glycosyltransferase 29 family
239	ST6GALNAC1	9606.ENSP00000156626	ST6GALNAC1	ST6 N-acetylgalactosaminide alpha-2,6-sialyltransferase 1; Sialyltransferases
240	STAG3	9606.ENSP00000477973	STAG3	Cohesin subunit SA-3; Meiosis specific component of cohesin complex. The cohesin complex is required for the cohesion of sister chromatids after DNA replication. The cohesin complex apparently forms a large proteinaceous ring within which sister chromatids can be trapped. At anaphase, the complex is cleaved and dissociates from chromatin, allowing sister chromatids to segregate. The meiosis-specific cohesin complex probably replaces mitosis specific cohesin complex when it dissociates from chromatin during prophase I; Armadillo-like helical domain containing
241	STEAP2	9606.ENSP00000378119	STEAP2	Metalloreductase STEAP2; Metalloreductase that has the ability to reduce both Fe(3+) to Fe(2+) and Cu(2+) to Cu(1+). Uses NAD(+) as acceptor (By similarity); STEAP family
242	STK10	9606.ENSP00000176763	STK10	Serine/threonine-protein kinase 10; Serine/threonine-protein kinase involved in regulation of lymphocyte migration. Phosphorylates MSN, and possibly PLK1. Involved in regulation of lymphocyte migration by mediating phosphorylation of ERM proteins such as MSN. Acts as a negative regulator of MAP3K1/MEKK1. May also act as a cell cycle regulator by acting as a polo kinase kinase: mediates phosphorylation of PLK1 in vitro; however such data require additional evidences in vivo; Belongs to the protein kinase superfamily. STE Ser/Thr protein kinase family. STE20 subfamily
243	STRADA	9606.ENSP00000336655	STRADA	STE20-related kinase adapter protein alpha; Pseudokinase which, in complex with CAB39/MO25 (CAB39/MO25alpha or CAB39L/MO25beta), binds to and activates STK11/LKB1. Adopts a closed conformation typical of active protein kinases and binds STK11/LKB1 as a pseudosubstrate, promoting conformational change of STK11/LKB1 in an active conformation
244	SULT2B1	9606.ENSP00000201586	SULT2B1	Sulfotransferase family cytosolic 2B member 1; Sulfotransferase that utilizes 3'-phospho-5'-adenylyl sulfate (PAPS) as sulfonate donor to catalyze the sulfate conjugation of many hormones, neurotransmitters, drugs and xenobiotic compounds. Sulfonation increases the water solubility of most compounds, and therefore their renal excretion, but it can also result in bioactivation to form active metabolites. Sulfates hydroxysteroids like DHEA. Isoform 1 preferentially sulfonates cholesterol, and isoform 2 avidly sulfonates pregnenolone but not cholesterol. Plays a role in epidermal choleste [...]
245	SUSD1	9606.ENSP00000363382	SUSD1	Sushi domain containing 1
246	SYNE2	9606.ENSP00000350719	SYNE2	Nesprin-2; Multi-isomeric modular protein which forms a linking network between organelles and the actin cytoskeleton to maintain the subcellular spatial organization. As a component of the LINC (LInker of Nucleoskeleton and Cytoskeleton) complex involved in the connection between the nuclear lamina and the cytoskeleton. The nucleocytoplasmic interactions established by the LINC complex play an important role in the transmission of mechanical forces across the nuclear envelope and in nuclear movement and positioning. Specifically, SYNE2 and SUN2 assemble in arrays of transmembrane acti [...]
247	SYT7	9606.ENSP00000444201	SYT7	Synaptotagmin-7; Ca(2+) sensor involved in Ca(2+)-dependent exocytosis of secretory and synaptic vesicles through Ca(2+) and phospholipid binding to the C2 domain (By similarity). Ca(2+) induces binding of the C2-domains to phospholipid membranes and to assembled SNARE-complexes; both actions contribute to triggering exocytosis (By similarity). SYT7 binds Ca(2+) with high affinity and slow kinetics compared to other synaptotagmins (By similarity). Involved in Ca(2+)-triggered lysosomal exocytosis, a major component of the plasma membrane repair. Ca(2+)- regulated delivery of lysosomal [...]
248	TAB3	9606.ENSP00000368215	TAB3	TGF-beta-activated kinase 1 and MAP3K7-binding protein 3; Adapter linking MAP3K7/TAK1 and TRAF6 or TRAF2. Mediator of MAP3K7 activation, respectively in the IL1 and TNF signaling pathways. Plays a role in activation of NF-kappa-B and AP1 transcription factor. Isoform 2 may be an oncogenic factor; Zinc fingers RANBP2-type
249	TAFA5	9606.ENSP00000383933	FAM19A5	Protein FAM19A5; Family with sequence similarity 19 member A5, C-C motif chemokine like; Belongs to the FAM19/TAFA family
250	TBL1XR1	9606.ENSP00000405574	TBL1XR1	F-box-like/WD repeat-containing protein TBL1XR1; F-box-like protein involved in the recruitment of the ubiquitin/19S proteasome complex to nuclear receptor-regulated transcription units. Plays an essential role in transcription activation mediated by nuclear receptors. Probably acts as integral component of the N-Cor corepressor complex that mediates the recruitment of the 19S proteasome complex, leading to the subsequent proteasomal degradation of N-Cor complex, thereby allowing cofactor exchange, and transcription activation; Belongs to the WD repeat EBI family
251	TBX4	9606.ENSP00000240335	TBX4	T-box transcription factor TBX4; Involved in the transcriptional regulation of genes required for mesoderm differentiation. Probably plays a role in limb pattern formation; T-boxes
252	TDRKH	9606.ENSP00000357812	TDRKH	Tudor and KH domain-containing protein; Participates in the primary piRNA biogenesis pathway and is required during spermatogenesis to repress transposable elements and prevent their mobilization, which is essential for the germline integrity. The piRNA metabolic process mediates the repression of transposable elements during meiosis by forming complexes composed of piRNAs and Piwi proteins and govern the methylation and subsequent repression of transposons. Required for the final steps of primary piRNA biogenesis by participating in the processing of 31-37 nt intermediates into mature [...]
253	TENM4	9606.ENSP00000278550	TENM4	Teneurin-4; Involved in neural development, regulating the establishment of proper connectivity within the nervous system. Plays a role in the establishment of the anterior-posterior axis during gastrulation. Regulates the differentiation and cellular process formation of oligodendrocytes and myelination of small- diameter axons in the central nervous system (CNS). Promotes activation of focal adhesion kinase. May function as a cellular signal transducer (By similarity); Belongs to the tenascin family. Teneurin subfamily
254	TEX29	9606.ENSP00000283547	TEX29	Testis-expressed protein 29; Testis expressed 29
255	THBS4	9606.ENSP00000339730	THBS4	Thrombospondin-4; Adhesive glycoprotein that mediates cell-to-cell and cell-to-matrix interactions and is involved in various processes including cellular proliferation, migration, adhesion and attachment, inflammatory response to CNS injury, regulation of vascular inflammation and adaptive responses of the heart to pressure overload and in myocardial function and remodeling. Binds to structural extracellular matrix (ECM) proteins and modulates the ECM in response to tissue damage, contributing to cardioprotective and adaptive ECM remodeling. Plays a role in ER stress response, via its [...]
256	TMED2	9606.ENSP00000262225	TMED2	Transmembrane emp24 domain-containing protein 2; Involved in vesicular protein trafficking. Mainly functions in the early secretory pathway but also in post-Golgi membranes. Thought to act as cargo receptor at the lumenal side for incorporation of secretory cargo molecules into transport vesicles and to be involved in vesicle coat formation at the cytoplasmic side. In COPII vesicle-mediated anterograde transport involved in the transport of GPI-anchored proteins and proposed to act together with TMED10 as their cargo receptor; the function specifically implies SEC24C and SEC24D of the [...]
257	TMX1	9606.ENSP00000393316	TMX1	Thioredoxin-related transmembrane protein 1; May participate in various redox reactions through the reversible oxidation of its active center dithiol to a disulfide and catalyze dithiol-disulfide exchange reactions; Protein disulfide isomerases
258	TNS2	9606.ENSP00000319756	TENC1	Tensin-2; Regulates cell motility and proliferation. May have phosphatase activity. Reduces AKT1 phosphorylation. Lowers AKT1 kinase activity and interferes with AKT1 signaling
259	TP53TG3D	9606.ENSP00000455596	TP53TG3D	TP53 target 3D; May play a significant role in p53/TP53-mediating signaling pathway
260	TRAM2	9606.ENSP00000182527	TRAM2	Translocating chain-associated membrane protein 2; Necessary for collagen type I synthesis. May couple the activity of the ER Ca(2+) pump SERCA2B with the activity of the translocon. This coupling may increase the local Ca(2+) concentration at the site of collagen synthesis, and a high Ca(2+) concentration may be necessary for the function of molecular chaperones involved in collagen folding. Required for proper insertion of the first transmembrane helix N-terminus of TM4SF20 into the ER lumen, may act as a ceramide sensor for regulated alternative translocation (RAT); TLC domain containing
261	TRIB2	9606.ENSP00000155926	TRIB2	Tribbles homolog 2; Interacts with MAPK kinases and regulates activation of MAP kinases. Does not display kinase activity (By similarity); Belongs to the protein kinase superfamily. CAMK Ser/Thr protein kinase family. Tribbles subfamily
262	TRIM35	9606.ENSP00000301924	TRIM35	Tripartite motif-containing protein 35; Reduces FGFR1-dependent tyrosine phosphorylation of PKM, inhibiting PKM-dependent lactate production, glucose metabolism, and cell growth. Involved in the cell death mechanism (By similarity); Ring finger proteins
263	TSLP	9606.ENSP00000339804	TSLP	Thymic stromal lymphopoietin; Isoform 1: Cytokine that induces the release of T-cell- attracting chemokines from monocytes and, in particular, enhances the maturation of CD11c(+) dendritic cells. Can induce allergic inflammation by directly activating mast cells
264	TSN	9606.ENSP00000374332	TSN	Translin; DNA-binding protein that specifically recognizes consensus sequences at the breakpoint junctions in chromosomal translocations, mostly involving immunoglobulin (Ig)/T-cell receptor gene segments. Seems to recognize single-stranded DNA ends generated by staggered breaks occurring at recombination hot spots; Belongs to the translin family
265	TTPA	9606.ENSP00000260116	TTPA	Alpha-tocopherol transfer protein; Binds alpha-tocopherol, enhances its transfer between separate membranes, and stimulates its release from liver cells. Binds both phosphatidylinol 3,4-bisphosphate and phosphatidylinol 4,5-bisphosphate; the resulting conformation change is important for the release of the bound alpha-tocopherol (By similarity)
266	TWSG1	9606.ENSP00000262120	TWSG1	Twisted gastrulation protein homolog 1; May be involved in dorsoventral axis formation. Seems to antagonize BMP signaling by forming ternary complexes with CHRD and BMPs, thereby preventing BMPs from binding to their receptors. In addition to the anti-BMP function, also has pro-BMP activity, partly mediated by cleavage and degradation of CHRD, which releases BMPs from ternary complexes. May be an important modulator of BMP-regulated cartilage development and chondrocyte differentiation. May play a role in thymocyte development (By similarity)
267	UCK2	9606.ENSP00000356853	UCK2	Uridine-cytidine kinase 2; Phosphorylates uridine and cytidine to uridine monophosphate and cytidine monophosphate. Does not phosphorylate deoxyribonucleosides or purine ribonucleosides. Can use ATP or GTP as a phosphate donor. Can also phosphorylate cytidine and uridine nucleoside analogs such as 6-azauridine, 5-fluorouridine, 4- thiouridine, 5-bromouridine, N(4)-acetylcytidine, N(4)-benzoylcytidine, 5-fluorocytidine, 2-thiocytidine, 5- methylcytidine, and N(4)-anisoylcytidine; Belongs to the uridine kinase family
268	UNCX	9606.ENSP00000314480	UNCX	Homeobox protein unc-4 homolog; Transcription factor involved in somitogenesis and neurogenesis. Required for the maintenance and differentiation of particular elements of the axial skeleton. May act upstream of PAX9. Plays a role in controlling the development of connections of hypothalamic neurons to pituitary elements, allowing central neurons to reach the peripheral blood circulation and to deliver hormones for control of peripheral functions (By similarity); PRD class homeoboxes and pseudogenes
269	USP39	9606.ENSP00000312981	USP39	U4/U6.U5 tri-snRNP-associated protein 2; Plays a role in pre-mRNA splicing as a component of the U4/U6-U5 tri-snRNP, one of the building blocks of the spliceosome. Regulates AURKB mRNA levels, and thereby plays a role in cytokinesis and in the spindle checkpoint. Does not have ubiquitin-specific peptidase activity, but could be a competitor of ubiquitin C-terminal hydrolases (UCHs)

270	VRK1	9606.ENSP00000216639	VRK1	Serine/threonine-protein kinase VRK1; Serine/threonine kinase involved in Golgi disassembly during the cell cycle: following phosphorylation by PLK3 during mitosis, required to induce Golgi fragmentation. Acts by mediating phosphorylation of downstream target protein. Phosphorylates 'Thr- 18' of p53/TP53 and may thereby prevent the interaction between p53/TP53 and MDM2. Phosphorylates casein and histone H3. Phosphorylates BANF1: disrupts its ability to bind DNA, reduces its binding to LEM domain-containing proteins and causes its relocalization from the nucleus to the cytoplasm. Phosph [...]
271	VRK3	9606.ENSP00000469880	VRK3	Inactive serine/threonine-protein kinase VRK3; Inactive kinase that suppresses ERK activity by promoting phosphatase activity of DUSP3 which specifically dephosphorylates and inactivates ERK in the nucleus
272	VWA3B	9606.ENSP00000417955	VWA3B	Von Willebrand factor A domain containing 3B
273	VWA5B2	9606.ENSP00000398688	VWA5B2	Von Willebrand factor A domain containing 5B2
274	YAP1	9606.ENSP00000478927	YAP1	Transcriptional coactivator YAP1; Transcriptional regulator which can act both as a coactivator and a corepressor and is the critical downstream regulatory target in the Hippo signaling pathway that plays a pivotal role in organ size control and tumor suppression by restricting proliferation and promoting apoptosis. The core of this pathway is composed of a kinase cascade wherein STK3/MST2 and STK4/MST1, in complex with its regulatory protein SAV1, phosphorylates and activates LATS1/2 in complex with its regulatory protein MOB1, which in turn phosphorylates and inactivates YAP1 oncopro [...]
275	YTHDF1	9606.ENSP00000359364	YTHDF1	YTH domain-containing family protein 1; Specifically recognizes and binds N6-methyladenosine (m6A)-containing mRNAs, and promotes mRNA translation efficiency. M6A is a modification present at internal sites of mRNAs and some non- coding RNAs and plays a role in the efficiency of mRNA splicing, processing and stability. Acts as a regulator of mRNA translation efficiency: promotes ribosome loading to m6A- containing mRNAs and interacts with translation initiation factors eIF3 (EIF3A or EIF3B) to facilitate translation initiation
276	ZBTB10	9606.ENSP00000387462	ZBTB10	Zinc finger and BTB domain-containing protein 10; May be involved in transcriptional regulation; BTB domain containing
277	ZBTB33	9606.ENSP00000314153	ZBTB33	Transcriptional regulator Kaiso; Transcriptional regulator with bimodal DNA-binding specificity. Binds to methylated CpG dinucleotides in the consensus sequence 5'-CGCG-3' and also binds to the non-methylated consensus sequence 5'-CTGCNA-3' also known as the consensus kaiso binding site (KBS). Recruits the N-CoR repressor complex to promote histone deacetylation and the formation of repressive chromatin structures in target gene promoters. May contribute to the repression of target genes of the Wnt signaling pathway. May also activate transcription of a subset of target genes by the re [...]
278	ZC3HC1	9606.ENSP00000351052	ZC3HC1	Nuclear-interacting partner of ALK; Essential component of a SCF-type E3 ligase complex, SCF(NIPA), a complex that controls mitotic entry by mediating ubiquitination and subsequent degradation of cyclin B1 (CCNB1). Its cell-cycle-dependent phosphorylation regulates the assembly of the SCF(NIPA) complex, restricting CCNB1 ubiquitination activity to interphase. Its inactivation results in nuclear accumulation of CCNB1 in interphase and premature mitotic entry. May have an antiapoptotic role in NPM-ALK-mediated signaling events; Zinc fingers
279	ZFHX3	9606.ENSP00000268489	ZFHX3	Zinc finger homeobox protein 3; Transcriptional regulator which can act as an activator or a repressor. Inhibits the enhancer element of the AFP gene by binding to its AT-rich core sequence. In concert with SMAD- dependent TGF-beta signaling can repress the transcription of AFP via its interaction with SMAD2/3. Regulates the circadian locomotor rhythms via transcriptional activation of neuropeptidergic genes which are essential for intercellular synchrony and rhythm amplitude in the suprachiasmatic nucleus (SCN) of the brain (By similarity). Regulator of myoblasts differentiation throu [...]
280	ZFYVE28	9606.ENSP00000290974	ZFYVE28	Lateral signaling target protein 2 homolog; Negative regulator of epidermal growth factor receptor (EGFR) signaling. Acts by promoting EGFR degradation in endosomes when not monoubiquitinated; Belongs to the lst-2 family
281	ZNF337	9606.ENSP00000365619	ZNF337	Zinc finger protein 337; May be involved in transcriptional regulation; Belongs to the krueppel C2H2-type zinc-finger protein family
282	ZNF469	9606.ENSP00000402343	ZNF469	Zinc finger protein 469; May be involved in transcriptional regulation; Belongs to the krueppel C2H2-type zinc-finger protein family
283	ZNF48	9606.ENSP00000480262	ZNF48	Zinc finger protein 48; May be involved in transcriptional regulation; Zinc fingers C2H2-type
284	ZNF571	9606.ENSP00000333660	ZNF571	Zinc finger protein 571; May be involved in transcriptional regulation; Zinc fingers C2H2-type
285	ZNF766	9606.ENSP00000409652	ZNF766	Zinc finger protein 766; May be involved in transcriptional regulation; Zinc fingers C2H2-type
286	ZNF771	9606.ENSP00000323945	ZNF771	Zinc finger protein 771; May be involved in transcriptional regulation; Zinc fingers C2H2-type
287	ZNF786	9606.ENSP00000417470	ZNF786	Zinc finger protein 786; May be involved in transcriptional regulation; Zinc fingers C2H2-type
288	ZNF81	9606.ENSP00000366153	ZNF81	Zinc finger protein 81; May be involved in transcriptional regulation; X-linked mental retardation
289	ZNRF2	9606.ENSP00000323879	ZNRF2	E3 ubiquitin-protein ligase ZNRF2; May play a role in the establishment and maintenance of neuronal transmission and plasticity via its ubiquitin ligase activity. E3 ubiquitin ligases accept ubiquitin from an E2 ubiquitin-conjugating enzyme in the form of a thioester and then directly transfer the ubiquitin to targeted substrates; Ring finger proteins
290	ZSCAN10	9606.ENSP00000252463	ZSCAN10	Zinc finger and SCAN domain-containing protein 10; Embryonic stem (ES) cell-specific transcription factor required to maintain ES cell pluripotency. Can both activate and /or repress expression of target genes, depending on the context. Specifically binds the 5'-[GA]CGCNNGCG[CT]-3' DNA consensus sequence. Regulates expression of POU5F1/OCT4, ZSCAN4 and ALYREF/THOC4 (By similarity); SCAN domain containing
291	ZSCAN26	9606.ENSP00000484931	ZSCAN26	Zinc finger and SCAN domain-containing protein 26; May be involved in transcriptional regulation; SCAN domain containing

Social Acknowledgement (SAQ)

Association with treatment

Gene list

ABTB2, ACTG1, ADCY6, ADD1, ADGRD1, AFAP1, AFDN, AK4, ALCAM, ALDOA, ALOX12, AMER3, AMIGO3, AP3S2, ARHGAP10, ARHGAP17, ARSJ, ARTN, ASXL2, ATF6B, ATL2, ATP6V1D, ATP8B2, BAIAP2L1, BANK1, BARHL2, BAZ1B, BOLA3, BRSK2, C18orf25, C19orf12, C1orf87, C2orf81, C9orf47, C9orf92, CA2, CA8, CACNA1C, CACNA2D2, CBX4, CCDC160, CCDC33, CCDC85C, CCDC88C, CCNG1, CD47, CD81, CDCP1, CDH5, CDHR2, CEBPB, CELF1, CENPH, CEP170B, CFAP46, CFAP47, CHID1, CHRNA10, CHSY3, CLUH, COL9A1, COX6B2, CPD, CPLX2, CS, CSF1R, CUX1, CYP11A1, DCDC2, DGKK, DGKZ, DHX37, DICER1, DIO2, DKC1, DLGAP4, DNAH6, DNAJC2, DNAL4, DPF3, DPH5, DUSP12, EFCAB2, EGFR, EHD2, ELL2P3, ELP6, EPSTI1, ETV6, EVI5, EYA1, FAAH, FAM133A, FAM135B, FAM167B, FAM185A, FAM8A6P, FBLN7, FBXW2, FCRL5, FCRLB, FEZ2, FEZF2, FGF3, FGFRL1, FLYWCH1, FMO5, GATA4, GATAD2A, GBP1, GCNA, GK, GNAQ, GNAS, GPATCH4, GPBP1, GPC1, GPC6, GPIHBP1, GPX5, GRIA3, GRID1, GRIFIN, GRM4, GSDME, HDAC4, HNRNPH2, HOXB2, HPCAL1, HSPG2, HTRA3, HUNK, IDH3G, IGHV1-2, IGSF21, IL12A, IL20RB, IL22, INPP5A, INTU, IRAK2, IRF2BP1, IRF8, IRX4, ITFG2, ITGB2, ITGB8, KCTD3, KDELR3, KIAA1549L, KIF20B, KIF26A, KIFC1, KNDC1, L1TD1, LBX1, LHX1, LIPI, LRATD2, LRP8, LRRC28, LRRC37B, LRRC58, LUZP4, LY6H, MAGT1, MAP2K4, MAPKAP1, MBL2, MED13L, MED21, MEF2D, MICALL2, MICU3, MIPOL1, MIR4275, MKLN1, MMS22L, MOCOS, MORC4, MTRNR2L1, MUC6, MYO5A, NEIL3, NKX1-1, NKX2-3, NKX2-5, NLRP8, NMD3, NOP58, NPRL3, NR4A1, NRP2, OSBPL3, OSR1, PALM2, PAPLN, PAQR5, PCBD2, PCDHB6, PCDHGA4, PCNX4, PDHX, PDIA2, PDZK1IP1, PLD2, PNPLA7, POLD2, POLG, PPFIA4, PPME1, PPP2R2A, PRDM7, PRKD1, PRR16, PTPRN2, RAD52, RAPGEF5, RELN, RGL1, RHOU, RHPN2, RIMS4, RIPOR2, RPL7P40, RPS2P32, S1PR1, SAMD5, SATB2, SDHAF3, SEZ6, SGCE, SHANK2, SIX1, SIX2, SLC17A8, SLC2A1, SLC35A5, SLC9A3, SLCO1A2, SLIT1, SLITRK6, SMNDC1, SMOC2, SOCS6, SORBS2, SOWAHC, SP9, SPATA31D1, SPIB, ST8SIA6, STARD7, STK33, STXBP2, SUPT3H, SYT13, SYT7, TAGLN, TBC1D22A, TBK1, TBX15, TCEAL8, TECR, THBS2, TLE7, TMEM132C, TMEM167B, TMEM170B, TMEM217, TMEM88B, TMSB15B, TNXB, TPI1, TRA, TRARG1, TRERF1, TRIB1, TRIM62, TRMT10C, TRS-AGA1-1, TSC22D3, TSKU, TTC28, TTC34, TTC9, TTC9B, WDPCP, WDR11, WLS, WSCD1, XPNPEP1, XPNPEP2, ZBTB8A, ZNF184, ZNF263, ZNF266, ZNF507, ZNF724, ZNF821

Network

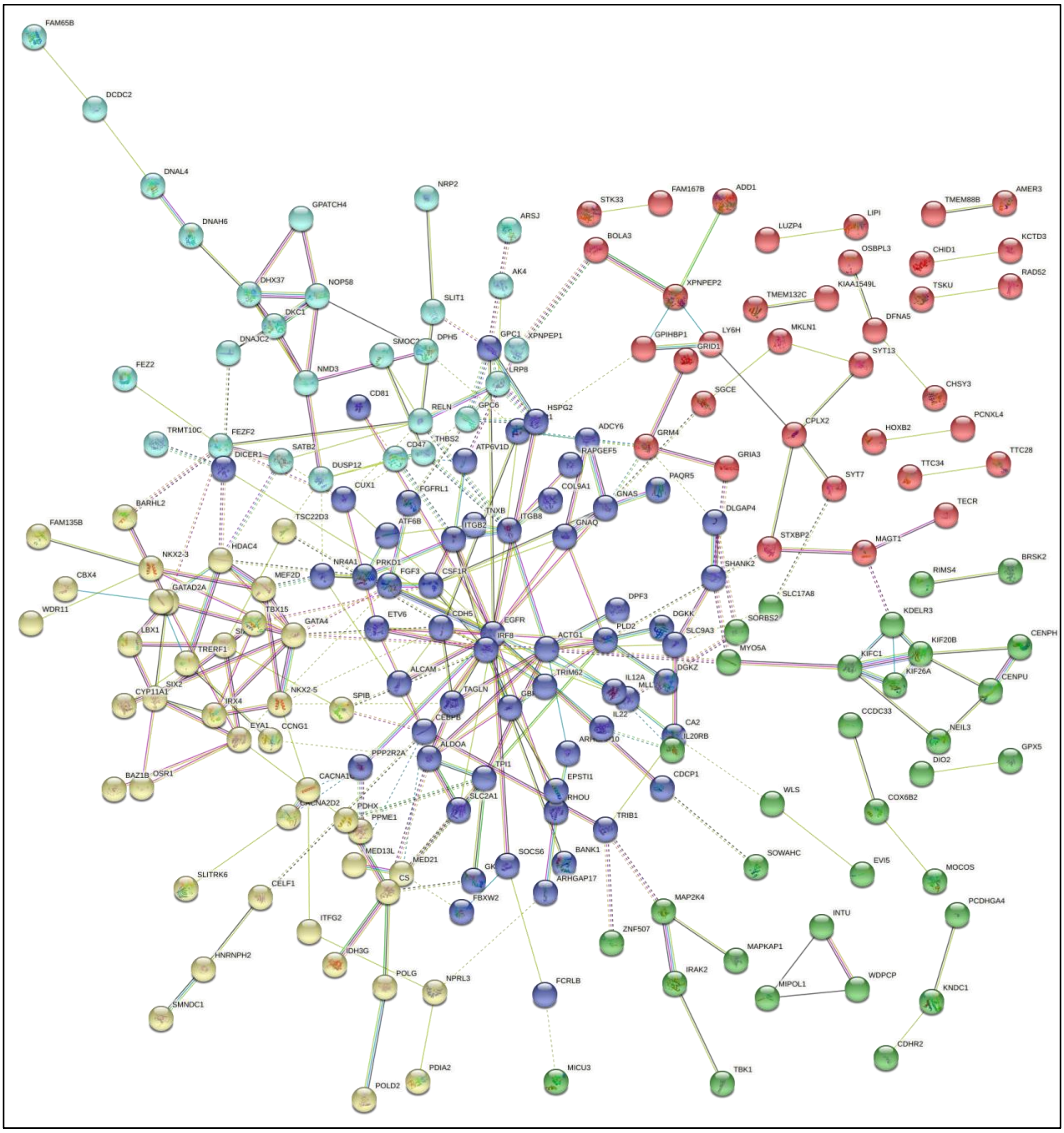

Treatment

Enrichments found

Network Stats

number of nodes:	289	expected number of edges:	244
number of edges:	298	PPI enrichment p-value:	0.000464
average node degree:	2.06	*your network has significantly more interactions than expected (what does that mean?)*	
avg. local clustering coefficient:	0.307		

Functional enrichments in your network

explain columns

Enrichment Table Columns

Count In Network:
The first number indicates how many proteins in your network are annotated with a particular term. The second number indicates how many proteins in total (in your network and in the background) have this term assigned.

Strength:
Log10(observed / expected). This measure describes how large the enrichment effect is. It's the ratio between i) the number of proteins in your network that are annotated with a term and ii) the number of proteins that we expect to be annotated with this term in a random network of the same size.

False Discovery Rate:
This measure describes how significant the enrichment is. Shown are p-values corrected for multiple testing within each category using the Benjamini–Hochberg procedure.

Biological Process (Gene Ontology)

GO-term	description	count in network	strength	false discovery rate
GO:0055017	cardiac muscle tissue growth	5 of 26	1.11	0.0425
GO:0048704	embryonic skeletal system morphogenesis	9 of 92	0.82	0.0422
GO:0048562	embryonic organ morphogenesis	15 of 279	0.56	0.0422
GO:0048598	embryonic morphogenesis	22 of 545	0.44	0.0422
GO:0009887	animal organ morphogenesis	30 of 865	0.37	0.0422
GO:0009653	anatomical structure morphogenesis	54 of 1992	0.26	0.0422
GO:0007399	nervous system development	56 of 2206	0.24	0.0422

(less ...)

Cellular Component (Gene Ontology)

GO-term	description	count in network	strength	false discovery rate
GO:0070062	extracellular exosome	8 of 80	0.83	0.0197

KEGG Pathways

pathway	description	count in network	strength	false discovery rate
hsa04927	Cortisol synthesis and secretion	7 of 63	0.88	0.0147
hsa04925	Aldosterone synthesis and secretion	8 of 93	0.77	0.0147
hsa04512	ECM-receptor interaction	7 of 81	0.77	0.0155
hsa04976	Bile secretion	6 of 71	0.76	0.0268
hsa04724	Glutamatergic synapse	9 of 112	0.74	0.0147
hsa04912	GnRH signaling pathway	7 of 88	0.73	0.0165
hsa04015	Rap1 signaling pathway	11 of 203	0.56	0.0155

(less ...)

Treatment

Mapping

#	queryItem	stringId	preferredName	annotation
1	ABTB2	9606.ENSP00000410157	ABTB2	Ankyrin repeat and BTB/POZ domain-containing protein 2; May be involved in the initiation of hepatocyte growth; Ankyrin repeat domain containing
2	ACTG1	9606.ENSP00000458162	ACTG1	Actin, cytoplasmic 2; Actins are highly conserved proteins that are involved in various types of cell motility and are ubiquitously expressed in all eukaryotic cells; Belongs to the actin family
3	ADCY6	9606.ENSP00000311405	ADCY6	Adenylate cyclase type 6; Catalyzes the formation of the signaling molecule cAMP downstream of G protein-coupled receptors. Functions in signaling cascades downstream of beta-adrenergic receptors in the heart and in vascular smooth muscle cells. Functions in signaling cascades downstream of the vasopressin receptor in the kidney and has a role in renal water reabsorption. Functions in signaling cascades downstream of PTH1R and plays a role in regulating renal phosphate excretion. Functions in signaling cascades downstream of the VIP and SCT receptors in pancreas and contributes to the [...]
4	ADD1	9606.ENSP00000264758	ADD1	Alpha-adducin; Membrane-cytoskeleton-associated protein that promotes the assembly of the spectrin-actin network. Binds to calmodulin
5	ADGRD1	9606.ENSP00000261654	GPR133	Adhesion G-protein coupled receptor D1; Orphan receptor. Signals via G(s)-alpha family of G- proteins. Has protumorigenic function especially in glioblastoma
6	AFAP1	9606.ENSP00000410689	AFAP1	Actin filament-associated protein 1; Can cross-link actin filaments into both network and bundle structures (By similarity). May modulate changes in actin filament integrity and induce lamellipodia formation. May function as an adapter molecule that links other proteins, such as SRC and PKC to the actin cytoskeleton. Seems to play a role in the development and progression of prostate adenocarcinoma by regulating cell-matrix adhesions and migration in the cancer cells; Pleckstrin homology domain containing
7	AFDN	9606.ENSP00000375960	MLLT4	Afadin; Belongs to an adhesion system, probably together with the E-cadherin-catenin system, which plays a role in the organization of homotypic, interneuronal and heterotypic cell-cell adherens junctions (AJs). Nectin- and actin-filament-binding protein that connects nectin to the actin cytoskeleton
8	AK4	9606.ENSP00000378743	AK4	Adenylate kinase 4, mitochondrial; Involved in maintaining the homeostasis of cellular nucleotides by catalyzing the interconversion of nucleoside phosphates. Efficiently phosphorylates AMP and dAMP using ATP as phosphate donor, but phosphorylates only AMP when using GTP as phosphate donor. Also displays broad nucleoside diphosphate kinase activity; Belongs to the adenylate kinase family. AK3 subfamily
9	ALCAM	9606.ENSP00000305988	ALCAM	CD166 antigen; Cell adhesion molecule that mediates both heterotypic cell-cell contacts via its interaction with CD6, as well as homotypic cell-cell contacts. Promotes T-cell activation and proliferation via its interactions with CD6. Contributes to the formation and maturation of the immunological synapse via its interactions with CD6. Mediates homotypic interactions with cells that express ALCAM. Required for normal hematopoietic stem cell engraftment in the bone marrow. Mediates attachment of dendritic cells onto endothelial cells via homotypic interaction. Inhibits endothelial cell [...]
10	ALDOA	9606.ENSP00000378669	ALDOA	Fructose-bisphosphate aldolase A; Plays a key role in glycolysis and gluconeogenesis. In addition, may also function as scaffolding protein (By similarity); Belongs to the class I fructose-bisphosphate aldolase family
11	ALOX12	9606.ENSP00000251535	ALOX12	Arachidonate 12-lipoxygenase, 12S-type; Non-heme iron-containing dioxygenase that catalyzes the stereo-specific peroxidation of free and esterified polyunsaturated fatty acids generating a spectrum of bioactive lipid mediators. Mainly converts arachidonic acid to (12S)-hydroperoxyeicosatetraenoic acid/(12S)-HPETE but can also metabolize linoleic acid. Has a dual activity since it also converts leukotriene A4/LTA4 into both the bioactive lipoxin A4/LXA4 and lipoxin B4/LXB4. Through the production of specific bioactive lipids like (12S)-HPETE it regulates different biological processes [...]
12	AMER3	9606.ENSP00000392700	AMER3	APC membrane recruitment protein 3; Regulator of the canonical Wnt signaling pathway. Acts by specifically binding phosphatidylinositol 4,5-bisphosphate (PtdIns(4,5)P2), translocating to the cell membrane (By similarity)
13	AMIGO3	9606.ENSP00000323096	AMIGO3	Amphoterin-induced protein 3; May mediate heterophilic cell-cell interaction. May contribute to signal transduction through its intracellular domain (By similarity); Immunoglobulin like domain containing
14	AP3S2	9606.ENSP00000338777	AP3S2	AP-3 complex subunit sigma-2; Part of the AP-3 complex, an adaptor-related complex which is not clathrin-associated. The complex is associated with the Golgi region as well as more peripheral structures. It facilitates the budding of vesicles from the Golgi membrane and may be directly involved in trafficking to lysosomes. In concert with the BLOC-1 complex, AP-3 is required to target cargos into vesicles assembled at cell bodies for delivery into neurites and nerve terminals
15	ARHGAP10	9606.ENSP00000336923	ARHGAP10	Rho GTPase-activating protein 10; GTPase activator for the small GTPases RhoA and Cdc42 by converting them to an inactive GDP-bound state. Essential for PTKB2 regulation of cytoskeletal organization via Rho family GTPases. Inhibits PAK2 proteolytic fragment PAK-2p34 kinase activity and changes its localization from the nucleus to the perinuclear region. Stabilizes PAK-2p34 thereby increasing stimulation of cell death (By similarity)
16	ARHGAP17	9606.ENSP00000289968	ARHGAP17	Rho GTPase-activating protein 17; Rho GTPase-activating protein involved in the maintenance of tight junction by regulating the activity of CDC42, thereby playing a central role in apical polarity of epithelial cells. Specifically acts as a GTPase activator for the CDC42 GTPase by converting it to an inactive GDP-bound state. The complex formed with AMOT acts by regulating the uptake of polarity proteins at tight junctions, possibly by deciding whether tight junction transmembrane proteins are recycled back to the plasma membrane or sent elsewhere. Participates in the Ca(2+)-dependent [...]
17	ARSJ	9606.ENSP00000320219	ARSJ	Arylsulfatase family member J; Sulfatases
18	ARTN	9606.ENSP00000387435	ARTN	Artemin; Ligand for the GFR-alpha-3-RET receptor complex but can also activate the GFR-alpha-1-RET receptor complex. Supports the survival of sensory and sympathetic peripheral neurons in culture and also supports the survival of dopaminergic neurons of the ventral mid-brain. Strong attractant of gut hematopoietic cells thus promoting the formation Peyer's patch-like structures, a major component of the gut-associated lymphoid tissue; GDNF family ligands
19	ASXL2	9606.ENSP00000383920	ASXL2	Putative Polycomb group protein ASXL2; Putative Polycomb group (PcG) protein. PcG proteins act by forming multiprotein complexes, which are required to maintain the transcriptionally repressive state of homeotic genes throughout development. PcG proteins are not required to initiate repression, but to maintain it during later stages of development. They probably act via methylation of histones, rendering chromatin heritably changed in its expressibility (By similarity). Involved in transcriptional regulation mediated by ligand-bound nuclear hormone receptors, such as peroxisome prolife [...]
20	ATF6B	9606.ENSP00000364349	ATF6B	Cyclic AMP-dependent transcription factor ATF-6 beta; Transcriptional factor that acts in the unfolded protein response (UPR) pathway by activating UPR target genes induced during ER stress. Binds DNA on the 5'-CCAC[GA]-3' half of the ER stress response element (ERSE) (5'-CCAATN(9)CCAC[GA]-3') when NF-Y is bound to ERSE; Basic leucine zipper proteins
21	ATL2	9606.ENSP00000368237	ATL2	Atlastin-2; GTPase tethering membranes through formation of trans- homooligomers and mediating homotypic fusion of endoplasmic reticulum membranes. Functions in endoplasmic reticulum tubular network biogenesis; Belongs to the TRAFAC class dynamin-like GTPase superfamily. GB1/RHD3-type GTPase family. GB1 subfamily
22	ATP6V1D	9606.ENSP00000216442	ATP6V1D	V-type proton ATPase subunit D; Subunit of the peripheral V1 complex of vacuolar ATPase. Vacuolar ATPase is responsible for acidifying a variety of intracellular compartments in eukaryotic cells, thus providing most of the energy required for transport processes in the vacuolar system (By similarity). May play a role in cilium biogenesis through regulation of the transport and the localization of proteins to the cilium
23	ATP8B2	9606.ENSP00000357475	ATP8B2	Phospholipid-transporting ATPase ID; Catalytic component of a P4-ATPase flippase complex which catalyzes the hydrolysis of ATP coupled to the transport of aminophospholipids from the outer to the inner leaflet of various membranes and ensures the maintenance of asymmetric distribution of phospholipids. Phospholipid translocation seems also to be implicated in vesicle formation and in uptake of lipid signaling molecules (Probable); Belongs to the cation transport ATPase (P-type) (TC 3.A.3) family. Type IV subfamily
24	BAIAP2L1	9606.ENSP00000005260	BAIAP2L1	Brain-specific angiogenesis inhibitor 1-associated protein 2-like protein 1; May function as adapter protein. Involved in the formation of clusters of actin bundles. Plays a role in the reorganization of the actin cytoskeleton in response to bacterial infection; I-BAR domain containing
25	BANK1	9606.ENSP00000320509	BANK1	B-cell scaffold protein with ankyrin repeats; Involved in B-cell receptor (BCR)-induced Ca(2+) mobilization from intracellular stores. Promotes Lyn-mediated phosphorylation of IP3 receptors 1 and 2; Ankyrin repeat domain containing
26	BARHL2	9606.ENSP00000359474	BARHL2	BarH-like 2 homeobox protein; Potential regulator of neural basic helix-loop-helix genes; NKL subclass homeoboxes and pseudogenes
27	BAZ1B	9606.ENSP00000342434	BAZ1B	Tyrosine-protein kinase BAZ1B; Atypical tyrosine-protein kinase that plays a central role in chromatin remodeling and acts as a transcription regulator. Involved in DNA damage response by phosphorylating 'Tyr-142' of histone H2AX (H2AXY142ph). H2AXY142ph plays a central role in DNA repair and acts as a mark that distinguishes between apoptotic and repair responses to genotoxic stress. Essential component of the WICH complex, a chromatin remodeling complex that mobilizes nucleosomes and reconfigures irregular chromatin to a regular nucleosomal array structure. The WICH complex regulates [...]
28	BOLA3	9606.ENSP00000331369	BOLA3	BolA-like protein 3; Acts as a mitochondrial iron-sulfur (Fe-S) cluster assembly factor that facilitates (Fe-S) cluster insertion into a subset of mitochondrial proteins. Probably acts together with NFU1; Belongs to the BolA/IbaG family
29	BRSK2	9606.ENSP00000371614	BRSK2	Serine/threonine-protein kinase BRSK2; Serine/threonine-protein kinase that plays a key role in polarization of neurons and axonogenesis, cell cycle progress and insulin secretion. Phosphorylates CDK16, CDC25C, MAPT/TAU, PAK1 and WEE1. Following phosphorylation and activation by STK11/LKB1, acts as a key regulator of polarization of cortical neurons, probably by mediating phosphorylation of microtubule-associated proteins such as MAPT/TAU at 'Thr-529' and 'Ser-579'. Also regulates neuron polarization by mediating phosphorylation of WEE1 at 'Ser-642' in postmitotic neurons, leading to d [...]
30	C18orf25	9606.ENSP00000481626	C18orf25	Uncharacterized protein C18orf25; Chromosome 18 open reading frame 25
31	C19orf12	9606.ENSP00000376103	C19orf12	Protein C19orf12; Chromosome 19 open reading frame 12
32	C1orf87	9606.ENSP00000360244	C1orf87	Uncharacterized protein C1orf87; Chromosome 1 open reading frame 87
33	C2orf81	9606.ENSP00000290390	C2orf81	Uncharacterized protein C2orf81; Chromosome 2 open reading frame 81
34	C9orf47	9606.ENSP00000335616	C9orf47	Uncharacterized protein C9orf47; Chromosome 9 open reading frame 47
35	C9orf92	9606.ENSP00000370058	C9orf92	Putative uncharacterized protein C9orf92; Chromosome 9 open reading frame 92
36	CA2	9606.ENSP00000285379	CA2	Carbonic anhydrase 2; Essential for bone resorption and osteoclast differentiation (By similarity). Reversible hydration of carbon dioxide. Can hydrate cyanamide to urea. Involved in the regulation of fluid secretion into the anterior chamber of the eye. Contributes to intracellular pH regulation in the duodenal upper villous epithelium during proton-coupled peptide absorption. Stimulates the chloride-bicarbonate exchange activity of SLC26A6; Carbonic anhydrases
37	CA8	9606.ENSP00000314407	CA8	Carbonic anhydrase-related protein; Does not have a carbonic anhydrase catalytic activity
38	CACNA1C	9606.ENSP00000266376	CACNA1C	Voltage-dependent L-type calcium channel subunit alpha-1C; Voltage-sensitive calcium channels (VSCC) mediate the entry of calcium ions into excitable cells and are also involved in a variety of calcium-dependent processes, including muscle contraction, hormone or neurotransmitter release, gene expression, cell motility, cell division and cell death. The isoform alpha-1C gives rise to L-type calcium currents. Long-lasting (L-type) calcium channels belong to the 'high-voltage activated' (HVA) group. They are blocked by dihydropyridines (DHP), phenylalkylamines, benzothiazepines, and by o [...]
39	CACNA2D2	9606.ENSP00000418081	CACNA2D2	Voltage-dependent calcium channel subunit alpha-2/delta-2; The alpha-2/delta subunit of voltage-dependent calcium channels regulates calcium current density and activation/inactivation kinetics of the calcium channel. Acts as a regulatory subunit for P/Q-type calcium channel (CACNA1A), N-type (CACNA1B), L-type (CACNA1C OR CACNA1D) and possibly T-type (CACNA1G). Overexpression induces apoptosis
40	CBX4	9606.ENSP00000269397	CBX4	E3 SUMO-protein ligase CBX4; E3 SUMO-protein ligase which facilitates SUMO1 conjugation by UBE2I. Involved in the sumoylation of HNRNPK, a p53/TP53 transcriptional coactivator, hence indirectly regulates p53/TP53 transcriptional activation resulting in p21/CDKN1A expression. Monosumoylates ZNF131; Chromobox family
41	CCDC160	9606.ENSP00000427951	CCDC160	Coiled-coil domain containing 160; Belongs to the CCDC160 family
42	CCDC33	9606.ENSP00000381795	CCDC33	Coiled-coil domain containing 33
43	CCDC85C	9606.ENSP00000369592	CCDC85C	Coiled-coil domain-containing protein 85C; May play an important role in cortical development, especially in the maintenance of radial glia
44	CCDC88C	9606.ENSP00000374507	CCDC88C	Protein Daple; Negative regulator of the canonical Wnt signaling pathway, acting downstream of DVL to inhibit CTNNB1/Beta-catenin stabilization (By similarity). May also activate the JNK signaling pathway; Belongs to the CCDC88 family
45	CCNG1	9606.ENSP00000344635	CCNG1	Cyclin-G1; May play a role in growth regulation. Is associated with G2/M phase arrest in response to DNA damage. May be an intermediate by which p53 mediates its role as an inhibitor of cellular proliferation (By similarity); Cyclins
46	CD47	9606.ENSP00000355361	CD47	Leukocyte surface antigen CD47; Has a role in both cell adhesion by acting as an adhesion receptor for THBS1 on platelets, and in the modulation of integrins. Plays an important role in memory formation and synaptic plasticity in the hippocampus (By similarity). Receptor for SIRPA, binding to which prevents maturation of immature dendritic cells and inhibits cytokine production by mature dendritic cells. Interaction with SIRPG

Treatment

#	queryItem	stringId	preferredName	annotation
				mediates cell-cell adhesion, enhances superantigen-dependent T-cell-mediated proliferation and costimulates T-cell activation. May play a role in membrane transp [...]
47	CD81	9606.ENSP00000263645	CD81	CD81 antigen; May play an important role in the regulation of lymphoma cell growth. Interacts with a 16-kDa Leu-13 protein to form a complex possibly involved in signal transduction. May act as the viral receptor for HCV; CD molecules
48	CDCP1	9606.ENSP00000296129	CDCP1	CUB domain-containing protein 1; May be involved in cell adhesion and cell matrix association. May play a role in the regulation of anchorage versus migration or proliferation versus differentiation via its phosphorylation. May be a novel marker for leukemia diagnosis and for immature hematopoietic stem cell subsets. Belongs to the tetraspanin web involved in tumor progression and metastasis; CD molecules
49	CDH5	9606.ENSP00000344115	CDH5	Cadherin-5; Cadherins are calcium-dependent cell adhesion proteins. They preferentially interact with themselves in a homophilic manner in connecting cells; cadherins may thus contribute to the sorting of heterogeneous cell types. This cadherin may play a important role in endothelial cell biology through control of the cohesion and organization of the intercellular junctions. It associates with alpha-catenin forming a link to the cytoskeleton. Acts in concert with KRIT1 to establish and maintain correct endothelial cell polarity and vascular lumen. These effects are mediated by recrui [...]
50	CDHR2	9606.ENSP00000424565	CDHR2	Cadherin-related family member 2; Intermicrovillar adhesion molecule that forms, via its extracellular domain, calcium-dependent heterophilic complexes with CDHR5 on adjacent microvilli. Thereby, controls the packing of microvilli at the apical membrane of epithelial cells. Through its cytoplasmic domain, interacts with microvillus cytoplasmic proteins to form the intermicrovillar adhesion complex/IMAC. This complex plays a central role in microvilli and epithelial brush border differentiation. May also play a role in cell-cell adhesion and contact inhibition in epithelial cells; Cadhe [...]
51	CEBPB	9606.ENSP00000305422	CEBPB	CCAAT/enhancer-binding protein beta; Important transcription factor regulating the expression of genes involved in immune and inflammatory responses. Plays also a significant role in adipogenesis, as well as in the gluconeogenic pathway, liver regeneration, and hematopoiesis. The consensus recognition site is 5'- T[TG]NNGNAA[TG]-3'. Its functional capacity is governed by protein interactions and post-translational protein modifications. During early embryogenesis, plays essential and redundant functions with CEBPA. Has a promitotic effect on many cell types such as hepatocytes and adip [...]
52	CELF1	9606.ENSP00000435926	CELF1	CUGBP Elav-like family member 1; RNA-binding protein implicated in the regulation of several post-transcriptional events. Involved in pre-mRNA alternative splicing, mRNA translation and stability. Mediates exon inclusion and/or exclusion in pre-mRNA that are subject to tissue-specific and developmentally regulated alternative splicing. Specifically activates exon 5 inclusion of cardiac isoforms of TNNT2 during heart remodeling at the juvenile to adult transition. Acts as both an activator and repressor of a pair of coregulated exons: promotes inclusion of the smooth muscle (SM) exon bu [...]
53	CENPH	9606.ENSP00000283006	CENPH	Centromere protein H; Component of the CENPA-NAC (nucleosome-associated) complex, a complex that plays a central role in assembly of kinetochore proteins, mitotic progression and chromosome segregation. The CENPA-NAC complex recruits the CENPA-CAD (nucleosome distal) complex and may be involved in incorporation of newly synthesized CENPA into centromeres. Required for chromosome congression and efficiently align the chromosomes on a metaphase plate
54	CEP170B	9606.ENSP00000404151	CEP170B	Centrosomal protein of 170 kDa protein B; Plays a role in microtubule organization
55	CFAP46	9606.ENSP00000357575	CFAP46	Cilia- and flagella-associated protein 46; As part of the central apparatus of the cilium axoneme plays a role in cilium movement; Cilia and flagella associated
56	CFAP47	9606.ENSP00000297866	CXorf22	Cilia- and flagella-associated protein 47; Chromosome X open reading frame 22
57	CHID1	9606.ENSP00000398722	CHID1	Chitinase domain-containing protein 1; Saccharide- and LPS-binding protein with possible roles in pathogen sensing and endotoxin neutralization. Ligand-binding specificity relates to the length of the oligosaccharides, with preference for chitotetraose (in vitro); Belongs to the glycosyl hydrolase 18 family
58	CHRNA10	9606.ENSP00000250699	CHRNA10	Neuronal acetylcholine receptor subunit alpha-10; Ionotropic receptor with a probable role in the modulation of auditory stimuli. Agonist binding may induce an extensive change in conformation that affects all subunits and leads to opening of an ion-conducting channel across the plasma membrane. The channel is permeable to a range of divalent cations including calcium, the influx of which may activate a potassium current which hyperpolarizes the cell membrane. In the ear, this may lead to a reduction in basilar membrane motion, altering the activity of auditory nerve fibers and reducin [...]
59	CHSY3	9606.ENSP00000302629	CHSY3	Chondroitin sulfate synthase 3; Has both beta-1,3-glucuronic acid and beta-1,4-N- acetylgalactosamine transferase activity. Transfers glucuronic acid (GlcUA) from UDP-GlcUA and N-acetylgalactosamine (GalNAc) from UDP-GalNAc to the non-reducing end of the elongating chondroitin polymer. Specific activity is much reduced compared to CHSY1
60	CLUH	9606.ENSP00000458986	CLUH	Clustered mitochondria protein homolog; mRNA-binding protein involved in proper cytoplasmic distribution of mitochondria. Specifically binds mRNAs of nuclear- encoded mitochondrial proteins in the cytoplasm and regulates transport or translation of these transcripts close to mitochondria, playing a role in mitochondrial biogenesis
61	COL9A1	9606.ENSP00000349790	COL9A1	Collagen alpha-1(IX) chain; Structural component of hyaline cartilage and vitreous of the eye; Collagen proteoglycans
62	COX6B2	9606.ENSP00000467266	COX6B2	Cytochrome c oxidase subunit 6B2; Connects the two COX monomers into the physiological dimeric form; Belongs to the cytochrome c oxidase subunit 6B family
63	CPD	9606.ENSP00000225719	CPD	M14 carboxypeptidases
64	CPLX2	9606.ENSP00000352544	CPLX2	Complexin-2; Negatively regulates the formation of synaptic vesicle clustering at active zone to the presynaptic membrane in postmitotic neurons. Positively regulates a late step in exocytosis of various cytoplasmic vesicles, such as synaptic vesicles and other secretory vesicles. Also involved in mast cell exocytosis (By similarity); Belongs to the complexin/synaphin family
65	CS	9606.ENSP00000342056	CS	Citrate synthase, mitochondrial; Citrate synthase
66	CSF1R	9606.ENSP00000286301	CSF1R	Macrophage colony-stimulating factor 1 receptor; Tyrosine-protein kinase that acts as cell-surface receptor for CSF1 and IL34 and plays an essential role in the regulation of survival, proliferation and differentiation of hematopoietic precursor cells, especially mononuclear phagocytes, such as macrophages and monocytes. Promotes the release of proinflammatory chemokines in response to IL34 and CSF1, and thereby plays an important role in innate immunity and in inflammatory processes. Plays an important role in the regulation of osteoclast proliferation and differentiation, the regulat [...]
67	CUX1	9606.ENSP00000353401	CUX1	Homeobox protein cut-like 1; Probably has a broad role in mammalian development as a repressor of developmentally regulated gene expression. May act by preventing binding of positively-activing CCAAT factors to promoters. Component of nf-munr repressor; binds to the matrix attachment regions (MARs) (5' and 3') of the immunoglobulin heavy chain enhancer. Represses T-cell receptor (TCR) beta enhancer function by binding to MARbeta, an ATC-rich DNA sequence located upstream of the TCR beta enhancer. Binds to the TH enhancer; may require the basic helix-loop-helix protein TCF4 as a coactiv [...]
68	CYP11A1	9606.ENSP00000268053	CYP11A1	Cholesterol side-chain cleavage enzyme, mitochondrial; Catalyzes the side-chain cleavage reaction of cholesterol to pregnenolone; Cytochrome P450 family 11
69	DCDC2	9606.ENSP00000367715	DCDC2	Doublecortin domain-containing protein 2; Protein that plays a role in the inhibition of canonical Wnt signaling pathway. May be involved in neuronal migration during development of the cerebral neocortex (By similarity). Involved in the control of ciliogenesis and ciliary length; Deafness associated genes
70	DGKK	9606.ENSP00000477515	DGKK	Diacylglycerol kinase kappa; Phosphorylates diacylglycerol (DAG) to generate phosphatidic acid (PA)
71	DGKZ	9606.ENSP00000412178	DGKZ	Diacylglycerol kinase zeta; Displays a strong preference for 1,2-diacylglycerols over 1,3-diacylglycerols, but lacks substrate specificity among molecular species of long chain diacylglycerols. Isoform 2 but not isoform 1 regulates RASGRP1 activity; Belongs to the eukaryotic diacylglycerol kinase family
72	DHX37	9606.ENSP00000311135	DHX37	Probable ATP-dependent RNA helicase DHX37; DEAH-box helicase 37
73	DICER1	9606.ENSP00000437256	DICER1	Endoribonuclease Dicer; Double-stranded RNA (dsRNA) endoribonuclease playing a central role in short dsRNA-mediated post-transcriptional gene silencing. Cleaves naturally occurring long dsRNAs and short hairpin pre-microRNAs (miRNA) into fragments of twenty-one to twenty-three nucleotides with 3' overhang of two nucleotides, producing respectively short interfering RNAs (siRNA) and mature microRNAs. SiRNAs and miRNAs serve as guide to direct the RNA- induced silencing complex (RISC) to complementary RNAs to degrade them or prevent their translation. Gene silencing mediated by siRNAs, a [...]
74	DIO2	9606.ENSP00000450980	DIO2	Iodothyronine deiodinase; Responsible for the deiodination of T4 (3,5,3',5'- tetraiodothyronine); Selenoproteins
75	DKC1	9606.ENSP00000358563	DKC1	H/ACA ribonucleoprotein complex subunit 4; Isoform 1: Required for ribosome biogenesis and telomere maintenance. Probable catalytic subunit of H/ACA small nucleolar ribonucleoprotein (H/ACA snoRNP) complex, which catalyzes pseudouridylation of rRNA. This involves the isomerization of uridine such that the ribose is subsequently attached to C5, instead of the normal N1. Each rRNA can contain up to 100 pseudouridine ('psi') residues, which may serve to stabilize the conformation of rRNAs. Also required for correct processing or intranuclear trafficking of TERC, the RNA component of the t [...]
76	DLGAP4	9606.ENSP00000363023	DLGAP4	Disks large-associated protein 4; May play a role in the molecular organization of synapses and neuronal cell signaling. Could be an adapter protein linking ion channel to the subsynaptic cytoskeleton. May induce enrichment of PSD-95/SAP90 at the plasma membrane
77	DNAH6	9606.ENSP00000374045	DNAH6	Dynein heavy chain 6, axonemal; Force generating protein of respiratory cilia. Produces force towards the minus ends of microtubules. Dynein has ATPase activity; the force-producing power stroke is thought to occur on release of ADP (By similarity); Belongs to the dynein heavy chain family
78	DNAJC2	9606.ENSP00000368565	DNAJC2	DnaJ homolog subfamily C member 2; Acts both as a chaperone in the cytosol and as a chromatin regulator in the nucleus. When cytosolic, acts as a molecular chaperone: component of the ribosome-associated complex (RAC), a complex involved in folding or maintaining nascent polypeptides in a folding-competent state. In the RAC complex, stimulates the ATPase activity of the ribosome-associated pool of Hsp70-type chaperones HSPA14 that bind to the nascent polypeptide chain. When nuclear, mediates the switching from polycomb- repressed genes to an active state: specifically recruited at hist [...]
79	DNAL4	9606.ENSP00000216068	DNAL4	Dynein light chain 4, axonemal; Force generating protein of respiratory cilia. Produces force towards the minus ends of microtubules. Dynein has ATPase activity (By similarity); Dyneins, axonemal
80	DPF3	9606.ENSP00000479526	DPF3	Zinc finger protein DPF3; Belongs to the neuron-specific chromatin remodeling complex (nBAF complex). During neural development a switch from a stem/progenitor to a post-mitotic chromatin remodeling mechanism occurs as neurons exit the cell cycle and become committed to their adult state. The transition from proliferating neural stem/progenitor cells to post-mitotic neurons requires a switch in subunit composition of the npBAF and nBAF complexes. As neural progenitors exit mitosis and differentiate into neurons, npBAF complexes which contain ACTL6A/BAF53A and PHF10/BAF45A, are exchange [...]
81	DPH5	9606.ENSP00000359127	DPH5	Diphthine methyl ester synthase; S-adenosyl-L-methionine-dependent methyltransferase that catalyzes four methylations of the modified target histidine residue in translation elongation factor 2 (EF-2), to form an intermediate called diphthine methyl ester. The four successive methylation reactions represent the second step of diphthamide biosynthesis
82	DUSP12	9606.ENSP00000356920	DUSP12	Dual specificity protein phosphatase 12; Dual specificity phosphatase; can dephosphorylate both phosphotyrosine and phosphoserine or phosphothreonine residues. Can dephosphorylate glucokinase (in vitro) (By similarity). Has phosphatase activity with the synthetic substrate 6,8-difluoro-4- methylumbelliferyl phosphate and other in vitro substrates; Belongs to the protein-tyrosine phosphatase family. Non-receptor class dual specificity subfamily
83	EFCAB2	9606.ENSP00000355480	EFCAB2	EF-hand calcium-binding domain-containing protein 2; EF-hand calcium binding domain 2; Cilia and flagella associated
84	EGFR	9606.ENSP00000275493	EGFR	Epidermal growth factor receptor; Receptor tyrosine kinase binding ligands of the EGF family and activating several signaling cascades to convert extracellular cues into appropriate cellular responses. Known ligands include EGF, TGFA/TGF-alpha, amphiregulin, epigen/EPGN, BTC/betacellulin, epiregulin/EREG and HBEGF/heparin-binding EGF. Ligand binding triggers receptor homo- and/or heterodimerization and autophosphorylation on key cytoplasmic residues. The phosphorylated receptor recruits adapter proteins like GRB2 which in turn activates complex downstream signaling cascades. Activates [...]
85	EHD2	9606.ENSP00000263277	EHD2	EH domain-containing protein 2; ATP- and membrane-binding protein that controls membrane reorganization/tubulation upon ATP hydrolysis (By similarity). Plays a role in membrane trafficking between the plasma membrane and endosomes. Important for the internalization of GLUT4. Required for fusion of myoblasts to skeletal muscle myotubes. Required for normal translocation of FER1L5 to the plasma membrane (By similarity). Regulates the equilibrium between cell surface-associated and cell surface-dissociated caveolae by constraining caveolae at the cell membrane; EF-hand domain containing
87	ELP6	9606.ENSP00000296149	ELP6	Elongator complex protein 6; Acts as subunit of the RNA polymerase II elongator complex, which is a histone acetyltransferase component of the RNA polymerase II (Pol II) holoenzyme and is involved in transcriptional elongation. Elongator may play a role in chromatin remodeling and is involved in acetylation of histones H3 and probably H4. Involved in cell migration
88	EPSTI1	9606.ENSP00000318982	EPSTI1	Epithelial stromal interaction 1

Treatment

#	queryItem	stringId	preferredName	annotation
89	ETV6	9606.ENSP00000379658	ETV6	Transcription factor ETV6; Transcriptional repressor; binds to the DNA sequence 5'- CCGGAAGT-3'. Plays a role in hematopoiesis and malignant transformation; Belongs to the ETS family
90	EVI5	9606.ENSP00000359356	EVI5	Ecotropic viral integration site 5 protein homolog; Functions as a regulator of cell cycle progression by stabilizing the FBXO5 protein and promoting cyclin-A accumulation during interphase. May play a role in cytokinesis
91	EYA1	9606.ENSP00000342626	EYA1	Eyes absent homolog 1; Functions both as protein phosphatase and as transcriptional coactivator for SIX1, and probably also for SIX2, SIX4 and SIX5 (By similarity). Tyrosine phosphatase that dephosphorylates 'Tyr-142' of histone H2AX (H2AXY142ph) and promotes efficient DNA repair via the recruitment of DNA repair complexes containing MDC1. 'Tyr-142' phosphorylation of histone H2AX plays a central role in DNA repair and acts as a mark that distinguishes between apoptotic and repair responses to genotoxic stress. Its function as histone phosphatase may contribute to its function in trans [...]
92	FAAH	9606.ENSP00000243167	FAAH	Fatty-acid amide hydrolase 1; Degrades bioactive fatty acid amides like oleamide, the endogenous cannabinoid, anandamide and myristic amide to their corresponding acids, thereby serving to terminate the signaling functions of these molecules. Hydrolyzes polyunsaturated substrate anandamide preferentially as compared to monounsaturated substrates
93	FAM133A	9606.ENSP00000318974	FAM133A	Protein FAM133A; Family with sequence similarity 133 member A
94	FAM135B	9606.ENSP00000378710	FAM135B	Protein FAM135B; Family with sequence similarity 135 member B
95	FAM167B	9606.ENSP00000362684	FAM167B	Protein FAM167B; Family with sequence similarity 167 member B; Belongs to the FAM167 (SEC) family
96	FAM185A	9606.ENSP00000395340	FAM185A	Protein FAM185A; Family with sequence similarity 185 member A
98	FBLN7	9606.ENSP00000331411	FBLN7	Fibulin-7; An adhesion molecule that interacts with extracellular matrix molecules in developing teeth and may play important roles in differentiation and maintenance of odontoblasts as well as in dentin formation; Fibulins
99	FBXW2	9606.ENSP00000476369	FBXW2	F-box/WD repeat-containing protein 2; Substrate-recognition component of the SCF (SKP1-CUL1-F- box protein)-type E3 ubiquitin ligase complex; F-box and WD repeat domain containing
100	FCRL5	9606.ENSP00000354691	FCRL5	Fc receptor-like protein 5; May be involved in B-cell development and differentiation in peripheral lymphoid organs and may be useful markers of B-cell stages. May have an immunoregulatory role in marginal zone B-cells; CD molecules
101	FCRLB	9606.ENSP00000356925	FCRLB	Fc receptor-like B; Immunoglobulin like domain containing
102	FEZ2	9606.ENSP00000368547	FEZ2	Fasciculation and elongation protein zeta-2; Involved in axonal outgrowth and fasciculation
103	FEZF2	9606.ENSP00000283268	FEZF2	Fez family zinc finger protein 2; Transcription repressor. Required for the specification of corticospinal motor neurons and other subcerebral projection neurons. May play a role in layer and neuronal subtype-specific patterning of subcortical projections and axonal fasciculation. Controls the development of dendritic arborization and spines of large layer V pyramidal neurons. May be involved in innate immunity (By similarity); Zinc fingers C2H2-type
104	FGF3	9606.ENSP00000334122	FGF3	Fibroblast growth factor 3; Plays an important role in the regulation of embryonic development, cell proliferation, and cell differentiation. Required for normal ear development; Belongs to the heparin-binding growth factors family
105	FGFRL1	9606.ENSP00000381498	FGFRL1	Fibroblast growth factor receptor-like 1; Has a negative effect on cell proliferation; I-set domain containing
106	FLYWCH1	9606.ENSP00000399938	FLYWCH1	FLYWCH-type zinc finger 1
107	FMO5	9606.ENSP00000254090	FMO5	Dimethylaniline monooxygenase [N-oxide-forming] 5; In contrast with other forms of FMO it does not seem to be a drug-metabolizing enzyme
108	GATA4	9606.ENSP00000334458	GATA4	Transcription factor GATA-4; Transcriptional activator that binds to the consensus sequence 5'-AGATAG-3' and plays a key role in cardiac development and function. In cooperation with TBX5, it binds to cardiac super-enhancers and promotes cardiomyocyte gene expression, while it downregulates endocardial and endothelial gene expression. Involved in bone morphogenetic protein (BMP)-mediated induction of cardiac-specific gene expression. Binds to BMP response element (BMPRE) DNA sequences within cardiac activating regions (By similarity). Acts as a transcriptional activator of ANF in coop [...]
109	GATAD2A	9606.ENSP00000353463	GATAD2A	Transcriptional repressor p66-alpha; Transcriptional repressor. Enhances MBD2-mediated repression. Efficient repression requires the presence of GATAD2B; GATA zinc finger domain containing
110	GBP1	9606.ENSP00000359504	GBP1	Guanylate-binding protein 1; Hydrolyzes GTP to GMP in 2 consecutive cleavage reactions. Exhibits antiviral activity against influenza virus. Promote oxidative killing and deliver antimicrobial peptides to autophagolysosomes, providing broad host protection against different pathogen classes
111	GCNA	9606.ENSP00000362799	ACRC	Acidic repeat-containing protein; Acidic repeat containing; Belongs to the serine-aspartate repeat-containing protein (SDr) family
112	GK	9606.ENSP00000401720	GK	Glycerol kinase; Key enzyme in the regulation of glycerol uptake and metabolism; Belongs to the FGGY kinase family
113	GNAQ	9606.ENSP00000286548	GNAQ	Guanine nucleotide-binding protein G(q) subunit alpha; Guanine nucleotide-binding proteins (G proteins) are involved as modulators or transducers in various transmembrane signaling systems. Regulates B-cell selection and survival and is required to prevent B-cell-dependent autoimmunity. Regulates chemotaxis of BM-derived neutrophils and dendritic cells (in vitro) (By similarity); Belongs to the G-alpha family. G(q) subfamily
114	GNAS	9606.ENSP00000360141	GNAS	Guanine nucleotide-binding protein G(s) subunit alpha isoforms XLas; Guanine nucleotide-binding proteins (G proteins) function as transducers in numerous signaling pathways controlled by G protein-coupled receptors (GPCRs). Signaling involves the activation of adenylyl cyclases, resulting in increased levels of the signaling molecule cAMP. GNAS functions downstream of several GPCRs, including beta-adrenergic receptors. XLas isoforms interact with the same set of receptors as GNAS isoforms (By similarity)
115	GPATCH4	9606.ENSP00000396441	GPATCH4	G-patch domain containing 4
116	GPBP1	9606.ENSP00000264779	GPBP1	Vasculin; Functions as a GC-rich promoter-specific transactivating transcription factor; Belongs to the vasculin family
117	GPC1	9606.ENSP00000264039	GPC1	Glypican-1; Cell surface proteoglycan that bears heparan sulfate. Binds, via the heparan sulfate side chains, alpha-4 (V) collagen and participates in Schwann cell myelination (By similarity). May act as a catalyst in increasing the rate of conversion of prion protein PRPN(C) to PRNP(Sc) via associating (via the heparan sulfate side chains) with both forms of PRPN, targeting them to lipid rafts and facilitating their interaction. Required for proper skeletal muscle differentiation by sequestering FGF2 in lipid rafts preventing its binding to receptors (FGFRs) and inhibiting the FGF-med [...]
118	GPC6	9606.ENSP00000366246	GPC6	Glypican-6; Cell surface proteoglycan that bears heparan sulfate. Putative cell surface coreceptor for growth factors, extracellular matrix proteins, proteases and anti-proteases (By similarity). Enhances migration and invasion of cancer cells through WNT5A signaling; Belongs to the glypican family
119	GPIHBP1	9606.ENSP00000480053	GPIHBP1	Glycosylphosphatidylinositol-anchored high density lipoprotein-binding protein 1; Plays a key role in the lipolytic processing of chylomicrons. Required for the transport of lipoprotein lipase LPL into the capillary lumen (By similarity); LY6/PLAUR domain containing
120	GPX5	9606.ENSP00000392398	GPX5	Epididymal secretory glutathione peroxidase; Protects cells and enzymes from oxidative damage, by catalyzing the reduction of hydrogen peroxide, lipid peroxides and organic hydroperoxide, by glutathione. May constitute a glutathione peroxidase-like protective system against peroxide damage in sperm membrane lipids
121	GRIA3	9606.ENSP00000481554	GRIA3	Glutamate receptor 3; Receptor for glutamate that functions as ligand-gated ion channel in the central nervous system and plays an important role in excitatory synaptic transmission. L-glutamate acts as an excitatory neurotransmitter at many synapses in the central nervous system. Binding of the excitatory neurotransmitter L- glutamate induces a conformation change, leading to the opening of the cation channel, and thereby converts the chemical signal to an electrical impulse. The receptor then desensitizes rapidly and enters a transient inactive state, characterized by the presence of [...]
122	GRID1	9606.ENSP00000330148	GRID1	Glutamate receptor ionotropic, delta-1; Receptor for glutamate. L-glutamate acts as an excitatory neurotransmitter at many synapses in the central nervous system. The postsynaptic actions of Glu are mediated by a variety of receptors that are named according to their selective agonists; Belongs to the glutamate-gated ion channel (TC 1.A.10.1) family. GRID1 subfamily
123	GRIFIN	9606.ENSP00000481185	GRIFIN	Grifin; Galectin-related inter-fiber protein
124	GRM4	9606.ENSP00000440556	GRM4	Metabotropic glutamate receptor 4; G-protein coupled receptor for glutamate. Ligand binding causes a conformation change that triggers signaling via guanine nucleotide-binding proteins (G proteins) and modulates the activity of down-stream effectors. Signaling inhibits adenylate cyclase activity; Glutamate metabotropic receptors
125	GSDME	9606.ENSP00000339587	DFNA5	Gasdermin-E; Plays a role in the TP53-regulated cellular response to DNA damage probably by cooperating with TP53
126	HDAC4	9606.ENSP00000264606	HDAC4	Histone deacetylase 4; Responsible for the deacetylation of lysine residues on the N-terminal part of the core histones (H2A, H2B, H3 and H4). Histone deacetylation gives a tag for epigenetic repression and plays an important role in transcriptional regulation, cell cycle progression and developmental events. Histone deacetylases act via the formation of large multiprotein complexes. Involved in muscle maturation via its interaction with the myocyte enhancer factors such as MEF2A, MEF2C and MEF2D. Involved in the MTA1-mediated epigenetic regulation of ESR1 expression in breast cancer. [...]
127	HNRNPH2	9606.ENSP00000361927	HNRNPH2	Heterogeneous nuclear ribonucleoprotein H2; This protein is a component of the heterogeneous nuclear ribonucleoprotein (hnRNP) complexes which provide the substrate for the processing events that pre-mRNAs undergo before becoming functional, translatable mRNAs in the cytoplasm. Binds poly(RG); RNA binding motif containing
128	HOXB2	9606.ENSP00000331741	HOXB2	Homeobox protein Hox-B2; Sequence-specific transcription factor which is part of a developmental regulatory system that provides cells with specific positional identities on the anterior-posterior axis; Belongs to the Antp homeobox family. Proboscipedia subfamily
129	HPCAL1	9606.ENSP00000483786	HPCAL1	Hippocalcin-like protein 1; May be involved in the calcium-dependent regulation of rhodopsin phosphorylation; Belongs to the recoverin family
130	HSPG2	9606.ENSP00000363827	HSPG2	Basement membrane-specific heparan sulfate proteoglycan core protein; Integral component of basement membranes. Component of the glomerular basement membrane (GBM), responsible for the fixed negative electrostatic membrane charge, and which provides a barrier which is both size- and charge-selective. It serves as an attachment substrate for cells. Plays essential roles in vascularization. Critical for normal heart development and for regulating the vascular response to injury. Also required for avascular cartilage development; I-set domain containing
131	HTRA3	9606.ENSP00000303766	HTRA3	Serine protease HTRA3; Serine protease that cleaves beta-casein/CSN2 as well as several extracellular matrix (ECM) proteoglycans such as decorin/DCN, biglycan/BGN and fibronectin/FN1. Inhibits signaling mediated by TGF-beta family proteins possibly indirectly by degradation of these ECM proteoglycans (By similarity). May act as a tumor suppressor. Negatively regulates, in vitro, trophoblast invasion during placental development and may be involved in the development of the placenta in vivo. May also have a role in ovarian development, granulosa cell differentiation and luteinization; B [...]
132	HUNK	9606.ENSP00000270112	HUNK	Hormonally up-regulated Neu-associated kinase; Belongs to the protein kinase superfamily. CAMK Ser/Thr protein kinase family. SNF1 subfamily
133	IDH3G	9606.ENSP00000217901	IDH3G	Isocitrate dehydrogenase [NAD] subunit gamma, mitochondrial; Regulatory subunit which plays a role in the allosteric regulation of the enzyme catalyzing the decarboxylation of isocitrate (ICT) into alpha-ketoglutarate. The heterodimer composed of the alpha (IDH3A) and beta (IDH3B) subunits and the heterodimer composed of the alpha (IDH3A) and gamma (IDH3G) subunits, have considerable basal activity but the full activity of the heterotetramer (containing two subunits of IDH3A, one of IDH3B and one of IDH3G) requires the assembly and cooperative function of both heterodimers
135	IGSF21	9606.ENSP00000251296	IGSF21	Immunoglobulin superfamily member 21; Immunoglobulin like domain containing
136	IL12A	9606.ENSP00000303231	IL12A	Interleukin-12 subunit alpha; Cytokine that can act as a growth factor for activated T and NK cells, enhance the lytic activity of NK/lymphokine-activated Killer cells, and stimulate the production of IFN-gamma by resting PBMC; Interleukins
137	IL20RB	9606.ENSP00000328133	IL20RB	Interleukin-20 receptor subunit beta; The IL20RA/IL20RB dimer is a receptor for IL19, IL20 and IL24. The IL22RA1/IL20RB dimer is a receptor for IL20 and IL24; Fibronectin type III domain containing
138	IL22	9606.ENSP00000442424	IL22	Interleukin-22; Cytokine that contributes to the inflammatory response in vivo; Interleukins
139	INPP5A	9606.ENSP00000357583	INPP5A	Type I inositol 1,4,5-trisphosphate 5-phosphatase; Major isoenzyme hydrolyzing the calcium-mobilizing second messenger Ins(1,4,5)P3, this is a signal-terminating reaction; Belongs to the inositol 1,4,5-trisphosphate 5- phosphatase type I family
140	INTU	9606.ENSP00000334003	INTU	Protein inturned; Plays a key role in ciliogenesis and embryonic development. Regulator of cilia formation by controlling the organization of the apical actin cytoskeleton and the positioning of the basal bodies at the apical cell surface, which in turn is essential for the normal orientation of elongating ciliary microtubules. Plays a key role in definition of cell polarity via its role in ciliogenesis but not via conversion extension. Has an indirect effect on hedgehog signaling (By similarity). Proposed to function as core component of the CPLANE (ciliogenesis and planar polarity ef [...]
141	IRAK2	9606.ENSP00000256458	IRAK2	Interleukin-1 receptor-associated kinase-like 2; Binds to the IL-1 type I receptor following IL-1 engagement, triggering intracellular signaling cascades leading to transcriptional up-regulation and mRNA stabilization

Treatment

#	queryItem	stringId	preferredName	annotation
142	IRF2BP1	9606.ENSP00000307265	IRF2BP1	Interferon regulatory factor 2-binding protein 1; Acts as a transcriptional corepressor in a IRF2- dependent manner; this repression is not mediated by histone deacetylase activities. May act as an E3 ligase towards JDP2, enhancing its polyubiquitination. Represses ATF2-dependent transcriptional activation
143	IRF8	9606.ENSP00000268638	IRF8	Interferon regulatory factor 8; Plays a role as a transcriptional activator or repressor. Specifically binds to the upstream regulatory region of type I IFN and IFN-inducible MHC class I genes (the interferon consensus sequence (ICS)). Plays a negative regulatory role in cells of the immune system. Involved in CD8(+) dendritic cell differentiation by forming a complex with the BATF-JUNB heterodimer in immune cells, leading to recognition of AICE sequence (5'-TGAnTCA/GAAA-3'), an immune-specific regulatory element, followed by cooperative binding of BATF and IRF8 and activation of genes [...]
144	IRX4	9606.ENSP00000482393	IRX4	Iroquois-class homeodomain protein IRX-4; Likely to be an important mediator of ventricular differentiation during cardiac development; TALE class homeoboxes and pseudogenes
145	ITFG2	9606.ENSP00000228799	ITFG2	KICSTOR complex protein ITFG2; As part of the KICSTOR complex functions in the amino acid-sensing branch of the TORC1 signaling pathway. Recruits, in an amino acid-independent manner, the GATOR1 complex to the lysosomal membranes and allows its interaction with GATOR2 and the RAG GTPases. Functions upstream of the RAG GTPases and is required to negatively regulate mTORC1 signaling in absence of amino acids. In absence of the KICSTOR complex mTORC1 is constitutively localized to the lysosome and activated. The KICSTOR complex is also probably involved in the regulation of mTORC1 by glucose
146	ITGB2	9606.ENSP00000380948	ITGB2	Integrin beta-2; Integrin ITGAL/ITGB2 is a receptor for ICAM1, ICAM2, ICAM3 and ICAM4. Integrins ITGAM/ITGB2 and ITGAX/ITGB2 are receptors for the iC3b fragment of the third complement component and for fibrinogen. Integrin ITGAX/ITGB2 recognizes the sequence G-P-R in fibrinogen alpha-chain. Integrin ITGAM/ITGB2 recognizes P1 and P2 peptides of fibrinogen gamma chain. Integrin ITGAM/ITGB2 is also a receptor for factor X. Integrin ITGAD/ITGB2 is a receptor for ICAM3 and VCAM1. Contributes to natural killer cell cytotoxicity. Involved in leukocyte adhesion and transmigration of leukocyte [...]
147	ITGB8	9606.ENSP00000222573	ITGB8	Integrin beta-8; Integrin alpha-V/beta-8 is a receptor for fibronectin
148	KCTD3	9606.ENSP00000259154	KCTD3	BTB/POZ domain-containing protein KCTD3; Accessory subunit of potassium/sodium hyperpolarization- activated cyclic nucleotide-gated channel 3 (HCN3) upregulating its cell-surface expression and current density without affecting its voltage dependence and kinetics; Belongs to the KCTD3 family
149	KDELR3	9606.ENSP00000386918	KDELR3	ER lumen protein-retaining receptor 3; Required for the retention of luminal endoplasmic reticulum proteins. Determines the specificity of the luminal ER protein retention system. Also required for normal vesicular traffic through the Golgi. This receptor recognizes K-D-E-L (By similarity)
150	KIAA1549L	9606.ENSP00000315295	KIAA1549L	UPF0606 protein KIAA1549L; KIAA1549 like; Belongs to the UPF0606 family
151	KIF20B	9606.ENSP00000360793	KIF20B	Kinesin-like protein KIF20B; Plus-end-directed motor enzyme that is required for completion of cytokinesis. Required for proper midbody organization and abscission in polarized cortical stem cells. Plays a role in the regulation of neuronal polarization by mediating the transport of specific cargos. Participates in the mobilization of SHTN1 and in the accumulation of PIP3 in the growth cone of primary hippocampal neurons in a tubulin and actin-dependent manner. In the developing telencephalon, cooperates with SHTN1 to promote both the transition from the multipolar to the bipolar stage [...]
152	KIF26A	9606.ENSP00000388241	KIF26A	Kinesin-like protein KIF26A; Atypical kinesin that plays a key role in enteric neuron development. Acts by repressing a cell growth signaling pathway in the enteric nervous system development, possibly via its interaction with GRB2 that prevents GRB2-binding to SHC, thereby attenating the GDNF-Ret signaling. Binds to microtubules but lacks microtubule-based motility due to the absence of ATPase activity (By similarity); Belongs to the TRAFAC class myosin-kinesin ATPase superfamily. Kinesin family. KIF26 subfamily
153	KIFC1	9606.ENSP00000393963	KIFC1	Kinesin-like protein KIFC1; Minus end-directed microtubule-dependent motor required for bipolar spindle formation. May contribute to movement of early endocytic vesicles (By similarity). Regulates cilium formation and structure (By similarity); Belongs to the TRAFAC class myosin-kinesin ATPase superfamily. Kinesin family. NCD subfamily
154	KNDC1	9606.ENSP00000304437	KNDC1	Protein very KIND; RAS-Guanine nucleotide exchange factor (GEF) that controls the negative regulation of neuronal dendrite growth by mediating a signaling pathway linking RAS and MAP2 (By similarity). May be involved in cellular senescence
155	L1TD1	9606.ENSP00000419901	L1TD1	LINE1 type transposase domain containing 1; Belongs to the transposase 22 family
156	LBX1	9606.ENSP00000359212	LBX1	Transcription factor LBX1; Transcription factor required for the development of GABAergic interneurons in the dorsal horn of the spinal cord and migration and further development of hypaxial muscle precursor cells for limb muscles, diaphragm and hypoglossal cord; NKL subclass homeoboxes and pseudogenes
157	LHX1	9606.ENSP00000477829	LHX1	LIM/homeobox protein Lhx1; Potential transcription factor. May play a role in early mesoderm formation and later in lateral mesoderm differentiation and neurogenesis; LIM class homeoboxes
158	LIPI	9606.ENSP00000343331	LIPI	Lipase member I; Hydrolyzes specifically phosphatidic acid (PA) to produce 2-acyl lysophosphatidic acid (LPA; a potent bioactive lipid mediator) and fatty acid. Does not hydrolyze other phospholipids, like phosphatidylserine (PS), phosphatidylcholine (PC) and phosphatidylethanolamine (PE) or triacylglycerol (TG); Belongs to the AB hydrolase superfamily. Lipase family
160	LRP8	9606.ENSP00000303634	LRP8	Low-density lipoprotein receptor-related protein 8; Cell surface receptor for Reelin (RELN) and apolipoprotein E (apoE)-containing ligands. LRP8 participates in transmitting the extracellular Reelin signal to intracellular signaling processes, by binding to DAB1 on its cytoplasmic tail. Reelin acts via both the VLDL receptor (VLDLR) and LRP8 to regulate DAB1 tyrosine phosphorylation and microtubule function in neurons. LRP8 has higher affinity for Reelin than VLDLR. LRP8 is thus a key component of the Reelin pathway which governs neuronal layering of the forebrain during embryonic brai [...]
161	LRRC28	9606.ENSP00000304923	LRRC28	Leucine-rich repeat-containing protein 28; Leucine rich repeat containing 28
162	LRRC37B	9606.ENSP00000340519	LRRC37B	Leucine-rich repeat-containing protein 37B; Leucine rich repeat containing 37B
163	LRRC58	9606.ENSP00000295628	LRRC58	Leucine-rich repeat-containing protein 58; Leucine rich repeat containing 58
164	LUZP4	9606.ENSP00000360988	LUZP4	Leucine zipper protein 4; Export adapter involved in mRNA nuclear export in cancer cells. Binds and enhances the RNA-binding activity of the nuclear RNA export factor NXF1. Can restore mRNA export function in cells compromised by loss of mRNA export adapters
165	LY6H	9606.ENSP00000399485	LY6H	Lymphocyte antigen 6H; Believed to act as a modulator of nicotinic acetylcholine receptors (nAChRs) activity. In vitro inhibits alpha-3:beta-4-containing nAChRs maximum response. May play a role in the intracellular trafficking of alpha-7-containing nAChRs and may inhibit their expression at the cell surface. Seems to inhibit alpha-7/CHRNA7 signaling in hippocampal neurons; LY6/PLAUR domain containing
166	MAGT1	9606.ENSP00000354649	MAGT1	Magnesium transporter protein 1; Acts as accessory component of the N-oligosaccharyl transferase (OST) complex which catalyzes the transfer of a high mannose oligosaccharide from a lipid-linked oligosaccharide donor to an asparagine residue within an Asn-X-Ser/Thr consensus motif in nascent polypeptide chains. Involved in N-glycosylation of STT3B-dependent substrates. Specifically required for the glycosylation of a subset of acceptor sites that are near cysteine residues; in this function seems to act redundantly with TUSC3. In its oxidized form proposed to form transient mixed disulf [...]
167	MAP2K4	9606.ENSP00000410402	MAP2K4	Dual specificity mitogen-activated protein kinase kinase 4; Dual specificity protein kinase which acts as an essential component of the MAP kinase signal transduction pathway. Essential component of the stress-activated protein kinase/c-Jun N-terminal kinase (SAP/JNK) signaling pathway. With MAP2K7/MKK7, is the one of the only known kinase to directly activate the stress-activated protein kinase/c-Jun N-terminal kinases MAPK8/JNK1, MAPK9/JNK2 and MAPK10/JNK3. MAP2K4/MKK4 and MAP2K7/MKK7 both activate the JNKs by phosphorylation, but they differ in their preference for the phosphorylati [...]
168	MAPKAP1	9606.ENSP00000265960	MAPKAP1	Target of rapamycin complex 2 subunit MAPKAP1; Subunit of mTORC2, which regulates cell growth and survival in response to hormonal signals. mTORC2 is activated by growth factors, but, in contrast to mTORC1, seems to be nutrient- insensitive. mTORC2 seems to function upstream of Rho GTPases to regulate the actin cytoskeleton, probably by activating one or more Rho-type guanine nucleotide exchange factors. mTORC2 promotes the serum-induced formation of stress-fibers or F-actin. mTORC2 plays a critical role in AKT1 'Ser-473' phosphorylation, which may facilitate the phosphorylation of the [...]
169	MBL2	9606.ENSP00000363079	MBL2	Mannose-binding protein C; Calcium-dependent lectin involved in innate immune defense. Binds mannose, fucose and N-acetylglucosamine on different microorganisms and activates the lectin complement pathway. Binds to late apoptotic cells, as well as to apoptotic blebs and to necrotic cells, but not to early apoptotic cells, facilitating their uptake by macrophages. May bind DNA; Collectins
170	MED13L	9606.ENSP00000281928	MED13L	Mediator of RNA polymerase II transcription subunit 13-like; Component of the Mediator complex, a coactivator involved in the regulated transcription of nearly all RNA polymerase II-dependent genes. Mediator functions as a bridge to convey information from gene-specific regulatory proteins to the basal RNA polymerase II transcription machinery. Mediator is recruited to promoters by direct interactions with regulatory proteins and serves as a scaffold for the assembly of a functional preinitiation complex with RNA polymerase II and the general transcription factors. This subunit may spe [...]
171	MED21	9606.ENSP00000282892	MED21	Mediator of RNA polymerase II transcription subunit 21; Component of the Mediator complex, a coactivator involved in the regulated transcription of nearly all RNA polymerase II-dependent genes. Mediator functions as a bridge to convey information from gene-specific regulatory proteins to the basal RNA polymerase II transcription machinery. Mediator is recruited to promoters by direct interactions with regulatory proteins and serves as a scaffold for the assembly of a functional preinitiation complex with RNA polymerase II and the general transcription factors
172	MEF2D	9606.ENSP00000271555	MEF2D	Myocyte-specific enhancer factor 2D; Transcriptional activator which binds specifically to the MEF2 element, 5'-YTA[AT](4)TAR-3', found in numerous muscle- specific, growth factor- and stress-induced genes. Mediates cellular functions not only in skeletal and cardiac muscle development, but also in neuronal differentiation and survival. Plays diverse roles in the control of cell growth, survival and apoptosis via p38 MAPK signaling in muscle-specific and/or growth factor-related transcription. Plays a critical role in the regulation of neuronal apoptosis (By similarity)
173	MICALL2	9606.ENSP00000297508	MICALL2	MICAL-like protein 2; Effector of small Rab GTPases which is involved in junctional complexes assembly through the regulation of cell adhesion molecules transport to the plasma membrane and actin cytoskeleton reorganization. Regulates the endocytic recycling of occludins, claudins and E-cadherin to the plasma membrane and may thereby regulate the establishment of tight junctions and adherens junctions. In parallel, may regulate actin cytoskeleton reorganization directly through interaction with F-actin or indirectly through actinins and filamins. Most probably involved in the processes [...]
174	MICU3	9606.ENSP00000321455	MICU3	Calcium uptake protein 3, mitochondrial; May play a role in mitochondrial calcium uptake; EF-hand domain containing
175	MIPOL1	9606.ENSP00000333539	MIPOL1	Mirror-image polydactyly 1
177	MKLN1	9606.ENSP00000323527	MKLN1	Muskelin; Acts as a mediator of cell spreading and cytoskeletal responses to the extracellular matrix component THBS1; CTLH complex
178	MMS22L	9606.ENSP00000275053	MMS22L	Protein MMS22-like; Component of the MMS22L-TONSL complex, a complex that stimulates the recombination-dependent repair of stalled or collapsed replication forks. The MMS22L-TONSL complex is required to maintain genome integrity during DNA replication by promoting homologous recombination-mediated repair of replication fork- associated double-strand breaks. It may act by mediating the assembly of RAD51 filaments on ssDNA
179	MOCOS	9606.ENSP00000261326	MOCOS	Molybdenum cofactor sulfurase; Sulfurates the molybdenum cofactor. Sulfation of molybdenum is essential for xanthine dehydrogenase (XDH) and aldehyde oxidase (ADO) enzymes in which molybdenum cofactor is liganded by 1 oxygen and 1 sulfur atom in active form. In vitro, the C-terminal domain is able to reduce N-hydroxylated prodrugs, such as benzamidoxime; Belongs to the class-V pyridoxal-phosphate-dependent aminotransferase family. MOCOS subfamily
180	MORC4	9606.ENSP00000347821	MORC4	MORC family CW-type zinc finger 4
181	MTRNR2L1	9606.ENSP00000439228	MTRNR2L1	Humanin-like 1; Plays a role as a neuroprotective and antiapoptotic factor; Belongs to the humanin family
182	MUC6	9606.ENSP00000406861	MUC6	Mucin-6; May provide a mechanism for modulation of the composition of the protective mucus layer related to acid secretion or the presence of bacteria and noxious agents in the lumen. Plays an important role in the cytoprotection of epithelial surfaces and are used as tumor markers in a variety of cancers. May play a role in epithelial organogenesis; Mucins
183	MYO5A	9606.ENSP00000382177	MYO5A	Unconventional myosin-Va; Processive actin-based motor that can move in large steps approximating the 36-nm pseudo-repeat of the actin filament. Involved in melanosome transport. Also mediates the transport of vesicles to the plasma membrane. May also be required for some polarization process involved in dendrite formation; Myosins, class V
184	NEIL3	9606.ENSP00000264596	NEIL3	Endonuclease 8-like 3; DNA glycosylase which prefers single-stranded DNA (ssDNA), or partially ssDNA structures such as bubble and fork structures, to double-stranded DNA (dsDNA). In vitro, displays strong glycosylase activity towards the hydantoin lesions spiroiminodihydantoin (Sp) and guanidinohydantoin (Gh) in both ssDNA and dsDNA; also recognizes FapyA, FapyG, 5-OHU, 5-OHC, 5- OHMH, Tg and 8-oxoA lesions in ssDNA. No activity on 8-oxoG detected. Also shows weak DNA-(apurinic or apyrimidinic site) lyase activity. In vivo, appears to be the primary enzyme involved in removing Sp and [...]

Treatment

#	queryItem	stringId	preferredName	annotation
185	NKX1-1	9606.ENSP00000407978	NKX1-1	NK1 transcription factor-related protein 1; NKL subclass homeoboxes and pseudogenes; Belongs to the NK-1 homeobox family
186	NKX2-3	9606.ENSP00000342828	NKX2-3	Homeobox protein Nkx-2.3; Transcription factor; NKL subclass homeoboxes and pseudogenes
187	NKX2-5	9606.ENSP00000327758	NKX2-5	Homeobox protein Nkx-2.5; Implicated in commitment to and/or differentiation of the myocardial lineage. Acts as a transcriptional activator of ANF in cooperation with GATA4 (By similarity). Binds to the core DNA motif of NPPA promoter. It is transcriptionally controlled by PBX1 and acts as a transcriptional repressor of CDKN2B (By similarity). It is required for spleen development; NKL subclass homeoboxes and pseudogenes
188	NLRP8	9606.ENSP00000291971	NLRP8	NACHT, LRR and PYD domains-containing protein 8; Involved in inflammation; NLR family
189	NMD3	9606.ENSP00000419004	NMD3	60S ribosomal export protein NMD3; Acts as an adapter for the XPO1/CRM1-mediated export of the 60S ribosomal subunit
190	NOP58	9606.ENSP00000264279	NOP58	Nucleolar protein 58; Required for 60S ribosomal subunit biogenesis (By similarity). Core component of box C/D small nucleolar ribonucleoprotein (snoRNP) particles. Required for the biogenesis of box C/D snoRNAs such as U3, U8 and U14 snoRNAs; Belongs to the NOP5/NOP56 family
191	NPRL3	9606.ENSP00000483814	NPRL3	GATOR complex protein NPRL3; As a component of the GATOR1 complex functions as an inhibitor of the amino acid-sensing branch of the TORC1 pathway. The GATOR1 complex strongly increases GTP hydrolysis by RRAGA and RRAGB within RRAGC-containing heterodimers, thereby deactivating RRAGs, releasing mTORC1 from lysosomal surface and inhibiting mTORC1 signaling. The GATOR1 complex is negatively regulated by GATOR2 the other GATOR subcomplex in this amino acid-sensing branch of the TORC1 pathway; Belongs to the NPR3 family
192	NR4A1	9606.ENSP00000440864	NR4A1	Nuclear receptor subfamily 4 group A member 1; Orphan nuclear receptor. May act concomitantly with NURR1 in regulating the expression of delayed-early genes during liver regeneration. Binds the NGFI-B response element (NBRE) 5'- AAAAGGTCA-3' (By similarity). May inhibit NF-kappa-B transactivation of IL2. Participates in energy homeostasis by sequestrating the kinase STK11 in the nucleus, thereby attenuating cytoplasmic AMPK activation; Nuclear hormone receptors
193	NRP2	9606.ENSP00000353582	NRP2	Neuropilin-2; High affinity receptor for semaphorins 3C, 3F, VEGF-165 and VEGF-145 isoforms of VEGF, and the PLGF-2 isoform of PGF
194	OSBPL3	9606.ENSP00000315410	OSBPL3	Oxysterol-binding protein-related protein 3; Phosphoinositide-binding protein which associates with both cell and endoplasmic reticulum (ER) membranes. Can bind to the ER membrane protein VAPA and recruit VAPA to plasma membrane sites, thus linking these intracellular compartments. The ORP3-VAPA complex stimulates RRAS signaling which in turn attenuates integrin beta-1 (ITGB1) activation at the cell surface. With VAPA, may regulate ER morphology. Has a role in regulation of the actin cytoskeleton, cell polarity and cell adhesion. Binds to phosphoinositides with preference for PI(3,4)P2 [...]
195	OSR1	9606.ENSP00000272223	OSR1	Protein odd-skipped-related 1; Transcription factor that plays a role in the regulation of embryonic heart and urogenital development; Zinc fingers C2H2-type
196	PALM2	9606.ENSP00000323805	PALM2	Paralemmin-2; Paralemmin 2
197	PAPLN	9606.ENSP00000345395	PAPLN	Papilin, proteoglycan like sulfated glycoprotein; Belongs to the papilin family
198	PAQR5	9606.ENSP00000378803	PAQR5	Membrane progestin receptor gamma; Plasma membrane progesterone (P4) receptor coupled to G proteins. Seems to act through a G(i) mediated pathway. May be involved in oocyte maturation; Progestin and adipoQ receptor family
199	PCBD2	9606.ENSP00000421544	PCBD2	Pterin-4-alpha-carbinolamine dehydratase 2; Involved in tetrahydrobiopterin biosynthesis. Seems to both prevent the formation of 7-pterins and accelerate the formation of quinonoid-BH2 (By similarity)
200	PCDHB6	9606.ENSP00000231136	PCDHB6	Protocadherin beta-6; Calcium-dependent cell-adhesion protein involved in cells self-recognition and non-self discrimination. Thereby, it is involved in the establishment and maintenance of specific neuronal connections in the brain; Clustered protocadherins
201	PCDHGA4	9606.ENSP00000458570	PCDHGA4	Protocadherin gamma-A4; Potential calcium-dependent cell-adhesion protein. May be involved in the establishment and maintenance of specific neuronal connections in the brain; Clustered protocadherins
202	PCNX4	9606.ENSP00000317396	PCNXL4	Pecanex-like 4 (Drosophila); Belongs to the pecanex family
203	PDHX	9606.ENSP00000227868	PDHX	Pyruvate dehydrogenase protein X component, mitochondrial; Required for anchoring dihydrolipoamide dehydrogenase (E3) to the dihydrolipoamide transacetylase (E2) core of the pyruvate dehydrogenase complexes of eukaryotes. This specific binding is essential for a functional PDH complex
204	PDIA2	9606.ENSP00000219406	PDIA2	Protein disulfide-isomerase A2; Acts as an intracellular estrogen-binding protein. May be involved in modulating cellular levels and biological functions of estrogens in the pancreas. May act as a chaperone that inhibits aggregation of misfolded proteins; Protein disulfide isomerases
205	PDZK1IP1	9606.ENSP00000294338	PDZK1IP1	PDZK1-interacting protein 1; May play an important role in tumor biology
206	PLD2	9606.ENSP00000263088	PLD2	Phospholipase D2; May have a role in signal-induced cytoskeletal regulation and/or endocytosis; Phospholipases
207	PNPLA7	9606.ENSP00000384610	PNPLA7	Patatin-like phospholipase domain-containing protein 7; Serine hydrolase, whose specific chemical modification by certain organophosphorus (OP) compounds leads to distal axonopathy; Patatin like phospholipase domain containing
208	POLD2	9606.ENSP00000480186	POLD2	DNA polymerase delta subunit 2; As a component of the trimeric and tetrameric DNA polymerase delta complexes (Pol-delta3 and Pol-delta4, respectively), plays a role in high fidelity genome replication, including in lagging strand synthesis, and repair. Pol-delta3 and Pol-delta4 are characterized by the absence or the presence of POLD4. They exhibit differences in catalytic activity. Most notably, Pol- delta3 shows higher proofreading activity than Pol-delta4. Although both Pol-delta3 and Pol-delta4 process Okazaki fragments in vitro, Pol-delta3 may also be better suited to fulfill this [...]
209	POLG	9606.ENSP00000268124	POLG	DNA polymerase subunit gamma-1; Involved in the replication of mitochondrial DNA. Associates with mitochondrial DNA; Belongs to the DNA polymerase type-A family
210	PPFIA4	9606.ENSP00000272198	PPFIA4	Liprin-alpha-4; May regulate the disassembly of focal adhesions. May localize receptor-like tyrosine phosphatases type 2A at specific sites on the plasma membrane, possibly regulating their interaction with the extracellular environment and their association with substrates (By similarity); Belongs to the liprin family. Liprin-alpha subfamily
211	PPME1	9606.ENSP00000381461	PPME1	Protein phosphatase methylesterase 1; Demethylates proteins that have been reversibly carboxymethylated. Demethylates PPP2CB (in vitro) and PPP2CA. Binding to PPP2CA displaces the manganese ion and inactivates the enzyme; Protein phosphatase 2 modulatory subunits
212	PPP2R2A	9606.ENSP00000325074	PPP2R2A	Serine/threonine-protein phosphatase 2A 55 kDa regulatory subunit B alpha isoform; The B regulatory subunit might modulate substrate selectivity and catalytic activity, and also might direct the localization of the catalytic enzyme to a particular subcellular compartment; Protein phosphatase 2 regulatory subunits
213	PRDM7	9606.ENSP00000396732	PRDM7	Probable histone-lysine N-methyltransferase PRDM7; Probable histone methyltransferase; PR/SET domain family
214	PRKD1	9606.ENSP00000333568	PRKD1	Serine/threonine-protein kinase D1; Serine/threonine-protein kinase that converts transient diacylglycerol (DAG) signals into prolonged physiological effects downstream of PKC, and is involved in the regulation of MAPK8/JNK1 and Ras signaling, Golgi membrane integrity and trafficking, cell survival through NF-kappa-B activation, cell migration, cell differentiation by mediating HDAC7 nuclear export, cell proliferation via MAPK1/3 (ERK1/2) signaling, and plays a role in cardiac hypertrophy, VEGFA-induced angiogenesis, genotoxic-induced apoptosis and flagellin-stimulated inflammatory res [...]
215	PRR16	9606.ENSP00000385118	PRR16	Protein Largen; Regulator of cell size that promotes cell size increase independently of mTOR and Hippo signaling pathways. Acts by stimulating the translation of specific mRNAs, including those encoding proteins affecting mitochondrial functions. Increases mitochondrial mass and respiration
216	PTPRN2	9606.ENSP00000374069	PTPRN2	Receptor-type tyrosine-protein phosphatase N2; Plays a role in vesicle-mediated secretory processes. Required for normal accumulation of secretory vesicles in hippocampus, pituitary and pancreatic islets. Required for the accumulation of normal levels of insulin-containing vesicles and preventing their degradation. Plays a role in insulin secretion in response to glucose stimuli. Required for normal accumulation of the neurotransmitters norepinephrine, dopamine and serotonin in the brain. In females, but not in males, required for normal accumulation and secretion of pituitary hormones [...]
217	RAD52	9606.ENSP00000351284	RAD52	DNA repair protein RAD52 homolog; Involved in double-stranded break repair. Plays a central role in genetic recombination and DNA repair by promoting the annealing of complementary single-stranded DNA and by stimulation of the RAD51 recombinase; Belongs to the RAD52 family
218	RAPGEF5	9606.ENSP00000343656	RAPGEF5	Rap guanine nucleotide exchange factor 5; Guanine nucleotide exchange factor (GEF) for RAP1A, RAP2A and MRAS/M-Ras-GTP. Its association with MRAS inhibits Rap1 activation
219	RELN	9606.ENSP00000392423	RELN	Reelin; Extracellular matrix serine protease that plays a role in layering of neurons in the cerebral cortex and cerebellum. Regulates microtubule function in neurons and neuronal migration. Affects migration of sympathetic preganglionic neurons in the spinal cord, where it seems to act as a barrier to neuronal migration. Enzymatic activity is important for the modulation of cell adhesion. Binding to the extracellular domains of lipoprotein receptors VLDLR and LRP8/APOER2 induces tyrosine phosphorylation of DAB1 and modulation of TAU phosphorylation (By similarity); Belongs to the reel [...]
220	RGL1	9606.ENSP00000303192	RGL1	Ral guanine nucleotide dissociation stimulator-like 1; Probable guanine nucleotide exchange factor
221	RHOU	9606.ENSP00000355652	RHOU	Rho-related GTP-binding protein RhoU; Acts upstream of PAK1 to regulate the actin cytoskeleton, adhesion turnover and increase cell migration. Stimulates quiescent cells to reenter the cell cycle. Has no detectable GTPase activity but its high intrinsic guanine nucleotide exchange activity suggests it is constitutively GTP- bound. Plays a role in the regulation of cell morphology and cytoskeletal organization. Required in the control of cell shape; Belongs to the small GTPase superfamily. Rho family
222	RHPN2	9606.ENSP00000254260	RHPN2	Rhophilin-2; Binds specifically to GTP-Rho. May function in a Rho pathway to limit stress fiber formation and/or increase the turnover of F-actin structures in the absence of high levels of RhoA activity; Belongs to the RHPN family
223	RIMS4	9606.ENSP00000439287	RIMS4	Regulating synaptic membrane exocytosis protein 4; Regulates synaptic membrane exocytosis
224	RIPOR2	9606.ENSP00000482957	FAM65B	Rho family-interacting cell polarization regulator 2; Acts as an inhibitor of the small GTPase RHOA and plays several roles in the regulation of myoblast and hair cell differentiation, lymphocyte T proliferation and neutrophil polarization. Inhibits chemokine-induced T lymphocyte responses, such as cell adhesion, polarization and migration. Involved also in the regulation of neutrophil polarization, chemotaxis and adhesion (By similarity). Required for normal development of inner and outer hair cell stereocilia within the cochlea of the inner ear (By similarity). Plays a role for maint [...]
227	S1PR1	9606.ENSP00000305416	S1PR1	Sphingosine 1-phosphate receptor 1; G-protein coupled receptor for the bioactive lysosphingolipid sphingosine 1-phosphate (S1P) that seems to be coupled to the G(i) subclass of heteromeric G proteins. Signaling leads to the activation of RAC1, SRC, PTK2/FAK1 and MAP kinases. Plays an important role in cell migration, probably via its role in the reorganization of the actin cytoskeleton and the formation of lamellipodia in response to stimuli that increase the activity of the sphingosine kinase SPHK1. Required for normal chemotaxis toward sphingosine 1-phosphate. Required for normal emb [...]
228	SAMD5	9606.ENSP00000356444	SAMD5	Sterile alpha motif domain containing 5
229	SATB2	9606.ENSP00000401112	SATB2	DNA-binding protein SATB2; Binds to DNA, at nuclear matrix- or scaffold-associated regions. Thought to recognize the sugar-phosphate structure of double-stranded DNA. Transcription factor controlling nuclear gene expression, by binding to matrix attachment regions (MARs) of DNA and inducing a local chromatin-loop remodeling. Acts as a docking site for several chromatin remodeling enzymes and also by recruiting corepressors (HDACs) or coactivators (HATs) directly to promoters and enhancers. Required for the initiation of the upper- layer neurons (UL1) specific genetic program and for th [...]
230	SDHAF3	9606.ENSP00000414066	ACN9	Succinate dehydrogenase assembly factor 3, mitochondrial; Plays an essential role in the assembly of succinate dehydrogenase (SDH), an enzyme complex (also referred to as respiratory complex II) that is a component of both the tricarboxylic acid (TCA) cycle and the mitochondrial electron transport chain, and which couples the oxidation of succinate to fumarate with the reduction of ubiquinone (coenzyme Q) to ubiquinol. Promotes maturation of the iron-sulfur protein subunit SDHB of the SDH catalytic dimer, protecting it from the deleterious effects of oxidants. May act together with SDHAF1
231	SEZ6	9606.ENSP00000312942	SEZ6	Seizure protein 6 homolog; May play a role in cell-cell recognition and in neuronal membrane signaling. Seems to be important for the achievement of the necessary balance between dendrite elongation and branching during the elaboration of a complex dendritic arbor. Involved in the development of appropriate excitatory synaptic connectivity (By similarity); Belongs to the SEZ6 family
232	SGCE	9606.ENSP00000398930	SGCE	Epsilon-sarcoglycan; Component of the sarcoglycan complex, a subcomplex of the dystrophin-glycoprotein complex which forms a link between the F-actin cytoskeleton and the extracellular matrix
233	SHANK2	9606.ENSP00000469689	SHANK2	SH3 and multiple ankyrin repeat domains protein 2; Seems to be an adapter protein in the postsynaptic density (PSD) of excitatory synapses that interconnects receptors of the postsynaptic membrane including NMDA-type and metabotropic glutamate receptors, and the actin-based cytoskeleton. May play a role in the structural and functional organization of the dendritic spine and synaptic junction; Belongs to the SHANK family
234	SIX1	9606.ENSP00000247182	SIX1	Homeobox protein SIX1; Transcription factor that is involved in the regulation of cell proliferation, apoptosis and embryonic development. Plays an important role in the development of several organs, including kidney, muscle and inner ear. Depending on context, functions as transcriptional repressor or activator. Lacks an activation domain, and requires interaction with EYA family members for transcription activation. Mediates

#	queryItem	stringId	preferredName	annotation
				nuclear translocation of EYA1 and EYA2. Binds the 5'-TCA[AG][AG]TTNC-3' motif present in the MEF3 element in the MYOG promoter. Regulates the expression of nume [...]
235	SIX2	9606.ENSP00000304502	SIX2	Homeobox protein SIX2; Transcription factor that plays an important role in the development of several organs, including kidney, skull and stomach. During kidney development, maintains cap mesenchyme multipotent nephron progenitor cells in an undifferentiated state by opposing the inductive signals emanating from the ureteric bud and cooperates with WNT9B to promote renewing progenitor cells proliferation. Acts through its interaction with TCF7L2 and OSR1 in a canonical Wnt signaling independent manner preventing transcription of differentiation genes in cap mesenchyme such as WNT4. Al [...]
236	SLC17A8	9606.ENSP00000316909	SLC17A8	Vesicular glutamate transporter 3; Mediates the uptake of glutamate into synaptic vesicles at presynaptic nerve terminals of excitatory neural cells. May also mediate the transport of inorganic phosphate; Deafness associated genes
237	SLC2A1	9606.ENSP00000416293	SLC2A1	Solute carrier family 2, facilitated glucose transporter member 1; Facilitative glucose transporter. This isoform may be responsible for constitutive or basal glucose uptake. Has a very broad substrate specificity; can transport a wide range of aldoses including both pentoses and hexoses; Belongs to the major facilitator superfamily. Sugar transporter (TC 2.A.1.1) family. Glucose transporter subfamily
238	SLC35A5	9606.ENSP00000417654	SLC35A5	Probable UDP-sugar transporter protein SLC35A5; Solute carrier family 35 member A5
239	SLC9A3	9606.ENSP00000264938	SLC9A3	Sodium/hydrogen exchanger 3; Involved in pH regulation to eliminate acids generated by active metabolism or to counter adverse environmental conditions. Major proton extruding system driven by the inward sodium ion chemical gradient. Plays an important role in signal transduction; Solute carriers
240	SLCO1A2	9606.ENSP00000305974	SLCO1A2	Solute carrier organic anion transporter family member 1A2; Mediates the Na(+)-independent transport of organic anions such as sulfobromophthalein (BSP) and conjugated (taurocholate) and unconjugated (cholate) bile acids (By similarity). Selectively inhibited by the grapefruit juice component naringin; Solute carriers
241	SLIT1	9606.ENSP00000266058	SLIT1	Slit homolog 1 protein; Thought to act as molecular guidance cue in cellular migration, and function appears to be mediated by interaction with roundabout homolog receptors. During neural development involved in axonal navigation at the ventral midline of the neural tube and projection of axons to different regions (By similarity). SLIT1 and SLIT2 together seem to be essential for midline guidance in the forebrain by acting as repulsive signal preventing inappropriate midline crossing by axons projecting from the olfactory bulb
242	SLITRK6	9606.ENSP00000383143	SLITRK6	SLIT and NTRK-like protein 6; Regulator of neurite outgrowth required for normal hearing and vision; Belongs to the SLITRK family
243	SMNDC1	9606.ENSP00000358616	SMNDC1	Survival of motor neuron-related-splicing factor 30; Necessary for spliceosome assembly. Overexpression causes apoptosis; Belongs to the SMN family
244	SMOC2	9606.ENSP00000346537	SMOC2	SPARC-related modular calcium-binding protein 2; Promotes matrix assembly and cell adhesiveness (By similarity). Can stimulate endothelial cell proliferation, migration, as well as angiogenesis; SPARC family
245	SOCS6	9606.ENSP00000381034	SOCS6	Suppressor of cytokine signaling 6; SOCS family proteins form part of a classical negative feedback system that regulates cytokine signal transduction. May be a substrate recognition component of a SCF-like ECS (Elongin BC-CUL2/5-SOCS-box protein) E3 ubiquitin-protein ligase complex which mediates the ubiquitination and subsequent proteasomal degradation of target proteins (By similarity). Regulates KIT degradation by ubiquitination of the tyrosine-phosphorylated receptor; SH2 domain containing
246	SORBS2	9606.ENSP00000347852	SORBS2	Sorbin and SH3 domain-containing protein 2; Adapter protein that plays a role in the assembling of signaling complexes, being a link between ABL kinases and actin cytoskeleton. Can form complex with ABL1 and CBL, thus promoting ubiquitination and degradation of ABL1 or with AKT1 and PAK1, thus mediating AKT1-mediated activation of PAK1. May play a role in the regulation of pancreatic cell adhesion, possibly by acting on WASF1 phosphorylation, enhancing phosphorylation by ABL1, as well as dephosphorylation by PTPN12. Isoform 6 increases water and sodium absorption in the intestine and g [...]
247	SOWAHC	9606.ENSP00000365830	SOWAHC	Ankyrin repeat domain-containing protein SOWAHC; Sosondowah ankyrin repeat domain family member C; Belongs to the SOWAH family
248	SP9	9606.ENSP00000378418	SP9	Transcription factor Sp9; Transcription factor which plays a key role in limb development. Positively regulates FGF8 expression in the apical ectodermal ridge (AER) and contributes to limb outgrowth in embryos (By similarity); Sp transcription factors
249	SPATA31D1	9606.ENSP00000341988	SPATA31D1	Spermatogenesis-associated protein 31D1; May play a role in spermatogenesis
250	SPIB	9606.ENSP00000471921	SPIB	Transcription factor Spi-B; Sequence specific transcriptional activator which binds to the PU-box, a purine-rich DNA sequence (5'-GAGGAA-3') that can act as a lymphoid-specific enhancer. Promotes development of plasmacytoid dendritic cells (pDCs), also known as type 2 DC precursors (pre-DC2) or natural interferon (IFN)-producing cells. These cells have the capacity to produce large amounts of interferon and block viral replication. May be required for B-cell receptor (BCR) signaling, which is necessary for normal B-cell development and antigenic stimulation; ETS transcription factor family
251	ST8SIA6	9606.ENSP00000366827	ST8SIA6	Alpha-2,8-sialyltransferase 8F; Prefers O-glycans to N-glycans or glycolipids as acceptor substrates. The minimal acceptor substrate is the NeuAc-alpha-2,3(6)-Gal sequence at the non-reducing end of their carbohydrate groups (By similarity); Sialyltransferases
252	STARD7	9606.ENSP00000338030	STARD7	StAR-related lipid transfer protein 7, mitochondrial; May play a protective role in mucosal tissues by preventing exaggerated allergic responses; StAR related lipid transfer domain containing
253	STK33	9606.ENSP00000416750	STK33	Serine/threonine-protein kinase 33; Serine/threonine protein kinase which phosphorylates VIME. May play a specific role in the dynamic behavior of the intermediate filament cytoskeleton by phosphorylation of VIME (By similarity). Not essential for the survival of KRAS-dependent AML cell lines
254	STXBP2	9606.ENSP00000413606	STXBP2	Syntaxin-binding protein 2; Involved in intracellular vesicle trafficking and vesicle fusion with membranes. Contributes to the granule exocytosis machinery through interaction with soluble N- ethylmaleimide-sensitive factor attachment protein receptor (SNARE) proteins that regulate membrane fusion. Regulates cytotoxic granule exocytosis in natural killer (NK) cells
255	SUPT3H	9606.ENSP00000360515	SUPT3H	Transcription initiation protein SPT3 homolog; Probable transcriptional activator; SAGA complex
256	SYT13	9606.ENSP00000020926	SYT13	Synaptotagmin-13; May be involved in transport vesicle docking to the plasma membrane; Belongs to the synaptotagmin family
257	SYT7	9606.ENSP00000444201	SYT7	Synaptotagmin-7; Ca(2+) sensor involved in Ca(2+)-dependent exocytosis of secretory and synaptic vesicles through Ca(2+) and phospholipid binding to the C2 domain (By similarity). Ca(2+) induces binding of the C2-domains to phospholipid membranes and to assembled SNARE-complexes; both actions contribute to triggering exocytosis (By similarity). SYT7 binds Ca(2+) with high affinity and slow kinetics compared to other synaptotagmins (By similarity). Involved in Ca(2+)-triggered lysosomal exocytosis, a major component of the plasma membrane repair. Ca(2+)- regulated delivery of lysosomal [...]
258	TAGLN	9606.ENSP00000432282	TAGLN	Transgelin; Actin cross-linking/gelling protein (By similarity). Involved in calcium interactions and contractile properties of the cell that may contribute to replicative senescence; Belongs to the calponin family
259	TBC1D22A	9606.ENSP00000336724	TBC1D22A	TBC1 domain family member 22A; May act as a GTPase-activating protein for Rab family protein(s)
260	TBK1	9606.ENSP00000329967	TBK1	Serine/threonine-protein kinase TBK1; Serine/threonine kinase that plays an essential role in regulating inflammatory responses to foreign agents. Following activation of toll-like receptors by viral or bacterial components, associates with TRAF3 and TANK and phosphorylates interferon regulatory factors (IRFs) IRF3 and IRF7 as well as DDX3X. This activity allows subsequent homodimerization and nuclear translocation of the IRFs leading to transcriptional activation of pro-inflammatory and antiviral genes including IFNA and IFNB. In order to establish such an antiviral state, TBK1 form s [...]
261	TBX15	9606.ENSP00000207157	TBX15	T-box transcription factor TBX15; Probable transcriptional regulator involved in the development of the skeleton of the limb, vertebral column and head. Acts by controlling the number of mesenchymal precursor cells and chondrocytes (By similarity); T-boxes
262	TCEAL8	9606.ENSP00000361770	TCEAL8	Transcription elongation factor A protein-like 8; May be involved in transcriptional regulation; Transcription elongation factor A like family
263	TECR	9606.ENSP00000215567	TECR	Very-long-chain enoyl-CoA reductase; Catalyzes the last of the four reactions of the long- chain fatty acids elongation cycle. This endoplasmic reticulum- bound enzymatic process, allows the addition of 2 carbons to the chain of long- and very long-chain fatty acids/VLCFAs per cycle. This enzyme reduces the trans-2,3-enoyl-CoA fatty acid intermediate to an acyl-CoA that can be further elongated by entering a new cycle of elongation. Thereby, it participates in the production of VLCFAs of different chain lengths that are involved in multiple biological processes as precursors of membran [...]
264	THBS2	9606.ENSP00000355751	THBS2	Thrombospondin-2; Adhesive glycoprotein that mediates cell-to-cell and cell-to-matrix interactions. Ligand for CD36 mediating antiangiogenic properties
266	TMEM132C	9606.ENSP00000410852	TMEM132C	Transmembrane protein 132C; Protein phosphatase 1 regulatory subunits
267	TMEM167B	9606.ENSP00000342148	TMEM167B	Protein kish-B; Involved in the early part of the secretory pathway
268	TMEM170B	9606.ENSP00000368737	TMEM170B	Transmembrane protein 170B
269	TMEM217	9606.ENSP00000338164	TMEM217	Transmembrane protein 217
270	TMEM88B	9606.ENSP00000455099	TMEM88B	Transmembrane protein 88B
271	TMSB15B	9606.ENSP00000455771	ENSG00000158427	Thymosin beta 15B
272	TNXB	9606.ENSP00000407685	TNXB	Tenascin-X; Appears to mediate interactions between cells and the extracellular matrix. Substrate-adhesion molecule that appears to inhibit cell migration. Accelerates collagen fibril formation. May play a role in supporting the growth of epithelial tumors; Fibrinogen C domain containing
273	TPI1	9606.ENSP00000229270	TPI1	Triosephosphate isomerase 1; Belongs to the triosephosphate isomerase family
274	TRA	9606.ENSP00000281453	CENPU	Centromere protein U; Component of the CENPA-NAC (nucleosome-associated) complex, a complex that plays a central role in assembly of kinetochore proteins, mitotic progression and chromosome segregation. The CENPA-NAC complex recruits the CENPA-CAD (nucleosome distal) complex and may be involved in incorporation of newly synthesized CENPA into centromeres. Plays an important role in the correct PLK1 localization to the mitotic kinetochores. A scaffold protein responsible for the initial recruitment and maintenance of the kinetochore PLK1 population until its degradation. Involved in tra [...]
276	TRERF1	9606.ENSP00000439689	TRERF1	Transcriptional-regulating factor 1; Binds DNA and activates transcription of CYP11A1. Interaction with CREBBP and EP300 results in a synergistic transcriptional activation of CYP11A1; Myb/SANT domain containing
277	TRIB1	9606.ENSP00000312150	TRIB1	Tribbles homolog 1; Adapter protein involved in protein degradation by interacting with RFWD2/COP1 ubiquitin ligase. The RFWD2-binding motif is masked by autoinhibitory interactions with the protein kinase domain. Serves to alter RFWD2 substrate specificity by directing the activity of RFWD2 toward CEBPA. Binds selectively the recognition sequence of CEBPA. Regulates myeloid cell differentiation by altering the expression of CEBPA in a RFWD2- dependent manner (By similarity). Controls macrophage, eosinophil and neutrophil differentiation via the COP1-binding domain (By similarity). Int [...]
278	TRIM62	9606.ENSP00000291416	TRIM62	E3 ubiquitin-protein ligase TRIM62; E3 ubiquitin ligase whose activity is dependent on E2 ubiquitin-conjugating enzyme UBE2D2; Ring finger proteins
279	TRMT10C	9606.ENSP00000312356	TRMT10C	tRNA methyltransferase 10 homolog C; Mitochondrial tRNA N(1)-methyltransferase involved in mitochondrial tRNA maturation. Component of mitochondrial ribonuclease P, a complex composed of TRMT10C/MRPP1, HSD17B10/MRPP2 and MRPP3, which cleaves tRNA molecules in their 5'-ends. Together with HSD17B10/MRPP2, forms a subcomplex of the mitochondrial ribonuclease P, named MRPP1-MRPP2 subcomplex, which displays functions that are independent of the ribonuclease P activity. The MRPP1-MRPP2 subcomplex catalyzes the formation of N(1)-methylguanine and N(1)-methyladenine at position 9 (m1G9 and m1 [...]
281	TSC22D3	9606.ENSP00000361458	TSC22D3	TSC22 domain family protein 3; Protects T-cells from IL2 deprivation-induced apoptosis through the inhibition of FOXO3A transcriptional activity that leads to the down-regulation of the pro-apoptotic factor BCL2L11. In macrophages, plays a role in the anti-inflammatory and immunosuppressive effects of glucocorticoids and IL10. In T-cells, inhibits anti-CD3-induced NFKB1 nuclear translocation. In vitro, suppresses AP1 and NFKB1 DNA-binding activities (By similarity). Isoform 1 inhibits myogenic differentiation and mediates anti- myogenic effects of glucocorticoids by binding and regulat [...]
282	TSKU	9606.ENSP00000434847	TSKU	Tsukushin; Tsukushi, small leucine rich proteoglycan
283	TTC28	9606.ENSP00000381003	TTC28	Tetratricopeptide repeat protein 28; During mitosis, may be involved in the condensation of spindle midzone microtubules, leading to the formation of midbody; Tetratricopeptide repeat domain containing
284	TTC34	9606.ENSP00000383873	TTC34	Tetratricopeptide repeat domain containing
285	TTC9	9606.ENSP00000256367	TTC9	Tetratricopeptide repeat domain containing; Belongs to the TTC9 family
286	TTC9B	9606.ENSP00000311760	TTC9B	Tetratricopeptide repeat domain containing
287	WDPCP	9606.ENSP00000272321	WDPCP	WD repeat-containing and planar cell polarity effector protein fritz homolog; Probable effector of the planar cell polarity signaling pathway which regulates the septin cytoskeleton in both ciliogenesis and collective cell movements. Together with FUZ and WDPCP proposed to function as core component of the CPLANE (ciliogenesis and planar polarity effectors) complex involved in the recruitment of peripheral IFT-A proteins to basal bodies (By similarity); Bardet-Biedl syndrome associated
288	WDR11	9606.ENSP00000263461	WDR11	WD repeat-containing protein 11; WD repeat domain containing

Treatment

#	queryItem	stringId	preferredName	annotation
289	WLS	9606.ENSP00000346829	WLS	Protein wntless homolog; Regulates Wnt proteins sorting and secretion in a feedback regulatory mechanism. This reciprocal interaction plays a key role in the regulation of expression, subcellular location, binding and organelle-specific association of Wnt proteins. Plays also an important role in establishment of the anterior-posterior body axis formation during development (By similarity)
290	WSCD1	9606.ENSP00000460825	WSCD1	WSC domain containing 1
291	XPNPEP1	9606.ENSP00000421566	XPNPEP1	Xaa-Pro aminopeptidase 1; Contributes to the degradation of bradykinin. Catalyzes the removal of a penultimate prolyl residue from the N-termini of peptides, such as Arg-Pro-Pro; Aminopeptidases
292	XPNPEP2	9606.ENSP00000360147	XPNPEP2	Xaa-Pro aminopeptidase 2; Membrane-bound metalloprotease which catalyzes the removal of a penultimate prolyl residue from the N-termini of peptides, such as Arg-Pro-Pro. May play a role in the metabolism of the vasodilator bradykinin; Belongs to the peptidase M24B family
293	ZBTB8A	9606.ENSP00000362609	ZBTB8A	Zinc finger and BTB domain-containing protein 8A; May be involved in transcriptional regulation; BTB domain containing
294	ZNF184	9606.ENSP00000211936	ZNF184	Zinc finger protein 184; May be involved in transcriptional regulation; Zinc fingers C2H2-type
295	ZNF263	9606.ENSP00000219069	ZNF263	Zinc finger protein 263; Might play an important role in basic cellular processes as a transcriptional repressor; Belongs to the krueppel C2H2-type zinc-finger protein family
296	ZNF266	9606.ENSP00000466714	ZNF266	Zinc finger protein 266; May be involved in transcriptional regulation; Zinc fingers C2H2-type
297	ZNF507	9606.ENSP00000312277	ZNF507	Zinc finger protein 507; May be involved in transcriptional regulation; Zinc fingers C2H2-type
298	ZNF724	9606.ENSP00000413411	ZNF724P	Zinc finger protein 724; May be involved in transcriptional regulation
299	ZNF821	9606.ENSP00000398089	ZNF821	Zinc finger protein 821; May be involved in transcriptional regulation; Belongs to the krueppel C2H2-type zinc-finger protein family

Treatment

Overlap among associations (with treatment)

Gene list (from Fig. 3; main text)

ABTB2, ACTG1, ADGRD1, ALCAM, ARHGAP10, ASXL2, BOLA3, C1orf87, C9orf47, CACNA2D2, CDH5, CELF1, CFAP46, CHRNA10, COX6B2, CPLX2, DICER1, DPF3, EGFR, EPSTI1, EVI5, EYA1, GATAD2A, GK, GNAS, GPX5, GRID1, IGSF21, INPP5A, IRF8, ITGB8, KIF20B, LBX1, LRP8, LRRC28, LY6H, MAP2K4, MED13L, MICALL2, MTRNR2L1, NKX2-5, PCDHGA4, PNPLA7, PPP2R2A, PTPRN2, RELN, SDHAF3, SHANK2, SIX2, SLCO1A2, SLIT1, SOWAHC, SP9, STK33, SYT7, TBC1D22A, THBS2, TRERF1, TRIB1, WDPCP, WSCD1, ZNF263

Overlap of associations with treatment

Gene network

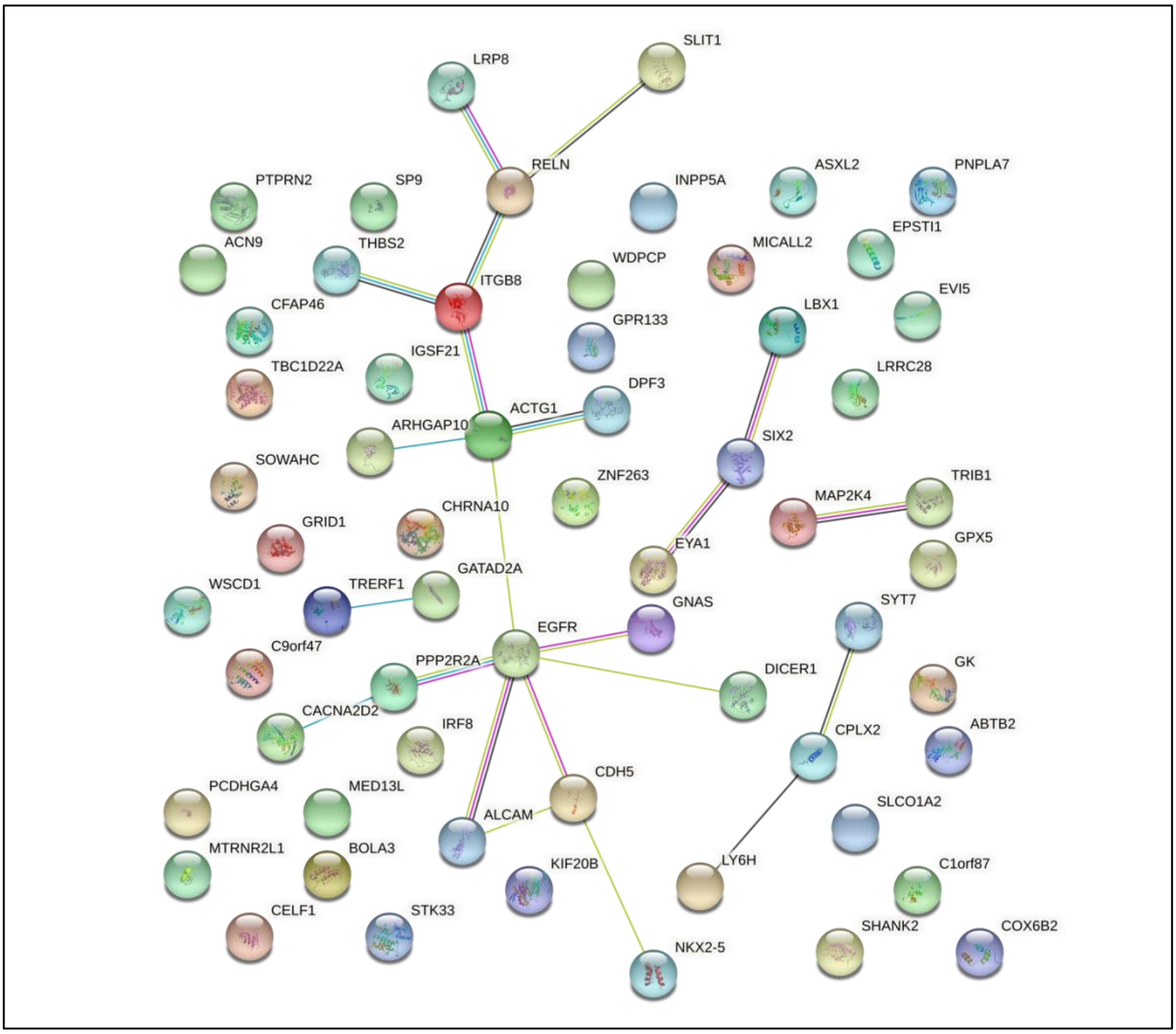

Overlap of associations with treatment

Enrichments found

Network Stats

number of nodes:	62	expected number of edges:	15
number of edges:	22	PPI enrichment p-value:	0.0636
average node degree:	0.71		
avg. local clustering coefficient:	0.297		

*your network does **not** have significantly more interactions than expected (what does that mean?)*

Biological Process (Gene Ontology)

#term ID	term description	gene count	background gene count	strength	FDR	matching proteins in your network (labels)
GO:0007275	multicellular organism development	29	4726	0.29	0.0352	ITGB8,CHRNA10,IGSF21,SLIT1,IRF8,WDPCP,EGFR,MICALL2,LRP8,SIX2,ALCAM,NKX2-5,EYA1,CDH5,CPLX2,LBX1,EVI5,GNAS,KIF20B,SP9,RELN,LY6H,MAP2K4,CELF1,DICER1,TRERF1,PCDHGA4,SHANK2,DPF3
GO:0007399	nervous system development	18	2206	0.41	0.0352	IGSF21,SLIT1,WDPCP,EGFR,MICALL2,LRP8,ALCAM,NKX2-5,EYA1,CPLX2,LBX1,KIF20B,RELN,LY6H,DICER1,PCDHGA4,SHANK2,DPF3
GO:0009653	anatomical structure morphogenesis	17	1992	0.43	0.0352	CHRNA10,SLIT1,WDPCP,EGFR,MICALL2,SIX2,ALCAM,NKX2-5,EYA1,LBX1,GNAS,KIF20B,SP9,RELN,LY6H,DICER1,ACTG1
GO:0009790	embryo development	11	890	0.59	0.0352	CHRNA10,WDPCP,EGFR,SIX2,NKX2-5,EYA1,LBX1,GNAS,KIF20B,SP9,CELF1
GO:0032502	developmental process	33	5401	0.29	0.0352	ITGB8,CHRNA10,IGSF21,SLIT1,IRF8,WDPCP,EGFR,MICALL2,LRP8,SIX2,ALCAM,PPP2R2A,NKX2-5,EYA1,CDH5,CPLX2,LBX1,EVI5,GNAS,KIF20B,SP9,PNPLA7,RELN,LY6H,MAP2K4,CACNA2D2,CELF1,DICER1,TRERF1,ACTG1,PCDHGA4,SHANK2,DPF3
GO:0032989	cellular component morphogenesis	10	720	0.64	0.0352	SLIT1,WDPCP,EGFR,MICALL2,ALCAM,NKX2-5,KIF20B,RELN,DICER1,ACTG1
GO:0038026	reelin-mediated signaling pathway	2	4	2.2	0.0352	LRP8,RELN
GO:0048562	embryonic organ morphogenesis	7	279	0.9	0.0352	CHRNA10,WDPCP,SIX2,NKX2-5,EYA1,LBX1,GNAS
GO:0048568	embryonic organ development	8	417	0.78	0.0352	CHRNA10,WDPCP,EGFR,SIX2,NKX2-5,EYA1,LBX1,GNAS
GO:0048598	embryonic morphogenesis	9	545	0.72	0.0352	CHRNA10,WDPCP,SIX2,NKX2-5,EYA1,LBX1,GNAS,KIF20B,SP9
GO:0048856	anatomical structure development	31	5085	0.28	0.0352	ITGB8,CHRNA10,IGSF21,SLIT1,IRF8,WDPCP,EGFR,MICALL2,LRP8,SIX2,ALCAM,NKX2-5,EYA1,CDH5,CPLX2,LBX1,EVI5,GNAS,KIF20B,SP9,RELN,LY6H,MAP2K4,CACNA2D2,CELF1,DICER1,TRERF1,ACTG1,PCDHGA4,SHANK2,DPF3
GO:0050804	modulation of chemical synaptic transmission	7	316	0.84	0.0352	EGFR,LRP8,CPLX2,RELN,CACNA2D2,SYT7,SHANK2
GO:2000026	regulation of multicellular organismal development	16	1876	0.43	0.0352	ITGB8,SLIT1,WDPCP,EGFR,LRP8,SIX2,TRIB1,NKX2-5,EYA1,CDH5,THBS2,LBX1,GNAS,KIF20B,RELN,DICER1
GO:2000114	regulation of establishment of cell polarity	3	21	1.65	0.0352	WDPCP,CDH5,KIF20B
GO:0007501	mesodermal cell fate specification	2	7	1.96	0.0382	SIX2,EYA1
GO:0009887	animal organ morphogenesis	10	865	0.56	0.0389	CHRNA10,SLIT1,WDPCP,EGFR,SIX2,NKX2-5,EYA1,LBX1,GNAS,LY6H
GO:0050793	regulation of developmental process	18	2416	0.37	0.0389	ITGB8,SLIT1,WDPCP,EGFR,LRP8,SIX2,TRIB1,NKX2-5,EYA1,CDH5,THBS2,LBX1,GNAS,KIF20B,ASXL2,RELN,CACNA2D2,DICER1
GO:0048846	axon extension involved in axon guidance	2	8	1.9	0.0405	SLIT1,ALCAM
GO:0042471	ear morphogenesis	4	112	1.05	0.042	CHRNA10,WDPCP,SIX2,EYA1
GO:0046164	alcohol catabolic process	3	46	1.31	0.042	INPP5A,GK,TRERF1
GO:0048731	system development	25	4144	0.28	0.042	ITGB8,CHRNA10,IGSF21,SLIT1,IRF8,WDPCP,EGFR,MICALL2,LRP8,SIX2,ALCAM,NKX2-5,EYA1,CDH5,CPLX2,LBX1,GNAS,KIF20B,RELN,LY6H,MAP2K4,DICER1,PCDHGA4,SHANK2,DPF3
GO:0016246	RNA interference	2	10	1.8	0.0473	CELF1,DICER1
GO:0051094	positive regulation of developmental process	12	1286	0.47	0.0473	ITGB8,LRP8,TRIB1,NKX2-5,CDH5,THBS2,GNAS,KIF20B,ASXL2,RELN,CACNA2D2,DICER1
GO:0055002	striated muscle cell development	4	121	1.02	0.0473	NKX2-5,MAP2K4,CACNA2D2,ACTG1
GO:0030182	neuron differentiation	10	940	0.53	0.0481	SLIT1,WDPCP,EGFR,MICALL2,ALCAM,EYA1,LBX1,KIF20B,RELN,DICER1
GO:0048468	cell development	13	1493	0.44	0.0481	SLIT1,WDPCP,EGFR,MICALL2,ALCAM,NKX2-5,KIF20B,RELN,MAP2K4,CACNA2D2,CELF1,DICER1,ACTG1
GO:0051239	regulation of multicellular organismal process	19	2788	0.33	0.0481	ITGB8,SLIT1,IRF8,WDPCP,EGFR,LRP8,SIX2,TRIB1,NKX2-5,EYA1,CDH5,THBS2,LBX1,GNAS,KIF20B,RELN,CACNA2D2,DICER1,SYT7
GO:0000902	cell morphogenesis	8	626	0.61	0.0495	SLIT1,WDPCP,EGFR,MICALL2,ALCAM,KIF20B,RELN,DICER1

Cellular Component (Gene Ontology)

#term ID	term description	gene count	background gene count	strength	FDR	matching proteins in your network (labels)
GO:0030424	axon	9	530	0.73	0.0127	CHRNA10,LRP8,ALCAM,KIF20B,PTPRN2,MAP2K4,DICER1,SYT7,SHANK2
GO:0030425	dendrite	9	531	0.73	0.0127	LRP8,ALCAM,CPLX2,GNAS,RELN,MAP2K4,DICER1,SYT7,SHANK2
GO:0036477	somatodendritic compartment	10	731	0.64	0.0127	CHRNA10,LRP8,ALCAM,CPLX2,GNAS,RELN,MAP2K4,DICER1,SYT7,SHANK2
GO:0043005	neuron projection	13	1142	0.56	0.0127	CHRNA10,MICALL2,LRP8,ALCAM,CPLX2,GNAS,KIF20B,PTPRN2,RELN,MAP2K4,DICER1,SYT7,SHANK2
GO:0043025	neuronal cell body	7	460	0.68	0.0301	CHRNA10,LRP8,ALCAM,CPLX2,MAP2K4,SYT7,SHANK2
GO:0120025	plasma membrane bounded cell projection	15	1900	0.4	0.0301	CHRNA10,WDPCP,MICALL2,LRP8,ALCAM,CPLX2,CFAP46,GNAS,KIF20B,PTPRN2,RELN,MAP2K4,DICER1,SYT7,SHANK2
GO:0150034	distal axon	5	250	0.8	0.0385	KIF20B,PTPRN2,DICER1,SYT7,SHANK2
GO:0045202	synapse	9	849	0.52	0.0389	CHRNA10,IGSF21,EGFR,LRP8,GRID1,CPLX2,PTPRN2,SYT7,SHANK2

KEGG Pathways

#term ID	term description	gene count	background gene count	strength	FDR	matching proteins in your network (labels)
hsa05414	Dilated cardiomyopathy (DCM)	4	88	1.16	0.0254	ITGB8,GNAS,CACNA2D2,ACTG1
hsa04510	Focal adhesion	5	197	0.9	0.0274	ITGB8,EGFR,THBS2,RELN,ACTG1
hsa05165	Human papillomavirus infection	6	317	0.78	0.0274	ITGB8,EGFR,PPP2R2A,THBS2,GNAS,RELN
hsa04921	Oxytocin signaling pathway	4	149	0.93	0.0432	EGFR,GNAS,CACNA2D2,ACTG1
hsa05412	Arrhythmogenic right ventricular cardiomyopathy (ARVC)	3	72	1.12	0.0432	ITGB8,CACNA2D2,ACTG1
hsa04512	ECM-receptor interaction	3	81	1.07	0.0493	ITGB8,THBS2,RELN
hsa04912	GnRH signaling pathway	3	88	1.03	0.0493	EGFR,GNAS,MAP2K4
hsa05410	Hypertrophic cardiomyopathy (HCM)	3	81	1.07	0.0493	ITGB8,CACNA2D2,ACTG1

Protein Domains (SMART)

#term ID	term description	gene count	background gene count	strength	FDR	matching proteins in your network (labels)
SM00181	Epidermal growth factor-like domain.	5	208	0.88	0.0354	ITGB8,SLIT1,LRP8,THBS2,RELN

Overlap of associations with treatment

Mapping

#	queryItem	stringId	preferredName	annotation
1	ABTB2	9606.ENSP00000410157	ABTB2	Ankyrin repeat and BTB/POZ domain-containing protein 2; May be involved in the initiation of hepatocyte growth; Ankyrin repeat domain containing
2	ACTG1	9606.ENSP00000458162	ACTG1	Actin, cytoplasmic 2; Actins are highly conserved proteins that are involved in various types of cell motility and are ubiquitously expressed in all eukaryotic cells; Belongs to the actin family
3	ADGRD1	9606.ENSP00000261654	GPR133	Adhesion G-protein coupled receptor D1; Orphan receptor. Signals via G(s)-alpha family of G- proteins. Has protumorigenic function especially in glioblastoma
4	ALCAM	9606.ENSP00000305988	ALCAM	CD166 antigen; Cell adhesion molecule that mediates both heterotypic cell-cell contacts via its interaction with CD6, as well as homotypic cell-cell contacts. Promotes T-cell activation and proliferation via its interactions with CD6. Contributes to the formation and maturation of the immunological synapse via its interactions with CD6. Mediates homotypic interactions with cells that express ALCAM. Required for normal hematopoietic stem cell engraftment in the bone marrow. Mediates attachment of dendritic cells onto endothelial cells via homotypic interaction. Inhibits endothelial cell [...]
5	ARHGAP10	9606.ENSP00000336923	ARHGAP10	Rho GTPase-activating protein 10; GTPase activator for the small GTPases RhoA and Cdc42 by converting them to an inactive GDP-bound state. Essential for PTKB2 regulation of cytoskeletal organization via Rho family GTPases. Inhibits PAK2 proteolytic fragment PAK-2p34 kinase activity and changes its localization from the nucleus to the perinuclear region. Stabilizes PAK-2p34 thereby increasing stimulation of cell death (By similarity)
6	ASXL2	9606.ENSP00000383920	ASXL2	Putative Polycomb group protein ASXL2; Putative Polycomb group (PcG) protein. PcG proteins act by forming multiprotein complexes, which are required to maintain the transcriptionally repressive state of homeotic genes throughout development. PcG proteins are not required to initiate repression, but to maintain it during later stages of development. They probably act via methylation of histones, rendering chromatin heritably changed in its expressibility (By similarity). Involved in transcriptional regulation mediated by ligand-bound nuclear hormone receptors, such as peroxisome prolife [...]
7	BOLA3	9606.ENSP00000331369	BOLA3	BolA-like protein 3; Acts as a mitochondrial iron-sulfur (Fe-S) cluster assembly factor that facilitates (Fe-S) cluster insertion into a subset of mitochondrial proteins. Probably acts together with NFU1; Belongs to the BolA/IbaG family
8	C1orf87	9606.ENSP00000360244	C1orf87	Uncharacterized protein C1orf87; Chromosome 1 open reading frame 87
9	C9orf47	9606.ENSP00000335616	C9orf47	Uncharacterized protein C9orf47; Chromosome 9 open reading frame 47
10	CACNA2D2	9606.ENSP00000418081	CACNA2D2	Voltage-dependent calcium channel subunit alpha-2/delta-2; The alpha-2/delta subunit of voltage-dependent calcium channels regulates calcium current density and activation/inactivation kinetics of the calcium channel. Acts as a regulatory subunit for P/Q-type calcium channel (CACNA1A), N-type (CACNA1B), L-type (CACNA1C OR CACNA1D) and possibly T-type (CACNA1G). Overexpression induces apoptosis
11	CDH5	9606.ENSP00000344115	CDH5	Cadherin-5; Cadherins are calcium-dependent cell adhesion proteins. They preferentially interact with themselves in a homophilic manner in connecting cells; cadherins may thus contribute to the sorting of heterogeneous cell types. This cadherin may play a important role in endothelial cell biology through control of the cohesion and organization of the intercellular junctions. It associates with alpha-catenin forming a link to the cytoskeleton. Acts in concert with KRIT1 to establish and maintain correct endothelial cell polarity and vascular lumen. These effects are mediated by recrui [...]
12	CELF1	9606.ENSP00000435926	CELF1	CUGBP Elav-like family member 1; RNA-binding protein implicated in the regulation of several post-transcriptional events. Involved in pre-mRNA alternative splicing, mRNA translation and stability. Mediates exon inclusion and/or exclusion in pre-mRNA that are subject to tissue-specific and developmentally regulated alternative splicing. Specifically activates exon 5 inclusion of cardiac isoforms of TNNT2 during heart remodeling at the juvenile to adult transition. Acts as both an activator and repressor of a pair of coregulated exons: promotes inclusion of the smooth muscle (SM) exon bu [...]
13	CFAP46	9606.ENSP00000357575	CFAP46	Cilia- and flagella-associated protein 46; As part of the central apparatus of the cilium axoneme plays a role in cilium movement; Cilia and flagella associated
14	CHRNA10	9606.ENSP00000250699	CHRNA10	Neuronal acetylcholine receptor subunit alpha-10; Ionotropic receptor with a probable role in the modulation of auditory stimuli. Agonist binding may induce an extensive change in conformation that affects all subunits and leads to opening of an ion-conducting channel across the plasma membrane. The channel is permeable to a range of divalent cations including calcium, the influx of which may activate a potassium current which hyperpolarizes the cell membrane. In the ear, this may lead to a reduction in basilar membrane motion, altering the activity of auditory nerve fibers and reducin [...]
15	COX6B2	9606.ENSP00000467266	COX6B2	Cytochrome c oxidase subunit 6B2; Connects the two COX monomers into the physiological dimeric form; Belongs to the cytochrome c oxidase subunit 6B family
16	CPLX2	9606.ENSP00000352544	CPLX2	Complexin-2; Negatively regulates the formation of synaptic vesicle clustering at active zone to the presynaptic membrane in postmitotic neurons. Positively regulates a late step in exocytosis of various cytoplasmic vesicles, such as synaptic vesicles and other secretory vesicles. Also involved in mast cell exocytosis (By similarity); Belongs to the complexin/synaphin family
17	DICER1	9606.ENSP00000437256	DICER1	Endoribonuclease Dicer; Double-stranded RNA (dsRNA) endoribonuclease playing a central role in short dsRNA-mediated post-transcriptional gene silencing. Cleaves naturally occurring long dsRNAs and short hairpin pre-microRNAs (miRNA) into fragments of twenty-one to twenty-three nucleotides with 3' overhang of two nucleotides, producing respectively short interfering RNAs (siRNA) and mature microRNAs. SiRNAs and miRNAs serve as guide to direct the RNA- induced silencing complex (RISC) to complementary RNAs to degrade them or prevent their translation. Gene silencing mediated by siRNAs, a [...]
18	DPF3	9606.ENSP00000479526	DPF3	Zinc finger protein DPF3; Belongs to the neuron-specific chromatin remodeling complex (nBAF complex). During neural development a switch from a stem/progenitor to a post-mitotic chromatin remodeling mechanism occurs as neurons exit the cell cycle and become committed to their adult state. The transition from proliferating neural stem/progenitor cells to post-mitotic neurons requires a switch in subunit composition of the npBAF and nBAF complexes. As neural progenitors exit mitosis and differentiate into neurons, npBAF complexes which contain ACTL6A/BAF53A and PHF10/BAF45A, are exchange [...]
19	EGFR	9606.ENSP00000275493	EGFR	Epidermal growth factor receptor; Receptor tyrosine kinase binding ligands of the EGF family and activating several signaling cascades to convert extracellular cues into appropriate cellular responses. Known ligands include EGF, TGFA/TGF-alpha, amphiregulin, epigen/EPGN, BTC/betacellulin, epiregulin/EREG and HBEGF/heparin-binding EGF. Ligand binding triggers receptor homo- and/or heterodimerization and autophosphorylation on key cytoplasmic residues. The phosphorylated receptor recruits adapter proteins like GRB2 which in turn activates complex downstream signaling cascades. Activates [...]
20	EPSTI1	9606.ENSP00000318982	EPSTI1	Epithelial stromal interaction 1
21	EVI5	9606.ENSP00000359356	EVI5	Ecotropic viral integration site 5 protein homolog; Functions as a regulator of cell cycle progression by stabilizing the FBXO5 protein and promoting cyclin-A accumulation during interphase. May play a role in cytokinesis
22	EYA1	9606.ENSP00000342626	EYA1	Eyes absent homolog 1; Functions both as protein phosphatase and as transcriptional coactivator for SIX1, and probably also for SIX2, SIX4 and SIX5 (By similarity). Tyrosine phosphatase that dephosphorylates 'Tyr-142' of histone H2AX (H2AXY142ph) and promotes efficient DNA repair via the recruitment of DNA repair complexes containing MDC1. 'Tyr-142' phosphorylation of histone H2AX plays a central role in DNA repair and acts as a mark that distinguishes between apoptotic and repair responses to genotoxic stress. Its function as histone phosphatase may contribute to its function in trans [...]
23	GATAD2A	9606.ENSP00000353463	GATAD2A	Transcriptional repressor p66-alpha; Transcriptional repressor. Enhances MBD2-mediated repression. Efficient repression requires the presence of GATAD2B; GATA zinc finger domain containing
24	GK	9606.ENSP00000401720	GK	Glycerol kinase; Key enzyme in the regulation of glycerol uptake and metabolism; Belongs to the FGGY kinase family
25	GNAS	9606.ENSP00000360141	GNAS	Guanine nucleotide-binding protein G(s) subunit alpha isoforms XLas; Guanine nucleotide-binding proteins (G proteins) function as transducers in numerous signaling pathways controlled by G protein-coupled receptors (GPCRs). Signaling involves the activation of adenylyl cyclases, resulting in increased levels of the signaling molecule cAMP. GNAS functions downstream of several GPCRs, including beta-adrenergic receptors. XLas isoforms interact with the same set of receptors as GNAS isoforms (By similarity)
26	GPX5	9606.ENSP00000392398	GPX5	Epididymal secretory glutathione peroxidase; Protects cells and enzymes from oxidative damage, by catalyzing the reduction of hydrogen peroxide, lipid peroxides and organic hydroperoxide, by glutathione. May constitute a glutathione peroxidase-like protective system against peroxide damage in sperm membrane lipids
27	GRID1	9606.ENSP00000330148	GRID1	Glutamate receptor ionotropic, delta-1; Receptor for glutamate. L-glutamate acts as an excitatory neurotransmitter at many synapses in the central nervous system. The postsynaptic actions of Glu are mediated by a variety of receptors that are named according to their selective agonists; Belongs to the glutamate-gated ion channel (TC 1.A.10.1) family. GRID1 subfamily
28	IGSF21	9606.ENSP00000251296	IGSF21	Immunoglobulin superfamily member 21; Immunoglobulin like domain containing
29	INPP5A	9606.ENSP00000357583	INPP5A	Type I inositol 1,4,5-trisphosphate 5-phosphatase; Major isoenzyme hydrolyzing the calcium-mobilizing second messenger Ins(1,4,5)P3, this is a signal-terminating reaction; Belongs to the inositol 1,4,5-trisphosphate 5- phosphatase type I family
30	IRF8	9606.ENSP00000268638	IRF8	Interferon regulatory factor 8; Plays a role as a transcriptional activator or repressor. Specifically binds to the upstream regulatory region of type I IFN and IFN-inducible MHC class I genes (the interferon consensus sequence (ICS)). Plays a negative regulatory role in cells of the immune system. Involved in CD8(+) dendritic cell differentiation by forming a complex with the BATF-JUNB heterodimer in immune cells, leading to recognition of AICE sequence (5'-TGAnTCA/GAAA-3'), an immune-specific regulatory element, followed by cooperative binding of BATF and IRF8 and activation of genes [...]
31	ITGB8	9606.ENSP00000222573	ITGB8	Integrin beta-8; Integrin alpha-V/beta-8 is a receptor for fibronectin
32	KIF20B	9606.ENSP00000360793	KIF20B	Kinesin-like protein KIF20B; Plus-end-directed motor enzyme that is required for completion of cytokinesis. Required for proper midbody organization and abscission in polarized cortical stem cells. Plays a role in the regulation of neuronal polarization by mediating the transport of specific cargos. Participates in the mobilization of SHTN1 and in the accumulation of PIP3 in the growth cone of primary hippocampal neurons in a tubulin and actin-dependent manner. In the developing telencephalon, cooperates with SHTN1 to promote both the transition from the multipolar to the bipolar stage [...]
33	LBX1	9606.ENSP00000359212	LBX1	Transcription factor LBX1; Transcription factor required for the development of GABAergic interneurons in the dorsal horn of the spinal cord and migration and further development of hypaxial muscle precursor cells for limb muscles, diaphragm and hypoglossal cord; NKL subclass homeoboxes and pseudogenes
34	LRP8	9606.ENSP00000303634	LRP8	Low-density lipoprotein receptor-related protein 8; Cell surface receptor for Reelin (RELN) and apolipoprotein E (apoE)-containing ligands. LRP8 participates in transmitting the extracellular Reelin signal to intracellular signaling processes, by binding to DAB1 on its cytoplasmic tail. Reelin acts via both the VLDL receptor (VLDLR) and LRP8 to regulate DAB1 tyrosine phosphorylation and microtubule function in neurons. LRP8 has higher affinity for Reelin than VLDLR. LRP8 is thus a key component of the Reelin pathway which governs neuronal layering of the forebrain during embryonic brai [...]
35	LRRC28	9606.ENSP00000304923	LRRC28	Leucine-rich repeat-containing protein 28; Leucine rich repeat containing 28
36	LY6H	9606.ENSP00000399485	LY6H	Lymphocyte antigen 6H; Believed to act as a modulator of nicotinic acetylcholine receptors (nAChRs) activity. In vitro inhibits alpha-3:beta-4-containing nAChRs maximum response. May play a role in the intracellular trafficking of alpha-7-containing nAChRs and may inhibit their expression at the cell surface. Seems to inhibit alpha-7/CHRNA7 signaling in hippocampal neurons; LY6/PLAUR domain containing
37	MAP2K4	9606.ENSP00000410402	MAP2K4	Dual specificity mitogen-activated protein kinase kinase 4; Dual specificity protein kinase which acts as an essential component of the MAP kinase signal transduction pathway. Essential component of the stress-activated protein kinase/c-Jun N-terminal kinase (SAP/JNK) signaling pathway. With MAP2K7/MKK7, is the one of the only known kinase to directly activate the stress-activated protein kinase/c-Jun N-terminal kinases MAPK8/JNK1, MAPK9/JNK2 and MAPK10/JNK3. MAP2K4/MKK4 and MAP2K7/MKK7 both activate the JNKs by phosphorylation, but they differ in their preference for the phosphorylati [...]
38	MED13L	9606.ENSP00000281928	MED13L	Mediator of RNA polymerase II transcription subunit 13-like; Component of the Mediator complex, a coactivator involved in the regulated transcription of nearly all RNA polymerase II-dependent genes. Mediator functions as a bridge to convey information from gene-specific regulatory proteins to the basal RNA polymerase II transcription machinery. Mediator is recruited to promoters by direct interactions with regulatory proteins and serves as a scaffold for the assembly of a functional preinitiation complex with RNA polymerase II and the general transcription factors. This subunit may spe [...]
39	MICALL2	9606.ENSP00000297508	MICALL2	MICAL-like protein 2; Effector of small Rab GTPases which is involved in junctional complexes assembly through the regulation of cell adhesion molecules transport to the plasma membrane and actin cytoskeleton reorganization. Regulates the endocytic recycling of occludins, claudins and E-cadherin to the plasma membrane and may thereby regulate the establishment of tight junctions and adherens junctions. In parallel, may regulate actin cytoskeleton reorganization directly through interaction with F-actin or indirectly through actinins and filamins. Most probably involved in the processes [...]
40	MTRNR2L1	9606.ENSP00000439228	MTRNR2L1	Humanin-like 1; Plays a role as a neuroprotective and antiapoptotic factor; Belongs to the humanin family
41	NKX2-5	9606.ENSP00000327758	NKX2-5	Homeobox protein Nkx-2.5; Implicated in commitment to and/or differentiation of the myocardial lineage. Acts as a transcriptional activator of ANF in cooperation with GATA4 (By similarity). Binds to the core DNA motif of NPPA promoter. It is transcriptionally controlled by PBX1 and acts as a transcriptional repressor of CDKN2B (By similarity). It is required for spleen development; NKL subclass homeoboxes and pseudogenes
42	PCDHGA4	9606.ENSP00000458570	PCDHGA4	Protocadherin gamma-A4; Potential calcium-dependent cell-adhesion protein. May be involved in the establishment and maintenance of specific neuronal connections in the brain; Clustered protocadherins
43	PNPLA7	9606.ENSP00000384610	PNPLA7	Patatin-like phospholipase domain-containing protein 7; Serine hydrolase, whose specific chemical modification by certain organophosphorus (OP) compounds leads to distal axonopathy; Patatin like phospholipase domain containing
44	PPP2R2A	9606.ENSP00000325074	PPP2R2A	Serine/threonine-protein phosphatase 2A 55 kDa regulatory subunit B alpha isoform; The B regulatory subunit might modulate substrate selectivity and catalytic activity, and also might direct the localization of the catalytic enzyme to a particular subcellular compartment; Protein phosphatase 2 regulatory subunits
45	PTPRN2	9606.ENSP00000374069	PTPRN2	Receptor-type tyrosine-protein phosphatase N2; Plays a role in vesicle-mediated secretory processes. Required for normal accumulation of secretory vesicles in hippocampus, pituitary and pancreatic islets. Required for the accumulation of normal levels of insulin-containing vesicles and preventing their degradation. Plays a role in insulin secretion in response to glucose stimuli. Required for normal accumulation of the neurotransmitters norepinephrine, dopamine and serotonin in the brain. In females, but not in males, required for normal accumulation and secretion of pituitary hormones [...]
46	RELN	9606.ENSP00000392423	RELN	Reelin; Extracellular matrix serine protease that plays a role in layering of neurons in the cerebral cortex and cerebellum. Regulates microtubule function in neurons and neuronal migration. Affects migration of sympathetic preganglionic neurons in the spinal cord, where it seems to act as a barrier to neuronal migration. Enzymatic activity is important for the modulation of cell adhesion. Binding to the extracellular domains of lipoprotein receptors VLDLR and LRP8/APOER2 induces tyrosine phosphorylation of DAB1 and modulation of TAU phosphorylation (By similarity); Belongs to the reel [...]
47	SDHAF3	9606.ENSP00000414066	ACN9	Succinate dehydrogenase assembly factor 3, mitochondrial; Plays an essential role in the assembly of succinate dehydrogenase (SDH), an enzyme complex (also referred to as respiratory complex II) that is a component of both the tricarboxylic acid (TCA) cycle and the mitochondrial electron transport chain, and which couples the oxidation of

Overlap of associations with treatment

#	queryItem	stringId	preferredName	annotation
				succinate to fumarate with the reduction of ubiquinone (coenzyme Q) to ubiquinol. Promotes maturation of the iron-sulfur protein subunit SDHB of the SDH catalytic dimer, protecting it from the deleterious effects of oxidants. May act together with SDHAF1
48	SHANK2	9606.ENSP00000469689	SHANK2	SH3 and multiple ankyrin repeat domains protein 2; Seems to be an adapter protein in the postsynaptic density (PSD) of excitatory synapses that interconnects receptors of the postsynaptic membrane including NMDA-type and metabotropic glutamate receptors, and the actin-based cytoskeleton. May play a role in the structural and functional organization of the dendritic spine and synaptic junction; Belongs to the SHANK family
49	SIX2	9606.ENSP00000304502	SIX2	Homeobox protein SIX2; Transcription factor that plays an important role in the development of several organs, including kidney, skull and stomach. During kidney development, maintains cap mesenchyme multipotent nephron progenitor cells in an undifferentiated state by opposing the inductive signals emanating from the ureteric bud and cooperates with WNT9B to promote renewing progenitor cells proliferation. Acts through its interaction with TCF7L2 and OSR1 in a canonical Wnt signaling independent manner preventing transcription of differentiation genes in cap mesenchyme such as WNT4. Al [...]
50	SLCO1A2	9606.ENSP00000305974	SLCO1A2	Solute carrier organic anion transporter family member 1A2; Mediates the Na(+)-independent transport of organic anions such as sulfobromophthalein (BSP) and conjugated (taurocholate) and unconjugated (cholate) bile acids (By similarity). Selectively inhibited by the grapefruit juice component naringin; Solute carriers
51	SLIT1	9606.ENSP00000266058	SLIT1	Slit homolog 1 protein; Thought to act as molecular guidance cue in cellular migration, and function appears to be mediated by interaction with roundabout homolog receptors. During neural development involved in axonal navigation at the ventral midline of the neural tube and projection of axons to different regions (By similarity). SLIT1 and SLIT2 together seem to be essential for midline guidance in the forebrain by acting as repulsive signal preventing inappropriate midline crossing by axons projecting from the olfactory bulb
52	SOWAHC	9606.ENSP00000365830	SOWAHC	Ankyrin repeat domain-containing protein SOWAHC; Sosondowah ankyrin repeat domain family member C; Belongs to the SOWAH family
53	SP9	9606.ENSP00000378418	SP9	Transcription factor Sp9; Transcription factor which plays a key role in limb development. Positively regulates FGF8 expression in the apical ectodermal ridge (AER) and contributes to limb outgrowth in embryos (By similarity); Sp transcription factors
54	STK33	9606.ENSP00000416750	STK33	Serine/threonine-protein kinase 33; Serine/threonine protein kinase which phosphorylates VIME. May play a specific role in the dynamic behavior of the intermediate filament cytoskeleton by phosphorylation of VIME (By similarity). Not essential for the survival of KRAS-dependent AML cell lines
55	SYT7	9606.ENSP00000444201	SYT7	Synaptotagmin-7; Ca(2+) sensor involved in Ca(2+)-dependent exocytosis of secretory and synaptic vesicles through Ca(2+) and phospholipid binding to the C2 domain (By similarity). Ca(2+) induces binding of the C2-domains to phospholipid membranes and to assembled SNARE-complexes; both actions contribute to triggering exocytosis (By similarity). SYT7 binds Ca(2+) with high affinity and slow kinetics compared to other synaptotagmins (By similarity). Involved in Ca(2+)-triggered lysosomal exocytosis, a major component of the plasma membrane repair. Ca(2+)- regulated delivery of lysosomal [...]
56	TBC1D22A	9606.ENSP00000336724	TBC1D22A	TBC1 domain family member 22A; May act as a GTPase-activating protein for Rab family protein(s)
57	THBS2	9606.ENSP00000355751	THBS2	Thrombospondin-2; Adhesive glycoprotein that mediates cell-to-cell and cell-to-matrix interactions. Ligand for CD36 mediating antiangiogenic properties
58	TRERF1	9606.ENSP00000439689	TRERF1	Transcriptional-regulating factor 1; Binds DNA and activates transcription of CYP11A1. Interaction with CREBBP and EP300 results in a synergistic transcriptional activation of CYP11A1; Myb/SANT domain containing
59	TRIB1	9606.ENSP00000312150	TRIB1	Tribbles homolog 1; Adapter protein involved in protein degradation by interacting with RFWD2/COP1 ubiquitin ligase. The RFWD2-binding motif is masked by autoinhibitory interactions with the protein kinase domain. Serves to alter RFWD2 substrate specificity by directing the activity of RFWD2 toward CEBPA. Binds selectively the recognition sequence of CEBPA. Regulates myeloid cell differentiation by altering the expression of CEBPA in a RFWD2- dependent manner (By similarity). Controls macrophage, eosinophil and neutrophil differentiation via the COP1-binding domain (By similarity). Int [...]
60	WDPCP	9606.ENSP00000272321	WDPCP	WD repeat-containing and planar cell polarity effector protein fritz homolog; Probable effector of the planar cell polarity signaling pathway which regulates the septin cytoskeleton in both ciliogenesis and collective cell movements. Together with FUZ and WDPCP proposed to function as core component of the CPLANE (ciliogenesis and planar polarity effectors) complex involved in the recruitment of peripheral IFT-A proteins to basal bodies (By similarity); Bardet-Biedl syndrome associated
61	WSCD1	9606.ENSP00000460825	WSCD1	WSC domain containing 1
62	ZNF263	9606.ENSP00000219069	ZNF263	Zinc finger protein 263; Might play an important role in basic cellular processes as a transcriptional repressor; Belongs to the krueppel C2H2-type zinc-finger protein family

Overlap of associations with treatment

PANTHER output

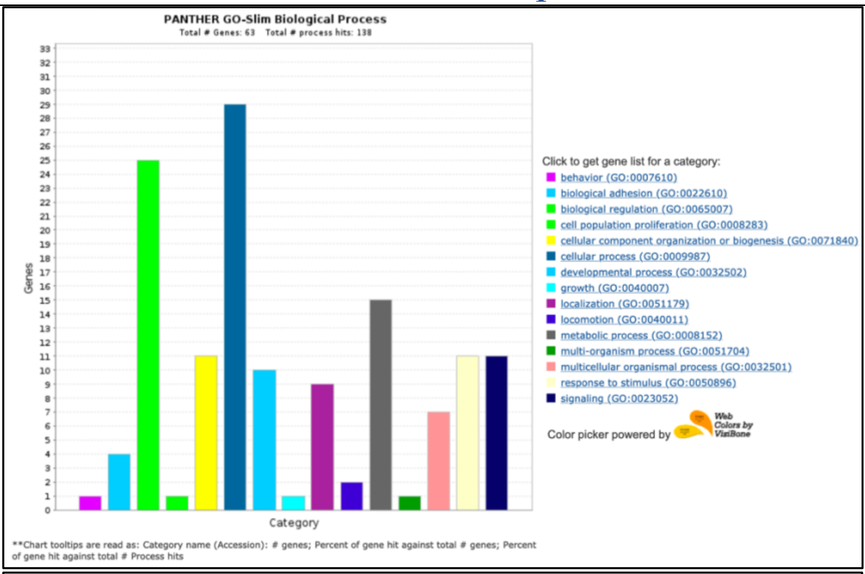

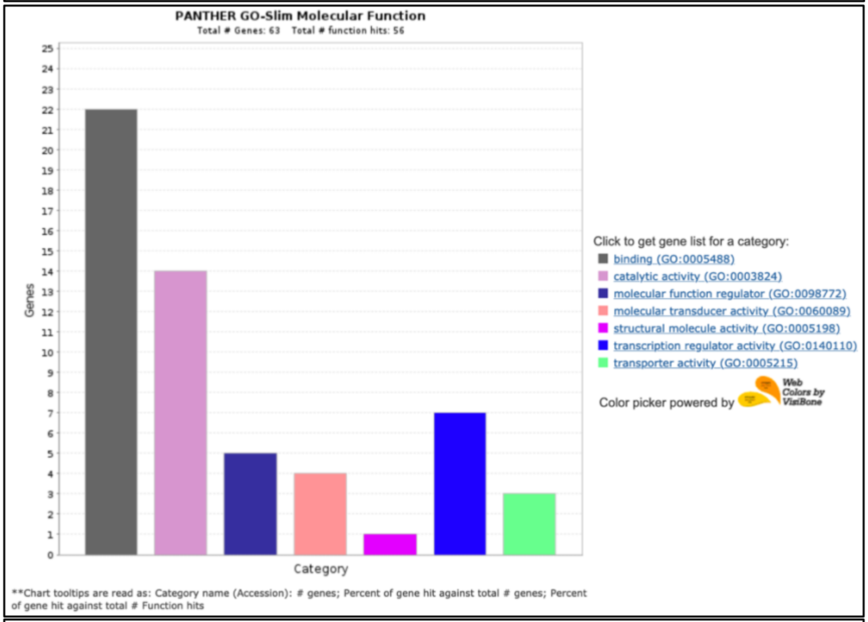

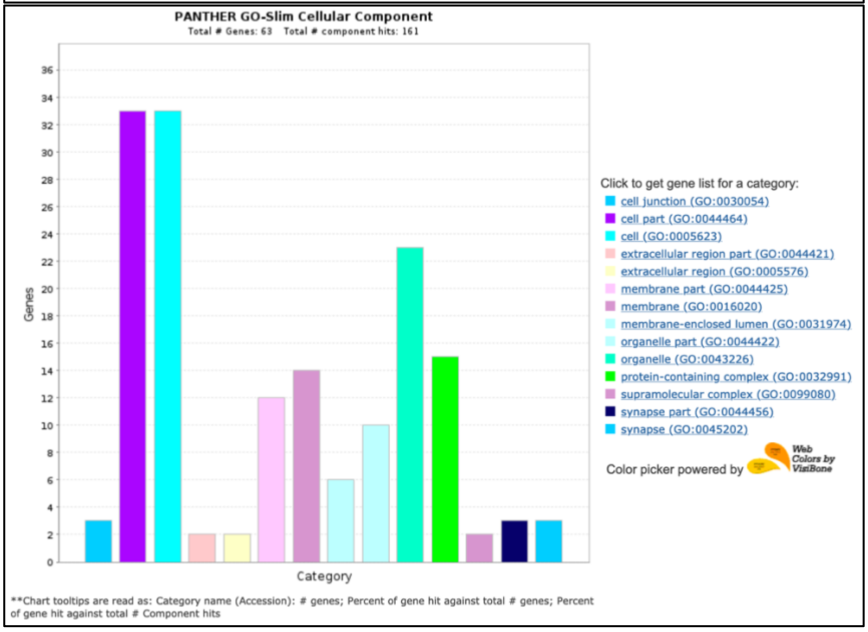

Overlap of associations with treatment

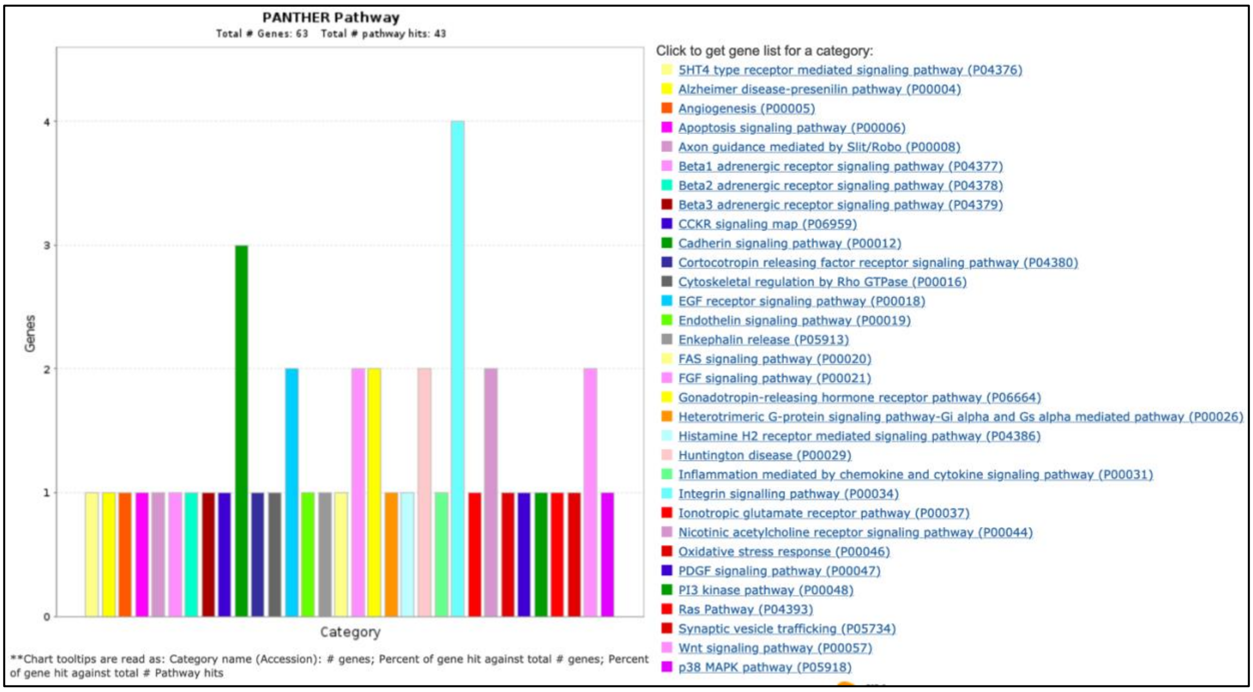

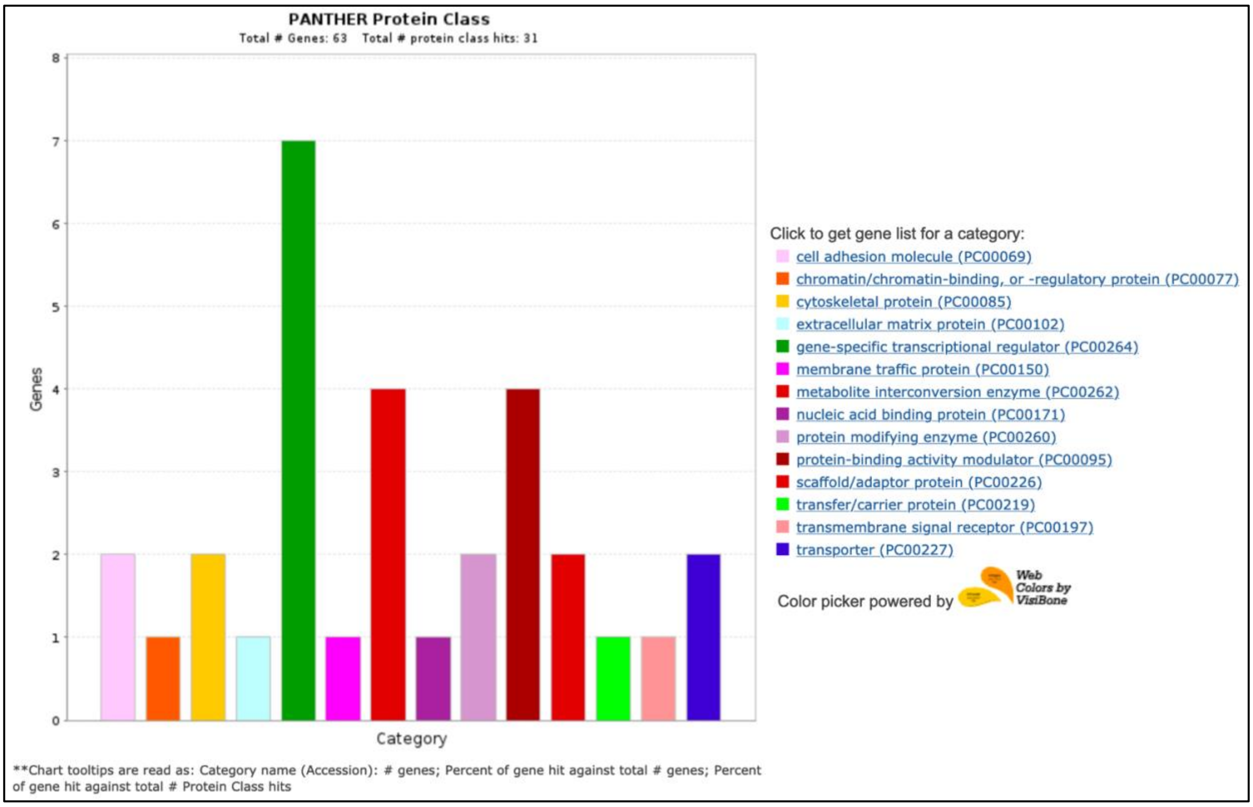

Overlap of associations with treatment

List output

Gene ID	Mapped ID	Gene name/symbol	Protein class	GO database MF complete	GO database BP complete	GO database CC complete	Pathway	Reactome pathway
HUMAN|HGNC=14568|UniProtKB=Q9BYT3	STK33	Serine/threonine-protein kinase 33		protein kinase activity(GO:0004672);protein serine/threonine kinase activity(GO:0004674);ATP binding(GO:0005524);protein serine kinase activity(GO:0106310);protein threonine kinase activity(GO:0106311)	protein phosphorylation(GO:0006468);signal transduction in response to DNA damage(GO:0042770);mitotic DNA damage checkpoint(GO:0044773);protein autophosphorylation(GO:0046777)	nucleus(GO:0005634);cytoplasm(GO:0005737);perinuclear region of cytoplasm(GO:0048471)		
HUMAN|HGNC=29060|UniProtKB=Q658N2	WSCD1	WSC domain-containing protein 1		sulfotransferase activity(GO:0008146)		membrane(GO:0016020);integral component of membrane(GO:0016021)		
HUMAN|HGNC=24380|UniProtKB=Q6YFQ2	COX6B2	Cytochrome c oxidase subunit 6B2		protein binding(GO:0005515)	oxidative phosphorylation(GO:0006119)	mitochondrion(GO:0005739);mitochondrial crista(GO:0030061)		
HUMAN|HGNC=1764|UniProtKB=P33151	CDH5	Cadherin-5		signaling receptor binding(GO:0005102);calcium ion binding(GO:0005509);protein binding(GO:0005515);beta-catenin binding(GO:0008013);protein phosphatase binding(GO:0019903);vascular endothelial growth factor receptor 2 binding(GO:0043184);ion channel binding(GO:0044325);cadherin binding(GO:0045296);fibrinogen binding(GO:0070051);BMP receptor binding(GO:0070700);protein tyrosine kinase binding(GO:1990782)	cell morphogenesis(GO:0000902);regulation of protein phosphorylation(GO:0001932);vasculature development(GO:0001944);blood vessel maturation(GO:0001955);cellular calcium ion homeostasis(GO:0006874);cell-cell junction assembly(GO:0007043);homophilic cell adhesion via plasma membrane adhesion molecules(GO:0007156);transforming growth factor beta receptor signaling pathway(GO:0007179);multicellular organism development(GO:0007275);negative regulation of cell population proliferation(GO:0008285);positive regulation of gene expression(GO:0010628);calcium-dependent cell-cell adhesion via plasma membrane cell adhesion molecules(GO:0016339);positive regulation of cell migration(GO:0030335);positive regulation of BMP signaling pathway(GO:0030513);negative regulation of microtubule polymerization(GO:0031115);positive regulation of protein-containing complex assembly(GO:0031334);adherens junction organization(GO:0034332);positive regulation of protein dephosphorylation(GO:0035307);maintenance of blood-brain barrier(GO:0035633);regulation of vascular permeability(GO:0043114);blood vessel endothelial cell migration(GO:0043534);cell-cell adhesion mediated by cadherin(GO:0044331);positive regulation of angiogenesis(GO:0045766);negative regulation of inflammatory response(GO:0050728);bicellular tight junction assembly(GO:0070830);cell-cell adhesion(GO:0098609);cell-cell adhesion via plasma-membrane adhesion molecules(GO:0098742);protein localization to bicellular tight junction(GO:1902396);positive regulation of establishment of endothelial barrier(GO:1903142);regulation of establishment of cell polarity(GO:2000114);negative regulation of endothelial cell apoptotic process(GO:2000352)	nucleoplasm(GO:0005654);plasma membrane(GO:0005886);cell-cell junction(GO:0005911);adherens junction(GO:0005912);bicellular tight junction(GO:0005923);external side of plasma membrane(GO:0009897);cell surface(GO:0009986);membrane(GO:0016020);integral component of membrane(GO:0016021);catenin complex(GO:0016342);cell junction(GO:0030054);nuclear membrane(GO:0031965)	Cadherin signaling pathway->Cadherin;Wnt signaling pathway->Cadherin;	Signal Transduction;Cell-Cell communication;Signaling by Receptor Tyrosine Kinases;Cell junction organization;Cell-cell junction organization;Adherens junctions interactions;VEGFR2 mediated vascular permeability;VEGFA-VEGFR2 Pathway;Signaling by VEGF
HUMAN|HGNC=28027|UniProtKB=O95876	WDPCP	WD repeat-containing and planar cell polarity effector protein fritz homolog			kidney development(GO:0001822);auditory receptor cell morphogenesis(GO:0002093);smoothened signaling pathway(GO:0007224);nervous system development(GO:0007399);regulation of fibroblast migration(GO:0010762);regulation of embryonic cell shape(GO:0016476);cell projection organization(GO:0030030);septin cytoskeleton organization(GO:0032185);regulation of protein localization(GO:0032880);embryonic digit morphogenesis(GO:0042733);camera-type eye development(GO:0043010);tongue morphogenesis(GO:0043587);cilium organization(GO:0044782);establishment of protein localization(GO:0045184);regulation of focal adhesion assembly(GO:0051893);digestive system development(GO:0055123);roof of mouth development(GO:0060021);cilium assembly(GO:0060271);respiratory system development(GO:0060541);glomerular visceral epithelial cell migration(GO:0090521);regulation of ruffle assembly(GO:1900027);regulation of establishment of cell polarity(GO:2000114)	cytoplasm(GO:0005737);cytoskeleton(GO:0005856);plasma membrane(GO:0005886);cilium(GO:0005929);axoneme(GO:0005930);cell cortex(GO:0005938);apical plasma membrane(GO:0016324);axonemal basal plate(GO:0097541)		
HUMAN|HGNC=26099|UniProtKB=A1A4S6	ARHGAP10	Rho GTPase-activating protein 10	GTPase-activating protein(PC00257)	GTPase activator activity(GO:0005096);protein binding(GO:0005515)	cytoskeleton organization(GO:0007010);signal transduction(GO:0007165);negative regulation of apoptotic process(GO:0043066);positive regulation of GTPase activity(GO:0043547);regulation of small GTPase mediated signal transduction(GO:0051056)	cytosol(GO:0005829);plasma membrane(GO:0005886);perinuclear region of cytoplasm(GO:0048471)	Integrin signalling pathway->GTPase regulator associated with FAK;PDGF signaling pathway->GTPase-activating protein Rho;	Programmed Cell Death;Apoptosis;Signal Transduction;Regulation of PAK-2p34 activity by PS-GAP/RHG10;Signaling by Rho GTPases;Rho GTPase cycle;Regulation of Apoptosis
HUMAN|HGNC=24768|UniProtKB=Q6ZV29	PNPLA7	Patatin-like phospholipase domain-containing protein 7	esterase(PC00097)	lysophospholipase activity(GO:0004622);phosphatidyl phospholipase B activity(GO:0102545)	phosphatidylcholine catabolic process(GO:0034638)	endoplasmic reticulum(GO:0005783);endoplasmic reticulum membrane(GO:0005789);lipid droplet(GO:0005811);integral component of membrane(GO:0016021)		Phospholipid metabolism;Metabolism of lipids;PI Metabolism;Glycerophospholipid catabolism;Metabolism
HUMAN|HGNC=4392|UniProtKB=Q5JWF2	GNAS	Guanine nucleotide-binding protein G(s) subunit alpha isoforms Xlas	heterotrimeric G-protein(PC00117)	G protein-coupled receptor binding(GO:0001664);molecular_function(GO:0003674);GTPase activity(GO:0003924);insulin-like growth factor receptor binding(GO:0005159);protein binding(GO:0005515);GTP binding(GO:0005525);adenylate cyclase activator activity(GO:0010856);guanyl nucleotide binding(GO:0019001);G-protein beta/gamma-subunit complex	tissue homeostasis(GO:0001894);endochondral ossification(GO:0001958);renal water homeostasis(GO:0003091);energy reserve metabolic process(GO:0006112);DNA methylation(GO:0006306);G protein-coupled receptor signaling pathway(GO:0007186);adenylate cyclase-modulating G protein-coupled receptor signaling pathway(GO:0007188);adenylate cyclase-activating G	ruffle(GO:0001726);extracellular region(GO:0005576);nucleus(GO:0005634);cytoplasm(GO:0005737);cytosol(GO:0005829);heterotrimeric G-protein complex(GO:0005834);plasma membrane(GO:0005886);membrane(GO:0016020);integral component of membrane(GO:0016021);apical plasma membrane(GO:0016324);transport vesicle(GO:0030133);dendrite(GO:0030425);intrinsic component of	Beta3 adrenergic receptor signaling pathway->G-Protein;Beta1 adrenergic receptor signaling pathway-	Hemostasis;Hedgehog 'off' state;Signaling by GPCR;G alpha (z) signalling events;Vasopressin regulates renal water

Overlap of associations with treatment

Gene ID	Mapped ID	Gene name/symbol	Protein class	GO database MF complete	GO database BP complete	GO database CC complete	Pathway	Reactome pathway
				binding(GO:0031683);beta-2 adrenergic receptor binding(GO:0031698);D1 dopamine receptor binding(GO:0031748);mu-type opioid receptor binding(GO:0031852);ionotropic glutamate receptor binding(GO:0035255);metal ion binding(GO:0046872);corticotropin-releasing hormone receptor 1 binding(GO:0051430)	protein-coupled receptor signaling pathway(GO:0007189);activation of adenylate cyclase activity(GO:0007190);adenylate cyclase-activating dopamine receptor signaling pathway(GO:0007191);female pregnancy(GO:0007565);sensory perception of chemical stimulus(GO:0007606);sensory perception of smell(GO:0007608);protein secretion(GO:0009306);regulation of signal transduction(GO:0009966);embryonic hindlimb morphogenesis(GO:0035116);multicellular organism growth(GO:0035264);negative regulation of multicellular organism growth(GO:0040015);post-embryonic body morphogenesis(GO:0040032);response to drug(GO:0042493);positive regulation of catalytic activity(GO:0043085);positive regulation of GTPase activity(GO:0043547);skin development(GO:0043588);positive regulation of cAMP-mediated signaling(GO:0043950);positive regulation of osteoblast differentiation(GO:0045669);positive regulation of osteoclast differentiation(GO:0045672);intracellular transport(GO:0046907);developmental growth(GO:0048589);embryonic cranial skeleton morphogenesis(GO:0048701);regulation of insulin secretion(GO:0050796);cognition(GO:0050890);cartilage development(GO:0051216);bone development(GO:0060348);hair follicle placode formation(GO:0060789);platelet aggregation(GO:0070527);response to parathyroid hormone(GO:0071107);cellular response to glucagon stimulus(GO:0071377);cellular response to prostaglandin E stimulus(GO:0071380);genetic imprinting(GO:0071514);cellular response to catecholamine stimulus(GO:0071870);adenylate cyclase-activating adrenergic receptor signaling pathway(GO:0071880);positive regulation of cold-induced thermogenesis(GO:0120162);regulation of parathyroid hormone secretion(GO:2000828)	membrane(GO:0031224);trans-Golgi network membrane(GO:0032588);perinuclear region of cytoplasm(GO:0048471);extracellular exosome(GO:0070062)	>G-Protein;Gonadotropin-releasing hormone receptor pathway->gnas;5HT4 type receptor mediated signaling pathway->G-protein;Enkephalin release->Gs-protein;Endothelin signaling pathway->stimulatory G protein;Histamine H2 receptor mediated signaling pathway->G-Protein;Beta2 adrenergic receptor signaling pathway->G-Protein;Heterotrimeric G-protein signaling pathway-Gi alpha and Gs alpha mediated pathway->Gs alpha subunit;Cortocotropin releasing factor receptor signaling pathway->G-Protein;	homeostasis via Aquaporins;Glucagon-like Peptide-1 (GLP1) regulates insulin secretion;Transport of small molecules;G alpha (i) signalling events;Signaling by Hedgehog;GPCR downstream signalling;Metabolism;Integration of energy metabolism;Platelet homeostasis;Regulation of insulin secretion;PKA activation in glucagon signalling;Prostacyclin signalling through prostacyclin receptor;Glucagon signaling in metabolic regulation;GPCR ligand binding;Signal Transduction;G alpha (s) signalling events;Glucagon-type ligand receptors;Aquaporin-mediated transport;Class B/2 (Secretin family receptors)
HUMAN|HGNC=16465|UniProtKB=Q96J88	EPSTI1	Epithelial-stromal interaction protein 1		protein binding(GO:0005515)				
HUMAN|HGNC=28355|UniProtKB=Q86X40	LRRC28	Leucine-rich repeat-containing protein 28	scaffold/adaptor protein(PC00226)					
HUMAN|HGNC=2310|UniProtKB=Q6PUV4	CPLX2	Complexin-2		SNARE binding(GO:0000149);protein binding(GO:0005515);syntaxin-1 binding(GO:0017075);syntaxin binding(GO:0019905);calcium-dependent protein binding(GO:0048306)	neurotransmitter transport(GO:0006836);exocytosis(GO:0006887);vesicle docking involved in exocytosis(GO:0006904);nervous system development(GO:0007399);synaptic vesicle exocytosis(GO:0016079);regulation of exocytosis(GO:0017157);cell differentiation(GO:0030154);regulation of synaptic vesicle fusion to presynaptic active zone membrane(GO:0031630);positive regulation of synaptic plasticity(GO:0031915);mast cell degranulation(GO:0043303);regulation of neurotransmitter secretion(GO:0046928)	nucleus(GO:0005634);cytosol(GO:0005829);dendrite(GO:0030425);SNARE complex(GO:0031201);terminal bouton(GO:0043195);perikaryon(GO:0043204);synapse(GO:0045202);synaptobrevin 2-SNAP-25-syntaxin-1a-complexin II complex(GO:0070033);synaptobrevin 2-SNAP-25-syntaxin-3-complexin complex(GO:0070554);glutamatergic synapse(GO:0098978)		
HUMAN|HGNC=6076|UniProtKB=Q14642	INPP5A	Type I inositol 1,4,5-trisphosphate 5-phosphatase	phosphatase(PC00181)	inositol-polyphosphate 5-phosphatase activity(GO:0004445);protein binding(GO:0005515);PH domain binding(GO:0042731);inositol-1,4,5-trisphosphate 5-phosphatase activity(GO:0052658);inositol-1,3,4,5-tetrakisphosphate 5-phosphatase activity(GO:0052659)	dephosphorylation(GO:0016311);inositol phosphate metabolic process(GO:0043647);inositol phosphate dephosphorylation(GO:0046855);phosphatidylinositol dephosphorylation(GO:0046856);inositol phosphate-mediated signaling(GO:0048016);negative regulation of neuron death(GO:1901215)	plasma membrane(GO:0005886);membrane(GO:0016020);dendrite(GO:0030425)	PI3 kinase pathway->SH2 domain-containing inositol 5-phosphatase;	Synthesis of IP2, IP, and Ins in the cytosol;Inositol phosphate metabolism;Metabolism
HUMAN|HGNC=3501|UniProtKB=O60447	EVI5	Ecotropic viral integration site 5 protein homolog	GTPase-activating protein(PC00257)	GTPase activator activity(GO:0005096);protein binding(GO:0005515);Rab GTPase binding(GO:0017137)	intracellular protein transport(GO:0006886);cell cycle(GO:0007049);retrograde transport, endosome to Golgi(GO:0042147);positive regulation of GTPase activity(GO:0043547);cell division(GO:0051301);activation of GTPase activity(GO:0090630)	nucleus(GO:0005634);microtubule organizing center(GO:0005815);spindle(GO:0005819);cytosol(GO:0005829)		
HUMAN|HGNC=5358|UniProtKB=Q02556	IRF8	Interferon regulatory factor 8	winged helix/forkhead transcription factor(PC00246)	transcription regulatory region sequence-specific DNA binding(GO:0000976);RNA polymerase II cis-regulatory region sequence-specific DNA binding(GO:0000978);DNA-binding transcription factor activity, RNA polymerase II-specific(GO:0000981);DNA-binding transcription repressor activity, RNA polymerase II-specific(GO:0001227);DNA-binding transcription factor activity(GO:0003700);protein binding(GO:0005515);sequence-specific double-stranded DNA binding(GO:1990837)	negative regulation of transcription by RNA polymerase II(GO:0000122);plasmacytoid dendritic cell differentiation(GO:0002273);immune system process(GO:0002376);regulation of transcription, DNA-templated(GO:0006355);regulation of transcription by RNA polymerase II(GO:0006357);phagocytosis(GO:0006909);autophagy(GO:0006914);immune response(GO:0006955);myeloid cell differentiation(GO:0030099);regulation of type I interferon production(GO:0032479);positive regulation of interferon-gamma production(GO:0032729);positive regulation of interleukin-12 production(GO:0032735);defense response to bacterium(GO:0042742);defense response to protozoan(GO:0042832);positive regulation of transcription by RNA polymerase II(GO:0045944);interferon-gamma-mediated signaling pathway(GO:0060333);type I interferon signaling pathway(GO:0060337);cellular response to lipopolysaccharide(GO:0071222);cellular response to interferon-gamma(GO:0071346);dendritic cell differentiation(GO:0097028)	nuclear chromatin(GO:0000790);nucleus(GO:0005634);nucleoplasm(GO:0005654);cytoplasm(GO:0005737);cytosol(GO:0005829)		Immune System;Interferon gamma signaling;Cytokine Signaling in Immune system;Interferon alpha/beta signaling;Interferon Signaling
HUMAN|HGNC=30690|UniProtKB=P0CG40	SP9	Transcription factor Sp9	C2H2 zinc finger transcription factor(PC00248)	RNA polymerase II cis-regulatory region sequence-specific DNA binding(GO:0000978);DNA-binding transcription factor activity, RNA polymerase II-specific(GO:0000981);metal ion binding(GO:0046872);sequence-specific double-stranded DNA binding(GO:1990837)	regulation of transcription by RNA polymerase II(GO:0006357);embryonic limb morphogenesis(GO:0030326)	nuclear chromatin(GO:0000790)		
HUMAN|HGNC=4289|UniProtKB=P32189	GK	Glycerol kinase	carbohydrate kinase(PC00065)	glycerol kinase activity(GO:0004370);protein binding(GO:0005515);ATP binding(GO:0005524);phosphotransferase activity, alcohol group as acceptor(GO:0016773)	carbohydrate metabolic process(GO:0005975);glycerol metabolic process(GO:0006071);triglyceride metabolic process(GO:0006641);phosphorylation(GO:0016310);triglyceride biosynthetic process(GO:0019432);glycerol catabolic	mitochondrion(GO:0005739);mitochondrial outer membrane(GO:0005741);cytosol(GO:0005829);extracellular exosome(GO:0070062)		Metabolism of lipids;Triglyceride biosynthesis;Triglyceride metabolism;Metabolism

Overlap of associations with treatment

Gene ID	Mapped ID	Gene name/symbol	Protein class	GO database MF complete	GO database BP complete	GO database CC complete	Pathway	Reactome pathway
					process(GO:0019563);glycerol-3-phosphate biosynthetic process(GO:0046167)			
HUMAN|HGNC=37155|UniProtKB=P0CJ68	MTRNR2L1	Humanin-like 1		receptor antagonist activity(GO:0048019)	negative regulation of execution phase of apoptosis(GO:1900118);negative regulation of signaling receptor activity(GO:2000272)	extracellular region(GO:0005576);cytoplasm(GO:0005737)		
HUMAN|HGNC=6728|UniProtKB=O94772	LY6H	Lymphocyte antigen 6H		protein binding(GO:0005515);acetylcholine receptor inhibitor activity(GO:0030550);acetylcholine receptor binding(GO:0033130)	nervous system development(GO:0007399);animal organ morphogenesis(GO:0009887);acetylcholine receptor signaling pathway(GO:0095500);negative regulation of signaling receptor activity(GO:2000272)	extracellular region(GO:0005576);plasma membrane(GO:0005886);anchored component of membrane(GO:0031225);synapse(GO:0045202)		Post-translational modification: synthesis of GPI-anchored proteins;Post-translational protein modification;Metabolism of proteins
HUMAN|HGNC=17427|UniProtKB=Q92784	DPF3	Zinc finger protein DPF3	histone modifying enzyme(PC00261)	transcription coregulator activity(GO:0003712);zinc ion binding(GO:0008270);histone binding(GO:0042393)	chromatin organization(GO:0006325);nervous system development(GO:0007399);biological_process(GO:0008150);negative regulation of transcription, DNA-templated(GO:0045892);positive regulation of transcription by RNA polymerase II(GO:0045944)	nuclear chromatin(GO:0000790);nucleoplasm(GO:0005654);nBAF complex(GO:0071565)		
HUMAN|HGNC=2488|UniProtKB=P52952	NKX2-5	Homeobox protein Nkx-2.5	homeodomain transcription factor(PC00119)	transcription regulatory region sequence-specific DNA binding(GO:0000976);RNA polymerase II cis-regulatory region sequence-specific DNA binding(GO:0000978);DNA-binding transcription factor activity, RNA polymerase II-specific(GO:0000981);DNA-binding transcription activator activity, RNA polymerase II-specific(GO:0001228);DNA binding(GO:0003677);chromatin binding(GO:0003682);DNA-binding transcription factor activity(GO:0003700);protein binding(GO:0005515);transcription factor binding(GO:0008134);protein homodimerization activity(GO:0042803);sequence-specific DNA binding(GO:0043565);sequence-specific double-stranded DNA binding(GO:1990837)	negative regulation of transcription by RNA polymerase II(GO:0000122);vasculogenesis(GO:0001570);heart looping(GO:0001947);heart morphogenesis(GO:0003007);outflow tract septum morphogenesis(GO:0003148);cardiac conduction system development(GO:0003161);bundle of His development(GO:0003166);Purkinje myocyte differentiation(GO:0003168);cardiac ventricle formation(GO:0003211);right ventricular cardiac muscle tissue morphogenesis(GO:0003221);ventricular trabecula myocardium morphogenesis(GO:0003222);apoptotic process involved in heart morphogenesis(GO:0003278);septum secundum development(GO:0003285);proepicardium development(GO:0003342);pulmonary myocardium development(GO:0003350);regulation of transcription by RNA polymerase II(GO:0006357);heart development(GO:0007507);adult heart development(GO:0007512);positive regulation of cell population proliferation(GO:0008284);negative regulation of cardiac muscle cell apoptotic process(GO:0010667);positive regulation of transcription via serum response element binding(GO:0010735);positive regulation of sodium ion transport(GO:0010765);negative regulation of myotube differentiation(GO:0010832);hemopoiesis(GO:0030097);cell differentiation(GO:0030154);thyroid gland development(GO:0030878);embryonic heart tube development(GO:0035050);negative regulation of apoptotic process(GO:0043066);sarcomere organization(GO:0045214);positive regulation of neuron differentiation(GO:0045666);positive regulation of heart contraction(GO:0045823);negative regulation of transcription, DNA-templated(GO:0045892);positive regulation of transcription, DNA-templated(GO:0045893);positive regulation of transcription by RNA polymerase II(GO:0045944);spleen development(GO:0048536);positive regulation of cardioblast differentiation(GO:0051891);ventricular cardiac myofibril assembly(GO:0055005);cardiac muscle cell differentiation(GO:0055007);cardiac muscle tissue morphogenesis(GO:0055008);atrial cardiac muscle cell development(GO:0055014);ventricular cardiac muscle cell development(GO:0055015);regulation of cardiac muscle contraction(GO:0055117);pharyngeal system development(GO:0060037);cardiac muscle cell proliferation(GO:0060038);regulation of cardiac muscle cell proliferation(GO:0060043);cardiac muscle contraction(GO:0060048);positive regulation of transcription initiation from RNA polymerase II promoter(GO:0060261);heart trabecula formation(GO:0060347);ventricular septum morphogenesis(GO:0060412);atrial septum morphogenesis(GO:0060413);atrioventricular node cell development(GO:0060928);atrioventricular node cell fate commitment(GO:0060929);embryonic heart tube left/right pattern formation(GO:0060971);negative regulation of canonical Wnt signaling pathway(GO:0090090);regulation of cardiac conduction(GO:1903779)	nuclear chromatin(GO:0000790);nucleus(GO:0005634);transcription regulator complex(GO:0005667);cytoplasm(GO:0005737);protein-containing complex(GO:0032991);protein-DNA complex(GO:0032993);RNA polymerase II transcription regulator complex(GO:0090575)		Cardiac conduction;YAP1- and WWTR1 (TAZ)-stimulated gene expression;Physiological factors;RNA Polymerase II Transcription;Generic Transcription Pathway;Gene expression (Transcription);Muscle contraction
HUMAN|HGNC=23669|UniProtKB=Q6ZRZ4	C9orf47	Uncharacterized protein C9orf47				extracellular region(GO:0005576)		
HUMAN|HGNC=10956|UniProtKB=P46721	SLCO1A2	Solute carrier organic anion transporter family member 1A2	transporter(PC00227)	organic anion transmembrane transporter activity(GO:0008514);bile acid transmembrane transporter activity(GO:0015125);sodium-independent organic anion transmembrane transporter activity(GO:0015347);transmembrane transporter activity(GO:0022857)	ion transport(GO:0006811);organic anion transport(GO:0015711);bile acid and bile salt transport(GO:0015721);sodium-independent organic anion transport(GO:0043252);transmembrane transport(GO:0055085)	plasma membrane(GO:0005886);integral component of plasma membrane(GO:0005887);membrane(GO:0016020);integral component of membrane(GO:0016021)		Metabolism;Recycling of bile acids and salts;Transport of organic anions;Transport of vitamins, nucleosides, and related molecules;Transport of small molecules;Bile acid and bile salt metabolism;Metabolism of lipids;Metabolism of steroids;SLC-mediated transmembrane transport

Overlap of associations with treatment

Gene ID	Mapped ID	Gene name/symbol	Protein class	GO database MF complete	GO database BP complete	GO database CC complete	Pathway	Reactome pathway
HUMAN|HGNC=28246|UniProtKB=Q96ID5	IGSF21	Immunoglobulin superfamily member 21			homophilic cell adhesion via plasma membrane adhesion molecules(GO:0007156);heterophilic cell-cell adhesion via plasma membrane cell adhesion molecules(GO:0007157);synapse maturation(GO:0060074)	adherens junction(GO:0005912);anchored component of external side of plasma membrane(GO:0031362);presynaptic membrane(GO:0042734);postsynaptic membrane(GO:0045211);inhibitory synapse(GO:0060077)		
HUMAN|HGNC=16891|UniProtKB=Q96RU8	TRIB1	Tribbles homolog 1	non-receptor serine/threonine protein kinase(PC00167)	protein kinase activity(GO:0004672);protein kinase inhibitor activity(GO:0004860);protein binding(GO:0005515);ATP binding(GO:0005524);transcription factor binding(GO:0008134);mitogen-activated protein kinase kinase binding(GO:0031434);ubiquitin protein ligase binding(GO:0031625);ubiquitin-protein transferase regulator activity(GO:0055106)	protein phosphorylation(GO:0006468);negative regulation of protein kinase activity(GO:0006469);JNK cascade(GO:0007254);negative regulation of smooth muscle cell migration(GO:0014912);negative regulation of lipopolysaccharide-mediated signaling pathway(GO:0031665);positive regulation of proteasomal ubiquitin-dependent protein catabolic process(GO:0032436);response to lipopolysaccharide(GO:0032496);regulation of MAP kinase activity(GO:0043405);negative regulation of DNA-binding transcription factor activity(GO:0043433);positive regulation of eosinophil differentiation(GO:0045645);positive regulation of macrophage differentiation(GO:0045651);negative regulation of neutrophil differentiation(GO:0045659);negative regulation of smooth muscle cell proliferation(GO:0048662)	nucleus(GO:0005634);cytoplasm(GO:0005737);cytosol(GO:0005829)		
HUMAN|HGNC=23725|UniProtKB=Q5T5U3	ARHGAP10	Rho GTPase-activating protein 21;ARHGAP21		GTPase activator activity(GO:0005096);protein binding(GO:0005515)	Golgi organization(GO:0007030);signal transduction(GO:0007165);positive regulation of GTPase activity(GO:0043547);regulation of small GTPase mediated signal transduction(GO:0051056);Golgi localization(GO:0051645);establishment of Golgi localization(GO:0051683);maintenance of Golgi location(GO:0051684);organelle transport along microtubule(GO:0072384)	Golgi membrane(GO:0000139);Golgi apparatus(GO:0005794);cytosol(GO:0005829);plasma membrane(GO:0005886);actin cytoskeleton(GO:0015629);cell junction(GO:0030054);cytoplasmic vesicle membrane(GO:0030659)		Signal Transduction;Signaling by Rho GTPases;Rho GTPase cycle
HUMAN|HGNC=144|UniProtKB=P63261	ACTG1	Actin, cytoplasmic 2	actin and actin related protein(PC00039)	structural constituent of cytoskeleton(GO:0005200);protein binding(GO:0005515);profilin binding(GO:0005522);ATP binding(GO:0005524);ubiquitin protein ligase binding(GO:0031625);identical protein binding(GO:0042802);structural constituent of postsynaptic actin cytoskeleton(GO:0098973)	angiogenesis(GO:0001525);morphogenesis of a polarized epithelium(GO:0001738);retina homeostasis(GO:0001895);positive regulation of gene expression(GO:0010628);positive regulation of cell migration(GO:0030335);cell junction assembly(GO:0034329);maintenance of blood-brain barrier(GO:0035633);Fc-gamma receptor signaling pathway involved in phagocytosis(GO:0038096);sarcomere organization(GO:0045214);ephrin receptor signaling pathway(GO:0048013);regulation of stress fiber assembly(GO:0051492);regulation of focal adhesion assembly(GO:0051893);membrane organization(GO:0061024);platelet aggregation(GO:0070527);cellular response to interferon-gamma(GO:0071346);positive regulation of wound healing(GO:0090303);postsynaptic actin cytoskeleton organization(GO:0098974);tight junction assembly(GO:0120192);regulation of transepithelial transport(GO:0150111);protein localization to bicellular tight junction(GO:1902396)	extracellular space(GO:0005615);nucleus(GO:0005634);cytosol(GO:0005829);cytoskeleton(GO:0005856);actin filament(GO:0005884);plasma membrane(GO:0005886);cell-cell junction(GO:0005911);focal adhesion(GO:0005925);membrane(GO:0016020);myofibril(GO:0030016);filamentous actin(GO:0031941);apical junction complex(GO:0043296);phagocytic vesicle(GO:0045335);extracellular exosome(GO:0070062);blood microparticle(GO:0072562);dense body(GO:0097433);basal body patch(GO:0120220)	Cadherin signaling pathway->Filamentous F-actin;Alzheimer disease-presenilin pathway->actin;Inflammation mediated by chemokine and cytokine signaling pathway->F-actin;Wnt signaling pathway->NFAT Target Genes;Nicotinic acetylcholine receptor signaling pathway->Actin;Huntington disease->Actin;Integrin signalling pathway->Actin;Cytoskeletal regulation by Rho GTPase->Actin;	EPH-ephrin mediated repulsion of cells;Adherens junctions interactions;L1CAM interactions;MAP2K and MAPK activation;EPHB-mediated forward signaling;RHO GTPase Effectors;RHO GTPases Activate Formins;Immune System;Disease;Gap junction trafficking and regulation;Gap junction trafficking;Developmental Biology;Cell-cell junction organization;Cell-extracellular matrix interactions;Regulation of actin dynamics for phagocytic cup formation;Fcgamma receptor (FCGR) dependent phagocytosis;Signal Transduction;Signaling by VEGF;RHO GTPases Activate WASPs and WAVEs;Innate Immune System;Axon guidance;Oncogenic MAPK signaling;Paradoxical activation of RAF signaling by kinase inactive BRAF;Cytokine Signaling in Immune system;Signaling by BRAF and RAF fusions;Signaling downstream of RAS mutants;Membrane Trafficking;EPH-Ephrin signaling;Gap junction degradation;Recycling pathway of L1;Formation of annular gap junctions;Signaling by Receptor Tyrosine Kinases;RAF/MAP kinase cascade;FLT3 Signaling;MAPK family signaling cascades;Diseases of signal transduction;Cell junction organization;VEGFA-VEGFR2 Pathway;Signaling by RAS mutants;Translocation of SLC2A4 (GLUT4) to the plasma

Overlap of associations with treatment

Gene ID	Mapped ID	Gene name/symbol	Protein class	GO database MF complete	GO database BP complete	GO database CC complete	Pathway	Reactome pathway
								membrane;Signaling by high-kinase activity BRAF mutants;MAPK1/MAPK3 signaling;RHO GTPases activate IQGAPs;Signaling by moderate kinase activity BRAF mutants;Cell-Cell communication;Signaling by Rho GTPases;Clathrin-mediated endocytosis;Vesicle-mediated transport;Interaction between L1 and Ankyrins
HUMAN|HGNC=26149|UniProtKB=Q53LP3	SOWAHC	Ankyrin repeat domain-containing protein SOWAHC						
HUMAN|HGNC=28547|UniProtKB=Q8N0U7	C1orf87	Uncharacterized protein C1orf87						
HUMAN|HGNC=24415|UniProtKB=Q53S33	BOLA3	BolA-like protein 3		molecular_function(GO:0003674);protein binding(GO:0005515)	biological_process(GO:0008150)	mitochondrion(GO:0005739);cytosol(GO:0005829);nuclear body(GO:0016604)		
HUMAN|HGNC=4575|UniProtKB=Q9ULK0	GRID1	Glutamate receptor ionotropic, delta-1		ionotropic glutamate receptor activity(GO:0004970);glutamate receptor activity(GO:0008066);ligand-gated ion channel activity(GO:0015276);signaling receptor activity(GO:0038023);transmitter-gated ion channel activity involved in regulation of postsynaptic membrane potential(GO:1904315)	ion transmembrane transport(GO:0034220);social behavior(GO:0035176);ionotropic glutamate receptor signaling pathway(GO:0035235);synaptic transmission, glutamatergic(GO:0035249);modulation of chemical synaptic transmission(GO:0050804);regulation of postsynaptic membrane potential(GO:0060078)	plasma membrane(GO:0005886);integral component of membrane(GO:0016021);postsynaptic membrane(GO:0045211);extracellular exosome(GO:0070062);glutamatergic synapse(GO:0098978);integral component of postsynaptic density membrane(GO:0099061)		
HUMAN|HGNC=9957|UniProtKB=P78509	RELN	Reelin	extracellular matrix glycoprotein(PC00100)	serine-type peptidase activity(GO:0008236);metal ion binding(GO:0046872);lipoprotein particle receptor binding(GO:0070325);very-low-density lipoprotein particle receptor binding(GO:0070326)	cell morphogenesis involved in differentiation(GO:0000904);neuron migration(GO:0001764);proteolysis(GO:0006508);cell adhesion(GO:0007155);axon guidance(GO:0007411);central nervous system development(GO:0007417);brain development(GO:0007420);long-term memory(GO:0007616);locomotory behavior(GO:0007626);associative learning(GO:0008306);glial cell differentiation(GO:0010001);positive regulation of neuron projection development(GO:0010976);positive regulation of phosphatidylinositol 3-kinase signaling(GO:0014068);dendrite development(GO:0016358);peptidyl-tyrosine phosphorylation(GO:0018108);spinal cord patterning(GO:0021511);ventral spinal cord development(GO:0021517);hippocampus development(GO:0021766);cerebral cortex tangential migration(GO:0021800);layer formation in cerebral cortex(GO:0021819);positive regulation of TOR signaling(GO:0032008);positive regulation of CREB transcription factor activity(GO:0032793);reelin-mediated signaling pathway(GO:0038026);positive regulation of protein kinase activity(GO:0045860);response to pain(GO:0048265);positive regulation of peptidyl-tyrosine phosphorylation(GO:0050731);regulation of behavior(GO:0050795);modulation of chemical synaptic transmission(GO:0050804);positive regulation of small GTPase mediated signal transduction(GO:0051057);positive regulation of synaptic transmission, glutamatergic(GO:0051968);long-term synaptic potentiation(GO:0060291);positive regulation of dendritic spine morphogenesis(GO:0061003);positive regulation of protein tyrosine kinase activity(GO:0061098);positive regulation of synapse maturation(GO:0090129);NMDA glutamate receptor clustering(GO:0097114);postsynaptic density protein 95 clustering(GO:0097119);receptor localization to synapse(GO:0097120);lateral motor column neuron migration(GO:0097477);positive regulation of long-term synaptic potentiation(GO:1900273);positive regulation of lateral motor column neuron migration(GO:1902078);regulation of NMDA receptor activity(GO:2000310);positive regulation of excitatory postsynaptic potential(GO:2000463);positive regulation of AMPA receptor activity(GO:2000969)	extracellular region(GO:0005576);extracellular space(GO:0005615);cytoplasm(GO:0005737);plasma membrane(GO:0005886);dendrite(GO:0030425);extracellular matrix(GO:0031012);neuron projection(GO:0043005)		Developmental Biology;Reelin signalling pathway;Axon guidance
HUMAN|HGNC=3236|UniProtKB=P00533	EGFR	Epidermal growth factor receptor		virus receptor activity(GO:0001618);chromatin binding(GO:0003682);double-stranded DNA binding(GO:0003690);MAP kinase kinase kinase activity(GO:0004709);protein tyrosine kinase activity(GO:0004713);transmembrane receptor protein tyrosine kinase activity(GO:0004714);transmembrane signaling receptor activity(GO:0004888);epidermal growth factor-activated receptor activity(GO:0005006);integrin binding(GO:0005178);protein binding(GO:0005515);calmodulin binding(GO:0005516);ATP binding(GO:0005524);enzyme binding(GO:0019899);kinase binding(GO:0019900);protein kinase binding(GO:0019901);protein phosphatase binding(GO:0019903);nitric-oxide synthase regulator activity(GO:0030235);ubiquitin protein ligase binding(GO:0031625);identical	MAPK cascade(GO:0000165);activation of MAPKK activity(GO:0000186);ossification(GO:0001503);embryonic placenta development(GO:0001892);positive regulation of protein phosphorylation(GO:0001934);hair follicle development(GO:0001942);regulation of transcription by RNA polymerase II(GO:0006357);translation(GO:0006412);receptor-mediated endocytosis(GO:0006898);response to osmotic stress(GO:0006970);signal transduction(GO:0007165);cell surface receptor signaling pathway(GO:0007166);transmembrane receptor protein tyrosine kinase signaling pathway(GO:0007169);epidermal growth factor receptor signaling pathway(GO:0007173);activation of phospholipase C activity(GO:0007202);multicellular organism	Golgi membrane(GO:0000139);extracellular space(GO:0005615);nucleus(GO:0005634);cytoplasm(GO:0005737);endosome(GO:0005768);endoplasmic reticulum membrane(GO:0005789);plasma membrane(GO:0005886);integral component of plasma membrane(GO:0005887);focal adhesion(GO:0005925);basal plasma membrane(GO:0009925);cell surface(GO:0009986);endosome membrane(GO:0010008);membrane(GO:0016020);integral component of membrane(GO:0016021);basolateral plasma membrane(GO:0016323);apical plasma membrane(GO:0016324);cell junction(GO:0030054);endocytic vesicle(GO:0030139);clathrin-coated vesicle membrane(GO:0030665);clathrin-coated endocytic vesicle membrane(GO:0030669);early endosome membrane(GO:0031901);nuclear membrane(GO:0031965);protein-containing	EGF receptor signaling pathway->epidermal growth factor receptor;Gonadotropin-releasing hormone receptor pathway->EGFR;Cadherin signaling pathway->Epidermal growth factor receptor;	Generic Transcription Pathway;Signaling by PTK6;L1CAM interactions;Constitutive Signaling by Ligand-Responsive EGFR Cancer Variants;Signaling by Non-Receptor Tyrosine Kinases;Intracellular signaling by second messengers;GAB1 signalosome;HCMV

Overlap of associations with treatment

Gene ID	Mapped ID	Gene name/symbol	Protein class	GO database MF complete	GO database BP complete	GO database CC complete	Pathway	Reactome pathway
				protein binding(GO:0042802);cadherin binding(GO:0045296);epidermal growth factor binding(GO:0048408);actin filament binding(GO:0051015);ATPase binding(GO:0051117)	development(GO:0007275);salivary gland morphogenesis(GO:0007435);midgut development(GO:0007494);learning or memory(GO:0007611);circadian rhythm(GO:0007623);positive regulation of cell population proliferation(GO:0008284);positive regulation of nitric oxide mediated signal transduction(GO:0010750);magnesium ion homeostasis(GO:0010960);regulation of phosphatidylinositol 3-kinase signaling(GO:0014066);diterpenoid metabolic process(GO:0016101);peptidyl-tyrosine phosphorylation(GO:0018108);viral life cycle(GO:0019058);cerebral cortex cell migration(GO:0021795);cell differentiation(GO:0030154);positive regulation of cell growth(GO:0030307);lung development(GO:0030324);positive regulation of cell migration(GO:0030335);positive regulation of superoxide anion generation(GO:0032930);positive regulation of peptidyl-serine phosphorylation(GO:0033138);response to cobalamin(GO:0033590);response to hydroxyisoflavone(GO:0033594);positive regulation of kinase activity(GO:0033674);cellular response to reactive oxygen species(GO:0034614);cellular response to drug(GO:0035690);peptidyl-tyrosine autophosphorylation(GO:0038083);ERBB2 signaling pathway(GO:0038128);negative regulation of epidermal growth factor receptor signaling pathway(GO:0042059);wound healing(GO:0042060);negative regulation of protein catabolic process(GO:0042177);positive regulation of phosphorylation(GO:0042327);ovulation cycle(GO:0042698);hydrogen peroxide metabolic process(GO:0042743);activation of phospholipase A2 activity by calcium-mediated signaling(GO:0043006);negative regulation of apoptotic process(GO:0043066);positive regulation of MAP kinase activity(GO:0043406);tongue development(GO:0043586);positive regulation of cyclin-dependent protein serine/threonine kinase activity(GO:0045737);positive regulation of DNA repair(GO:0045739);positive regulation of DNA replication(GO:0045740);negative regulation of Notch signaling pathway(GO:0045746);positive regulation of bone resorption(GO:0045780);positive regulation of transcription, DNA-templated(GO:0045893);positive regulation of vasoconstriction(GO:0045907);negative regulation of mitotic cell cycle(GO:0045930);positive regulation of transcription by RNA polymerase II(GO:0045944);regulation of JNK cascade(GO:0046328);viral entry into host cell(GO:0046718);protein autophosphorylation(GO:0046777);astrocyte activation(GO:0048143);positive regulation of fibroblast proliferation(GO:0048146);digestive tract morphogenesis(GO:0048546);positive regulation of smooth muscle cell proliferation(GO:0048661);neuron projection morphogenesis(GO:0048812);positive regulation of epithelial cell proliferation(GO:0050679);positive regulation of inflammatory response(GO:0050729);regulation of peptidyl-tyrosine phosphorylation(GO:0050730);regulation of nitric-oxide synthase activity(GO:0050999);protein insertion into membrane(GO:0051205);response to calcium ion(GO:0051592);positive regulation of protein kinase B signaling(GO:0051897);positive regulation of synaptic transmission, glutamatergic(GO:0051968);morphogenesis of an epithelial fold(GO:0060571);membrane organization(GO:0061024);eyelid development in camera-type eye(GO:0061029);response to UV-A(GO:0070141);regulation of ERK1 and ERK2 cascade(GO:0070372);positive regulation of ERK1 and ERK2 cascade(GO:0070374);cellular response to amino acid stimulus(GO:0071230);cellular response to mechanical stimulus(GO:0071260);cellular response to cadmium ion(GO:0071276);cellular response to epidermal growth factor stimulus(GO:0071364);cellular response to estradiol stimulus(GO:0071392);cellular response to dexamethasone stimulus(GO:0071549);positive regulation of canonical Wnt signaling pathway(GO:0090263);liver regeneration(GO:0097421);positive regulation of blood vessel diameter(GO:0097755);cell-cell adhesion(GO:0098609);positive regulation of protein kinase C activity(GO:1900020);positive regulation of G1/S transition of mitotic cell cycle(GO:1900087);negative regulation of ERBB signaling pathway(GO:1901185);positive regulation of NIK/NF-kappaB signaling(GO:1901224);positive regulation of prolactin secretion(GO:1902722);positive regulation of protein localization to plasma membrane(GO:1903078);positive regulation of production of miRNAs involved in gene silencing by miRNA(GO:1903800);negative regulation of cardiocyte differentiation(GO:1905208);regulation of cell motility(GO:2000145)	complex(GO:0032991);receptor complex(GO:0043235);membrane raft(GO:0045121);synapse(GO:0045202);perinuclear region of cytoplasm(GO:0048471);Shc-EGFR complex(GO:0070435);multivesicular body, internal vesicle lumen(GO:0097489)		Infection;ERBB2 Regulates Cell Motility;PIP3 activates AKT signaling;Gastrin-CREB signalling pathway via PKC and MAPK;Immune System;Gene expression (Transcription);Signaling by Ligand-Responsive EGFR Variants in Cancer;Disease;RNA Polymerase II Transcription;Constitutive Signaling by Aberrant PI3K in Cancer;GPCR downstream signalling;Signaling by EGFRvIII in Cancer;ERBB2 Activates PTK6 Signaling;Constitutive Signaling by EGFRvIII;Downregulation of ERBB2 signaling;EGFR interacts with phospholipase C-gamma;Transcriptional regulation by the AP-2 (TFAP2) family of transcription factors;ESR-mediated signaling;Signaling by NOTCH3;PI5P, PP2A and IER3 Regulate PI3K/AKT Signaling;EGFR Transactivation by Gastrin;Developmental Biology;PTK6 promotes HIF1A stabilization;PLCG1 events in ERBB2 signaling;G alpha (q) signalling events;Signaling by EGFR in Cancer;Signal Transduction;Signaling by NOTCH;PI3K/AKT Signaling in Cancer;Axon guidance;Signal transduction by L1;Estrogen-dependent nuclear events downstream of ESR-membrane signaling;HCMV Early Events;Inhibition of Signaling by Overexpressed EGFR;Signaling by Overexpressed Wild-Type EGFR in Cancer;PI3K events in ERBB2 signaling;GRB2 events in ERBB2 signaling;Cytokine Signaling in Immune system;Signaling by EGFR;Signaling by GPCR;NOTCH3 Activation and Transmission of Signal to the Nucleus;Membrane Trafficking;Signaling by ERBB4;Signaling by ERBB2 in Cancer;EGFR downregulation;Extra-nuclear estrogen signaling;Signaling by Receptor Tyrosine Kinases;RAF/MAP kinase cascade;Infectious disease;FLT3 Signaling;Signaling by Nuclear Receptors;MAPK family signaling cascades;Diseases of signal

Gene ID	Mapped ID	Gene name/symbol	Protein class	GO database MF complete	GO database BP complete	GO database CC complete	Pathway	Reactome pathway
								transduction;Negative regulation of the PI3K/AKT network;Signaling by ERBB2 KD Mutants;MAPK1/MAPK3 signaling;SHC1 events in EGFR signaling;SHC1 events in ERBB2 signaling;Clathrin-mediated endocytosis;Vesicle-mediated transport;TFAP2 (AP-2) family regulates transcription of growth factors and their receptors;Cargo recognition for clathrin-mediated endocytosis;GRB2 events in EGFR signaling;Signaling by ERBB2
HUMAN|HGNC=29672|UniProtKB=Q8IY33	MICALL2	MICAL-like protein 2		protein binding(GO:0005515);Rab GTPase binding(GO:0017137);filamin binding(GO:0031005);metal ion binding(GO:0046872);actin filament binding(GO:0051015)	actin filament polymerization(GO:0030041);neuron projection development(GO:0031175);actin cytoskeleton reorganization(GO:0031532);endocytic recycling(GO:0032456);substrate adhesion-dependent cell spreading(GO:0034446);bicellular tight junction assembly(GO:0070830);positive regulation of protein targeting to mitochondrion(GO:1903955)	stress fiber(GO:0001725);cytosol(GO:0005829);plasma membrane(GO:0005886);cell-cell junction(GO:0005911);bicellular tight junction(GO:0005923);actin filament bundle(GO:0032432);neuron projection(GO:0043005);recycling endosome(GO:0055037)		
HUMAN|HGNC=400|UniProtKB=Q13740	ALCAM	CD166 antigen	immunoglobulin superfamily cell adhesion molecule(PC00125)	signaling receptor binding(GO:0005102);protein binding(GO:0005515);identical protein binding(GO:0042802)	adaptive immune response(GO:0002250);cell adhesion(GO:0007155);heterophilic cell-cell adhesion via plasma membrane cell adhesion molecules(GO:0007157);signal transduction(GO:0007165);motor neuron axon guidance(GO:0008045);retinal ganglion cell axon guidance(GO:0031290);axon extension involved in axon guidance(GO:0048846);neuron projection extension(GO:1990138)	immunological synapse(GO:0001772);plasma membrane(GO:0005886);integral component of plasma membrane(GO:0005887);focal adhesion(GO:0005925);external side of plasma membrane(GO:0009897);integral component of membrane(GO:0016021);axon(GO:0030424);dendrite(GO:0030425);intrinsic component of plasma membrane(GO:0031226);T cell receptor complex(GO:0042101);neuronal cell body(GO:0043025);extracellular exosome(GO:0070062)		Developmental Biology;L1CAM interactions;Axon guidance
HUMAN|HGNC=6844|UniProtKB=P45985	MAP2K4	Dual specificity mitogen-activated protein kinase kinase 4		protein kinase activity(GO:0004672);protein serine/threonine kinase activity(GO:0004674);MAP kinase kinase activity(GO:0004708);protein tyrosine kinase activity(GO:0004713);protein binding(GO:0005515);ATP binding(GO:0005524);JUN kinase kinase activity(GO:0008545);mitogen-activated protein kinase kinase kinase binding(GO:0031435)	activation of MAPK activity(GO:0000187);apoptotic process(GO:0006915);signal transduction(GO:0007165);JNK cascade(GO:0007254);activation of JUN kinase activity(GO:0007257);response to wounding(GO:0009611);peptidyl-tyrosine phosphorylation(GO:0018108);positive regulation of smooth muscle cell apoptotic process(GO:0034393);Fc-epsilon receptor signaling pathway(GO:0038095);positive regulation of neuron apoptotic process(GO:0043525);positive regulation of DNA replication(GO:0045740);positive regulation of nitric-oxide synthase biosynthetic process(GO:0051770);cell growth involved in cardiac muscle cell development(GO:0061049);cellular response to mechanical stimulus(GO:0071260);cellular response to sorbitol(GO:0072709);negative regulation of motor neuron apoptotic process(GO:2000672)	nucleus(GO:0005634);cytosol(GO:0005829);axon(GO:0030424);dendrite cytoplasm(GO:0032839);perikaryon(GO:0043204)	Apoptosis signaling pathway->SAPK/ERK kinasae 1;Huntington disease->Mitogen activated protein kinase kinase 4;EGF receptor signaling pathway->Dual specificity mitogen-activated protein kinase kinase 4 and 7;Angiogenesis->c-Jun N-terminal Kinase Kinase 1;CCKR signaling map->MAP2K4;Ras Pathway->Mitogen activated protein kinase kinase 4/7;FAS signaling pathway->Mitogen-activated protein kinase kinase 4;FGF signaling pathway->Dual specificity mitogen-activated protein kinase kinase 4 and 7;p38 MAPK pathway->mitogen-activated protein kinase kinase 4;Integrin signalling pathway->Mitogen-activated protein kinase kinase;Oxidative stress response->Mitogen-activated protein kinase kinase 4;Huntington disease->Stress signalling kinase 1;	Interleukin-1 family signaling;Cellular Senescence;Interleukin-17 signaling;Oxidative Stress Induced Senescence;Cellular responses to stress;Signaling by Interleukins;MyD88-independent TLR4 cascade ;FCERI mediated MAPK activation;Toll-like Receptor Cascades;Toll Like Receptor 2 (TLR2) Cascade;Immune System;Disease;Uptake and actions of bacterial toxins;Uptake and function of anthrax toxins;Toll Like Receptor TLR6:TLR2 Cascade;Toll Like Receptor 7/8 (TLR7/8) Cascade;MAP kinase activation;Fc epsilon receptor (FCERI) signaling;Innate Immune System;MyD88 cascade initiated on plasma membrane;TRAF6 mediated induction of NFkB and MAP kinases upon TLR7/8 or 9 activation;MyD88:MAL(TIRAP) cascade initiated on plasma membrane;Cytokine Signaling in Immune system;Toll Like Receptor TLR1:TLR2 Cascade;JNK (c-Jun kinases) phosphorylation and activation mediated by activated human TAK1;Toll Like Receptor 5 (TLR5) Cascade;Toll Like Receptor 10 (TLR10) Cascade;TRIF(TICAM1)-mediated TLR4 signaling ;MAP3K8 (TPL2)-dependent MAPK1/3

Gene ID	Mapped ID	Gene name/symbol	Protein class	GO database MF complete	GO database BP complete	GO database CC complete	Pathway	Reactome pathway
								activation;Infectious disease;Toll Like Receptor 4 (TLR4) Cascade;Toll Like Receptor 9 (TLR9) Cascade;MyD88 dependent cascade initiated on endosome;Cellular responses to external stimuli;Toll Like Receptor 3 (TLR3) Cascade;Interleukin-1 signaling
HUMAN|HGNC=17098|UniProtKB=Q9UPY3	DICER1	Endoribonuclease Dicer	endodeoxyribonuclease(PC00093);endoribonuclease(PC00094)	DNA binding(GO:0003677);RNA binding(GO:0003723);double-stranded RNA binding(GO:0003725);helicase activity(GO:0004386);endoribonuclease activity(GO:0004521);ribonuclease III activity(GO:0004525);deoxyribonuclease I activity(GO:0004530);protein binding(GO:0005515);ATP binding(GO:0005524);protein domain specific binding(GO:0019904);siRNA binding(GO:0035197);metal ion binding(GO:0046872);pre-miRNA binding(GO:0070883)	negative regulation of transcription by RNA polymerase II(GO:0000122);apoptotic DNA fragmentation(GO:0006309);RNA processing(GO:0006396);miRNA metabolic process(GO:0010586);negative regulation of Schwann cell proliferation(GO:0010626);negative regulation of gene expression(GO:0010629);positive regulation of Schwann cell differentiation(GO:0014040);nerve development(GO:0021675);production of siRNA involved in RNA interference(GO:0030422);targeting of mRNA for destruction involved in RNA interference(GO:0030423);pre-miRNA processing(GO:0031054);positive regulation of myelination(GO:0031643);peripheral nervous system myelin formation(GO:0032290);negative regulation of tumor necrosis factor production(GO:0032720);conversion of ds siRNA to ss siRNA involved in RNA interference(GO:0033168);siRNA loading onto RISC involved in RNA interference(GO:0035087);tube formation(GO:0035148);production of miRNAs involved in gene silencing by miRNA(GO:0035196);miRNA loading onto RISC involved in gene silencing by miRNA(GO:0035280);conversion of ds siRNA to ss siRNA(GO:0036404);NIK/NF-kappaB signaling(GO:0038061);neuron projection morphogenesis(GO:0048812);RNA phosphodiester bond hydrolysis(GO:0090501);RNA phosphodiester bond hydrolysis, endonucleolytic(GO:0090502)	nucleus(GO:0005634);cytoplasm(GO:0005737);endoplasmic reticulum-Golgi intermediate compartment(GO:0005793);cytosol(GO:0005829);RISC complex(GO:0016442);dendrite(GO:0030425);growth cone(GO:0030426);ARC complex(GO:0033167);perinuclear region of cytoplasm(GO:0048471);extracellular exosome(GO:0070062);RISC-loading complex(GO:0070578)		Gene Silencing by RNA;Gene expression (Transcription);MicroRNA (miRNA) biogenesis;Small interfering RNA (siRNA) biogenesis
HUMAN|HGNC=23842|UniProtKB=Q8N961	ABTB2	Ankyrin repeat and BTB/POZ domain-containing protein 2	scaffold/adaptor protein(PC00226)	molecular_function(GO:0003674);protein heterodimerization activity(GO:0046982)	cellular response to toxic substance(GO:0097237)	nucleoplasm(GO:0005654)		
HUMAN|HGNC=22962|UniProtKB=Q71F56	MED13L	Mediator of RNA polymerase II transcription subunit 13-like		transcription coregulator activity(GO:0003712)	regulation of transcription by RNA polymerase II(GO:0006357)	nucleus(GO:0005634);mediator complex(GO:0016592)		Developmental Biology;Metabolism of lipids;PPARA activates gene expression;Regulation of lipid metabolism by PPARalpha;Transcriptional regulation of white adipocyte differentiation;Metabolism
HUMAN|HGNC=6163|UniProtKB=P26012	ITGB8	Integrin beta-8	cell adhesion molecule(PC00069)	integrin binding(GO:0005178);signaling receptor activity(GO:0038023);extracellular matrix protein binding(GO:1990430)	vasculogenesis(GO:0001570);ganglioside metabolic process(GO:0001573);cell adhesion(GO:0007155);integrin-mediated signaling pathway(GO:0007229);positive regulation of gene expression(GO:0010628);negative regulation of gene expression(GO:0010629);cell migration(GO:0016477);extracellular matrix organization(GO:0030198);cell adhesion mediated by integrin(GO:0033627);positive regulation of angiogenesis(GO:0045766);cartilage development(GO:0051216);placenta blood vessel development(GO:0060674);regulation of transforming growth factor beta activation(GO:1901388)	plasma membrane(GO:0005886);focal adhesion(GO:0005925);integrin complex(GO:0008305);cell surface(GO:0009986);integrin alphav-beta8 complex(GO:0034686);extracellular exosome(GO:0070062)	Integrin signalling pathway->Integrin beta;	Integrin cell surface interactions;Elastic fibre formation;Extracellular matrix organization;Molecules associated with elastic fibres
HUMAN|HGNC=19893|UniProtKB=Q6QNK2	ADGRD1	Adhesion G-protein coupled receptor D1	G-protein coupled receptor(PC00021)	G protein-coupled receptor activity(GO:0004930);protein binding(GO:0005515)	cell surface receptor signaling pathway(GO:0007166);G protein-coupled receptor signaling pathway(GO:0007186);adenylate cyclase-activating G protein-coupled receptor signaling pathway(GO:0007189)	plasma membrane(GO:0005886);integral component of plasma membrane(GO:0005887);integral component of membrane(GO:0016021)		
HUMAN|HGNC=21752|UniProtKB=Q9NRP4	SDHAF3	Succinate dehydrogenase assembly factor 3, mitochondrial			succinate metabolic process(GO:0006105);regulation of gluconeogenesis(GO:0006111);mitochondrial respiratory chain complex II assembly(GO:0034553)	mitochondrion(GO:0005739);mitochondrial intermembrane space(GO:0005758);mitochondrial matrix(GO:0005759)		
HUMAN|HGNC=6700|UniProtKB=Q14114	LRP8	Low-density lipoprotein receptor-related protein 8	apolipoprotein(PC00052)	transmembrane signaling receptor activity(GO:0004888);low-density lipoprotein particle receptor activity(GO:0005041);calcium ion binding(GO:0005509);protein binding(GO:0005515);high-density lipoprotein particle binding(GO:0008035);kinesin binding(GO:0019894);very-low-density lipoprotein particle receptor activity(GO:0030229);apolipoprotein binding(GO:0034185);cargo receptor activity(GO:0038024);reelin receptor activity(GO:0038025)	retinoid metabolic process(GO:0001523);proteolysis(GO:0006508);lipid metabolic process(GO:0006629);endocytosis(GO:0006897);signal transduction(GO:0007165);cytokine-mediated signaling pathway(GO:0019221);ammon gyrus development(GO:0021541);cerebral cortex development(GO:0021987);positive regulation of CREB transcription factor activity(GO:0032793);reelin-mediated signaling pathway(GO:0038026);response to drug(GO:0042493);regulation of apoptotic process(GO:0042981);regulation of innate immune response(GO:0045088);positive regulation of peptidyl-tyrosine phosphorylation(GO:0050731);modulation of chemical synaptic transmission(GO:0050804);positive regulation of dendritic spine morphogenesis(GO:0061003);positive regulation of protein tyrosine kinase activity(GO:0061098);cellular response to growth factor stimulus(GO:0071363);cellular response to	extracellular region(GO:0005576);microtubule associated complex(GO:0005875);plasma membrane(GO:0005886);caveola(GO:0005901);postsynaptic density(GO:0014069);membrane(GO:0016020);integral component of membrane(GO:0016021);axon(GO:0030424);dendrite(GO:0030425);neuronal cell body(GO:0043025);receptor complex(GO:0043235)		Signal Transduction;Metabolism;Metabolism of fat-soluble vitamins;Visual phototransduction;Metabolism of vitamins and cofactors;G alpha (i) signalling events;Retinoid metabolism and transport;Platelet homeostasis;Platelet sensitization by LDL;Signaling by GPCR;Hemostasis;GPCR downstream signalling

Overlap of associations with treatment

Gene ID	Mapped ID	Gene name/symbol	Protein class	GO database MF complete	GO database BP complete	GO database CC complete	Pathway	Reactome pathway
					cholesterol(GO:0071397);positive regulation of dendrite development(GO:1900006)			
HUMAN|HGNC=1400|UniProtKB=Q9NY47	CACNA2D2	Voltage-dependent calcium channel subunit alpha-2/delta-2		voltage-gated ion channel activity(GO:0005244);voltage-gated calcium channel activity(GO:0005245);calcium channel activity(GO:0005262);metal ion binding(GO:0046872)	neuromuscular junction development(GO:0007528);regulation of ion transmembrane transport(GO:0034765);regulation of multicellular organism growth(GO:0040014);positive regulation of organ growth(GO:0046622);muscle fiber development(GO:0048747);regulation of insulin secretion(GO:0050796);rhythmic synaptic transmission(GO:0060024);cardiac conduction(GO:0061337);calcium ion transmembrane transport(GO:0070588)	plasma membrane(GO:0005886);voltage-gated calcium channel complex(GO:0005891);integral component of membrane(GO:0016021)		Metabolism;Neuronal System;Adrenaline,noradrenaline inhibits insulin secretion;Transmission across Chemical Synapses;Muscle contraction;Presynaptic depolarization and calcium channel opening;Phase 2 - plateau phase;Regulation of insulin secretion;Phase 0 - rapid depolarisation;Cardiac conduction;Integration of energy metabolism
HUMAN|HGNC=16960|UniProtKB=P52954	LBX1	Transcription factor LBX1	homeodomain transcription factor(PC00119)	DNA-binding transcription factor activity, RNA polymerase II-specific(GO:0000981);DNA binding(GO:0003677);protein binding(GO:0005515);sequence-specific double-stranded DNA binding(GO:1990837)	heart looping(GO:0001947);regulation of transcription by RNA polymerase II(GO:0006357);muscle organ development(GO:0007517);negative regulation of cell population proliferation(GO:0008285);anatomical structure morphogenesis(GO:0009653);spinal cord motor neuron differentiation(GO:0021522);regulation of transcription from RNA polymerase II promoter involved in spinal cord association neuron specification(GO:0021920);negative regulation of neuron differentiation(GO:0045665);neuron fate determination(GO:0048664)	nuclear chromatin(GO:0000790);nucleus(GO:0005634);transcription regulator complex(GO:0005667)		
HUMAN|HGNC=25247|UniProtKB=Q8IYW2	CFAP46	Cilia- and flagella-associated protein 46			axoneme assembly(GO:0035082);cilium movement involved in cell motility(GO:0060294)	axoneme(GO:0005930)		
HUMAN|HGNC=7212|UniProtKB=Q96Q89	KIF20B	Kinesin-like protein KIF20B	microtubule binding motor protein(PC00156)	microtubule motor activity(GO:0003777);protein binding(GO:0005515);ATP binding(GO:0005524);microtubule binding(GO:0008017);ATP-dependent microtubule motor activity, plus-end-directed(GO:0008574);ATPase activity(GO:0016887);protein homodimerization activity(GO:0042803);WW domain binding(GO:0050699)	neural tube closure(GO:0001843);microtubule-based movement(GO:0007018);cell cycle arrest(GO:0007050);regulation of mitotic nuclear division(GO:0007088);positive regulation of cell population proliferation(GO:0008284);positive regulation of cytokinesis(GO:0032467);protein localization to microtubule(GO:0035372);neuron projection morphogenesis(GO:0048812);cell division(GO:0051301);positive regulation of intracellular protein transport(GO:0090316);positive regulation of mitotic cytokinetic process(GO:1903438);regulation of establishment of cell polarity(GO:2000114);positive regulation of neuron migration(GO:2001224)	nucleus(GO:0005634);nucleoplasm(GO:0005654);nucleolus(GO:0005730);cytoplasm(GO:0005737);centrosome(GO:0005813);cytosol(GO:0005829);kinesin complex(GO:0005871);microtubule(GO:0005874);growth cone(GO:0030426);midbody(GO:0030496);intercellular bridge(GO:0045171);perinuclear region of cytoplasm(GO:0048471);spindle midzone(GO:0051233);contractile ring(GO:0070938);mitotic spindle pole(GO:0097431);mitotic spindle midzone(GO:1990023)		Membrane Trafficking;COPI-dependent Golgi-to-ER retrograde traffic;Vesicle-mediated transport;Golgi-to-ER retrograde transport;Hemostasis;Kinesins;Intra-Golgi and retrograde Golgi-to-ER traffic;Factors involved in megakaryocyte development and platelet production
HUMAN|HGNC=10888|UniProtKB=Q9NPC8	SIX2	Homeobox protein SIX2	homeodomain transcription factor(PC00119)	RNA polymerase II cis-regulatory region sequence-specific DNA binding(GO:0000978);DNA-binding transcription factor activity, RNA polymerase II-specific(GO:0000981);DNA-binding transcription activator activity, RNA polymerase II-specific(GO:0001228);DNA-binding transcription factor activity(GO:0003700);protein binding(GO:0005515);transcription factor binding(GO:0008134);protein-containing complex binding(GO:0044877);sequence-specific double-stranded DNA binding(GO:1990837)	kidney development(GO:0001822);chondrocyte differentiation(GO:0002062);mesenchymal to epithelial transition involved in metanephros morphogenesis(GO:0003337);regulation of transcription by RNA polymerase II(GO:0006357);protein import into nucleus(GO:0006606);mesodermal cell fate specification(GO:0007501);cell population proliferation(GO:0008283);anatomical structure morphogenesis(GO:0009653);anterior/posterior axis specification(GO:0009948);cell migration(GO:0016477);regulation of ossification(GO:0030278);regulation of chondrocyte differentiation(GO:0032330);middle ear morphogenesis(GO:0042474);negative regulation of cell differentiation(GO:0045596);positive regulation of transcription by RNA polymerase II(GO:0045944);embryonic digestive tract morphogenesis(GO:0048557);embryonic cranial skeleton morphogenesis(GO:0048701);nephron development(GO:0072006);nephron morphogenesis(GO:0072028);mesenchymal stem cell maintenance involved in nephron morphogenesis(GO:0072038);condensed mesenchymal cell proliferation(GO:0072137);mesenchymal cell differentiation involved in kidney development(GO:0072161);regulation of branching involved in ureteric bud morphogenesis(GO:0090189);mesenchymal stem cell proliferation(GO:0097168);positive regulation of chondrocyte proliferation(GO:1902732)	nuclear chromatin(GO:0000790);nucleus(GO:0005634);transcription regulator complex(GO:0005667)		
HUMAN|HGNC=29989|UniProtKB=Q86YP4	GATAD2A	Transcriptional repressor p66-alpha	DNA-binding transcription factor(PC00218)	protein binding(GO:0005515);zinc ion binding(GO:0008270);protein-macromolecule adaptor activity(GO:0030674);sequence-specific DNA binding(GO:0043565)	negative regulation of transcription by RNA polymerase II(GO:0000122);DNA methylation(GO:0006306);negative regulation of transcription, DNA-templated(GO:0045892)	nucleus(GO:0005634);nucleoplasm(GO:0005654);NuRD complex(GO:0016581);nuclear speck(GO:0016607)		Regulation of PTEN gene transcription;ERCC6 (CSB) and EHMT2 (G9a) positively regulate rRNA expression;PIP3 activates AKT signaling;Regulation of TP53 Activity;Gene expression (Transcription);Chromatin modifying enzymes;Regulation of TP53 Activity through Acetylation;Transcriptional Regulation by TP53;RNA Polymerase I Transcription

Overlap of associations with treatment

Gene ID	Mapped ID	Gene name/symbol	Protein class	GO database MF complete	GO database BP complete	GO database CC complete	Pathway	Reactome pathway
								Initiation;RNA Polymerase II Transcription;Generic Transcription Pathway;RNA Polymerase I Promoter Clearance;Chromatin organization;PTEN Regulation;Signal Transduction;Epigenetic regulation of gene expression;RNA Polymerase I Transcription;Positive epigenetic regulation of rRNA expression;Intracellular signaling by second messengers;HDACs deacetylate histones
HUMAN|HGNC=11085|UniProtKB=O75093	SLIT1	Slit homolog 1 protein		calcium ion binding(GO:0005509);protein binding(GO:0005515);heparin binding(GO:0008201);Roundabout binding(GO:0048495)	nuclear migration(GO:0007097);nervous system development(GO:0007399);axon guidance(GO:0007411);motor neuron axon guidance(GO:0008045);cell differentiation(GO:0030154);retinal ganglion cell axon guidance(GO:0031290);dorsal/ventral axon guidance(GO:0033563);axon extension involved in axon guidance(GO:0048846);forebrain morphogenesis(GO:0048853);negative chemotaxis(GO:0050919);negative regulation of synapse assembly(GO:0051964)	extracellular region(GO:0005576);extracellular space(GO:0005615)	Axon guidance mediated by Slit/Robo->Slit;	Netrin-1 signaling;Developmental Biology;Regulation of cortical dendrite branching;Regulation of commissural axon pathfinding by SLIT and ROBO;Signaling by ROBO receptors;Regulation of expression of SLITs and ROBOs;Axon guidance
HUMAN|HGNC=9304|UniProtKB=P63151	PPP2R2A	Serine/threonine-protein phosphatase 2A 55 kDa regulatory subunit B alpha isoform	protein phosphatase(PC00195)	protein binding(GO:0005515);protein phosphatase regulator activity(GO:0019888);protein-containing complex binding(GO:0044877);tau protein binding(GO:0048156);protein phosphatase 2A binding(GO:0051721)	G2/M transition of mitotic cell cycle(GO:0000086);nuclear-transcribed mRNA catabolic process, nonsense-mediated decay(GO:0000184);protein dephosphorylation(GO:0006470);response to morphine(GO:0043278);regulation of phosphoprotein phosphatase activity(GO:0043666);peptidyl-serine dephosphorylation(GO:0070262)	protein phosphatase type 2A complex(GO:0000159);nucleoplasm(GO:0005654);cytosol(GO:0005829);glutamatergic synapse(GO:0098978)	FGF signaling pathway->protein phosphatase 2A;	G2/M Transition;Mitotic Metaphase and Anaphase;Mitotic G1 phase and G1/S transition;Cyclin A/B1/B2 associated events during G2/M transition;Nuclear Envelope Reassembly;Nonsense Mediated Decay (NMD) enhanced by the Exon Junction Complex (EJC);M Phase;Mitotic G2-G2/M phases;G1 Phase;Mitotic Anaphase;Initiation of Nuclear Envelope Reformation;Cyclin D associated events in G1;Metabolism of RNA;Cell Cycle;Nonsense-Mediated Decay (NMD);Cell Cycle, Mitotic
HUMAN|HGNC=18273|UniProtKB=Q96PN7	TRERF1	Transcriptional-regulating factor 1		DNA binding(GO:0003677);transcription coactivator activity(GO:0003713);transcription corepressor activity(GO:0003714);protein binding(GO:0005515);transcription factor binding(GO:0008134);nuclear receptor transcription coactivator activity(GO:0030374);progesterone receptor binding(GO:0033142);metal ion binding(GO:0046872)	regulation of transcription by RNA polymerase II(GO:0006357);histone deacetylation(GO:0016575);negative regulation of transcription, DNA-templated(GO:0045892);positive regulation of transcription, DNA-templated(GO:0045893);positive regulation of transcription by RNA polymerase II(GO:0045944);progesterone receptor signaling pathway(GO:0050847);cellular response to progesterone stimulus(GO:0071393)	histone deacetylase complex(GO:0000118);fibrillar center(GO:0001650);nucleus(GO:0005634);nucleoplasm(GO:0005654);transcription regulator complex(GO:0005667);cytosol(GO:0005829)		
HUMAN|HGNC=11786|UniProtKB=P35442	THBS2	Thrombospondin-2		extracellular matrix structural constituent(GO:0005201);calcium ion binding(GO:0005509);protein binding(GO:0005515);heparin binding(GO:0008201)	cell adhesion(GO:0007155);negative regulation of angiogenesis(GO:0016525);positive regulation of synapse assembly(GO:0051965)	extracellular region(GO:0005576);basement membrane(GO:0005604);platelet alpha granule(GO:0031091);collagen-containing extracellular matrix(GO:0062023)		Signal Transduction;Signaling by Receptor Tyrosine Kinases;Post-translational protein modification;Diseases of glycosylation;Defective B3GALTL causes Peters-plus syndrome (PpS);Metabolism of proteins;Diseases associated with O-glycosylation of proteins;Disease;Signaling by PDGF;O-glycosylation of TSR domain-containing proteins;O-linked glycosylation
HUMAN|HGNC=11514|UniProtKB=O43581	SYT7	Synaptotagmin-7	membrane trafficking regulatory protein(PC00151)	SNARE binding(GO:0000149);phosphatidylserine binding(GO:0001786);calcium ion binding(GO:0005509);protein binding(GO:0005515);calmodulin binding(GO:0005516);calcium-dependent phospholipid binding(GO:0005544);phosphatidylinositol-4,5-bisphosphate	plasma membrane repair(GO:0001778);exocytosis(GO:0006887);phagocytosis(GO:0006909);regulation of dopamine secretion(GO:0014059);vesicle-mediated transport(GO:0016192);calcium-ion regulated exocytosis(GO:0017156);regulation of calcium ion-dependent	lysosome(GO:0005764);lysosomal membrane(GO:0005765);peroxisome(GO:0005777);peroxisomal membrane(GO:0005778);cytosol(GO:0005829);plasma membrane(GO:0005886);synaptic vesicle(GO:0008021);integral component of membrane(GO:0016021);dendrite(GO:0030425);phagocytic vesicle	Synaptic vesicle trafficking->Synaptotagmin;	Protein-protein interactions at synapses;Neuronal System;Neurexins and neuroligins

Overlap of associations with treatment

Gene ID	Mapped ID	Gene name/symbol	Protein class	GO database MF complete	GO database BP complete	GO database CC complete	Pathway	Reactome pathway
				binding(GO:0005546);syntaxin binding(GO:0019905);clathrin binding(GO:0030276)	exocytosis(GO:0017158);synaptic vesicle recycling(GO:0036465);positive regulation of calcium ion-dependent exocytosis(GO:0045956);regulation of bone remodeling(GO:0046850);calcium ion-regulated exocytosis of neurotransmitter(GO:0048791);regulation of phagocytosis(GO:0050764);regulation of insulin secretion(GO:0050796);regulation of glucagon secretion(GO:0070092);cellular response to calcium ion(GO:0071277);vesicle-mediated cholesterol transport(GO:0090119);phagosome-lysosome fusion(GO:0090385);calcium-dependent activation of synaptic vesicle fusion(GO:0099502);short-term synaptic potentiation(GO:1990926);calcium ion regulated lysosome exocytosis(GO:1990927)	membrane(GO:0030670);synaptic vesicle membrane(GO:0030672);dense core granule(GO:0031045);early phagosome(GO:0032009);presynaptic membrane(GO:0042734);neuronal cell body(GO:0043025);axon terminus(GO:0043679);extracellular exosome(GO:0070062);exocytic vesicle(GO:0070382);glutamatergic synapse(GO:0098978);GABA-ergic synapse(GO:0098982)		
HUMAN|HGNC=4557|UniProtKB=O75715	GPX5	Epididymal secretory glutathione peroxidase	peroxidase(PC00180)	peroxidase activity(GO:0004601);glutathione peroxidase activity(GO:0004602)	lipid metabolic process(GO:0006629);cellular response to oxidative stress(GO:0034599);oxidation-reduction process(GO:0055114);cellular oxidant detoxification(GO:0098869)	extracellular region(GO:0005576)		Detoxification of Reactive Oxygen Species;Cellular responses to stress;Cellular responses to external stimuli
HUMAN|HGNC=3519|UniProtKB=Q99502	EYA1	Eyes absent homolog 1		RNA binding(GO:0003723);protein tyrosine phosphatase activity(GO:0004725);protein binding(GO:0005515);metal ion binding(GO:0046872);protein serine phosphatase activity(GO:0106306);protein threonine phosphatase activity(GO:0106307)	metanephros development(GO:0001656);branching involved in ureteric bud morphogenesis(GO:0001658);outflow tract morphogenesis(GO:0003151);DNA repair(GO:0006281);double-strand break repair(GO:0006302);pattern specification process(GO:0007389);mesodermal cell fate specification(GO:0007501);sensory perception of sound(GO:0007605);anatomical structure morphogenesis(GO:0009653);response to ionizing radiation(GO:0010212);striated muscle tissue development(GO:0014706);histone dephosphorylation(GO:0016576);protein sumoylation(GO:0016925);cell differentiation(GO:0030154);peptidyl-tyrosine dephosphorylation(GO:0035335);aorta morphogenesis(GO:0035909);ear morphogenesis(GO:0042471);outer ear morphogenesis(GO:0042473);middle ear morphogenesis(GO:0042474);regulation of neuron differentiation(GO:0045664);positive regulation of DNA repair(GO:0045739);positive regulation of transcription by RNA polymerase II(GO:0045944);neuron fate specification(GO:0048665);embryonic skeletal system morphogenesis(GO:0048704);semicircular canal morphogenesis(GO:0048752);anatomical structure development(GO:0048856);positive regulation of epithelial cell proliferation(GO:0050679);pharyngeal system development(GO:0060037);otic vesicle morphogenesis(GO:0071600);positive regulation of secondary heart field cardioblast proliferation(GO:0072513);cochlea morphogenesis(GO:0090103);negative regulation of extrinsic apoptotic signaling pathway in absence of ligand(GO:2001240)	nucleus(GO:0005634);nucleoplasm(GO:0005654);cytoplasm(GO:0005737);nuclear body(GO:0016604);protein-DNA complex(GO:0032993)		Recruitment and ATM-mediated phosphorylation of repair and signaling proteins at DNA double strand breaks;DNA Double Strand Break Response;DNA Repair;DNA Double-Strand Break Repair
HUMAN|HGNC=13056|UniProtKB=O14978	ZNF263	Zinc finger protein 263	C2H2 zinc finger transcription factor(PC00248)	RNA polymerase II cis-regulatory region sequence-specific DNA binding(GO:0000978);DNA-binding transcription factor activity, RNA polymerase II-specific(GO:0000981);DNA-binding transcription repressor activity, RNA polymerase II-specific(GO:0001227);DNA-binding transcription factor activity(GO:0003700);protein binding(GO:0005515);sequence-specific DNA binding(GO:0043565);metal ion binding(GO:0046872);sequence-specific double-stranded DNA binding(GO:1990837)	negative regulation of transcription by RNA polymerase II(GO:0000122);regulation of transcription, DNA-templated(GO:0006355);regulation of transcription by RNA polymerase II(GO:0006357)	nuclear chromatin(GO:0000790);nucleus(GO:0005634)		RNA Polymerase II Transcription;Generic Transcription Pathway;Gene expression (Transcription)
HUMAN|HGNC=8702|UniProtKB=Q9Y5G9	PCDHGA4	Protocadherin gamma-A4		calcium ion binding(GO:0005509);protein binding(GO:0005515)	cell adhesion(GO:0007155);homophilic cell adhesion via plasma membrane adhesion molecules(GO:0007156);spermatogenesis(GO:0007283)	integral component of plasma membrane(GO:0005887)		
HUMAN|HGNC=13800|UniProtKB=Q9GZZ6	CHRNA10	Neuronal acetylcholine receptor subunit alpha-10	ligand-gated ion channel(PC00141)	transmembrane signaling receptor activity(GO:0004888);signaling receptor binding(GO:0005102);extracellular ligand-gated ion channel activity(GO:0005230);calcium channel activity(GO:0005262);acetylcholine-gated cation-selective channel activity(GO:0022848);neurotransmitter receptor activity(GO:0030594)	signal transduction(GO:0007165);positive regulation of cytosolic calcium ion concentration(GO:0007204);chemical synaptic transmission(GO:0007268);synaptic transmission, cholinergic(GO:0007271);ion transmembrane transport(GO:0034220);regulation of cell population proliferation(GO:0042127);regulation of membrane potential(GO:0042391);inner ear morphogenesis(GO:0042472);nervous system process(GO:0050877);detection of mechanical stimulus involved in sensory perception of sound(GO:0050910);excitatory postsynaptic potential(GO:0060079);negative regulation of ERK1 and ERK2 cascade(GO:0070373);calcium ion transmembrane transport(GO:0070588)	integral component of plasma membrane(GO:0005887);membrane(GO:0016020);integral component of membrane(GO:0016021);axon(GO:0030424);neuron projection(GO:0043005);perikaryon(GO:0043204);synapse(GO:0045202);cholinergic synapse(GO:0098981);integral component of postsynaptic specialization membrane(GO:0099060)	Nicotinic acetylcholine receptor signaling pathway->nicotinic acetylcholine receptor alpha;Nicotinic acetylcholine receptor signaling pathway->Nicotinic Acetylcholine Receptor;	
HUMAN|HGNC=13090|UniProtKB=Q8WUA7	TBC1D22A	TBC1 domain family member 22A	GTPase-activating protein(PC00257)	GTPase activator activity(GO:0005096);protein binding(GO:0005515);Rab GTPase binding(GO:0017137);protein homodimerization activity(GO:0042803);14-3-3 protein binding(GO:0071889)	intracellular protein transport(GO:0006886);activation of GTPase activity(GO:0090630)			
HUMAN|HGNC=23805|UniProtKB=Q76L83	ASXL2	Putative Polycomb group protein ASXL2		DNA binding(GO:0003677);chromatin binding(GO:0003682);protein binding(GO:0005515);peroxisome proliferator activated receptor binding(GO:0042975);metal ion binding(GO:0046872)	transcription, DNA-templated(GO:0006351);regulation of transcription, DNA-templated(GO:0006355);animal organ morphogenesis(GO:0009887);positive regulation of lipid storage(GO:0010884);protein deubiquitination(GO:0016579);positive regulation of peroxisome proliferator activated receptor signaling pathway(GO:0035360);positive regulation of fat cell differentiation(GO:0045600);positive regulation of transcription by RNA polymerase II(GO:0045944)	nuclear chromatin(GO:0000790);nucleus(GO:0005634);nucleoplasm(GO:0005654);PR-DUB complex(GO:0035517)		Post-translational protein modification;UCH proteinases;Deubiquitination;Metabolism of proteins
HUMAN|HGNC=9677	PTPRN2	Receptor-type tyrosine-protein phosphatase N2		protein tyrosine phosphatase activity(GO:0004725);transmembrane receptor protein tyrosine phosphatase activity(GO:0005001)	protein dephosphorylation(GO:0006470);lipid metabolic process(GO:0006629);neurotransmitter secretion(GO:0007269);negative regulation of GTPase activity(GO:0034260);peptidyl-tyrosine	endoplasmic reticulum lumen(GO:0005788);plasma membrane(GO:0005886);integral component of plasma membrane(GO:0005887);secretory granule(GO:0030141);transport vesicle membrane(GO:0030658);secretory granule membrane(GO:0030667);synaptic		Immune System;Innate Immune

Gene ID	Mapped ID	Gene name/symbol	Protein class	GO database MF complete	GO database BP complete	GO database CC complete	Pathway	Reactome pathway
|UniProtKB=Q92932					dephosphorylation(GO:0035335);insulin secretion involved in cellular response to glucose stimulus(GO:0035773);neutrophil degranulation(GO:0043312)	vesicle membrane(GO:0030672);terminal bouton(GO:0043195);receptor complex(GO:0043235);synapse(GO:0045202);ficolin-1-rich granule membrane(GO:0101003)		System;Neutrophil degranulation
HUMAN|HGNC=14295|UniProtKB=Q9UPX8	SHANK2	SH3 and multiple ankyrin repeat domains protein 2		protein binding(GO:0005515);SH3 domain binding(GO:0017124);synaptic receptor adaptor activity(GO:0030160);ionotropic glutamate receptor binding(GO:0035255)	synapse assembly(GO:0007416);behavior(GO:0007610);learning(GO:0007612);memory(GO:0007613);positive regulation of cell population proliferation(GO:0008284);adult behavior(GO:0030534);social behavior(GO:0035176);negative regulation of hippo signaling(GO:0035331);exploration behavior(GO:0035640);brain morphogenesis(GO:0048854);synaptic growth at neuromuscular junction(GO:0051124);positive regulation of synaptic transmission, glutamatergic(GO:0051968);long-term synaptic potentiation(GO:0060291);long-term synaptic depression(GO:0060292);dendritic spine morphogenesis(GO:0060997);positive regulation of dendritic spine development(GO:0060999);vocalization behavior(GO:0071625);postsynaptic density assembly(GO:0097107);maintenance of postsynaptic density structure(GO:0099562);regulation of AMPA receptor activity(GO:2000311);positive regulation of excitatory postsynaptic potential(GO:2000463)	photoreceptor outer segment(GO:0001750);photoreceptor inner segment(GO:0001917);cellular_component(GO:0005575);cytosol(GO:0005829);neurofilament(GO:0005883);plasma membrane(GO:0005886);ionotropic glutamate receptor complex(GO:0008328);postsynaptic density(GO:0014069);apical plasma membrane(GO:0016324);growth cone(GO:0030426);brush border membrane(GO:0031526);neuron projection(GO:0043005);neuronal cell body(GO:0043025);dendritic spine(GO:0043197);postsynaptic membrane(GO:0045211);ciliary membrane(GO:0060170)	Ionotropic glutamate receptor pathway->PSD95;	Protein-protein interactions at synapses;Neuronal System;Neurexins and neuroligins
HUMAN|HGNC=2549|UniProtKB=Q92879	CELF1	CUGBP Elav-like family member 1		translation repressor activity, mRNA regulatory element binding(GO:0000900);RNA binding(GO:0003723);mRNA binding(GO:0003729);mRNA 3'-UTR binding(GO:0003730);protein binding(GO:0005515);translation initiation factor binding(GO:0031369);pre-mRNA binding(GO:0036002);BRE binding(GO:0042835)	regulation of alternative mRNA splicing, via spliceosome(GO:0000381);mRNA splice site selection(GO:0006376);mRNA processing(GO:0006397);germ cell development(GO:0007281);spermatid development(GO:0007286);embryo development ending in birth or egg hatching(GO:0009792);positive regulation of gene expression(GO:0010628);negative regulation of gene expression(GO:0010629);positive regulation of cell death(GO:0010942);RNA interference(GO:0016246);posttranscriptional gene silencing(GO:0016441);positive regulation of multicellular organism growth(GO:0040018);regulation of RNA splicing(GO:0043484);regulation of inflammatory response(GO:0050727);mRNA destabilization(GO:0061157)	nucleus(GO:0005634);nucleoplasm(GO:0005654);cytoplasm(GO:0005737);cytoplasmic stress granule(GO:0010494);membrane(GO:0016020);perinucleolar compartment(GO:0097356);ribonucleoprotein complex(GO:1990904)		

Overlap of associations with treatment

References

1 Mi, H., Muruganujan, A., Ebert, D., Huang, X. & Thomas, P. D. PANTHER version 14: more genomes, a new PANTHER GO-slim and improvements in enrichment analysis tools. *Nucleic Acids Research* **47**, D419-D426 (2018).

2 Szklarczyk, D. *et al.* STRING v11: protein-protein association networks with increased coverage, supporting functional discovery in genome-wide experimental datasets. *Nucleic Acids Research* **47**, D607-D613 (2019).

Overlap of associations with treatment
