# Supplementary material for: DNA methylation changes following narrative exposure therapy in a randomized controlled trial with female former child soldiers
Source: Sci Rep. 2021 Sep 16;11:18493. doi: 10.1038/s41598-021-98067-9 (PMC8445994; doi:10.1038/s41598-021-98067-9)
Supplement: Supplementary file 5 — Supplementary Information 5. [file 41598_2021_98067_MOESM5_ESM.pdf]

DNA methylation changes following Narrative Exposure Therapy in a randomized controlled trial with female former child soldiers

Samuel Carleial, Daniel Nätt, Eva Unternährer, Thomas Elbert, Katy Robjant, Sarah Wilker, Vanja Vukojevic, Iris-Tatjana Kolassa, Anja C. Zeller, and Anke Koebach

Supplement S5

Summary results of replication tests.

Table S5 below shows all intersections between treatment and outcomes (per gene). Mean baseline DNAm of tested CpGs (n = 97) were similar between discovery (mean $\pm$ SD; 0.60 $\pm$ 0.24) and replication samples (0.56 $\pm$ 0.20). DNAm most commonly increased over time, with an average of 0.56% in $FU6_{TAU}$, 0.72% in $FU6_{NET}$ (discovery sample) and 0.95% in $FU10_{NET}$ (replication sample).

Replication of percentage change for treatment

Eleven out of 62 (22%) CpGs found in association with treatment were successfully replicated, namely cg06125671 (*ABTB2*) and cg22356726 (*KIF20B*) with the strongest replications (>1.5% percentage change), followed by cg12774902 (*CHRNA10*), cg03259703 (*IGSF21*), cg16662768 (*IRF8*), cg09102332 (*ITGB8*), cg15509069 (*SDHAF3*), cg04070142 (*SLCO1A2*), cg06665305 (*SOWAHC*), cg20401896 (*WDPCP*), cg26212904 (*WSCD1*) (>0.5% percentage change).

Replication of percentage change for traits

Regarding CpGs associated with clinical outcomes, cg18855674 (*EYA1*), cg14376987 (*KIF20B*), cg00961418 (*STK33*) and cg07395826 (*WDPCP*) were also replicated.

Summary numbers

62 genes enlisted

131 CpGs in total: 34 n.a.; 62 treatment; 39 trait; 4 overlapping between treatment + trait
131 CpGs in total: 34 n.a. + 97 tested (present in discovery and replication samples)

62 treatment associated CpGs
11 success (22%)
51 failure

97 tested (treatment + trait) CpGs
18 success
79 failure (19%)

Summary table

Table S5. List of associated CpGs and related genes that intersect between treatment and clinical/social outcome associations. For the discovery and replication samples, DNAm is represented as mean and standard deviation at baseline (BL), and as mean percentage change at 4, 6 or 10 month follow-ups (FU4-6-10, respectively) per treatment group (TAU vs NET). Discovery sample consists of female former ex-combatants affected by the M23 insurgency in Eastern DRC (N = 84) assessed at two timepoints. Replication sample consists of female former child soldiers affected by the LRA insurgency in Northern Uganda (N = 53) assessed at three timepoints. Only clinical outcomes could be replicated between studies: PTSD (PSS-I), depression (PHQ-9) and appetitive aggression (AAS). Successful percentage change replications are marked in bold and grouped by cut-offs (≤ 0.5% [·]; 0.5% [*]; 1.5% [**]; 5% [***]) and CpGs that were not tested or available are marked as “n.a.”.

**CpG**	**Gene symbol**	**Discovery sample**				**Replication sample**				
		**Association**	**DNAm mean (SD)**	**DNAm change**	**DNAm change**	**DNAm mean (SD)**	**DNAm change**		**DNAm change**	
			BL	$FU6_{TAU}$	$FU6_{NET}$	BL	$FU4_{NET}$		$FU10_{NET}$	
**cg06125671**	**ABTB2**	**treatment**	**0.44 (0.05)**	**-2.3%**	**7.4%**	**0.45 (0.042)**	**3.5%**	**	**3.3%**	**
cg10885779		PHQ-9	0.74 (0.071)	0.1%	0.3%	0.67 (0.066)	0.7%	·	0.5%	·
cg10034690	ACTG1	treatment	0.4 (0.053)	5.7%	-1.7%	0.38 (0.038)	-0.5%	·	1.2%	
cg25256099		PSS-I	0.64 (0.065)	1.1%	-1.7%	0.57 (0.063)	-0.9%	*	1.0%	
cg07544459	ADGRD1	treatment	0.72 (0.057)	2.6%	0.7%	0.66 (0.047)	-0.8%		-1.0%	
cg00633768		PSS-I	0.81 (0.035)	0.6%	1.2%	0.68 (0.042)	0.7%	*	0.5%	·
cg21964649		AAS	0.78 (0.041)	0.6%	0.9%	0.72 (0.033)	1.0%	*	0.3%	·
cg23719209	ALCAM	treatment/PHQ-9	0.8 (0.033)	1.3%	-2.3%	0.7 (0.032)	-0.2%	·	1.1%	
cg23619970	ARHGAP10	treatment	0.64 (0.069)	-1.8%	6.6%		n.a.			
cg20866785		AAGS	0.86 (0.031)	-0.3%	0.5%		n.a.			
cg01738095	ASXL2	treatment	0.75 (0.055)	1.3%	-2.8%	0.62 (0.052)	-0.5%	·	0.5%	
cg02562678		PSS-I	0.68 (0.06)	-2.0%	-1.2%	0.55 (0.044)	1.1%		3.3%	
cg19729930	BOLA3	treatment	0.47 (0.11)	3.8%	-0.2%	0.46 (0.13)	-0.5%	·	1.0%	
cg00411544		SAQ	0.16 (0.043)	-1.9%	2.5%		n.a.			
cg06405860	C1orf87	treatment	0.24 (0.025)	3.4%	-3.4%	0.3 (0.025)	2.3%		1.3%	
cg24740868		PHQ-9	0.2 (0.033)	1.9%	0.7%	0.32 (0.025)	-1.0%		0.4%	·
cg13786031	C9orf47	treatment	0.76 (0.037)	-2.0%	1.0%	0.65 (0.035)	-1.2%		0.1%	·
cg27301893		PSS-I	0.64 (0.045)	1.7%	1.0%	0.6 (0.035)	0.0%		-0.4%	
cg09451427	CACNA2D2	treatment/SAQ	0.29 (0.071)	-5.4%	8.0%	0.41 (0.088)	-0.1%		1.2%	*
cg13033387	CDH5	treatment	0.15 (0.022)	-3.3%	5.5%	0.17 (0.023)	-0.5%		1.1%	*
cg19944059		SAQ	0.21 (0.055)	-1.0%	1.7%		n.a.			
cg19146902	CELF1	treatment	0.68 (0.042)	-1.6%	3.5%	0.64 (0.043)	-0.1%		0.7%	*
cg23337648		CVB	0.59 (0.049)	2.2%	1.6%		n.a.			
cg00056497	CFAP46	treatment	0.59 (0.073)	3.5%	-0.4%	0.55 (0.044)	0.0%		-0.6%	·
cg14667769		treatment	0.86 (0.021)	1.1%	-0.7%	0.78 (0.024)	0.1%		-0.7%	*
cg27264384		AAS	0.86 (0.042)	1.1%	0.8%	0.81 (0.049)	0.0%		0.0%	
cg16551240		CVB	0.89 (0.024)	0.6%	0.3%		n.a.			
**cg12774902**	**CHRNA10**	**treatment**	**0.79 (0.037)**	**-1.0%**	**0.9%**	**0.67 (0.033)**	**1.5%**	*	**1.5%**	*
cg24969467		SAQ	0.82 (0.026)	-0.6%	0.5%		n.a.			
cg14015300	COX6B2	treatment	0.63 (0.032)	2.2%	-0.5%	0.57 (0.031)	1.4%		1.0%	
cg26562532		AAGS	0.85 (0.022)	0.6%	1.3%		n.a.			
cg16967191	CPLX2	treatment	0.46 (0.063)	3.2%	-5.3%	0.38 (0.057)	0.1%		2.9%	
cg01284438		AAGS	0.76 (0.05)	0.8%	0.2%		n.a.			
cg14422899	DICER1	treatment	0.82 (0.026)	-0.6%	1.7%	0.74 (0.029)	-1.7%		-0.7%	
cg00793719		CVB	0.51 (0.057)	0.5%	1.1%		n.a.			
cg22607472	DPF3	treatment	0.76 (0.029)	-1.2%	1.5%	0.71 (0.03)	0.0%		0.5%	·
cg12816088		AAS	0.77 (0.055)	2.2%	0.2%	0.73 (0.051)	0.7%	·	0.0%	
cg07313064	EGFR	treatment	0.85 (0.029)	-1.2%	1.1%	0.75 (0.048)	-0.2%		0.2%	·
cg19083626		PHQ-9	0.84 (0.038)	-0.8%	0.0%	0.72 (0.053)	-0.1%		0.3%	·
cg06562969	EPSTI1	treatment	0.79 (0.041)	2.5%	-1.2%	0.65 (0.081)	-0.4%	·	0.8%	
cg26714230		PHQ-9	0.12 (0.043)	-0.7%	-4.6%	0.12 (0.022)	2.2%		5.5%	
cg16939412	EVI5	treatment	0.67 (0.04)	-2.6%	2.0%	0.63 (0.05)	-0.9%		0.2%	·
cg19032370		SAQ	0.82 (0.025)	0.0%	0.7%		n.a.			
cg22348290	EYA1	treatment	0.63 (0.042)	3.6%	-3.0%	0.55 (0.043)	0.6%		-1.5%	**
cg18855674		PHQ-9	0.084 (0.028)	3.2%	2.3%	0.12 (0.03)	2.3%	**	5.9%	**
cg23784400	GATAD2A	treatment/AAGS	0.87 (0.026)	1.1%	-1.1%	0.78 (0.031)	1.3%		0.5%	
cg25645198	GK	treatment	0.8 (0.036)	-0.6%	1.9%		n.a.			
cg08470157		AAS	0.34 (0.073)	-3.8%	-4.8%		n.a.			
cg20019489	GNAS	treatment	0.74 (0.042)	3.0%	-0.7%	0.63 (0.048)	-0.1%	·	-0.5%	·
cg14176797		SAQ	0.59 (0.029)	0.3%	-0.5%		n.a.			
cg04417860	GPX5	treatment	0.37 (0.054)	6.2%	-2.8%	0.32 (0.035)	-0.6%	*	2.2%	
cg01204927		AAS	0.32 (0.041)	2.0%	0.9%	0.29 (0.036)	2.0%	*	2.7%	*

CpG	Gene symbol	Discovery sample				Replication sample				
		Association	DNAm mean (SD)	DNAm change	DNAm change	DNAm mean (SD)	DNAm change		DNAm change	
cg14794991	GRID1	treatment	0.73 (0.038)	1.3%	-1.8%	0.62 (0.035)	0.3%		-0.1%	·
cg16375547		PHQ-9	0.72 (0.036)	-0.4%	-1.1%	0.61 (0.033)	1.6%		1.8%	
**cg03259703**	**IGSF21**	**treatment**	**0.85 (0.024)**	**0.4%**	**-1.1%**	**0.76 (0.042)**	**-0.8%**	*	**-0.7%**	*
cg07262842		CVB	0.53 (0.079)	-3.4%	-0.1%		n.a.			
cg19726666		CVB	0.46 (0.086)	-3.2%	-2.7%		n.a.			
cg02636808	INPP5A	treatment	0.66 (0.049)	4.4%	-0.8%	0.66 (0.037)	1.1%		0.2%	
cg23103009		PSS-I	0.86 (0.029)	0.6%	0.2%	0.78 (0.028)	1.2%	·	0.2%	·
**cg16662768**	**IRF8**	**treatment**	**0.42 (0.07)**	**-4.4%**	**1.2%**	**0.46 (0.058)**	**0.7%**	*	**0.6%**	*
cg27507473		CVB	0.41 (0.084)	8.3%	6.0%		n.a.			
**cg09102332**	**ITGB8**	**treatment/AAS**	**0.55 (0.13)**	**-3.4%**	**1.8%**	**0.41 (0.084)**	**1.0%**	*	**2.6%**	**
**cg22356726**	**KIF20B**	**treatment**	**0.11 (0.066)**	**-10.0%**	**3.3%**	**0.15 (0.077)**	**2.7%**	**	**3.8%**	**
cg11732055		PSS-I	0.81 (0.034)	0.0%	-0.5%	0.75 (0.031)	0.9%		0.0%	·
cg14376987		PSS-I	0.2 (0.029)	-1.5%	7.4%	0.18 (0.033)	5.6%	***	10.0%	***
cg18452703	LBX1	treatment	0.82 (0.029)	-1.3%	1.5%	0.72 (0.047)	-0.7%		0.0%	·
cg10996596		AAGS	0.075 (0.029)	2.5%	-4.2%		n.a.			
cg24107728	LRP8	treatment/AAS	0.8 (0.041)	3.0%	-0.2%	0.75 (0.036)	0.8%		0.6%	
cg09314675	LRRC28	treatment	0.81 (0.031)	2.3%	-0.3%	0.74 (0.03)	0.7%		-0.9%	·
cg11433098		PHQ-9	0.47 (0.051)	1.4%	-0.3%	0.46 (0.035)	0.5%		-0.4%	·
cg16285941	LY6H	treatment	0.55 (0.036)	3.1%	-0.1%	0.53 (0.028)	0.5%		-0.2%	·
cg22522939		CVB	0.89 (0.036)	0.7%	-0.1%		n.a.			
cg02201051		CVB	0.83 (0.048)	1.5%	0.6%		n.a.			
cg11389565	MAP2K4	treatment	0.89 (0.018)	0.8%	-0.6%	0.8 (0.025)	0.8%		0.5%	
cg25629796		SAQ	0.86 (0.03)	0.2%	-0.3%		n.a.			
cg20776758	MED13L	treatment	0.64 (0.05)	3.8%	-1.0%	0.56 (0.041)	-0.1%	·	-1.6%	*
cg08800242		PSS-I	0.89 (0.022)	-0.1%	0.8%	0.81 (0.031)	-0.3%		-1.1%	
cg21535156	MICALL2	treatment	0.44 (0.037)	1.8%	-2.5%	0.43 (0.045)	1.1%		-1.2%	*
cg10875499		treatment	0.78 (0.049)	-0.3%	3.6%	0.76 (0.038)	-1.2%		-0.5%	
cg02759751		PSS-I	0.92 (0.035)	1.3%	0.0%	0.87 (0.026)	-0.5%	·	-1.1%	·
cg08401219		CVB	0.87 (0.027)	0.5%	0.7%		n.a.			
cg11382589	MTRNR2L1	treatment	0.62 (0.053)	-0.9%	1.1%	0.57 (0.035)	0.2%	·	0.9%	*
cg07342674		AAGS	0.72 (0.035)	0.7%	1.3%		n.a.			
cg07841848	NKX2-5	treatment	0.81 (0.035)	1.4%	-0.3%	0.71 (0.032)	0.6%		0.3%	
cg05234035		PHQ-9	0.22 (0.046)	-2.2%	1.3%	0.29 (0.056)	3.1%	*	7.6%	*
cg03486475	PCDHGA4	treatment	0.64 (0.065)	4.8%	0.2%	0.57 (0.057)	0.4%	·	1.4%	·
cg00808170		AAS	0.5 (0.032)	0.5%	0.8%	0.51 (0.027)	1.0%	*	0.6%	*
cg21580428		AAS	0.11 (0.025)	-4.6%	-3.6%	0.16 (0.027)	-0.2%	·	2.9%	
cg08713851	PNPLA7	treatment	0.87 (0.058)	1.6%	-0.7%	0.81 (0.066)	-0.2%	·	-0.1%	·
cg16466201		CVB	0.86 (0.04)	0.7%	0.7%		n.a.			
cg15623260		AAGS	0.88 (0.026)	1.0%	0.2%		n.a.			
cg17291136	PPP2R2A	treatment	0.75 (0.032)	-1.8%	1.8%	0.64 (0.028)	-1.1%		-0.4%	
cg25247998		AAGS	0.82 (0.032)	-0.1%	0.5%		n.a.			
cg14211350	PTPRN2	treatment	0.56 (0.035)	-1.1%	2.6%	0.53 (0.044)	-0.1%		1.4%	*
cg24241410		PSS-I	0.84 (0.026)	0.5%	0.2%	0.76 (0.033)	0.8%	·	0.3%	·
cg19205240	RELN	treatment	0.82 (0.031)	1.4%	-0.7%	0.73 (0.032)	-1.0%	*	-0.5%	·
cg11912608		AAGS	0.73 (0.052)	2.0%	1.8%		n.a.			
**cg15509069**	**SDHAF3**	**treatment**	**0.57 (0.025)**	**-0.7%**	**0.7%**	**0.53 (0.019)**	**0.9%**	*	**1.1%**	*
cg08438525		PSS-I	0.51 (0.085)	0.8%	-0.3%	0.43 (0.078)	-1.8%	·	1.4%	
cg05684381	SHANK2	treatment	0.66 (0.038)	-1.8%	2.6%	0.68 (0.054)	0.4%	·	1.6%	**
cg04262428		PSS-I	0.14 (0.057)	3.3%	7.2%	0.3 (0.069)	-2.3%		-3.1%	
cg14851284		PHQ-9	0.77 (0.031)	0.2%	-0.3%	0.68 (0.02)	1.4%		1.1%	
cg23903396		SAQ	0.38 (0.043)	0.1%	1.1%		n.a.			
cg27434326	SIX2	treatment	0.69 (0.069)	1.3%	-1.2%	0.59 (0.079)	-0.4%	·	0.8%	
cg11358114		SAQ	0.61 (0.061)	-2.3%	-1.1%		n.a.			
**cg04070142**	**SLCO1A2**	**treatment**	**0.84 (0.024)**	**1.2%**	**-0.5%**	**0.8 (0.028)**	**-0.6%**	*	**-0.9%**	*
cg19659215		SAQ	0.72 (0.041)	1.4%	1.5%		n.a.			
cg18390345	SLIT1	treatment	0.49 (0.053)	3.9%	0.5%	0.45 (0.045)	-1.3%		0.3%	·
cg06157313		PHQ-9	0.87 (0.025)	0.2%	0.5%	0.79 (0.023)	0.7%	*	0.4%	·
cg01056004		AAGS	0.32 (0.08)	23.0%	4.3%		n.a.			
**cg06665305**	**SOWAHC**	**treatment/PSS-I**	**0.23 (0.043)**	**4.0%**	**-1.1%**	**0.31 (0.056)**	**-1.8%**	*	**-2.3%**	*
cg11664139	SP9	treatment	0.38 (0.051)	-6.8%	2.4%	0.33 (0.039)	-3.0%		1.1%	*
cg19049194		AAS	0.27 (0.067)	-5.1%	-1.4%	0.24 (0.06)	-0.7%	*	8.1%	
cg27431761	STK33	treatment	0.24 (0.078)	-1.7%	13.0%	0.4 (0.094)	-0.2%		-1.2%	
cg00961418		PSS-I	0.16 (0.052)	1.5%	6.4%	0.15 (0.031)	0.8%	*	7.9%	***
cg03490719	SYT7	treatment	0.82 (0.024)	1.2%	0.0%	0.74 (0.018)	1.2%		0.8%	
cg15167955		SAQ	0.81 (0.034)	-0.1%	-0.1%		n.a.			
cg20398969	TBC1D22A	treatment	0.63 (0.06)	1.7%	-0.3%	0.58 (0.042)	1.0%		1.0%	
cg10140974		CVB	0.81 (0.065)	2.1%	1.2%		n.a.			
cg21279806	THBS2	treatment	0.084 (0.019)	-5.6%	14.0%	0.16 (0.027)	-0.6%		4.0%	**
cg02768671		PSS-I	0.75 (0.063)	3.1%	2.1%	0.71 (0.049)	0.8%	*	-1.1%	
cg00199091	TRERF1	treatment	0.51 (0.066)	5.8%	-0.8%	0.42 (0.045)	1.0%		0.6%	

CpG	Gene symbol	Discovery sample				Replication sample				
		**Association**	**DNAm mean (SD)**	**DNAm change**	**DNAm change**	**DNAm mean (SD)**	**DNAm change**		**DNAm change**	
cg01673485		PHQ-9	0.77 (0.027)	0.7%	0.8%	0.69 (0.033)	0.2%	·	1.0%	*
cg19791321	TRIB1	treatment	0.84 (0.066)	2.2%	-0.5%	0.78 (0.049)	1.4%		0.2%	
cg07043360		AAS	0.24 (0.034)	1.3%	-0.6%	0.23 (0.025)	0.1%		0.1%	
**cg20401896**	**WDPCP**	**treatment**	**0.76 (0.03)**	**-2.2%**	**0.7%**	**0.68 (0.03)**	**0.8%**	*	**0.8%**	*
cg07395826		AAS	0.26 (0.065)	8.8%	8.0%	0.31 (0.058)	1.1%	*	4.0%	**
**cg26212904**	**WSCD1**	**treatment**	**0.77 (0.045)**	**1.7%**	**-2.6%**	**0.65 (0.054)**	**-0.8%**	*	**-1.1%**	*
cg04525002		PHQ-9	0.87 (0.025)	0.3%	0.5%	0.81 (0.029)	0.2%	·	-0.2%	
cg09392245	ZNF263	treatment	0.83 (0.027)	-1.3%	1.0%	0.73 (0.041)	-0.1%		2.0%	*
cg01548742		CVB	0.85 (0.033)	1.6%	1.1%			n.a.		
